# Atroposelective Access to π‐Conjugated 1,2‐Azaborepines Enabled by Palladium‐Catalyzed Cyclization of *N*‐Heterobiaryls with Alkynylboronates

**DOI:** 10.1002/advs.75755

**Published:** 2026-05-19

**Authors:** Fengya He, Zhen Wang, Haoyu Guo, Shuguang Chen, Xu Zhang, Yongjia Shang, Hui Wang

**Affiliations:** ^1^ Anhui Key Laboratory of Molecular‐Based Materials College of Chemistry and Materials Science Key Laboratory of Functional Molecular Solids (Ministry of Education) Anhui Normal University Wuhu China

**Keywords:** 1,2‐Migration, DyKAT, medium‐sized ring, *N*‐Heterobiaryl triflates, π‐Conjugated organoborons

## Abstract

π‐Conjugated organoboron compounds that feature N→B coordination bonds exhibit attractive photophysical and electronic properties. However, the efficient catalytic asymmetric synthesis of medium‐sized BN‐heterocycles remains underdeveloped. Herein, we report a palladium‐catalyzed atroposelective cyclization of racemic *N*‐heterobiaryl triflates with alkynyl boronates, yielding a variety of 1,2‐BN‐bridged seven‐membered biaryls in high yields (up to 99%) and excellent enantioselectivity (>99% ee). The reaction occurs via a palladium‐catalyzed dynamic kinetic asymmetric transformation (DyKAT) of *N*‐heterobiaryl triflates to generate a palladium species, which then undergoes *cis*‐carbopalladation with the alkyne moiety of alkynylboronates, followed by a 1,2‐migration to afford the chiral π‐conjugated 1,2‐azaborepines. The reaction shows broad substrate scope and excellent functional group tolerance. And the resulting boron‐containing products are amenable to further transformations, including direct oxidation and Suzuki–Miyaura cross‐couplings.

## Graphical abstract

We report a palladium‐catalyzed atroposelective cyclization of *N*‐heterobiaryl triflates with alkynyl boronates to access seven‐membered 1,2‐BN‐bridged biaryls with high yields and excellent enantioselectivity via a cascade DyKAT, *cis*‐carbopalladation, and 1,2‐migration, which expands the toolbox for axially chiral π‐conjugated boron compounds and opens new avenues for BN‐embedded functional materials.

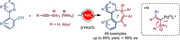

## 1 Introduction

**SCHEME 1:**
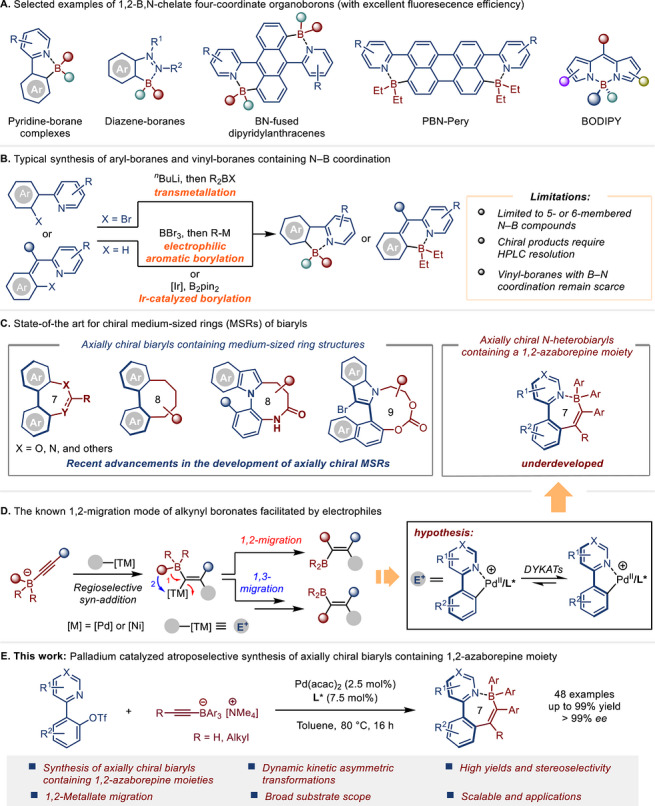
π‐Conjugated boron compounds and 1,2‐migrations of alkynylborates induced by electrophiles.

π‐Conjugated boron compounds have attracted widespread attention as a versatile class of materials because of their exceptional electronic and photophysical properties [1, 2, 3, 4, 5, 6, 7]. The presence of intramolecular N→B coordinate bonds typically could extend the π‐conjugation system and lower the LUMO energy level, thereby enhancing the electron affinity and endowing the compounds with strong fluorescence and photochromic properties [8, 9, 10, 11, 12, 13, 14]. As a result, a wide variety of π‐conjugated boron frameworks, including pyridine boranes, diazene boranes, and boron dipyrromethene (BODIPY) cores, have been extensively studied, with applications in organic electronics, optoelectronics, and functional dyes (Scheme 1A) [15, 16, 17, 18, 19, 20, 21, 22, 23]. Therefore, the development of effective synthetic routes to such frameworks remains a key goal in modern synthetic chemistry. For aryl‐based N→B ladder‐type boranes, synthetic strategies have primarily relied on the directed *ortho*‐metalation–borylations [24, 25, 26], electrophilic aromatic borylations [27, 28, 29, 30], or transition metal‐catalyzed C─H borylations [31, 32, 33], which have successfully afforded various fluorescent arylboranes bearing five‐ or six‐membered N→B chelating rings (Scheme 1B). Of note, their chiral congeners [34, 35, 36, 37, 38], which are promising candidates for materials exhibiting unique chiroptical properties such as circularly polarized luminescence (CPL), continue to pose synthetic challenges. The enantioenriched forms are typically obtained by preparative chiral HPLC resolution; to the best of our knowledge, however, no example of *de novo* asymmetric synthesis has been reported to date. Furthermore, although π‐conjugated boron compounds containing five‐ or six‐membered N→B rings have been well established, their medium‐sized BN‐heterocyclic analogues, such as 1,2‐azaborepines, only limited examples have been reported [39, 40], mainly stemming from the lack of efficient synthesis methods.

Axially chiral medium‐sized rings have attracted considerable attention in recent years, and diverse synthetic strategies, including post‐synthetic modification [41, 42, 43, 44, 45, 46, 47, 48], ring expansion [49, 50], (dynamic) kinetic resolution [51, 52, 53], cyclization [54, 55, 56, 57, 58, 59, 60, 61, 62, 63], and cycloaddition [64, 65], have been developed to access these scaffolds. Nevertheless, current methods remain confined mainly to the preparation of atropisomeric biaryls linked by C─C, C─O, and C─N bonds, whereas no synthetic methods have been reported for such medium‐sized BN‐heterocycles (Scheme 1C). We envision that integrating π‐conjugated organoboron units during the construction of axially chiral *N*‐heterobiaryl frameworks would offer a viable strategy to access axially chiral medium‐sized BN‐heterocycles, such as axially chiral 1,2‐azaborinines. This approach would thereby not only open a new avenue to chiral medium‐sized π‐conjugated boron compounds but also potentially endow the resulting materials with unique physicochemical properties.

Our synthetic strategy is based on the migration chemistry of alkynyl boronates, which can serve as versatile intermediates for the stereoselective access to multi‐substituted alkenes via electrophile‐induced 1,2‐ or 1,3‐metallate shifts [66, 67, 68, 69, 70, 71, 72, 73]. Notably, transition metal‐complexes, such as Pd(II), Ir(III), and Ni(II), can act as electrophiles to induce these migrations in a catalytic and highly enantioselective manner [74, 75, 76, 77, 78, 79, 80, 81, 82, 83]. In this context, we recognized that *N*‐heterobiaryl transition metal‐complexes, known to enable axial chirality control in *N*‐heterobiaryls via dynamic kinetic asymmetric transformations (DyKATs) [84, 85, 86, 87, 88, 89, 90, 91, 92, 93, 94, 95, 96, 97, 98, 99, 100], could also function as the electrophile to induce the migrations of alkynyl boronates. This thereby facilitates the construction of the axially chiral, seven‐membered 1,2‐BN‐biaryl compounds (Scheme 1D). However, to achieve the Pd‐catalyzed atroposelective reactions, several inherent challenges need to be addressed: (1) how to avoid the direct alkynylation via the transmetalation of alkynyl boronic esters with *N*‐heterobiaryl Pd(II)‐complexes? (2) How to achieve the high stereoselectivity and regioselectivity (1,2‐migration vs. 1,3‐migration) of the desired 1,2‐azaborepines? (3) How to ensure the formation of the alkenyl borane in the configuration required for stable N→B coordination and avoid the protodeboronation? Herein, we report an unprecedented palladium‐catalyzed enantioselective cyclization of *N*‐heterobiaryl triflates with alkynyl boronates, resulting in a variety of 1,2‐BN‐bridged seven‐membered biaryl compounds in high yields and with excellent enantioselectivities (Scheme 1E). The reaction proceeds through palladium‐catalyzed DyKAT of *N*‐heterobiaryl triflates to generate a chiral palladium species, which then undergoes sequential *cis*‐carbopalladation with the alkyne moiety of alkynylboronates, followed by a 1,2‐migration to afford the chiral π‐conjugated 1,2‐azaborepines. The approach demonstrates broad substrate scope and excellent functional group tolerance, providing a versatile route to valuable axially chiral π‐conjugated organoboron compounds.

## 2 Results and Discussion

To evaluate the feasibility of the designed Pd‐catalyzed atroposelective cyclization, initial experiments were conducted using racemic 1‐(isoquinolin‐1‐yl)naphthalen‐2‐yl trifluoromethanesulfonate (**1a**) and alkynyl boronate **2a** as model substrates (Table 1). To our delight, when the reaction was carried out with [Pd(allyl)Cl]_2_ and the chiral ligand (*R*)‐**L1**, the desired seven‐membered 1,2‐BN bridged biaryl product **3** was obtained in 92% yield and with 93% ee (entry 1). The use of the enantiomeric ligand (*S*)‐**L1** also led to a similarly favorable result (entry 2). The use of (*R*)‐**L2** as the ligand resulted in improved outcomes, yielding product **3** in 94% yield and 94% ee (entry 3). In contrast, the more sterically hindered ligand (*R*)‐**L3** failed to deliver any of the desired product (entry 4). The binaphthyl‐based bisphosphine ligand (*R*)‐**L4** afforded comparable reactivity with even higher enantioselectivity (98% ee) (entry 5). Further screening of reaction temperatures established 80°C as the optimal condition (entries 5–8). Notably, the reaction maintained high efficiency even when the catalyst loading of [Pd(allyl)Cl]_2_ was reduced to 2.5 mol% (entry 9). Moreover, several other palladium catalysts, including Pd(OAc)_2_, Pd(dba)_2_, and Pd(acac)_2_, were evaluated, all of which furnished the product **3** in high yields with excellent enantioselectivity (98–99% ee) (entries 10–12). Among them, Pd(acac)_2_ afforded nearly quantitative conversion and 99% ee; thus, it was selected as the optimal catalyst, delivering **3** in 96% isolated yield. Screening of various solvents, such as THF, CH_3_CN, and DMF, showed that, although high enantioselectivity was maintained in all cases, none surpassed toluene in terms of reaction efficiency (entries 13–15). Control experiments confirmed that both the palladium catalyst and chiral ligand are essential for the success of the transformation (entries 16–17).

**TABLE 1:** Optimization of the Pd‐catalyzed atroposelective cyclization.^a^

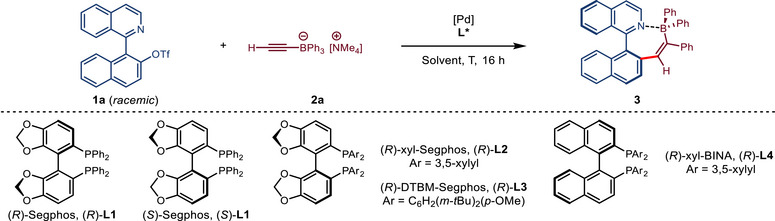						
Entry	[Pd] / 5.0 mol %	L* / 15 mol %	T / °C	Solvent	Yield of 3 / %^b^	*Ee* / %^c^
1	[Pd(allyl)Cl]_2_	*(R)‐* **L1**	80	Toluene	92	93
2	[Pd(allyl)Cl]_2_	*(S)‐* **L1**	80	Toluene	83	−90
3	[Pd(allyl)Cl]_2_	*(R)‐* **L2**	80	Toluene	94	94
4	[Pd(allyl)Cl]_2_	*(R)‐* **L3**	80	Toluene	Trace	—
5	[Pd(allyl)Cl]_2_	*(R)‐* **L4**	80	Toluene	94	98
6	[Pd(allyl)Cl]_2_	*(R)‐* **L4**	25	Toluene	18	82
7	[Pd(allyl)Cl]_2_	*(R)‐* **L4**	60	Toluene	92	90
8	[Pd(allyl)Cl]_2_	*(R)‐* **L4**	100	Toluene	80	93
9^d^	[Pd(allyl)Cl]_2_	*(R)‐* **L4**	80	Toluene	93	98
10^d^	Pd(OAc)_2_	*(R)‐* **L4**	80	Toluene	93	98
11^d^	Pd(dba)_2_	*(R)‐* **L4**	80	Toluene	80	99
**12** ^d^	**Pd(acac)_2_**	***(R)‐*L4**	**80**	**Toluene**	**99 (96)** ^e^	**99**
13^d^	Pd(acac)_2_	*(R)‐* **L4**	80	THF	65	98
14^d^	Pd(acac)_2_	*(R)‐* **L4**	80	CH_3_CN	30	97
15^d^	Pd(acac)_2_	*(R)‐* **L4**	80	DMF	46	97
16	—	*(R)‐* **L4**	80	toluene	n.d.	—
17^d^	Pd(acac)_2_	—	80	toluene	43	0

^a^ Reaction conditions: **1a** (0.2 mmol, 1.0 equiv.), **2a** (0.3 mmol, 1.5 equiv.), [Pd] (5.0 mol %), and L* (15.0 mol %) in toluene (2.0 mL), 16 h, N_2_.

^b^ Yields were determined by ^1^H NMR analysis using 1,3,5‐trimethoxybenzene as an internal standard.

^c^ The ee values were determined by chiral HPLC analysis.

^d^ [Pd] (2.5 mol %) and L* (7.5 mol %)

^e^ Product **3** was obtained with an isolated yield given in parentheses. *Ee* = Enantiomeric excess. DMF = *N,N*‐Dimethylformamide. n.d. = not detected.

**SCHEME 2:**
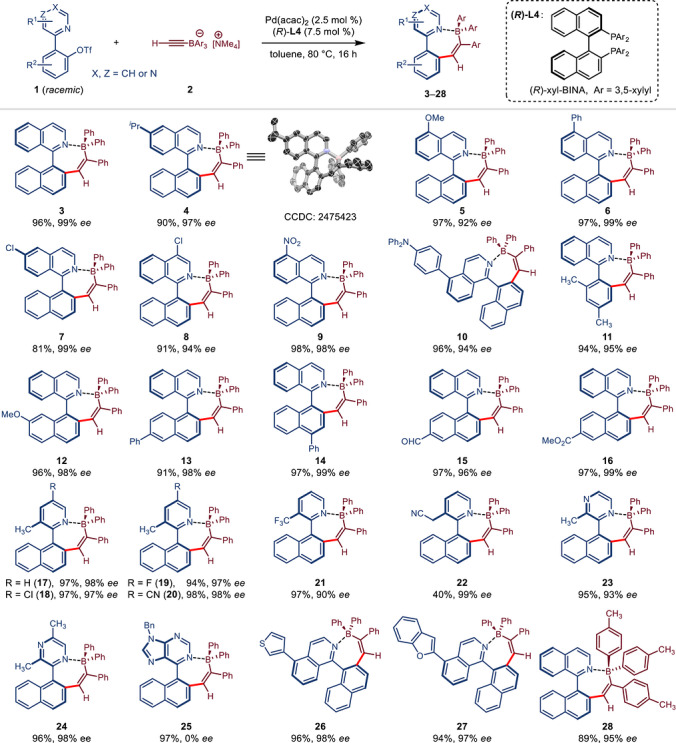
Substrate scope for *N*‐heterobiaryl triflates.

Having established the optimal reaction conditions, we next evaluated the scope of this palladium‐catalyzed DyKAT of racemic *N*‐heterobiaryl triflates using ethynyltriarylborates **2** as the coupling partners (Scheme 2). The reaction demonstrated excellent compatibility with a broad range of substituents on the *N*‐heterobiaryl triflate scaffolds. For instance, isoquinoline rings bearing both electron‐donating and electron‐withdrawing substituents at the C4–C6 positions were all well accommodated, affording the corresponding products **3–10** in excellent yields (81–99%) with excellent enantioselectivities (92 to >99% ee). The DFT‐calculated rotational barrier of compound **3** is 39.5 kcal/mol, and a similar ee value was observed even after stirring at 160 °C in toluene for 16 h (see Tables S10 and S13 for more details), confirming the high configurational stability of this compound. The absolute configuration of the enantiomerically enriched seven‐membered 1,2‐BN bridged biaryl product **4** was determined by x‐ray crystallography [101]. Similarly, substitution at various sites of the naphthalene ring was also feasible, delivering products **11–16** in 91–97% yields and with 95–99% ee. Moreover, the substrate scope was also successfully extended to other *N*‐heterocyclic frameworks, including pyridine and pyrazine derivatives that underwent efficient asymmetric transformation to afford products **17–24** in good yields with high ee. Notably, the reaction proved compatible with a wide array of functional groups, such as methoxy (**5**, **12**), halides (**7**, **8**, **18**, **19**), nitro (**9**), amino (**10**), formyl (**15**), ester (**16**), trifluoromethyl (**21**), and cyano (**20**, **22**) moieties. Interestingly, while an *N*‐purine‐derived substrate provided the corresponding product in 97% yield, no enantioselectivity was observed (**25**). This is presumably due to the absence of a substituent on the nitrogen atom of the C═N double bond in the purine ring, combined with its five‐membered ring structure, which results in a lower steric hindrance and consequently a low rotational barrier, leading to facile racemization under the reaction conditions. Substrates in which the isoquinoline ring is substituted with other heteroaromatic groups, such as thiophene and benzofuran, were also compatible, furnishing products **26** and **27** in 96% and 98% yields, with 98% and 97% ee, respectively. Finally, the 4‐methyl‐substituted aryl ethynylboronate was also identified as a viable substrate, yielding product **28** in 89% yield and 95% ee.

**SCHEME 3:**
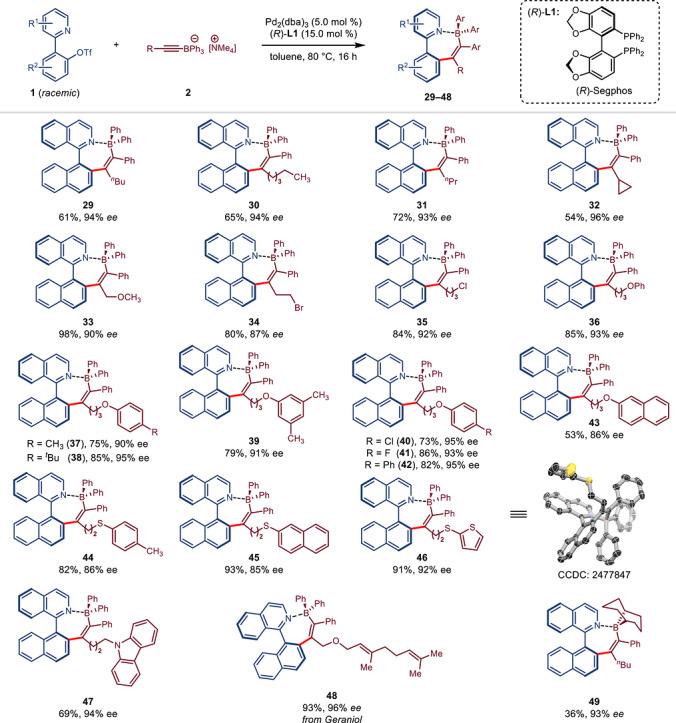
Substrate scope for internal alkynyl boronates.

Building upon the success of the enantioselective reactions with ethynyltriarylborates, we then sought to explore the reactivity of alkyl‐substituted alkynyl boronates. However, when the above optimized conditions were applied to the coupling of **1a** with alkyl‐substituted alkynyl boronate **2c**, no desired product was observed. Subsequent reaction optimization revealed that employing Pd_2_(dba)_3_ as the catalyst and (*R*)‐**L1** as the ligand afforded the target product **29** in 61% isolated yield with 94% ee (see the Tables S6–S8 for details). Under these modified conditions, the scope of alkyl‐substituted alkynyl boronates was then investigated (Scheme 3). A range of alkyl‐substituted alkynyl boronates, including those bearing sterically demanding groups such as isopropyl and cyclopropyl, all effectively coupled with **1a** to furnish products **29–35** in good yields and with excellent enantioselectivity (93–96% ee). Furthermore, various aryl ether‐containing substrates containing both electron‐donating and electron‐withdrawing substituents on the phenyl ring proved compatible under the standard conditions, delivering products **36–42** in moderate to good yields (73%–86%) and with high enantioselectivity (90% to 95% ee). The 2‐naphthyl‐substituted substrate was also tolerated, affording axially chiral product **43** in 53% yield and moderate ee. Moreover, the catalytic system proved effective for thioether‐containing substrates, yielding the desired products **44–46** in high yields and good enantioselectivity. The absolute configuration of enantiomerically enriched compound **46** was assigned by x‐ray crystallography [102]. Finally, carbazole‐ and geraniol‐derived substrates proved both compatible with the standard catalytic system, delivering products **47** and **48** in 69% and 98% yield, with 94% and 90% ee, respectively. Moreover, the alkynyl‐9‐BBN substrate, generated in situ from phenyl‐9‐borabicyclo[3.3.1]nonane (Ph‐9‐BBN) and alkynyllithium, also participated successfully in the reaction, affording product **49** in an acceptable yield and 93% ee.

**SCHEME 4:**
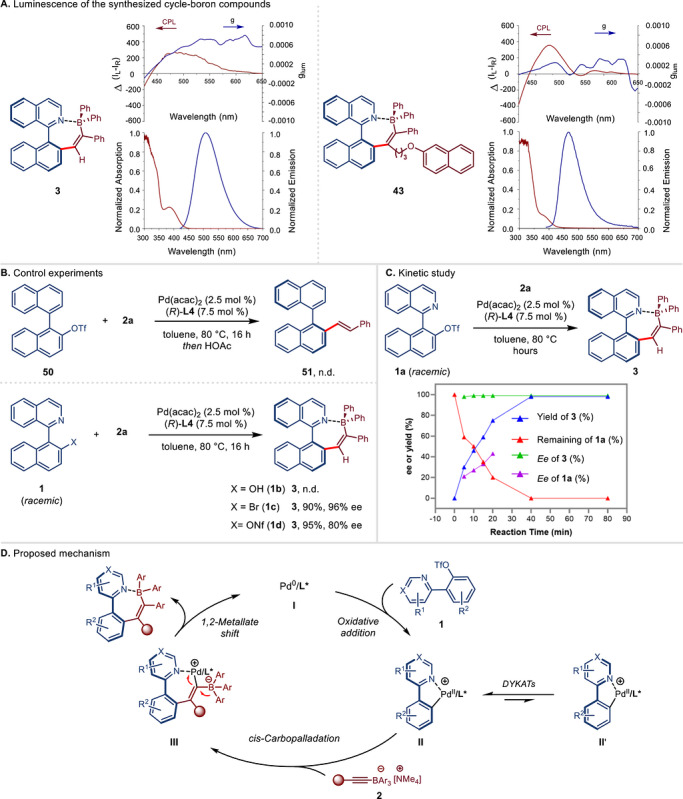
Photophysical property investigation and mechanistic studies.

The photophysical and chiroptical properties of the representative axially chiral π‐conjugated 1,2‐azaborepines **3** and **43** were investigated in acetonitrile (Scheme 4A). The absorption and emission maxima for **3** and **43** were observed at 385/501 nm (Φ = 0.67%) and 383/464 nm (Φ = 0.11%), respectively. Notably, strong positive circularly polarized luminescence (CPL) was observed for both compounds, with peaks at approximately 482 nm for compound **3** and 469 nm for compound **43**. The respective luminescence dissymmetry factors (g_lum_) were 5.7 × 10^−3^ and 1.7 × 10^−4^. These results demonstrate the strong potential of such axially chiral molecules for developing new organic chiroptical functional materials. Moreover, several control experiments were next designed to probe the reaction mechanism. Under standard conditions with HOAc workup, the use of biaryl triflate **50** as the electrophile in place of **1a** did not produce the expected styrene product **51**, revealing the essential directing role of the isoquinoline moiety. Replacing the OTf group with a hydroxyl group completely suppressed the transformation, while bromide as a leaving group exhibited similar reactivity and enantioselectivity to OTf (90% yield and 96% ee). The ONf group, while delivering an excellent yield of **3**, a significant decrease in ee was obtained (Scheme 4B). Moreover, a time‐course analysis of the model reaction provided further kinetic insights (Scheme 4C). Product (*R*)‐**3** was obtained with a constant 99% ee throughout the reaction. And the enantiomeric excess of the recovered **1a** increased progressively (from 0% to 40% ee over 20 min), supporting an efficient kinetic resolution pathway. On the basis of these experimental results and previous reports [66, 67, 68, 69, 70, 71, 72, 73, 74, 75, 76, 77, 78, 79, 80, 81, 82, 83, 84, 85, 86, 87, 88, 89, 90, 91, 92, 93, 94, 95, 96, 97, 98, 99], a plausible catalytic cycle is proposed (Scheme 4D). Initially, the coordination of the isoquinoline moiety to the in situ formed complex Pd^0^/L* (**I**) enables C─O bond oxidative addition in **1** to give palladacycle **II** and **II′**. Following facile epimerization, arylpalladium species **II** undergoes a *cis*‐carbopalladation with alkynylborate **2** to form intermediate **III**, wherein palladium resides in the α‐position to the boron atom. Finally, intermediate **III** undergoes a 1,2‐migration affords the desired 1,2‐azaborepine products and regenerates the active palladium catalyst.

**SCHEME 5:**
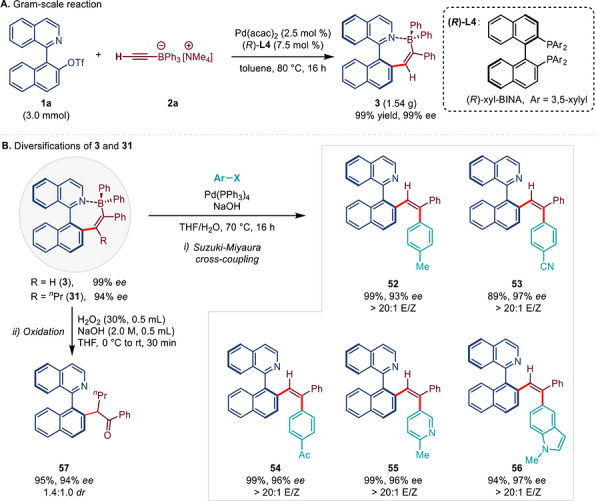
Scale‐up synthesis and derivatizations.

The synthetic potential of this methodology was further verified through a gram‐scale reaction of **1a** with **2a**, which produced **3** (1.54 g) in excellent yield (99%) with exceptional enantioselectivity (99%) (Scheme 5A). Moreover, the boron group in products also served as a versatile handle for diverse transformations (Scheme 5B). Notably, Pd‐catalyzed Suzuki–Miyaura cross‐coupling of compound **3** with various aryl halides proceeded smoothly [103, 104], furnishing chiral styrene derivatives **52–56** in high yields with excellent retention of enantiopurity and high *E*/*Z* selectivity. Treatment of **31** with H_2_O_2_/NaOH yielded ketone **57** in 95% yield with full retention of enantiopurity.

## 3 Conclusions

In summary, we have described a palladium‐catalyzed atroposelective cyclization of *N*‐heterobiaryl triflates with alkynyl boronates, which provides a variety of seven‐membered 1,2‐BN‐bridged biaryls in high yields and with excellent enantioselectivity. The transformation proceeds via a cascade of Pd‐catalyzed DyKAT, *cis*‐carbopalladation, and 1,2‐migration of alkynyl boronates. The reaction proceeds under mild conditions and demonstrates a broad substrate scope, as well as good tolerance for various functional groups. Moreover, the synthetic utility of this methodology has been illustrated through gram‐scale reactions and diverse transformations of the BN‐embedded products. This work not only expands the toolbox for synthesizing axially chiral π‐conjugated boron compounds but also opens new avenues for the exploration of BN‐embedded functional materials.

## Conflicts of Interest

The authors declare no conflicts of interest.

## Supporting information




**Supporting File 1**: advs75755‐sup‐0001‐SuppMat.pdf.

**Supporting File 2**: advs75755‐sup‐0002‐cif.zip.

## Data Availability

The data that supports the findings of this study are available in the supplementary material of this article.
