# Supplementary material for: Atroposelective Access to π‐Conjugated 1,2‐Azaborepines Enabled by Palladium‐Catalyzed Cyclization of N‐Heterobiaryls with Alkynylboronates
Source: Adv Sci (Weinh). 2026 May 19;13(45):e75755. doi: 10.1002/advs.75755 (PMC13335858; doi:10.1002/advs.75755)
Supplement: Supplementary file 1 [file ADVS-13-e75755-s002.pdf]

SUPPORTING INFORMATION

Atroposelective Access to π-Conjugated 1,2-Azaborepines Enabled by Palladium-Catalyzed Cyclization of N-Heterobiaryls with Alkynylboronates

Fengya He,+[a] Zhen Wang,+[a] Haoyu Guo,[a] Shuguang Chen,*[a] Xu Zhang,[a] Yongjia Shang,*[a] and Hui Wang*[a]

[a]Key Laboratory of Functional Molecular Solids (Ministry of Education), Anhui Key Laboratory of Molecular Based Materials, College of Chemistry and Materials Science, Anhui Normal University, Wuhu, 241002 (China)

E-mail: shugchen@ahnu.edu.cn; shyj@ahnu.edu.cn; wanghui29085@ahnu.edu.cn

TABLE OF CONTENTS

1. MATERIALS AND GENERAL METHODS 2

1.1. Glassware, Solvents and Reagents 2

1.2. Chromatography and Instrumentations 2

1.3 Photophysical Studies 3

2. EXPERIMENTAL DATA 4

2.1. Screening of the Reaction Conditions for Terminal Alkynylboronate **2a** 4

2.2. Screening of the Reaction Conditions for Internal Alkynylboronate **2b** 7

2.3. Synthesis of Starting Materials 9

2.3.1. Synthesis of Heterobiaryl Triflates **1b−1j** 9

2.3.2 Synthesis of Alkynylboronates **2** 10

2.4 Synthesis of Products **3–49** 12

2.5 Characterization Data 14

3. PHOTOPHYSICAL PROPERTY STUDIES 115

4. MECHANISTIC EXPERIMENTS 118

4.1 Control Experiments 118

4.2 Kinetic Experiments 120

4.3 Temperature Studies for the Racemization 121

5 SYNTHETIC APPLICATIONS 125

5.1 Gram-Scale Reaction 125

5.2 Transformations of Chiral Axial Seven-Membered Borates 126

5.2.1 Suzuki-Miyaura Cross-Coupling of Compound **3** 126

5.2.2 Direct Oxidation of Compound **31** 135

6. X-RAY CRYSTALLOGRAPHIC DATA 138

7. COMPUTATIONAL DETAILS 141

8. NMR SPECTRA OF ALL COMPOUNDS 152

9. SUPPLEMENTARY REFERENCES 246

1. MATERIALS AND GENERAL METHODS

1.1. Glassware, Solvents and Reagents

All manipulations were performed with oven-dried (120 °C for a minimum of 12 h) glassware under air or an atmosphere of nitrogen, unless otherwise stated.

All anhydrous solvents were commercially supplied. Reagents were purchased from commercial sources and used as received.

1.2. Chromatography and Instrumentations

Thin layer chromatography (TLC) was performed using Sillicorey 60 Å F254 fluorescent treated silica, which was visualised under UV light, or by staining with aqueous basic potassium permanganate followed by heating, or Hanessian's stain (CAM stain) followed by heating, as stated.

Flash column chromatography (FCC) was carried out using Sili Corey silica gel (200-300 mesh).

NMR spectra were recorded, using Bruker 400 MHz and 500 MHz for ^{1}H, ^{11}B, ^{13}C and ^{19}F acquisitions. All NMR spectra were recorder at 25 °C unless otherwise stated. Chemical shifts (δ) are reported in parts per million (ppm) and referenced to $CDCl_3$ (^{1}H: 7.26 ppm; ^{13}C: 77.16 ppm) or d_6-DMSO (^{1}H: 2.50 ppm; ^{13}C: 39.5 ppm). Coupling constants (J) are given in Hertz (Hz) and refer to apparent multiplicities (s = singlet, d = doublet, t = triplet, q = quartet, quin = quintet, hex = hextet, h = heptet, m = multiplet, brs = broad signal, dd = doublet of doublets, etc.). The ^{1}H NMR spectra are reported as follows: chemical shift (multiplicity, coupling constants, number of protons).

HPLC analysis were performed on Agilent 1260 Infinity II Prime system or Shimadzu LC-20A liquid chromatograph and with Daicel CHIRALPAK® columns.

IR spectra were recorded were recorded on Bruker INVENIO. Selected absorption maxima (ν_{max}) are reported in wavenumbers (cm^{-1}).

High resolution mass spectra (HRMS) were recorded on a Bruker Daltonics MicroTOF II by Electrospray Ionisation (ESI).

Melting point (M. p.): Stuart melting point apparatus X-4, Ruihongcheng Scientific, values are

uncorrected.

1.3 Photophysical Studies

UV-visible absorption and fluorescence emission spectra were recorded on a commercial spectrophotometer (Shimadzu UV-2450 and Edinburgh FS5 spectrometers, 190–900 nm scan range). **Absolute fluorescence quantum yields** were determined using a Hamamatsu Quantaurus spectrofluorometer equipped with an integrating sphere.

Circular dichroism (CD) spectra were measured on a BioLogic MOS-500 CD Spectrometer.

Circularly polarized luminescence (CPL) measurements were performed on an OLIS CPL SOLO spectrometer.

2. EXPERIMENTAL DATA

2.1. Screening of the Reaction Conditions for Terminal Alkynylboronate 2a

Table S1: Screening of Reaction Temperatures[a]

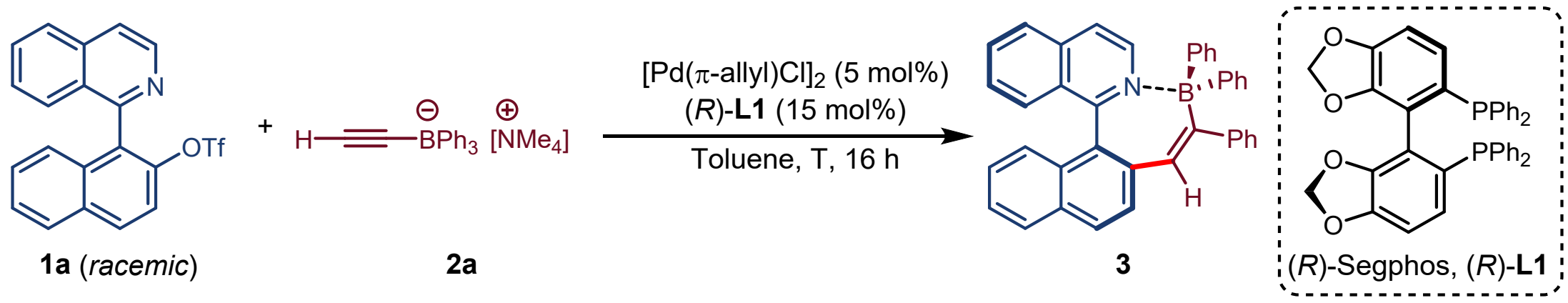

Entry	T	Yield (%)[b]	*Ee* (%)[c]
1	25 °C	18	82
2	40 °C	91	89
3	60 °C	92	90
4	80 °C	92	93
5	100 °C	80	93

[a] Reaction conditions: **1a** (0.2 mmol, 1.0 equiv.), **2a** (0.3 mmol, 1.5 equiv.), $[Pd(\pi\text{-allyl})Cl]_2$ (5 mol%), and (*R*)-**L1** (15 mol%) in anhydrous toluene (2.0 mL) for 16 hours under N_2. [b] The yield was determined by ^1H NMR analysis by using 1,3,5-trimethoxybenzene as an internal standard. [c] The *ee* values were determined by chiral HPLC analysis. *Ee* = Enantiomeric excess.

Table S2: Screening of Different Ligands[a]

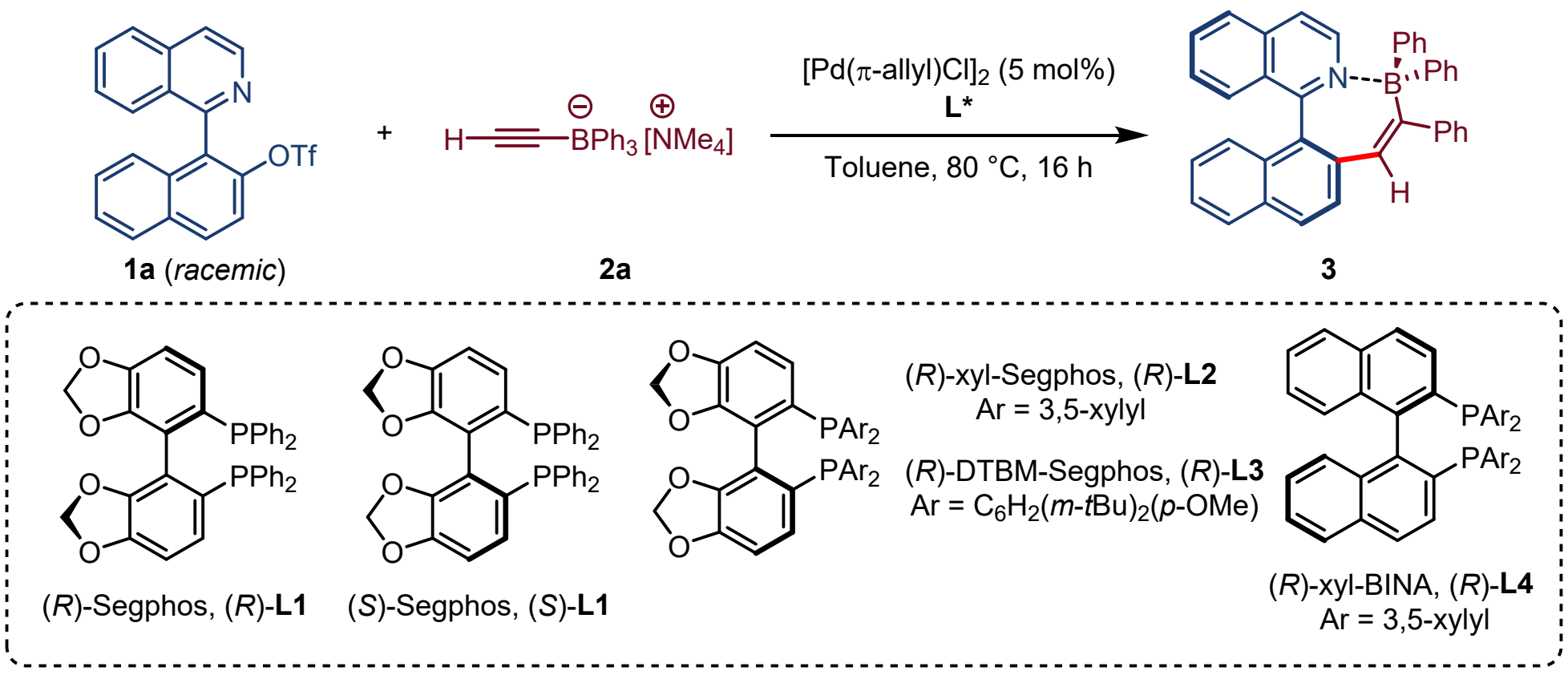

Entry	L*	Yield (%)[b]	*Ee* (%)[c]
1	(*S*)-**L1** (15 mol%)	83	-90
2	(*R*)-**L2** (15 mol%)	94	94
3	(*R*)-**L3** (15 mol%)	trace	--
4	(*R*)-**L4** (15 mol%)	94	98
5[d]	(*R*)-**L4** (7.5 mol%)	93	98

[a] Reaction conditions: **1a** (0.2 mmol, 1.0 equiv.), **2a** (0.3 mmol, 1.5 equiv.), [Pd(π-allyl)Cl]$_2$ (5 mol%), and **L*** (15 mol%) in anhydrous toluene (2.0 mL) at 80 °C for 16 hours under N_2. [b] The yield was determined by ^{1}H NMR analysis by using 1,3,5-trimethoxybenzene as an internal standard. [c] The *ee* values were determined by chiral HPLC analysis. [d][Pd(π-allyl)Cl]$_2$ (2.5 mol%). *Ee* = Enantiomeric excess.

Table S3: Screening of Different Pd-Catalysts[a]

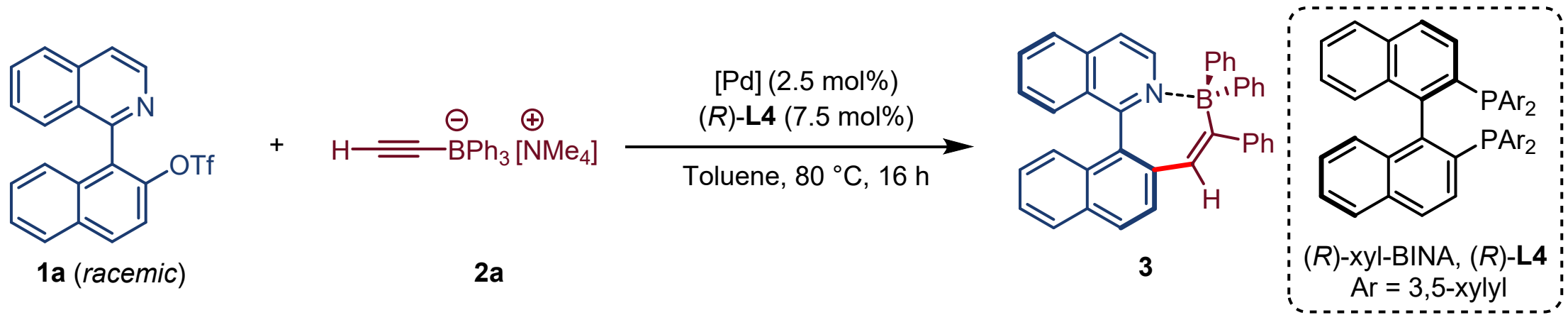

Entry	[Pd]	Yield (%)[b]	*Ee* (%)[c]
1	$Pd_2(dba)_3$ (2.5 mol%)	85	95
2	$Pd(dba)_2$ (2.5 mol%)	80	99
3	$Pd(acac)_2$ (2.5 mol%)	99(96)[d]	99
4	$Pd(OAc)_2$ (2.5 mol%)	86	98

[a] Reaction conditions: **1a** (0.2 mmol, 1.0 equiv.), **2a** (0.3 mmol, 1.5 equiv.), [Pd] (2.5 mol%), and (*R*)-**L4** (7.5 mol%) in anhydrous toluene (2.0 mL) 80 °C for 16 hours under nitrogen atmosphere. [b] The yield was determined by ^{1}H NMR analysis by using 1,3,5-trimethoxybenzene as an internal standard. [c] The *ee* values were determined by chiral HPLC analysis. [d]Isolated yield. *Ee* = Enantiomeric excess.

Table S4: Screening of Various Solvents[a]

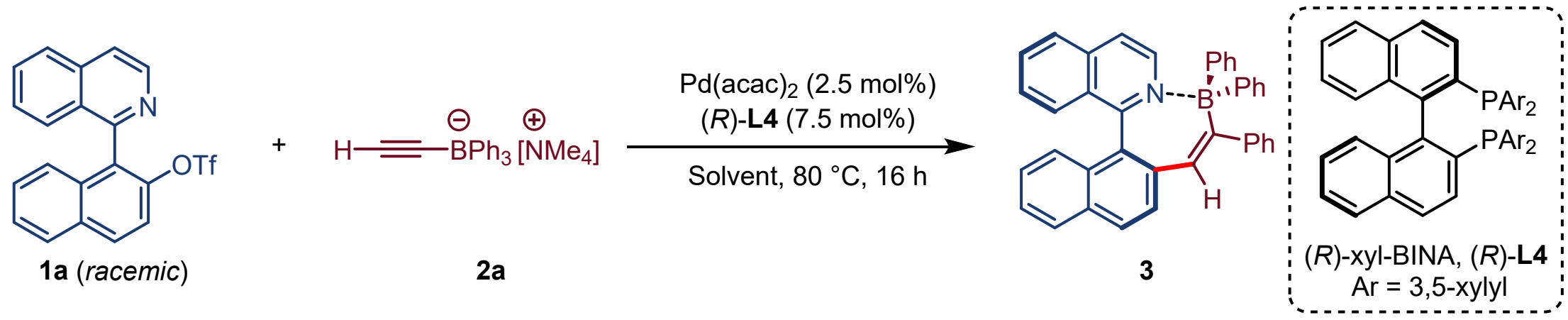

Entry	Solvent	Yield (%)[b]	*Ee* (%)[c]
1	THF	65	98
2	MeCN	30	97
3	DMF	46	97

[a] Reaction conditions: **1a** (0.2 mmol, 1.0 equiv.), **2a** (0.3 mmol, 1.5 equiv.), $Pd(acac)_2$ (2.5 mol%), and (*R*)-**L4** (7.5 mol%) in anhydrous solvent (2.0 mL) at 80 °C for 16 hours under N_2. [b] The yield was determined by ^{1}H NMR analysis by using 1,3,5-trimethoxybenzene as an internal standard. [c] The *ee* values were determined by chiral HPLC analysis. *Ee* = Enantiomeric excess.

Table S5: Control Experiments [a]

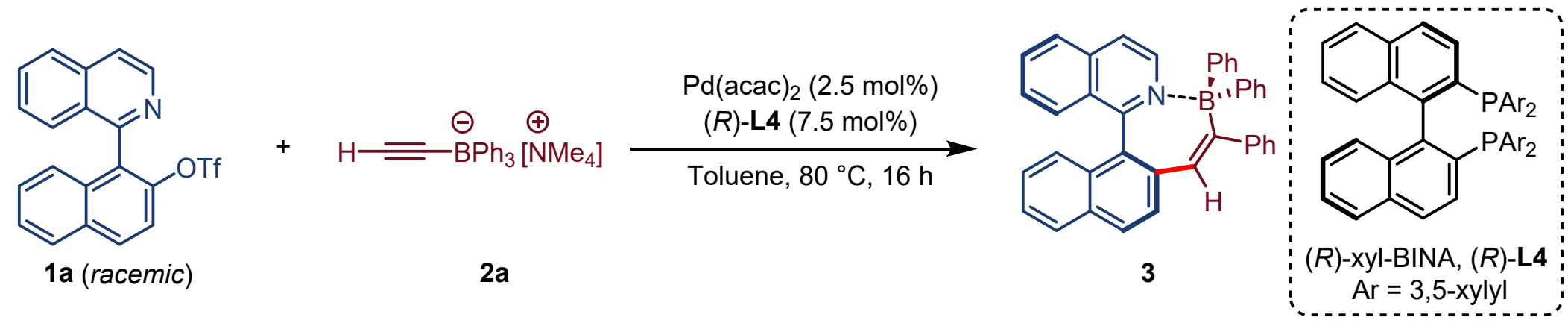

Entry	[Pd]	(*R*)-**L4**	Yield (%)[b]	*Ee* (%)[c]
1	--	(*R*)-**L4** (7.5 mol%)	N.D.	--
2	Pd(acac)$_2$ (2.5 mol%)	-	43	0

[a] Reaction conditions: **1a** (0.2 mmol, 1.0 equiv.), **2a** (0.3 mmol, 1.5 equiv.), Pd(acac)$_2$ (2.5 mol%), and (*R*)-**L4** (7.5 mol%) in anhydrous toluene (2.0 mL) at 80 °C for 16 hours under nitrogen atmosphere. [b] The yield was determined by ^{1}H NMR analysis by using 1,3,5-trimethoxybenzene as an internal standard. [c] The *ee* values were determined by chiral HPLC analysis. *Ee* = Enantiomeric excess.

2.2. Screening of the Reaction Conditions for Internal Alkynylboronate 2b

Table S6: Screening of the Pd-catalysts [a]

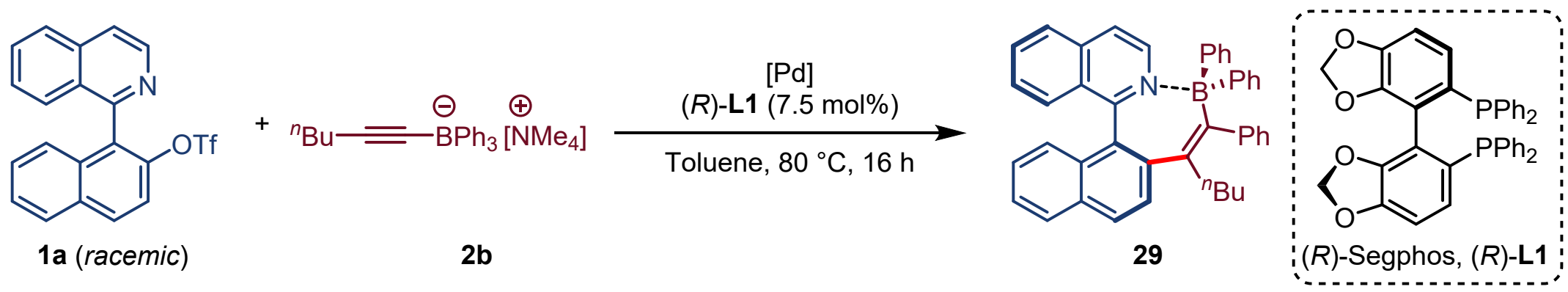

Entry	[Pd]	Yield (%)[b]	*Ee* (%)[c]
1[d]	Pd(acac)$_2$ (2.5 mol%)	N.D.	--
2	Pd$_2$(dba)$_3$ (2.5 mol%)	52	94
3[e]	Pd$_2$(dba)$_3$ (5.0 mol%)	65(61)[f]	94
4[e]	PdCl$_2$(PPh$_3$)$_2$ (5.0 mol%)	44	94
5[e]	PdCl$_2$(dppf) (5.0 mol%)	26	92
6[e]	Pd(PPh$_3$)$_4$ (5.0 mol%)	45	90

[a] Reaction conditions: **1a** (0.2 mmol, 1.0 equiv.), **2b** (0.3 mmol, 1.5 equiv.), [Pd] (2.5 mol%), and (*R*)-**L1** (7.5 mol%) in anhydrous toluene (2.0 mL) at 80 °C for 16 hours under N$_2$. [b] The yield was determined by ^{1}H NMR analysis with 1,3,5-trimethoxybenzene as an internal standard. [c] The *ee* values were determined by chiral HPLC analysis. *Ee* = Enantiomeric excess. [d] (*R*)-**L4** (15 mol%). [e] (*R*)-**L1** (15 mol%). [f] Isolated yield.

Table S7: Screening of Reaction Temperatures [a]

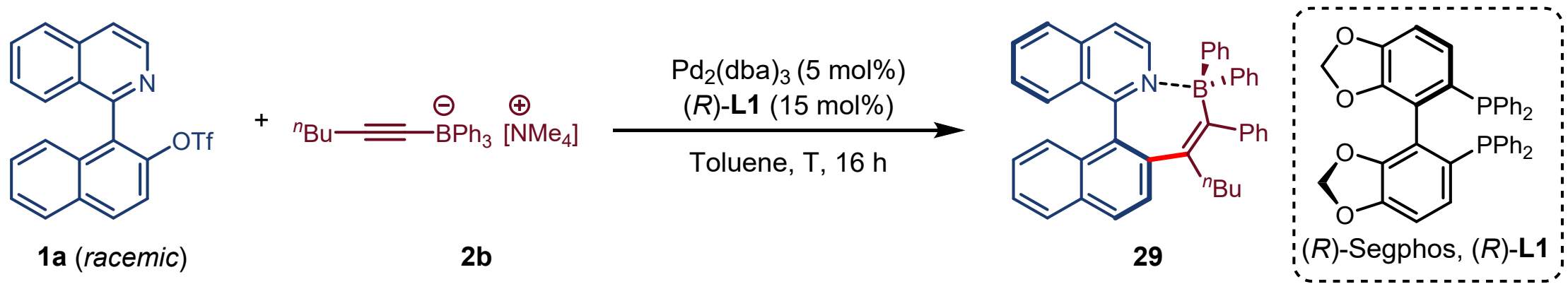

Entry	Temp.	Yield (%)[b]	*Ee* (%)[c]
1	60 °C	15	96
2	80 °C	65 (61)[d]	94
3	100 °C	47	89

[a] Reaction conditions: **1a** (0.2 mmol, 1.0 equiv.), **2b** (0.3 mmol, 1.5 equiv.), $Pd_2(dba)_3$ (5 mol%), and (*R*)-**L1** (15 mol%) in anhydrous toluene (2.0 mL) for 16 hours under N_2. [b] The yield was determined by ^{1}H NMR analysis with 1,3,5-trimethoxybenzene as an internal standard. [c] The ee values were determined by chiral HPLC analysis. *Ee* = Enantiomeric excess. [d] Isolated yield.

Table S8: Screening Different Ligands[a]

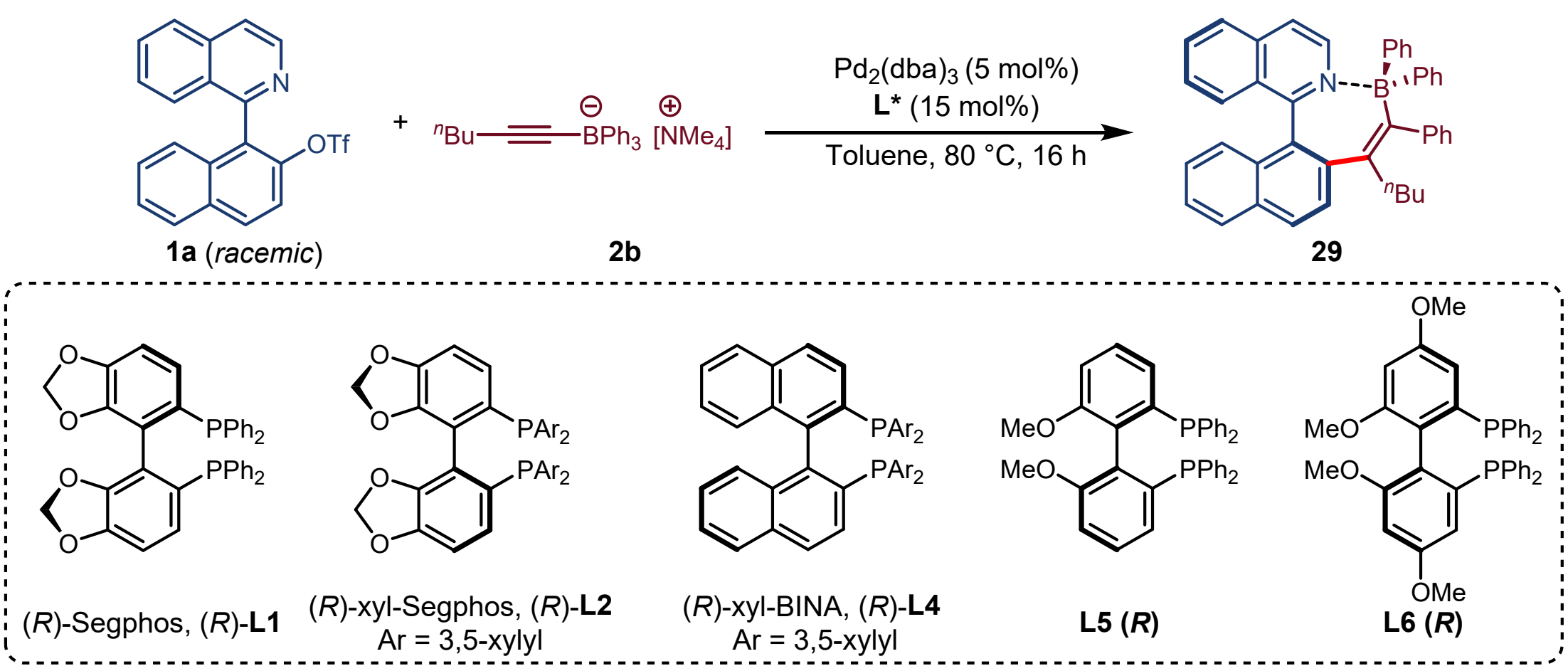

Entry	L*	Yield (%)[b]	*Ee* (%)[c]
1	(*R*)-**L2**	28	90
2	(*R*)-**L4**	24	67
3	(*R*)-**L5**	30	87
4	(*R*)-**L6**	40	78

[a] Reaction conditions: **1a** (0.2 mmol, 1.0 equiv.), **2a** (0.3 mmol, 1.5 equiv.), $Pd_2(dba)_3$ (5 mol%), and **L*** (15 mol%) in anhydrous toluene (2.0 mL) at 80°C for 16 hours under N_2. [b] The yield was determined by ^{1}H NMR analysis with 1,3,5-trimethoxybenzene as an internal standard. [c] The ee values were determined by chiral HPLC analysis. *Ee* = Enantiomeric excess.

2.3. Synthesis of Starting Materials

2.3.1. Synthesis of Heterobiaryl Triflates 1b–1j[1-6]

General procedure A: for Heterobiaryl Triflates Synthesis from Phenols

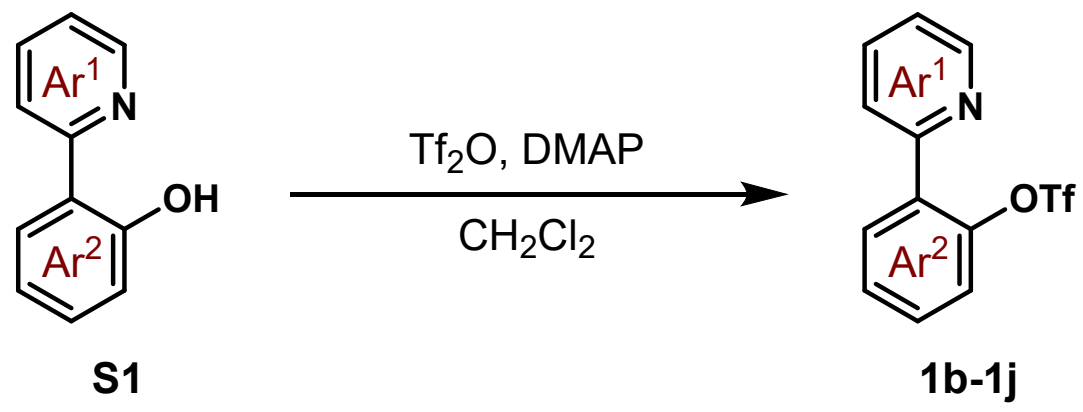

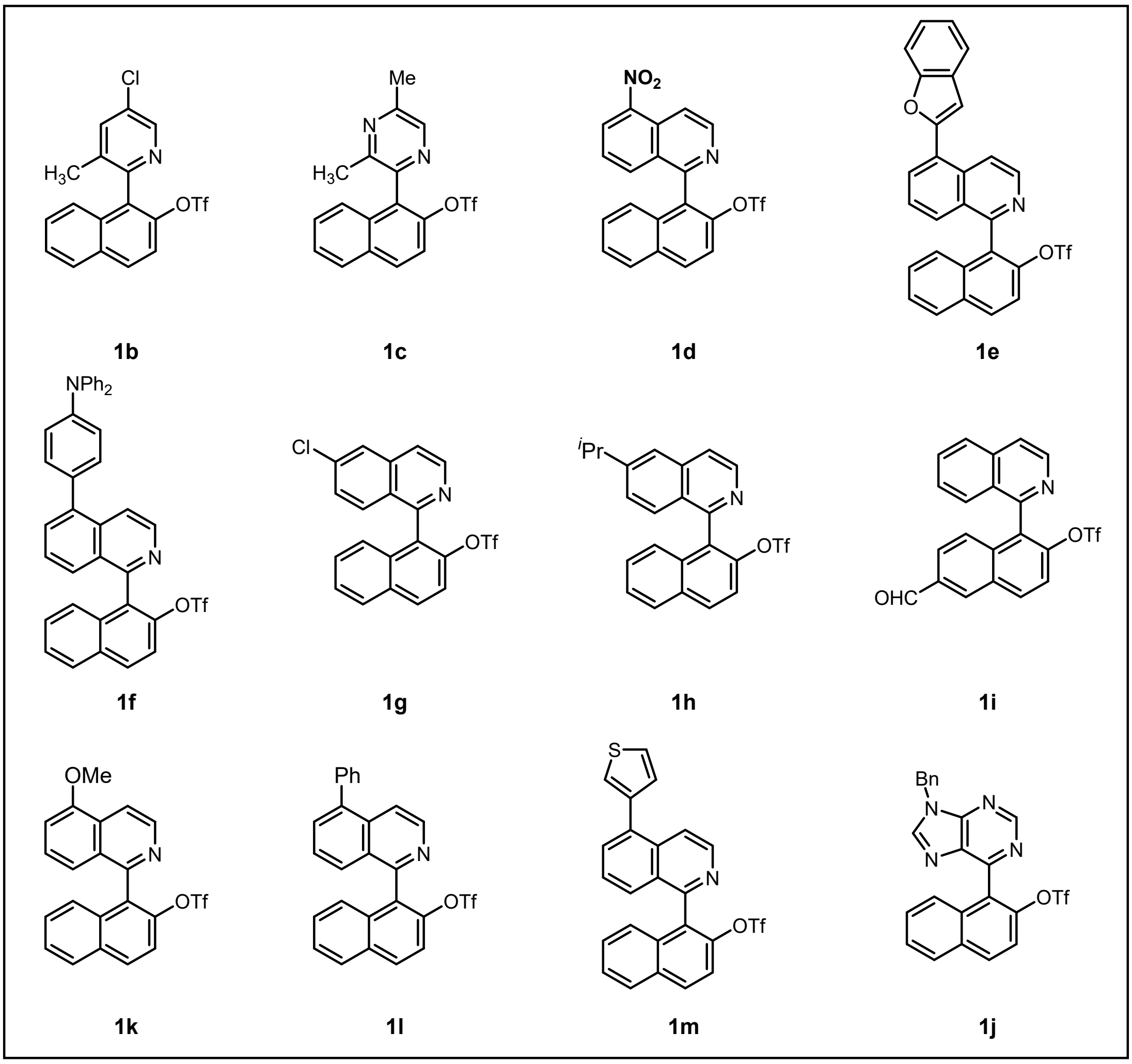

Following a described procedure[1-6], to a solution of the mixture of corresponding **S1** (5.0 mmol, 1.0 equiv.) and DMAP (10.0 mmol, 2.0 equiv.) in dry CH_2Cl_2 (10.0 mL) was added Tf_2O (6.0 mmol, 1.2 equiv.) dropwise at 0 °C (ice bath) within 5 min. The reaction mixture was stirred at 25 °C until corresponding **S1** was fully consumed as monitored by TLC analysis. The reaction mixture was diluted with CH_2Cl_2 (10.0 mL) and $NaHCO_3$ aq. (15.0 mL). The aqueous phase was extracted with CH_2Cl_2 (3 ×

10.0 mL). The combined organic phases were washed with brine, dried over $MgSO_4$ and then filtered. Filtrate was concentrated under reduced pressure. The resulted crude material was purified by flash column chromatography (*n*-hexane/EtOAc) to afford the desired product **1b-1j**.

The followed *N*-heterobiaryl triflates **1a**, **1n-1y** was synthesized according to the literature:[2-4],[6]

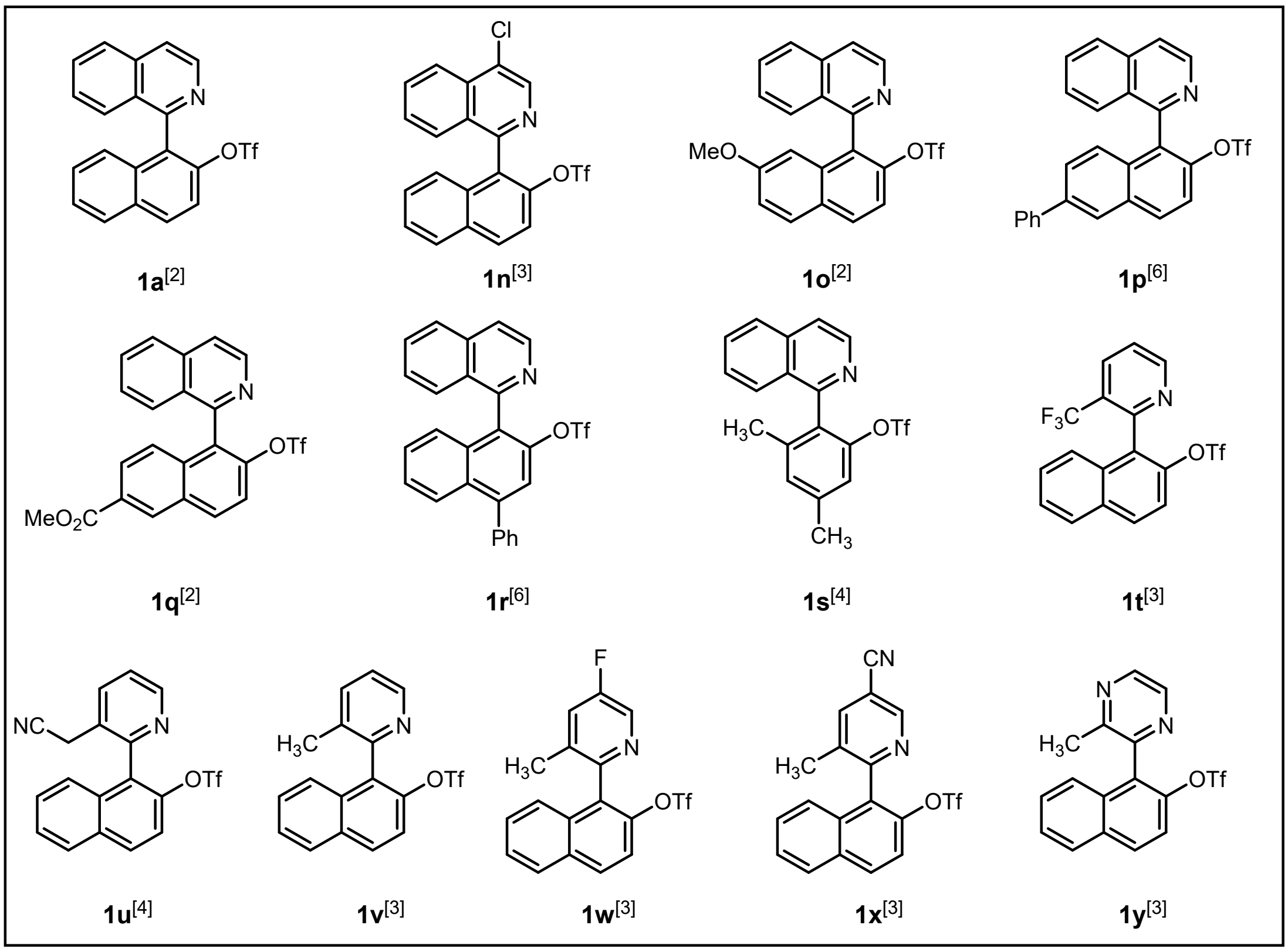

2.3.2 Synthesis of Alkynylboronates 2

General procedure B: Lithiation-Borylation[7-8]

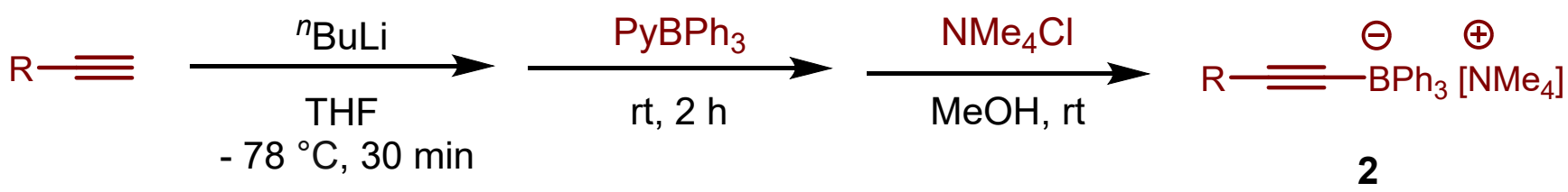

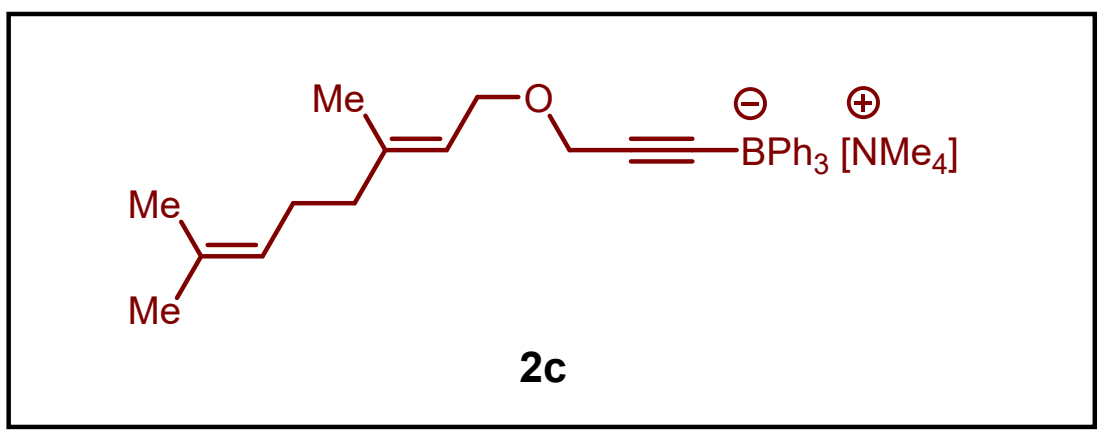

To a stirred solution of alkyne (10.0 mmol, 1.0 equiv.) in anhydrous THF (20 mL) at −78 °C (dry ice/acetone) was added *n*-BuLi (1.6 M in *n*-hexane, 11.0 mmol, 1.1 equiv.). After 30 min at the same

temperature, Ph₃B·Py (11.0 mmol, 1.1 equiv.) was added, and the cooling bath was removed. The mixture was stirred for 2 hours at room temperature before quenching with MeOH (5 mL). Volatile materials were removed under reduced pressure and the residue was dissolved in MeOH (20 mL). Me_4NCl (20 mmol, 2.0 equiv.) was added with stirring, resulting a white solid, which was collected by filtration and washed with cold MeOH (20 mL×3) to afford the alkynylboronates **2**.

The followed alkynylboronates **2a**-**2b**, **2d**-**2w** was synthesized according to the literature:[7-10]

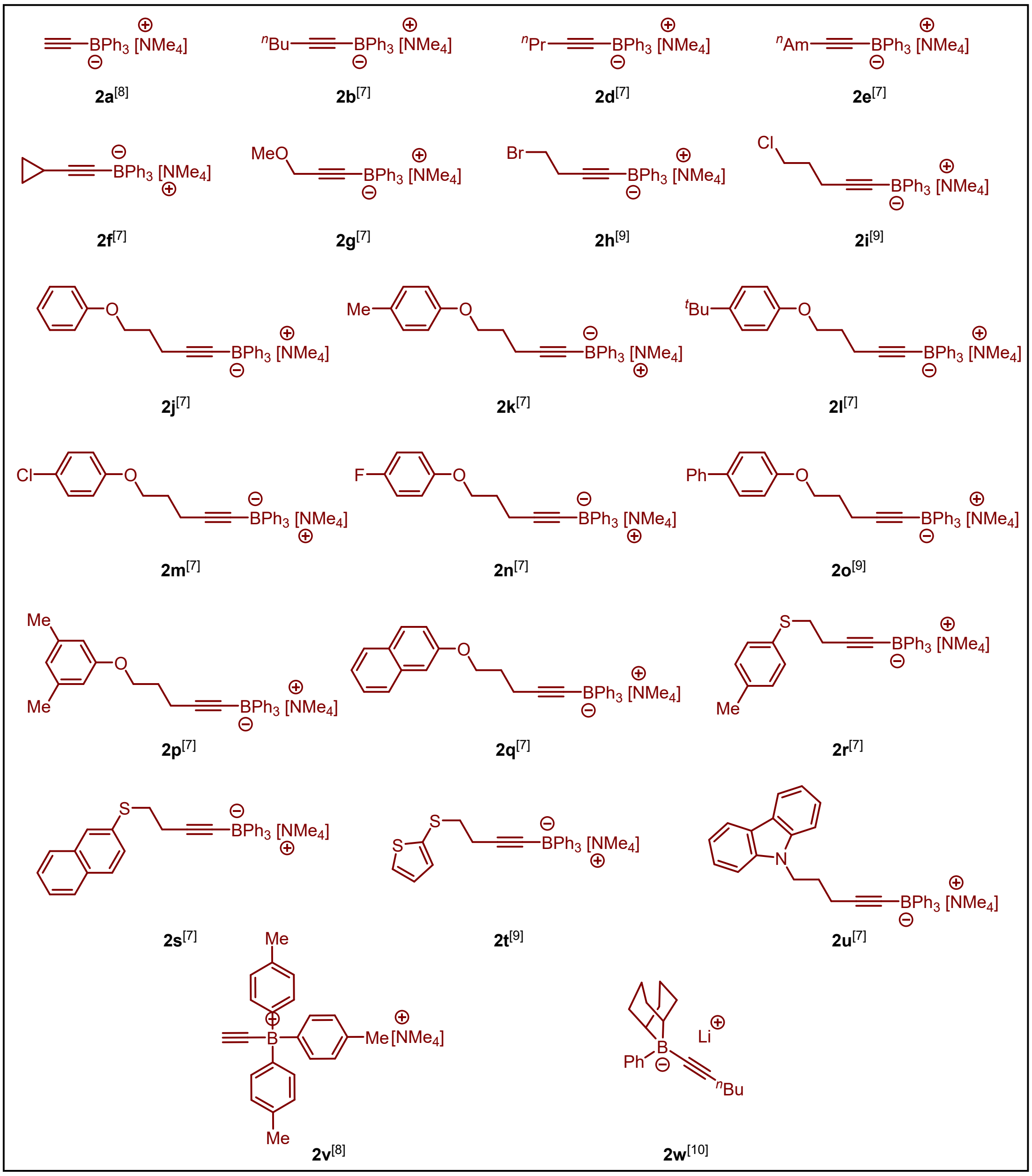

2.4 Synthesis of Products 3−49

General procedure C: Pd-catalyzed Reaction of N-heterobiaryl Triflates with Terminal Alkynylboronates for the product 3−28

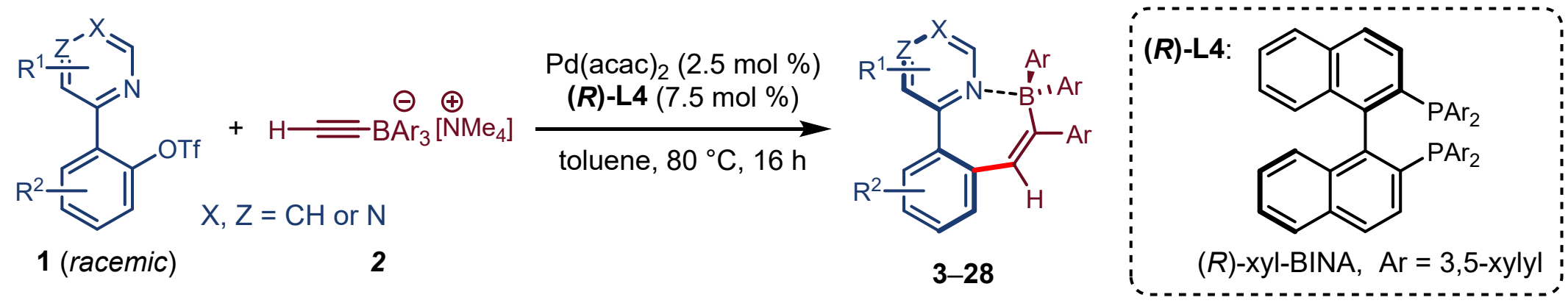

In glovebox, to an oven-dried 8.0 mL vial equipped with a magnetic stir bar was added Pd(acac)$_2$ (2.5 mol%, 1.5 mg) and (*R*)-**L4** (0.015 mmol, 7.5 mol%, 11.0 mg) under N_2 atmosphere at room temperature. The anhydrous toluene (2.0 mL) was added to the vial and the mixture was stirred for 30 min at the same temperature to afford the stock solution. Alkyne *tetra*-coordinate borate **2** (0.3 mmol, 1.5 equiv.) and heterobiaryl triflate **1** (0.2 mmol, 1.0 equiv.) were added into a 10 mL Schlenk tube and the stock solution was then transferred to the Schlenk tube. Finally, the Schlenk tube was sealed with a septum, removed from the glovebox, and then was heated to 80 °C at an oil bath and stirred for 16 h at the same temperature. After that, the reaction was cooled to room temperature and the crude reaction mixture was quenched by two drops of water and then filtered through a pad of silica gel. The resulted filtrate was concentrated under reduced pressure. The crude material was finally purified by flash column chromatography (*n*-hexane/EtOAc) to afford the desired product **3−28**.

General procedure D: Pd-catalyzed Reaction of N-heterobiaryl Triflates with Internal Alkynylboronates for the product 29−48

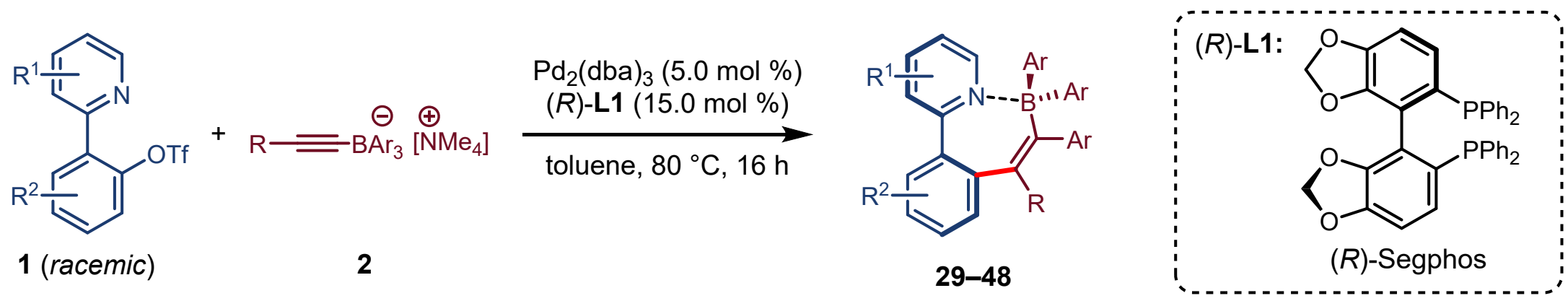

In glovebox, to an oven-dried 8.0 mL vial equipped with a magnetic stir bar was added Pd$_2$(dba)$_3$ (0.01 mmol, 5.0 mol%, 9.2 mg) and (*R*)-**L1** (0.03 mmol, 15.0 mol%, 15.3 mg) under N_2 atmosphere at room temperature. The anhydrous toluene (2.0 mL) was added to the vial and the mixture was stirred for 30 min at ambient temperature to afford stock solution. Alkyne tetracoordinate borate **2** (0.3 mmol, 1.5

equiv.) and **1** (0.2 mmol, 1.0 equiv.) were added into a 10 mL Schlenk tube and the stock solution was then transferred to the Schlenk tube. Finally, the Schlenk tube was sealed with a septum, removed from the glovebox, and then was heated to 80 °C at an oil bath and stirred for 16 h at the same temperature. The resulted crude reaction mixture was filtered through a pad of silica gel and filtrate was concentrated under reduced pressure. The crude material was purified by flash column chromatography (*n*-hexane/EtOAc) to afford the desired product **29–48**.

General procedure E: Pd-catalyzed Reaction of N-heterobiaryl Triflates with Internal Alkynylboronates for the product 49

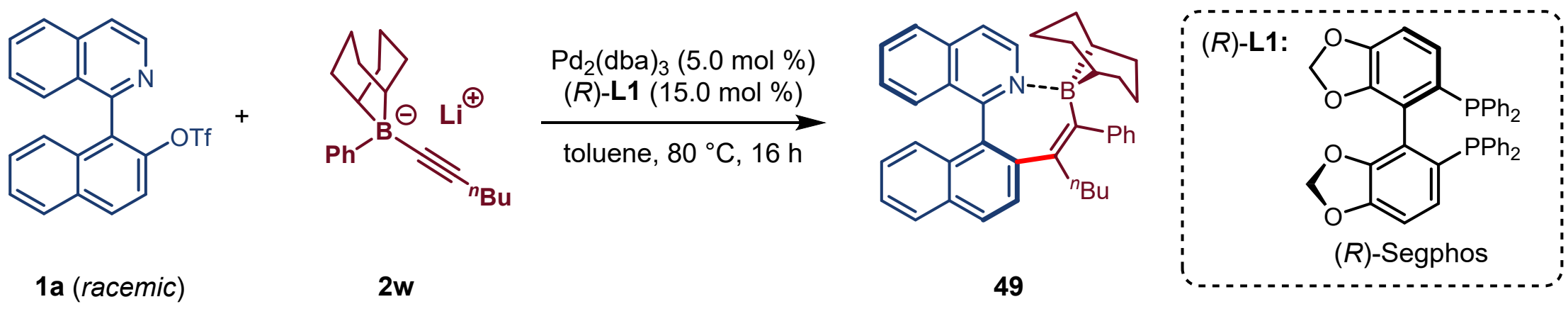

In glovebox, to an oven-dried 8.0 mL vial equipped with a magnetic stir bar was added $Pd_2(dba)_3$ (0.01 mmol, 5.0 mol%, 9.2 mg) and (*R*)-**L1** (0.03 mmol, 15.0 mol%, 15.3 mg) under N_2 atmosphere at room temperature. The anhydrous toluene (2.0 mL) was added to the vial and the mixture was stirred for 30 min at ambient temperature to afford stock solution. Alkyne tetracoordinate borate **2w** (0.3 mmol, 1.5 equiv., 85.8 mg) and **1a** (0.2 mmol, 1.0 equiv., 80.6 mg) were added into a 10 mL Schlenk tube and the stock solution was then transferred to the Schlenk tube. Finally, the Schlenk tube was sealed with a septum, removed from the glovebox, and then was heated to 80 °C at an oil bath and stirred for 16 h at the same temperature. The resulted crude reaction mixture was filtered through a pad of silica gel and filtrate was concentrated under reduced pressure. The crude material was purified by flash column chromatography (*n*-hexane/EtOAc) to afford the desired product **49**.

2.5 Characterization Data

1-(5-Chloro-3-methylpyridin-2-yl)naphthalen-2-yl trifluoromethanesulfonate (1b)

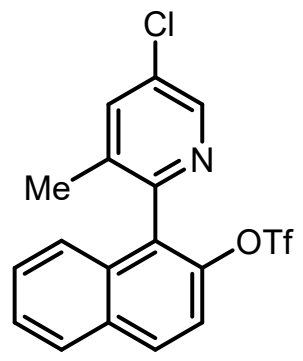

1b

Prepared following **Procedure A**, using 1-(5-chloro-3-methylpyridin-2-yl)naphthalen-2-ol (2.69 g, 10.0 mmol, 1.0 equiv.), DMAP (0.12 g, 1.0 mmol, 10 mol%), Pyridine (0.97 mL, 12.0 mmol, 1.2 equiv.), and Tf_2O (2.02 mL, 12.0 mmol, 1.2 equiv.). Purification by flash column chromatography (Petroleum ether/EtOAc: 10/1) to afford the title compound (3.81 g, 95%) as a colorless oil.

TLC: R_f = 0.5 (Petroleum ether/EtOAc: 10/1, $KMnO_4$ stain).

NMR Spectroscopy (*see spectra*):

^{1}**H NMR** (500 MHz, $CDCl_3$) δ_H = 8.64 (d, J = 2.3 Hz, 1H), 8.01 (d, J = 9.2 Hz, 1H), 7.95 (d, J = 8.4 Hz, 1H), 7.74 (d, J = 2.3 Hz, 1H), 7.57 (dt, J = 1.5, 6.9 Hz, 1H), 7.51 (d, J = 9.0 Hz, 1H), 7.50 (dt, J = 2.6, 6.9 Hz, 1H), 7.35 (d, J = 9.6 Hz, 1H), 2.08 (s, 3H) ppm;

^{13}**C NMR** (125 MHz, $CDCl_3$) δ_C = 150.3, 146.4, 144.4, 137.8, 135.5, 132.6, 132.2, 132.0, 131.3, 129.5, 128.5, 128.2, 127.3, 125.7, 119.5, 118.4 (q, $^1J_{C\text{-}F}$ = 320.2 Hz), 18.5 ppm;

^{19}**F NMR** (376 MHz, $CDCl_3$) δ_F = -74.34 ppm.

IR (film): ν_{max} 3426, 1631, 1599, 1423, 1217, 1141, 947, 835, 677, 626, 591 cm^{-1}.

HRMS (ESI): m/z calculated for $C_{17}H_{12}ClF_3NO_3S^+$ [M+H]$^+$, 402.0173, found, 402.0170.

1-(3,5-Dimethylpyrazin-2-yl)naphthalen-2-yl trifluoromethanesulfonate (1c)

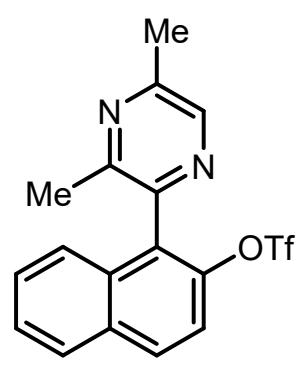

1c

Prepared following **Procedure A**, using 1-(3,5-dimethylpyrazin-2-yl)naphthalen-2-ol (2.50 g, 10.0

mmol, 1.0 equiv.), DMAP (122.1 mg, 1.0 mmol, 10 mol%), Pyridine (0.97 mL, 12.0 mmol, 1.2 equiv.), and Tf_2O (2.02 mL, 12.0 mmol, 1.2 equiv.). Purification by flash column chromatography (Petroleum ether/EtOAc: 10/1) to afford the title compound (3.51 g, 92%) as a colorless oil.

TLC: R_f = 0.4 (Petroleum ether/EtOAc: 10/1, $KMnO_4$ stain).

NMR Spectroscopy (*see spectra*):

^{1}H NMR (400 MHz, $CDCl_3$) δ_H = 8.52 (s, 1H), 8.02 (d, J = 9.1 Hz, 1H), 7.94 (d, J = 8.3 Hz, 1H), 7.56 (td, J = 1.4, 7.5 Hz, 1H), 7.53 – 7.47 (m, 2H), 7.35 (d, J = 8.6 Hz, 1H), 2.66 (s, 3H), 2.30 (s, 3H) ppm;

^{13}C NMR (125 MHz, $CDCl_3$) δ_C = 153.3, 153.0, 144.7, 144.5, 141.8, 132.7, 132.3, 131.5, 128.7, 128.6, 128.2, 127.3, 125.6, 119.5, 118.4 (q, $^1J_{C-F}$ = 320.2 Hz), 21.7, 21.6 ppm;

^{19}F NMR (376 MHz, $CDCl_3$) δ_F = -74.34 ppm.

IR (film): ν_{max} 1630, 1597, 1423, 1354, 1216, 1141, 946, 835, 627 cm^{-1}.

HRMS (ESI): m/z calculated for $C_{17}H_{14}F_3N_2O_3S^+$ $[M+H]^+$, 383.0672, found, 383.0667.

1-(5-Nitroisoquinolin-1-yl)naphthalen-2-yl trifluoromethanesulfonate (1d)

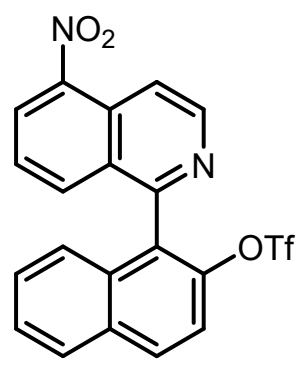

1d

Prepared following **Procedure A**, using 1-(5-nitroisoquinolin-1-yl)naphthalen-2-ol (3.16 g, 10.0 mmol, 1.0 equiv.), DMAP (122.1 mg, 1.0 mmol, 10 mol%), Pyridine (0.97 mL, 12.0 mmol, 1.2 equiv.), and Tf_2O (2.02 mL, 12.0 mmol, 1.2 equiv.). Purification by flash column chromatography (Petroleum ether/EtOAc: 10/1) to afford the title compound (4.03 g, 90%) as a colorless oil.

TLC: R_f = 0.5 (Petroleum ether/EtOAc: 10/1, $KMnO_4$ stain).

NMR Spectroscopy (*see spectra*):

^{1}H NMR (400 MHz, $CDCl_3$) δ_H = 8.99 (d, J = 6.3 Hz, 1H), 8.66 (d, J = 7.1 Hz, 1H), 8.54 (d, J =

7.6 Hz, 1H), 8.15 (d, J = 9.1 Hz, 1H), 8.02 (d, J = 8.3 Hz, 1H), 7.81 (d, J = 8.5 Hz, 1H), 7.65 – 7.56 (m, 2H), 7.54 (t, J = 9.7 Hz, 1H), 7.43 (t, J = 8.4 Hz, 1H), 7.18 (d, J = 8.5 Hz, 1H) ppm;

^{13}C NMR (100 MHz, $CDCl_3$) δ_C = 155.2, 146.0, 145.3, 145.0, 134.0, 133.0, 132.6, 132.1, 128.9, 128.7, 128.6, 128.6, 128.6, 128.4, 127.5, 126.3, 126.1, 119.6, 118.2 (q, $^1J_{C-F}$ = 320.1 Hz), 116.5 ppm;

^{19}F NMR (376 MHz, $CDCl_3$) δ_F = -74.37 ppm.

IR (film): ν_{max} 2868, 2769, 1701, 1623, 1525, 1310, 986, 945, 825, 810, 740 cm^{-1}.

HRMS (ESI): m/z calculated for $C_{20}H_{12}F_3N_2O_5S^+$ [M+H]$^+$, 449.0414, found, 449.0419.

1-[5-(Benzofuran-2-yl)isoquinolin-1-yl]naphthalen-2-yl trifluoromethanesulfonate (1e)

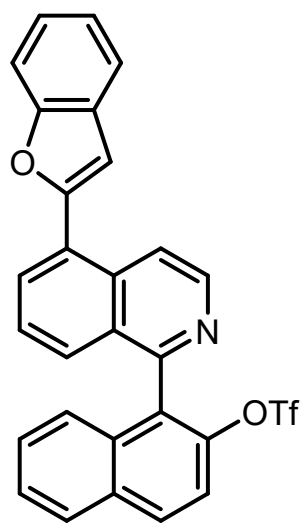

1e

Prepared following **Procedure A**, using 1-[5-(benzofuran-2-yl)isoquinolin-1-yl]naphthalen-2-ol (3.87 g, 10.0 mmol, 1.0 equiv.), DMAP (122.1 mg, 1.0 mmol, 10 mol%), Pyridine (0.97 mL, 12.0 mmol, 1.2 equiv.), and Tf_2O (2.02 mL, 12.0 mmol, 1.2 equiv.). Purification by flash column chromatography (Petroleum ether/EtOAc: 10/1) to afford the title compound (4.72 g, 91%) as a white solid.

TLC: R_f = 0.5 (Petroleum ether/EtOAc: 10/1, $KMnO_4$ stain).

M. p.: 84 – 85 °C.

NMR Spectroscopy (*see spectra*):

^{1}H NMR (400 MHz, $CDCl_3$) δ_H = 8.86 (d, J = 6.0 Hz, 1H), 8.52 (d, J = 6.0 Hz, 1H), 8.16 – 8.10 (m, 2H), 8.01 (d, J = 8.3 Hz, 1H), 7.73 (d, J = 6.8 Hz, 1H), 7.6 (d, J = 10.2 Hz, 1H), 7.62 (d, J = 11.4 Hz, 1H), 7.58 (dt, J = 1.8, 10.4 Hz, 1H), 7.55 – 7.50 (m, 2H), 7.45 – 7.38 (m, 2H), 7.35 (dt, J = 1.4, 9.3 Hz, 1H), 7.28 (d, J = 7.6 Hz, 1H), 7.22 (s, 1H) ppm;

^{13}**C NMR** (100 MHz, $CDCl_3$) δ_C = 155.3, 154.8, 154.2, 145.1, 143.7, 133.9, 133.3, 132.6, 131.5, 131.2, 129.6, 128.9, 128.9, 128.4, 128.1, 128.1, 127.3, 127.3, 126.6, 125.1, 123.4, 121.4, 119.6, 119.1, 118.3 (d, $^1J_{C-F}$ = 320.4 Hz), 111.6, 106.8 ppm;

^{19}**F NMR** (376 MHz, $CDCl_3$) δ_F = -74.38 ppm.

IR (film): ν_{max} 1703, 1555, 1510, 1426, 1308, 1249, 1212, 1136, 963, 943, 832, 754 cm^{-1}.

HRMS (ESI): m/z calculated for $C_{28}H_{17}F_3NO_4S^+$ [M+H]$^+$, 520.0825, found, 520.0824.

1-{5-[4-(Diphenylamino)phenyl]isoquinolin-1-yl}naphthalen-2-yl trifluoromethanesulfonate (1f)

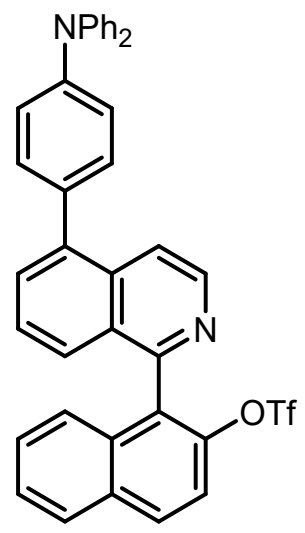

1f

Prepared following **Procedure A**, using 1-{5-[4-(diphenylamino)phenyl]isoquinolin-1-yl}naphthalen-2-ol (5.14 g, 10.0 mmol, 1.0 equiv.), DMAP (122.1 mg, 1.0 mmol, 10 mol%), Pyridine (0.97 mL, 12.0 mmol, 1.2 equiv.), and Tf_2O (2.02 mL, 12.0 mmol, 1.2 equiv.). Purification by flash column chromatography (Petroleum ether/EtOAc: 10/1) to afford the title compound (5.81 g, 90%) as a white solid.

TLC: R_f = 0.5 (Petroleum ether/EtOAc: 10/1, $KMnO_4$ stain).

M. p.: 93 – 94 °C.

NMR Spectroscopy (*see spectra*):

^{1}**H NMR** (400 MHz, $CDCl_3$) δ_H = 8.74 (d, J = 6.0 Hz, 1H), 8.11 (d, J = 9.1 Hz, 1H), 8.03 (d, J = 7.4 Hz, 1H), 8.00 (d, J = 9.7 Hz, 1H), 7.68 (dd, J = 1.5, 6.8 Hz, 1H), 7.62 (d, J = 9.0 Hz, 1H), 7.58 (t, J = 8.2 Hz, 1H), 7.48 – 7.40 (m, 5H), 7.33 (t, J = 7.9 Hz, 5H), 7.23 (dt, J = 1.3,9.1 Hz, 6H), 7.09 (t, J = 7.3 Hz, 2H) ppm;

^{13}**C NMR** (100 MHz, $CDCl_3$) δ_C = 154.3, 147.8, 147.7, 145.2, 142.8, 139.6, 134.9, 133.3, 132.7,

132.6, 131.4, 131.1, 131.0, 129.8, 129.5, 128.9, 128.4, 128.0, 127.3, 127.3, 126.7, 126.1, 124.9, 123.4, 123.2, 119.6, 118.3 (q, $^{1}J_{C\text{-}F}$ = 320.7 Hz) ppm;

^{19}F NMR (376 MHz, $CDCl_3$) δ_F = -74.42 ppm.

IR (film): ν_{max} 1736, 1509, 1489, 1422, 1315, 1275, 1214, 1140, 945, 818, 753, 697 cm^{-1}.

HRMS (ESI): m/z calculated for $C_{38}H_{26}F_3N_2O_3S^+$ [M+H]$^+$, 647.1611, found, 647.1603.

1-(6-Chloroisoquinolin-1-yl)naphthalen-2-yl trifluoromethanesulfonate (1g)

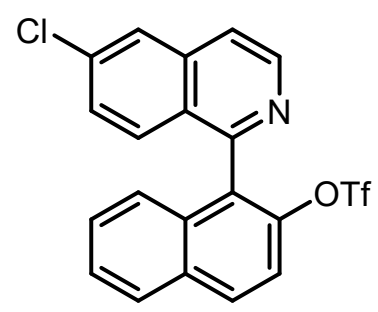

1g

Prepared following **Procedure A**, using 1-(6-chloroisoquinolin-1-yl)naphthalen-2-ol (3.05 g, 10.0 mmol, 1.0 equiv.), DMAP (122.1 mg, 1.0 mmol, 10 mol%), Pyridine (0.97 mL, 12.0 mmol, 1.2 equiv.), and Tf_2O (2.02 mL, 12.0 mmol, 1.2 equiv.). Purification by flash column chromatography (Petroleum ether/EtOAc: 10/1) to afford the title compound (3.93 g, 90%) as a white solid.

TLC: R_f = 0.5 (Petroleum ether/EtOAc: 10/1, $KMnO_4$ stain).

M. p.: 66 – 67 °C.

NMR Spectroscopy (*see spectra*):

^{1}H NMR (400 MHz, $CDCl_3$) δ_H = 8.78 (d, J = 5.8 Hz, 1H), 8.11 (d, J = 9.1 Hz, 1H), 8.00 (d, J = 8.3 Hz, 1H), 7.96 (s, 1H), 7.76 (d, J = 5.8 Hz, 1H), 7.63 – 7.54 (m, 2H), 7.4 – 7.40 (m, 3H), 7.23 (d, J = 8.5 Hz, 1H) ppm;

^{13}C NMR (100 MHz, $CDCl_3$) δ_C = 154.3, 145.0, 143.9, 137.2, 137.0, 133.1, 132.6, 131.6, 129.0, 129.0, 128.8, 128.5, 128.2, 127.4, 126.8, 126.4, 126.0, 120.5, 118.2 (q, $^{1}J_{C\text{-}F}$ = 320.4 Hz), 119.6 ppm;

^{19}F NMR (376 MHz, $CDCl_3$) δ_F = -74.43 ppm.

IR (film): ν_{max} 1613, 1557, 1427, 1345, 1212, 1136, 963, 943, 828, 624, 601 cm^{-1}.

HRMS (ESI): m/z calculated for $C_{20}H_{12}ClF_3NO_3S^+$ [M+H]$^+$, 438.0173, found, 438.0183.

1-(6-Isopropylisoquinolin-1-yl)naphthalen-2-yl trifluoromethanesulfonate (1h)

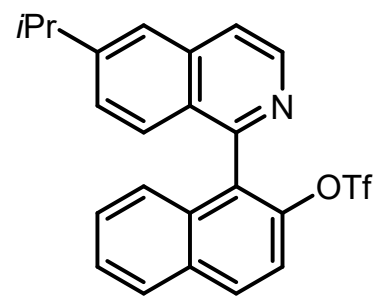

1h

Prepared following **Procedure A**, using 1-(6-isopropylisoquinolin-1-yl)naphthalen-2-ol (3.13 g, 10.0 mmol, 1.0 equiv.), DMAP (122.1 mg, 1.0 mmol, 10 mol%), Pyridine (0.97 mL, 12.0 mmol, 1.2 equiv.), and Tf_2O (2.02 mL, 12.0 mmol, 1.2 equiv.). Purification by flash column chromatography (Petroleum ether/EtOAc: 10/1) to afford the title compound (4.10 g, 92%) as a colorless oil.

TLC: R_f = 0.5 (Petroleum ether/EtOAc: 10/1, $KMnO_4$ stain).

NMR Spectroscopy (*see spectra*):

^{1}**H NMR** (400 MHz, $CDCl_3$) δ_H = 8.71 (d, J = 5.7 Hz, 1H), 8.09 (d, J = 9.0 Hz, 1H), 7.99 (d, J = 8.1 Hz, 1H), 7.78 (d, J = 5.8 Hz, 1H), 7.75 (s, 1H), 7.59 (d, J = 9.1 Hz, 1H), 7.55 (t, J = 8.3 Hz, 1H), 7.43 – 7.33 (m, 3H), 7.27 (d, J = 8.4 Hz, 1H), 3.10 (hept, J = 6.8 Hz, 1H), 1.35 (dd, J = 1.8, 6.9 Hz, 6H) ppm;

^{13}**C NMR** (125 MHz, $CDCl_3$) δ_C = 153.6, 151.6, 145.1, 142.7, 136.9, 133.3, 132.6, 131.2, 129.7, 128.3, 128.0, 127.9, 127.4, 127.2, 126.8, 126.7, 123.3, 121.2, 119.6, 118.3 (q, $^1J_{C\text{-}F}$ = 320.7 Hz), 34.5, 23.7, 23.6 ppm;

^{19}**F NMR** (376 MHz, $CDCl_3$) δ_F = -74.46 ppm.

IR (film): ν_{max} 3048, 2964, 1599, 1580, 1563, 1422, 1346, 1214, 1139, 946, 831, 614 cm^{-1}.

HRMS (ESI): m/z calculated for $C_{23}H_{19}F_3NO_3S^+$ [M+H]$^+$, 446.1032, found, 446.1042.

6-Formyl-1-(isoquinolin-1-yl)naphthalen-2-yl trifluoromethanesulfonate (1i)

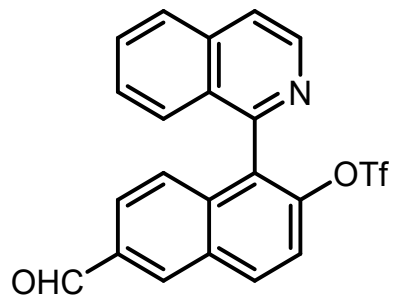

1i

Prepared following **Procedure A**, using 6-hydroxy-5-(isoquinolin-1-yl)-2-naphthaldehyde (2.99 g, 10.0 mmol, 1.0 equiv.), DMAP (122.1 mg, 1.0 mmol, 10 mol%), Pyridine (0.97 mL, 12.0 mmol, 1.2 equiv.), and Tf_2O (2.02 mL, 12.0 mmol, 1.2 equiv.). Purification by flash column chromatography (Petroleum ether/EtOAc: 10/1) to afford the title compound (3.88 g, 90%) as a colorless oil.

TLC: $R_f = 0.5$ (Petroleum ether/EtOAc: 10/1, $KMnO_4$ stain).

NMR Spectroscopy (*see spectra*):

^{1}**H NMR** (400 MHz, $CDCl_3$) δ_H = 10.18 (s, 1H), 8.78 (d, J = 5.8 Hz, 1H), 8.49 (d, J = 1.9 Hz, 1H), 8.27 (d, J = 9.0 Hz, 1H), 7.98 (d, J = 8.4 Hz, 1H), 7.91 – 7.83 (m, 2H), 7.77 – 7.68 (m, 2H), 7.46 (td, J = 7.5, 1.2 Hz, 1H), 7.39 (dd, J = 3.3, 8.6 Hz, 2H) ppm;

^{13}**C NMR** (100 MHz, $CDCl_3$) δ_C = 191.7, 153.2, 147.1, 142.8, 136.5, 136.4, 134.9, 134.0, 132.8, 132.0, 130.9, 130.1, 128.4, 128.1, 127.8, 127.4, 126.4, 124.9, 121.7, 121.0, 118.2 (q, $^1J_{C\text{-}F}$ = 320.3 Hz) ppm;

^{19}**F NMR** (376 MHz, $CDCl_3$) δ_F = -74.37 ppm.

IR (film): ν_{max} 1704, 1423, 1216, 1158, 1138, 949, 852, 830 cm^{-1}.

HRMS (ESI): m/z calculated for $C_{21}H_{13}F_3NO_4S^+$ $[M+H]^+$, 432.0512, found, 432.0511.

1-(9-Benzyl-9*H*-purin-6-yl)naphthalen-2-yl trifluoromethanesulfonate (1j)

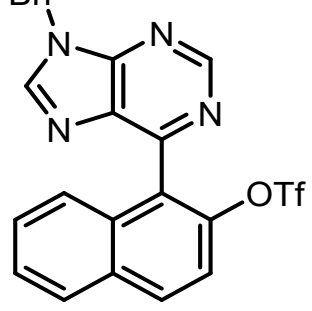

1j

Prepared following **Procedure A**, using 1-(9-benzyl-9*H*-purin-6-yl)naphthalen-2-ol (3.52 g, 10.0 mmol,

1.0 equiv.), DMAP (122.1 mg, 1.0 mmol, 10 mol%), Pyridine (0.97 mL, 12.0 mmol, 1.2 equiv.), and Tf_2O (2.02 mL, 12.0 mmol, 1.2 equiv.). Purification by flash column chromatography (Petroleum ether/EtOAc: 10/1) to afford the title compound (4.45 g, 92%) as a colorless oil.

TLC: R_f = 0.5 (Petroleum ether/EtOAc: 10/1, $KMnO_4$ stain).

NMR Spectroscopy (*see spectra*):

^{1}H NMR (500 MHz, $CDCl_3$) δ_H = 9.25 (s, 1H), 8.09 (s, 1H), 8.08 (d, J = 8.7 Hz, 1H), 7.95 (d, J = 7.2 Hz, 1H), 7.68 (d, J = 8.4 Hz, 1H), 7.59 – 7.54 (m, 2H), 7.52 – 7.47 (m, 1H), 7.40 – 7.33 (m, 5H), 5.52 (d, J = 2.6 Hz, 2H) ppm;

^{13}C NMR (125 MHz, $CDCl_3$) δ_C = 152.9, 152.3, 152.2, 145.6, 145.1, 135.0, 133.7, 132.7, 132.4, 132.0, 129.3, 128.8, 128.4, 128.2, 127.9, 127.3, 126.2, 125.7, 119.5, 118.3 (d, $^1J_{C\text{-}F}$ = 320.2 Hz), 47.6 ppm;

^{19}F NMR (376 MHz, $CDCl_3$) δ_F = -74.33 ppm.

IR (film): ν_{max} 3035, 1631, 1499, 1456, 1422, 1212, 1139, 953, 920, 834 cm^{-1}.

HRMS (ESI): m/z calculated for $C_{23}H_{16}F_3N_4O_3S^+$ $[M+H]^+$, 485.0890, found, 485.0892.

(*Z*)-Tetramethylammonium {3-[(3,7-dimethylocta-2,6-dien-1-yl)oxy]prop-1-yn-1-yl}triphenyl borate(2c)

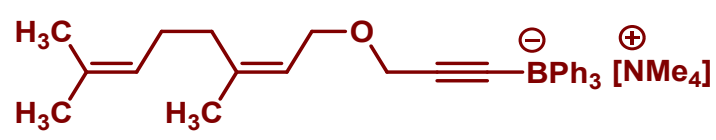

2c

Prepared following **Procedure B**, using (*Z*)-3,7-dimethyl-1-(prop-2-yn-1-yloxy)octa-2,6-diene (1.92 g, 10.0 mmol, 1.0 equiv.), *n*-BuLi (1.6 M in *n*-hexane, 6.9 mL, 11.0 mmol, 1.1 equiv.), $Ph_3B{\cdot}Py$ (3.53 g, 11.0 mmol, 1.1 equiv.), and Me_4NCl (2.19 g, 20 mmol, 2.0 equiv.). It was collected by filtration and washed with cold MeOH to afford the title compound (4.1 g, 80%) as a white solid.

M. p.:58 – 59 °C.

NMR Spectroscopy (*see spectra*):

^{1}H NMR (400 MHz, d_6-DMSO) δ_H = 7.28 (d, J = 7.5 Hz, 6H), 6.93 (t, J = 7.5 Hz, 6H), 6.80 (t, J

= 7.2 Hz, 3H), 5.28 (s, 1H), 5.08 (d, J = 9.0 Hz, 1H), 4.09 (s, 4H), 3.08 (s, 12H), 2.17 – 1.89 (m, 4H), 1.64 (s, 6H), 1.57 (s, 3H) ppm;

^{13}C NMR (126 MHz, d_6-DMSO) δ_C = 138.5, 134.3, 130.9, 125.5, 123.9, 122.1, 121.5, 64.2, 58.4, 54.3, 54.3, 54.3, 25.9, 25.5, 17.5, 16.2.ppm. The carbon attached to boron was not observed due to quadrupolar relaxation;

^{11}B NMR (160 MHz, d_6-DMSO) δ_B = -12.40 ppm.

IR (film): ν_{max} 3057, 3012, 2990, 2361, 1581, 1480, 1429, 1134, 945, 747, 700, 617 cm^{-1}.

(*R*)-4,4,5-Triphenyl-4*H*-3l4,4l4-naphtho[2',1':5,6][1,2]azaborepino[7,1-a]isoquinoline (3)

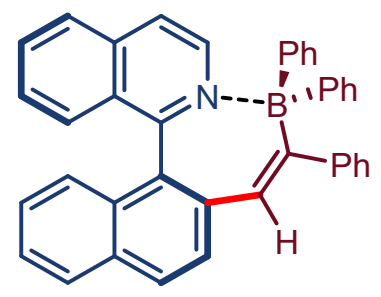

3

Prepared following **Procedure C**, using 1-(isoquinolin-1-yl)naphthalen-2-yl trifluoromethanesulfonate (80.6 mg, 0.2 mmol, 1.0 equiv.), tetramethylammonium ethynyltriphenylborate (102.3 mg, 0.3 mmol, 1.5 equiv.), Pd(acac)$_2$ (1.5 mg, 2.5 mol%), and (*R*)-**L4** (11.0 mg, 7.5 mol%). Purification by flash column chromatography (Petroleum ether/EtOAc: 50/1) to afford the title compound (100.0 mg, 96%) as a yellow solid.

TLC: R_f = 0.5 (Petroleum ether/EtOAc: 20/1, $KMnO_4$ stain).

M. p.: 150 – 151 °C.

[α]$^{18}_D$: +263.89 (c 0.20, CH_2Cl_2).

NMR Spectroscopy (*see spectra*):

^{1}H NMR (400 MHz, $CDCl_3$) δ_H = 8.83 (d, J = 7.0 Hz, 1H), 7.95 (d, J = 10.6 Hz, 1H), 7.78 (t, J = 5.5 Hz, 2H), 7.70 (d, J = 10.6 Hz, 1H), 7.66 (d, J = 10.4 Hz, 1H), 7.38 (d, J = 8.6 Hz, 1H), 7.32 (t, J = 8.1 Hz, 1H), 7.26 (t, J = 7.5 Hz, 1H), 7.21 (s, 2H), 7.15 – 7.00 (m, 8H), 7.00 – 6.77 (m, 5H), 6.73 – 6.42 (m, 4H) ppm;

^{13}C NMR (100 MHz, $CDCl_3$) δ_C = 158.1, 151.3, 139.9, 138.7, 138.2, 136.3, 135.4, 132.9, 132.7,

131.3, 130.5, 130.3, 129.4, 128.5, 128.2, 128.2, 127.7, 127.3, 126.8, 126.5, 126.4, 126.1, 125.7, 125.4, 124.8, 124.8, 123.7, 121.3 ppm. The carbon attached to boron was not observed due to quadrupolar relaxation;

^{11}B NMR (128 MHz, $CDCl_3$) δ_B = 3.54 ppm.

IR (film): ν_{max} 3768, 3043, 2997, 2779, 1709, 1624, 1503, 1486, 1429, 1318, 862, 702 cm^{-1}.

HRMS (ESI): m/z calculated for $C_{39}H_{29}BN^+$ $[M+H]^+$, 522.2388, found, 522.2389.

HPLC analysis: HPLC conditions: Chiral column IB, *n*-hexane/isopropanol: 99/1, flow rate = 1.0 mL/min, wavelength = 254 nm, t_R = 8.130 min for major isomer, t_R = 9.343 min for minor isomer, 99% ee.

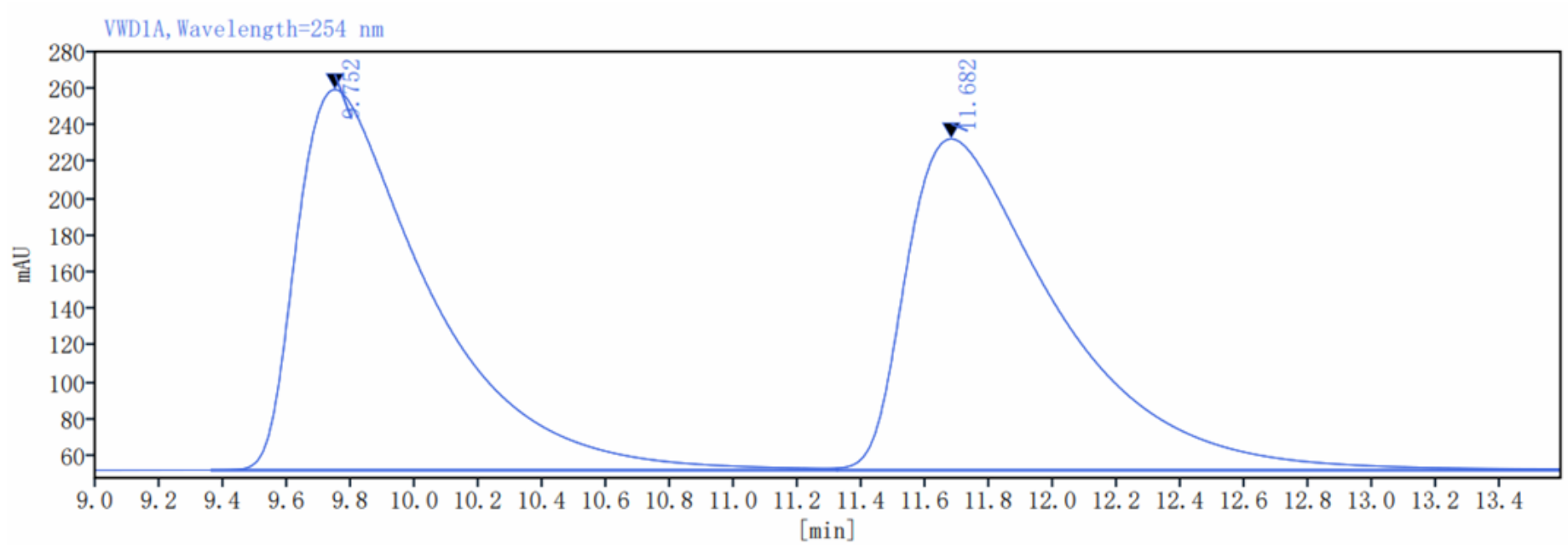

信号: VWD1A, Wavelength=254 nm

Retention Time [min]	Int Type	Width [min]	Area	Height	Area%
9.752	MM m	1.96	5954.05	207.01	49.87
11.682	MB m	2.92	5984.98	180.17	50.13

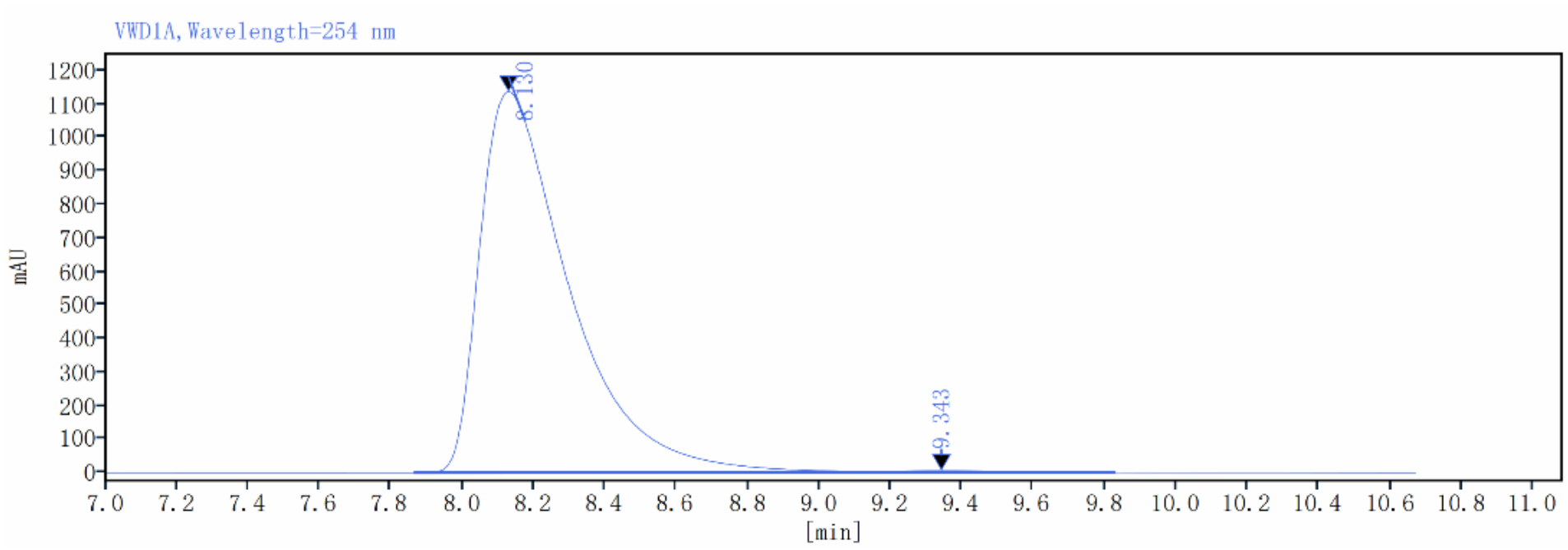

信号: VWD1A, Wavelength=254 nm

Retention Time [min]	Int Type	Width [min]	Area	Height	Area%
8.130	BM m	1.29	19839.16	1135.89	99.42
9.343	MM m	0.67	116.31	5.34	0.58

(*R*)-15-Isopropyl-4,4,5-triphenyl-4*H*-3l4,4l4-naphtho[2',1':5,6][1,2]azaborepino[7,1-a]isoquinoline (4)

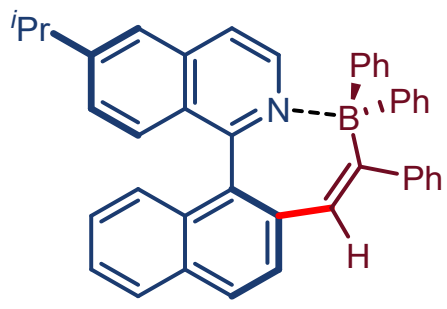

4

Prepared following **Procedure C**, using 1-(6-isopropylisoquinolin-1-yl)naphthalen-2-yl trifluoromethanesulfonate (89.0 mg, 0.2 mmol, 1.0 equiv.), tetramethylammonium ethynyltriphenylborate (102.3 mg, 0.3 mmol, 1.5 equiv.), $Pd(acac)_2$ (1.5 mg, 2.5 mol%), and (*R*)-**L4** (11.0 mg, 7.5 mol%). Purification by flash column chromatography (Petroleum ether/EtOAc: 50/1) to afford the title compound (101.3 mg, 90%) as a yellow solid.

TLC: R_f = 0.5 (Petroleum ether/EtOAc: 50/1, $KMnO_4$ stain).

M. p.: 113 – 114 °C.

$[\alpha]^{18}_D$: +299.37 (c 0.20, CH_2Cl_2).

NMR Spectroscopy (*see spectra*):

^{1}H NMR (400 MHz, $CDCl_3$) δ_H = 8.73 (d, *J* = 6.9 Hz, 1H), 7.70 (d, *J* = 7.4 Hz, 2H), 7.65 (d, *J* = 8.5 Hz, 1H), 7.62 (d, *J* = 8.0 Hz, 1H), 7.33 (d, *J* = 8.4 Hz, 1H), 7.24 – 7.17 (m, 2H), 7.13 (s, 1H), 7.09 – 6.98 (m, 9H), 6.93 – 6.76 (m, 5H), 6.63 – 6.42 (m, 4H), 3.07 (sept, *J* = 7.0 Hz, 1H), 1.31 (t, *J* = 6.4 Hz, 6H) ppm;

^{13}C NMR (100 MHz, $CDCl_3$) δ_C = 157.5, 154.2, 151.4, 139.9, 138.7, 138.2, 136.8, 135.4, 132.9, 131.2, 130.5, 129.3, 129.0, 128.7, 128.2, 127.6, 127.5, 127.4, 126.8, 126.4, 126.2, 125.7, 125.3, 124.8, 124.7, 123.6, 122.7, 121.0, 34.5, 23.6, 23.4 ppm. The carbon attached to boron was not observed due to quadrupolar relaxation;

^{11}B NMR (128 MHz, $CDCl_3$) δ_B = 3.06 ppm.

IR (film): ν_{max} 3686, 3044, 2897, 2765, 1731, 1488, 1301, 1000, 883, 740, 703 cm^{-1}.

HRMS (ESI): m/z calculated for $C_{42}H_{35}BN^+$ $[M+H]^+$, 564.2857, found, 564.2855.

HPLC analysis: HPLC conditions: Chiral column OD–H, *n*-hexane/isopropanol: 99/1, flow rate = 0.8

mL/min, wavelength = 254 nm, t_R = 11.153 min for major isomer, t_R = 10.073 min for minor isomer, 97% ee.

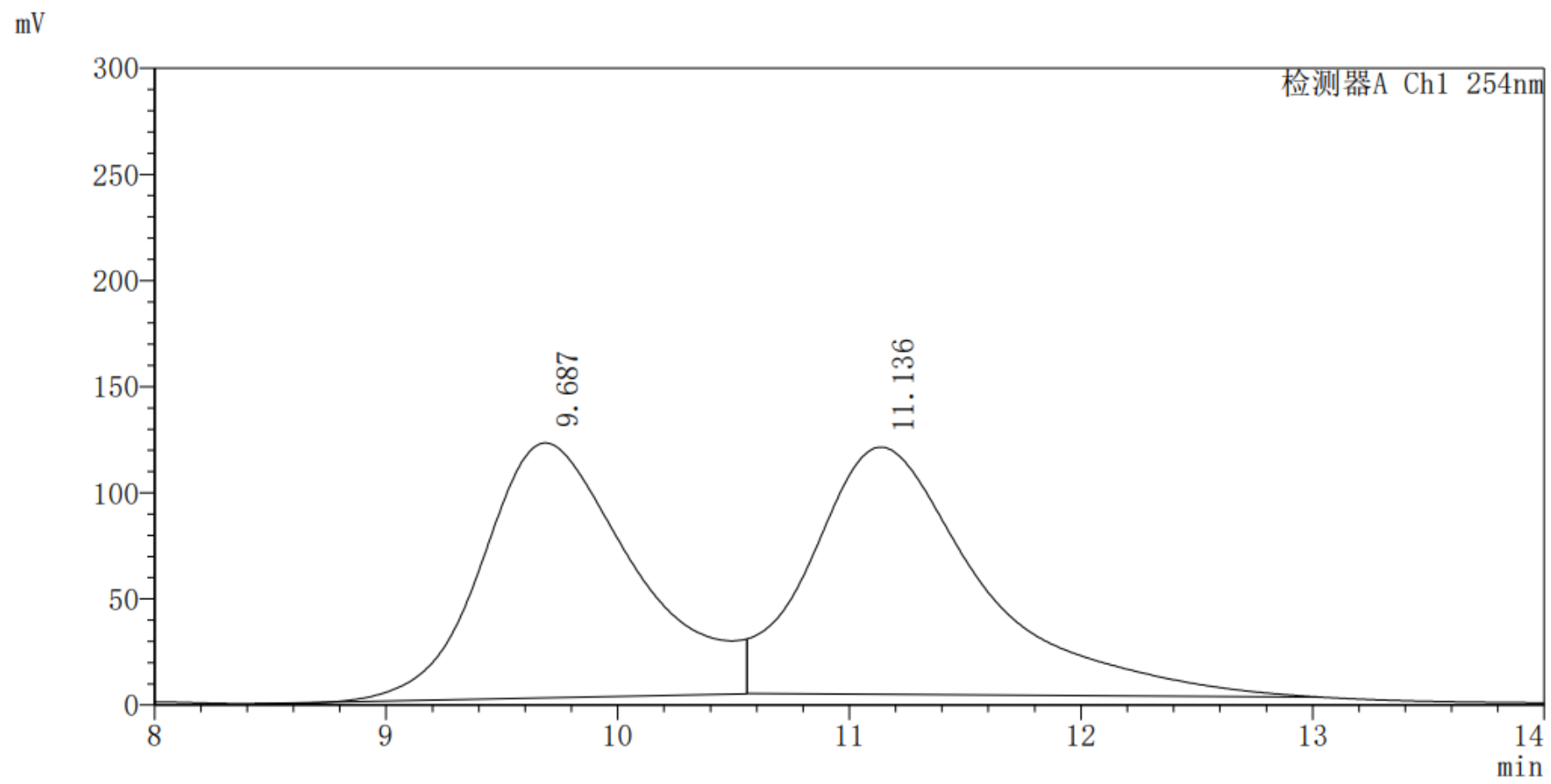

检测器A Ch1 254nm

No.	R.Time	Height	Height%	Area	Area%
1	9.687	120145	50.788	5503915	48.046
2	11.136	116415	49.212	5951592	51.954

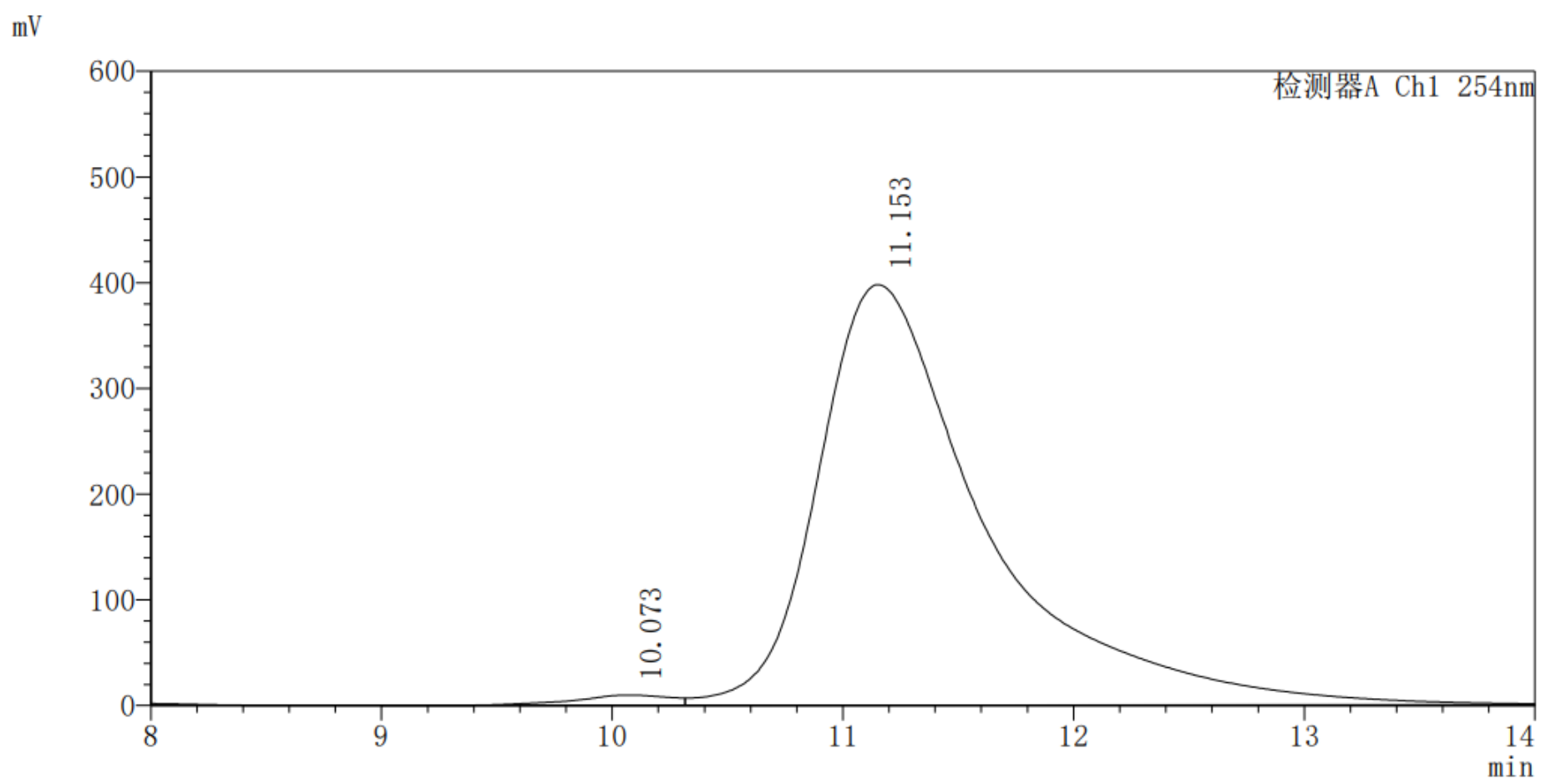

检测器A Ch1 254nm

No.	R.Time	Height	Height%	Area	Area%
1	10.073	10054	2.464	305943	1.493
2	11.153	398042	97.536	20191399	98.507

(*R*)-16-Methoxy-4,4,5-triphenyl-4*H*-3l4,4l4-naphtho[2',1':5,6][1,2]azaborepino[7,1-a]isoquinoline (5)

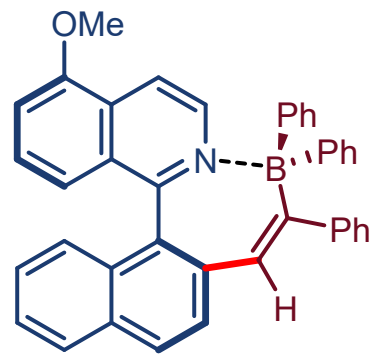

5

Prepared following **Procedure C**, using 1-(5-methoxyisoquinolin-1-yl)naphthalen-2-yl trifluoromethanesulfonate (86.6 mg, 0.2 mmol, 1.0 equiv.), tetramethylammonium ethynyltriphenylborate (102.3 mg, 0.3 mmol, 1.5 equiv.), $Pd(acac)_2$ (1.5 mg, 2.5 mol%), and (*R*)-**L4** (11.0 mg, 7.5 mol%). Purification by flash column chromatography (Petroleum ether/EtOAc: 50/1) to afford the title compound (106.9 mg, 97%) as a yellow solid.

TLC: R_f = 0.5 (Petroleum ether/EtOAc: 50/1, $KMnO_4$ stain).

M. p.: 261 – 262 °C.

$[\alpha]^{18}_D$: +274.59 (c 0.20, CH_2Cl_2).

NMR Spectroscopy (*see spectra*):

^{1}**H NMR** (500 MHz, $CDCl_3$) δ_H = 8.83 (d, *J* = 7.0 Hz, 1H), 8.23 (d, *J* = 7.0 Hz, 1H), 7.69 (d, *J* = 8.5 Hz, 1H), 7.65 (d, *J* = 8.1 Hz, 1H), 7.38 (d, *J* = 8.5 Hz, 1H), 7.28 – 7.24 (m, 1H), 7.19 (t, *J* = 4.2 Hz, 2H), 7.16 – 7.00 (m, 9H), 6.99 – 6.77 (m, 5H), 6.72 (d, *J* = 8.7 Hz, 1H), 6.69 – 6.45 (m, 4H), 4.06 (s, 3H) ppm;

^{13}**C NMR** (100 MHz, $CDCl_3$) δ_C = 157.3, 154.2, 151.3, 139.7, 138.7, 137.8, 135.5, 132.8, 131.2, 130.4, 129.3, 129.1, 128.7, 128.1, 127.7, 127.6, 127.3, 126.8, 126.3, 126.1, 125.6, 125.3, 124.8, 124.7, 123.6, 122.5, 116.0, 109.4, 56.1 ppm. The carbon attached to boron was not observed due to quadrupolar relaxation;

^{11}**B NMR** (128 MHz, $CDCl_3$) δ_B = 4.01 ppm.

IR (film): ν_{max} 3677, 3048, 2875, 1719, 1502, 1317, 1265, 1051, 889, 739, 705 cm^{-1}.

HRMS (ESI): m/z calculated for $C_{40}H_{31}BNO^+$ $[M+H]^+$, 552.2493, found, 552.2490.

HPLC analysis: HPLC conditions: Chiral column AD–H, *n*-hexane/isopropanol: 99/1, flow rate = 1.0

mL/min, wavelength = 254 nm, t_R = 6.801 min for major isomer, t_R = 17.650 min for minor isomer, 92% ee.

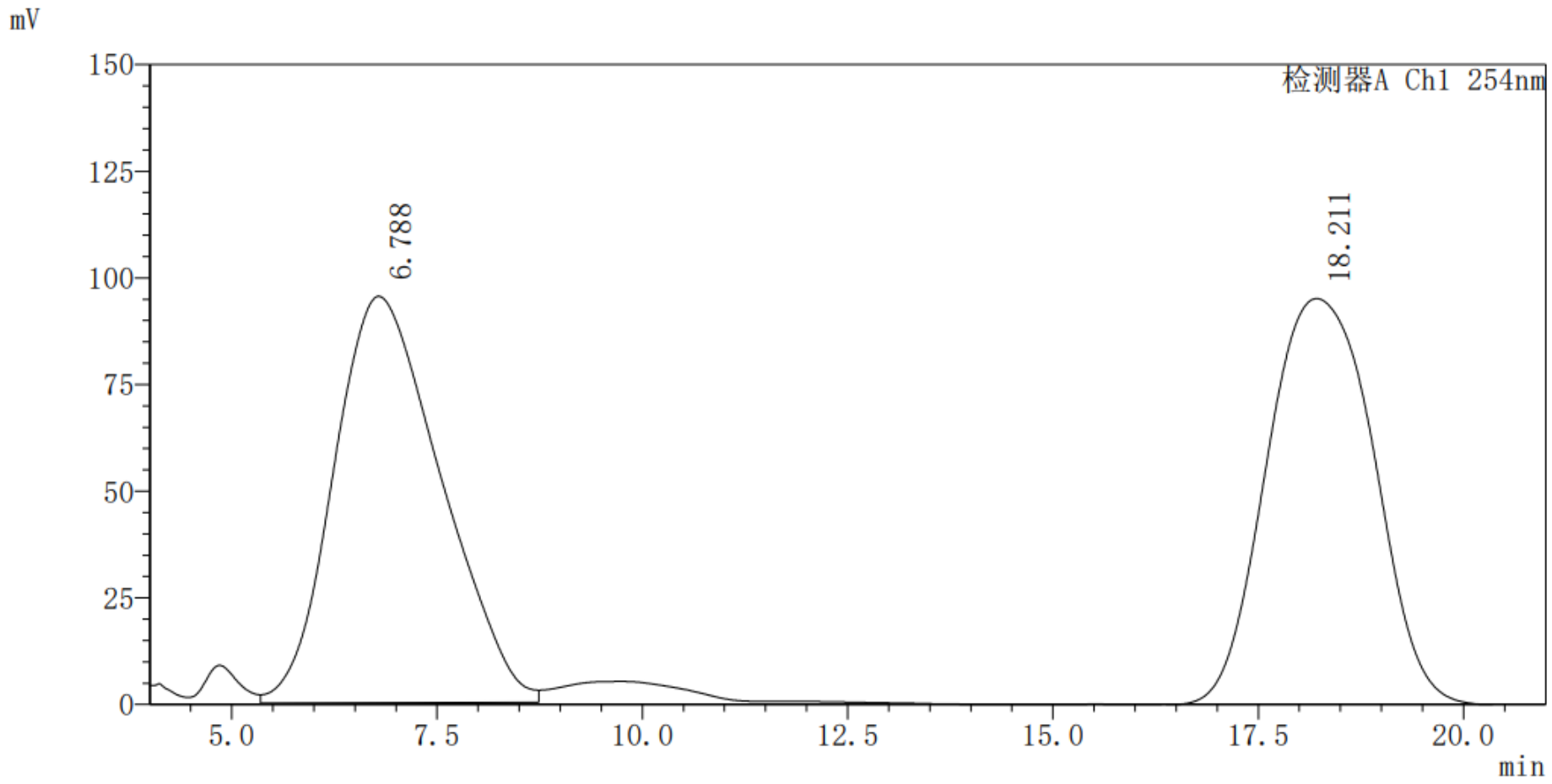

检测器A Ch1 254nm

No.	R.Time	Height	Height%	Area	Area%
1	6.788	95307	50.000	8658937	49.912
2	18.211	95308	50.000	8689427	50.088

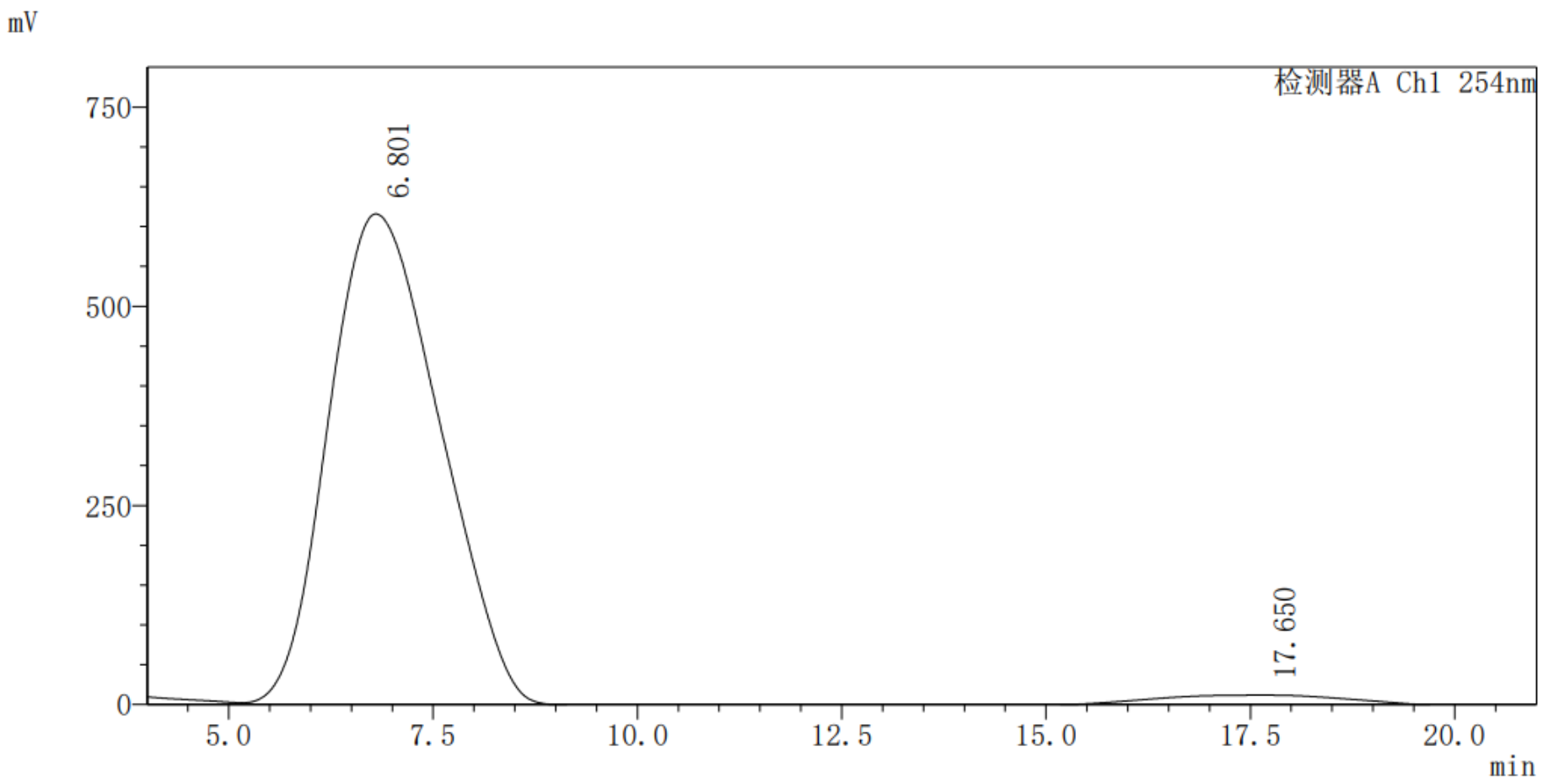

检测器A Ch1 254nm

No.	R.Time	Height	Height%	Area	Area%
1	6.801	616990	97.810	58356598	95.984
2	17.650	13813	2.190	2441561	4.016

(*R*)-4,4,5,16-Tetraphenyl-4*H*-3l4,4l4-naphtho[2',1':5,6][1,2]azaborepino[7,1-a]isoquinoline (6)

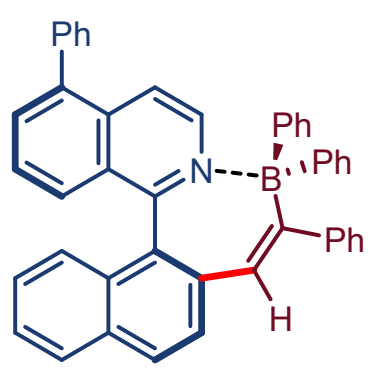

6

Prepared following **Procedure C**, using 1-(5-phenylisoquinolin-1-yl)naphthalen-2-yl trifluoromethanesulfonate (95.8 mg, 0.2 mmol, 1.0 equiv.), tetramethylammonium ethynyltriphenylborate (102.3 mg, 0.3 mmol, 1.5 equiv.), $Pd(acac)_2$ (1.5 mg, 2.5 mol%), and (*R*)-**L4** (11.0 mg, 7.5 mol%). Purification by flash column chromatography (Petroleum ether/EtOAc: 50/1) to afford the title compound (115.9 mg, 97%) as a yellow solid.

TLC: R_f = 0.5 (Petroleum ether/EtOAc: 50/1, $KMnO_4$ stain).

M. p.: 190 – 191 °C.

$[\alpha]^{18}_D$: +128.25 (c 0.20, CH_2Cl_2).

NMR Spectroscopy (*see spectra*):

^{1}H NMR (500 MHz, $CDCl_3$) δ_H = 8.74 (d, *J* = 7.2 Hz, 1H), 7.90 (d, *J* = 7.2 Hz, 1H), 7.71 (d, *J* = 8.1 Hz, 2H), 7.67 (d, *J* = 7.8 Hz, 1H), 7.59 – 7.50 (m, 5H), 7.40 (d, *J* = 8.4 Hz, 1H), 7.34 (dd, *J* = 7.0, 8.9 Hz, 1H), 7.28 (t, *J* = 7.4 Hz, 1H), 7.22 (t, *J* = 4.3 Hz, 2H), 7.20 – 6.79 (m, 13H), 6.78 – 6.47 (m, 4H) ppm;

^{13}C NMR (126 MHz, $CDCl_3$) δ_C = 158.2, 151.3, 140.0, 139.4, 138.6, 138.3, 135.5, 134.8, 133.0, 130.7, 130.7, 130.5, 130.0, 129.4, 128.9, 128.4, 128.2, 128.0, 127.7, 127.5, 127.4, 126.8, 126.4, 126.2, 125.8, 125.4, 124.9, 124.8, 123.7, 119.5 ppm. The carbon attached to boron was not observed due to quadrupolar relaxation;

^{11}B NMR (160 MHz, $CDCl_3$) δ_B = 5.93 ppm.

IR (film): ν_{max} 3678, 3043, 2779, 1671, 1487, 1429, 1317, 909, 818, 701, 658 cm^{-1}.

HRMS (ESI): m/z calculated for $C_{45}H_{33}BN^+$ $[M+H]^+$, 598.2701, found, 598.2696.

HPLC analysis: HPLC conditions: Chiral column OD–H, *n*-hexane/isopropanol: 97/3, flow rate = 1.0 mL/min, wavelength = 254 nm, t_R = 7.113 min for major isomer, t_R = 11.832 min for minor isomer, 99% ee.

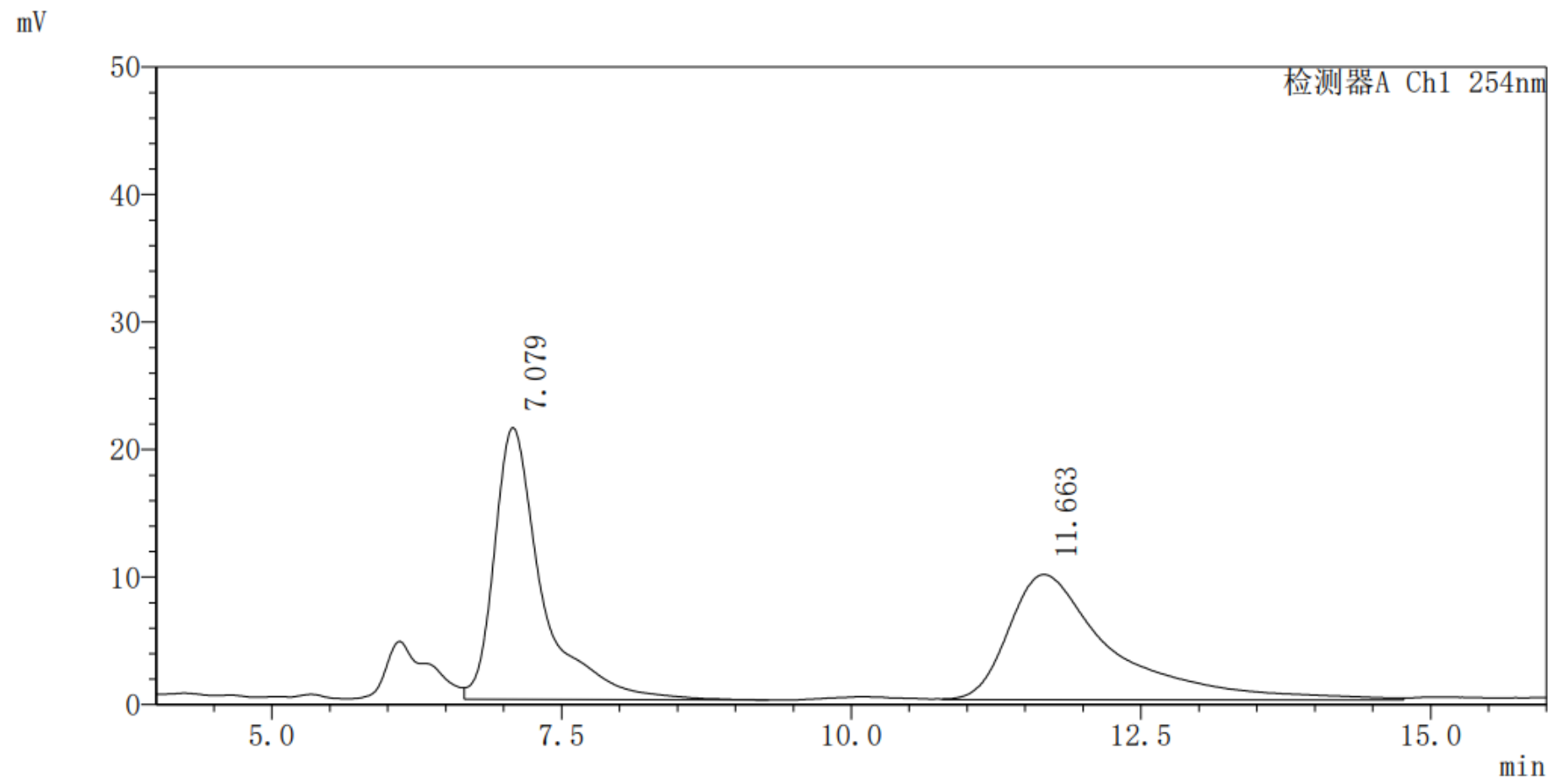

检测器A Ch1 254nm

No.	R.Time	Height	Height%	Area	Area%
1	7.079	21299	68.465	613447	50.205
2	11.663	9810	31.535	608429	49.795

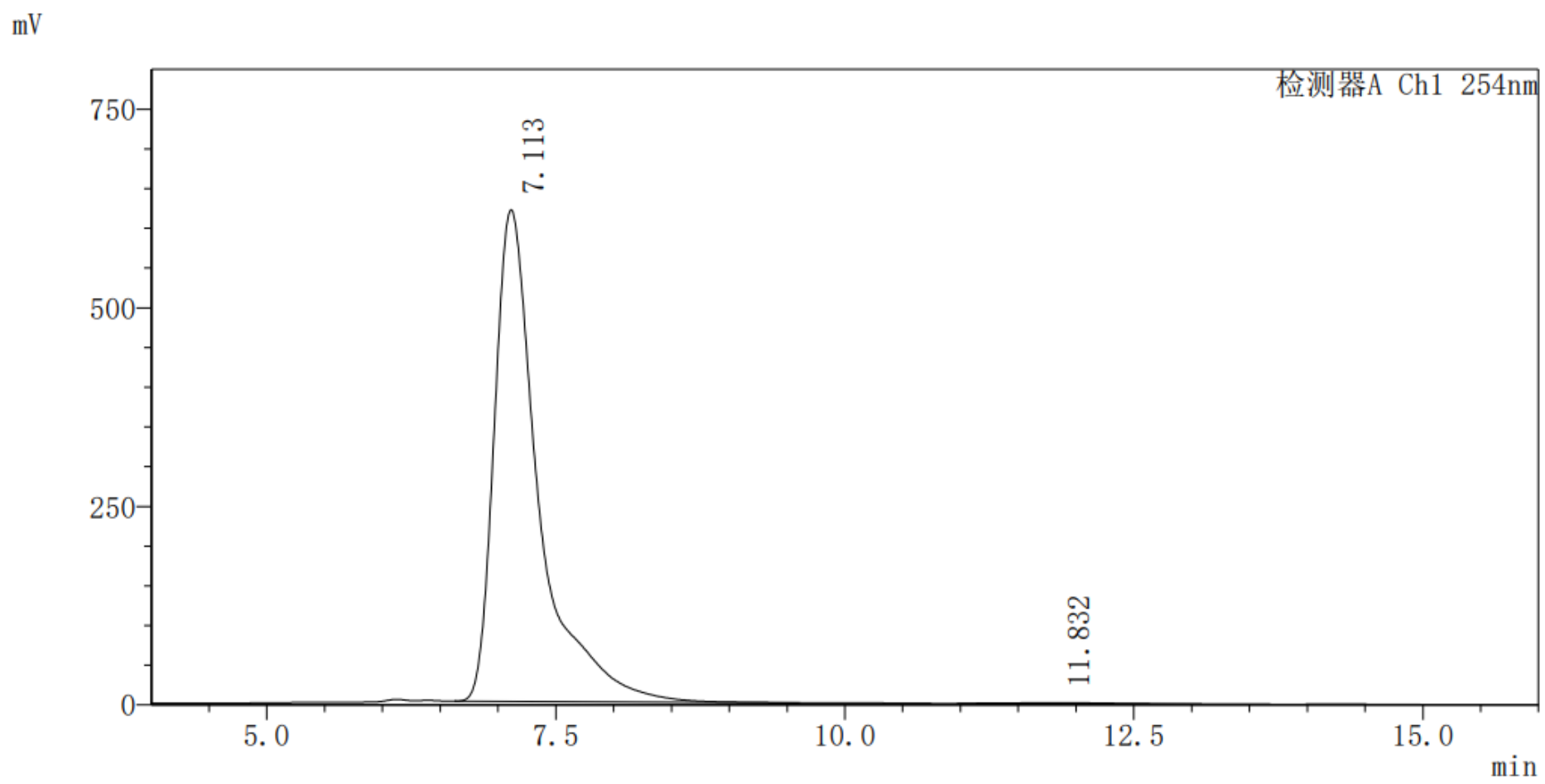

检测器A Ch1 254nm

No.	R.Time	Height	Height%	Area	Area%
1	7.113	618771	99.826	16612247	99.708
2	11.832	1078	0.174	48659	0.292

(*R*)-15-Chloro-4,4,5-triphenyl-4*H*-3l4,4l4-naphtho[2',1':5,6][1,2]azaborepino[7,1-a]isoquinoline (7)

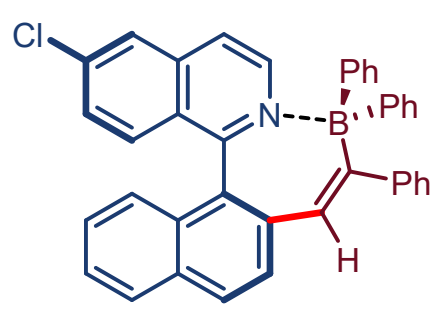

7

Prepared following **Procedure C**, using 1-(6-chloroisoquinolin-1-yl)naphthalen-2-yl trifluoromethanesulfonate (87.4 mg, 0.2 mmol, 1.0 equiv.), tetramethylammonium

ethynyltriphenylborate (102.3 mg, 0.3 mmol, 1.5 equiv.), Pd(acac)$_2$ (1.5 mg, 2.5 mol%), and (*R*)-**L4** (11.0 mg, 7.5 mol%). Purification by flash column chromatography (Petroleum ether/EtOAc: 50/1) to afford the title compound (89.9 mg, 81%) as a yellow solid.

TLC: R_f = 0.5 (Petroleum ether/EtOAc: 50/1, $KMnO_4$ stain).

M. p.: 251 – 252 °C.

$[\alpha]^{18}_D$: +122.54 (c 0.20, CH_2Cl_2).

NMR Spectroscopy (*see spectra*):

^{1}H NMR (400 MHz, $CDCl_3$) δ_H = 8.80 (d, *J* = 7.0 Hz, 1H), 7.92 (d, *J* = 2.1 Hz, 1H), 7.67 (d, *J* = 7.8 Hz, 2H), 7.63 (d, *J* = 7.4 Hz, 1H), 7.35 (d, *J* = 8.5 Hz, 1H), 7.26 – 7.17 (m, 2H), 7.15 (s, 1H), 7.04 (td, *J* = 7.9, 15.4 Hz, 9H), 6.95 – 6.78 (m, 5H), 6.65 – 6.41 (m, 4H) ppm;

^{13}C NMR (100 MHz, $CDCl_3$) δ_C = 158.3, 151.2, 140.1, 139.5, 139.4, 138.7, 137.1, 135.4, 133.1, 132.7, 130.5, 129.7, 129.7, 128.6, 128.2, 127.8, 127.1, 126.9, 126.8, 126.5, 126.2, 126.0, 125.5, 125.3, 125.0, 124.9, 123.8, 120.3 ppm. The carbon attached to boron was not observed due to quadrupolar relaxation;

^{11}B NMR (128 MHz, $CDCl_3$) δ_B = 3.83 ppm.

IR (film): ν_{max} 3679, 3063, 2779, 1617, 1545, 1486, 1353, 1171, 1090, 889, 880, 744, 705, 665 cm^{-1}.

HRMS (ESI): m/z calculated for $C_{39}H_{28}BClN^+$ $[M+H]^+$, 556.1998, found, 556.2001.

HPLC analysis: HPLC conditions: Chiral column OD-H, *n*-hexane/isopropanol: 99/1, flow rate = 1.0 mL/min, wavelength = 254 nm, t_R = 18.892 min for major isomer, t_R = 20.863 min for minor isomer, 99% ee.

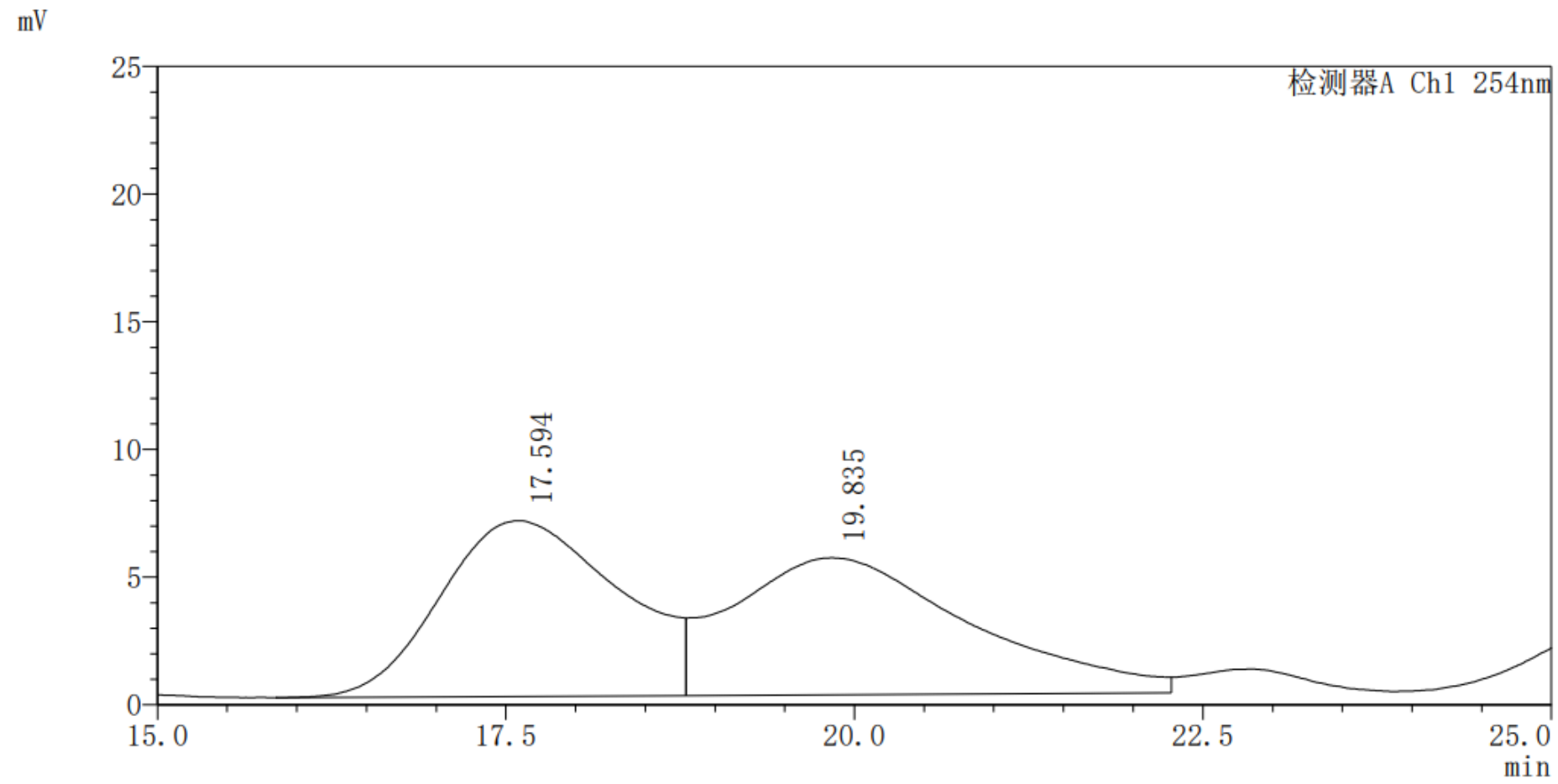

检测器A Ch1 254nm

No.	R.Time	Height	Height%	Area	Area%
1	17.594	6883	56.231	614132	49.109
2	19.835	5358	43.769	636408	50.891

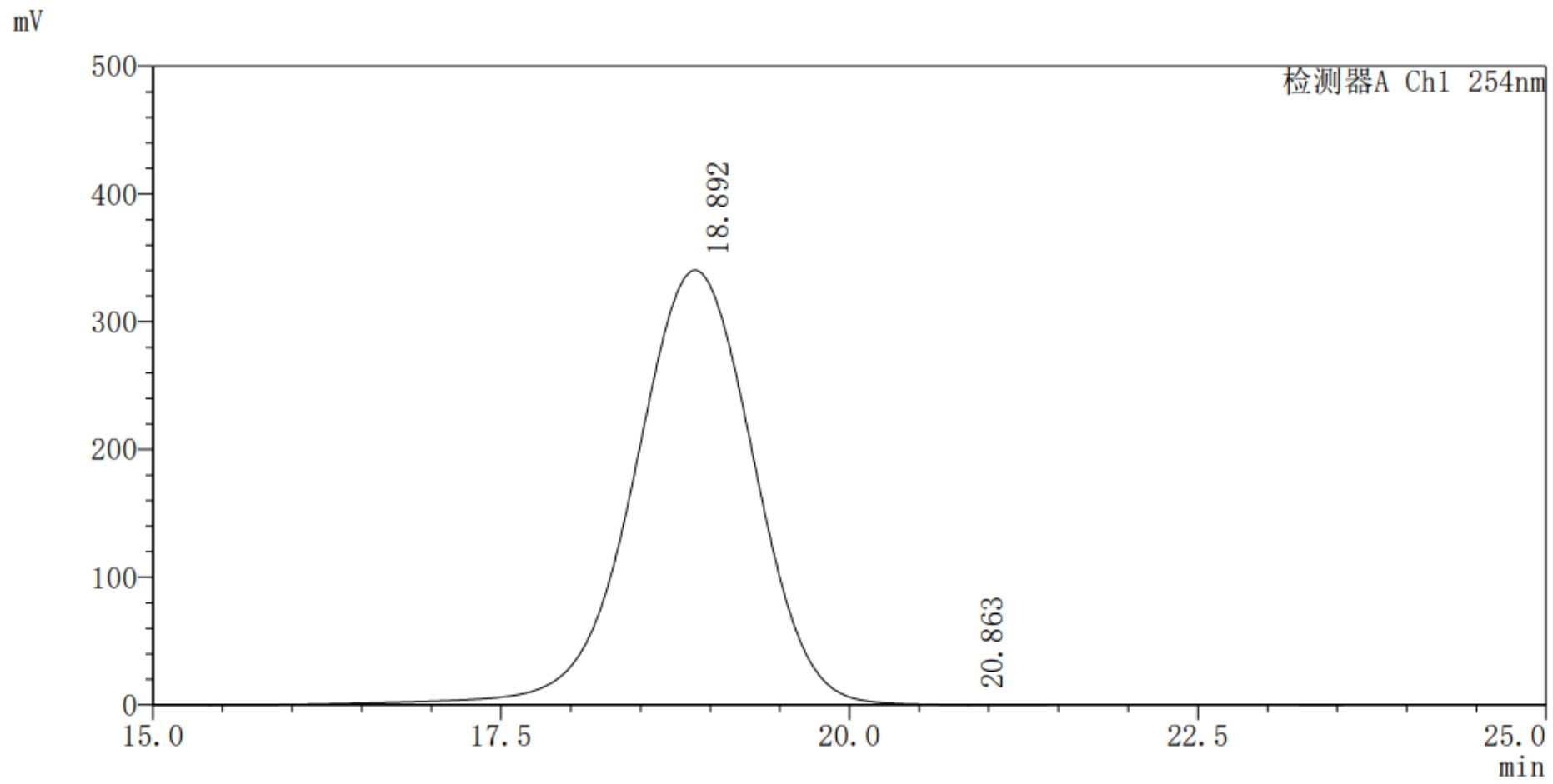

检测器A Ch1 254nm

No.	R.Time	Height	Height%	Area	Area%
1	18.892	340855	99.780	20579039	99.849
2	20.863	753	0.220	31185	0.151

(*R*)-1-Chloro-4,4,5-triphenyl-4*H*-3l4,4l4-naphtho[2',1':5,6][1,2]azaborepino[7,1-a]isoquinoline (8)

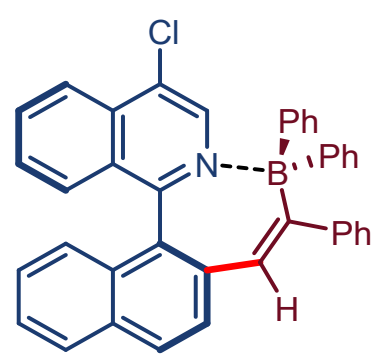

8

Prepared following **Procedure C**, using 1-(4-chloroisoquinolin-1-yl)naphthalen-2-yl trifluoromethanesulfonate (87.4 mg, 0.2 mmol, 1.0 equiv.), tetramethylammonium ethynyltriphenylborate (102.3 mg, 0.3 mmol, 1.5 equiv.), Pd(acac)$_2$ (1.5 mg, 2.5 mol%), and (*R*)-**L4** (11.0

mg, 7.5 mol%). Purification by flash column chromatography (Petroleum ether/EtOAc: 50/1) to afford the title compound (101.0 mg, 91%) as a yellow solid.

TLC: R_f = 0.5 (Petroleum ether/EtOAc: 50/1, $KMnO_4$ stain).

M. p.: 146 – 147 °C.

$[\alpha]^{18}_D$: +211.24 (c 0.20, CH_2Cl_2).

NMR Spectroscopy (*see spectra*):

^{1}H NMR (400 MHz, $CDCl_3$) δ_H = 9.01 – 8.90 (m, 1H), 8.34 (d, *J* = 8.5 Hz, 1H), 7.87 (t, *J* = 7.7 Hz, 1H), 7.71 (dd, *J* = 8.5, 4.3 Hz, 1H), 7.65 (dd, *J* = 8.3, 3.2 Hz, 1H), 7.43 – 7.32 (m, 2H), 7.29 – 7.19 (m, 3H), 7.17 – 7.01 (m, 8H), 7.01 – 6.77 (m, 5H), 6.73 – 6.45 (m, 4H) ppm;

^{13}C NMR (100 MHz, $CDCl_3$) δ_C = 157.3, 151.0, 140.2, 138.6, 137.0, 135.5, 134.2, 133.7, 132.8, 131.9, 130.7, 130.5, 129.7, 129.3, 129.0, 128.2, 127.7, 127.2, 126.9, 126.7, 126.6, 126.1, 125.9, 125.8, 125.0, 124.9, 123.8, 123.5 ppm. The carbon attached to boron was not observed due to quadrupolar relaxation;

^{11}B NMR (128 MHz, $CDCl_3$) δ_B = 5.57 ppm.

IR (film): ν_{max} 3692, 3678, 3044, 2998, 2779, 2708, 1850, 1622, 1541, 1373, 1353, 880 cm^{-1}.

HRMS (ESI): m/z calculated for $C_{39}H_{28}BClN^+$ $[M+H]^+$, 556.1998, found, 556.1995.

HPLC analysis: HPLC conditions: Chiral column AD–H, *n*-hexane/isopropanol: 99/1, flow rate = 1.0 mL/min, wavelength = 254 nm, t_R = 4.399 min for major isomer, t_R = 6.247 min for minor isomer, 94% ee.

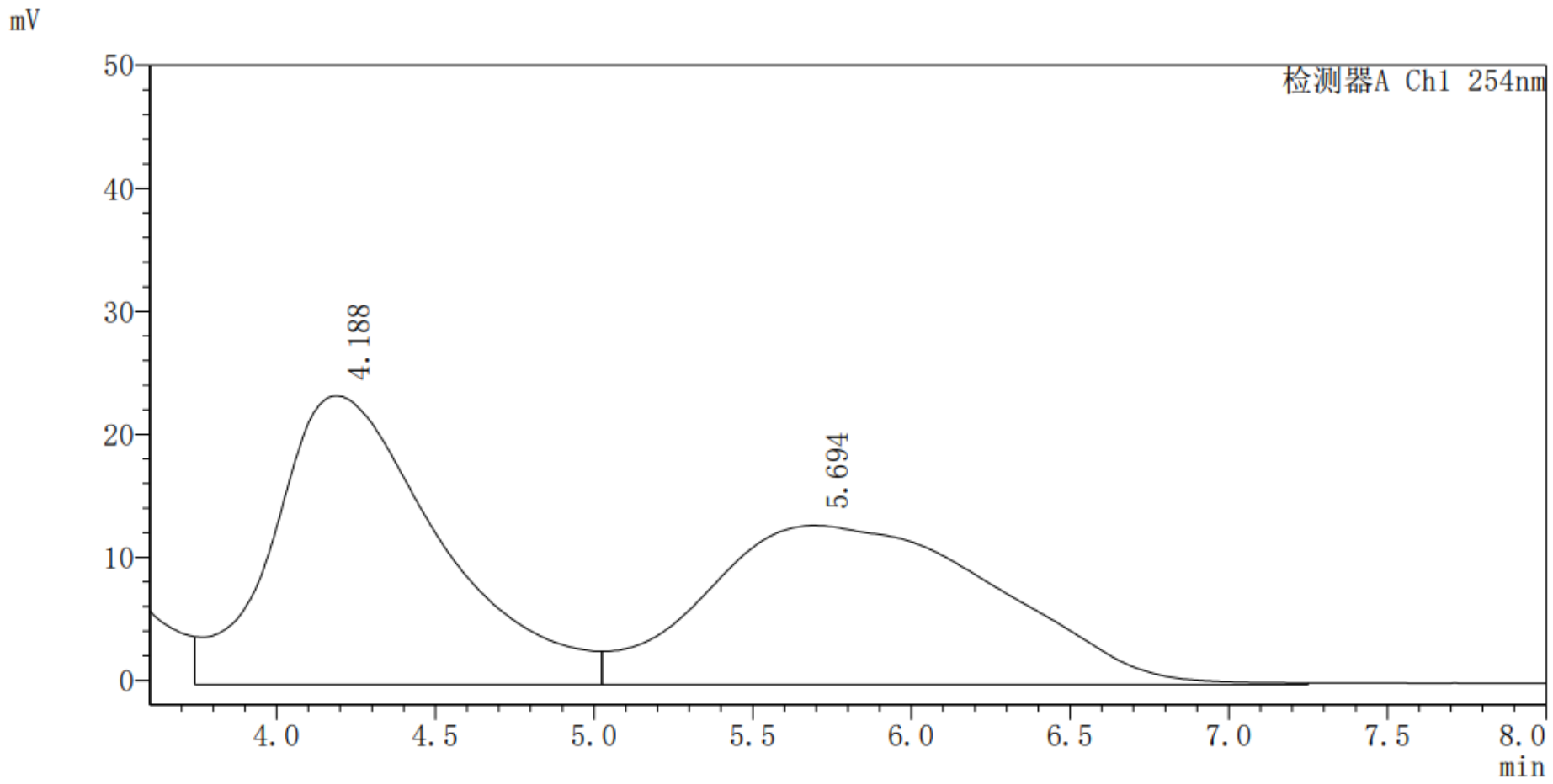

检测器A Ch1 254nm

No.	R.Time	Height	Height%	Area	Area%
1	4.188	23484	64.498	857882	50.882
2	5.694	12926	35.502	828130	49.118

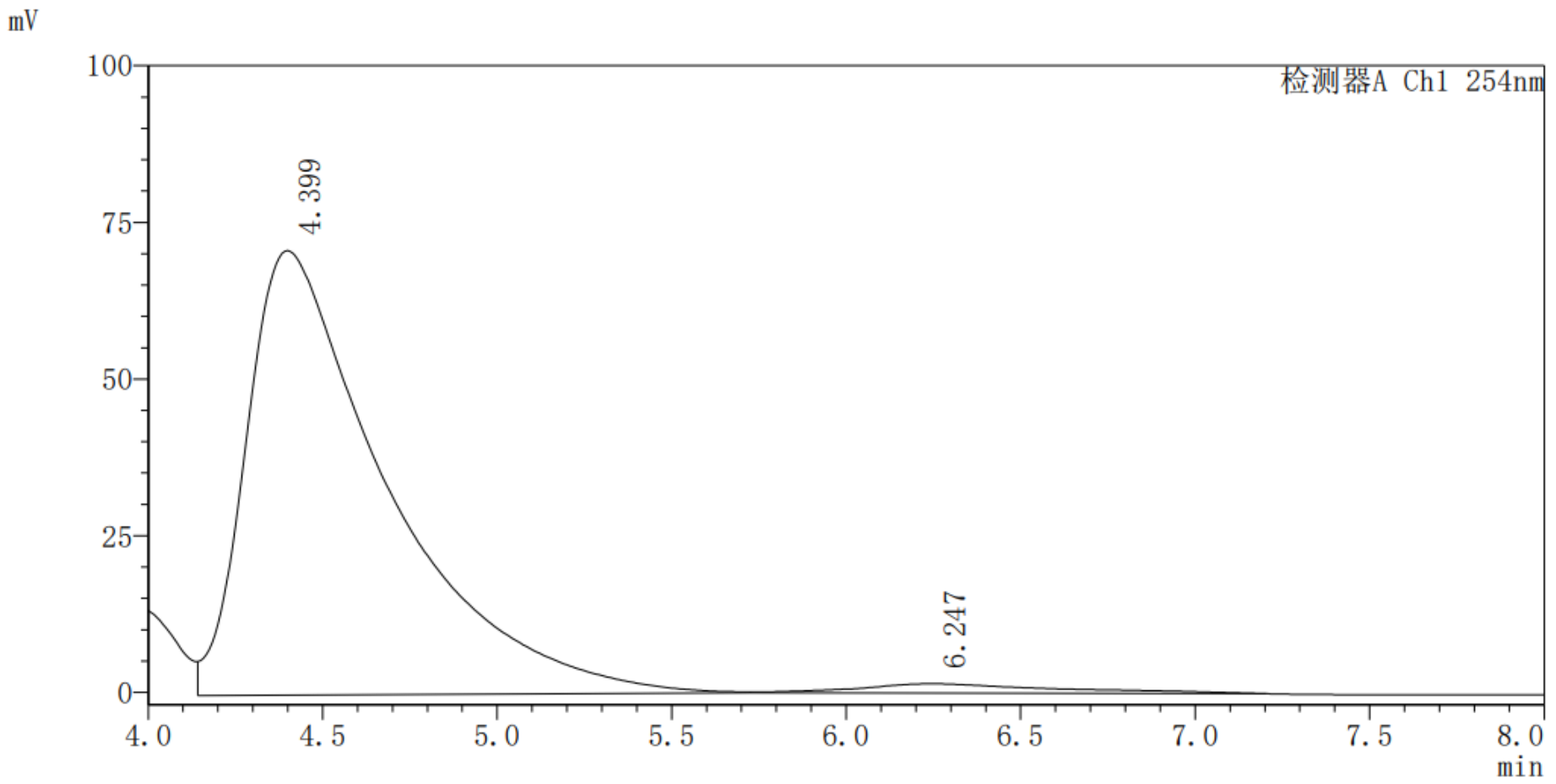

检测器A Ch1 254nm

No.	R.Time	Height	Height%	Area	Area%
1	4.399	70920	97.937	1995759	97.098
2	6.247	1494	2.063	59657	2.902

(*R*)-16-Nitro-4,4,5-triphenyl-4*H*-3l4,4l4-naphtho[2',1':5,6][1,2]azaborepino[7,1-a]isoquinoline (9)

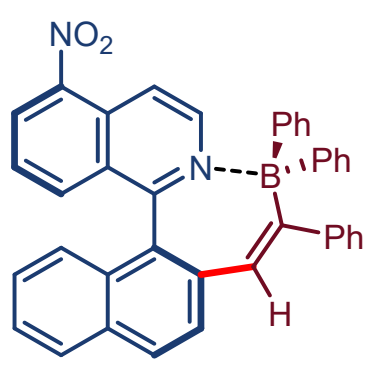

9

Prepared following **Procedure C**, using 1-(5-nitroisoquinolin-1-yl)naphthalen-2-yl trifluoromethanesulfonate (89.6 mg, 0.2 mmol, 1.0 equiv.), tetramethylammonium

ethynyltriphenylborate (102.3 mg, 0.3 mmol, 1.5 equiv.), Pd(acac)$_2$ (1.5 mg, 2.5 mol%), and (*R*)-**L4** (11.0 mg, 7.5 mol%). Purification by flash column chromatography (Petroleum ether/EtOAc: 50/1) to afford the title compound (111.0 mg, 98%) as a yellow solid.

TLC: $R_f = 0.5$ (Petroleum ether/EtOAc: 20/1, $KMnO_4$ stain).

M. p.: 99 – 100 °C.

$[\alpha]^{18}_{D}$: +93.91 (c 0.20, CH_2Cl_2).

NMR Spectroscopy (*see spectra*):

^{1}H NMR (400 MHz, $CDCl_3$) δ_H = 9.07 (d, *J* = 7.4 Hz, 1H), 8.69 (d, *J* = 7.4 Hz, 1H), 8.56 (dd, *J* = 1.2, 7.7 Hz, 1H), 7.76 (d, *J* = 8.5 Hz, 1H), 7.69 (d, *J* = 7.5 Hz, 1H), 7.59 (d, *J* = 8.6 Hz, 1H), 7.43 (d, *J* = 8.5 Hz, 1H), 7.38 (t, *J* = 8.2 Hz, 1H), 7.33 – 7.26 (m, 2H), 7.11 (dq, *J* = 7.7, 15.4 Hz, 8H), 7.02 – 6.84 (m, 5H), 6.68 – 6.60 (m, 2H), 6.52 (t, *J* = 7.3 Hz, 1H), 6.47 (d, *J* = 8.5 Hz, 1H) ppm;

^{13}C NMR (100 MHz, $CDCl_3$) δ_C = 159.0, 150.9, 144.7, 141.1, 140.5, 138.5, 138.1, 135.3, 132.9, 131.2, 130.5, 130.2, 130.2, 129.1, 128.1, 127.9, 127.0, 126.9, 126.9, 126.6, 126.3, 126.3, 126.0, 125.8, 125.2, 125.0, 124.0, 116.6 ppm. The carbon attached to boron was not observed due to quadrupolar relaxation;

^{11}B NMR (128 MHz, $CDCl_3$) δ_B = 4.81 ppm.

IR (film): ν_{max} 3680, 3045, 2781, 1705, 1529, 1487, 1317, 1073, 891, 819, 703 cm^{-1}.

HRMS (ESI): m/z calculated for $C_{39}H_{28}BN_2O_2^+$ [M+H]$^+$, 567.2238, found, 567.2232.

HPLC analysis: HPLC conditions: Chiral column OD–H, *n*-hexane/isopropanol: 90/10, flow rate = 1.0 mL/min, wavelength = 254 nm, t_R = 12.421 min for major isomer, t_R = 6.602 min for minor isomer, 98% ee.

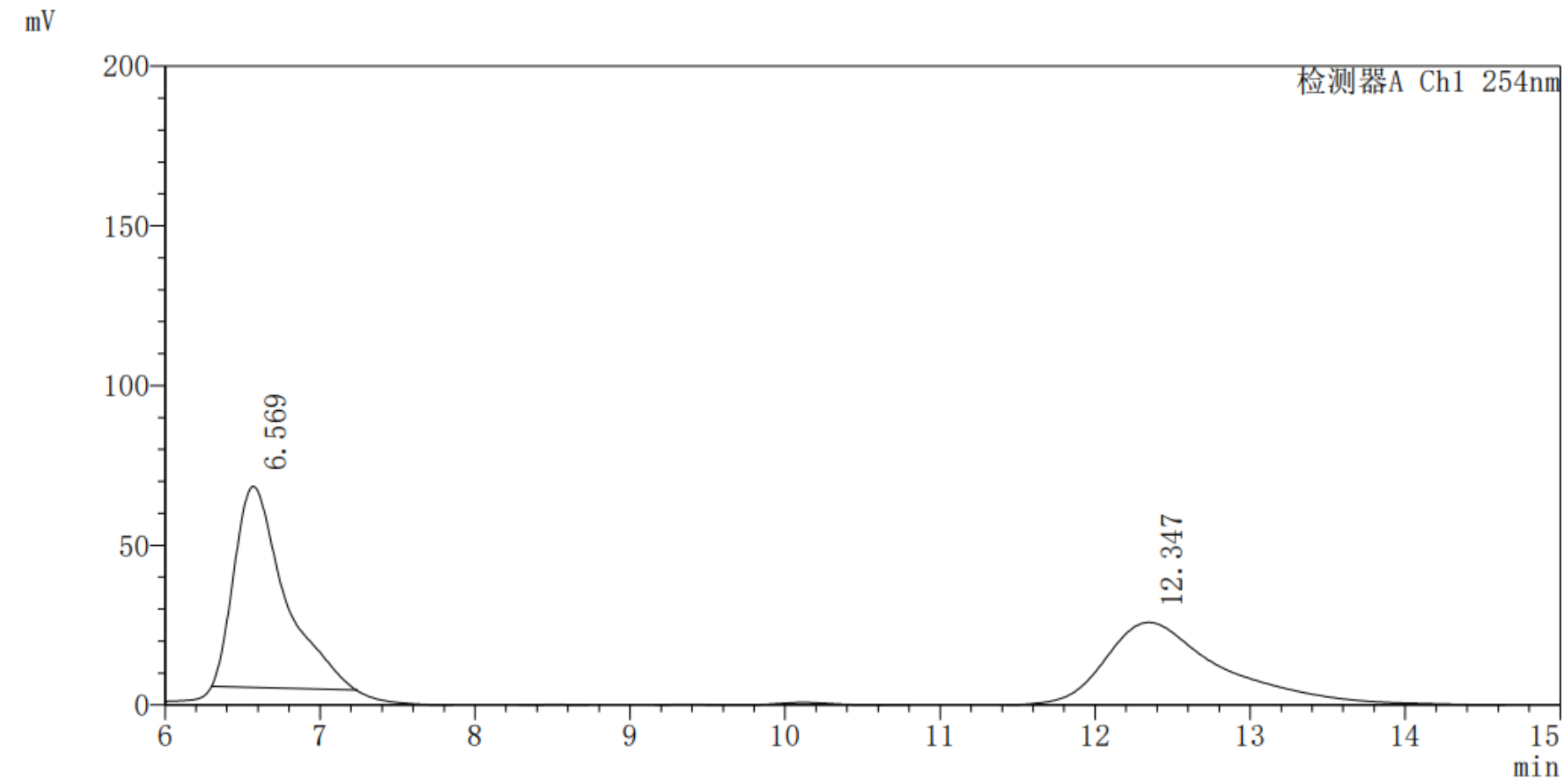

检测器A Ch1 254nm

No.	R.Time	Height	Height%	Area	Area%
1	6.569	62945	70.723	1430519	50.437
2	12.347	26057	29.277	1405757	49.563

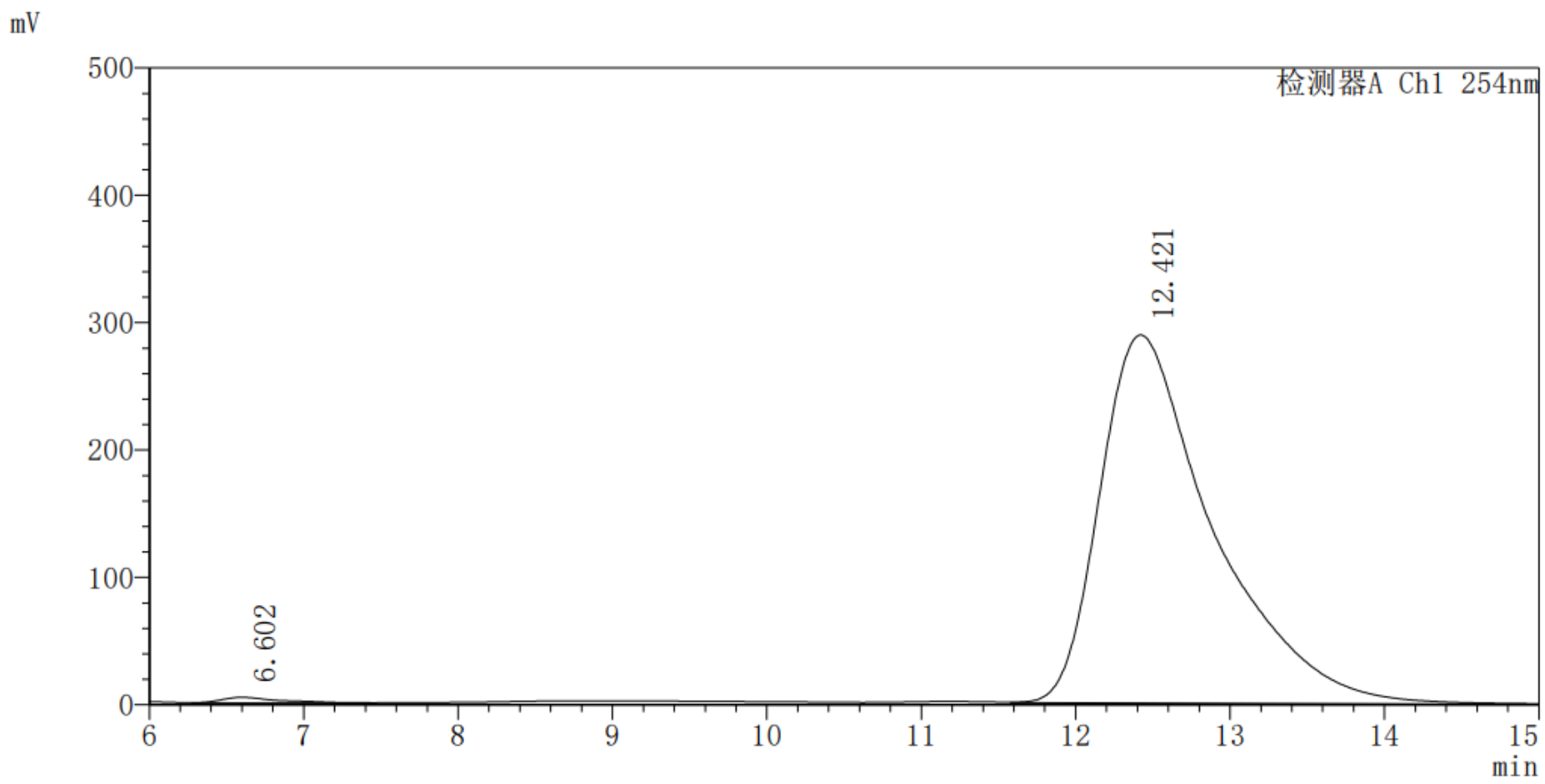

检测器A Ch1 254nm

No.	R.Time	Height	Height%	Area	Area%
1	6.602	4576	1.559	121215	0.816
2	12.421	288946	98.441	14733125	99.184

(*R*)-*N*,*N*-Diphenyl-4-(4,4,5-triphenyl-4*H*-3l4,4l4-naphtho[2',1':5,6][1,2]azaborepino[7,1-a] isoquinolin-16-yl)aniline (10)

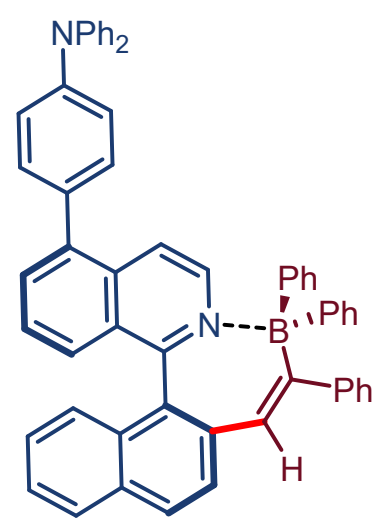

10

Prepared following **Procedure C**, using 1-{5-[4-(diphenylamino)phenyl]isoquinolin-1-yl}naphthalen-2-yl trifluoromethanesulfonate (129.3 mg, 0.2 mmol, 1.0 equiv.), tetramethylammonium ethynyltriphenylborate (102.3 mg, 0.3 mmol, 1.5 equiv.), $Pd(acac)_2$ (1.5 mg, 2.5 mol%), and (*R*)-**L4** (11.0 mg, 7.5 mol%). Purification by flash column chromatography (Petroleum ether/EtOAc: 50/1) to afford the title compound (146.8 mg, 96%) as a yellow solid.

TLC: R_f = 0.5 (Petroleum ether/EtOAc: 50/1, $KMnO_4$ stain).

M. p.: 188 – 189 °C.

$[\alpha]^{18}_D$: +95.02 (c 0.20, CH_2Cl_2).

NMR Spectroscopy (*see spectra*):

> **^{1}H NMR** (400 MHz, $CDCl_3$) δ_H = δ 8.79 (d, *J* = 7.1 Hz, 1H), 8.06 (d, *J* = 7.3 Hz, 1H), δ 7.74 (d, *J* = 2.6 Hz, 1H), 7.72 (d, *J* = 3.8 Hz, 1H), 7.69 (d, *J* = 7.9 Hz, 1H), 7.45 – 7.34 (m, 8H), 7.32 – 7.25 (m, 8H), 7.24 – 7.02 (m, 12H), 7.02 – 6.95 (m, 4H), 6.74 – 6.50 (m, 4H) ppm;
>
> **^{13}C NMR** (100 MHz, $CDCl_3$) δ_C = 158.1, 151.3, 148.1, 147.5, 140.0, 139.1, 138.7, 138.1, 135.5, 134.9, 133.0, 132.8, 131.5, 130.8, 130.5, 130.3, 129.6, 129.4, 128.2, 128.1, 127.6, 127.5, 127.4, 126.8, 126.4, 126.1, 125.7, 125.3, 125.0, 124.8, 124.8, 123.6, 123.6, 122.9, 119.6 ppm. The carbon attached to boron was not observed due to quadrupolar relaxation;
>
> **^{11}B NMR** (128 MHz, $CDCl_3$) δ_B = 3.07 ppm.

IR (film): ν_{max} 3661, 2998, 2779, 1648, 1593, 1490, 1316, 1273, 1029, 882, 816, 697 cm^{-1}.

HRMS (ESI): m/z calculated for $C_{57}H_{42}BN_2^+$ $[M+H]^+$, 765.3436, found, 765.3440.

HPLC analysis: HPLC conditions: Chiral column IB, *n*-hexane/isopropanol: 93/7, flow rate = 1.0 mL/min, wavelength = 254 nm, t_R = 5.105 min for major isomer, t_R = 4.796 min for minor isomer, 94% ee.

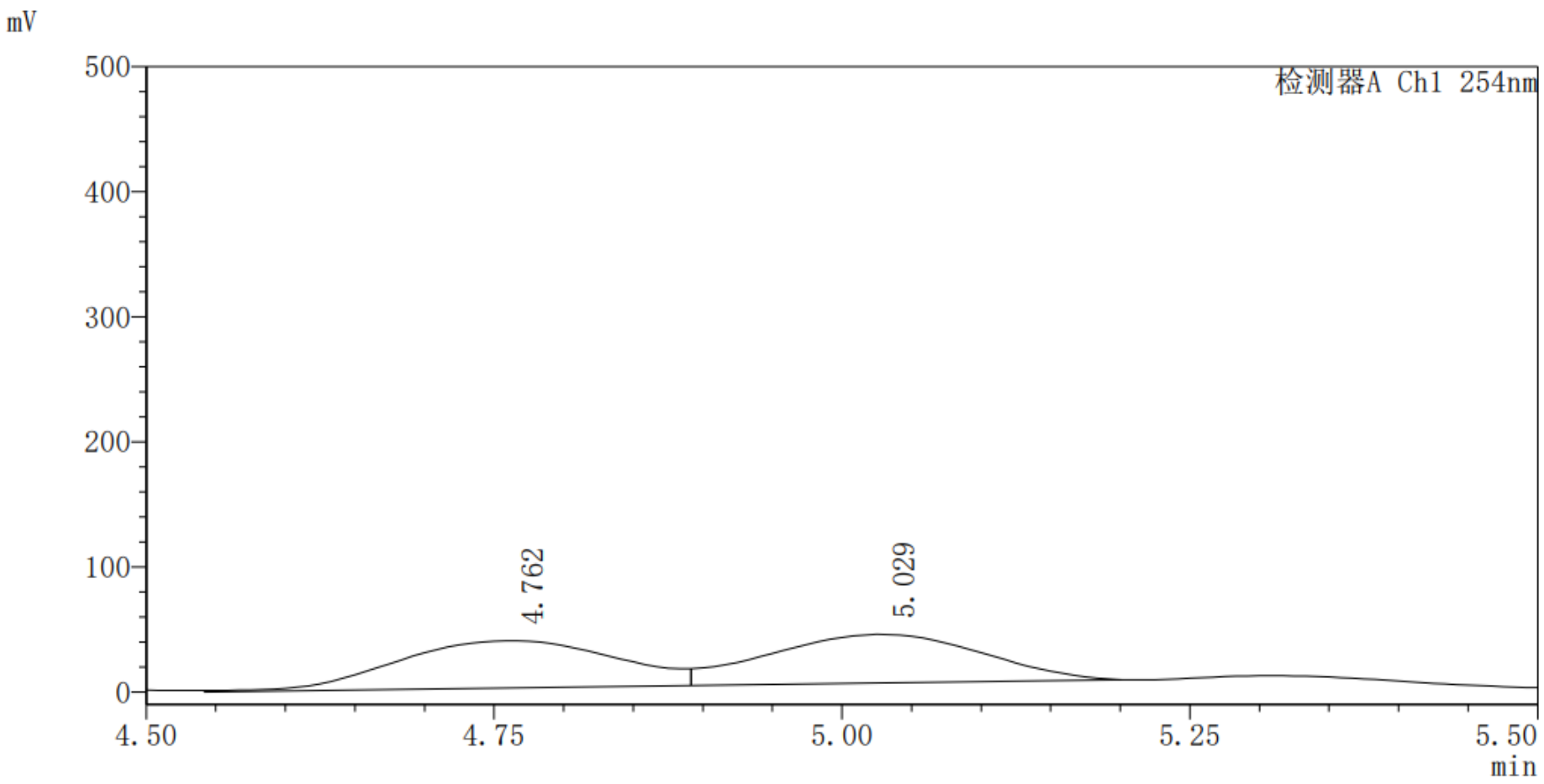

检测器A Ch1 254nm

No.	R.Time	Height	Height%	Area	Area%
1	4.762	37775	49.393	419432	50.337
2	5.029	38704	50.607	413817	49.663

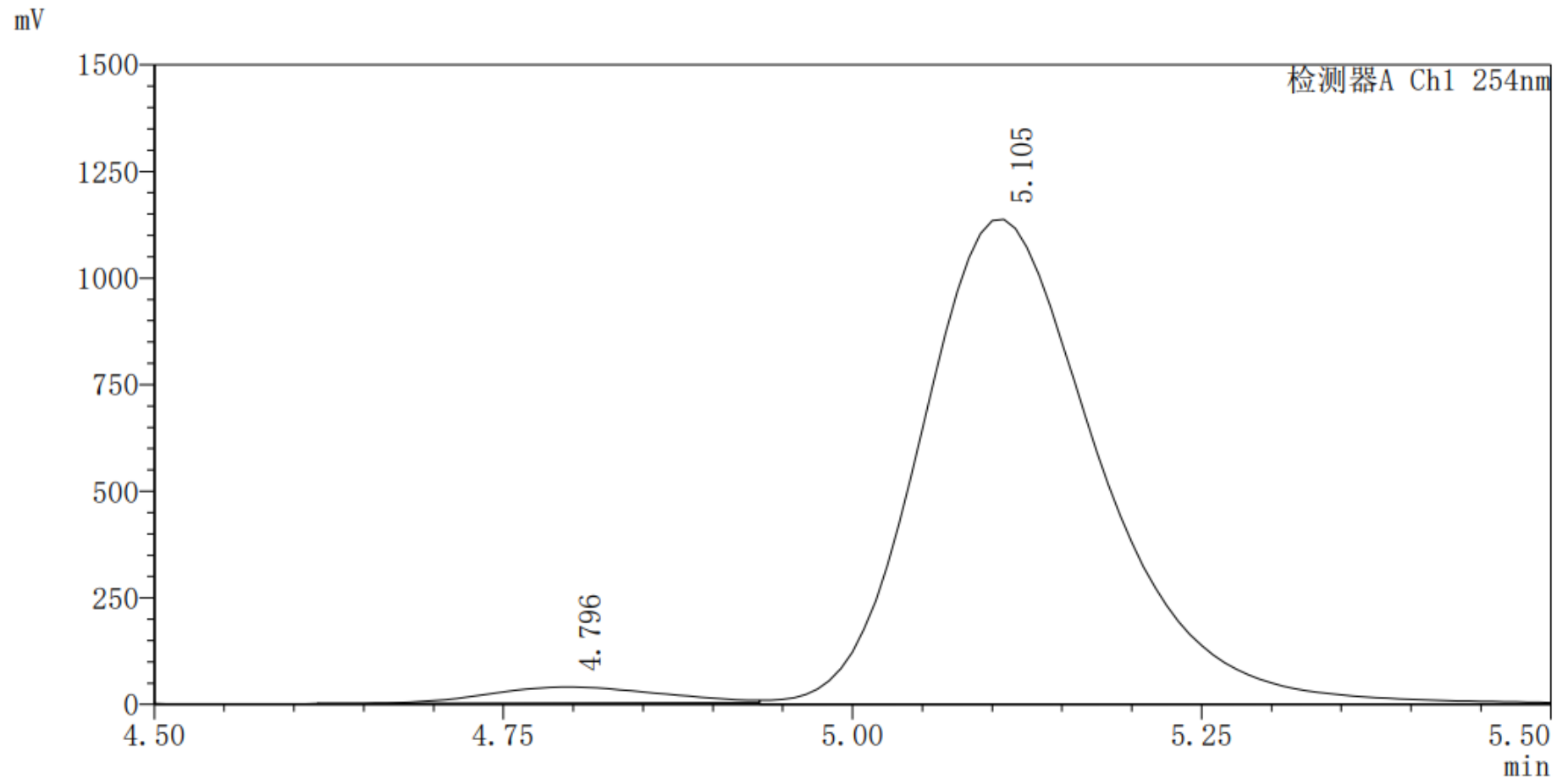

检测器A Ch1 254nm

No.	R.Time	Height	Height%	Area	Area%
1	4.796	37201	3.169	322619	3.086
2	5.105	1136737	96.831	10130169	96.914

(*R*)-12,14-Dimethyl-8,8,9-triphenyl-8*H*-7l4,8l4-benzo[5,6][1,2]azaborepino[7,1-a]isoquinoline (11)

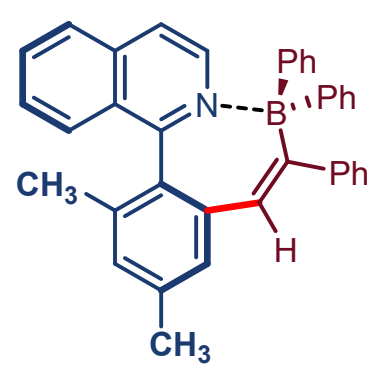

11

Prepared following **Procedure C**, using 2-(isoquinolin-1-yl)-3,5-dimethylphenyl trifluoromethanesulfonate (76.2 mg, 0.2 mmol, 1.0 equiv.), tetramethylammonium

ethynyltriphenylborate (102.3 mg, 0.3 mmol, 1.5 equiv.), $Pd(acac)_2$ (1.5 mg, 2.5 mol%), and (*R*)-**L4** (11.0 mg, 7.5 mol%). Purification by flash column chromatography (Petroleum ether/EtOAc: 50/1) to afford the title compound (93.9 mg, 94%) as a yellow solid.

TLC: R_f = 0.5 (Petroleum ether/EtOAc: 50/1, $KMnO_4$ stain).

M. p.: 214 – 215 °C.

$[\alpha]^{18}_D$: +270.50 (c 0.20, CH_2Cl_2).

NMR Spectroscopy (*see spectra*):

^{1}H NMR (400 MHz, $CDCl_3$) δ_H = 8.74 (d, *J* = 6.9 Hz, 1H), 7.92 (d, *J* = 8.1 Hz, 1H), 7.81 (ddd, *J* = 3.0, 4.9, 8.1 Hz, 1H), 7.67 (d, *J* = 7.0 Hz, 1H), 7.56 (d, *J* = 2.5 Hz, 2H), 7.14 – 6.89 (m, 15H), 6.83 (s, 2H), 6.54 (s, 1H), 2.32 (s, 3H), 1.40 (s, 3H) ppm;

^{13}C NMR (100 MHz, $CDCl_3$) δ_C = 159.7, 151.8, 141.0, 138.7, 138.6, 138.0, 137.4, 136.3, 135.7, 132.7, 130.2, 129.7, 129.0, 128.2, 128.1, 127.7, 126.7, 126.5, 126.3, 125.6, 125.2, 124.3, 120.5, 21.5, 21.3 ppm. The carbon attached to boron was not observed due to quadrupolar relaxation;

^{11}B NMR (128 MHz, $CDCl_3$) δ_B = 3.24 ppm.

IR (film): ν_{max} 3707, 2998, 2779, 2684, 1666, 1644, 1623, 1503, 1353, 765, 703 cm^{-1}.

HRMS (ESI): m/z calculated for $C_{37}H_{31}BN^+$ $[M+H]^+$, 500.2544, found, 500.2535.

HPLC analysis: HPLC conditions: Chiral column IG, *n*-hexane/isopropanol: 99/1, flow rate = 1.0 mL/min, wavelength = 254 nm, t_R = 8.787 min for major isomer, t_R = 5.484 min for minor isomer, 95% ee.

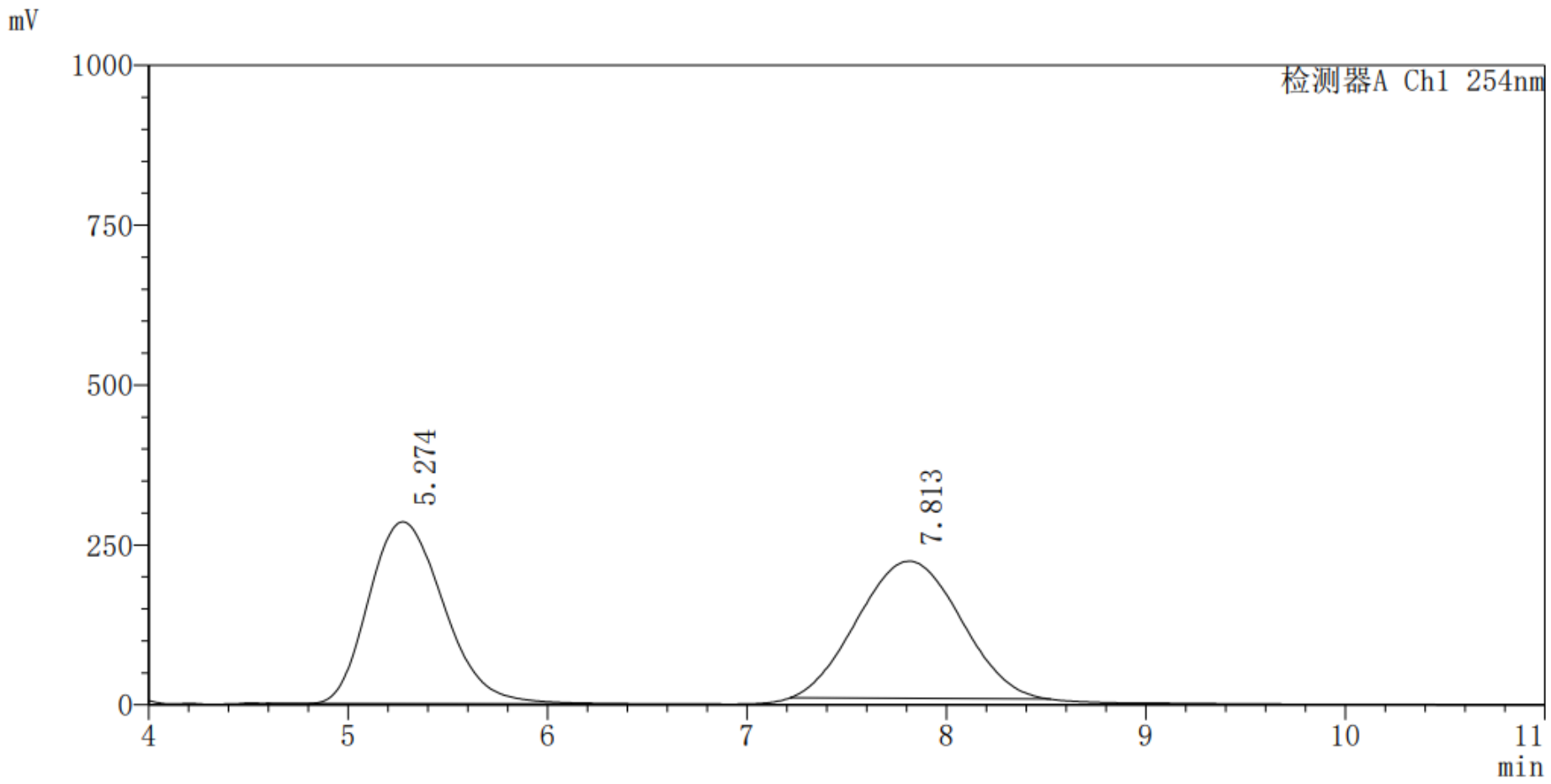

检测器A Ch1 254nm

No.	R.Time	Height	Height%	Area	Area%
1	5.274	284568	57.017	7447971	49.121
2	7.813	214526	42.983	7714373	50.879

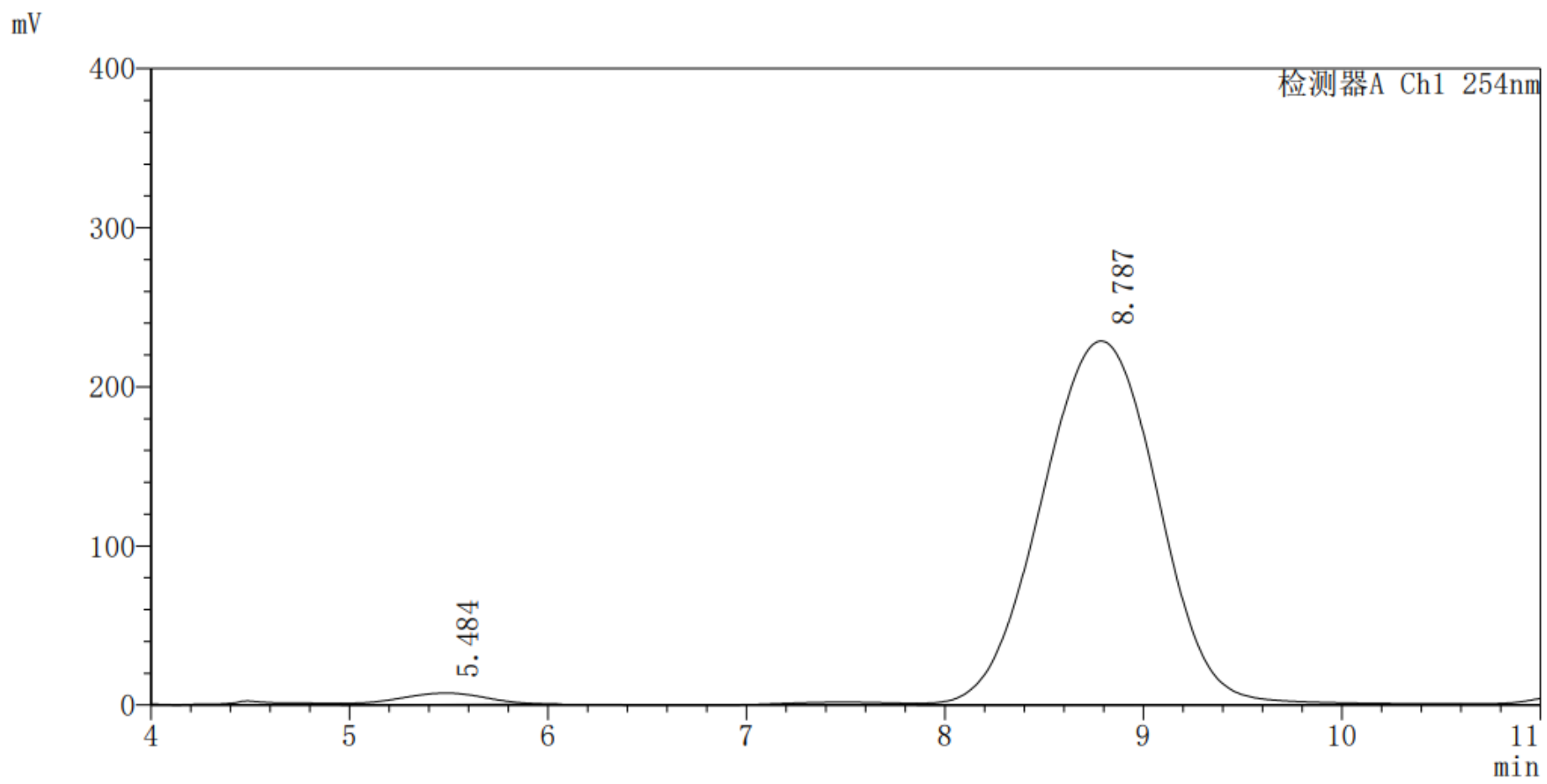

检测器A Ch1 254nm

No.	R.Time	Height	Height%	Area	Area%
1	5.484	7323	3.104	227451	2.379
2	8.787	228560	96.896	9332940	97.621

(*R*)-11-Methoxy-4,4,5-triphenyl-4*H*-3l4,4l4-naphtho[2',1':5,6][1,2]azaborepino[7,1-a]isoquinoline (12)

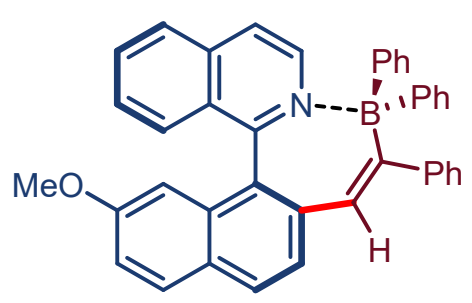

12

Prepared following **Procedure C**, using 1-(isoquinolin-1-yl)-7-methoxynaphthalen-2-yl trifluoromethanesulfonate (86.6 mg, 0.2 mmol, 1.0 equiv.), tetramethylammonium

ethynyltriphenylborate (102.3 mg, 0.3 mmol, 1.5 equiv.), Pd(acac)$_2$ (1.5 mg, 2.5 mol%), and (*R*)-**L4** (11.0 mg, 7.5 mol%). Purification by flash column chromatography (Petroleum ether/EtOAc: 50/1) to afford the title compound (105.8 mg, 96%) as a yellow solid.

TLC: $R_f = 0.5$ (Petroleum ether/EtOAc: 50/1, $KMnO_4$ stain).

M. p.: 160 – 161 °C.

$[\alpha]^{18}_D$: +273.46 (c 0.20, CH_2Cl_2).

NMR Spectroscopy (*see spectra*):

^{1}H NMR (400 MHz, $CDCl_3$) δ_H = 8.81 (d, *J* = 7.0 Hz, 1H), 7.94 (d, *J* = 8.1 Hz, 1H), 7.75 (t, *J* = 7.8 Hz, 2H), 7.62 (d, *J* = 8.4 Hz, 1H), 7.55 (d, *J* = 8.9 Hz, 1H), 7.34 (t, *J* = 7.8 Hz, 1H), 7.24 (s, 2H), 7.17 (s, 1H), 7.15 – 7.01 (m, 7H), 7.00 – 6.78 (m, 6H), 6.71 – 6.57 (m, 2H), 6.52 (t, *J* = 7.2 Hz, 1H), 5.76 (s, 1H), 3.35 (s, 3H) ppm;

^{13}C NMR (100 MHz, $CDCl_3$) δ_C = 158.3, 157.2, 151.4, 140.4, 138.7, 138.2, 136.2, 135.7, 134.1, 132.7, 131.3, 129.9, 129.2, 129.1, 128.6, 128.1, 126.8, 126.4, 126.4, 126.1, 125.3, 124.7, 124.0, 123.7, 121.2, 116.7, 107.2, 55.0 ppm. The carbon attached to boron was not observed due to quadrupolar relaxation;

^{11}B NMR (160 MHz, $CDCl_3$) δ_B = 3.38 ppm.

IR (film): ν_{max} 3670, 2816, 2779, 1643, 1621, 1509, 1455, 1220, 833, 762, 707 cm^{-1}.

HRMS (ESI): m/z calculated for $C_{40}H_{31}BNO^+$ $[M+H]^+$, 552.2493, found, 552.2497.

HPLC analysis: HPLC conditions: Chiral column AD–H, *n*-hexane/isopropanol: 99/1, flow rate = 1.0 mL/min, wavelength = 254 nm, t_R = 8.636 min for major isomer, t_R = 11.482 min for minor isomer, 98% ee.

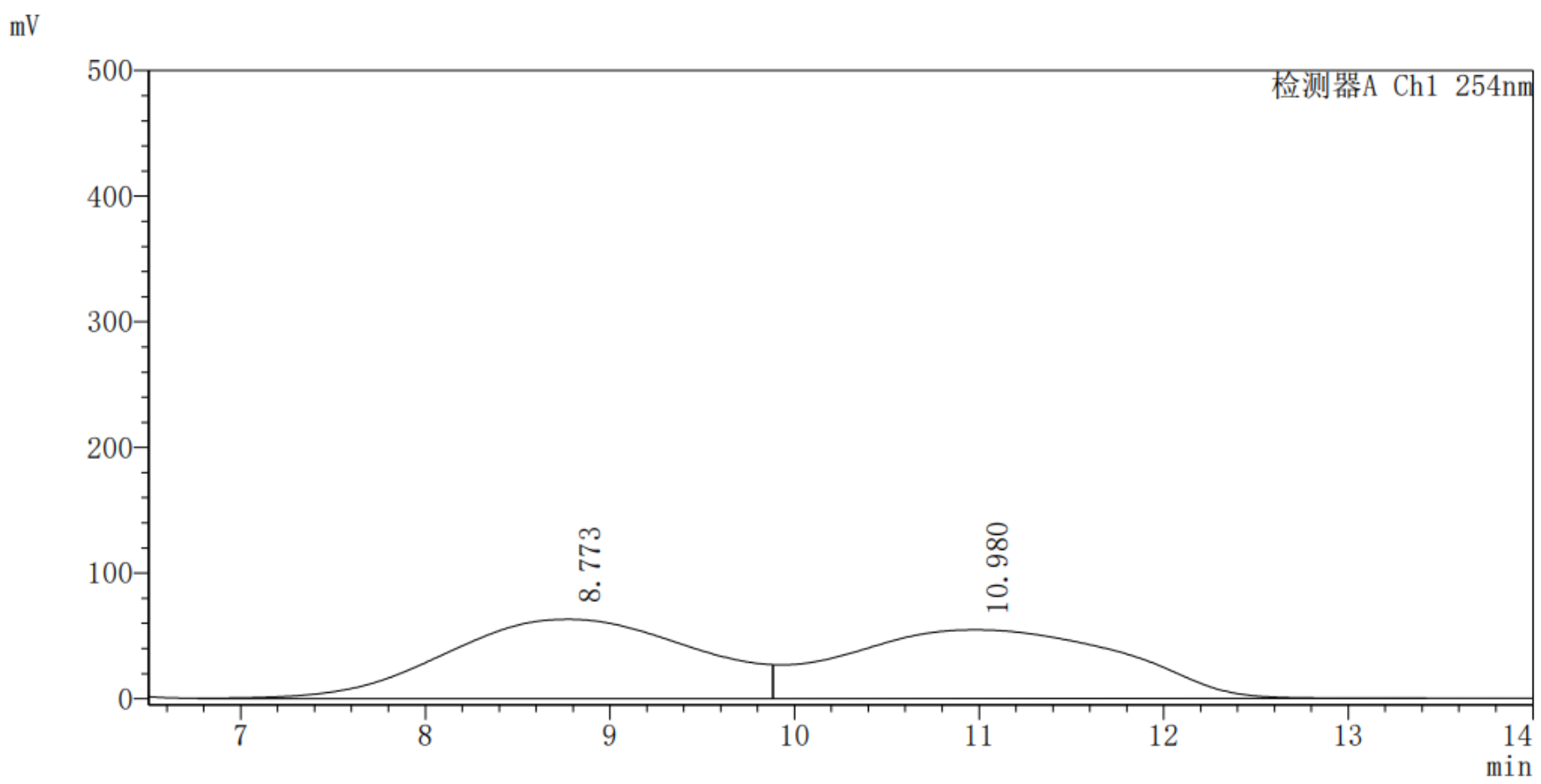

检测器A Ch1 254nm

No.	R.Time	Height	Height%	Area	Area%
1	8.773	63076	53.590	5922815	50.733
2	10.980	54624	46.410	5751719	49.267

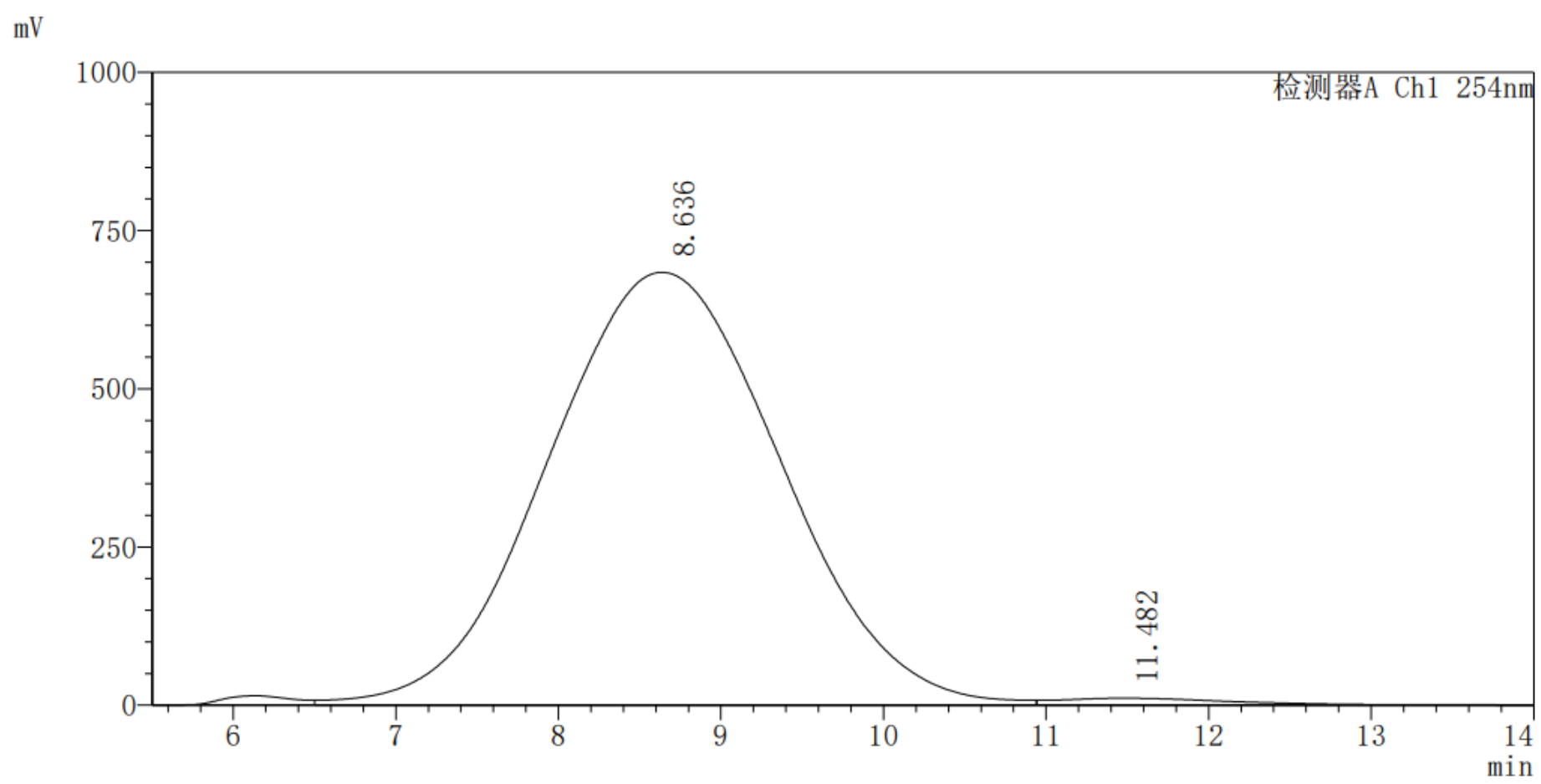

检测器A Ch1 254nm

No.	R.Time	Height	Height%	Area	Area%
1	8.636	683672	98.452	68051077	98.823
2	11.482	10751	1.548	810556	1.177

(*R*)-4,4,5,10-Tetraphenyl-4*H*-3l4,4l4-naphtho[2',1':5,6][1,2]azaborepino[7,1-a]isoquinoline (13)

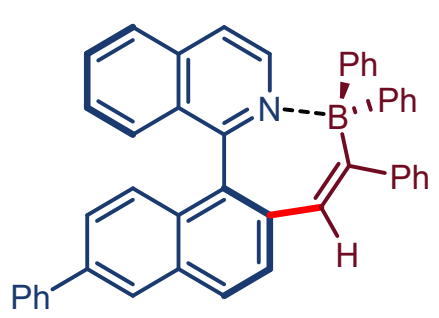

13

Prepared following **Procedure C**, using 1-(isoquinolin-1-yl)-6-phenylnaphthalen-2-yl trifluoromethanesulfonate (95.8 mg, 0.2 mmol, 1.0 equiv.), tetramethylammonium ethynyltriphenylborate (102.3 mg, 0.3 mmol, 1.5 equiv.), $Pd(acac)_2$ (1.5 mg, 2.5 mol%), and (*R*)-**L4** (11.0

mg, 7.5 mol%). Purification by flash column chromatography (Petroleum ether/EtOAc: 50/1) to afford the title compound (108.7 mg, 91%) as a yellow solid.

TLC: R_f = 0.5 (Petroleum ether/EtOAc: 50/1, $KMnO_4$ stain).

M. p.: 182 – 183 °C.

$[\alpha]^{18}_D$: +433.00 (c 0.20, CH_2Cl_2).

NMR Spectroscopy (*see spectra*):

^{1}H NMR (400 MHz, $CDCl_3$) δ_H = 8.76 (d, *J* = 6.9 Hz, 1H), 7.85 (d, *J* = 8.3 Hz, 1H), 7.80 (s, 1H), 7.67 (dd, *J* = 5.3, 7.8 Hz, 3H), 7.60 (d, *J* = 7.8 Hz, 2H), 7.39 (t, *J* = 7.7 Hz, 2H), 7.31 (dd, *J* = 4.3, 8.0 Hz, 3H), 7.23 – 7.11 (m, 3H), 7.10 – 6.95 (t, *J* = 8.6 Hz, 7H), 6.95 – 6.73 (m, 5H), 6.66 – 6.39 (m, 4H) ppm;

^{13}C NMR (100 MHz, $CDCl_3$) δ_C = 158.0, 151.3, 140.4, 140.0, 138.7, 138.2, 137.1, 136.3, 135.5, 132.8, 132.1, 131.2, 130.8, 130.2, 129.7, 128.9, 128.6, 128.2, 127.8, 127.5, 127.2, 127.1, 126.8, 126.6, 126.5, 126.4, 125.4, 125.3, 125.1, 124.8, 123.8, 121.3 ppm. The carbon attached to boron was not observed due to quadrupolar relaxation;

^{11}B NMR (128 MHz, $CDCl_3$) δ_B = 4.18 ppm.

IR (film): ν_{max} 3692, 3043, 2997, 2778, 1752, 1448, 1168, 890, 822, 743, 701 cm^{-1}.

HRMS (ESI): m/z calculated for $C_{45}H_{33}BN^+$ $[M+H]^+$, 598.2701, found, 598.2692.

HPLC analysis: HPLC conditions: Chiral column AD–H, *n*-hexane/isopropanol: 99/1, flow rate = 1.0 mL/min, wavelength = 254 nm, t_R = 9.162 min for major isomer, t_R = 14.394 min for minor isomer, 98% ee.

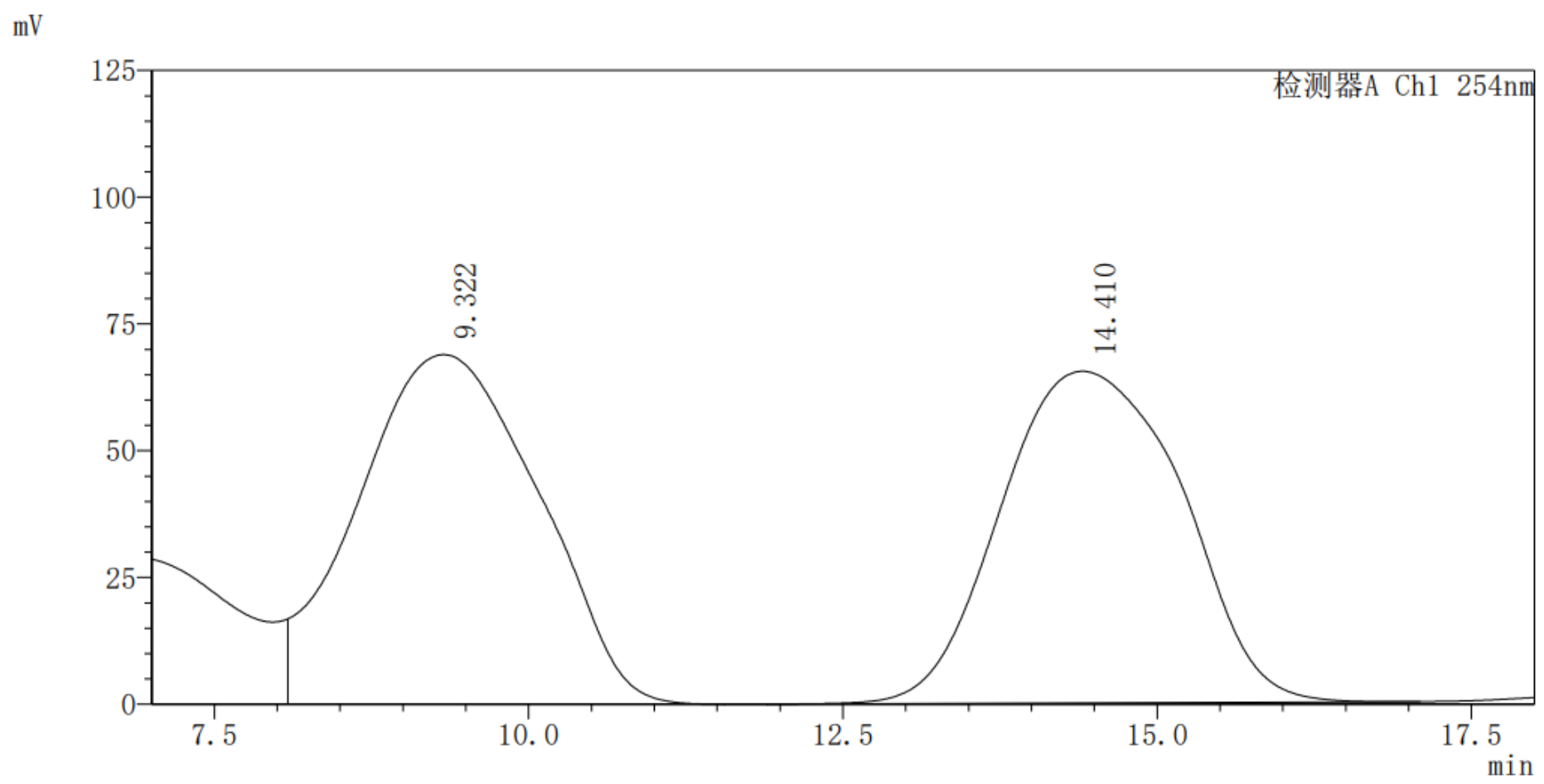

检测器A Ch1 254nm

No.	R.Time	Height	Height%	Area	Area%
1	9.322	69103	51.363	6913607	51.225
2	14.410	65436	48.637	6582866	48.775

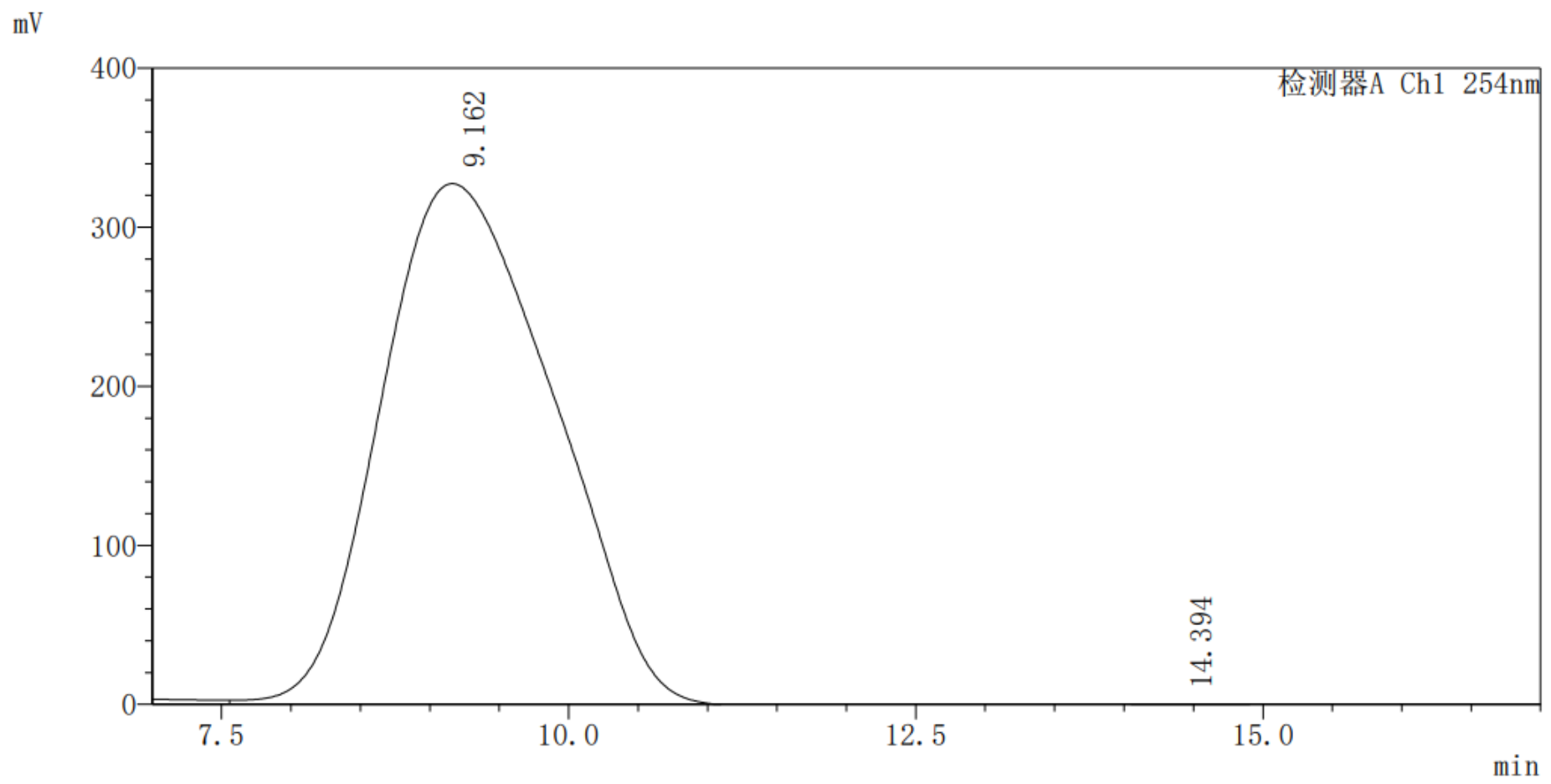

检测器A Ch1 254nm

No.	R.Time	Height	Height%	Area	Area%
1	9.162	329209	99.213	28587530	99.180
2	14.394	2613	0.787	236287	0.820

(*R*)-4,4,5,8-Tetraphenyl-4*H*-3l4,4l4-naphtho[2',1':5,6][1,2]azaborepino[7,1-a]isoquinoline (14)

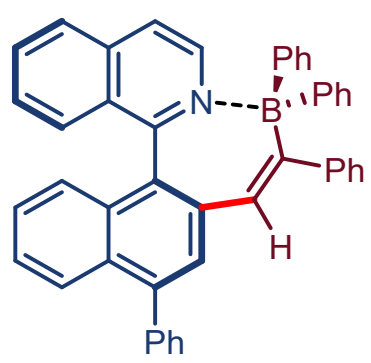

14

Prepared following **Procedure C**, using 1-(isoquinolin-1-yl)-4-phenylnaphthalen-2-yl trifluoromethanesulfonate (95.8 mg, 0.2 mmol, 1.0 equiv.), tetramethylammonium

ethynyltriphenylborate (102.3 mg, 0.3 mmol, 1.5 equiv.), Pd(acac)$_2$ (1.5 mg, 2.5 mol%), and (*R*)-**L4** (11.0 mg, 7.5 mol%). Purification by flash column chromatography (Petroleum ether/EtOAc: 50/1) to afford the title compound (115.9 mg, 97%) as a yellow solid.

TLC: R_f = 0.5 (Petroleum ether/EtOAc: 50/1, $KMnO_4$ stain).

M. p.: 191 – 192 °C.

$[\alpha]^{18}_D$: +253.93 (c 0.20, CH_2Cl_2).

NMR Spectroscopy (*see spectra*):

^{1}H NMR (400 MHz, $CDCl_3$) δ_H = 8.90 (d, *J* = 6.9 Hz, 1H), 7.98 (d, *J* = 8.1 Hz, 1H), 7.79 (t, *J* = 7.7 Hz, 2H), 7.71 (d, *J* = 8.5 Hz, 1H), 7.60 – 7.49 (m, 5H), 7.45 (d, *J* = 7.9 Hz, 1H), 7.39 (d, *J* = 6.8 Hz, 1H), 7.36 (s, 1H), 7.24 – 7.17 (m, 2H), 7.18 – 7.02 (m, 10H), 7.03 – 6.86 (m, 3H), 6.82 – 6.44 (m, 4H) ppm;

^{13}C NMR (100 MHz, $CDCl_3$) δ_C = 158.1, 151.2, 141.5, 140.4, 139.7, 138.8, 138.2, 136.3, 135.3, 133.2, 132.8, 131.3, 130.4, 130.0, 129.0, 128.6, 128.4, 128.1, 127.6, 126.9, 126.8, 126.8, 126.5, 126.4, 125.9, 125.5, 125.4, 124.8, 124.7, 123.6, 121.3 ppm. The carbon attached to boron was not observed due to quadrupolar relaxation;

^{11}B NMR (128 MHz, $CDCl_3$) δ_B = 4.78 ppm.

IR (film): ν_{max} 3678, 2815, 2779, 2708, 2683, 1647, 1624, 1370, 1353, 765, 702 cm^{-1}.

HRMS (ESI): m/z calculated for $C_{45}H_{33}BN^+$ $[M+H]^+$, 598.2701, found, 598.2711.

HPLC analysis: HPLC conditions: Chiral column AD–H, *n*-hexane/isopropanol: 99/1, flow rate = 1.0 mL/min, wavelength = 254 nm, t_R = 7.720 min for major isomer, t_R = 10.320 min for minor isomer, 99% ee.

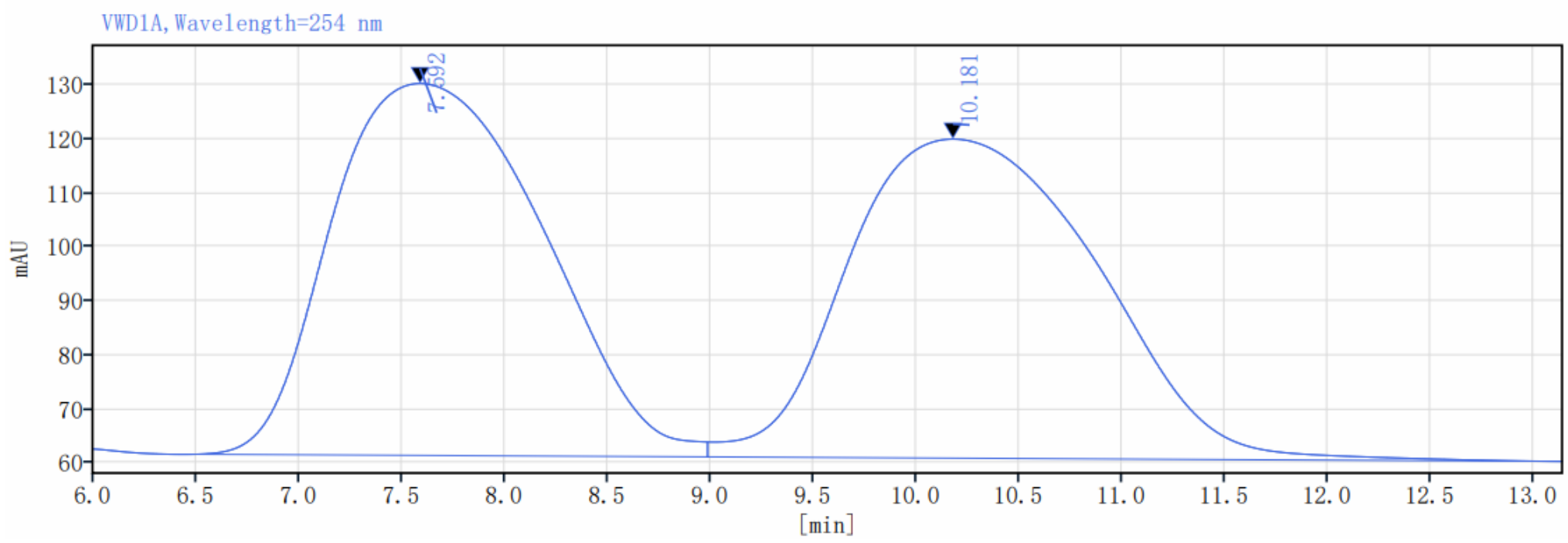

信号: VWD1A, Wavelength=254 nm

Retention Time [min]	Int Type	Width [min]	Area	Height	Area%
7.592	BM m	2.49	4914.17	68.81	49.71
10.181	MM m	4.18	4970.83	59.03	50.29

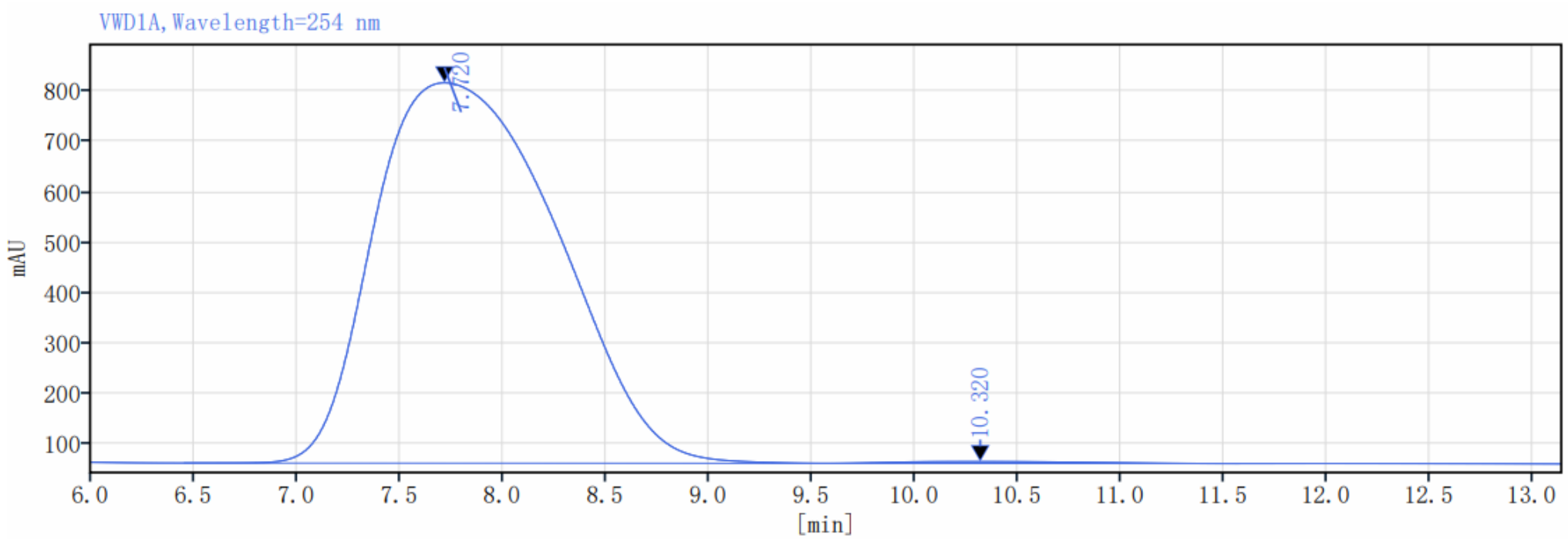

信号: VWD1A, Wavelength=254 nm

Retention Time [min]	Int Type	Width [min]	Area	Height	Area%
7.720	BM m	3.11	46497.56	754.02	99.52
10.320	MM m	2.02	224.04	3.53	0.48

(*R*)-4,4,5-Triphenyl-4*H*-3l4,4l4-naphtho[2',1':5,6][1,2]azaborepino[7,1-a]isoquinoline-10-carbaldehyde (15)

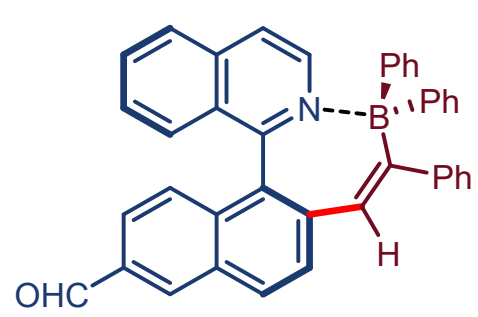

15

Prepared following **Procedure C**, using 6-formyl-1-(isoquinolin-1-yl)naphthalen-2-yl trifluoromethanesulfonate (86.2 mg, 0.2 mmol, 1.0 equiv.), tetramethylammonium ethynyltriphenylborate (102.3 mg, 0.3 mmol, 1.5 equiv.), $Pd(acac)_2$ (1.5 mg, 2.5 mol%), and (*R*)-**L4** (11.0 mg, 7.5 mol%). Purification by flash column chromatography (Petroleum ether/EtOAc: 50/1) to afford

the title compound (106.5 mg, 97%) as a yellow solid.

TLC: R_f = 0.5 (Petroleum ether/EtOAc: 50/1, $KMnO_4$ stain).

M. p.: 195 – 196 °C.

$[\alpha]^{18}_D$: +569.93 (c 0.20, CH_2Cl_2).

NMR Spectroscopy (*see spectra*):

^{1}H NMR (400 MHz, $CDCl_3$) δ_H = 10.04 (s, 1H), 8.85 (d, J = 6.9 Hz, 1H), 8.13 (s, 1H), 7.98 (d, J = 8.2 Hz, 1H), 7.83 (d, J = 7.8 Hz, 2H), 7.78 (t, J = 7.6 Hz, 1H), 7.49 (t, J = 9.8 Hz, 2H), 7.33 (t, J = 7.7 Hz, 1H), 7.22 (s, 1H), 7.17 – 7.00 (m, 8H), 6.99 – 6.73 (m, 5H), 6.69 – 6.42 (m, 4H) ppm;

^{13}C NMR (100 MHz, $CDCl_3$) δ_C = 191.9, 157.2, 151.1, 142.7, 138.6, 138.3, 136.5, 136.1, 135.2, 133.9, 133.0, 132.8, 130.6, 130.1, 130.1, 129.7, 128.9, 128.2, 128.1, 127.5, 127.5, 126.9, 126.7, 126.5, 125.5, 125.0, 123.9, 122.7, 121.7 ppm. The carbon attached to boron was not observed due to quadrupolar relaxation;

^{11}B NMR (128 MHz, $CDCl_3$) δ_B = 4.01 ppm.

IR (film): ν_{max} 3692, 3043, 2997, 2815, 2779, 1695, 1621, 1487, 1318, 1162, 1135, 822, 744, 703 cm^{-1}.

HRMS (ESI): m/z calculated for $C_{40}H_{29}BNO^+$ $[M+H]^+$, 550.2337, found, 550.2345.

HPLC analysis: HPLC conditions: Chiral column AD–H, *n*-hexane/isopropanol: 98/2, flow rate = 1.0 mL/min, wavelength = 254 nm, t_R = 14.998 min for major isomer, t_R = 17.894 min for minor isomer, 96% ee.

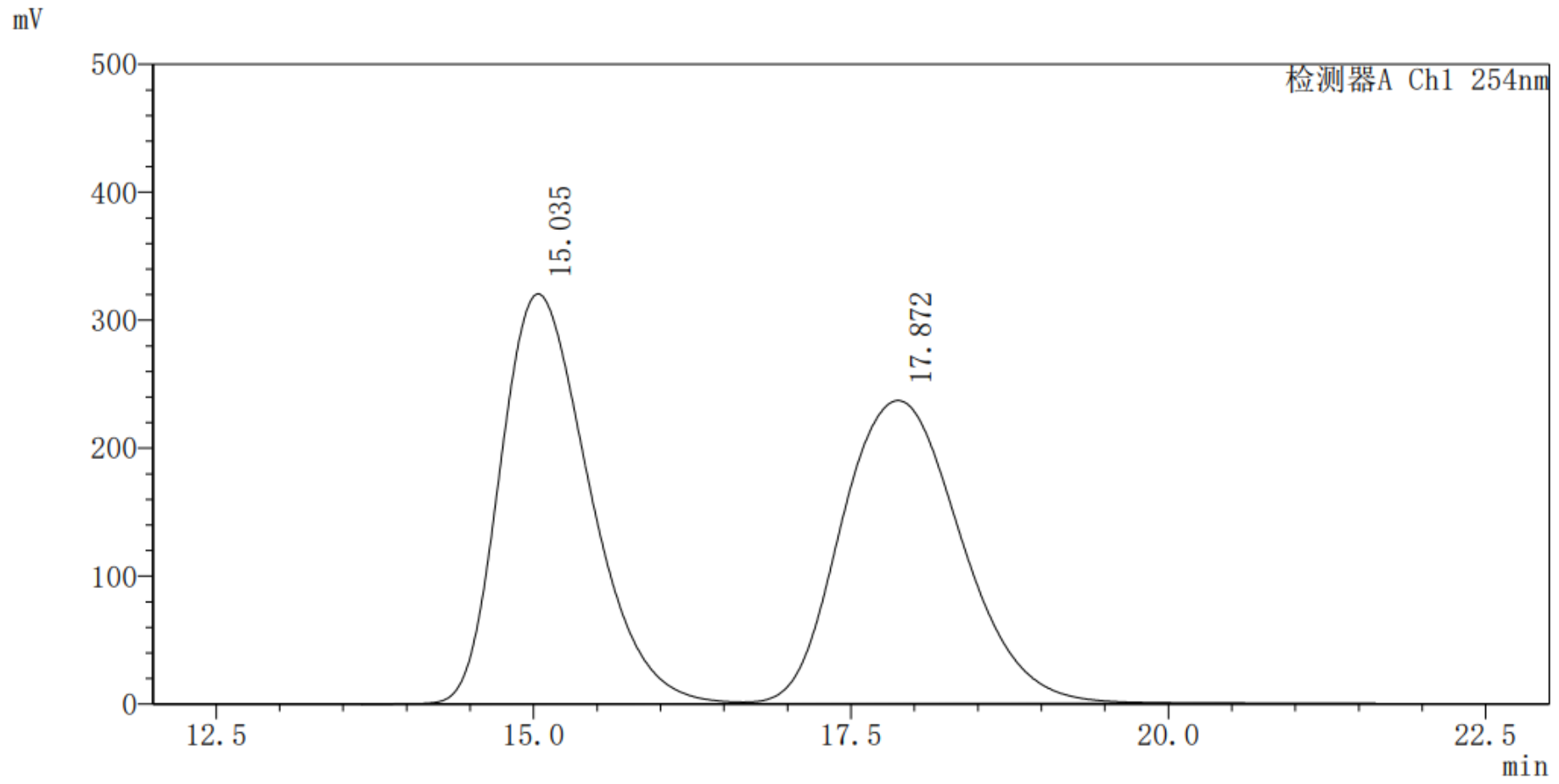

检测器A Ch1 254nm

No.	R.Time	Height	Height%	Area	Area%
1	15.035	320461	57.503	15641403	50.217
2	17.872	236831	42.497	15506339	49.783

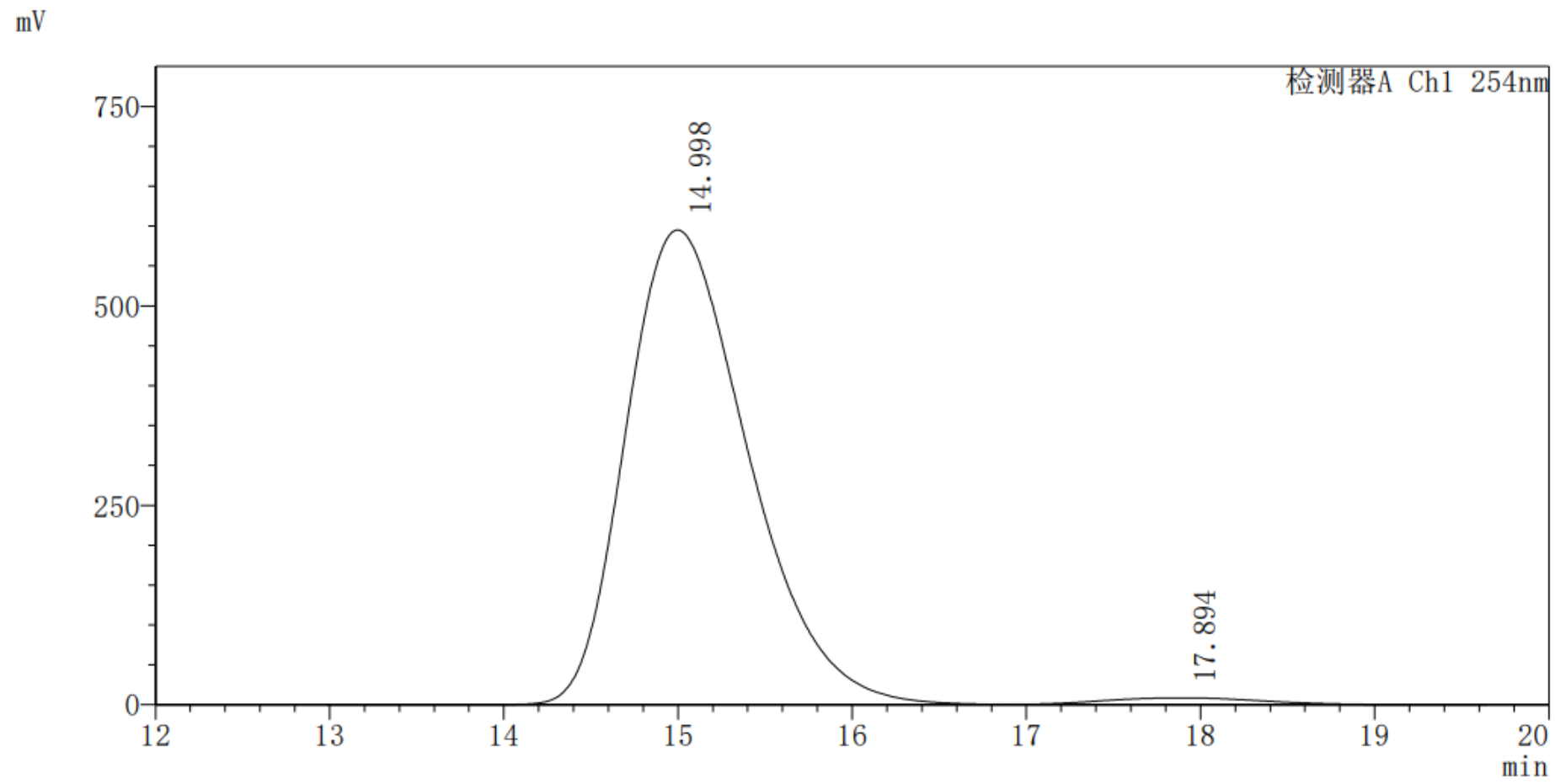

检测器A Ch1 254nm

No.	R.Time	Height	Height%	Area	Area%
1	14.998	595374	98.524	29089322	98.037
2	17.894	8921	1.476	582308	1.963

(*R*)-Methyl 4,4,5-triphenyl-4*H*-3l4,4l4-naphtho[2',1':5,6][1,2]azaborepino[7,1-a]isoquinoline-10-carboxylate (16)

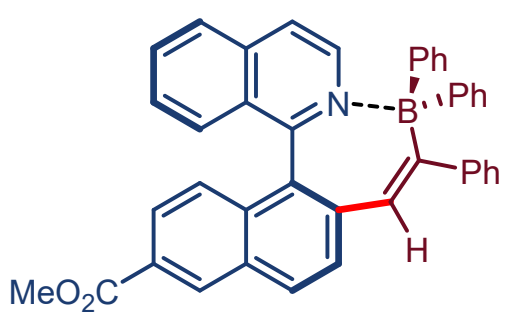

16

Prepared following **Procedure C,** using methyl 5-(isoquinolin-1-yl)-6-{[(trifluoromethyl)sulfonyl]oxy}-2-naphthoate (92.2 mg, 0.2 mmol, 1.0 equiv.), tetramethylammonium

ethynyltriphenylborate (102.3 mg, 0.3 mmol, 1.5 equiv.), $Pd(acac)_2$ (1.5 mg, 2.5 mol%), and (*R*)-**L4** (11.0 mg, 7.5 mol%). Purification by flash column chromatography (Petroleum ether/EtOAc: 50/1) to afford the title compound (112.4 mg, 97%) as a yellow solid.

TLC: R_f = 0.5 (Petroleum ether/EtOAc: 50/1, $KMnO_4$ stain).

M. p.: 182 – 183 °C.

$[\alpha]^{18}_D$: +353.92 (c 0.20, CH_2Cl_2).

NMR Spectroscopy (*see spectra*):

^{1}H NMR (400 MHz, $CDCl_3$) δ_H = 8.83 (d, *J* = 6.9 Hz, 1H), 8.41 (s, 1H), 7.96 (d, *J* = 8.3 Hz, 1H), 7.85 – 7.71 (m, 3H), 7.61 (d, *J* = 8.6 Hz, 1H), 7.43 (d, *J* = 8.5 Hz, 1H), 7.31 (t, *J* = 7.8 Hz, 1H), 7.20 (s, 1H), 7.06 (dq, *J* = 7.9, 14.3 Hz, 8H), 6.99 – 6.73 (m, 5H), 6.73 – 6.40 (m, 4H), 3.95 (s, 3H) ppm;

^{13}C NMR (100 MHz, $CDCl_3$) δ_C = 167.0, 157.4, 151.1, 141.9, 138.6, 138.2, 136.4, 135.2, 135.1, 132.9, 130.8, 130.6, 130.5, 130.1, 129.5, 128.8, 128.1, 127.4, 127.2, 127.0, 126.8, 126.6, 126.4, 126.1, 125.4, 125.1, 124.9, 123.9, 121.6, 52.2 ppm. The carbon attached to boron was not observed due to quadrupolar relaxation;

^{11}B NMR (128 MHz, $CDCl_3$) δ_B = 4.18 ppm.

IR (film): ν_{max} 3673, 2998, 2816, 2779, 1773, 1642, 1623, 1433, 1285, 1266, 1108, 882, 748, 704 cm^{-1}.

HRMS (ESI): m/z calculated for $C_{41}H_{31}BNO_2^+$ $[M+H]^+$, 580.2442, found, 580.2432.

HPLC analysis: HPLC conditions: Chiral column AD-H, *n*-hexane/isopropanol: 99/1, flow rate = 1.0 mL/min, wavelength = 254 nm, t_R = 16.221 min for major isomer, t_R = 31.217 min for minor isomer, 99% ee.

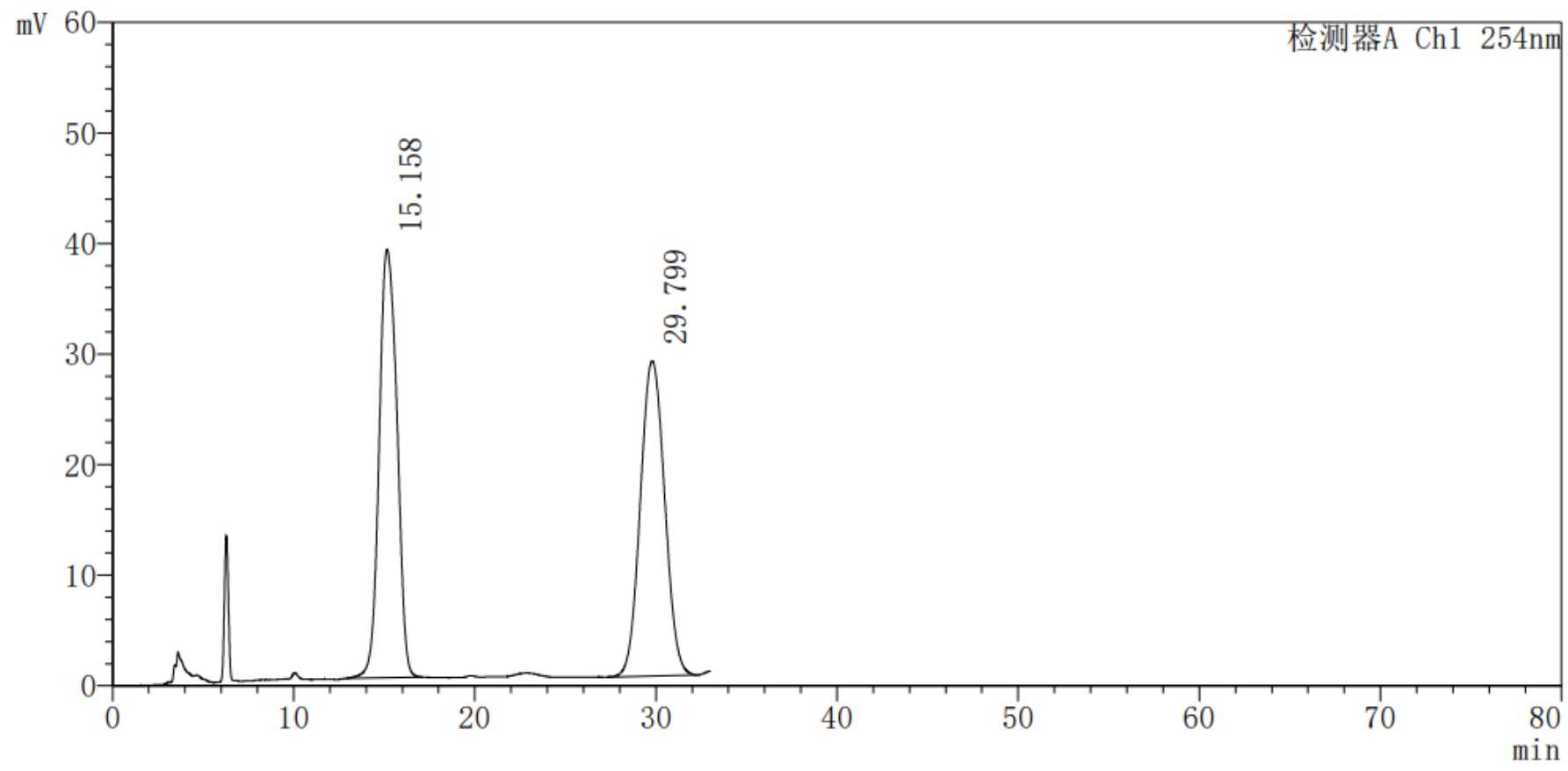

检测器A Ch1 254nm

No.	R.Time	Height	Height%	Area	Area%
1	15.158	38762	57.630	2653351	49.904
2	29.799	28498	42.370	2663527	50.096

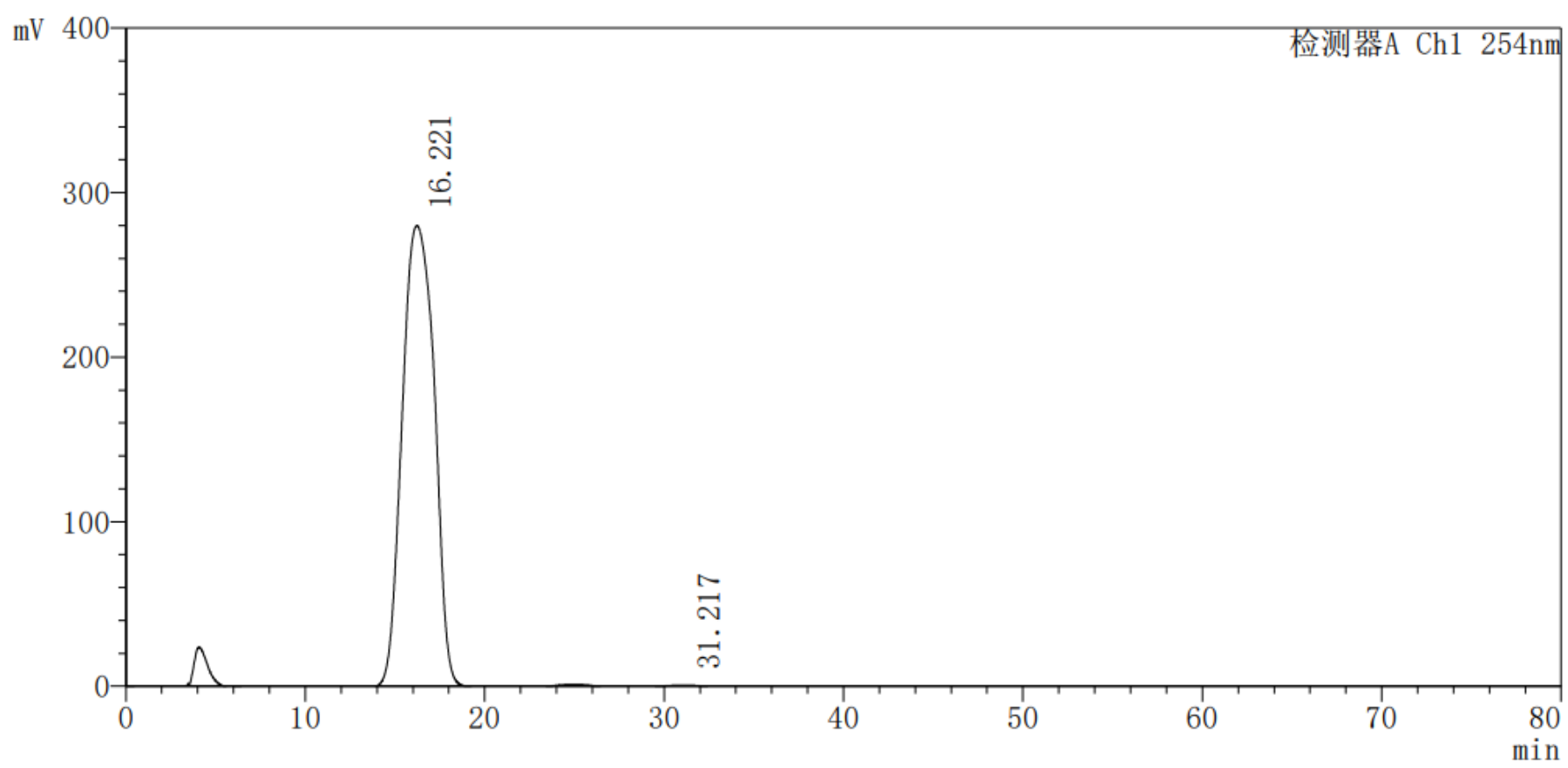

检测器A Ch1 254nm

No.	R.Time	Height	Height%	Area	Area%
1	16.221	280741	99.472	35067772	99.358
2	31.217	1490	0.528	226692	0.642

(*R*)-1-Methyl-6,6,7-triphenyl-6*H*-5l4,6l4-naphtho[2,1-e]pyrido[2,1-g][1,2]azaborepine (17)

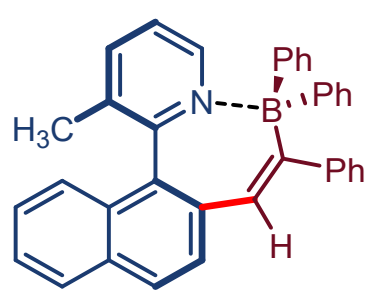

17

Prepared following **Procedure C**, using 1-(3-methylpyridin-2-yl)naphthalen-2-yl trifluoromethanesulfonate (73.4 mg, 0.2 mmol, 1.0 equiv.), tetramethylammonium ethynyltriphenylborate (102.3 mg, 0.3 mmol, 1.5 equiv.), $Pd(acac)_2$ (1.5 mg, 2.5 mol%), and (*R*)-**L4** (11.0 mg, 7.5 mol%). Purification by flash column chromatography (Petroleum ether/EtOAc: 50/1) to afford

the title compound (94.1 mg, 97%) as a yellow solid.

TLC: R_f = 0.5 (Petroleum ether/EtOAc: 50/1, $KMnO_4$ stain).

M. p.: 106 – 107 °C.

$[\alpha]^{18}_D$: +280.04 (c 0.20, CH_2Cl_2).

NMR Spectroscopy (*see spectra*):

^{1}H NMR (400 MHz, $CDCl_3$) δ_H = 8.81 (d, J = 4.9 Hz, 1H), 7.85 (d, J = 6.5 Hz, 1H), 7.60 (d, J = 8.0 Hz, 1H), 7.56 (d, J = 8.5 Hz, 1H), 7.40 – 7.34 (m, 1H), 7.29 (d, J = 5.3 Hz, 2H), 7.23 (d, J = 6.6 Hz, 1H), 7.09 (s, 1H), 7.07 – 6.92 (m, 7H), 6.91 – 6.69 (m, 6H), 6.61 – 6.40 (m, 3H), 1.83 (s, 3H) ppm;

^{13}C NMR (100 MHz, $CDCl_3$) δ_C = 154.8, 151.5, 143.9, 141.3, 138.8, 138.6, 138.3, 135.6, 130.8, 130.8, 128.8, 128.5, 128.2, 128.1, 126.8, 126.6, 126.4, 126.2, 125.3, 124.9, 124.7, 123.6, 122.3, 21.4 ppm. The carbon attached to boron was not observed due to quadrupolar relaxation;

^{11}B NMR (128 MHz, $CDCl_3$) δ_B = 3.24 ppm.

IR (film): ν_{max} 3671, 2869, 2771, 1720, 1503, 1298, 1134, 990, 880, 815, 741, 702 cm^{-1}.

HRMS (ESI): m/z calculated for $C_{36}H_{29}BN^+$ $[M+H]^+$, 486.2388, found, 486.2386.

HPLC analysis: HPLC conditions: Chiral column IB, *n*-hexane/isopropanol: 99/1, flow rate = 1.0 mL/min, wavelength = 254 nm, t_R = 7.126 min for major isomer, t_R = 10.180 min for minor isomer, 98% ee.

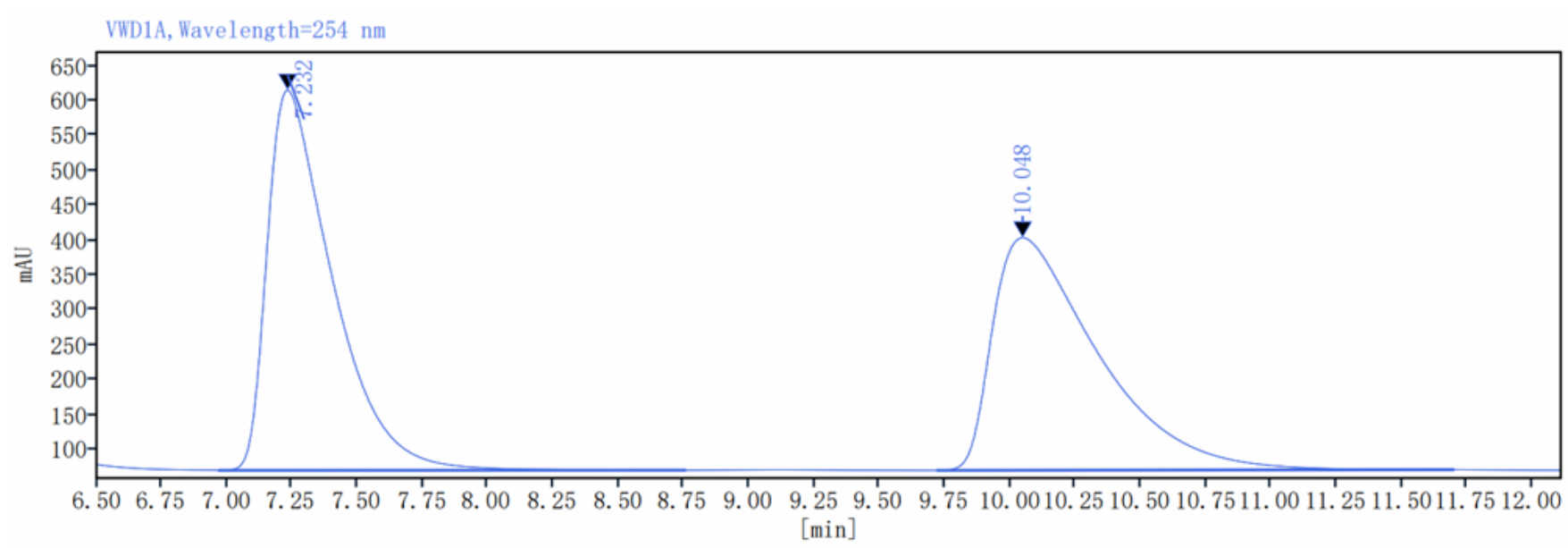

信号: VWD1A, Wavelength=254 nm

Retention Time [min]	Int Type	Width [min]	Area	Height	Area%
7.232	VB	1.79	9498.85	545.14	50.36
10.048	MM m	1.98	9364.89	333.34	49.64

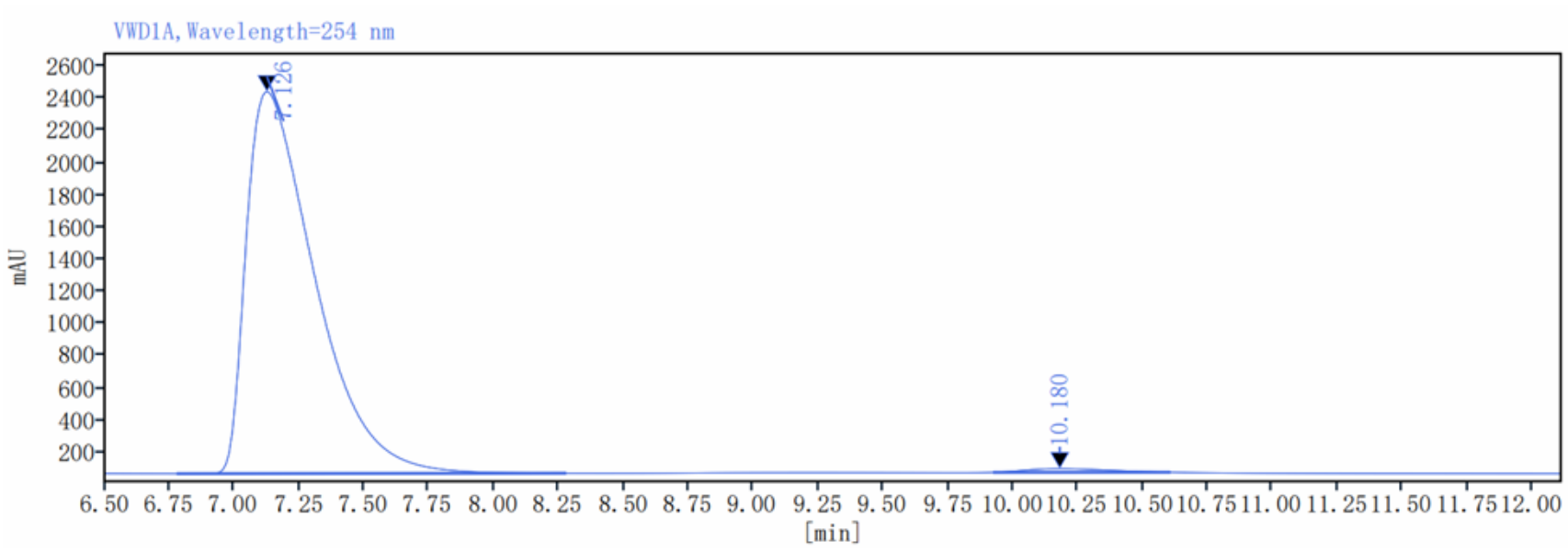

信号: VWD1A, Wavelength=254 nm

Retention Time [min]	Int Type	Width [min]	Area	Height	Area%
7.126	MM m	1.50	43624.14	2371.19	98.86
10.180	MM m	0.68	501.84	22.84	1.14

(*R*)-3-Chloro-1-methyl-6,6,7-triphenyl-6*H*-5l4,6l4-naphtho[2,1-e]pyrido[2,1-g][1,2]azaborepine (18)

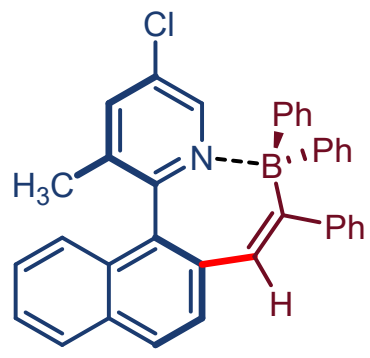

18

Prepared following **Procedure C**, using 1-(5-chloro-3-methylpyridin-2-yl)naphthalen-2-yl trifluoromethanesulfonate (80.2 mg, 0.2 mmol, 1.0 equiv.), tetramethylammonium ethynyltriphenylborate (102.3 mg, 0.3 mmol, 1.5 equiv.), $Pd(acac)_2$ (1.5 mg, 2.5 mol%), and (*R*)-**L4** (11.0 mg, 7.5 mol%). Purification by flash column chromatography (Petroleum ether/EtOAc: 50/1) to afford the title compound (100.7 mg, 97%) as a yellow solid.

TLC: R_f = 0.5 (Petroleum ether/EtOAc: 50/1, $KMnO_4$ stain).

M. p.: 94 – 95 °C.

$[\alpha]^{18}_D$: +168.34 (c 0.20, CH_2Cl_2).

NMR Spectroscopy (*see spectra*):

^{1}H NMR (500 MHz, $CDCl_3$) δ_H = 8.96 (d, *J* = 2.4 Hz, 1H), 7.88 (d, *J* = 2.4 Hz, 1H), 7.67 (d, *J* = 7.9 Hz, 1H), 7.64 (d, *J* = 8.4 Hz, 1H), 7.38 – 7.31 (m, 3H), 7.21 (s, 1H), 7.18 – 7.05 (m, 7H), 7.03 – 6.85 (m, 6H), 6.69 – 6.50 (br 2H), 6.53 (t, *J* = 7.3 Hz, 1H), 1.88 (s, 3H) ppm;

^{13}C NMR (125 MHz, $CDCl_3$) δ_C = 153.5, 151.2, 142.7, 140.8, 139.3, 138.8, 138.5, 135.7, 130.9, 130.7, 130.5, 129.1, 128.1, 127.5, 126.9, 126.6, 126.5, 126.4, 125.7, 125.14, 125.08, 124.8, 123.8, 21.4 ppm. The carbon attached to boron was not observed due to quadrupolar relaxation;

^{11}B NMR (160 MHz, $CDCl_3$) δ_B = 4.55 ppm.

IR (film): ν_{max} 3659, 3096, 2988, 1753, 1459, 1429, 1167, 909, 737, 700, 653 cm^{-1}.

HRMS (ESI): m/z calculated for $C_{36}H_{28}BClN^+$ $[M+H]^+$, 520.1998, found, 520.1993.

HPLC analysis: HPLC conditions: Chiral column IB, *n*-hexane/isopropanol: 99/1, flow rate = 1.0 mL/min, wavelength = 254 nm, t_R = 6.446 min for major isomer, t_R = 7.447 min for minor isomer, 97% ee.

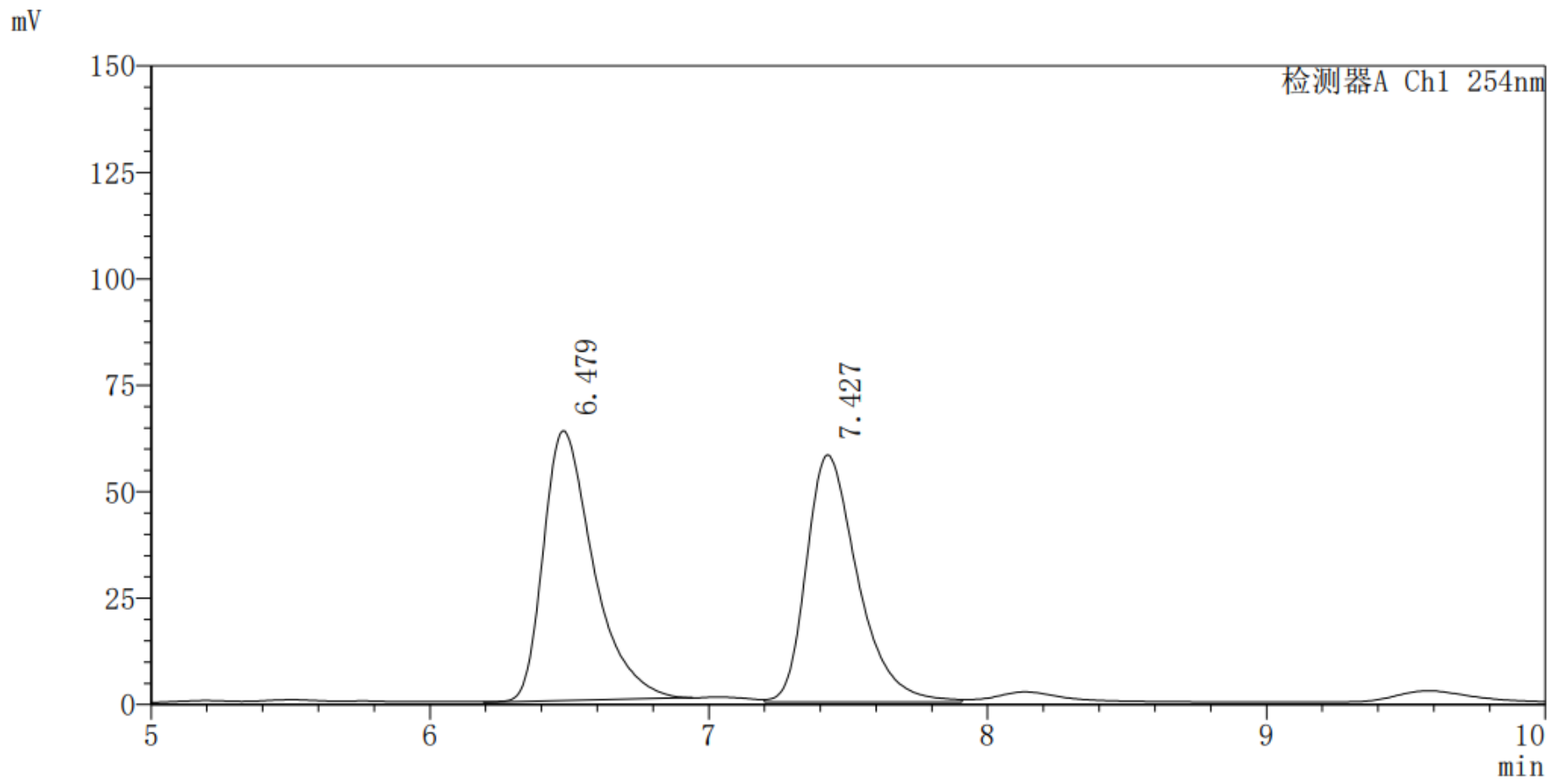

检测器A Ch1 254nm

No.	R.Time	Height	Height%	Area	Area%
1	6.479	63325	52.158	789645	51.484
2	7.427	58085	47.842	744137	48.516

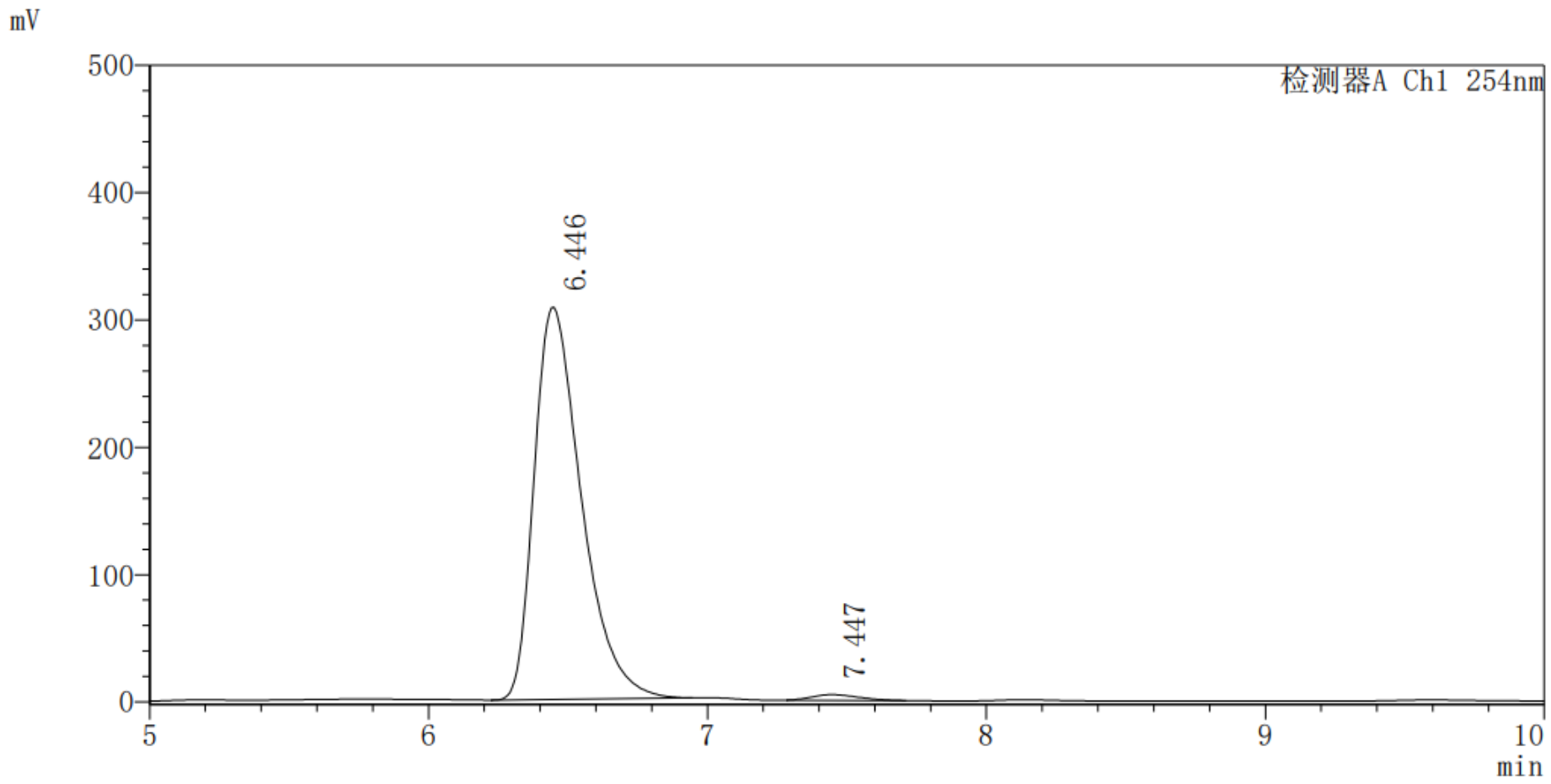

检测器A Ch1 254nm

No.	R.Time	Height	Height%	Area	Area%
1	6.446	307827	98.560	3627119	98.577
2	7.447	4497	1.440	52350	1.423

(*R*)-3-Fluoro-1-methyl-6,6,7-triphenyl-6*H*-5l4,6l4-naphtho[2,1-e]pyrido[2,1-g][1,2]azaborepine (19)

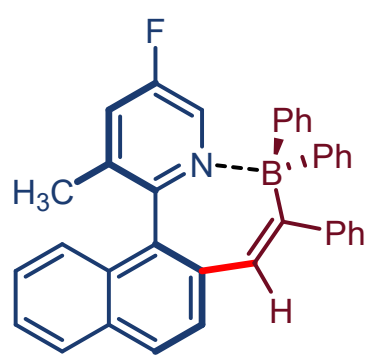

19

Prepared following **Procedure C**, using 1-(5-fluoro-3-methylpyridin-2-yl)naphthalen-2-yl trifluoromethanesulfonate (77.0 mg, 0.2 mmol, 1.0 equiv.), tetramethylammonium ethynyltriphenylborate (102.3 mg, 0.3 mmol, 1.5 equiv.), $Pd(acac)_2$ (1.5 mg, 2.5 mol%), and (*R*)-**L4** (11.0 mg, 7.5 mol%). Purification by flash column chromatography (Petroleum ether/EtOAc: 50/1) to afford the title compound (94.6 mg, 94%) as a yellow solid.

TLC: $R_f = 0.5$ (Petroleum ether/EtOAc: 50/1, $KMnO_4$ stain).

M. p.: 97 – 98 °C.

$[\alpha]^{18}_D$: +206.50 (c 0.20, CH_2Cl_2).

NMR Spectroscopy (*see spectra*):

^{1}H NMR (500 MHz, $CDCl_3$) δ_H = 8.92 – 8.84 (m, 1H), 7.67 (d, J = 6.9 Hz, 2H), 7.64 (d, J = 8.5

Hz, 1H), 7.40 – 7.31 (m, 3H), 7.21 (s, 1H), 7.19 – 7.03 (m, 7H), 6.95 (d, J = 8.4 Hz, 6H), 6.71 – 6.56 (br, 2H), 6.53 (t, J = 7.2 Hz, 1H), 1.91 (s, 3H) ppm;

^{13}C NMR (100 MHz, $CDCl_3$) δ_C = 158.1 (d, $^1J_{C\text{-}F}$ = 201.4 Hz), 151.7 (d, $^4J_{C\text{-}F}$ = 4.0 Hz), 151.2, 140.0 (d, $^3J_{C\text{-}F}$ = 5.8 Hz), 138.9, 138.6, 135.8, 133.2, 132.8 (d, $^2J_{C\text{-}F}$ = 27.7 Hz), 131.0, 130.7, 130.7, 129.0, 128.4 (d, $^2J_{C\text{-}F}$ = 14.0 Hz), 128.1, 127.5, 126.9, 126.6, 126.5, 126.4, 125.7, 125.1, 124.8, 123.8, 21.8 ppm. The carbon attached to boron was not observed due to quadrupolar relaxation;

^{11}B NMR (160 MHz, $CDCl_3$) δ_B = 5.48 ppm;

^{19}F NMR (471 MHz, $CDCl_3$) δ_F = -125.4 ppm.

IR (film): ν_{max} 3692, 2894, 1742, 1624, 1469, 1290, 1201, 1137, 845, 739, 701 cm^{-1}.

HRMS (ESI): m/z calculated for $C_{36}H_{28}BFN^+$ $[M+H]^+$, 504.2293, found, 504.2288.

HPLC analysis: HPLC conditions: Chiral column IB, *n*-hexane/isopropanol: 99/1, flow rate = 1.0 mL/min, wavelength = 254 nm, t_R = 6.965 min for major isomer, t_R = 7.950 min for minor isomer, 97% ee.

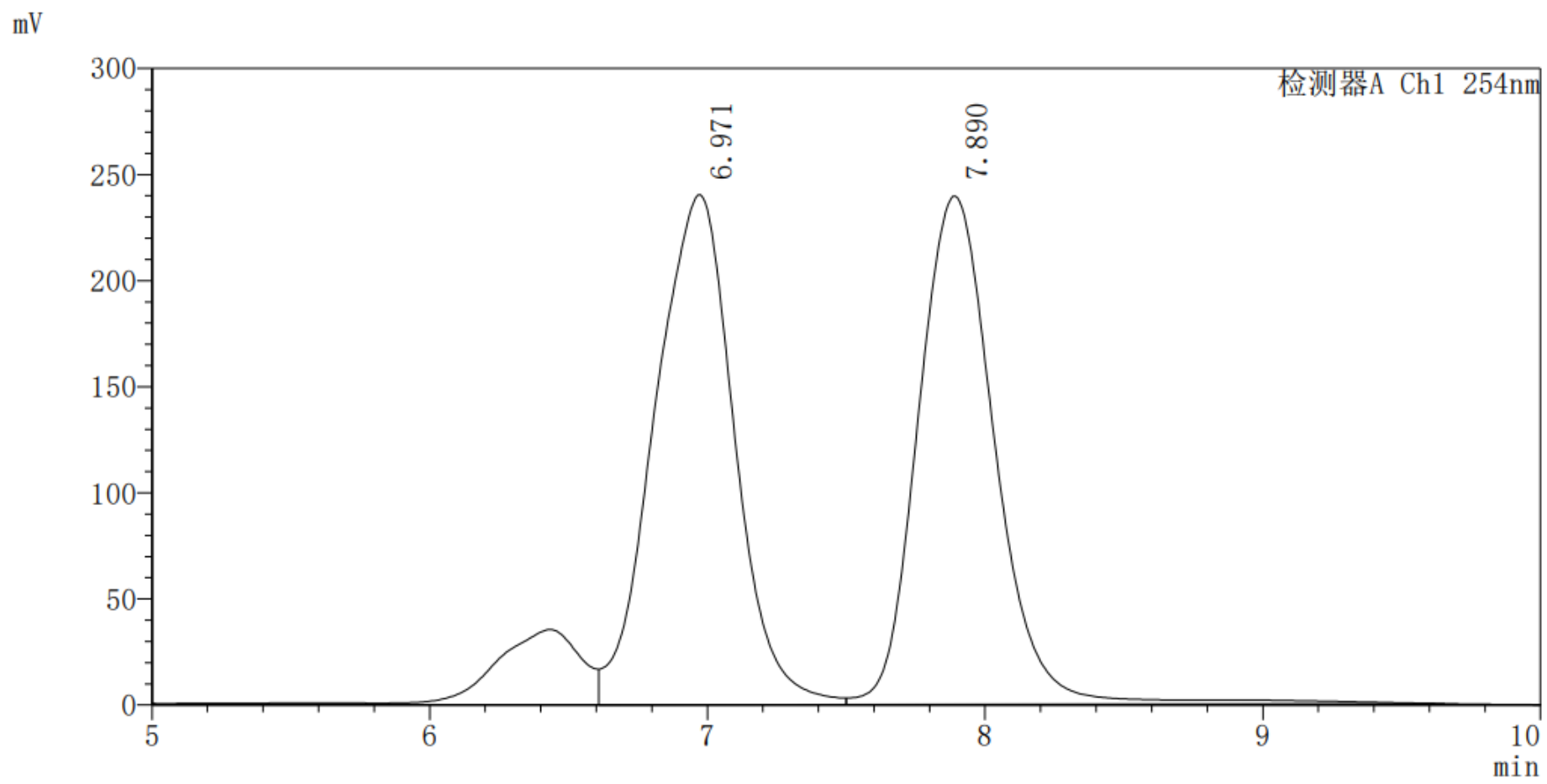

检测器A Ch1 254nm

No.	R.Time	Height	Height%	Area	Area%
1	6.971	240236	50.067	4781864	50.859
2	7.890	239591	49.933	4620345	49.141

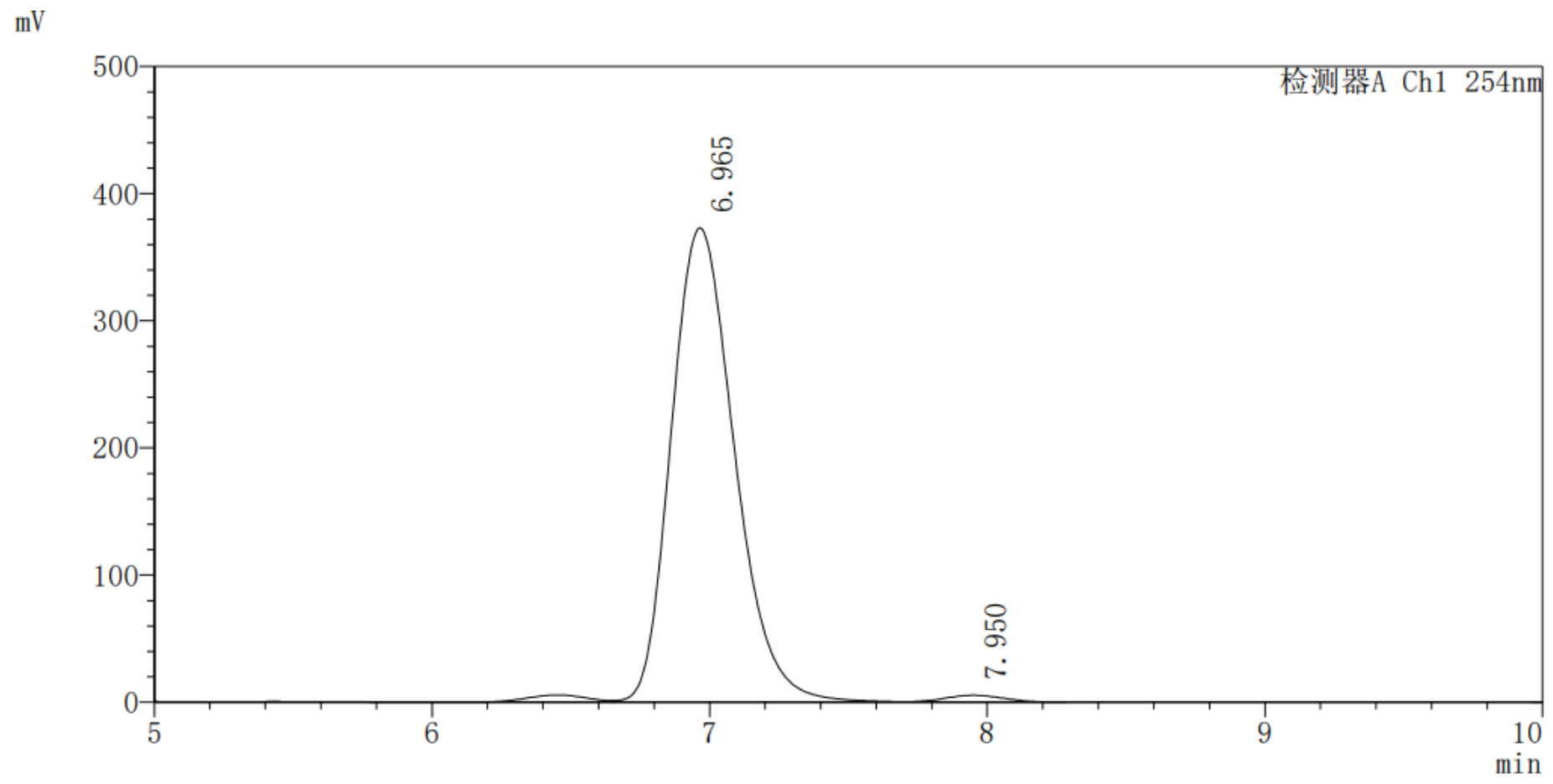

检测器A Ch1 254nm

No.	R.Time	Height	Height%	Area	Area%
1	6.965	373320	98.440	5931851	98.370
2	7.950	5915	1.560	98291	1.630

(*R*)-1-Methyl-6,6,7-triphenyl-6*H*-5l4,6l4-naphtho[2,1-e]pyrido[2,1-g][1,2]azaborepine-3-carbonitrile (20)

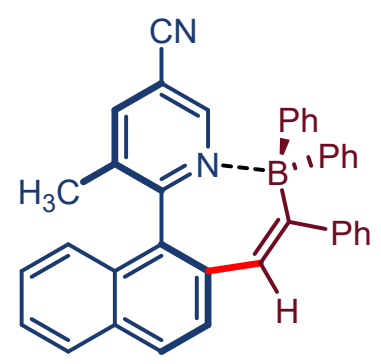

20

Prepared following **Procedure C**, using 1-(5-cyano-3-methylpyridin-2-yl)naphthalen-2-yl trifluoromethanesulfonate (78.4 mg, 0.2 mmol, 1.0 equiv.), tetramethylammonium ethynyltriphenylborate (102.3 mg, 0.3 mmol, 1.5 equiv.), $Pd(acac)_2$ (1.5 mg, 2.5 mol%), and (*R*)-**L4** (11.0 mg, 7.5 mol%). Purification by flash column chromatography (Petroleum ether/EtOAc: 50/1) to afford the title compound (100.0 mg, 98%) as a yellow solid.

TLC: $R_f = 0.5$ (Petroleum ether/EtOAc: 50/1, $KMnO_4$ stain).

M. p.: 125 – 126 °C.

$[\alpha]^{18}_D$: +92.89 (c 0.20, CH_2Cl_2).

NMR Spectroscopy (<u>*see spectra*</u>):

^{1}H NMR (400 MHz, $CDCl_3$) δ_H = 9.18 (s, 1H), 8.07 (s, 1H), 7.67 – 7.58 (m, 2H), 7.37 – 7.26 (m,

3H), 7.14 (s, 1H), 7.13 – 7.00 (m, 6H), 6.81 (dd, J = 7.5, 16.1 Hz, 7H), 6.56 (t, J = 7.4 Hz, 2H), 6.46 (t, J = 7.3 Hz, 1H), 1.88 (s, 3H) ppm;

^{13}C NMR (100 MHz, $CDCl_3$) δ_C = 159.0, 150.8, 146.3, 142.9, 139.7, 139.0, 138.4, 135.7, 130.7, 130.1, 130.0, 128.4, 127.1, 127.0, 126.9, 126.9, 126.5, 126.2, 125.5, 125.1, 124.7, 124.1, 115.0, 108.8, 21.6 ppm. The carbon attached to boron was not observed due to quadrupolar relaxation;

^{11}B NMR (128 MHz, $CDCl_3$) δ_B = 4.60 ppm.

IR (film): ν_{max} 3677, 2816, 2776, 2708, 2684, 1647, 1622, 1372, 1353, 765, 740, 701 cm^{-1}.

HRMS (ESI): m/z calculated for $C_{37}H_{28}BN_2^+$ $[M+H]^+$, 511.2340, found, 511.2339.

HPLC analysis: HPLC conditions: Chiral column IB, *n*-hexane/isopropanol: 95/5, flow rate = 1.0 mL/min, wavelength = 254 nm, t_R = 9.133 min for major isomer, t_R = 10.082 min for minor isomer, 98% ee.

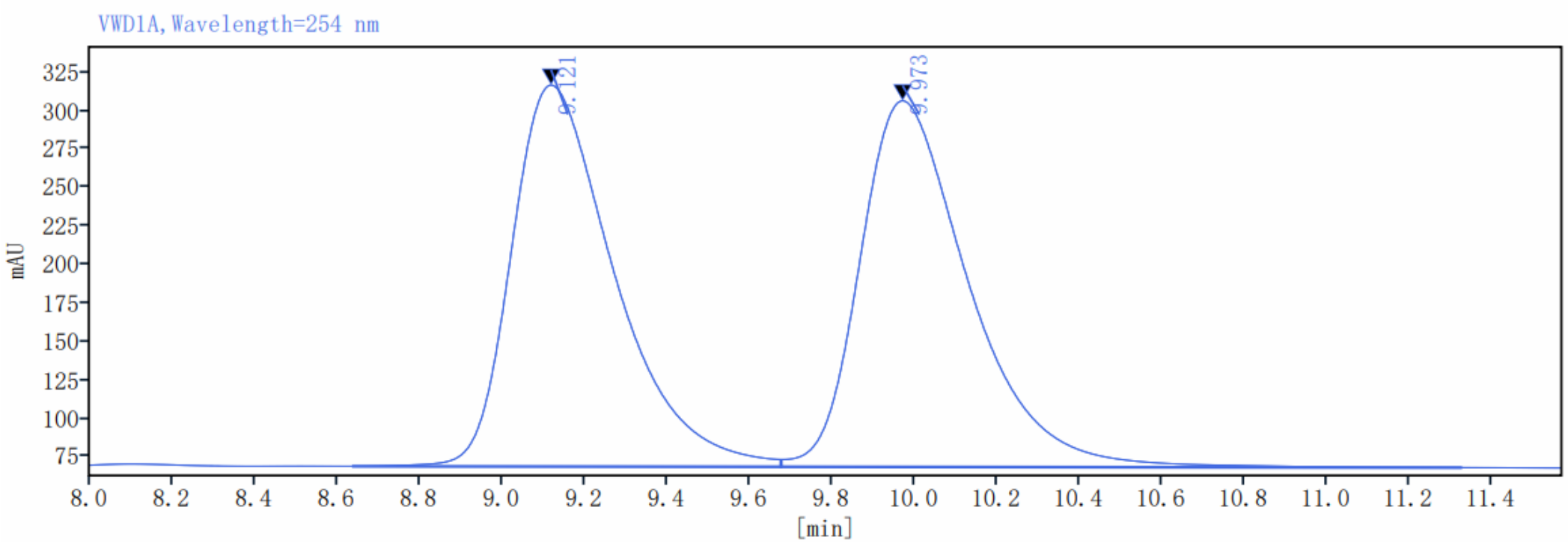

信号: VWD1A, Wavelength=254 nm

Retention Time [min]	Int Type	Width [min]	Area	Height	Area%
9.121	MM m	1.04	4302.53	248.04	49.60
9.973	MM m	1.65	4372.69	238.11	50.40

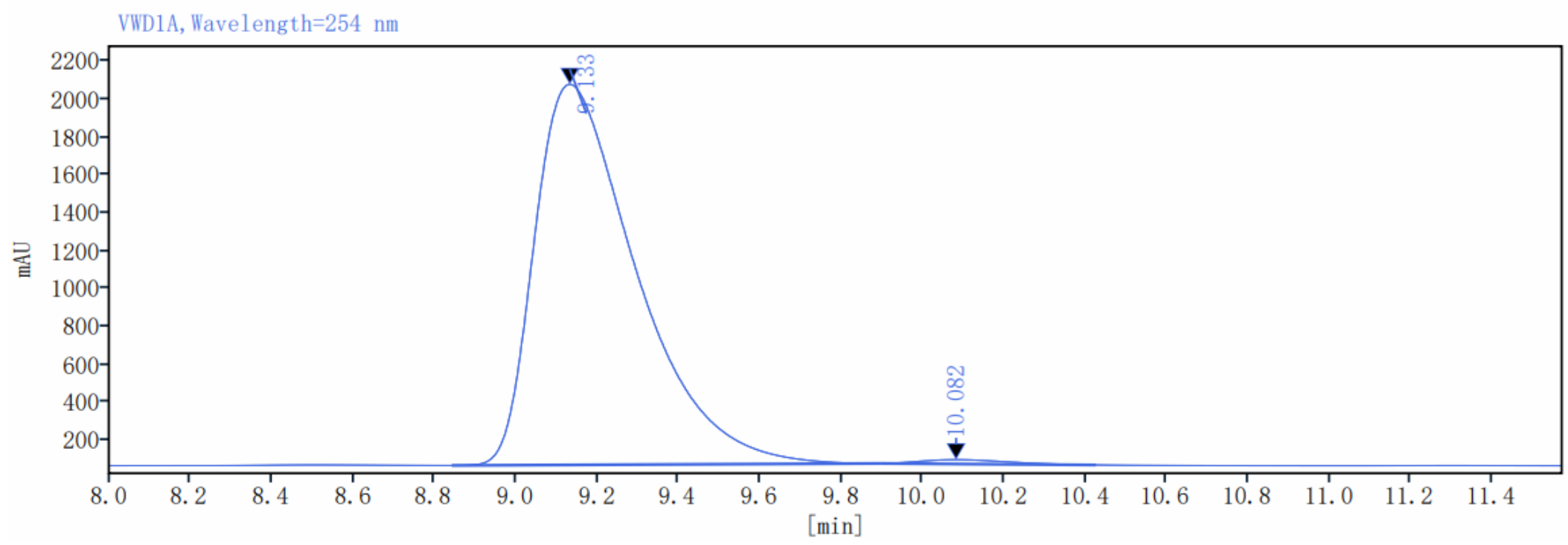

信号: VWD1A, Wavelength=254 nm

Retention Time [min]	Int Type	Width [min]	Area	Height	Area%
9.133	MM m	1.06	34960.98	2010.18	99.05
10.082	MM m	0.53	334.51	22.57	0.95

(*R*)-6,6,7-Triphenyl-1-(trifluoromethyl)-6*H*-5l4,6l4-naphtho[2,1-e]pyrido[2,1-g][1,2]azaborepine (21)

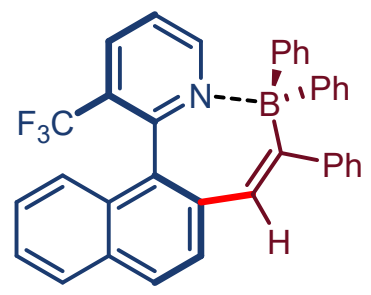

21

Prepared following **Procedure C**, using 1-[3-(trifluoromethyl)pyridin-2-yl]naphthalen-2-yl trifluoromethanesulfonate (84.2 mg, 0.2 mmol, 1.0 equiv.), tetramethylammonium ethynyltriphenylborate (102.3 mg, 0.3 mmol, 1.5 equiv.), $Pd(acac)_2$ (1.5 mg, 2.5 mol%), and (*R*)-**L4** (11.0 mg, 7.5 mol%). Purification by flash column chromatography (Petroleum ether/EtOAc: 50/1) to afford the title compound (104.6 mg, 97%) as a yellow solid.

TLC: $R_f = 0.5$ (Petroleum ether/EtOAc: 50/1, $KMnO_4$ stain).

M. p.: 114 – 115 °C.

$[\alpha]^{18}_D$: +200.00 (c 0.20, CH_2Cl_2).

NMR Spectroscopy (*see spectra*):

^{1}H NMR (500 MHz, $CDCl_3$) δ_H = 9.11 (d, *J* = 6.1 Hz, 1H), 8.36 (d, *J* = 7.9 Hz, 1H), 7.66 – 7.55 (m, 3H), 7.28 (d, *J* = 8.4 Hz, 2H), 7.22 – 7.15 (m, 2H), 7.14 – 6.73 (m, 12H), 6.69 (d, *J* = 8.4 Hz, 1H), 6.54 (t, *J* = 7.4 Hz, 2H), 6.46 (t, *J* = 7.3 Hz, 1H) ppm;

^{13}C NMR (100 MHz, CDCl$_3$) δ_C = 155.1, 151.0, 148.8, 139.1, 138.8 (q, $^3J_{C-F}$ = 4.3 Hz), 138.5, 135.9, 132.1 (q, $^3J_{C-F}$ = 2.2 Hz), 131.9 (q, $^2J_{C-F}$ = 32.6 Hz), 130.7, 130.1, 128.2 (br), 127.5, 126.9, 126.7, 126.2, 126.2, 125.9, 125.6, 125.6, 125.2, 124.9, 124.0, 122.4, 122.3 (q, $^1J_{C-F}$ = 275.0 Hz) ppm; The carbon attached to boron was not observed due to quadrupolar relaxation;

^{11}B NMR (128 MHz, CDCl$_3$) δ_B = 4.78 ppm;

^{19}F NMR (376 MHz, CDCl$_3$) δ_F = -57.91 ppm.

IR (film): ν_{max} 3065, 2816, 2779, 2708, 1640, 1452, 1372, 1310, 1126, 1059, 818, 765, 702 cm^{-1}.

HRMS (ESI): m/z calculated for $C_{36}H_{26}BF_3N^+$ [M+H]$^+$, 540.2105, found, 540.2109.

HPLC analysis: HPLC conditions: Chiral column IB, *n*-hexane/isopropanol: 98/2, flow rate = 1.0 mL/min, wavelength = 254 nm, t_R = 6.744 min for major isomer, t_R = 12.764 min for minor isomer, 90% ee.

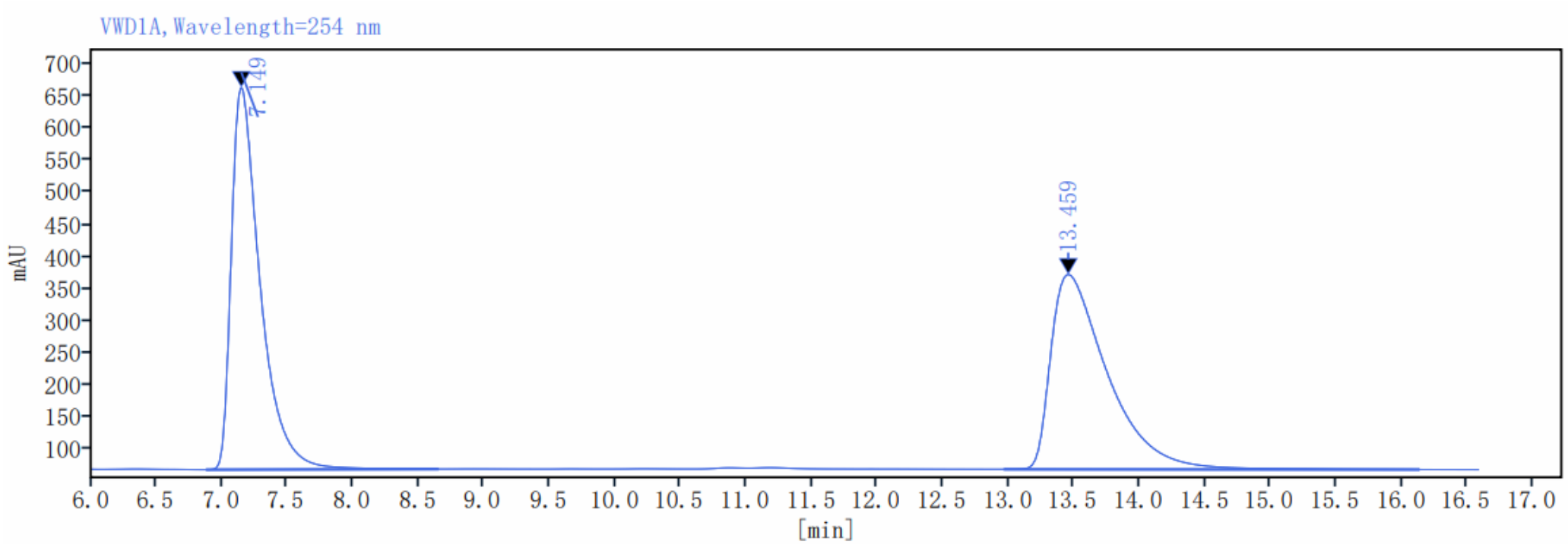

信号: VWD1A, Wavelength=254 nm

Retention Time [min]	Int Type	Width [min]	Area	Height	Area%
7.149	BB	1.77	9114.04	595.07	49.97
13.459	BM m	3.17	9123.40	303.39	50.03

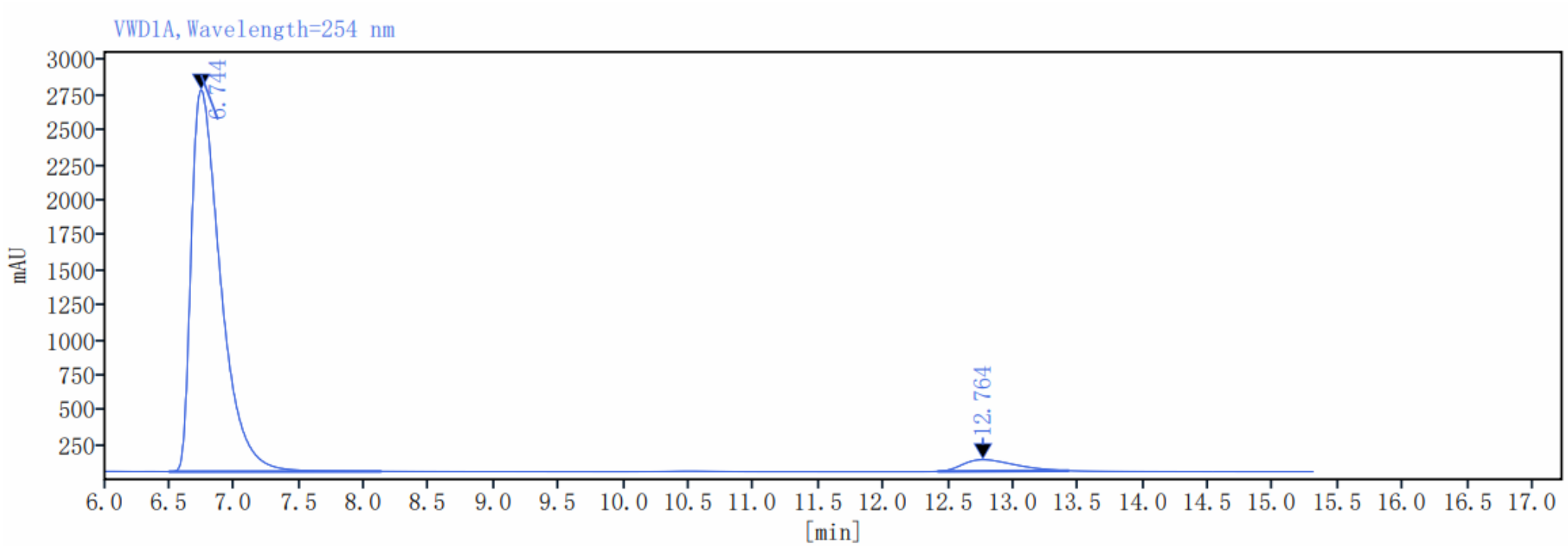

信号: VWD1A, Wavelength=254 nm

Retention Time [min]	Int Type	Width [min]	Area	Height	Area%
6.744	BM m	1.64	42827.27	2722.20	94.94
12.764	MM m	1.01	2282.11	82.22	5.06

(*R*)-2-(6,6,7-Triphenyl-6*H*-5l4,6l4-naphtho[2,1-e]pyrido[2,1-g][1,2]azaborepin-1-yl)acetonitrile (22)

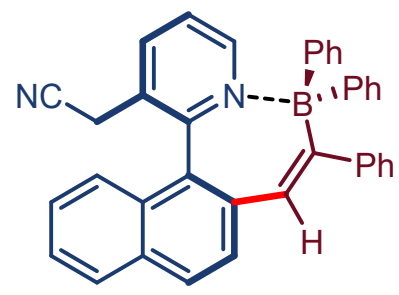

22

Prepared following **Procedure C**, using 1-[3-(cyanomethyl)pyridin-2-yl]naphthalen-2-yl trifluoromethanesulfonate (78.4 mg, 0.2 mmol, 1.0 equiv.), tetramethylammonium ethynyltriphenylborate (102.3 mg, 0.3 mmol, 1.5 equiv.), $Pd(acac)_2$ (1.5 mg, 2.5 mol%), and (*R*)-**L4** (11.0 mg, 7.5 mol%). Purification by flash column chromatography (Petroleum ether/EtOAc: 50/1) to afford the title compound (40.8 mg, 40%) as a yellow solid.

TLC: R_f = 0.5 (Petroleum ether/EtOAc: 50/1, $KMnO_4$ stain).

M. p.: 244 – 245 °C.

$[\alpha]^{18}_D$: –71.77 (c 0.20, CH_2Cl_2).

NMR Spectroscopy (*see spectra*):

^{1}**H NMR** (400 MHz, $CDCl_3$) δ_H = 9.05 (d, *J* = 7.9 Hz, 1H), 8.19 (d, *J* = 8.8 Hz, 1H), 8.08 (d, *J* = 7.8 Hz, 1H), 7.70 (d, *J* = 8.0 Hz, 1H), 7.48 – 7.39 (m, 3H), 7.27 – 7.22 (m, 2H), 7.18 – 7.12 (m, 3H), 7.06 – 6.91 (m, 7H), 6.76 (d, *J* = 6.8 Hz, 2H), 6.71 (t, *J* = 9.1 Hz, 1H), 6.47 (t, *J* = 7.3 Hz,

1H), 6.07 (s, 1H), 5.94 (d, J = 7.8 Hz, 1H), 4.24 (d, J = 10.6 Hz, 1H), 3.83 (d, J = 10.6 Hz, 1H) ppm;

^{13}C NMR (100 MHz, $CDCl_3$) δ_C = 190.6, 153.1, 149.9, 145.4, 144.8, 139.9, 138.5, 134.8, 134.6, 133.8, 132.8, 132.3, 132.0, 131.9, 131.5, 130.9, 130.8, 130.3, 128.0, 127.4, 127.3, 127.2, 126.5, 125.6, 125.3, 124.8, 124.1, 123.7, 123.4, 49.9 ppm. The carbon attached to boron was not observed due to quadrupolar relaxation;

^{11}B NMR (128 MHz, $CDCl_3$) δ_B = 3.06 ppm.

IR (film): ν_{max} 3677, 2921, 1691, 1488, 1454, 1429, 1267, 1064, 885, 706 cm^{-1}.

HRMS (ESI): m/z calculated for $C_{37}H_{28}BN_2^+$ [M+H]$^+$, 511.2340, found, 511.2347.

HPLC analysis: HPLC conditions: Chiral column IB, *n*-hexane/isopropanol: 95/5, flow rate = 1.0 mL/min, wavelength = 254 nm, t_R = 13.417 min for major isomer, t_R = 6.490 min for minor isomer, 99% ee.

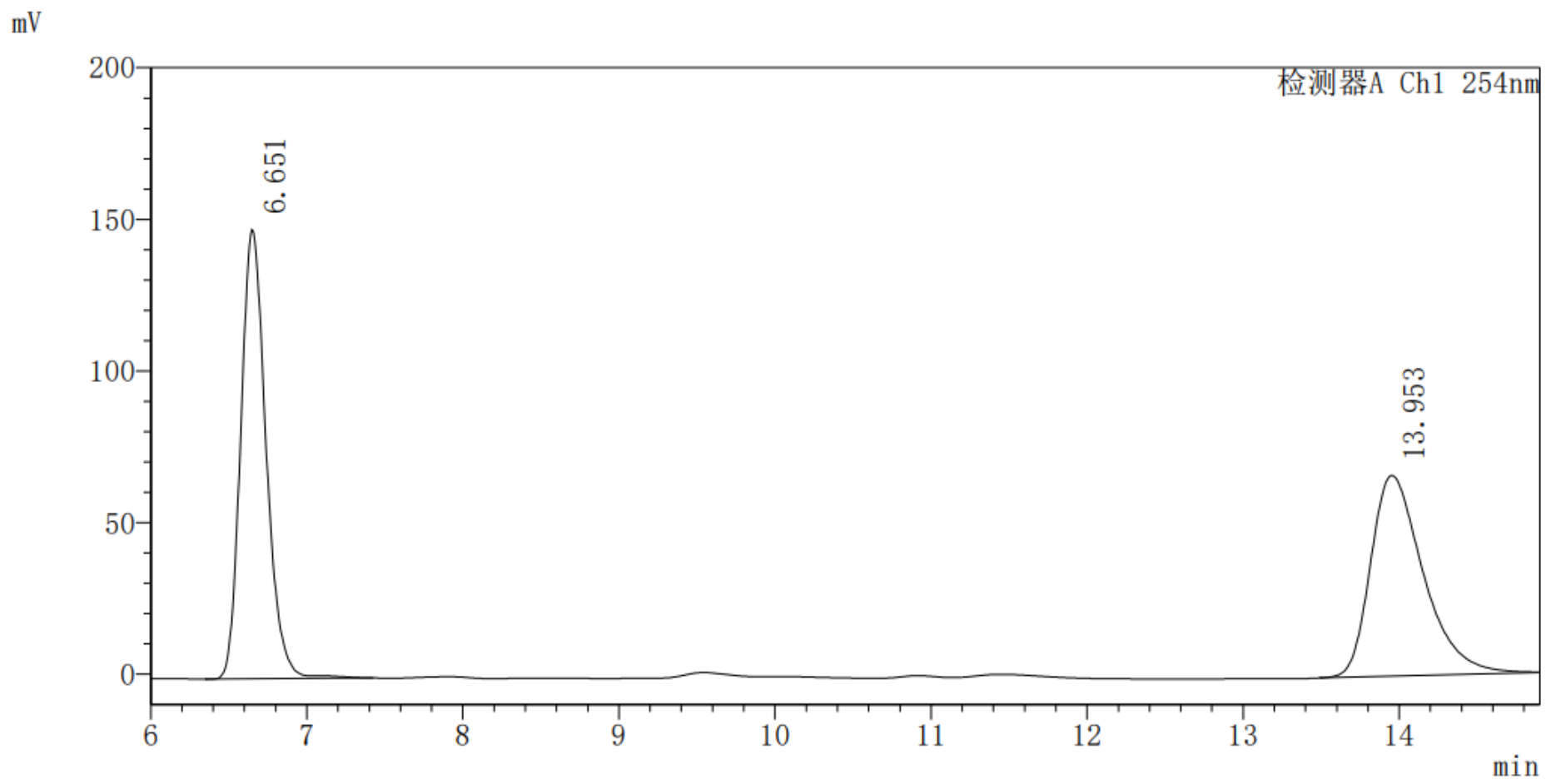

检测器A Ch1 254nm

No.	R.Time	Height	Height%	Area	Area%
1	6.651	148020	69.108	1667104	51.708
2	13.953	66167	30.892	1557000	48.292

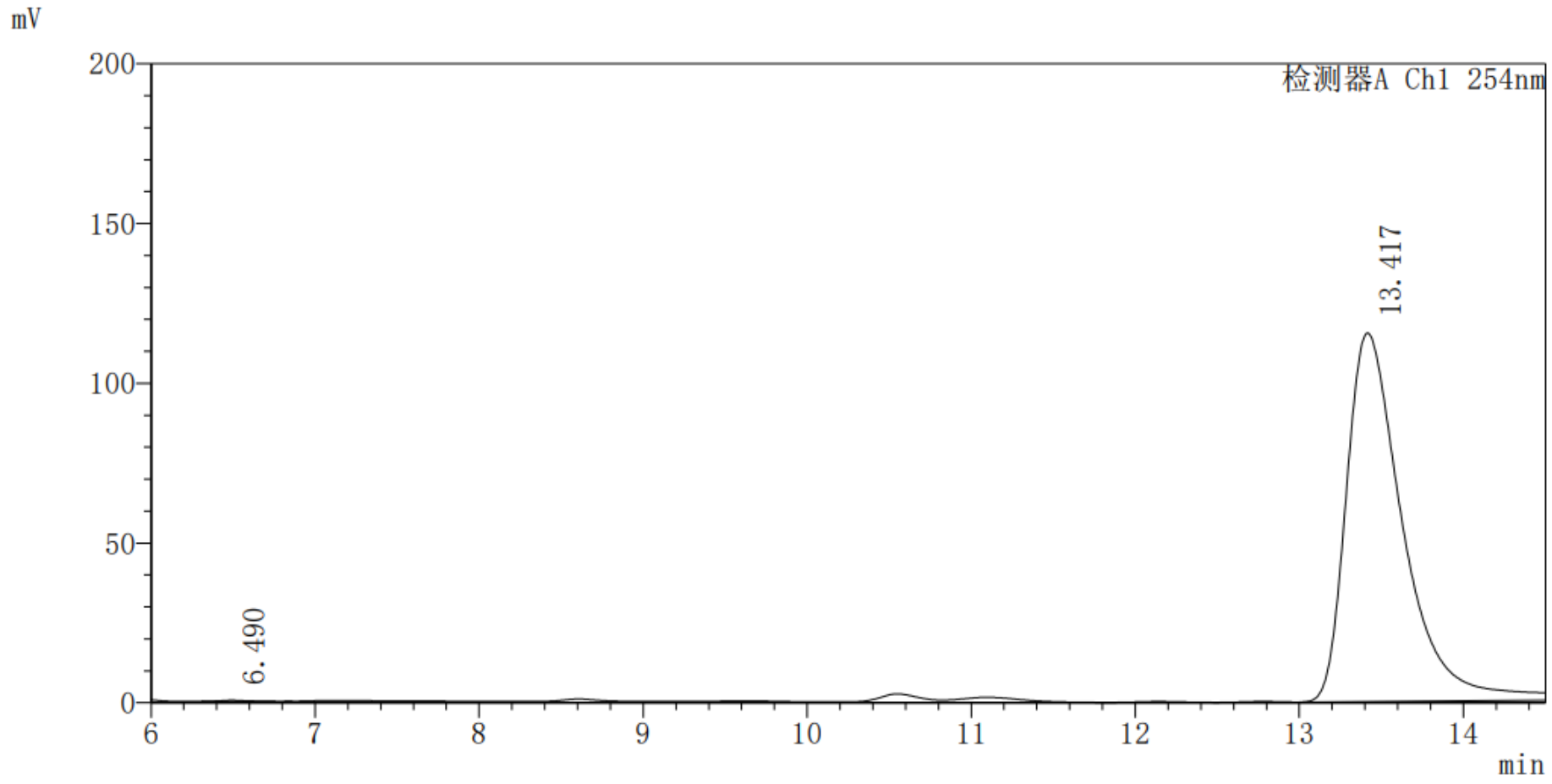

检测器A Ch1 254nm

No.	R. Time	Height	Height%	Area	Area%
1	6. 490	357	0. 309	3706	0. 133
2	13. 417	115396	99. 691	2772402	99. 867

(*R*)-1-Methyl-6,6,7-triphenyl-6*H*-5l4,6l4-naphtho[2,1-e]pyrazino[2,1-g][1,2]azaborepine (23)

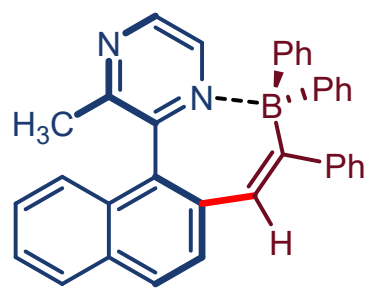

23

Prepared following **Procedure C**, using 1-(3-methylpyrazin-2-yl)naphthalen-2-yl trifluoromethanesulfonate (73.6 mg, 0.2 mmol, 1.0 equiv.), tetramethylammonium ethynyltriphenylborate (102.3 mg, 0.3 mmol, 1.5 equiv.), $Pd(acac)_2$ (1.5 mg, 2.5 mol%), and (*R*)-**L4** (11.0 mg, 7.5 mol%). Purification by flash column chromatography (Petroleum ether/EtOAc: 50/1) to afford the title compound (92.4 mg, 95%) as a yellow solid.

TLC: R_f = 0.5 (Petroleum ether/EtOAc: 50/1, $KMnO_4$ stain).

M. p.: 96 – 97 °C.

$[\alpha]^{18}_D$: +365.66 (c 0.20, CH_2Cl_2).

NMR Spectroscopy (*see spectra*):

^{1}**H NMR** (400 MHz, $CDCl_3$) δ_H = 8.74 (d, *J* = 3.6 Hz, 1H), 8.70 (d, *J* = 3.6 Hz, 1H), 7.65 (dd, *J* = 4.2, 8.2 Hz, 2H), 7.36 – 7.28 (m, 3H), 7.19 (s, 1H), 7.13 – 6.98 (m, 7H), 6.97 – 6.73 (m, 6H), 6.57 (br, 2H), 6.48 (t, *J* = 7.3 Hz, 1H). 2.15 (s, 3H) ppm;

^{13}C NMR (100 MHz, $CDCl_3$) δ_C = 160.3, 150.9, 148.2, 143.7, 139.6, 138.5, 135.8, 135.1, 130.7, 130.4, 129.8, 128.3, 127.0, 126.8, 126.7, 126.7, 126.4, 125.9, 125.5, 125.1, 124.9, 124.2, 24.9 ppm. The carbon attached to boron was not observed due to quadrupolar relaxation;

^{11}B NMR (128 MHz, $CDCl_3$) δ_B = 3.83 ppm.

IR (film): ν_{max} 3678, 2998, 2778, 1742, 1486, 1302, 1178, 910, 741, 702 cm^{-1}.

HRMS (ESI): m/z calculated for $C_{35}H_{28}BN^+$ $[M+H]^+$, 487.2340, found, 487.2338.

HPLC analysis: HPLC conditions: Chiral column IB, *n*-hexane/isopropanol: 97/3, flow rate = 1.0 mL/min, wavelength = 254 nm, t_R = 5.002 min for major isomer, t_R = 13.184 min for minor isomer, 93% ee.

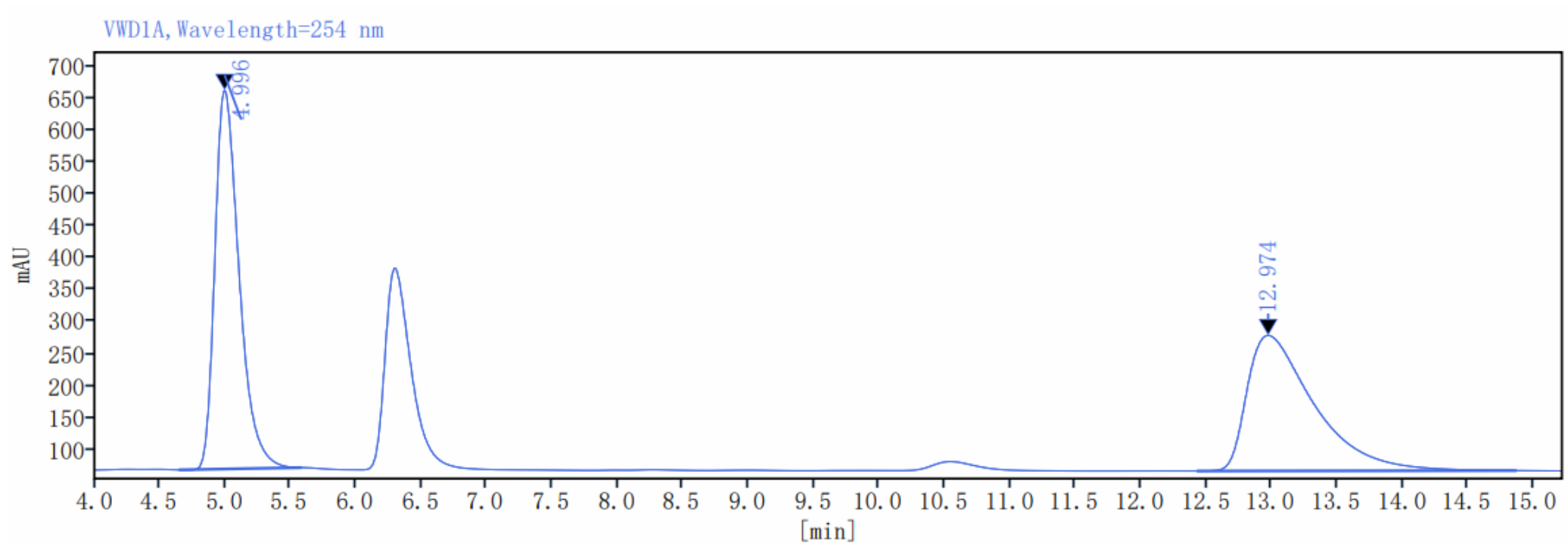

信号: VWD1A, Wavelength=254 nm

Retention Time [min]	Int Type	Width [min]	Area	Height	Area%
4.996	MM m	0.94	7631.75	591.82	50.30
12.974	BM m	2.45	7541.25	211.71	49.70

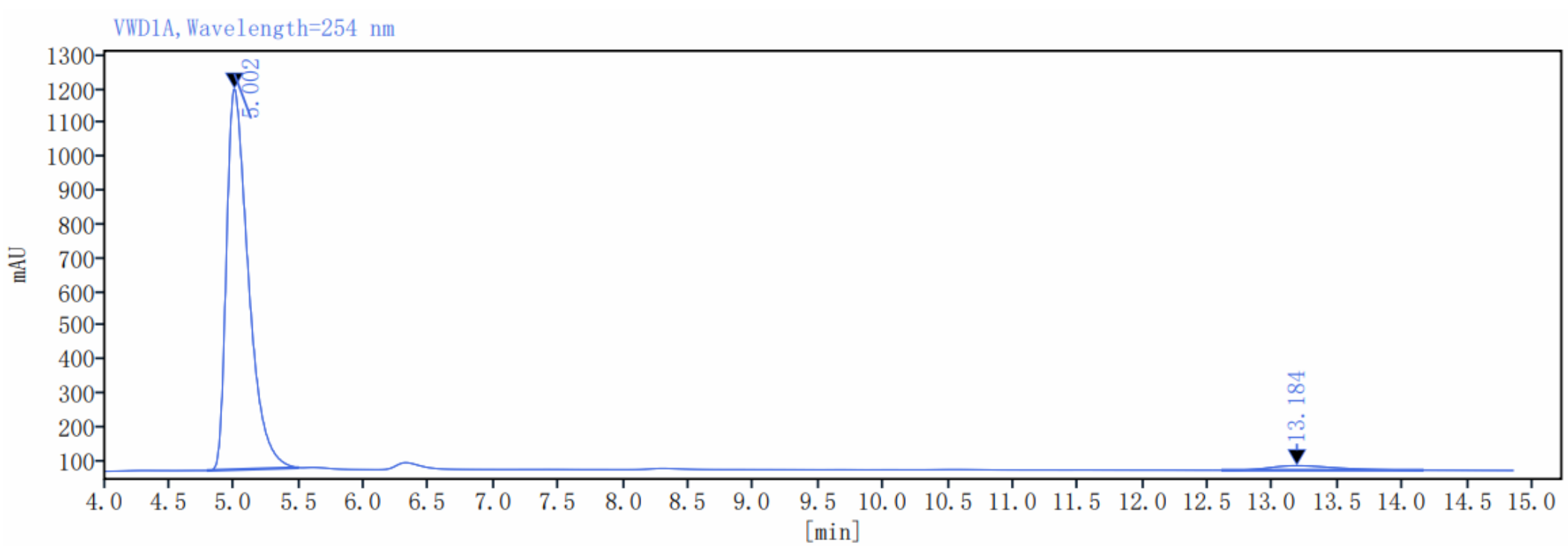

信号: VWD1A, Wavelength=254 nm

Retention Time [min]	Int Type	Width [min]	Area	Height	Area%
5.002	MM m	0.71	13208.23	1127.90	96.53
13.184	BM m	1.56	475.30	13.28	3.47

(*R*)-1,3-Dimethyl-6,6,7-triphenyl-6*H*-5l4,6l4-naphtho[2,1-e]pyrazino[2,1-g][1,2]azaborepine (24)

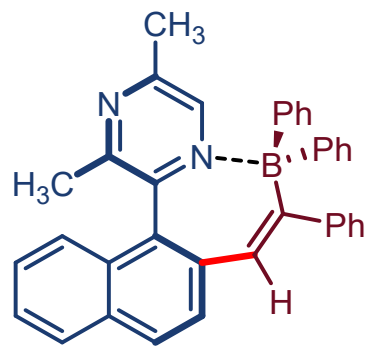

24

Prepared following **Procedure C**, using 1-(3,5-dimethylpyrazin-2-yl)naphthalen-2-yl trifluoromethanesulfonate (76.4 mg, 0.2 mmol, 1.0 equiv.), tetramethylammonium ethynyltriphenylborate (102.3 mg, 0.3 mmol, 1.5 equiv.), $Pd(acac)_2$ (1.5 mg, 2.5 mol%), and (*R*)-**L4** (11.0 mg, 7.5 mol%). Purification by flash column chromatography (Petroleum ether/EtOAc: 50/1) to afford the title compound (96.0 mg, 96%) as a yellow solid.

TLC: R_f = 0.5 (Petroleum ether/EtOAc: 50/1, $KMnO_4$ stain).

M. p.: 127 – 128 °C.

$[\alpha]^{18}_D$: +425.01 (c 0.20, CH_2Cl_2).

NMR Spectroscopy (*see spectra*):

> **^{1}H NMR** (400 MHz, $CDCl_3$) δ_H = 8.63 (s, 1H), 7.60 (t, *J* = 7.8 Hz, 2H), 7.33 – 7.26 (m, 3H), 7.18 (s, 1H), 7.08 – 7.00 (m, 7H), 6.95 – 6.69 (m, 6H), 6.66 (br, 2H), 6.55 (t, *J* = 7.3 Hz, 1H), 2.59 (s, 3H), 2.10 (s, 3H) ppm;
>
> **^{13}C NMR** (100 MHz, $CDCl_3$) δ_C = 159.0, 153.4, 150.9, 145.0, 139.4, 138.4, 135.9, 134.4, 130.7, 130.5, 129.4, 128.2, 126.9, 126.6, 126.6, 126.6, 126.5, 125.8, 125.3, 125.0, 125.0, 124.1, 24.7, 21.9 ppm. The carbon attached to boron was not observed due to quadrupolar relaxation;
>
> **^{11}B NMR** (128 MHz, $CDCl_3$) δ_B = 4.03 ppm.

IR (film): ν_{max} 3678, 3044, 2998, 1735, 1487, 1309, 1162, 878, 740, 701 cm^{-1}.

HRMS (ESI): m/z calculated for $C_{36}H_{30}BN_2^+$ $[M+H]^+$, 501.2497, found, 501.2501.

HPLC analysis: HPLC conditions: Chiral column OD–H, *n*-hexane/isopropanol: 99/1, flow rate = 1.0 mL/min, wavelength = 254 nm, t_R = 5.639 min for major isomer, t_R = 8.561 min for minor isomer, 98%

ee.

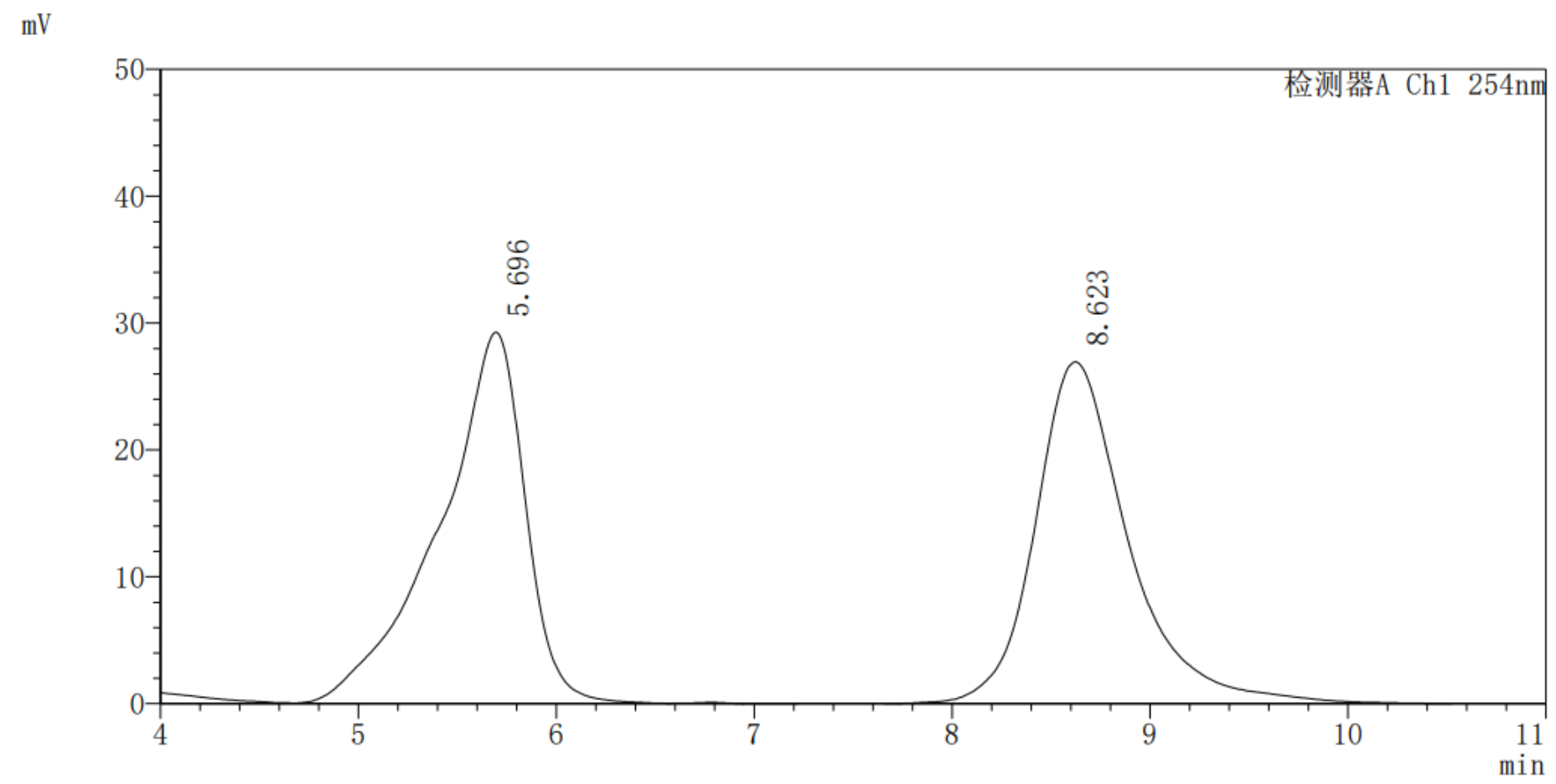

检测器A Ch1 254nm

No.	R.Time	Height	Height%	Area	Area%
1	5.696	29325	52.007	895018	49.507
2	8.623	27062	47.993	912844	50.493

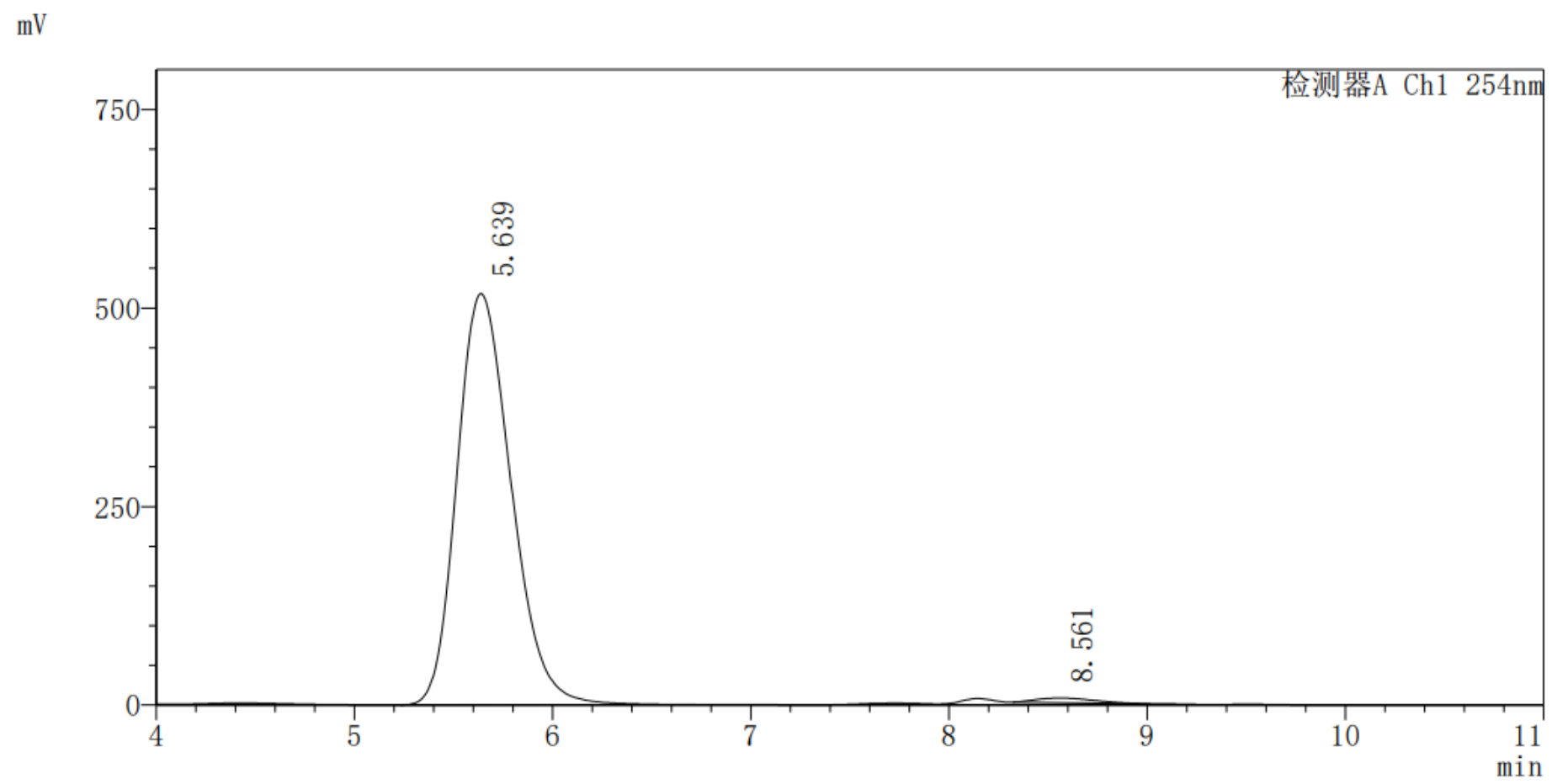

检测器A Ch1 254nm

No.	R.Time	Height	Height%	Area	Area%
1	5.639	517977	98.908	9838798	98.835
2	8.561	5720	1.092	115945	1.165

(*R*)-3-Benzyl-7,7,8-triphenyl-3,7-dihydro-6l4,7l4-naphtho[2',1':5,6][1,2]azaborepino[7,1-i]purine (25)

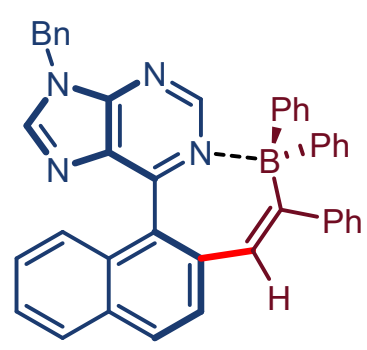

25

Prepared following **Procedure C**, using 1-(9-benzyl-9*H*-purin-6-yl)naphthalen-2-yl trifluoromethanesulfonate (96.8 mg, 0.2 mmol, 1.0 equiv.), tetramethylammonium ethynyltriphenylborate (102.3 mg, 0.3 mmol, 1.5 equiv.), Pd(acac)$_2$ (1.5 mg, 2.5 mol%), and (*R*)-**L4** (11.0 mg, 7.5 mol%). Purification by flash column chromatography (Petroleum ether/EtOAc: 50/1) to afford the title compound (116.9 mg, 97%) as a yellow solid.

TLC: R_f = 0.5 (Petroleum ether/EtOAc: 50/1, $KMnO_4$ stain).

M. p.: 259 – 260 °C.

$[\alpha]^{18}_D$: +14.34 (c 0.18, $CHCl_3$).

NMR Spectroscopy (*see spectra*):

^{1}H NMR (400 MHz, $CDCl_3$) δ_H = 9.49 (s, 1H), 8.01 (s, 1H), 7.71 (d, *J* = 8.6 Hz, 1H), 7.66 (d, *J* = 7.6 Hz, 1H), 7.42 (d, *J* = 6.5 Hz, 5H), 7.35 – 7.30 (m, 2H), 7.20 – 7.16 (m, 2H), 7.15 – 7.02 (m, 7H), 6.98 (t, *J* = 7.4 Hz, 6H), 6.64 (br, 2H), 6.52 (t, *J* = 7.3 Hz, 1H), 5.43 (s, 2H) ppm;

^{13}C NMR (100 MHz, $CDCl_3$) δ_C = 154.2, 151.0, 150.9, 150.6, 146.6, 139.3, 138.5, 136.0, 134.1, 133.8, 131.2, 131.0, 130.9, 129.5, 129.2, 128.4, 127.8, 127.0, 127.0, 126.8, 126.7, 125.6, 125.6, 125.4, 125.1, 124.5, 124.1, 48.0 ppm. The carbon attached to boron was not observed due to quadrupolar relaxation;

^{11}B NMR (128 MHz, $CDCl_3$) δ_B = 3.35 ppm.

IR (film): ν_{max} 3659, 3046, 2998, 2779, 1665, 1489, 1466, 1344, 1209, 1137, 741, 708 cm^{-1}.

HRMS (ESI): m/z calculated for $C_{42}H_{32}BN_4^+$ $[M+H]^+$, 603.2715, found, 603.2710.

HPLC analysis: HPLC conditions: Chiral column AD-H, *n*-hexane/isopropanol: 95/5, flow rate = 0.8 mL/min, wavelength = 254 nm, t_R = 11.632 min for major isomer, t_R = 12.940 min for minor isomer, 0% ee.

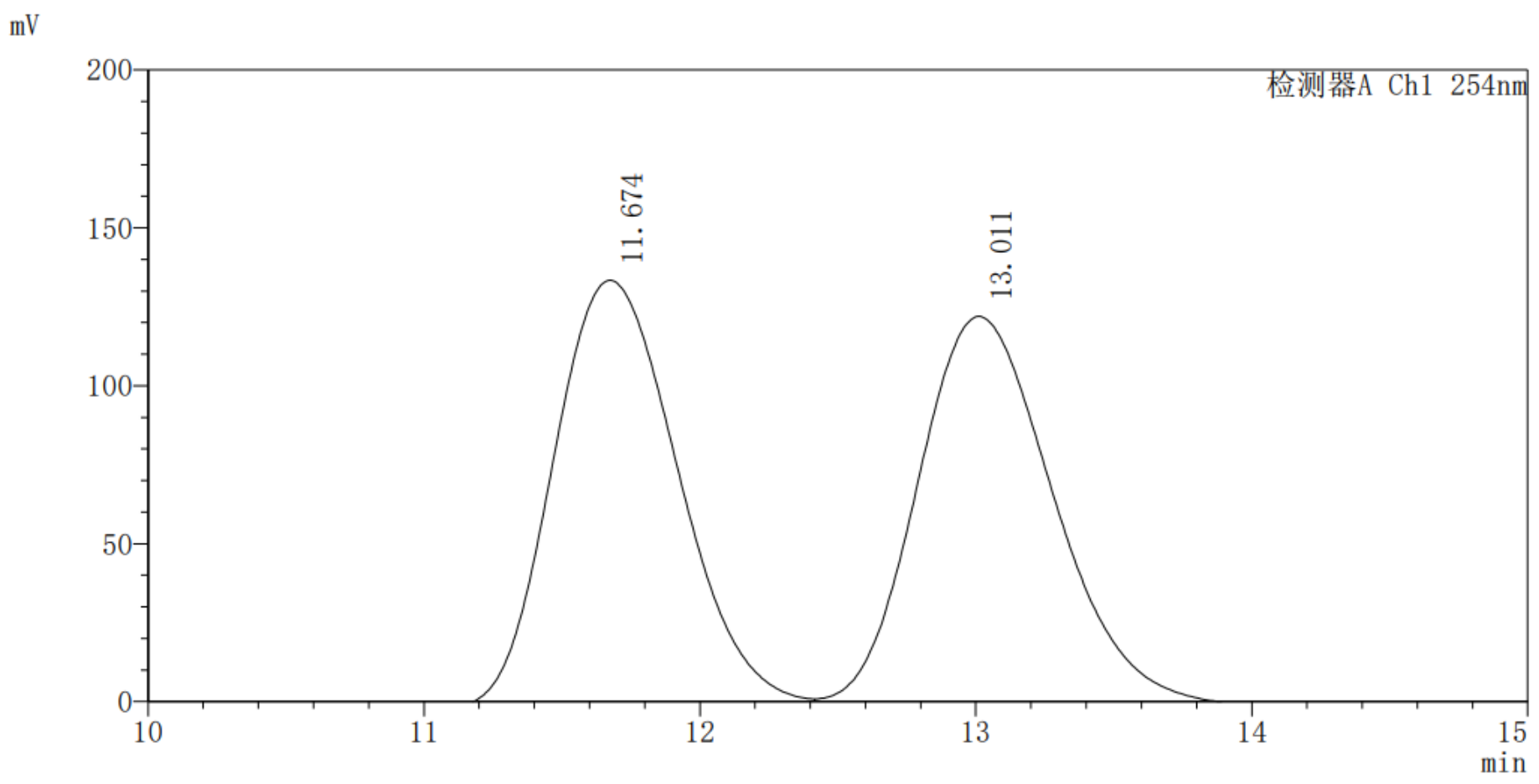

检测器A Ch1 254nm

No.	R.Time	Height	Height%	Area	Area%
1	11.674	135401	52.214	4271201	49.734
2	13.011	123919	47.786	4316938	50.266

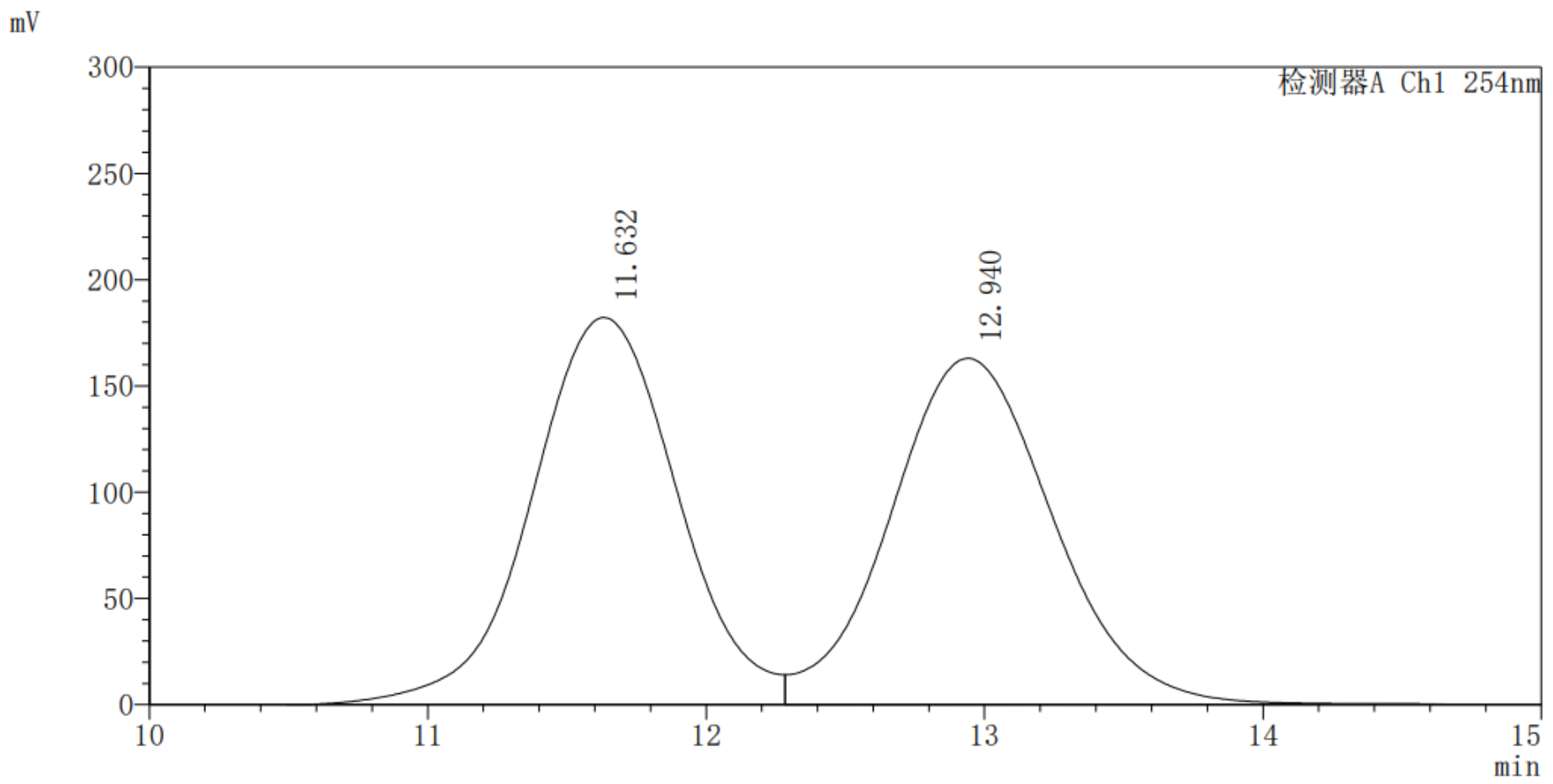

检测器A Ch1 254nm

No.	R.Time	Height	Height%	Area	Area%
1	11.632	182387	52.814	6649780	50.167
2	12.940	162954	47.186	6605607	49.833

(*R*)-4,4,5-Triphenyl-16-(thiophen-3-yl)-4*H*-3l4,4l4-naphtho[2',1':5,6][1,2]azaborepino[7,1-a]isoquinoline (26)

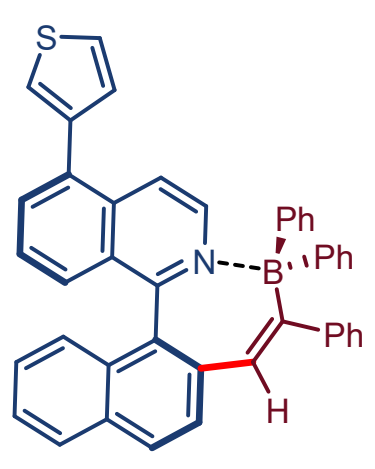

26

Prepared following **Procedure C**, using 1-[5-(thiophen-3-yl)isoquinolin-1-yl]naphthalen-2-yl trifluoromethanesulfonate (97.0 mg, 0.2 mmol, 1.0 equiv.), tetramethylammonium ethynyltriphenylborate (102.3 mg, 0.3 mmol, 1.5 equiv.), $Pd(acac)_2$ (1.5 mg, 2.5 mol%), and (*R*)-**L4** (11.0 mg, 7.5 mol%). Purification by flash column chromatography (Petroleum ether/EtOAc: 50/1) to afford the title compound (115.8 mg, 96%) as a yellow solid.

TLC: $R_f = 0.5$ (Petroleum ether/EtOAc: 50/1, $KMnO_4$ stain).

M. p.: 253 – 254 °C.

$[\alpha]^{18}_D$: +186.02 (c 0.20, CH_2Cl_2).

NMR Spectroscopy (*see spectra*):

^{1}H NMR (400 MHz, $CDCl_3$) δ_H = 8.81 (d, *J* = 7.1 Hz, 1H), 8.06 (d, *J* = 7.1 Hz, 1H), 7.86 – 7.66 (m, 3H), 7.63 – 7.48 (m, 2H), 7.45 – 7.22 (m, 6H), 7.20 – 6.88 (m, 13H), 6.77 – 6.49 (m, 4H) ppm;

^{13}C NMR (100 MHz, $CDCl_3$) δ_C = 158.2, 151.2, 140.0, 138.6, 138.3, 135.5, 134.9, 134.2, 132.9, 132.9, 130.7, 130.7, 130.5, 129.4, 129.0, 128.2, 128.0, 127.7, 127.4, 127.3, 126.8, 126.8, 126.7, 126.4, 126.1, 125.8, 125.4, 124.9, 124.8, 123.7, 119.4 ppm. The carbon attached to boron was not observed due to quadrupolar relaxation;

^{11}B NMR (128 MHz, $CDCl_3$) δ_B = 4.60 ppm.

IR (film): ν_{max} 3678, 3041, 2973, 1721, 1487, 1428, 1162, 882, 740, 705, 657 cm^{-1}.

HRMS (ESI): m/z calculated for $C_{43}H_{31}BNS^+$ $[M+H]^+$, 604.2265, found, 604.2275.

HPLC analysis: HPLC conditions: Chiral column OD–H, *n*-hexane/isopropanol: 90/10, flow rate = 1.0 mL/min, wavelength = 254 nm, t_R = 6.561 min for major isomer, t_R = 10.154 min for minor isomer, 98% ee.

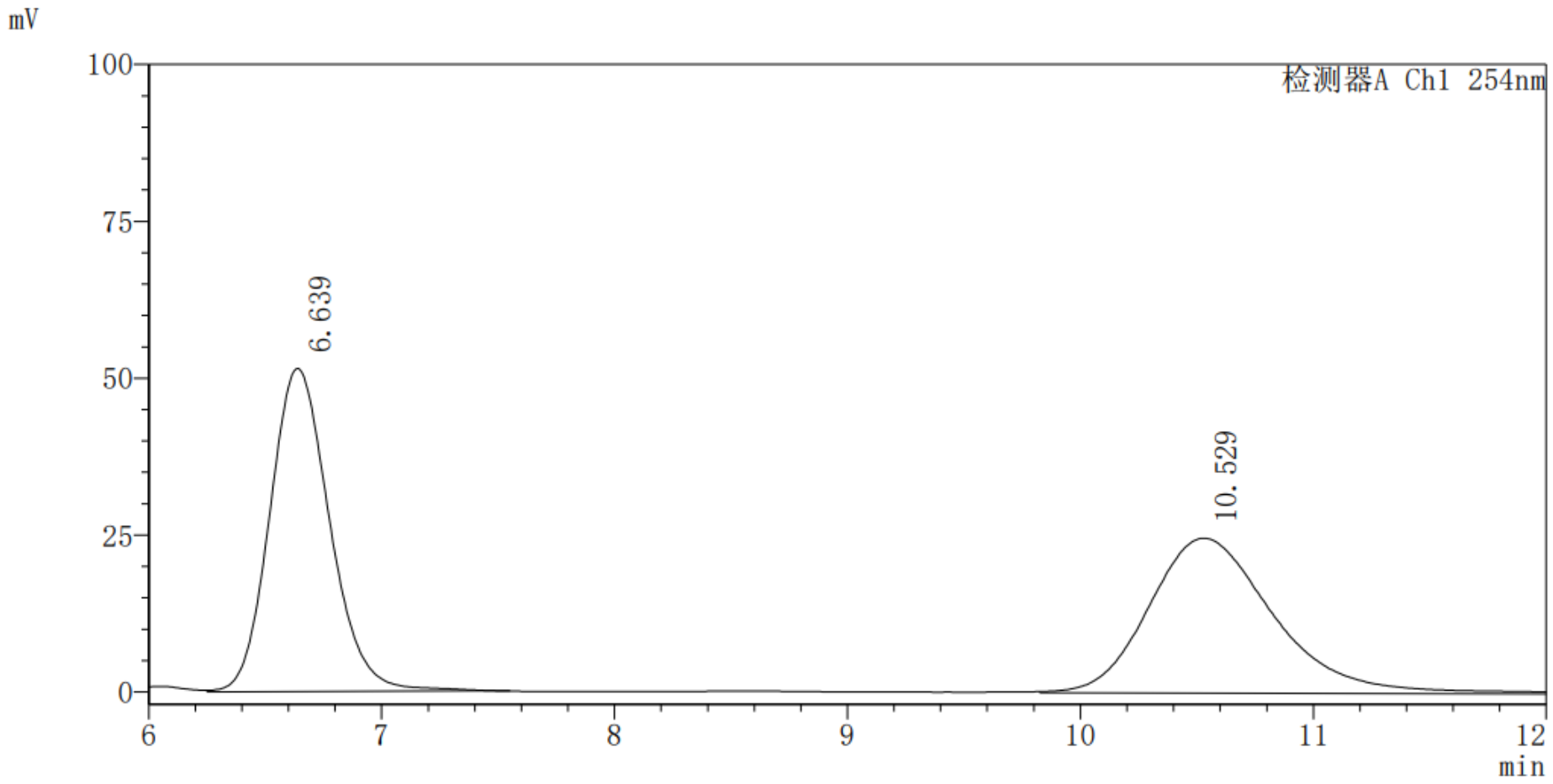

检测器A Ch1 254nm

No.	R.Time	Height	Height%	Area	Area%
1	6.639	51518	67.602	919493	49.894
2	10.529	24690	32.398	923405	50.106

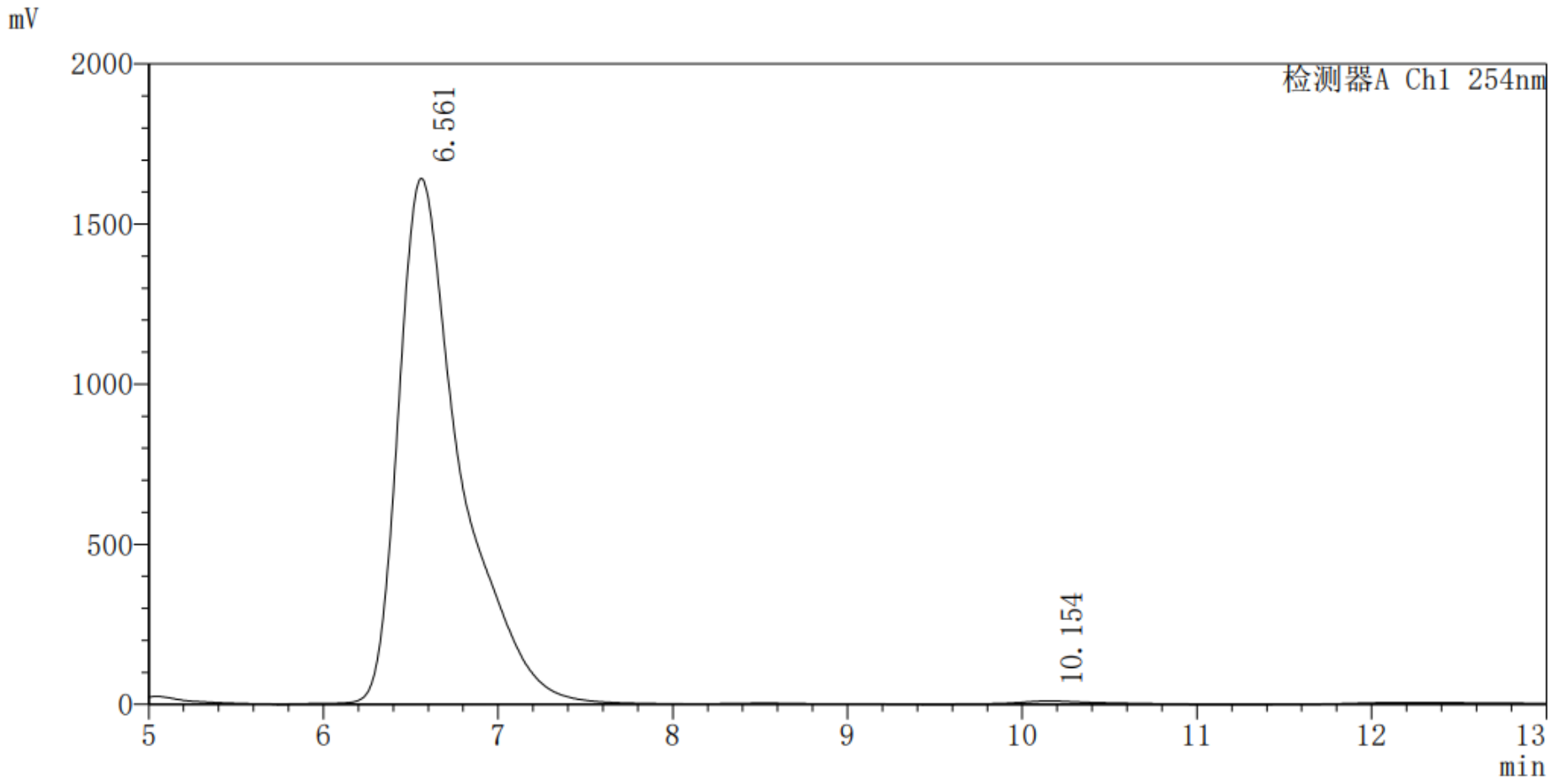

检测器A Ch1 254nm

No.	R.Time	Height	Height%	Area	Area%
1	6.561	1642108	99.383	40541901	99.164
2	10.154	10201	0.617	341943	0.836

(*R*)-16-(Benzofuran-2-yl)-4,4,5-triphenyl-4*H*-3l4,4l4-naphtho[2',1':5,6][1,2]azaborepino[7,1-a]isoquinoline (27)

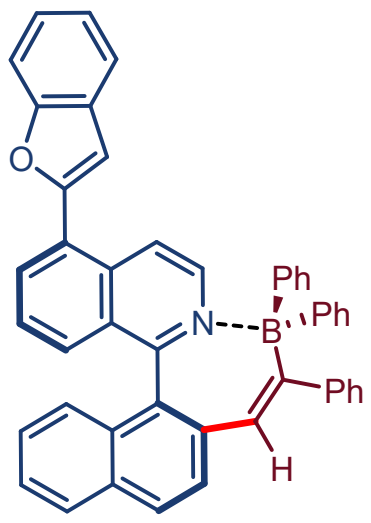

27

Prepared following **Procedure C**, using 1-[5-(benzofuran-2-yl)isoquinolin-1-yl]naphthalen-2-yl trifluoromethanesulfonate (103.8 mg, 0.2 mmol, 1.0 equiv.), tetramethylammonium ethynyltriphenylborate (102.3 mg, 0.3 mmol, 1.5 equiv.), $Pd(acac)_2$ (1.5 mg, 2.5 mol%), and (*R*)-**L4** (11.0 mg, 7.5 mol%). Purification by flash column chromatography (Petroleum ether/EtOAc: 50/1) to afford the title compound (119.8 mg, 94%) as a yellow solid.

TLC: $R_f = 0.5$ (Petroleum ether/EtOAc: 50/1, $KMnO_4$ stain).

M. p.: 134 – 135 °C.

$[\alpha]^{18}_D$: +148.04 (c 0.20, CH_2Cl_2).

NMR Spectroscopy (*see spectra*):

^{1}H NMR (400 MHz, $CDCl_3$) δ_H = 8.92 (d, J = 7.3 Hz, 1H), 8.54 (d, J = 7.3 Hz, 1H), 8.13 (d, J = 8.3 Hz, 1H), 7.75 (t, J = 7.1 Hz, 2H), 7.70 (d, J = 7.9 Hz, 1H), 7.66 (d, J = 8.0 Hz, 1H), 7.49 – 7.42 (m, 2H), 7.41 – 7.36 (t, J = 8.7 Hz , 1H), 7.35 – 7.26 (m, 4H), 7.24 – 6.87 (m, 14H), 6.79 – 6.52 (m, 4H) ppm;

^{13}C NMR (100 MHz, $CDCl_3$) δ_C = 158.4, 155.2, 153.3, 151.2, 140.0, 138.8, 138.6, 135.5, 133.7, 133.0, 132.7, 132.2, 131.0, 130.5, 129.6, 128.5, 128.2, 128.0, 127.7, 127.5, 127.2, 126.9, 126.5, 126.1, 125.9, 125.5, 125.3, 124.9, 124.8, 123.7, 123.5, 121.5, 119.2, 111.5, 107.1 ppm. The carbon attached to boron was not observed due to quadrupolar relaxation;

^{11}B NMR (128 MHz, $CDCl_3$) δ_B = 4.78 ppm.

IR (film): ν_{max} 3672, 2779, 1731, 1621, 1407, 1454, 1257, 1165, 883, 815, 741, 702, 657 cm^{-1}.

HRMS (ESI): m/z calculated for $C_{47}H_{33}BNO^+$ $[M+H]^+$, 638.2650, found, 638.2654.

HPLC analysis: HPLC conditions: Chiral column OD-H, *n*-hexane/isopropanol: 93/7, flow rate = 1.0 mL/min, wavelength = 254 nm, t_R = 7.706 min for major isomer, t_R = 9.645 min for minor isomer, 97% ee.

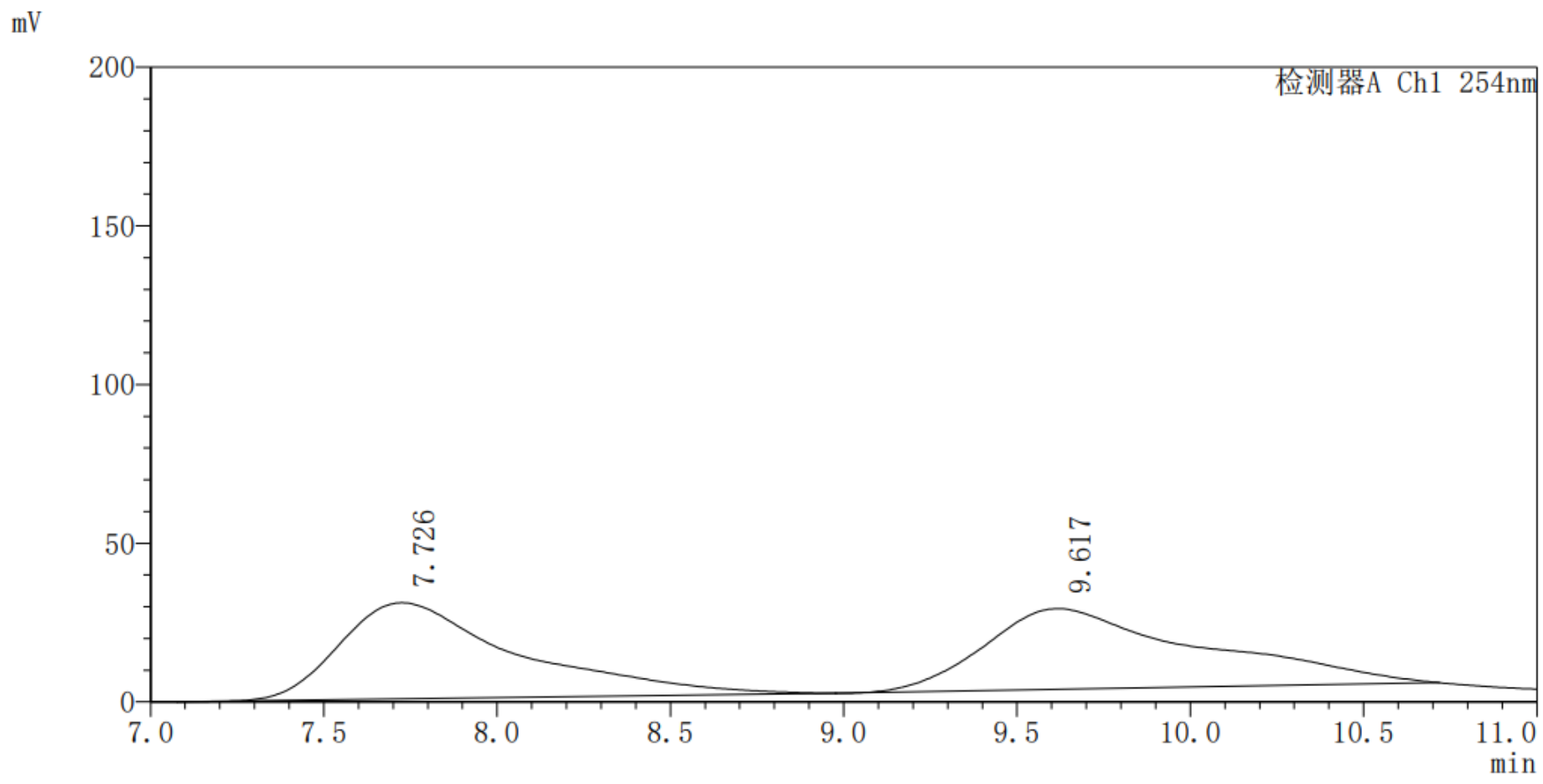

检测器A Ch1 254nm

No.	R.Time	Height	Height%	Area	Area%
1	7.726	30269	54.341	1078138	49.583
2	9.617	25432	45.659	1096261	50.417

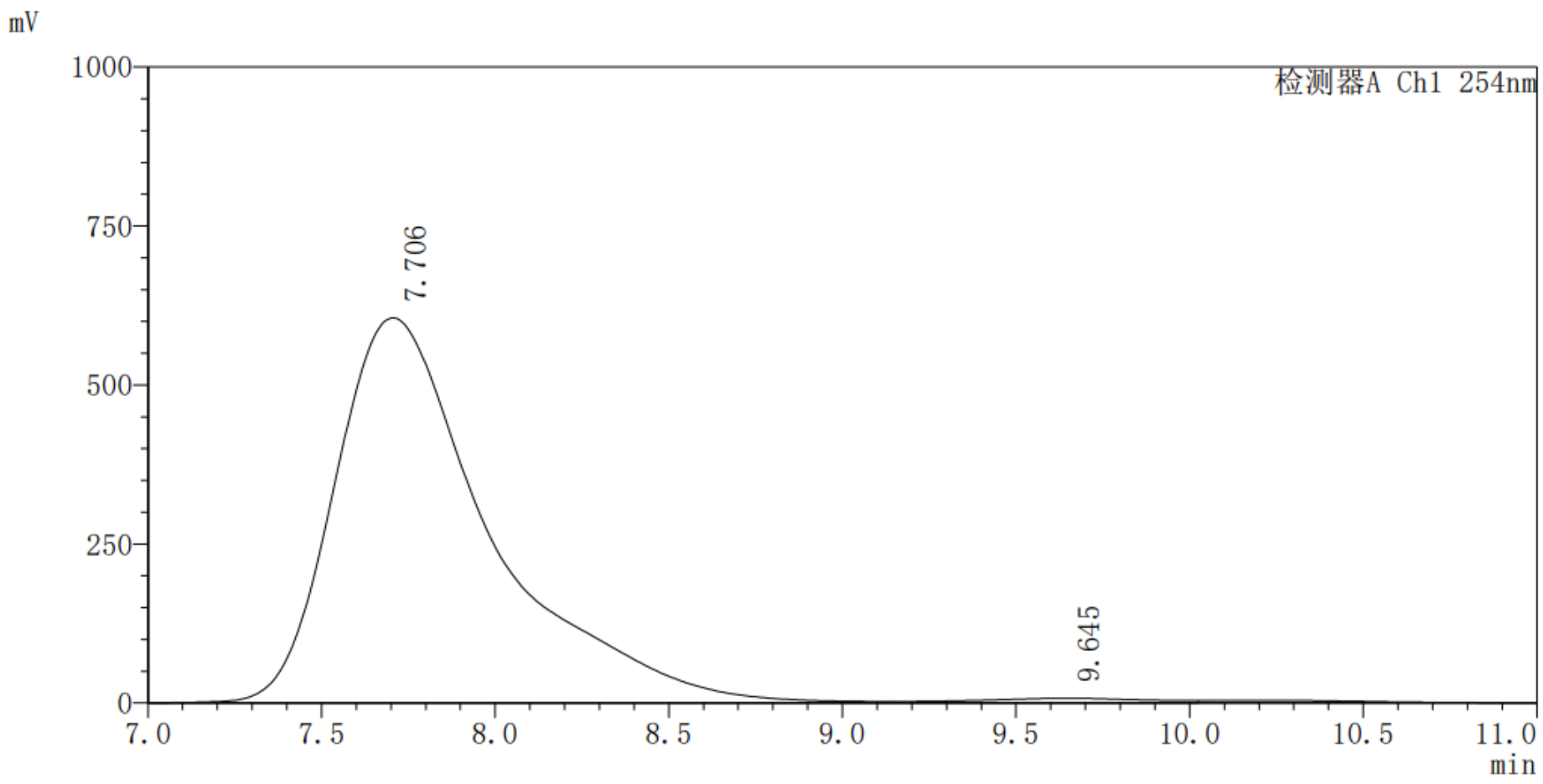

检测器A Ch1 254nm

No.	R.Time	Height	Height%	Area	Area%
1	7.706	606057	98.729	18950435	98.527
2	9.645	7799	1.271	283299	1.473

(*R*)-4,4,5-*tri*-*p*-Tolyl-4*H*-3l4,4l4-naphtho[2',1':5,6][1,2]azaborepino[7,1-a]isoquinoline (28)

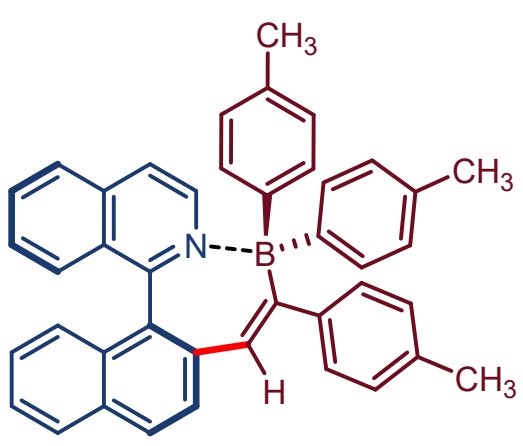

28

Prepared following **Procedure C**, using 1-(isoquinolin-1-yl)naphthalen-2-yl trifluoromethanesulfonate (80.6 mg, 0.2 mmol, 1.0 equiv.), tetramethylammonium ethynyltri-*p*-tolylborate (115.0 mg, 0.3 mmol,

1.5 equiv.), $Pd(acac)_2$ (1.5 mg, 2.5 mol%), and (*R*)-**L4** (11.0 mg, 7.5 mol%). Purification by flash column chromatography (Petroleum ether/EtOAc: 50/1) to afford the title compound (100.2 mg, 89%) as a yellow solid.

TLC: R_f = 0.5 (Petroleum ether/EtOAc: 50/1, $KMnO_4$ stain).

M. p.: 120 – 121 °C.

$[\alpha]^{18}_D$: +430.65 (c 0.20, CH_2Cl_2).

NMR Spectroscopy (*see spectra*):

^{1}H NMR (400 MHz, $CDCl_3$) δ_H = 8.73 (d, *J* = 6.9 Hz, 1H), 7.90 (d, *J* = 8.3 Hz, 1H), 7.71 (t, *J* = 7.6 Hz, 2H), 7.64 (d, *J* = 8.5 Hz, 1H), 7.59 (d, *J* = 8.1 Hz, 1H), 7.31 (d, *J* = 8.6 Hz, 1H), 7.29 – 7.24 (dt, *J* = 1.5, 15.9 Hz, 1H), 7.19 (t, *J* = 6.9 Hz, 1H), 7.15 (d, *J* = 8.9 Hz, 1H). 7.08 (s, 1H), 7.00 – 6.95 (m, 1H), 6.93 – 6.69 (m, 10H), 6.44 (d, *J* = 8.4 Hz, 1H), 6.30 (brs, 2H), 2.27 (s, 6H), 1.78 (s, 3H) ppm;

^{13}C NMR (100 MHz, $CDCl_3$) δ_C = 158.0, 148.7, 140.0, 138.7, 138.2, 136.3, 135.2, 134.5, 134.1, 132.8, 132.5, 131.3, 130.5, 130.2, 129.2, 128.4, 128.1, 127.6, 127.5, 127.3, 126.8, 126.5, 126.1, 125.5, 124.6, 121.1, 21.4, 21.2, 20.8 ppm. The carbon attached to boron was not observed due to quadrupolar relaxation;

^{11}B NMR (128 MHz, $CDCl_3$) δ_B = 3.83 ppm.

IR (film): ν_{max} 3677, 2919, 2861, 2345, 1750, 1505, 1316, 1201, 895, 822, 749 cm^{-1}.

HRMS (ESI): m/z calculated for $C_{42}H_{35}BN^+$ $[M+H]^+$, 564.2857, found, 564.2854.

HPLC analysis: HPLC conditions: Chiral column IB, *n*-hexane/isopropanol: 99/1, flow rate = 1.0 mL/min, wavelength = 254 nm, t_R = 8.602 min for major isomer, t_R = 7.780 min for minor isomer, 95% ee.

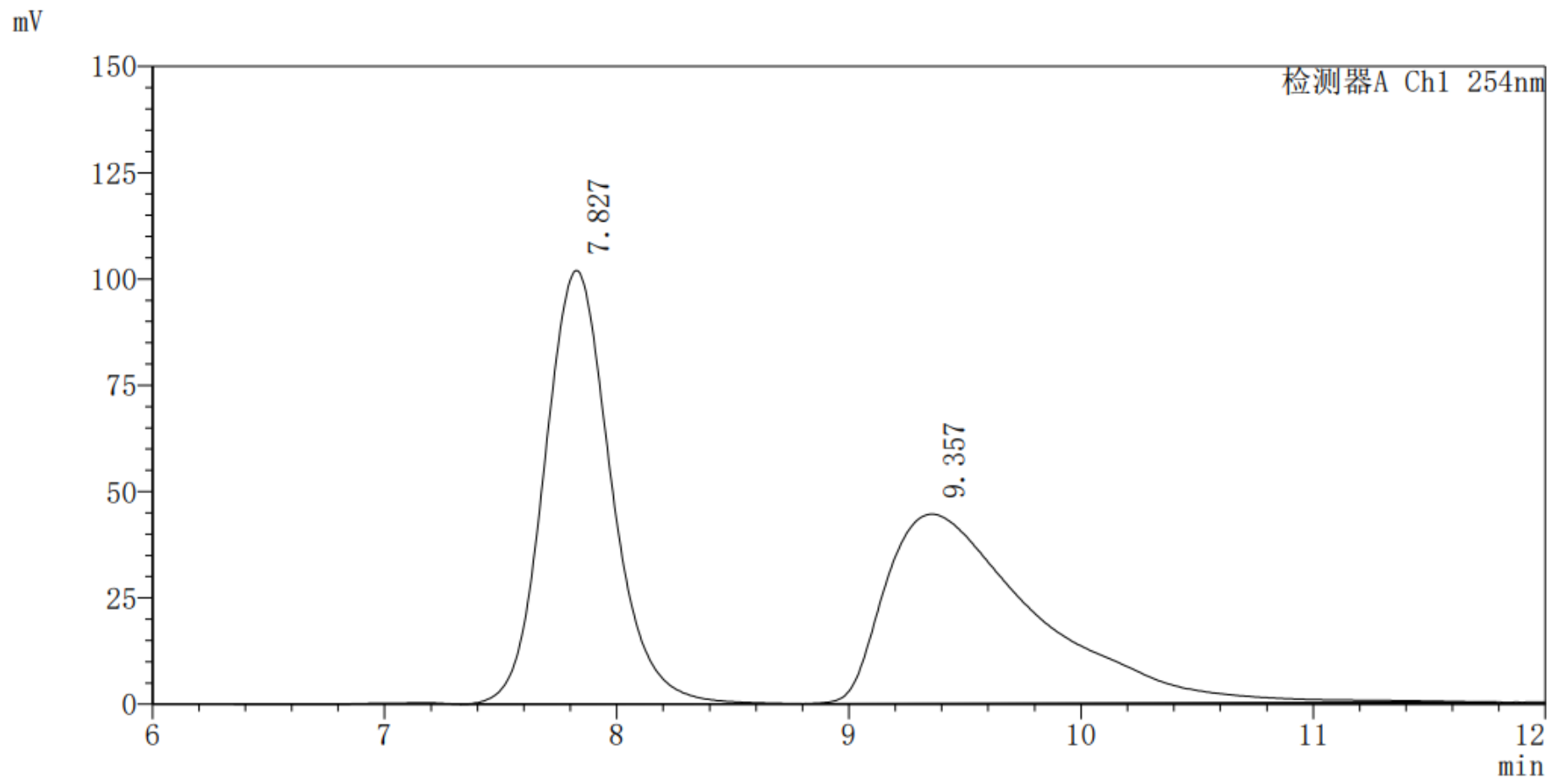

检测器A Ch1 254nm

No.	R.Time	Height	Height%	Area	Area%
1	7.827	101991	69.628	2038381	50.437
2	9.357	44490	30.372	2003071	49.563

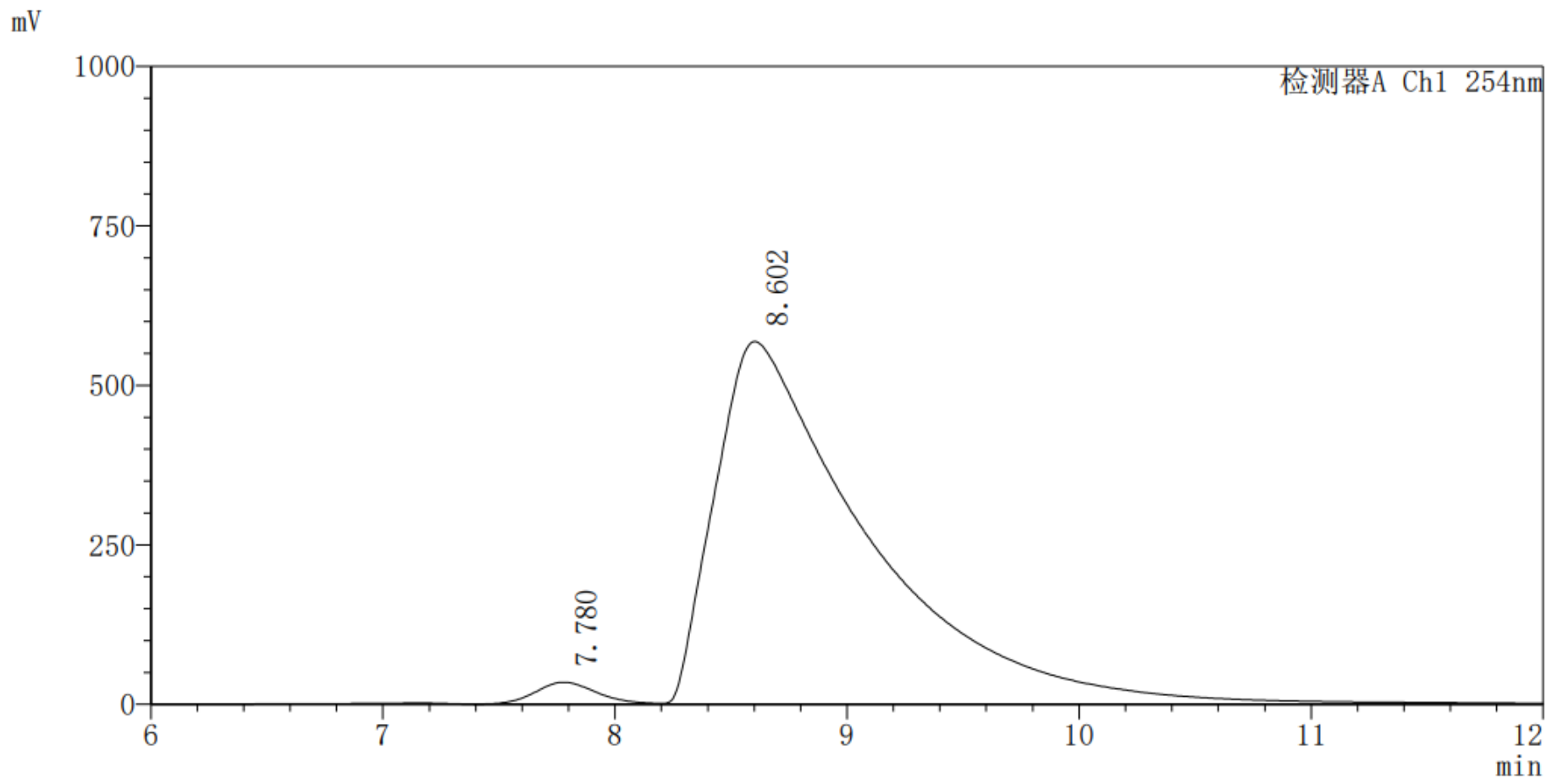

检测器A Ch1 254nm

No.	R.Time	Height	Height%	Area	Area%
1	7.780	34622	5.737	651227	2.411
2	8.602	568841	94.263	26359957	97.589

(*R*)-6-Butyl-4,4,5-triphenyl-4*H*-3l4,4l4-naphtho[2',1':5,6][1,2]azaborepino[7,1-a]isoquinoline (29)

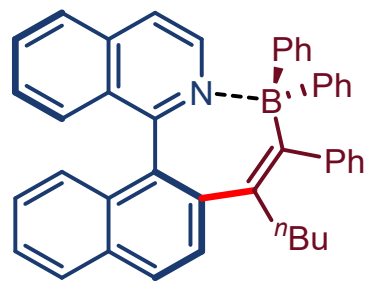

29

Prepared following **Procedure D**, using 1-(isoquinolin-1-yl)naphthalen-2-yl trifluoromethanesulfonate (80.6 mg, 0.2 mmol, 1.0 equiv.), tetramethylammonium hex-1-yn-1-yltriphenylborate (119.2 mg, 0.3 mmol, 1.5 equiv.), $Pd_2(dba)_3$ (9.16 mg, 0.001 mmol, 5.0 mol%) and (*R*)-**L1** (18.3 mg, 0.03 mmol, 15

mol%). Purification by flash column chromatography (Petroleum ether/EtOAc: 50/1) to afford the title compound (70.4 mg, 61%) as a yellow solid.

TLC: R_f = 0.5 (Petroleum ether/EtOAc: 50/1, $KMnO_4$ stain).

M. p.: 82 – 83 °C.

$[\alpha]^{18}_{D}$: +337.80 (c 0.20, CH_2Cl_2).

NMR Spectroscopy (*see spectra*):

^{1}H NMR (400 MHz, $CDCl_3$) δ_H = 8.91 (d, *J* = 6.9 Hz, 1H), 7.89 (d, *J* = 8.3 Hz, 1H), 7.72 (d, *J* = 6.9 Hz, 1H), 7.68 (t, *J* = 7.5 Hz, 1H), 7.54 – 7.46 (m, 3H), 7.36 (d, *J* = 8.5 Hz, 1H), 7.29 (d, *J* = 7.4 Hz, 1H), 7.13 (t, *J* = 7.5 Hz, 1H), 7.07 (d, *J* = 7.6 Hz, 1H), 7.02 (t, *J* = 7.4 Hz, 1H), 6.96 – 6.83 (m, 3H), 6.83 – 6.73 (m, 3H), 6.67 – 6.57 (m, 4H), 6.51 (t, *J* = 7.3 Hz, 2H), 6.32 (t, *J* = 8.9 Hz, 2H), 6.11 (t, *J* = 7.3 Hz, 1H), 2.63 – 2.52 (m, 1H), 2.43 – 2.31 (m, 1H), 0.97 – 0.85 (m, 2H), 0.81 – 0.75 (m, 2H), 0.41 (t, *J* = 7.2 Hz, 3H) ppm;

^{13}C NMR (100 MHz, $CDCl_3$) δ_C = 158.6, 149.5, 143.6, 139.8, 138.1, 138.0, 136.3, 132.9, 132.6, 131.7, 131.0, 130.8, 130.7, 130.5, 129.6, 129.5, 129.4, 128.7, 128.0, 127.7, 127.2, 127.0, 126.6, 126.5, 126.0, 125.9, 125.8, 124.9, 124.8, 124.5, 124.1, 123.9, 122.8, 120.9, 32.5, 32.4, 22.4, 14.0 ppm. The carbon attached to boron was not observed due to quadrupolar relaxation;

^{11}B NMR (128 MHz, $CDCl_3$) δ_B = 3.76 ppm.

IR (film): ν_{max} 3686, 2954, 2912, 2779, 1731, 1504, 1302, 1008, 822, 749, 702 cm^{-1}.

HRMS (ESI): m/z calculated for $C_{43}H_{37}BN^+$ $[M+H]^+$, 578.3014, found, 578.3018.

HPLC analysis: HPLC conditions: Chiral column IB, *n*-hexane/isopropanol: 99/1, flow rate = 1.0 mL/min, wavelength = 254 nm, t_R = 6.846 min for major isomer, t_R = 8.370 min for minor isomer, 94% ee.

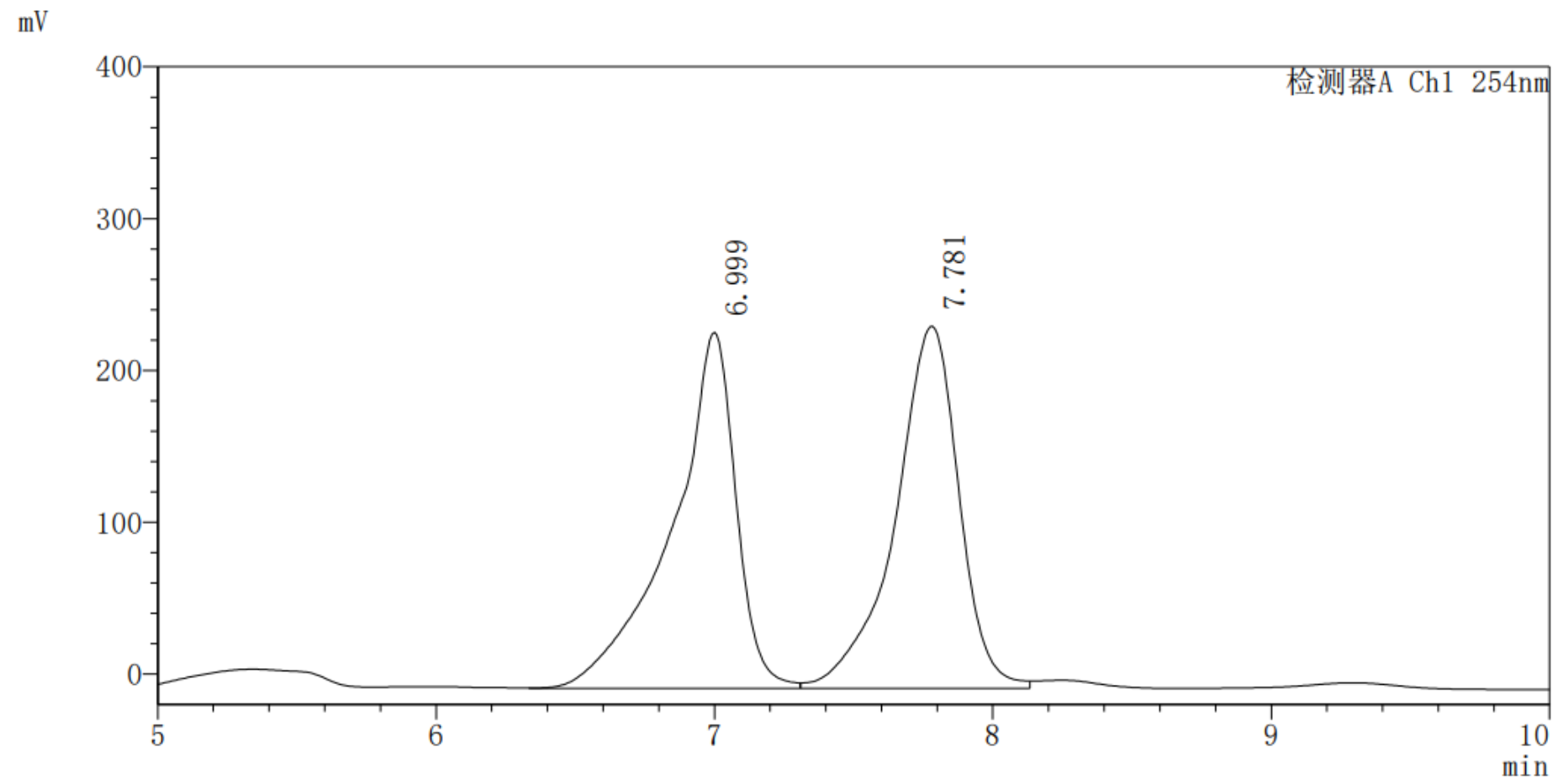

检测器A Ch1 254nm

No.	R.Time	Height	Height%	Area	Area%
1	6.999	234534	49.570	3754617	49.730
2	7.781	238602	50.430	3795404	50.270

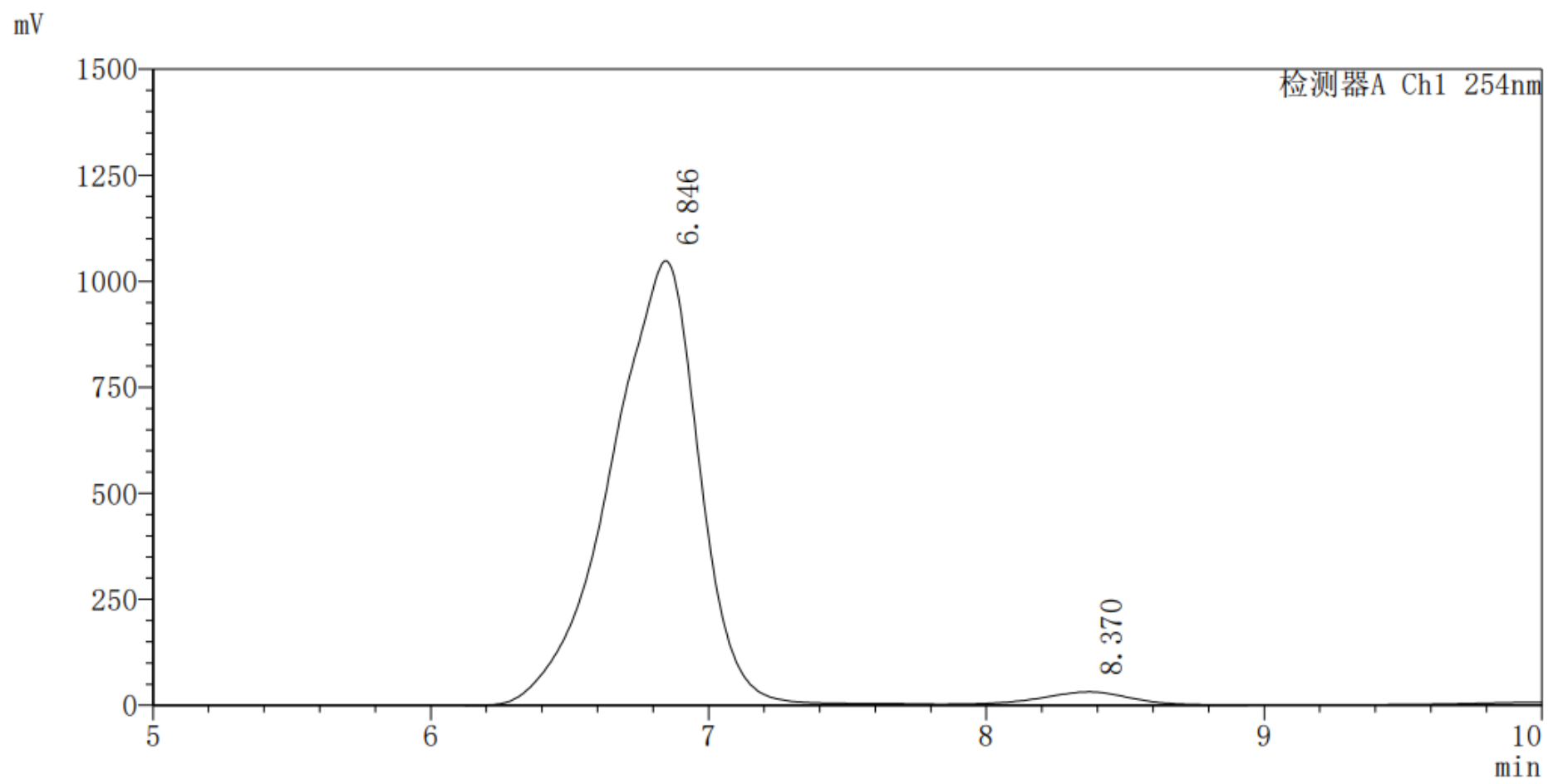

检测器A Ch1 254nm

No.	R.Time	Height	Height%	Area	Area%
1	6.846	1047999	97.062	23264822	96.817
2	8.370	31718	2.938	764932	3.183

(*R*)-6-Pentyl-4,4,5-triphenyl-4*H*-3l4,4l4-naphtho[2',1':5,6][1,2]azaborepino[7,1-a]isoquinoline (30)

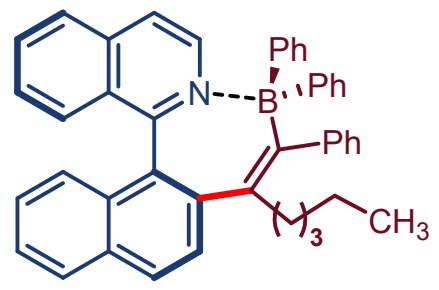

30

Prepared following **Procedure D**, using 1-(isoquinolin-1-yl)naphthalen-2-yl trifluoromethanesulfonate (80.6 mg, 0.2 mmol, 1.0 equiv.), tetramethylammonium hept-1-yn-1-yltriphenylborate (123.4 mg, 0.3 mmol, 1.5 equiv.), $Pd_2(dba)_3$ (9.16 mg, 0.001 mmol, 5.0 mol%) and (*R*)-**L1** (18.3 mg, 0.03 mmol, 15

mol%). Purification by flash column chromatography (Petroleum ether/EtOAc: 50/1) to afford the title compound (76.9 mg, 65%) as a yellow solid.

TLC: R_f = 0.5 (Petroleum ether/EtOAc: 50/1, $KMnO_4$ stain).

M. p.: 99 – 100 °C.

$[\alpha]^{18}_D$: +585.97 (c 0.20, CH_2Cl_2).

NMR Spectroscopy (*see spectra*):

> **^{1}H NMR** (500 MHz, $CDCl_3$) δ_H = 9.05 (dd, *J* = 3.8, 6.9 Hz, 1H), 8.02 (d, *J* = 8.2 Hz, 1H), 7.86 (d, *J* = 6.9 Hz, 1H), 7.81 (t, *J* = 7.6 Hz, 1H), 7.66 – 7.60 (m, 3H), 7.48 (d, *J* = 5.2 Hz, 1H), 7.42 – 7.39 (m, 1H), 7.26 (t, *J* = 6.1 Hz, 1H), 7.23 – 7.13 (m, 2H), 7.07 – 7.01 (m, 2H), 6.97 – 6.88 (m, 4H), 6.81 – 6.69 (m, 4H), 6.64 (q, *J* = 7.3 Hz, 2H), 6.49 – 6.42 (m, 2H), 6.27 – 6.21 (m, 1H), 2.76 – 2.65 (m, 1H), 2.54 – 2.43 (m, 1H), 1.30 – 1.21 (m, 1H), 1.12 – 1.05 (m, 1H), 0.98 – 0.87 (m, 3H), 0.82 – 0.77 (m, 1H), 0.51 (td, *J* = 3.5, 7.1 Hz, 3H) ppm;
>
> **^{13}C NMR** (125 MHz, $CDCl_3$) δ_C = 158.5, 149.5, 143.6, 139.8, 138.1, 138.0, 136.3, 132.9, 132.5, 131.7, 131.0, 130.8, 130.7, 130.5, 129.5, 129.3, 128.7, 128.1, 127.7, 127.2, 127.0, 126.6, 126.5, 126.0, 125.8, 124.9, 124.8, 124.4, 124.1, 123.9, 122.8, 120.9, 32.5, 31.5, 29.8, 22.6, 13.9 ppm. The carbon attached to boron was not observed due to quadrupolar relaxation;
>
> **^{11}B NMR** (128 MHz, $CDCl_3$) δ_B = 3.65 ppm.

IR (film): ν_{max} 3441, 2923, 1624, 1598, 1328, 1164, 822, 742, 702 cm^{-1}.

HRMS (ESI): m/z calculated for $C_{44}H_{38}BNNa^+$ $[M+Na]^+$, 592.3170, found, 592.3172.

HPLC analysis: HPLC conditions: Chiral column IB, *n*-hexane/isopropanol: 99/1, flow rate = 1.0 mL/min, wavelength = 254 nm, t_R = 6.587 min for major isomer, t_R = 8.041 min for minor isomer, 94% ee.

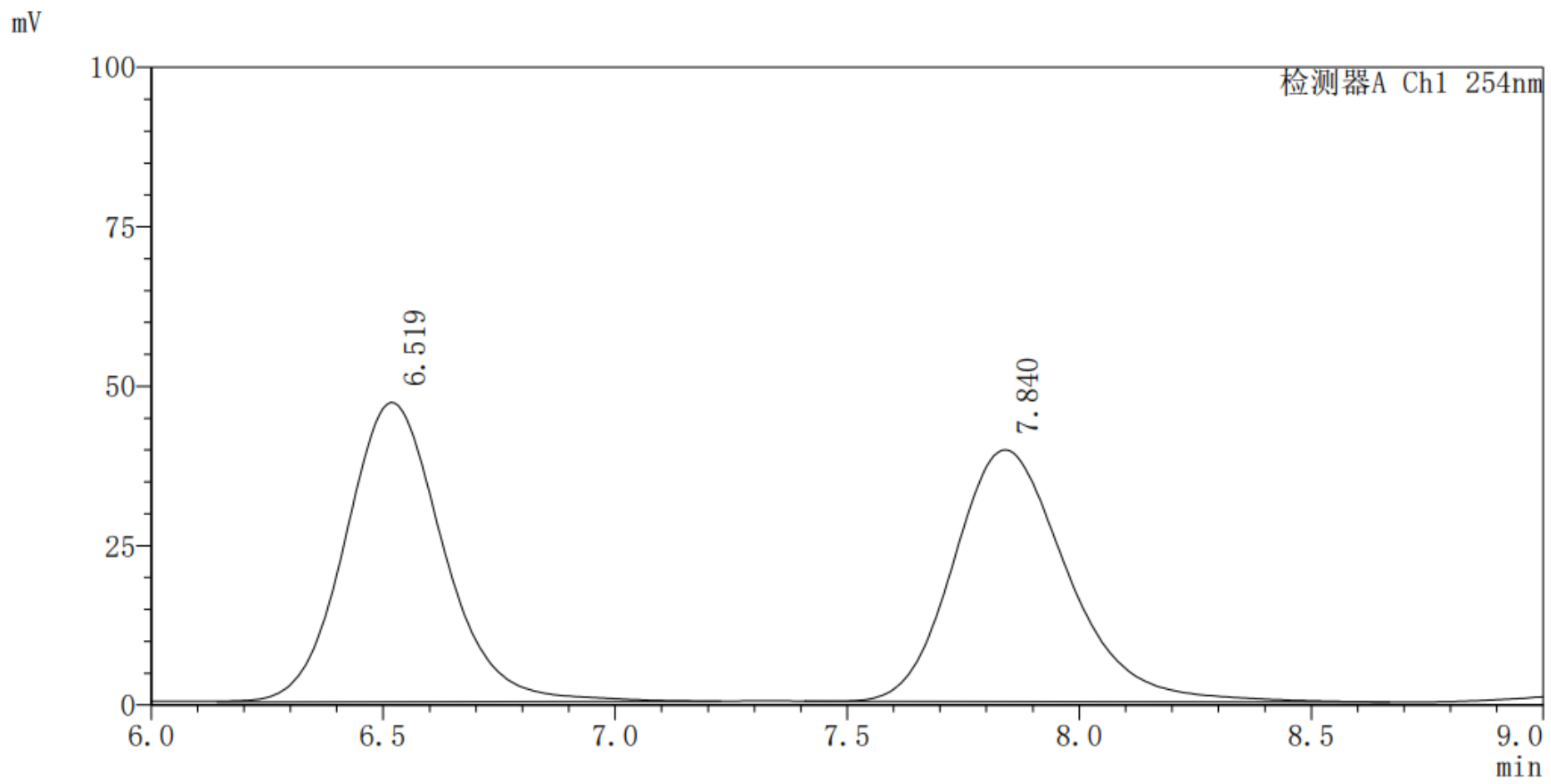

检测器A Ch1 254nm

No.	R.Time	Height	Height%	Area	Area%
1	6.519	46959	54.319	689820	50.494
2	7.840	39492	45.681	676316	49.506

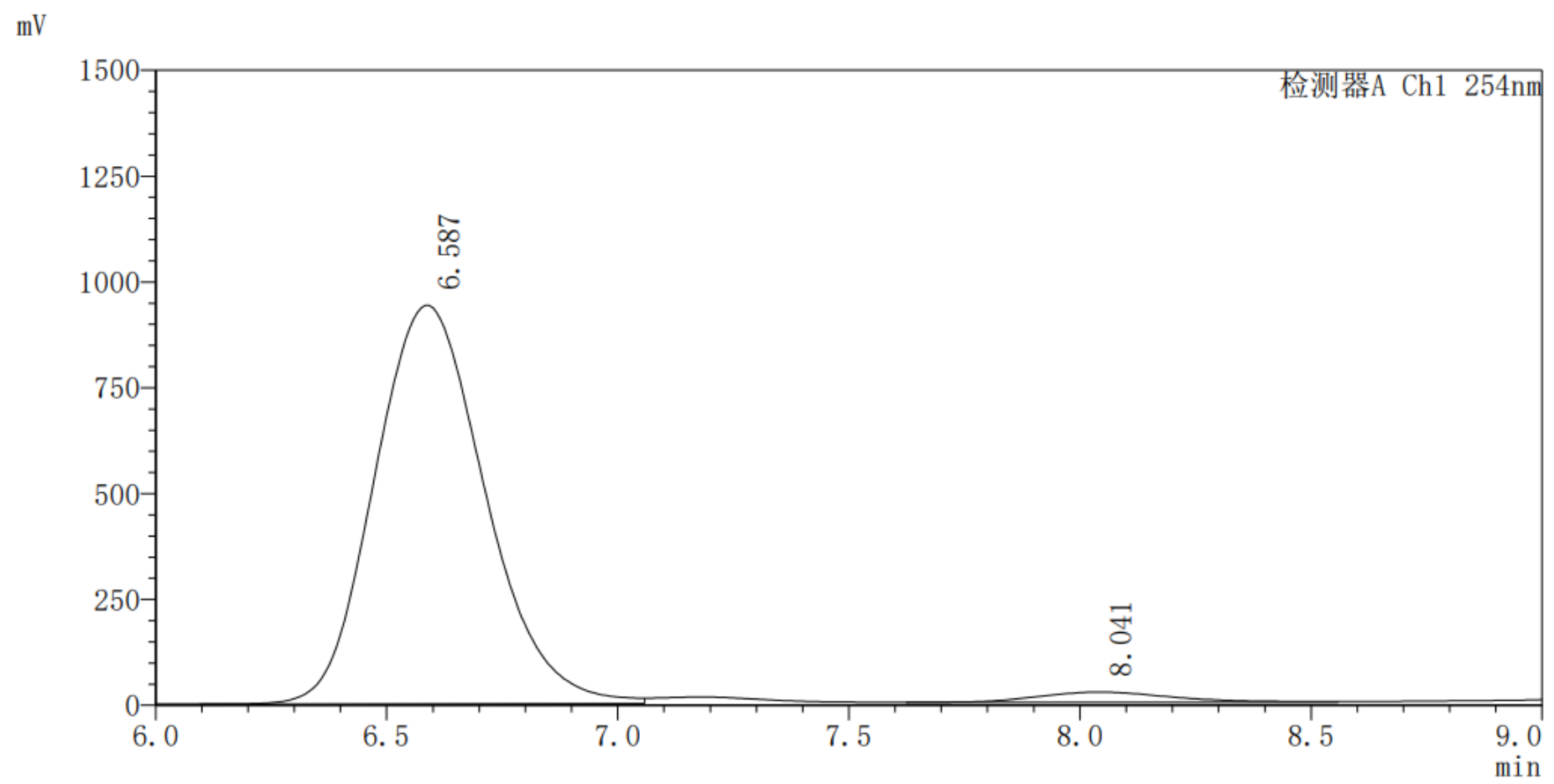

检测器A Ch1 254nm

No.	R.Time	Height	Height%	Area	Area%
1	6.587	940810	97.523	15647373	97.007
2	8.041	23895	2.477	482727	2.993

(*R*)-4,4,5-Triphenyl-6-propyl-4*H*-3l4,4l4-naphtho[2',1':5,6][1,2]azaborepino[7,1-a]isoquinoline (31)

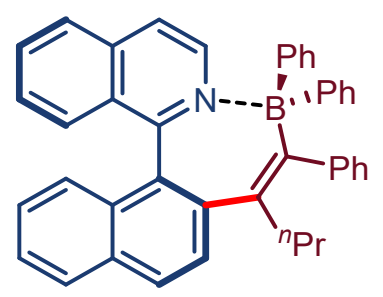

31

Prepared following **Procedure D**, using 1-(isoquinolin-1-yl)naphthalen-2-yl trifluoromethanesulfonate (80.6 mg, 0.2 mmol, 1.0 equiv.), tetramethylammonium pent-1-yn-1-yltriphenylborate (114.9 mg, 0.3

mmol, 1.5 equiv.), $Pd_2(dba)_3$ (9.16 mg, 0.001 mmol, 5.0 mol%) and (*R*)-**L1** (18.3 mg, 0.03 mmol, 15 mol%). Purification by flash column chromatography (Petroleum ether/EtOAc: 50/1) to afford the title compound (81.1 mg, 72%) as a yellow solid.

TLC: R_f = 0.5 (Petroleum ether/EtOAc: 50/1, $KMnO_4$ stain).

M. p.: 145 – 146 °C.

$[\alpha]^{18}_D$: +394.44 (c 0.20, CH_2Cl_2).

NMR Spectroscopy (*see spectra*):

^{1}H NMR (500 MHz, $CDCl_3$) δ_H = 9.05 (d, *J* = 6.7 Hz, 1H), 8.03 (d, *J* = 8.2 Hz, 1H), 7.86 (d, *J* = 6.9 Hz, 1H), 7.83 (d, *J* = 7.9 Hz, 1H), 7.71 – 7.61 (m, 3H), 7.50 (d, *J* = 8.7 Hz, 1H), 7.42 (t, *J* = 7.7 Hz, 1H), 7.28 (d, *J* = 7.6 Hz, 1H), 7.22 (d, *J* = 7.6 Hz, 1H), 7.17 (t, *J* = 7.5 Hz, 1H), 7.10 – 7.01 (m, 2H), 6.97 (q, *J* = 7.4 Hz, 2H), 6.91 (t, *J* = 7.2 Hz, 2H), 6.82 – 6.72 (m, 4H), 6.81 – 6.71 (m, 2H), 6.49 – 6.41 (m, 2H), 6.26 (t, *J* = 7.3 Hz, 1H), 2.77 – 2.65 (m, 1H), 2.52 – 2.43 (m, 1H), 1.33 – 1.25 (m, 1H), 1.18 – 1.06 (m, 1H), 0.57 (t, *J* = 7.3 Hz, 3H) ppm;

^{13}C NMR (125 MHz, $CDCl_3$) δ_C = 158.6, 149.6, 143.6, 139.7, 138.1, 138.1, 136.3, 132.9, 132.6, 131.7, 131.0, 130.8, 130.7, 130.5, 129.6, 129.5, 129.3, 128.7, 128.0, 127.7, 127.2, 127.0, 126.6, 126.5, 126.0, 125.9, 125.8, 124.9, 124.8, 124.5, 124.1, 123.9, 122.9, 120.8, 34.7, 23.3, 13.9 ppm. The carbon attached to boron was not observed due to quadrupolar relaxation;

^{11}B NMR (160 MHz, $CDCl_3$) δ_B = 3.79 ppm.

IR (film): ν_{max} 3705, 2955, 2026, 1597, 1461, 1365, 1161, 823, 738, 702 cm^{-1}.

HRMS (ESI): m/z calculated for $C_{42}H_{35}BN^+$ $[M+H]^+$, 564.2857, found, 564.2848.

HPLC analysis: HPLC conditions: Chiral column IB, *n*-hexane/isopropanol: 99/1, flow rate = 1.0 mL/min, wavelength = 254 nm, t_R = 7.095 min for major isomer, t_R = 8.715 min for minor isomer, 93% ee.

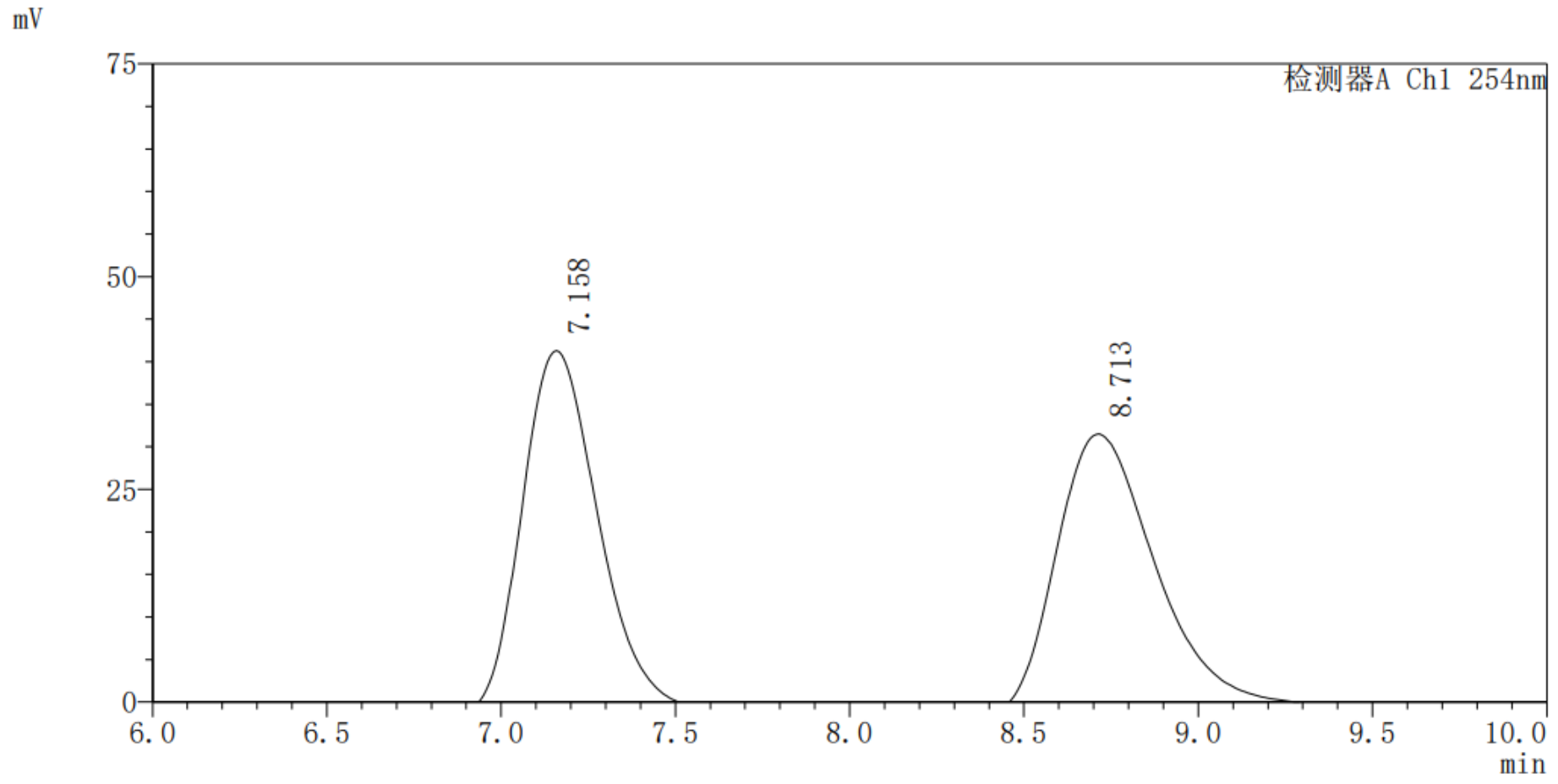

检测器A Ch1 254nm

No.	R.Time	Height	Height%	Area	Area%
1	7.158	42819	56.516	676803	49.531
2	8.713	32946	43.484	689619	50.469

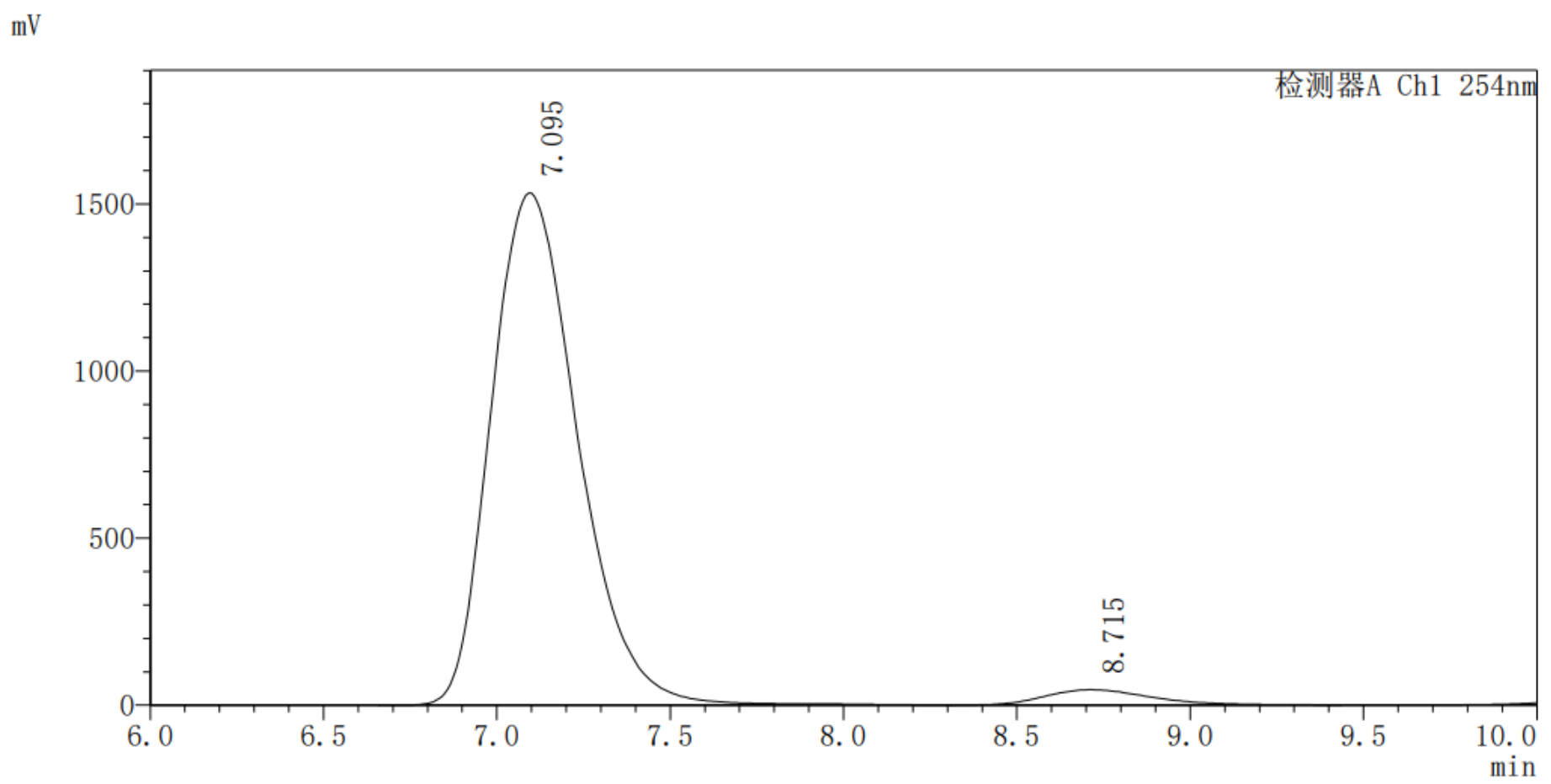

检测器A Ch1 254nm

No.	R.Time	Height	Height%	Area	Area%
1	7.095	1533229	97.088	26682897	96.443
2	8.715	45994	2.912	984151	3.557

(*R*)-6-Cyclopropyl-4,4,5-triphenyl-4*H*-3l4,4l4-naphtho[2',1':5,6][1,2]azaborepino[7,1-a] isoquinoline (32)

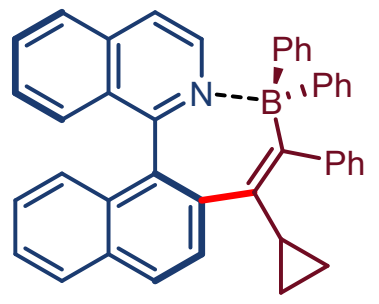

32

Prepared following **Procedure D**, using 1-(isoquinolin-1-yl)naphthalen-2-yl trifluoromethanesulfonate (80.6 mg, 0.2 mmol, 1.0 equiv.), tetramethylammonium (cyclopropylethynyl)triphenylborate (114.3 mg,

0.3 mmol, 1.5 equiv.), $Pd_2(dba)_3$ (9.16 mg, 0.001 mmol, 5.0 mol%) and (*R*)-**L1** (18.3 mg, 0.03 mmol, 15 mol%). Purification by flash column chromatography (Petroleum ether/EtOAc: 50/1) to afford the title compound (60.6 mg, 54%) as a yellow solid.

TLC: R_f = 0.5 (Petroleum ether/EtOAc: 50/1, $KMnO_4$ stain).

M. p.: 100 – 101 °C.

$[\alpha]^{18}_D$: +558.51 (c 0.20, CH_2Cl_2).

NMR Spectroscopy (*see spectra*):

^{1}**H NMR** (500 MHz, $CDCl_3$) δ_H = 8.98 (d, *J* = 6.9 Hz, 1H), 8.04 (d, *J* = 8.2 Hz, 1H), 7.85 (d, *J* = 6.7 Hz, 2H), 7.63-7.55 (m, 3H), 7.48 (d, *J* = 8.5 Hz, 1H), 7.45 – 7.41 (m, 1H), 7.25 (t, *J* = 7.5 Hz, 1H), 7.17-7.11 (m, 2H), 7.07 – 6.90 (m, 5H), 6.87 (t, *J* = 7.2 Hz, 2H), 6.74 (d, *J* = 6.9 Hz, 2H), 6.69 (t, *J* = 7.6 Hz, 1H), 6.65 (d, *J* = 7.5 Hz, 1H), 6.58 (d, *J* = 7.8 Hz, 1H), 6.40 (t, *J* = 7.3 Hz, 1H), 6.36 (d, *J* = 8.4 Hz, 1H), 6.23 (t, *J* = 7.4 Hz, 1H), 1.62 – 1.58 (m, 1H), 0.83 – 0.73 (m, 1H), 0.63 – 0.55 (m, 2H), -0.15 – -0.24 (m, 1H) ppm;

^{13}**C NMR** (100 MHz, $CDCl_3$) δ_C = 158.5, 149.3, 142.0, 140.5, 138.3, 138.1, 136.3, 133.0, 132.1, 131.9, 131.0, 130.7, 130.5, 130.4, 129.4, 129.4, 129.1, 128.8, 128.5, 127.8, 127.4, 127.1, 126.8, 126.7, 126.1, 126.0, 125.9, 125.8, 124.9, 124.8, 124.6, 123.8, 122.9, 120.9, 16.0, 11.6, 8.6 ppm. The carbon attached to boron was not observed due to quadrupolar relaxation;

^{11}**B NMR** (128 MHz, $CDCl_3$) δ_B = 4.02 ppm.

IR (film): ν_{max} 2996, 2832, 1630, 1597, 1364, 1135, 879, 748, 701 cm^{-1}.

HRMS (ESI): m/z calculated for $C_{42}H_{33}BN^+$ $[M+H]^+$, 562.2701, found, 562.2700.

HPLC analysis: HPLC conditions: Chiral column IB, *n*-hexane/isopropanol: 99/1, flow rate = 1.0 mL/min, wavelength = 254 nm, t_R = 7.602 min for major isomer, t_R = 8.729 min for minor isomer, 96% ee.

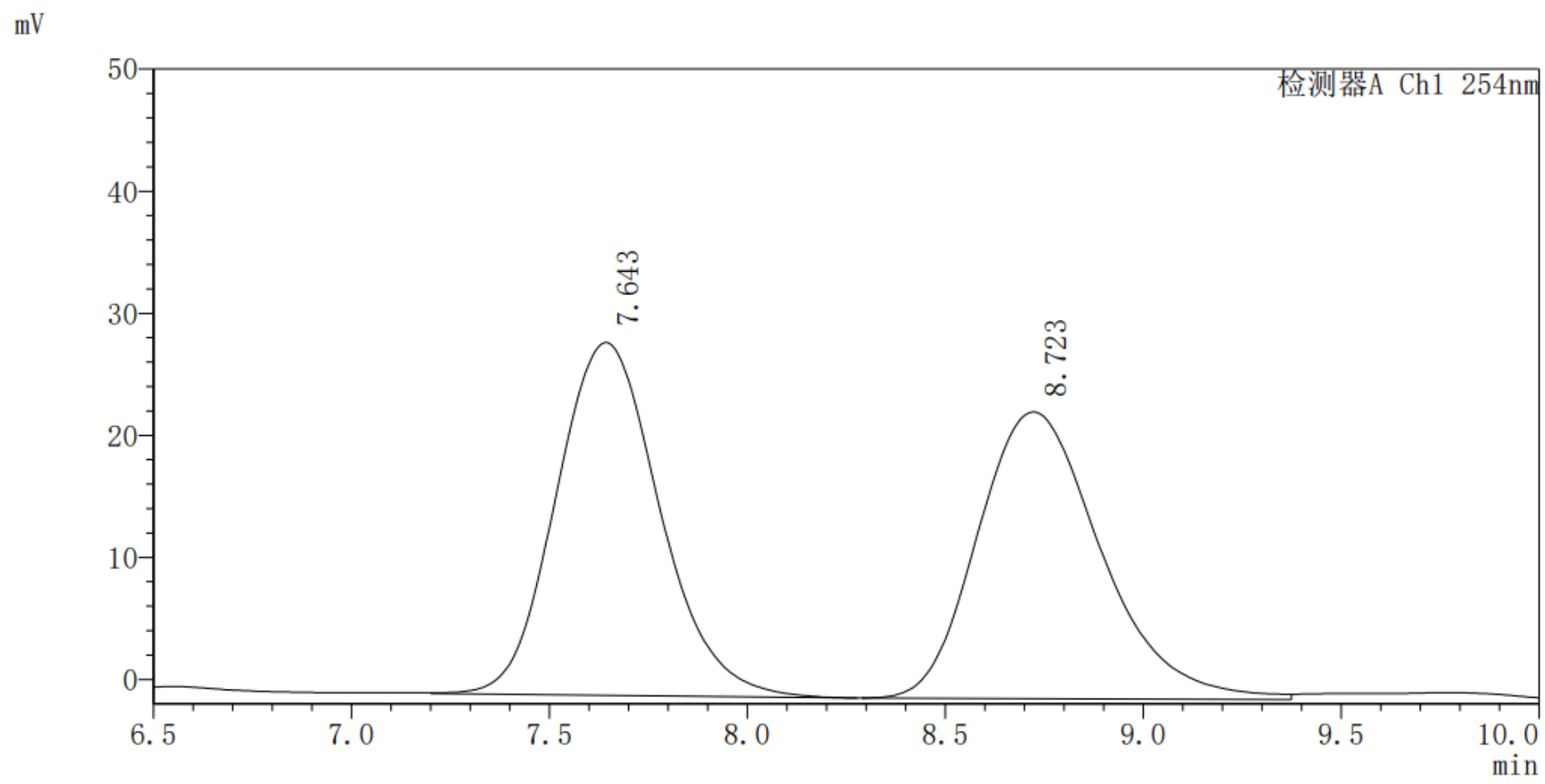

检测器A Ch1 254nm

No.	R.Time	Height	Height%	Area	Area%
1	7.643	28915	55.156	526433	50.825
2	8.723	23509	44.844	509335	49.175

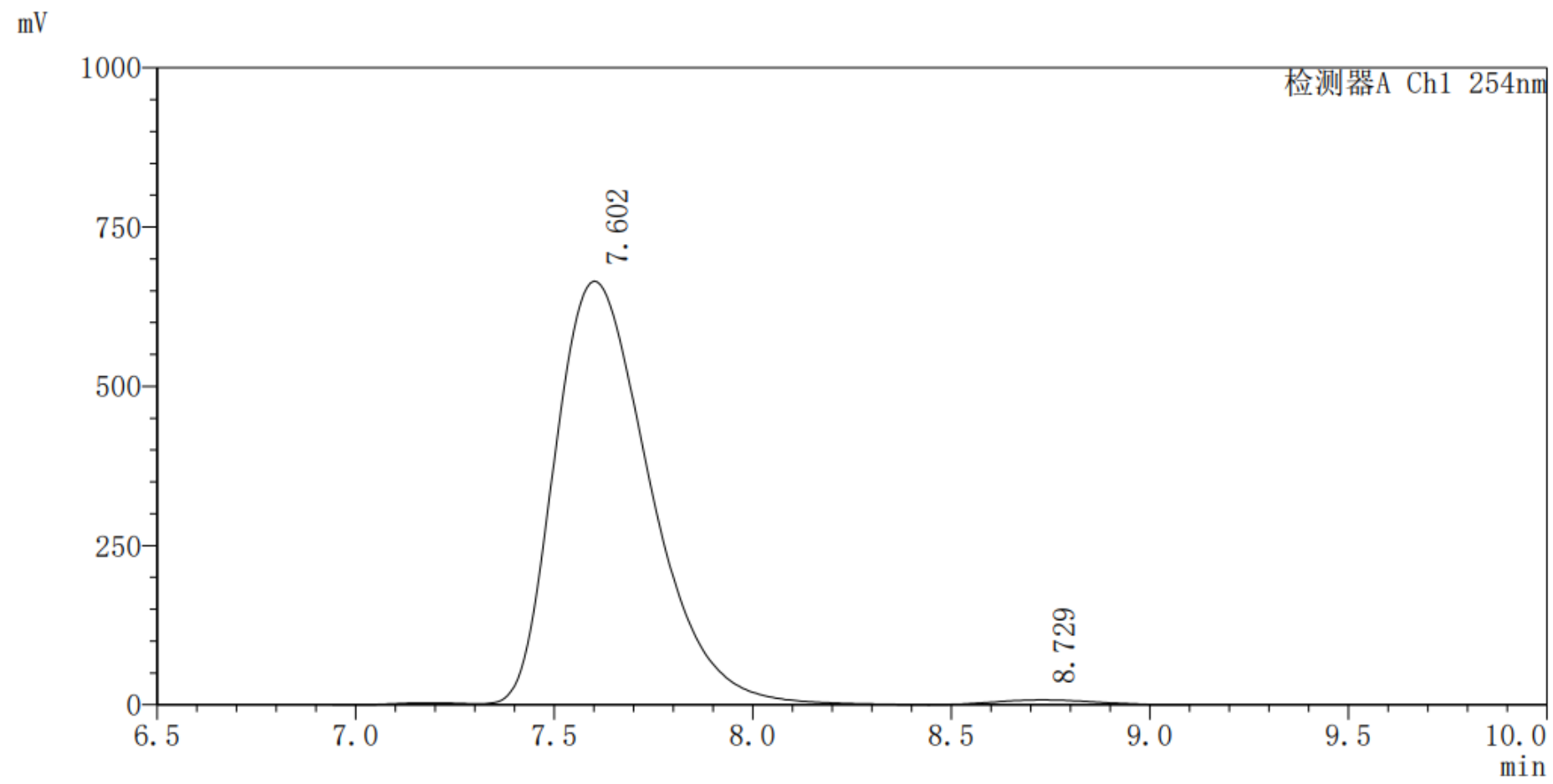

检测器A Ch1 254nm

No.	R.Time	Height	Height%	Area	Area%
1	7.602	666970	98.560	11223163	97.960
2	8.729	9747	1.440	233747	2.040

(*R*)-6-(Methoxymethyl)-4,4,5-triphenyl-4*H*-3l4,4l4-naphtho[2',1':5,6][1,2]azaborepino[7,1-a] isoquinoline (33)

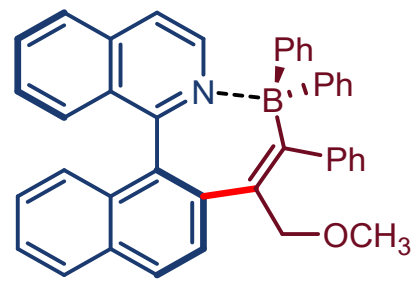

33

Prepared following **Procedure D**, using 1-(isoquinolin-1-yl)naphthalen-2-yl trifluoromethanesulfonate (80.6 mg, 0.2 mmol, 1.0 equiv.), tetramethylammonium (3-methoxyprop-1-yn-1-yl)triphenylborate

(115.5 mg, 0.3 mmol, 1.5 equiv.), $Pd_2(dba)_3$ (9.16 mg, 0.001 mmol, 5.0 mol%) and (*R*)-**L1** (18.3 mg, 0.03 mmol, 15 mol%). Purification by flash column chromatography (Petroleum ether/EtOAc: 50/1) to afford the title compound (110.7 mg, 98%) as a yellow solid.

TLC: R_f = 0.5 (Petroleum ether/EtOAc: 50/1, $KMnO_4$ stain).

M. p.: 89 – 90 °C.

$[\alpha]^{18}_D$: +410.68 (c 0.20, CH_2Cl_2).

NMR Spectroscopy (*see spectra*):

^{1}**H NMR** (400 MHz, $CDCl_3$) δ_H = 9.08 (d, *J* = 6.8 Hz, 1H), 8.01 (d, *J* = 8.3 Hz, 1H), 7.88 (d, *J* = 6.9 Hz, 1H), 7.81 (t, *J* = 7.6 Hz, 1H), 7.73 (q, *J* = 8.8 Hz, 2H), 7.65 (d, *J* = 7.9 Hz, 1H), 7.52 (d, *J* = 8.6 Hz, 1H), 7.39 (t, *J* = 7.8 Hz, 1H), 7.29 (d, *J* = 7.1 Hz, 1H), 7.24 – 7.15 (m, 2H), 7.06 (t, *J* = 7.8 Hz, 2H), 7.02 – 6.89 (m, 5H), 6.81 (d, *J* = 6.6 Hz, 2H), 6.78 – 6.68 (m, 2H), 6.64 (d, *J* = 7.6 Hz, 1H), 6.51 – 6.42 (m, 2H), 6.30 (t, *J* = 7.4 Hz, 1H), 4.70 (d, *J* = 12.0 Hz, 1H), 4.26 (d, *J* = 12.1 Hz, 1H), 3.02 (s, 3H) ppm;

^{13}**C NMR** (100 MHz, $CDCl_3$) δ_C = 158.4, 148.2, 142.2, 138.0, 136.5, 136.4, 133.0, 132.6, 131.8, 131.0, 130.9, 130.7, 130.6, 129.6, 129.5, 129.3, 128.8, 127.7, 127.3, 127.2, 127.0, 126.8, 126.6, 126.1, 125.9, 125.8, 125.1, 124.9, 124.6, 124.3, 124.1, 123.1, 121.1, 72.0, 57.3 ppm. The carbon attached to boron was not observed due to quadrupolar relaxation;

^{11}**B NMR** (128 MHz, $CDCl_3$) δ_B = 3.49 ppm.

IR (film): ν_{max} 3043, 2917, 1795, 1720, 1503, 1428, 1426, 1085, 823, 750, 701 cm^{-1}.

HRMS (ESI): m/z calculated for $C_{41}H_{33}BNO^+$ $[M+H]^+$, 566.2650, found, 566.2648.

HPLC analysis: HPLC conditions: Chiral column IB, *n*-hexane/isopropanol: 97/3, flow rate = 1.0 mL/min, wavelength = 254 nm, t_R = 7.471 min for major isomer, t_R = 9.443 min for minor isomer, 90% ee.

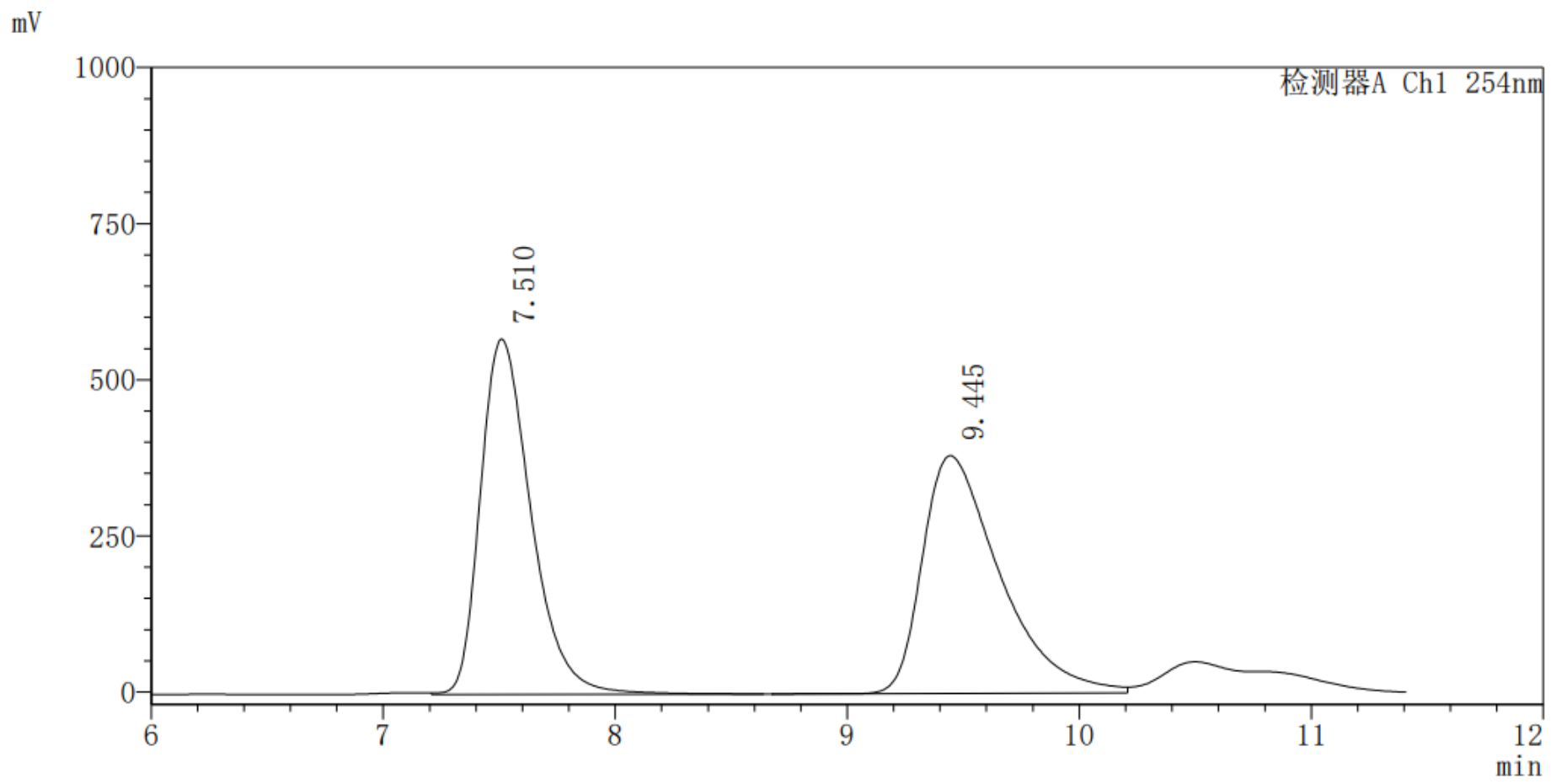

检测器A Ch1 254nm

No.	R.Time	Height	Height%	Area	Area%
1	7.510	569160	59.928	8635483	49.462
2	9.445	380579	40.072	8823354	50.538

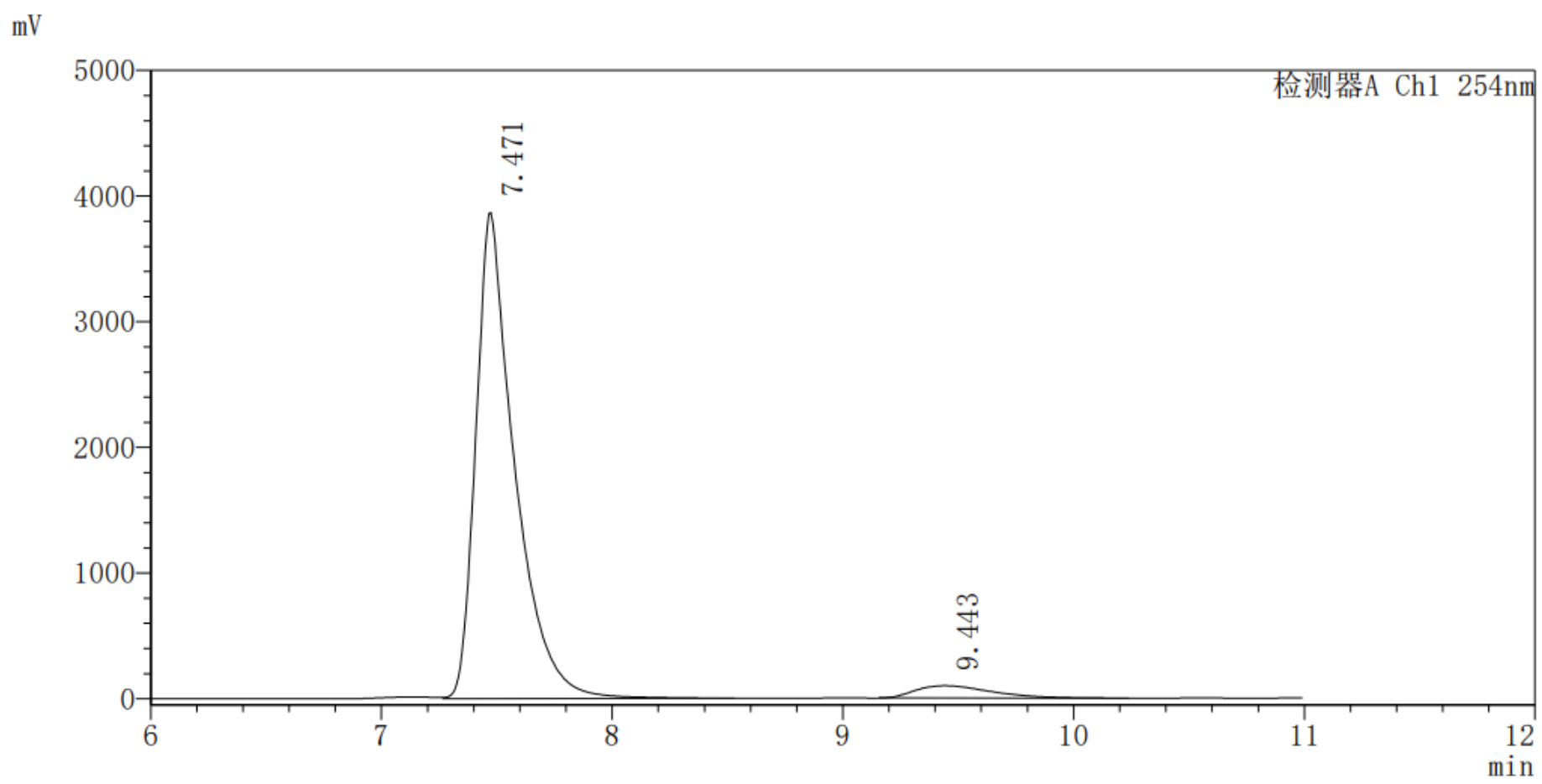

检测器A Ch1 254nm

No.	R.Time	Height	Height%	Area	Area%
1	7.471	3867572	97.527	45176005	95.195
2	9.443	98066	2.473	2280284	4.805

(*R*)-6-(2-Bromoethyl)-4,4,5-triphenyl-4*H*-3l4,4l4-naphtho[2',1':5,6][1,2]azaborepino[7,1-a] isoquinoline (34)

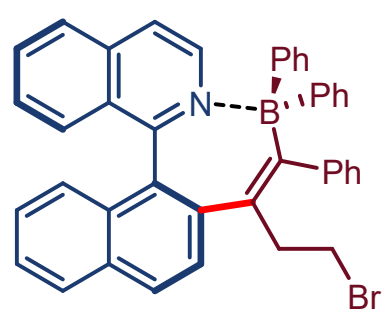

34

Prepared following **Procedure D**, using 1-(isoquinolin-1-yl)naphthalen-2-yl trifluoromethanesulfonate (80.6 mg, 0.2 mmol, 1.0 equiv.), tetramethylammonium (4-bromobut-1-yn-1-yl)triphenylborate (134.1

mg, 0.3 mmol, 1.5 equiv.), $Pd_2(dba)_3$ (9.16 mg, 0.001 mmol, 5.0 mol%) and (*R*)-**L1** (18.3 mg, 0.03 mmol, 15 mol%). Purification by flash column chromatography (Petroleum ether/EtOAc: 50/1) to afford the title compound (100.3 mg, 80%) as a yellow solid.

TLC: R_f = 0.5 (Petroleum ether/EtOAc: 50/1, $KMnO_4$ stain).

M. p.: 81 – 82 °C.

$[\alpha]^{18}_D$: +378.37 (c 0.20, CH_2Cl_2).

NMR Spectroscopy (*see spectra*):

> ^{1}**H NMR** (500 MHz, $CDCl_3$) δ_H = 9.03 (d, *J* = 6.9 Hz, 1H), 8.01 (d, *J* = 8.2 Hz, 1H), 7.86 (d, *J* = 6.9 Hz, 1H), 7.81 (ddd, *J* = 1.4, 6.8, 8.2 Hz, 1H), 7.67 (d, *J* = 8.5 Hz, 1H), 7.63 (d, *J* = 6.9 Hz, 1H), 7.56 (d, *J* = 8.7 Hz, 1H), 7.48 (d, *J* = 7.6 Hz, 1H), 7.43 – 7.39 (m, 1H), 7.30 – 7.27 (m, 1H), 7.19 (td, *J* = 7.5, 1.5 Hz, 1H), 7.15 (d, *J* = 7.6 Hz, 1H), 7.09 – 7.02 (m, 2H), 6.98 – 6.92 (m, 2H), 6.91 – 6.85 (m, 3H), 6.77 – 6.68 (m, 3H), 6.64 (d, *J* = 7.5 Hz, 1H), 6.60 (d, *J* = 7.6 Hz, 1H), 6.49 – 6.42 (m, 2H), 6.25 (t, *J* = 6.7 Hz, 1H), 3.32 – 3.24 (m, 2H), 3.24 – 3.17 (m, 1H), 3.15 – 3.06 (m, 1H) ppm;
>
> ^{13}**C NMR** (126 MHz, $CDCl_3$) δ_C = 158.2, 148.7, 141.8, 138.0, 136.3, 135.8, 133.0, 132.6, 131.8, 131.1, 130.9, 130.8, 130.6, 130.2, 129.9, 129.1, 128.7, 128.0, 127.7, 127.4, 127.1, 126.9, 126.9, 126.5, 126.1, 126.0, 125.9, 125.1, 125.0, 124.6, 124.3, 123.4, 123.0, 121.1, 35.5, 32.9 ppm. The carbon attached to boron was not observed due to quadrupolar relaxation;
>
> ^{11}**B NMR** (128 MHz, $CDCl_3$) δ_B = 3.83 ppm.

IR (film): ν_{max} 3693, 2997, 2759, 1776, 1503, 1264, 961, 823, 702 cm^{-1}.

HRMS (ESI): m/z calculated for $C_{41}H_{32}BBrN^+$ $[M+H]^+$, 628.1806, found, 628.1806.

HPLC analysis: HPLC conditions: Chiral column IB, *n*-hexane/isopropanol: 99/1, flow rate = 1.0 mL/min, wavelength = 254 nm, t_R = 9.186 min for major isomer, t_R = 11.515 min for minor isomer, 87% ee.

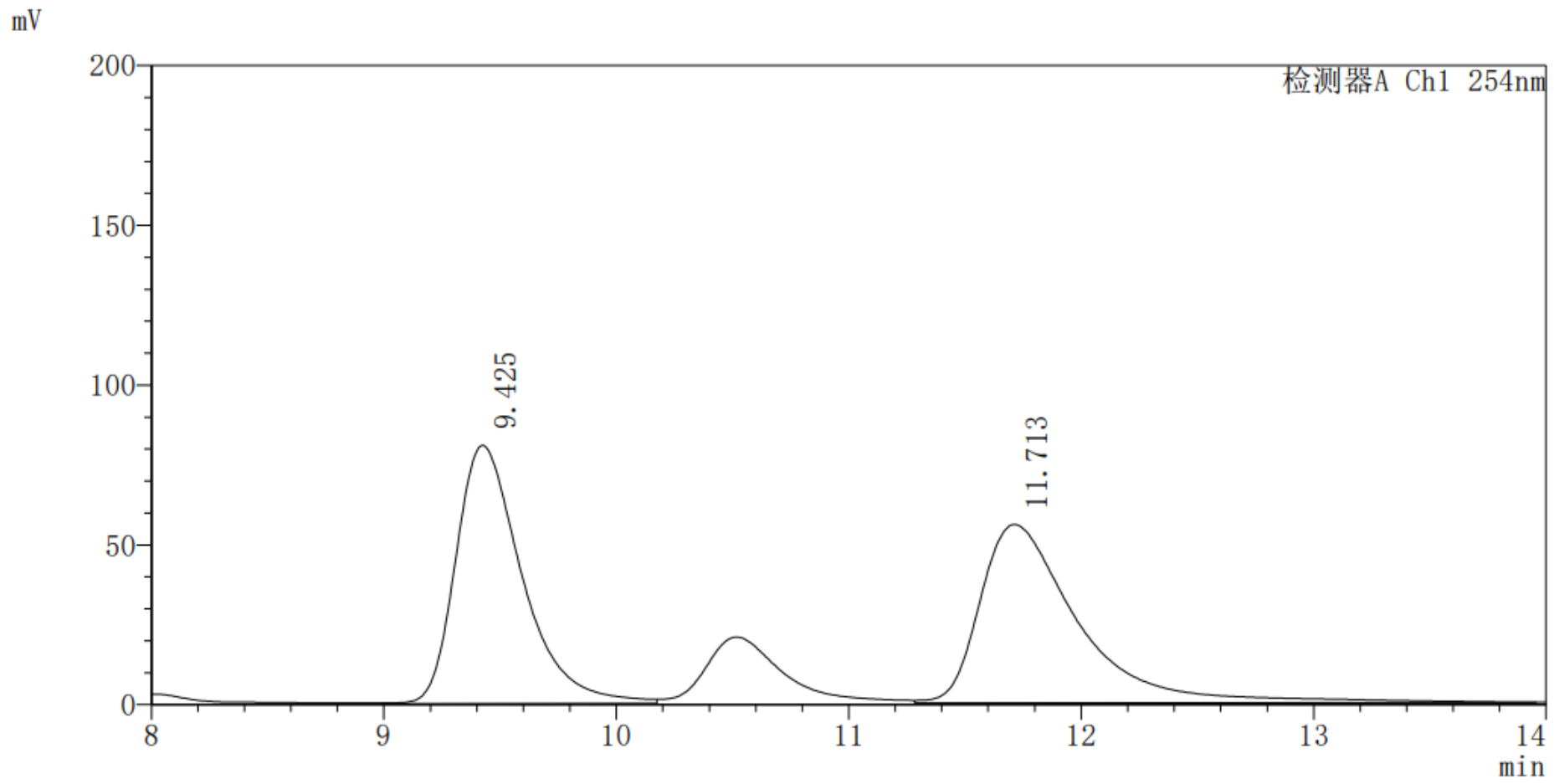

检测器A Ch1 254nm

No.	R.Time	Height	Height%	Area	Area%
1	9.425	80787	59.132	1602349	49.326
2	11.713	55834	40.868	1646161	50.674

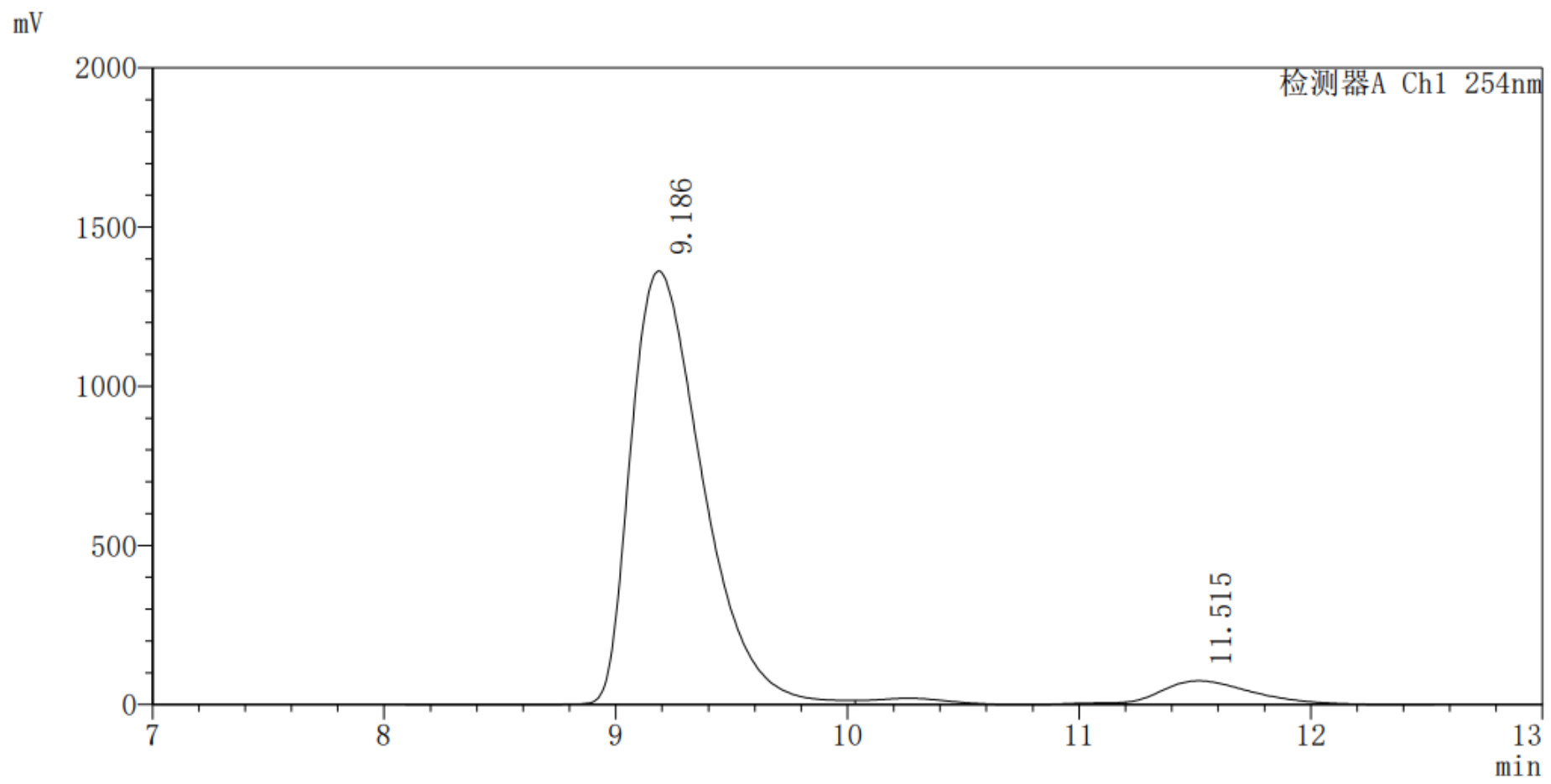

检测器A Ch1 254nm

No.	R.Time	Height	Height%	Area	Area%
1	9.186	1364729	94.741	29752914	93.258
2	11.515	75751	5.259	2150947	6.742

(*R*)-6-(3-Chloropropyl)-4,4,5-triphenyl-4*H*-3l4,4l4-naphtho[2',1':5,6][1,2]azaborepino[7,1-a]isoquinoline (35)

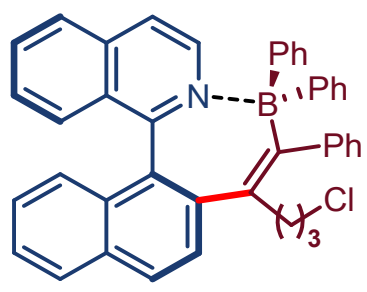

35

Prepared following **Procedure D**, using 1-(isoquinolin-1-yl)naphthalen-2-yl trifluoromethanesulfonate (80.6 mg, 0.2 mmol, 1.0 equiv.), tetramethylammonium (5-chloropent-1-yn-1-yl)triphenylborate (125.1

mg, 0.3 mmol, 1.5 equiv.), $Pd_2(dba)_3$ (9.16 mg, 0.001 mmol, 5.0 mol%) and (*R*)-**L1** (18.3 mg, 0.03 mmol, 15 mol%). Purification by flash column chromatography (Petroleum ether/EtOAc: 50/1) to afford the title compound (100.3 mg, 84%) as a yellow solid.

TLC: $R_f = 0.5$ (Petroleum ether/EtOAc: 50/1, $KMnO_4$ stain).

M. p.: 96 – 97 °C.

$[\alpha]^{18}_D$: +322.84 (c 0.20, CH_2Cl_2).

NMR Spectroscopy (*see spectra*):

^{1}H NMR (400 MHz, $CDCl_3$) δ_H = 9.03 (d, *J* = 6.7 Hz, 1H), 8.03 (d, *J* = 8.4 Hz, 1H), 7.87 (d, *J* = 6.7 Hz, 1H), 7.83 (t, *J* = 7.4 Hz, 1H), 7.70 – 7.61 (m, 3H), 7.48 – 7.43 (m, 2H), 7.28 (d, *J* = 7.2 Hz, 1H), 7.16 (t, *J* = 8.3 Hz, 2H), 7.08 – 7.01 (m, 2H), 6.97 (t, *J* = 7.5 Hz, 1H), 6.94 (d, *J* = 6.9 Hz, 1H), 6.89 (t, *J* = 7.7 Hz, 2H), 6.78-6.69 (m, 4H), 6.63 (t, *J* = 8.9 Hz, 2H), 6.49 – 6.41 (m, 2H), 6.24 (t, *J* = 7.4 Hz, 1H), 3.24 – 3.14 (m, 1H), 3.13 – 3.03 (m, 1H), 2.88 – 2.84 (m, 1H), 2.65 – 2.56 (m, 1H), 1.79 – 1.66 (m, 1H), 1.66 – 1.54 (m, 1H) ppm;

^{13}C NMR (125 MHz, $CDCl_3$) δ_C = 158.3, 149.2, 142.8, 138.1, 138.0, 137.9, 136.3, 133.1, 132.6, 131.7, 130.9, 130.5, 130.4, 129.9, 129.7, 129.2, 129.0, 128.0, 127.7, 127.6, 127.4, 127.0, 126.8, 126.7, 126.0, 125.9, 125.0, 124.6, 124.2, 123.7, 123.0, 121.0, 44.7, 33.3, 30.5 ppm. The carbon attached to boron was not observed due to quadrupolar relaxation;

^{11}B NMR (128 MHz, $CDCl_3$) δ_B = 3.65 ppm.

IR (film): ν_{max} 3692, 3059, 2026, 1803, 1630, 1487, 1263, 911, 823, 703 cm^{-1}.

HRMS (ESI): m/z calculated for $C_{42}H_{34}BClN^+$ $[M+H]^+$, 598.2467, found, 598.2461.

HPLC analysis: HPLC conditions: Chiral column IB, *n*-hexane/isopropanol: 99/1, flow rate = 1.0 mL/min, wavelength = 254 nm, t_R = 8.944 min for major isomer, t_R = 11.083 min for minor isomer, 92% ee.

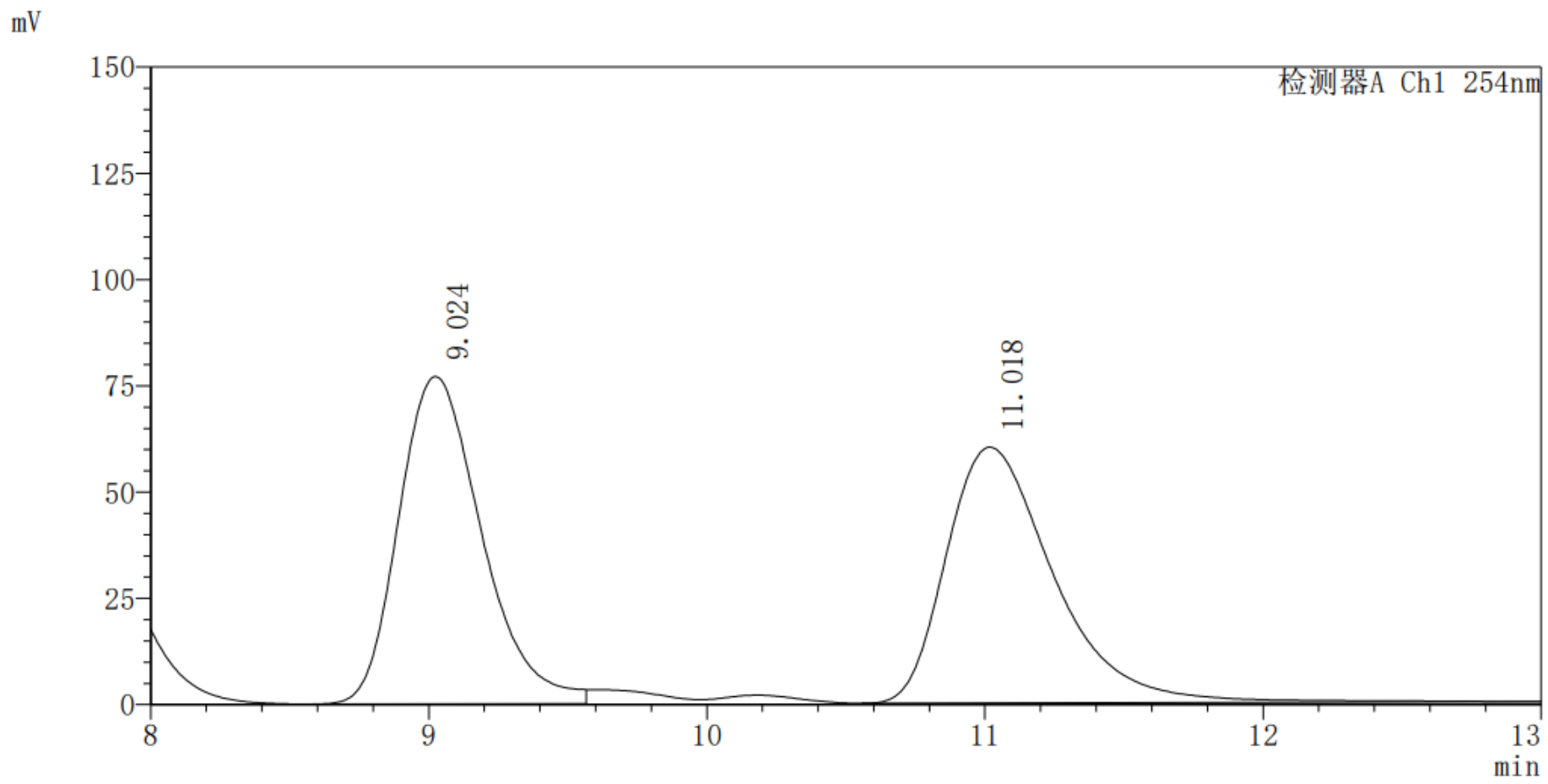

检测器A Ch1 254nm

No.	R.Time	Height	Height%	Area	Area%
1	9.024	77092	56.135	1599052	49.575
2	11.018	60242	43.865	1626487	50.425

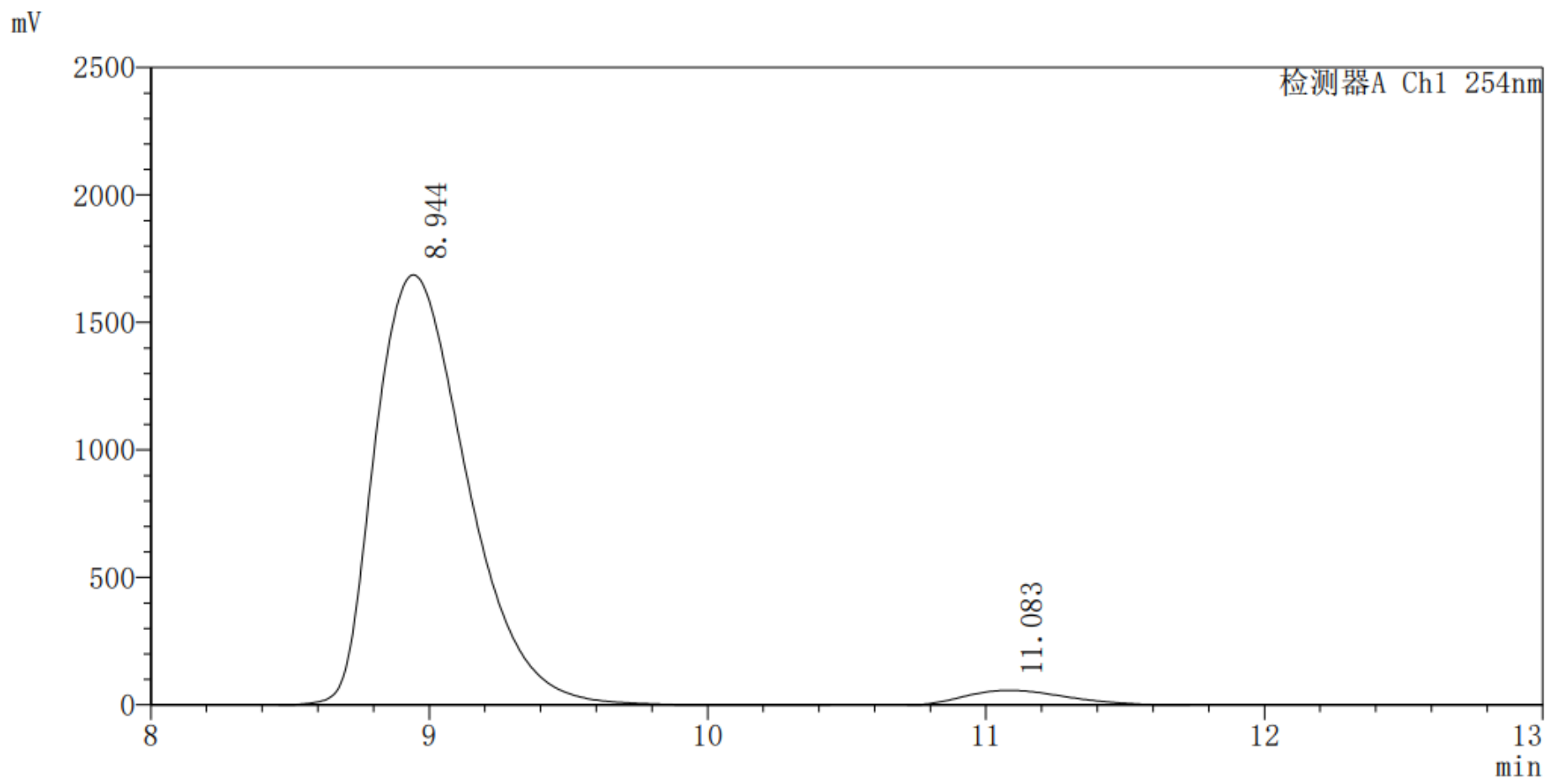

检测器A Ch1 254nm

No.	R.Time	Height	Height%	Area	Area%
1	8.944	1691537	96.526	39245753	95.951
2	11.083	60879	3.474	1656305	4.049

(*R*)-6-(3-Phenoxypropyl)-4,4,5-triphenyl-4*H*-3l4,4l4-naphtho[2',1':5,6][1,2]azaborepino[7,1-a]isoquinoline (36)

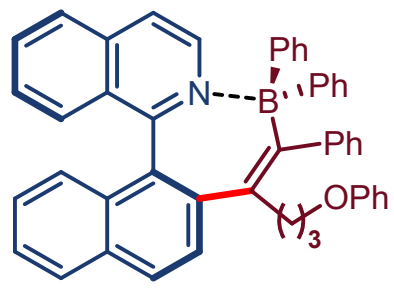

36

Prepared following **Procedure D**, using 1-(isoquinolin-1-yl)naphthalen-2-yl trifluoromethanesulfonate (80.6 mg, 0.2 mmol, 1.0 equiv.), tetramethylammonium (5-phenoxypent-1-yn-1-yl)triphenylborate

(142.5 mg, 0.3 mmol, 1.5 equiv.), $Pd_2(dba)_3$ (9.16 mg, 0.001 mmol, 5.0 mol%) and (*R*)-**L1** (18.3 mg, 0.03 mmol, 15 mol%). Purification by flash column chromatography (Petroleum ether/EtOAc: 50/1) to afford the title compound (111.3 mg, 85%) as a yellow solid.

TLC: R_f = 0.5 (Petroleum ether/EtOAc: 50/1, $KMnO_4$ stain).

M. p.: 65 – 66 °C.

$[\alpha]^{18}_D$: +348.14 (c 0.20, CH_2Cl_2).

NMR Spectroscopy (*see spectra*):

^{1}**H NMR** (400 MHz, $CDCl_3$) δ_H = 9.03 (d, *J* = 6.9 Hz, 1H), 7.97 (d, *J* = 8.3 Hz, 1H), 7.87 – 7.82 (m, 2H), 7.72 (t, *J* = 7.6 Hz, 1H), 7.69-7.61 (m, 3H), 7.50 (d, *J* = 7.5 Hz, 1H), 7.47 – 7.42 (m, 2H), 7.29 (d, *J* = 6.6 Hz, 2H), 7.20 (d, *J* = 7.5 Hz, 1H), 7.17 – 7.12 (m, 3H), 7.08 – 7.01 (m, 2H), 6.97 – 6.87 (m, 4H), 6.76 (d, *J* = 6.9 Hz, 2H), 6.72 – 6.60 (m, 3H), 6.49 – 6.42 (m, 3H), 6.25 (t, *J* = 7.3 Hz, 1H), 3.48 (q, *J* = 7.3 Hz, 1H), 3.39 (q, *J* = 8.3 Hz, 1H), 2.92 – 2.81 (m, 1H), 2.79 – 2.67 (m, 1H), 1.84 – 1.74 (m, 1H), 1.70 – 1.61 (m, 1H) ppm;

^{13}**C NMR** (100 MHz, $CDCl_3$) δ_C = 158.7, 158.3, 149.3, 143.6, 142.9, 138.2, 138.1, 138.0, 136.3, 134.8, 133.0, 132.6, 131.7, 131.1, 130.9, 130.8, 130.7, 130.4, 130.3, 129.8, 129.7, 129.7, 129.2, 129.2, 129.1, 128.9, 128.5, 128.0, 127.8, 127.7, 127.3, 127.0, 126.7, 126.5, 126.0, 125.9, 125.5, 124.9, 124.5, 124.1, 123.9, 122.9, 121.0, 120.2, 114.0, 66.8, 29.2, 28.7 ppm. The carbon attached to boron was not observed due to quadrupolar relaxation;

^{11}**B NMR** (128 MHz, $CDCl_3$) δ_B = 3.24 ppm.

IR (film): ν_{max} 3675, 3037, 2872, 2778, 1717, 1498, 1302, 1245, 999, 826, 750, 702 cm^{-1}.

HRMS (ESI): m/z calculated for $C_{48}H_{39}BNO^+$ $[M+H]^+$, 656.3119, found, 656.3128.

HPLC analysis: HPLC conditions: Chiral column IB, *n*-hexane/isopropanol: 99/1, flow rate = 1.0 mL/min, wavelength = 254 nm, t_R = 11.165 min for major isomer, t_R = 14.637 min for minor isomer, 93% ee.

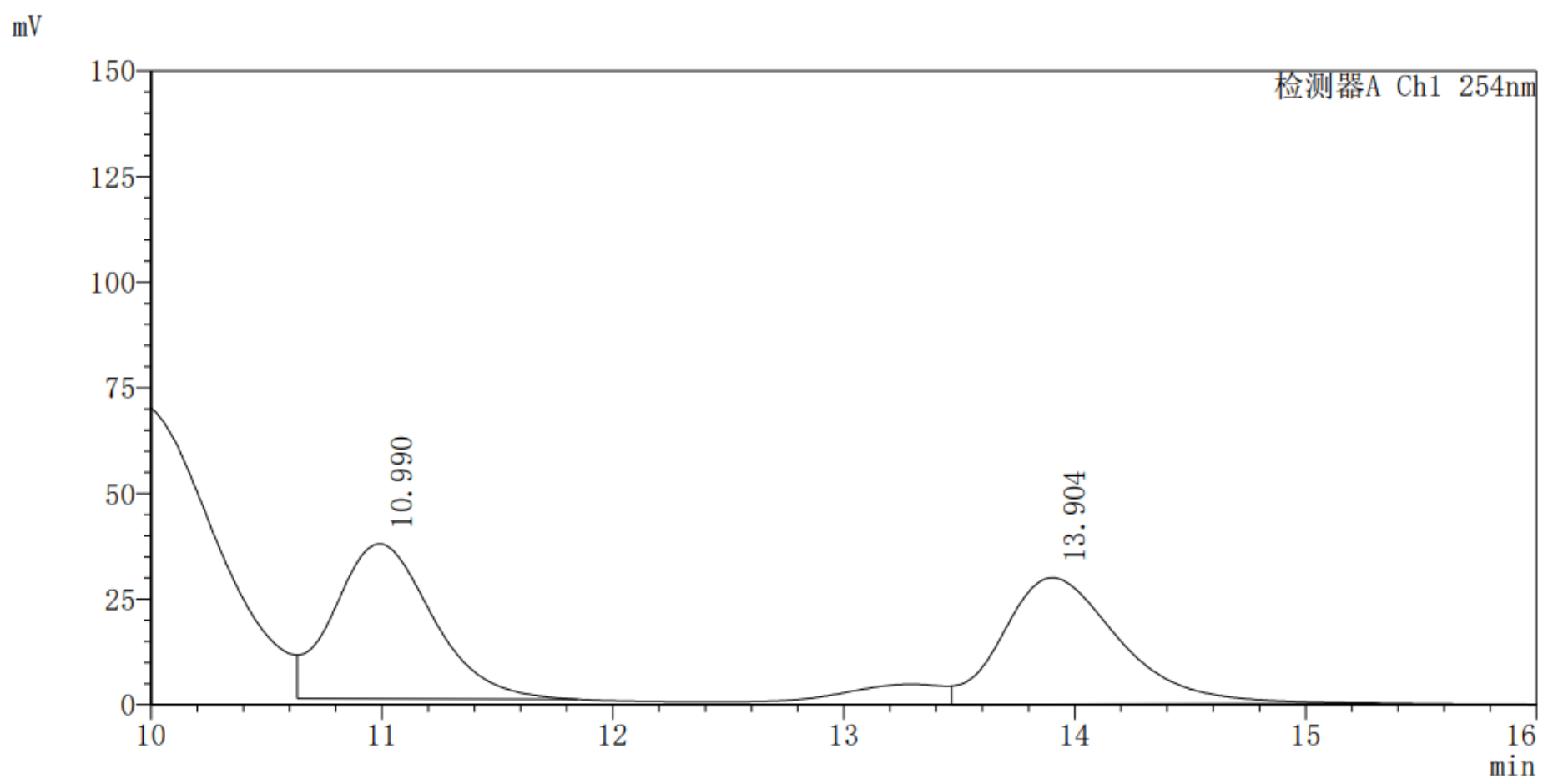

检测器A Ch1 254nm

No.	R.Time	Height	Height%	Area	Area%
1	10.990	36619	54.940	1089821	50.687
2	13.904	30034	45.060	1060280	49.313

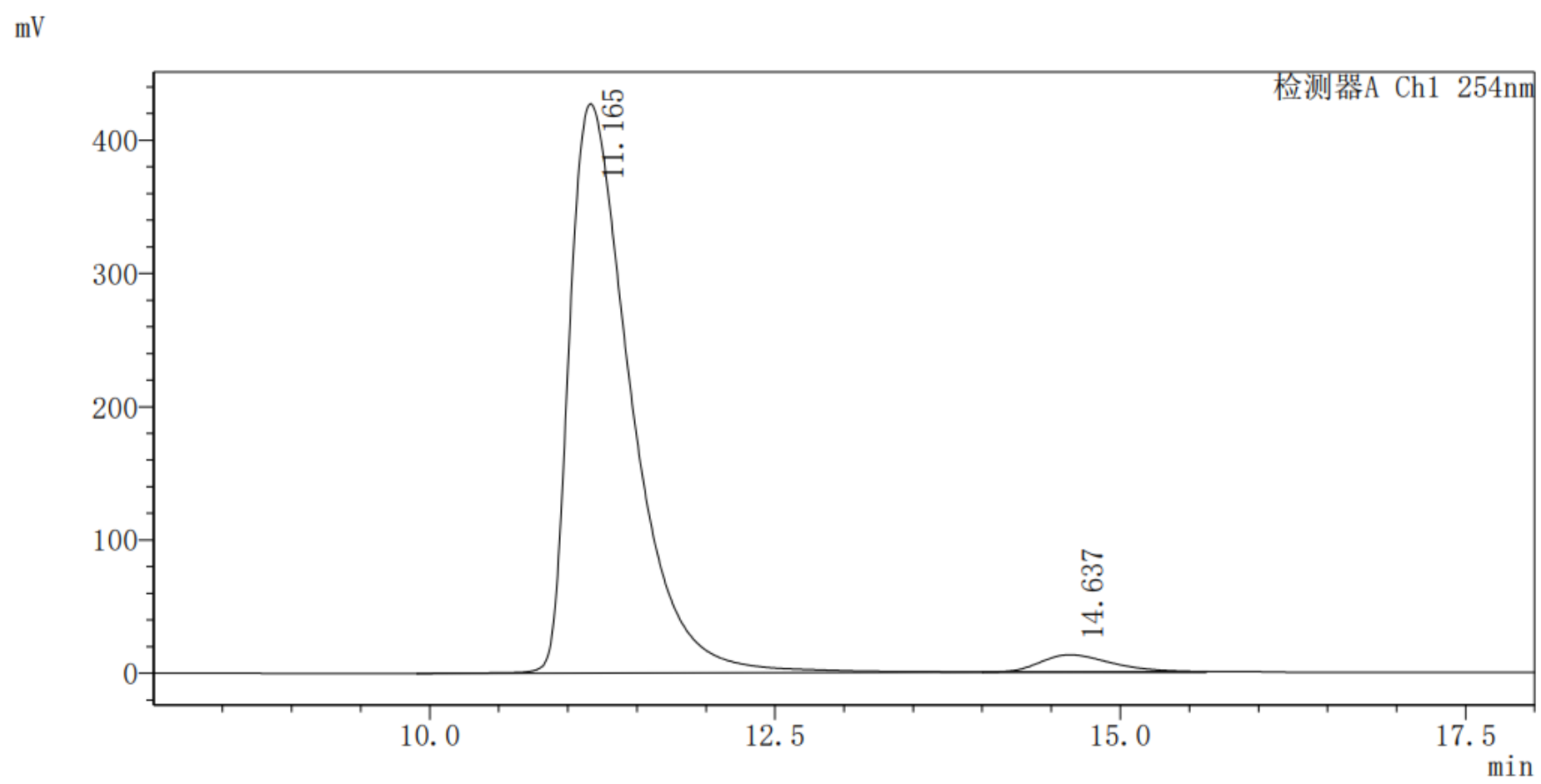

检测器A Ch1 254nm

No.	R.Time	Height	Height%	Area	Area%
1	11.165	427244	97.043	13173679	96.489
2	14.637	13020	2.957	479356	3.511

(*R*)-4,4,5-Triphenyl-6-[3-(*p*-tolyloxy)propyl]-4*H*-3l4,4l4-naphtho[2',1':5,6][1,2]azaborepino[7,1-a] isoquinoline (37)

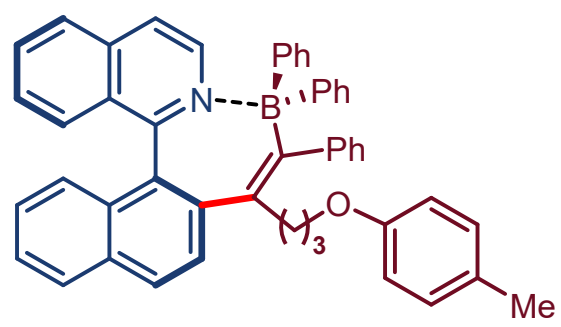

37

Prepared following **Procedure D**, using 1-(isoquinolin-1-yl)naphthalen-2-yl trifluoromethanesulfonate (80.6 mg, 0.2 mmol, 1.0 equiv.), tetramethylammonium triphenyl(5-(p-tolyloxy)pent-1-yn-1-yl)borate

(146.8 mg, 0.3 mmol, 1.5 equiv.), $Pd_2(dba)_3$ (9.16 mg, 0.001 mmol, 5.0 mol%) and (*R*)-**L1** (18.3 mg, 0.03 mmol, 15 mol%). Purification by flash column chromatography (Petroleum ether/EtOAc: 50/1) to afford the title compound (100.3 mg, 75%) as a yellow solid.

TLC: R_f = 0.5 (Petroleum ether/EtOAc: 50/1, $KMnO_4$ stain).

M. p.: 90 – 91 °C.

$[\alpha]^{18}_D$: +482.42 (c 0.20, CH_2Cl_2).

NMR Spectroscopy (*see spectra*):

^{1}H NMR (400 MHz, $CDCl_3$) δ_H = 9.01 (d, *J* = 6.9 Hz, 1H), 7.98 (d, *J* = 8.3 Hz, 1H), 7.83 (d, *J* = 6.9 Hz, 1H), 7.74 (dd, *J* = 7.3, 15.4 Hz, 2H), 7.64 (d, *J* = 5.8 Hz, 4H), 7.46 – 7.41 (m, 3H), 7.26 (d, *J* = 14.0 Hz, 2H), 7.17 (d, *J* = 8.0 Hz, 1H), 7.12 – 7.10 (m, 1H), 7.04 (dd, *J* = 7.1, 15.3 Hz, 2H), 6.93 – 6.88 (m, 3H), 6.73 (d, *J* = 6.6 Hz, 3H), 6.62 (t, *J* = 7.1 Hz, 2H), 6.43 (t, *J* = 8.8 Hz, 2H), 6.33 (d, *J* = 8.5 Hz, 2H), 6.23 (t, *J* = 7.3 Hz, 1H), 3.49 – 3.34 (m, 2H), 2.82 (td, *J* = 4.3, 8.9 Hz, 1H), 2.74 – 2.64 (m, 1H), 2.26 (s, 3H), 1.79 – 1.71 (m, 1H), 1.64 – 1.57 (m, 1H) ppm;

^{13}C NMR (100 MHz, $CDCl_3$) δ_C = 158.3, 156.7, 149.3, 143.5, 143.0, 138.4, 138.1, 138.1, 136.3, 134.9, 133.0, 132.6, 131.7, 130.9, 130.9, 130.7, 130.5, 130.4, 129.8, 129.7, 129.4, 129.2, 129.1, 128.9, 128.5, 127.9, 127.7, 127.3, 127.0, 126.7, 126.5, 126.0, 126.0, 125.9, 125.5, 124.9, 124.5, 124.1, 123.9, 122.9, 121.0, 113.9, 67.0, 29.4, 28.8, 20.5 ppm. The carbon attached to boron was not observed due to quadrupolar relaxation;

^{11}B NMR (128 MHz, $CDCl_3$) δ_B = 4.58 ppm.

IR (film): ν_{max} 33674, 3010, 2872, 2757, 1374, 1508, 1289, 1242, 999, 824, 749, 702 cm^{-1}.

HRMS (ESI): m/z calculated for $C_{49}H_{41}BNO^+$ $[M+H]^+$, 670.3276, found, 670.3269.

HPLC analysis: HPLC conditions: Chiral column IB, *n*-hexane/isopropanol: 99/1, flow rate = 1.0 mL/min, wavelength = 254 nm, t_R = 10.007 min for major isomer, t_R = 13.135 min for minor isomer, 90% ee.

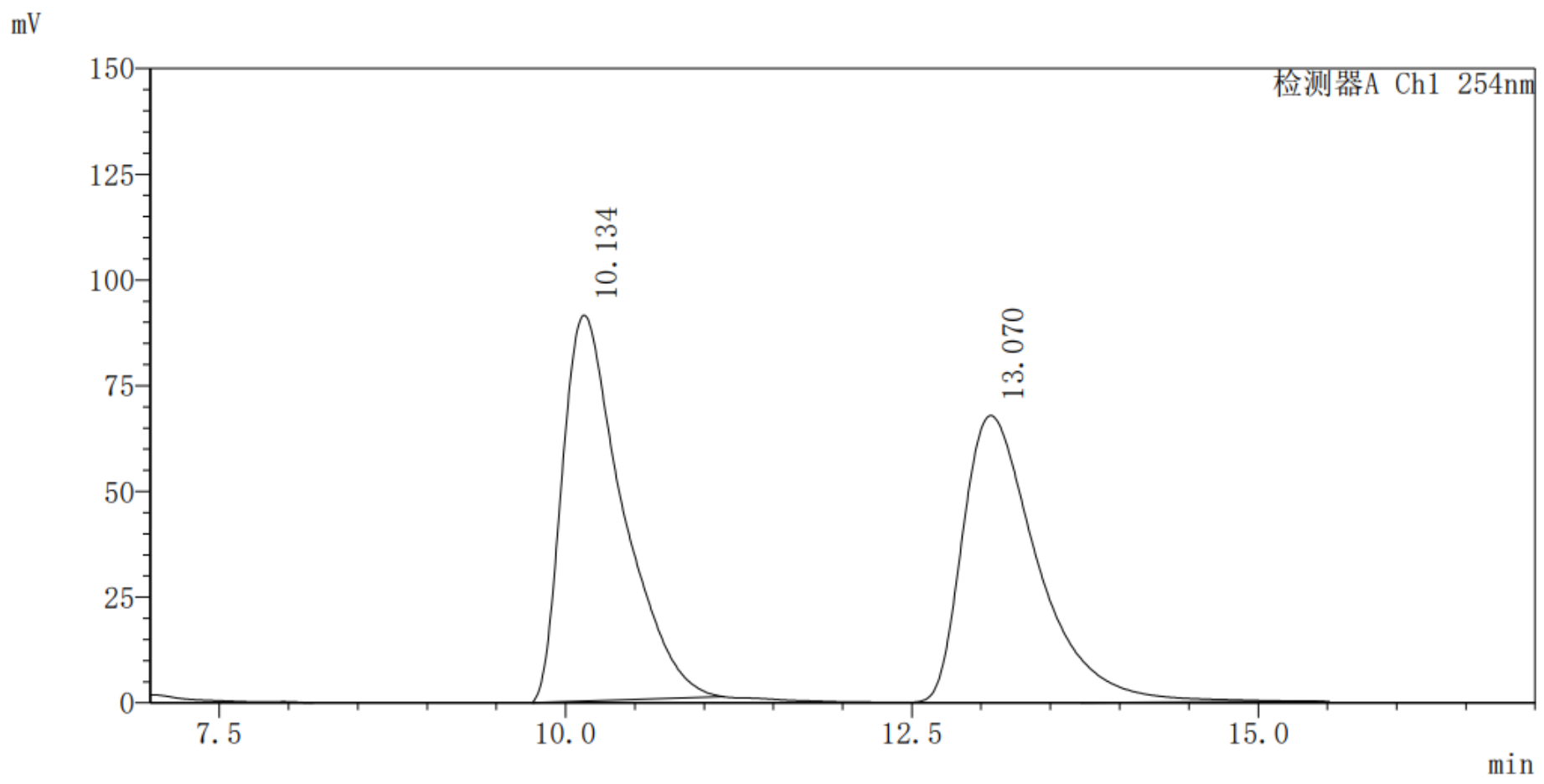

检测器A Ch1 254nm

No.	R.Time	Height	Height%	Area	Area%
1	10.134	91277	57.247	2763063	51.533
2	13.070	68166	42.753	2598653	48.467

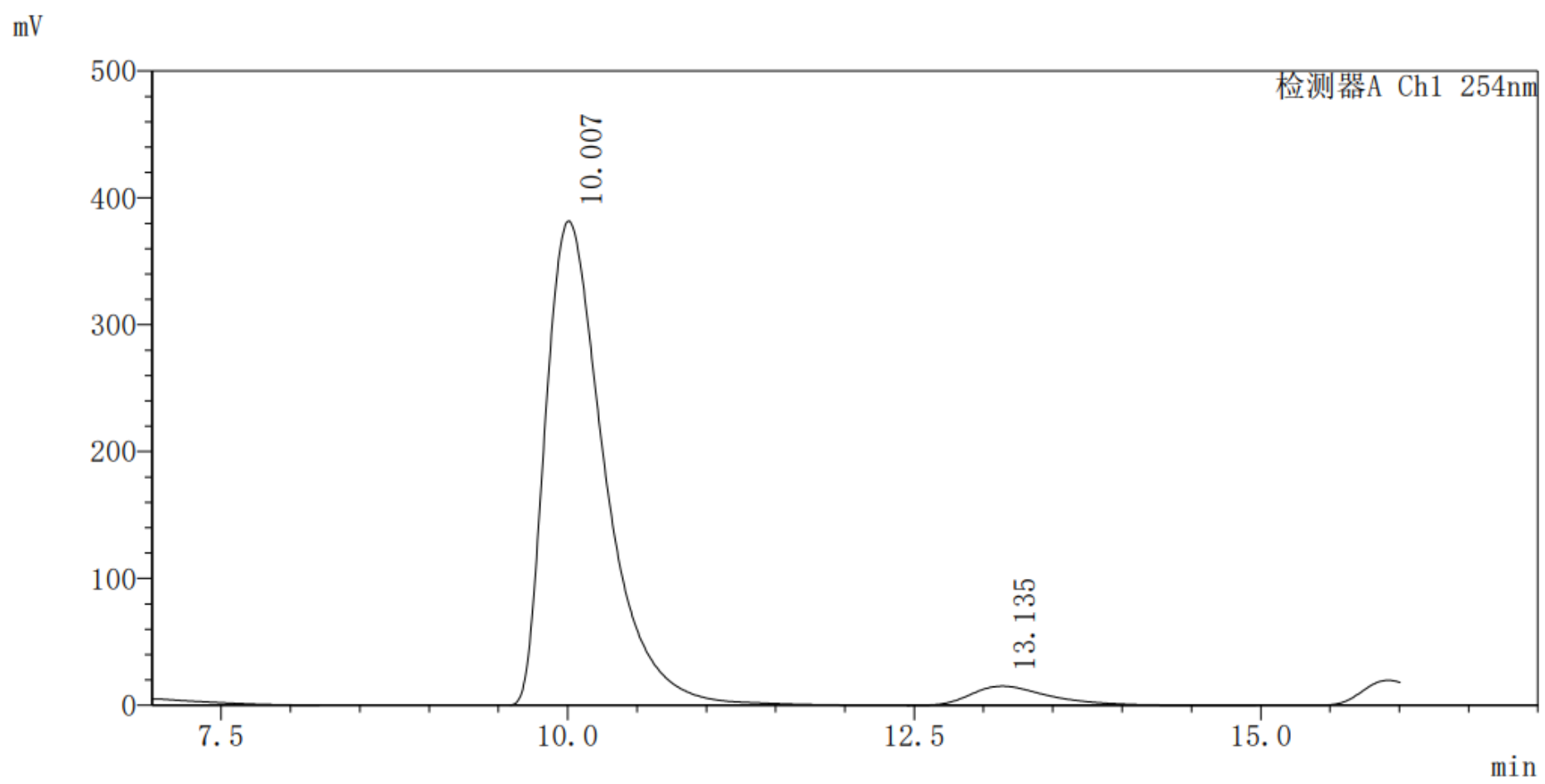

检测器A Ch1 254nm

No.	R.Time	Height	Height%	Area	Area%
1	10.007	382761	96.176	11451386	94.947
2	13.135	15218	3.824	609496	5.053

(*R*)-6-{3-[4-(*tert*-Butyl)phenoxy]propyl}-4,4,5-triphenyl-4*H*-3l4,4l4-naphtho[2',1':5,6][1,2] azaborepino[7,1-a]isoquinoline (38)

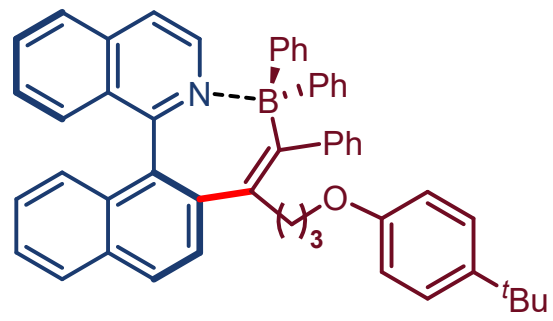

38

Prepared following **Procedure D**, using 1-(isoquinolin-1-yl)naphthalen-2-yl trifluoromethanesulfonate (80.6 mg, 0.2 mmol, 1.0 equiv.), tetramethylammonium {5-[4-(*tert*-butyl)phenoxy]pent-1-yn-1-

yl}triphenylborate (159.3 mg, 0.3 mmol, 1.5 equiv.), $Pd_2(dba)_3$ (9.16 mg, 0.001 mmol, 5.0 mol%) and (*R*)-**L1** (18.3 mg, 0.03 mmol, 15 mol%). Purification by flash column chromatography (Petroleum ether/EtOAc: 50/1) to afford the title compound (120.9 mg, 85%) as a yellow solid.

TLC: R_f = 0.5 (Petroleum ether/EtOAc: 50/1, $KMnO_4$ stain).

M. p.: 96 – 97 °C.

$[\alpha]^{18}_D$: +374.34 (c 0.20, CH_2Cl_2).

NMR Spectroscopy (*see spectra*):

^{1}H NMR (500 MHz, $CDCl_3$) δ_H = 9.04 (d, *J* = 6.9 Hz, 1H), 7.96 (d, *J* = 8.2 Hz, 1H), 7.85 (d, *J* = 6.9 Hz, 1H), 7.70 (t, *J* = 7.6 Hz, 1H), 7.67 (s, 2H), 7.64 (d, *J* = 8.1 Hz, 1H), 7.44 (d, *J* = 8.7 Hz, 1H), 7.31 – 7.26 (m, 2H), 7.22 – 7.12 (m, 4H), 7.05 (dt, *J* = 7.8, 14.6 Hz, 2H), 7.01 – 6.92 (m, 2H), 6.89 (t, *J* = 7.3 Hz, 2H), 6.79 – 6.69 (m, 4H), 6.65 (d, *J* = 7.6 Hz, 2H), 6.51 – 6.37 (m, 4H), 6.24 (t, *J* = 7.3 Hz, 1H), 3.51 (q, *J* = 7.2 Hz, 1H), 3.40 (td, *J* = 5.3, 8.5 Hz, 1H), 2.90 – 2.80 (m, 1H), 2.75 – 2.63 (m, 1H), 1.81-1.53 (m, 1H), 1.66-1.58 (m, 1H), 1.33 (s, 9H) ppm;

^{13}C NMR (125 MHz, $CDCl_3$) δ_C = 158.3, 156.5, 149.3, 143.0, 142.8, 138.4, 138.1, 138.0, 136.3, 133.0, 132.6, 131.7, 130.9, 130.9, 130.5, 130.4, 129.8, 129.7, 129.2, 128.9, 127.8, 127.7, 127.3, 127.0, 126.7, 126.5, 126.0, 125.9, 124.9, 124.5, 124.1, 123.9, 122.9, 121.0, 113.5, 67.1, 34.1, 31.7, 29.4, 28.9 ppm. The carbon attached to boron was not observed due to quadrupolar relaxation;

^{11}B NMR (128 MHz, $CDCl_3$) δ_B = 4.60 ppm.

IR (film): ν_{max} 3043, 2997, 2865, 1512, 1486, 1245, 1183, 909, 825, 729, 702 cm^{-1}.

HRMS (ESI): m/z calculated for $C_{52}H_{47}BNO^+$ $[M+H]^+$, 712.3745, found, 712.3747.

HPLC analysis: HPLC conditions: Chiral column IB, *n*-hexane/isopropanol: 99/1, flow rate = 1.0 mL/min, wavelength = 254 nm, t_R = 7.432 min for major isomer, t_R = 9.357 min for minor isomer, 95% ee.

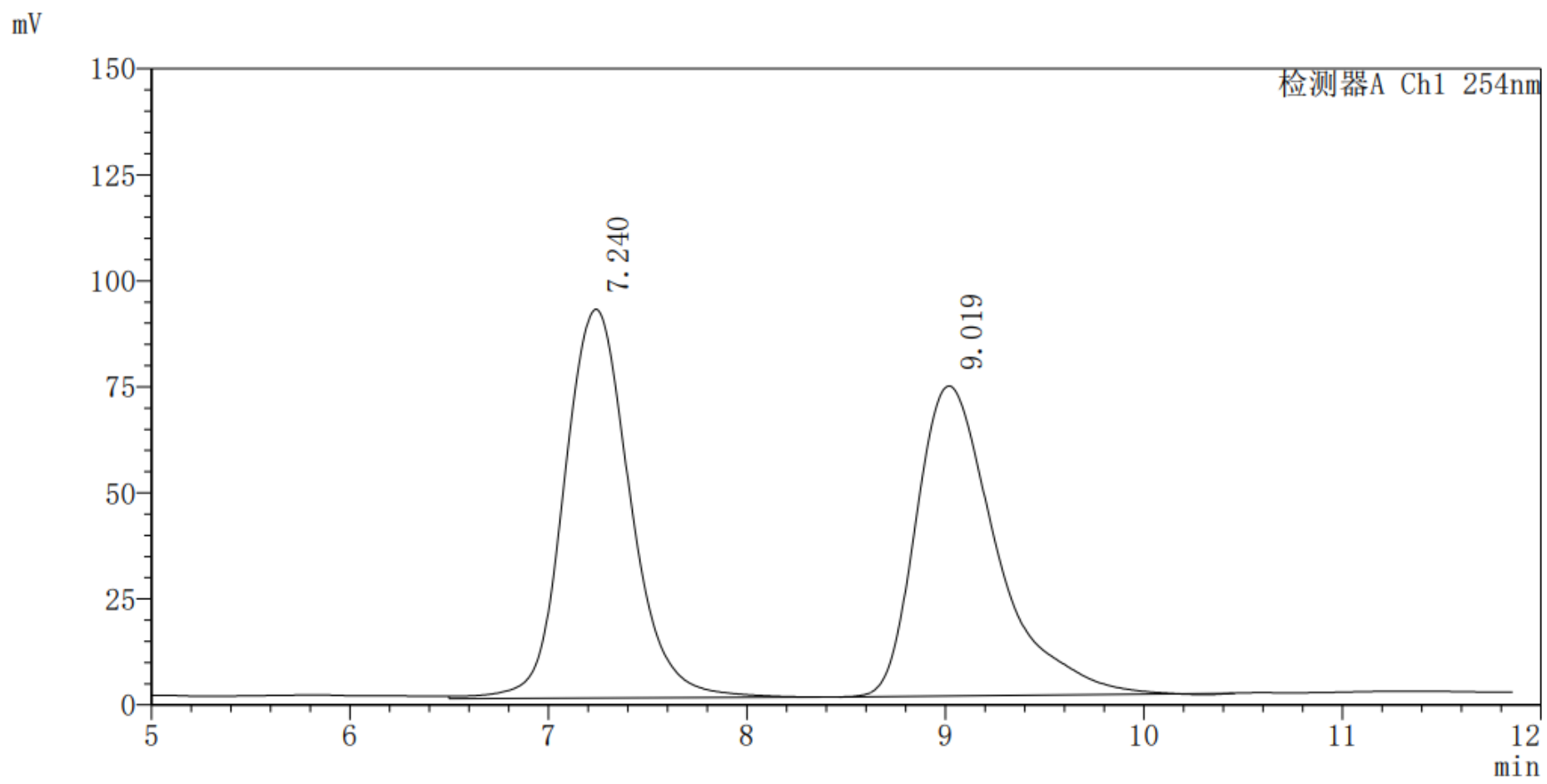

检测器A Ch1 254nm

No.	R.Time	Height	Height%	Area	Area%
1	7.240	91699	55.652	2117751	50.817
2	9.019	73074	44.348	2049623	49.183

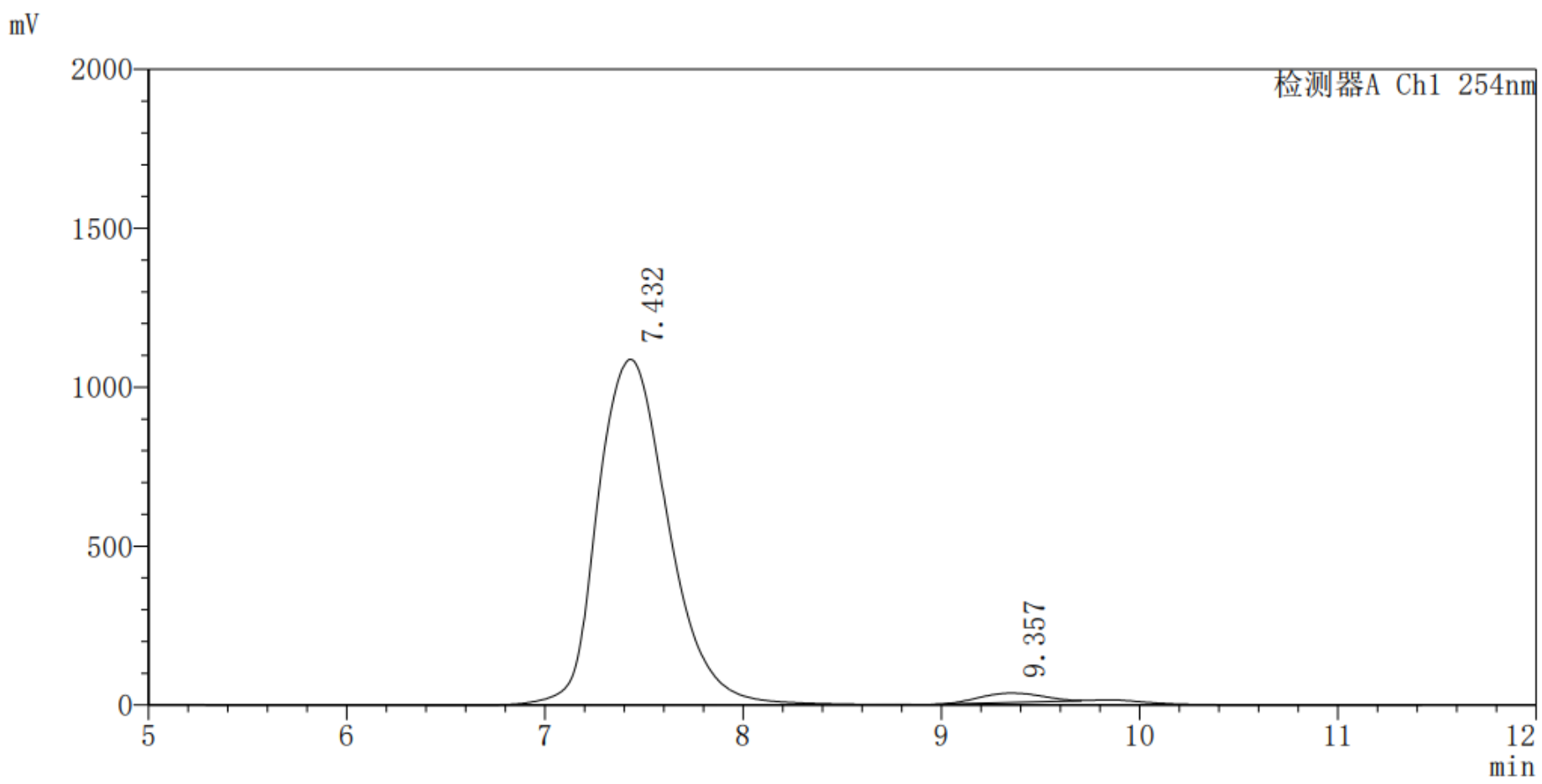

检测器A Ch1 254nm

No.	R.Time	Height	Height%	Area	Area%
1	7.432	1087244	97.387	26894554	97.641
2	9.357	29170	2.613	649822	2.359

(*R*)-6-[3-(3,5-Dimethylphenoxy)propyl]-4,4,5-triphenyl-4*H*-3l4,4l4-naphtho[2',1':5,6][1,2] azaborepino [7,1-a]isoquinoline (39)

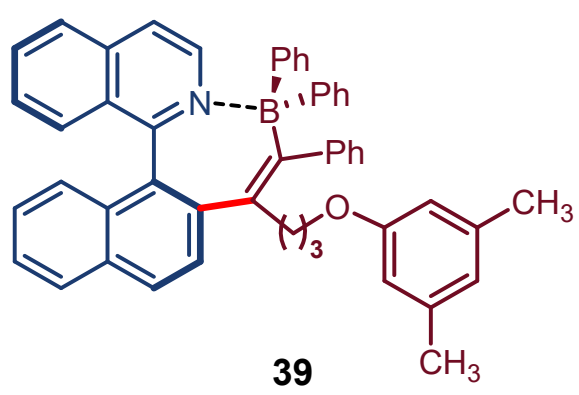

39

Prepared following **Procedure D**, using 1-(isoquinolin-1-yl)naphthalen-2-yl trifluoromethanesulfonate (80.6 mg, 0.2 mmol, 1.0 equiv.), tetramethylammonium [5-(3,5-dimethylphenoxy)pent-1-yn-1-

yl]triphenylborate (150.9 mg, 0.3 mmol, 1.5 equiv.), $Pd_2(dba)_3$ (9.16 mg, 0.001 mmol, 5.0 mol%) and (*R*)-**L1** (18.3 mg, 0.03 mmol, 15 mol%). Purification by flash column chromatography (Petroleum ether/EtOAc: 50/1) to afford the title compound (107.9 mg, 79%) as a yellow solid.

TLC: R_f = 0.5 (Petroleum ether/EtOAc: 50/1, $KMnO_4$ stain).

M. p.: 83 – 84 °C.

$[\alpha]^{18}_D$: +291.62 (c 0.20, CH_2Cl_2).

NMR Spectroscopy (*see spectra*):

> **^{1}H NMR** (400 MHz, $CDCl_3$) δ_H = 9.02 (d, *J* = 6.8 Hz, 1H), 7.97 (d, *J* = 8.3 Hz, 1H), 7.84 (d, *J* = 7.0 Hz, 1H), 7.73 (t, *J* = 7.0 Hz, 1H), 7.66 (s, 2H), 7.62 (d, *J* = 8.4 Hz, 1H), 7.46 – 7.41 (m, 2H), 7.30 (t, *J* = 7.1 Hz, 2H), 7.20 – 7.11 (m, 2H), 7.05 – 6.88 (m, 5H), 6.77 – 6.68 (m, 4H), 6.63 (d, *J* = 7.6 Hz, 2H), 6.52 (s, 1H), 6.44 (d, *J* = 8.6 Hz, 2H), 6.23 (t, *J* = 7.3 Hz, 1H), 6.12 (s, 2H), 3.51 – 3.33 (m, 2H), 2.89-2.78 (m, 1H), 2.75-2.62 (m, 1H), 2.21 (s, 6H), 1.80 – 11.70 (m, 1H), 1.63 – 1.55 (m, 1H) ppm;

> **^{13}C NMR** (100 MHz, $CDCl_3$) δ_C = 138.8, 138.4, 138.1, 138.0, 136.3, 134.9, 133.0, 132.6, 131.7, 130.9, 130.9, 130.6, 130.5, 130.4, 129.8, 129.7, 129.2, 129.1, 128.9, 128.5, 127.8, 127.7, 127.3, 127.0, 126.7, 126.5, 126.0, 126.0, 125.9, 125.5, 124.9, 124.5, 124.1, 123.9, 122.9, 122.1, 120.9, 111.9, 66.8, 29.5, 28.9, 21.5 ppm. The carbon attached to boron was not observed due to quadrupolar relaxation;

> **^{11}B NMR** (128 MHz, $CDCl_3$) δ_B = 4.01 ppm.

IR (film): ν_{max} 3688, 3010, 2873, 2758, 1742, 1623, 1474, 1294, 999, 827, 749, 702 cm^{-1}.

HRMS (ESI): m/z calculated for $C_{50}H_{43}BNO^+$ $[M+H]^+$, 684.3432, found, 684.3441.

HPLC analysis: HPLC conditions: Chiral column IB, *n*-hexane/isopropanol: 99/1, flow rate = 1.0 mL/min, wavelength = 254 nm, t_R = 9.550 min for major isomer, t_R = 11.760 min for minor isomer, 91% ee.

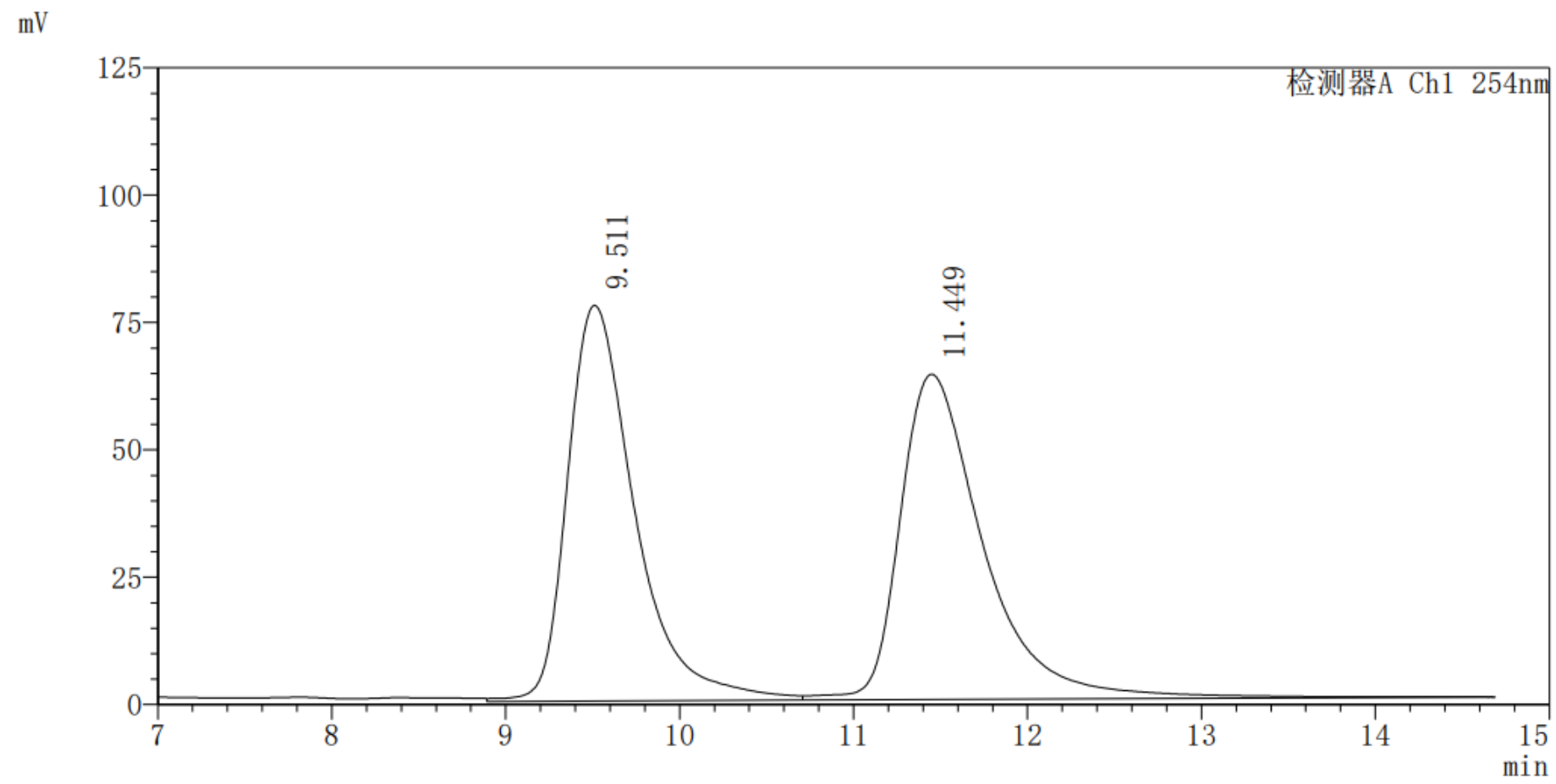

检测器A Ch1 254nm

No.	R.Time	Height	Height%	Area	Area%
1	9.511	77686	54.885	2092604	49.439
2	11.449	63858	45.115	2140075	50.561

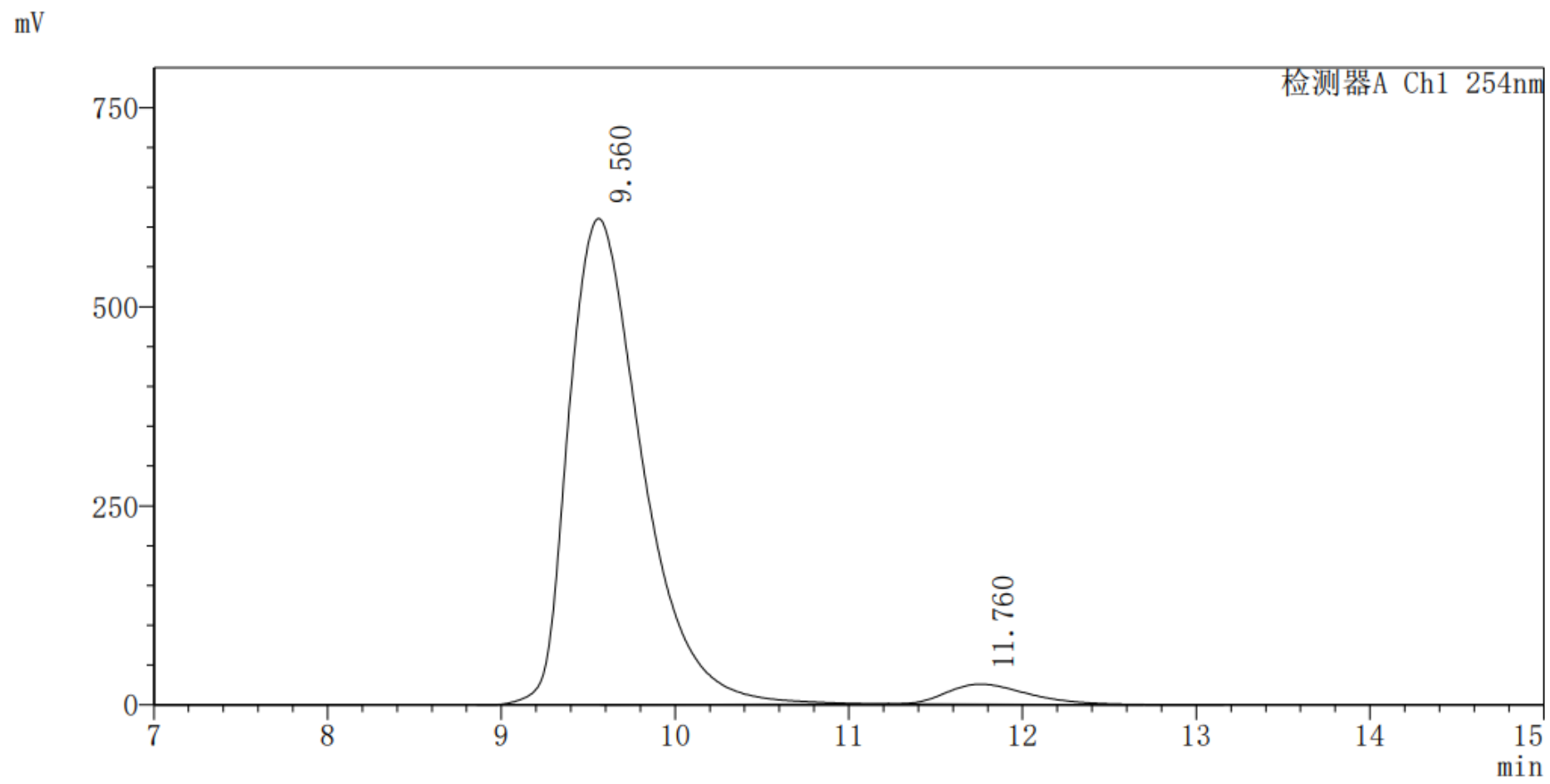

检测器A Ch1 254nm

No.	R.Time	Height	Height%	Area	Area%
1	9.560	611033	96.011	17975129	95.434
2	11.760	25384	3.989	860023	4.566

(*R*)-6-[3-(4-Chlorophenoxy)propyl]-4,4,5-triphenyl-4*H*-3l4,4l4-naphtho[2',1':5,6][1,2] azaborepino [7,1-a] isoquinoline (40)

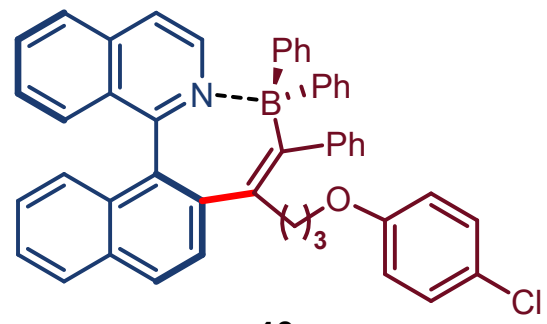

40

Prepared following **Procedure D**, using 1-(isoquinolin-1-yl)naphthalen-2-yl trifluoromethanesulfonate (80.6 mg, 0.2 mmol, 1.0 equiv.), tetramethylammonium [5-(4-chlorophenoxy)pent-1-yn-1-

yl]triphenylborate (152.7 mg, 0.3 mmol, 1.5 equiv.), $Pd_2(dba)_3$ (9.16 mg, 0.001 mmol, 5.0 mol%) and (*R*)-**L1** (18.3 mg, 0.03 mmol, 15 mol%). Purification by flash column chromatography (Petroleum ether/EtOAc: 50/1) to afford the title compound (100.6 mg, 73%) as a yellow solid.

TLC: $R_f = 0.5$ (Petroleum ether/EtOAc: 50/1, $KMnO_4$ stain).

M. p.: 97 – 98 °C.

$[\alpha]^{18}_D$: +528.36 (c 0.20, CH_2Cl_2).

NMR Spectroscopy (*see spectra*):

^{1}H NMR (400 MHz, $CDCl_3$) δ_H = 9.04 (d, *J* = 6.9 Hz, 1H), 7.99 (d, *J* = 8.3 Hz, 1H), 7.85 (d, *J* = 6.9 Hz, 1H), 7.74 (t, *J* = 7.0 Hz, 1H), 7.69 – 7.62 (m, 4H), 7.48 – 7.40 (m, 3H), 7.29 (d, *J* = 8.4 Hz, 2H), 7.18 (d, *J* = 7.5 Hz, 1H), 7.13 (d, *J* = 8.6 Hz, 1H), 7.06 (d, *J* = 9.0 Hz, 3H), 6.97 – 6.87 (m, 3H), 6.75 (d, *J* = 6.6 Hz, 2H), 6.64 (d, *J* = 7.5 Hz, 3H), 6.45 (q, *J* = 8.6 Hz, 2H), 6.29 (d, *J* = 8.9 Hz, 2H), 6.23 (t, *J* = 6.9 Hz, 1H), 3.41 (q, *J* = 7.1 Hz, 1H), 3.35 – 3.26 (m, 1H), 2.90 – 2.79 (m, 1H), 2.77 – 2.65 (m, 1H), 1.85 – 1.71 (m, 1H), 1.67 – 1.59 (m, 1H) ppm;

^{13}C NMR (100 MHz, $CDCl_3$) δ_C = 158.2, 157.4, 149.2, 143.5, 142.8, 138.1, 138.1, 138.0, 136.3, 134.8, 133.5, 133.0, 132.5, 131.7, 130.8, 130.6, 130.4, 130.3, 129.8, 129.7, 129.2, 129.1, 128.9, 128.5, 127.7, 127.4, 127.0, 126.7, 126.6, 126.0, 125.9, 125.5, 125.0, 125.0, 124.9, 124.5, 124.1, 123.8, 122.9, 121.0, 115.2, 67.1, 28.9, 28.6 ppm. The carbon attached to boron was not observed due to quadrupolar relaxation;

^{11}B NMR (128 MHz, $CDCl_3$) δ_B = 4.01 ppm.

IR (film): ν_{max} 3693, 3043, 2997, 2773, 1622, 1491, 1283, 1243, 1093, 823, 750, 702 cm^{-1}.

HRMS (ESI): m/z calculated for $C_{48}H_{38}BClNO^+$ $[M+H]^+$, 690.2729, found, 670.2732.

HPLC analysis: HPLC conditions: Chiral column IB, *n*-hexane/isopropanol: 99/1, flow rate = 1.0 mL/min, wavelength = 254 nm, t_R = 10.940 min for major isomer, t_R = 14.811 min for minor isomer, 95% ee.

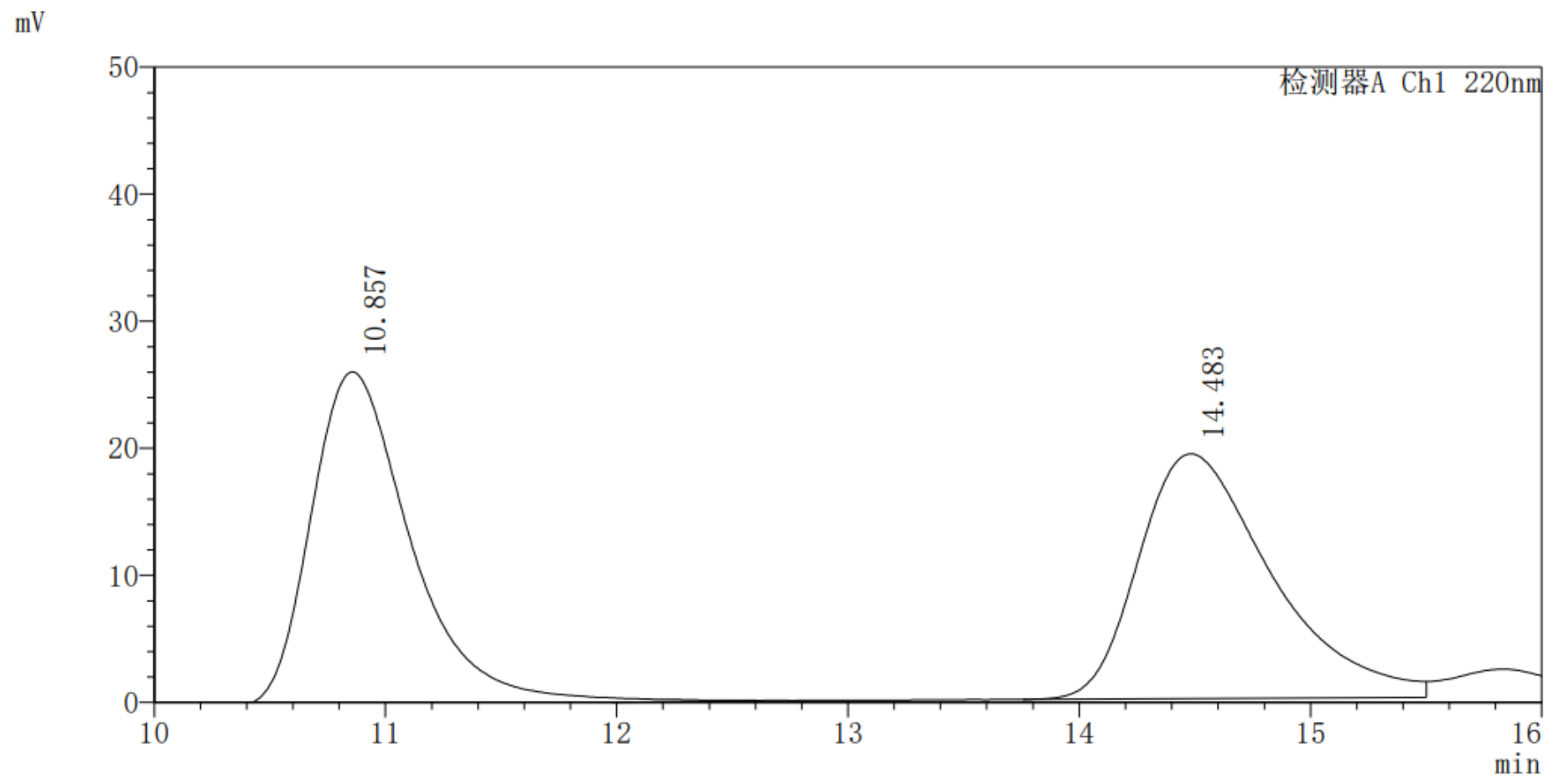

检测器A Ch1 220nm

No.	R.Time	Height	Height%	Area	Area%
1	10.857	26243	57.668	790542	50.658
2	14.483	19265	42.332	769992	49.342

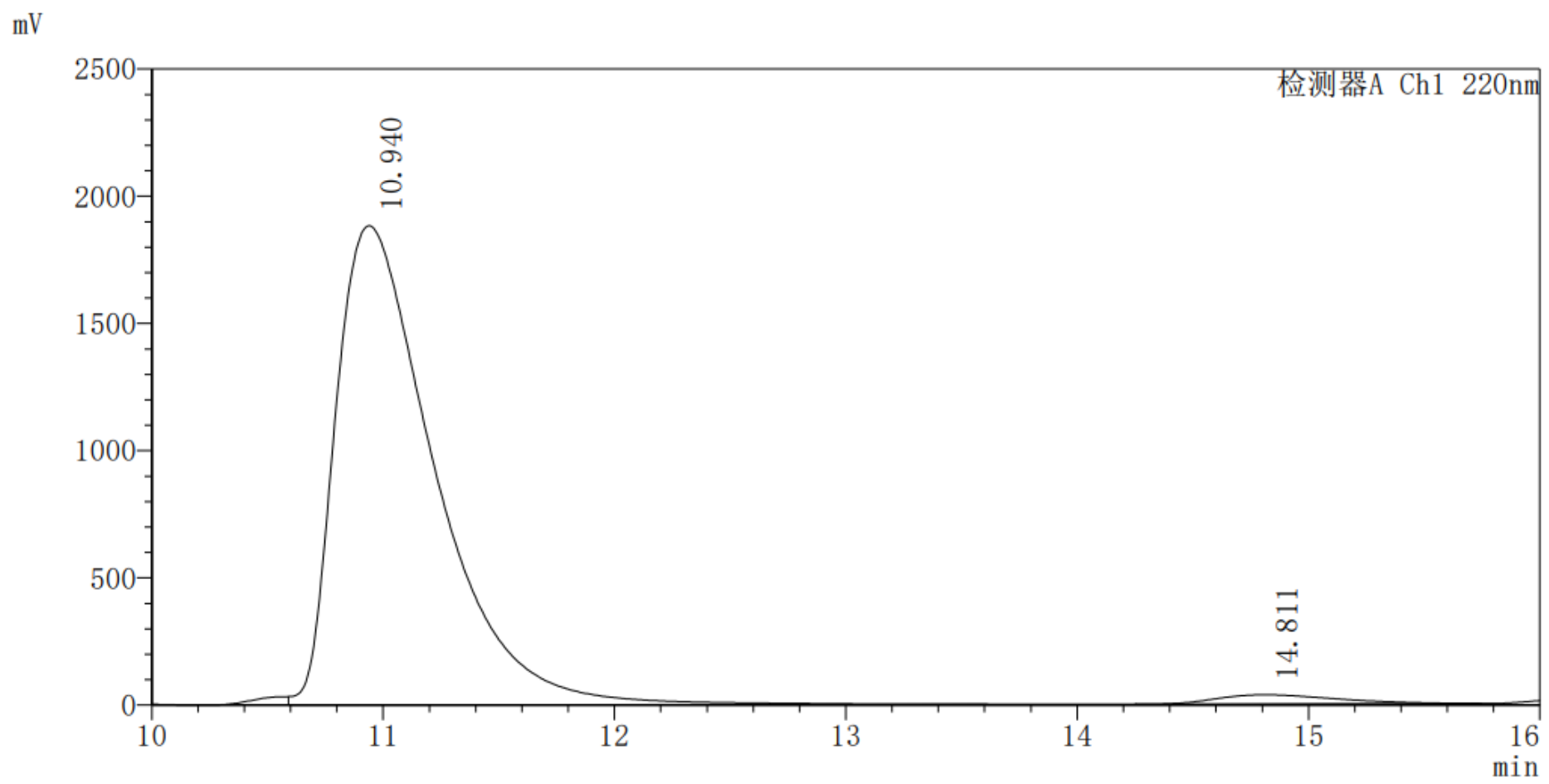

检测器A Ch1 220nm

No.	R.Time	Height	Height%	Area	Area%
1	10.940	1883890	98.109	56080261	97.712
2	14.811	36305	1.891	1313074	2.288

(*R*)-6-[3-(4-Fluorophenoxy)propyl]-4,4,5-triphenyl-4*H*-3l4,4l4-naphtho[2',1':5,6][1,2] azaborepino [7,1-a] isoquinoline (41)

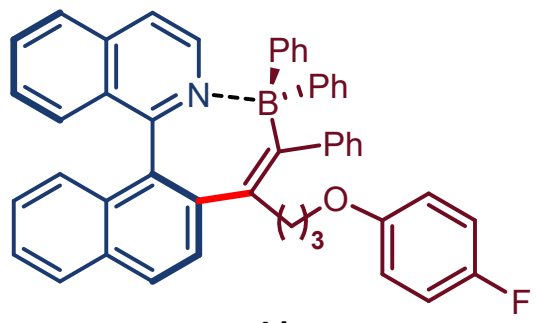

41

Prepared following **Procedure D**, using 1-(isoquinolin-1-yl)naphthalen-2-yl trifluoromethanesulfonate (80.6 mg, 0.2 mmol, 1.0 equiv.), tetramethylammonium [5-(4-fluorophenoxy)pent-1-yn-1-

yl]triphenylborate (147.9 mg, 0.3 mmol, 1.5 equiv.), $Pd_2(dba)_3$ (9.16 mg, 0.001 mmol, 5.0 mol%) and (*R*)-**L1** (18.3 mg, 0.03 mmol, 15 mol%). Purification by flash column chromatography (Petroleum ether/EtOAc: 50/1) to afford the title compound (115.8 mg, 86%) as a yellow solid.

TLC: R_f = 0.5 (Petroleum ether/EtOAc: 50/1, $KMnO_4$ stain).

M. p.: 78 – 79 °C.

$[\alpha]^{18}_D$: +283.48 (c 0.20, CH_2Cl_2).

NMR Spectroscopy (*see spectra*):

^{1}H NMR (400 MHz, $CDCl_3$) δ_H = 9.06 (d, *J* = 6.9 Hz, 1H), 7.99 (d, *J* = 8.1 Hz, 1H), 7.86 (t, *J* = 7.7 Hz, 2H), 7.74 (t, *J* = 7.0 Hz, 1H), 7.69 – 7.65 (m, 3H), 7.52 – 7.44 (m, 3H), 7.32 – 7.26 (m, 2H), 7.21 (d, *J* = 8.9 Hz, 1H), 7.18 – 7.13 (m, 1H), 7.09 – 7.05 (m, 1H), 7.00 – 6.90 (m, 3H), 6.85 – 6.77 (m, 4H), 6.71 – 6.65 (m, 3H), 6.51 – 6.43 (m, 2H), 6.38 – 6.30 (m, 2H), 6.26 (td, *J* = 1.4, 7.4 Hz, 1H), 3.49 – 3.39 (m, 1H), 3.37 – 3.29 (m, 1H), 2.92 – 2.87 (m, 1H), 2.74 (dt, *J* = 8.3, 14.6 Hz, 1H), 1.82 – 1.75 (m, 1H), 1.69 – 1.62 (m, 1H) ppm;

^{13}C NMR (100 MHz, $CDCl_3$) δ_C = 158.3, 156.9 (d, $^1J_{C\text{-}F}$ = 237.6 Hz), 154.9, 149.3, 143.5, 142.9, 138.2, 138.0, 136.3, 134.9, 133.0, 132.6, 131.7, 130.9, 130.7, 130.5, 130.4, 129.8, 129.7, 129.2, 129.1, 128.9, 128.5, 127.8, 127.7, 127.4, 127.0, 126.7, 126.6, 126.0, 125.9, 125.5, 125.0, 124.6, 124.1, 123.9, 122.9, 121.0, 115.5 (d, $^2J_{C\text{-}F}$ = 23.2 Hz), 114.8 (d,$^3J_{C\text{-}F}$ = 7.8 Hz), 67.5, 29.1, 28.7 ppm. The carbon attached to boron was not observed due to quadrupolar relaxation;

^{11}B NMR (128 MHz, $CDCl_3$) δ_B = 3.65 ppm;

^{19}F NMR (376 MHz, $CDCl_3$) δ_F = -124.58 ppm.

IR (film): ν_{max} 3703, 3044, 2922, 1631, 1550, 1505, 1470, 1429, 1246, 1160, 825, 750, 701 cm^{-1}.

HRMS (ESI): m/z calculated for $C_{48}H_{38}BFNO^+$ $[M+H]^+$, 674.3025, found, 674.3021.

HPLC analysis: HPLC conditions: Chiral column IB, *n*-hexane/isopropanol: 99/1, flow rate = 1.0 mL/min, wavelength = 254 nm, t_R = 10.896 min for major isomer, t_R = 14.794 min for minor isomer, 93% ee.

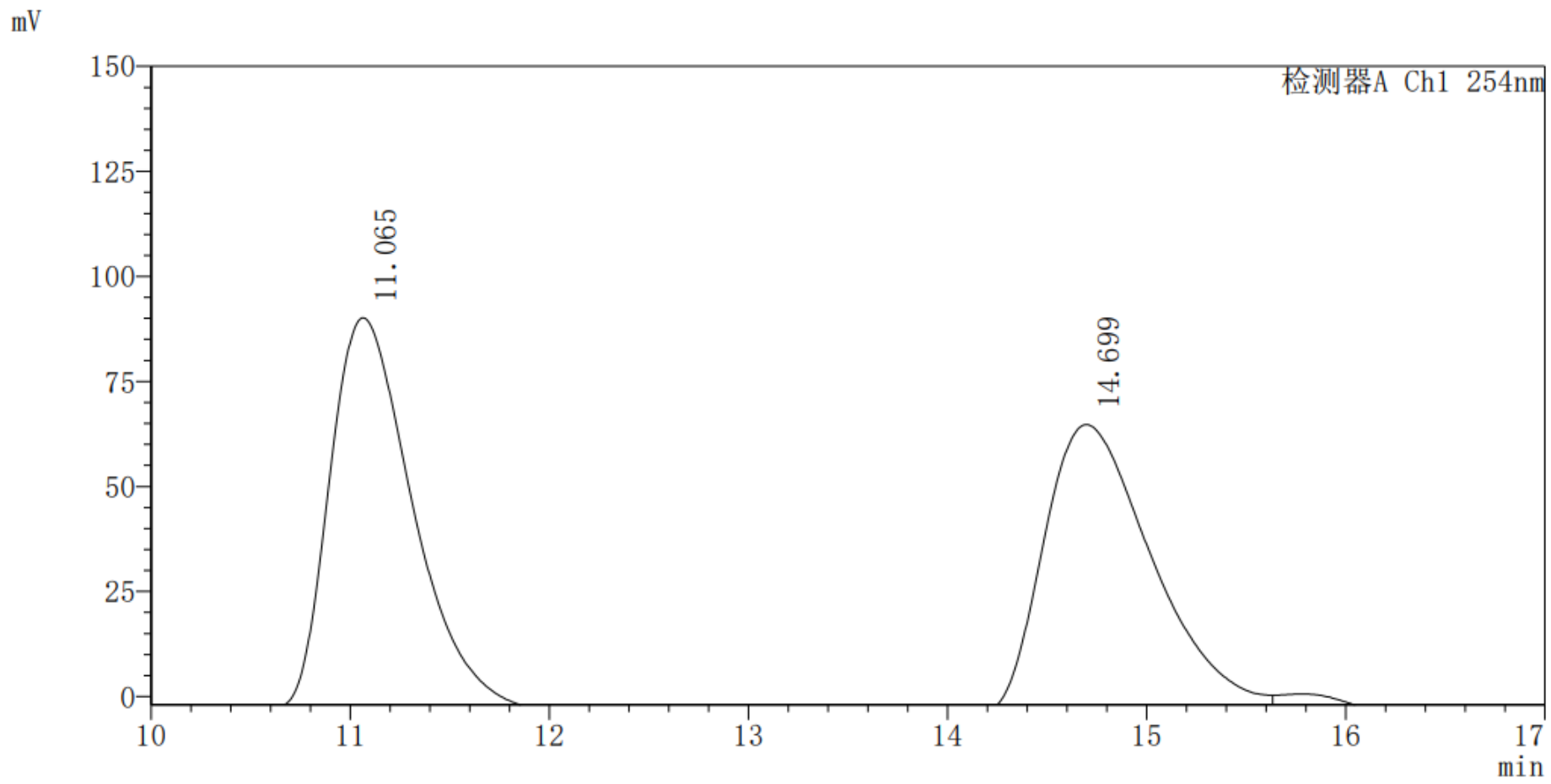

检测器A Ch1 254nm

No.	R.Time	Height	Height%	Area	Area%
1	11.065	95113	57.499	2967310	51.287
2	14.699	70302	42.501	2818379	48.713

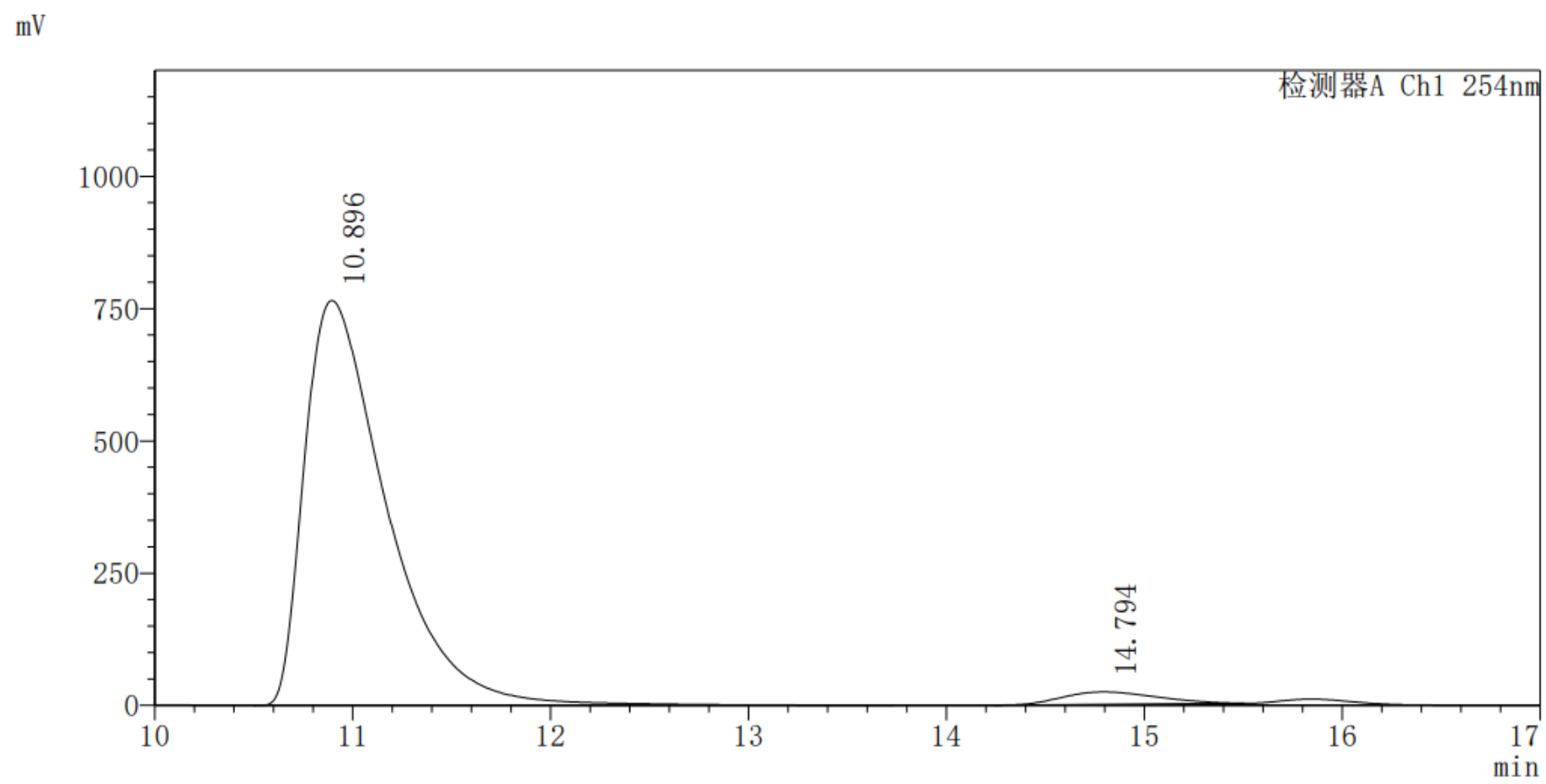

检测器A Ch1 254nm

No.	R.Time	Height	Height%	Area	Area%
1	10.896	765577	97.013	22156591	96.568
2	14.794	23572	2.987	787398	3.432

(*R*)-6-{3-([1,1'-Biphenyl]-4-yloxy)propyl}-4,4,5-triphenyl-4*H*-3l4,4l4 naphtho[2',1':5,6] [1,2] azaborepino [7,1-a]isoquinoline (42)

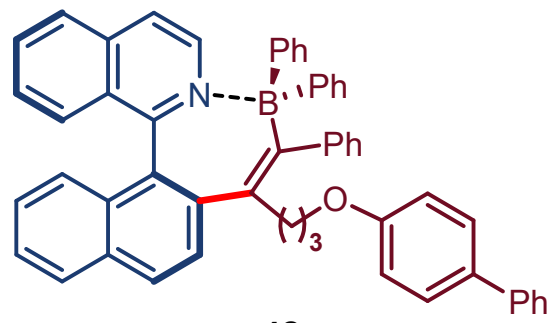

42

Prepared following **Procedure D**, using 1-(isoquinolin-1-yl)naphthalen-2-yl trifluoromethanesulfonate (80.6 mg, 0.2 mmol, 1.0 equiv.), tetramethylammonium {5-([1,1'-biphenyl]-4-yloxy)pent-1-yn-1-

yl}triphenylborate (165.3 mg, 0.3 mmol, 1.5 equiv.), $Pd_2(dba)_3$ (9.16 mg, 0.001 mmol, 5.0 mol%) and (*R*)-**L1** (18.3 mg, 0.03 mmol, 15 mol%). Purification by flash column chromatography (Petroleum ether/EtOAc: 50/1) to afford the title compound (119.9 mg, 82%) as a yellow solid.

TLC: R_f = 0.5 (Petroleum ether/EtOAc: 50/1, $KMnO_4$ stain).

M. p.: 86 – 87 °C.

$[\alpha]^{18}_D$: +208.15 (c 0.20, CH_2Cl_2).

NMR Spectroscopy (*see spectra*):

> **^{1}H NMR** (400 MHz, $CDCl_3$) δ_H = 9.03 (d, *J* = 6.9 Hz, 1H), 7.96 (d, *J* = 8.3 Hz, 1H), 7.84 (d, *J* = 6.8 Hz, 1H), 7.71 – 7.62 (m, 4H), 7.57 – 7.53 (m, 2H), 7.45 (t, *J* = 7.7 Hz, 3H), 7.38 – 7.33 (m, 3H), 7.30 – 7.25 (m, 2H), 7.19 – 7.12 (m, 2H), 7.08 – 6.96 (m, 3H), 6.93 – 6.84 (m, 3H), 6.77 – 6.68 (m, 4H), 6.66 – 6.61 (m, 2H), 6.49 – 6.40 (m, 4H), 6.23 (t, *J* = 6.7 Hz, 1H), 3.51 (q, *J* = 7.3 Hz, 1H), 3.44 – 3.35 (m, 1H), 2.93 – 2.80 (m, 1H), 2.78 – 2.66 (m, 1H), 1.83 – 1.76 (m, 1H), 1.69 – 1.62 (m, 1H) ppm;
>
> **^{13}C NMR** (100 MHz, $CDCl_3$) δ_C = 158.4, 158.3, 149.3, 142.9, 140.9, 138.2, 138.1, 138.0, 136.3, 133.2, 133.0, 132.6, 131.7, 130.9, 130.9, 130.5, 130.4, 129.8, 129.8, 129.2, 129.1, 128.9, 128.9, 128.5, 127.9, 127.8, 127.8, 127.4, 127.0, 126.7, 126.7, 126.5, 126.0, 125.9, 125.0, 124.5, 124.1, 123.9, 122.9, 121.0, 115.4, 114.3, 67.1, 29.2, 28.7 ppm. The carbon attached to boron was not observed due to quadrupolar relaxation;
>
> **^{11}B NMR** (128 MHz, $CDCl_3$) δ_B = 3.41 ppm.

IR (film): ν_{max} 3690, 3642, 2998, 2757, 1748, 1626, 1487, 1290, 1246, 993, 830, 750, 702 cm^{-1}.

HRMS (ESI): m/z calculated for $C_{54}H_{43}BNO^+$ $[M+H]^+$, 732.3432, found, 732.3436.

HPLC analysis: HPLC conditions: Chiral column IB, *n*-hexane/isopropanol: 99/1, flow rate = 1.0 mL/min, wavelength = 254 nm, t_R = 29.281 min for major isomer, t_R = 28.039 min for minor isomer, 95% ee.

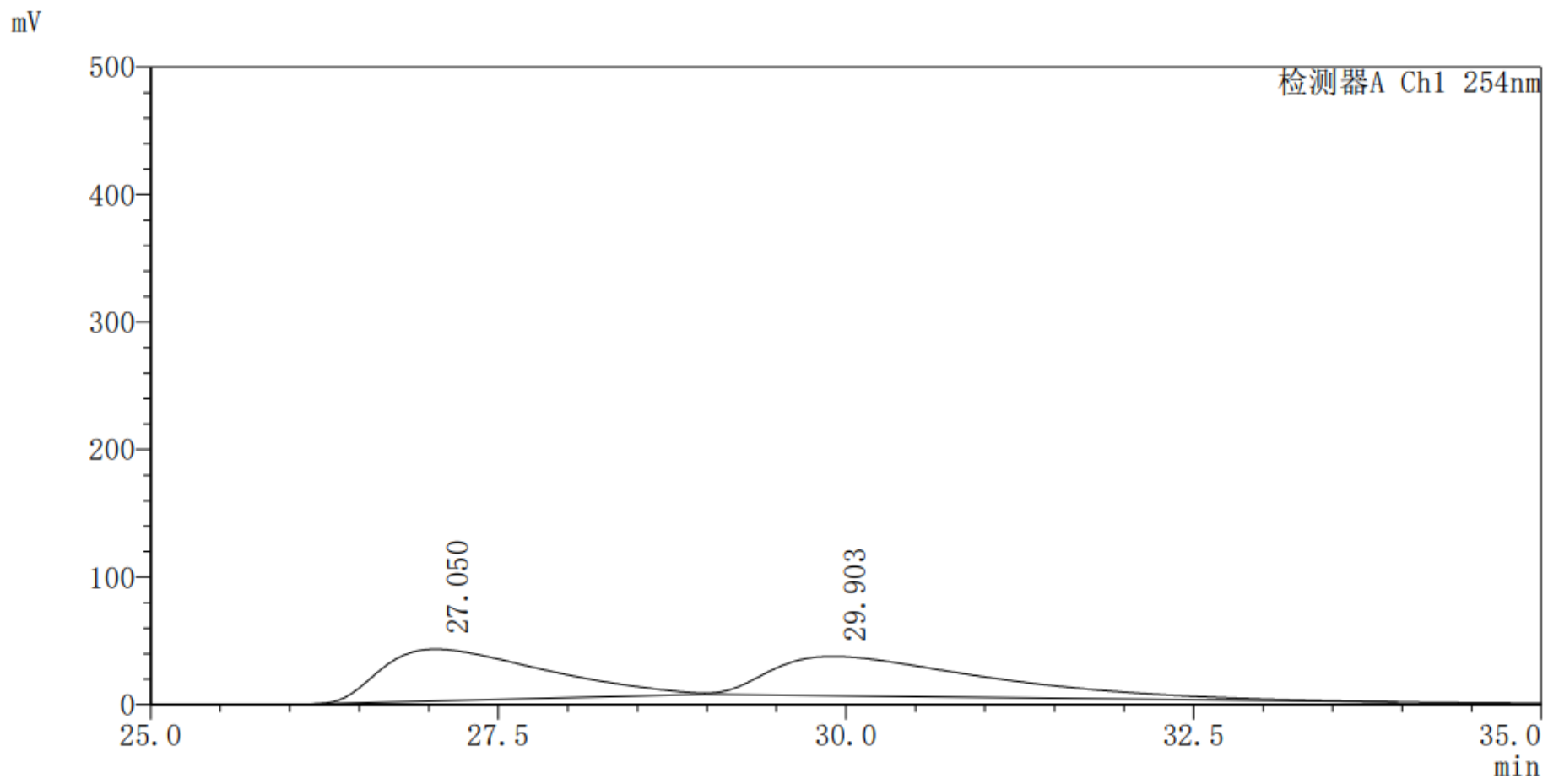

检测器A Ch1 254nm

No.	R.Time	Height	Height%	Area	Area%
1	27.050	40537	56.911	3292790	49.811
2	29.903	30693	43.089	3317840	50.189

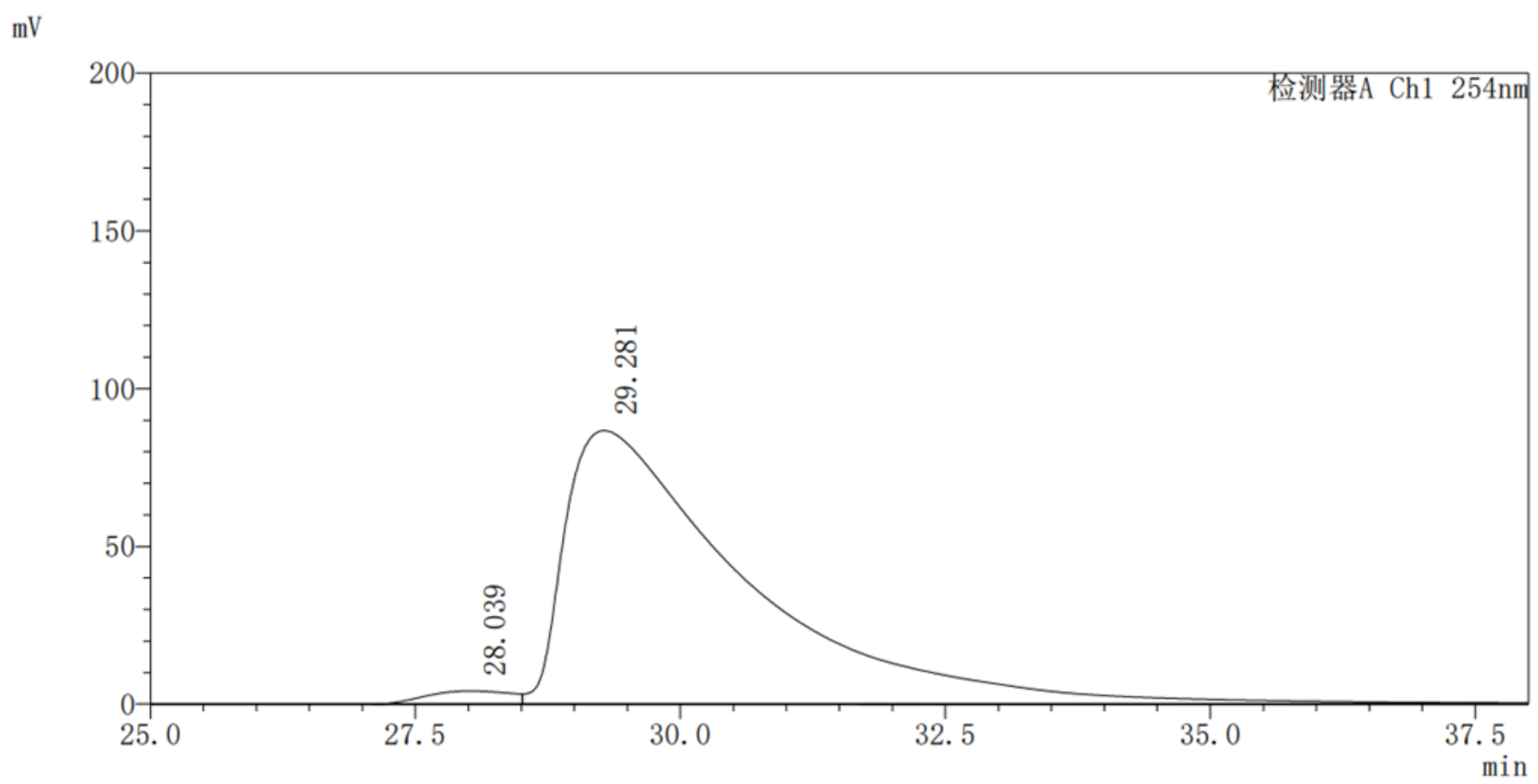

检测器A Ch1 254nm

No.	R.Time	Height	Height%	Area	Area%
1	28.039	4428	4.849	255555	2.411
2	29.281	86892	95.151	10345506	97.589
总计		91321	100.000	10601061	100.000

(*R*)-6-[3-(Naphthalen-2-yloxy)propyl]-4,4,5-triphenyl-4*H*-3l4,4l4-naphtho[2',1':5,6][1,2] azaborepino [7,1-a]isoquinoline (43)

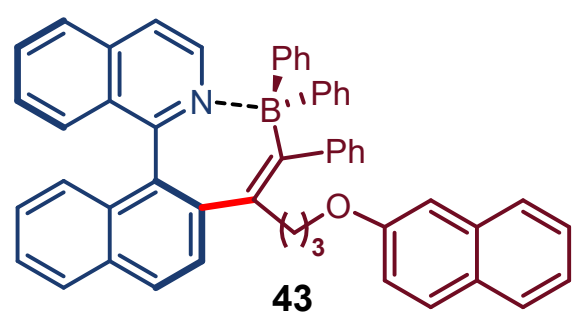

43

Prepared following **Procedure D**, using 1-(isoquinolin-1-yl)naphthalen-2-yl trifluoromethanesulfonate (80.6 mg, 0.2 mmol, 1.0 equiv.), tetramethylammonium [5-(naphthalen-2-yloxy)pent-1-yn-1-yl]triphenylborate (157.5 mg, 0.3 mmol, 1.5 equiv.), $Pd_2(dba)_3$ (9.16 mg, 0.001 mmol, 5.0 mol%) and

(*R*)-**L1** (18.3 mg, 0.03 mmol, 15 mol%). Purification by flash column chromatography (Petroleum ether/EtOAc: 50/1) to afford the title compound (74.7 mg, 53%) as a yellow solid.

TLC: R_f = 0.5 (Petroleum ether/EtOAc: 50/1, $KMnO_4$ stain).

M. p.: 89 – 90 °C.

$[\alpha]^{18}_D$: +343.26 (c 0.20, CH_2Cl_2).

NMR Spectroscopy (*see spectra*):

^{1}H NMR (400 MHz, $CDCl_3$) δ_H = 9.02 (d, *J* = 6.9 Hz, 1H), 7.82 –.78 (m, 2H), 7.73 (d, *J* = 7.3 Hz, 1H), 7.66 – 7.61 (m, 4H), 7.50 (d, *J* = 8.0 Hz, 1H), 7.44 – 7.40 (m, 3H), 7.37 – 7.30 (m, 2H), 7.24 (t, *J* = 1.6 Hz, 1H), 7.21 – 7.15 (m, 2H), 7.13 – 7.10 (m, 1H), 7.05 – 6.98 (m, 3H), 6.90 – 6.84 (m, 3H), 6.75 – 6.60 (m, 6H), 6.47 – 6.37 (m, 3H), 6.20 (t, *J* = 6.7 Hz, 1H), 3.53 (q, *J* = 8.3 Hz, 1H), 3.46 – 3.35 (m, 1H), 2.92 – 2.85 (m, 1H), 2.79 – 2.69 (m, 1H), 1.87 – 1.74 (m, 1H), 1.66 (t, *J* = 8.1 Hz, 1H) ppm;

^{13}C NMR (100 MHz, $CDCl_3$) δ_C = 158.3, 156.7, 149.3, 143.5, 142.9, 138.2, 138.1, 137.9, 136.2, 134.5, 132.9, 132.6, 131.7, 130.9, 130.7, 130.4, 130.3, 129.8, 129.2, 129.1, 129.1, 128.8, 128.5, 127.8, 127.8, 127.7, 127.4, 127.0, 126.8, 126.7, 126.5, 126.2, 126.0, 125.9, 125.5, 125.0, 124.5, 124.1, 123.9, 123.5, 122.9, 121.0, 118.9, 105.9, 66.9, 29.1, 28.7 ppm. The carbon attached to boron was not observed due to quadrupolar relaxation;

^{11}B NMR (128 MHz, $CDCl_3$) δ_B = 4.01 ppm.

IR (film): ν_{max} 3692, 3043, 2923, 1626, 1599, 1506, 1364, 1216, 1031, 748, 701 cm^{-1}.

HRMS (ESI): m/z calculated for $C_{52}H_{41}BNO^+$ $[M+H]^+$, 706.3276, found, 706.3285.

HPLC analysis: HPLC conditions: Chiral column IB, *n*-hexane/isopropanol: 99/1, flow rate = 1.0 mL/min, wavelength = 254 nm, t_R = 18.810 min for major isomer, t_R = 20.992 min for minor isomer, 86% ee.

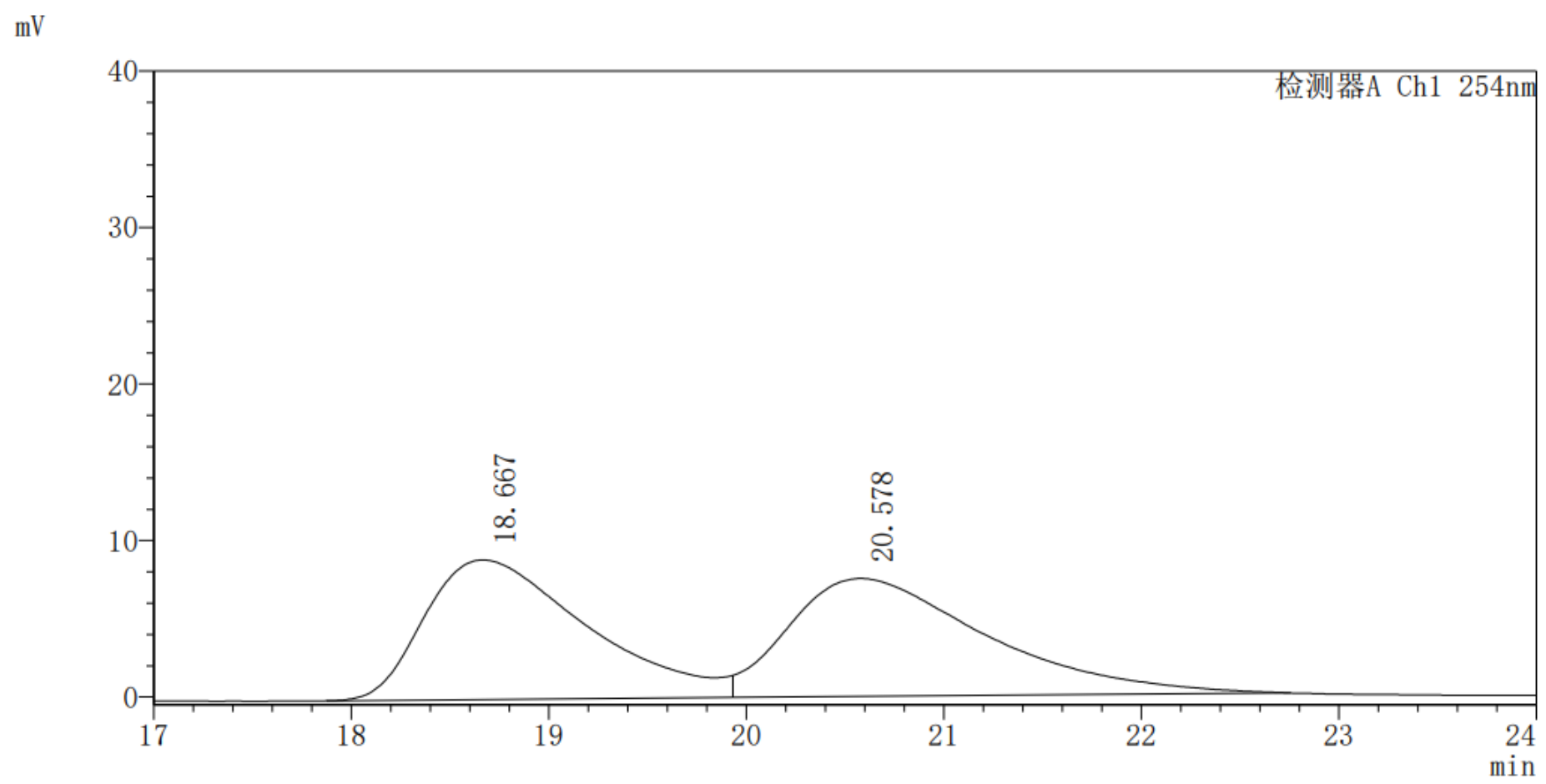

检测器A Ch1 254nm

No.	R.Time	Height	Height%	Area	Area%
1	18.667	8929	54.203	511313	49.383
2	20.578	7544	45.797	524096	50.617

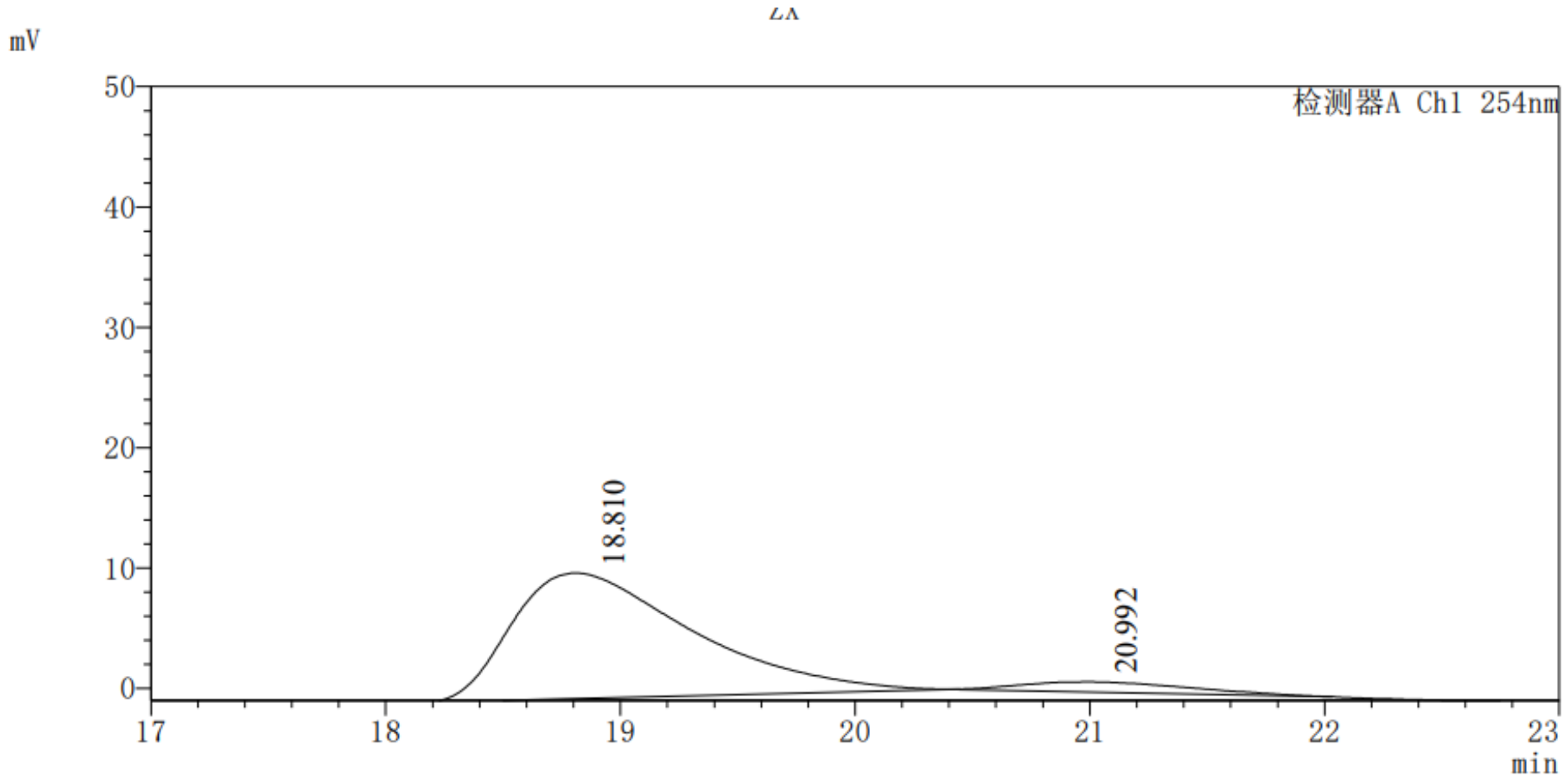

检测器A Ch1 254nm

No.	R.Time [min]	Height	Height%	Area	Area%
1	18.810	10454	92.507	559345	92.962
2	20.992	847	7.493	42348	7.038
总计		11301	100.000	601693	100.000

(*R*)-4,4,5-Triphenyl-6-[2-(*p*-tolylthio)ethyl]-4*H*-3l4,4l4-naphtho[2',1':5,6][1,2]azaborepino[7,1-a] isoquinoline (44)

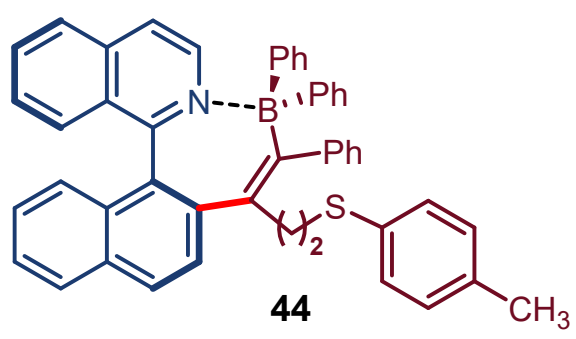

44

Prepared following **Procedure D**, using 1-(isoquinolin-1-yl)naphthalen-2-yl trifluoromethanesulfonate (80.6 mg, 0.2 mmol, 1.0 equiv.), tetramethylammonium triphenyl[4-(*p*-tolylthio)but-1-yn-1-yl]borate (147.3 mg, 0.3 mmol, 1.5 equiv.), $Pd_2(dba)_3$ (9.16 mg, 0.001 mmol, 5.0 mol%) and (*R*)-**L1** (18.3 mg,

0.03 mmol, 15 mol%). Purification by flash column chromatography (Petroleum ether/EtOAc: 50/1) to afford the title compound (110.0 mg, 82%) as a yellow solid.

TLC: R_f = 0.5 (Petroleum ether/EtOAc: 50/1, $KMnO_4$ stain).

M. p.: 86 – 87 °C.

$[\alpha]^{18}_D$: +387.66 (c 0.20, CH_2Cl_2).

NMR Spectroscopy (*see spectra*):

^{1}H NMR (400 MHz, $CDCl_3$) δ_H = 9.05 (d, J = 6.9 Hz, 1H), 8.02 (d, J = 8.1 Hz, 1H), 7.87 (d, J = 6.5 Hz, 1H), 7.82 (d, J = 8.1 Hz, 1H), 7.70 – 7.62 (m, 2H), 7.56 (d, J = 8.8 Hz, 1H), 7.50 – 7.43 (m, 2H), 7.37 – 7.27 (m, 2H), 7.19 – 7.16 (m, 1H), 7.13 – 7.02 (m, 3H), 6.99 – 6.90 (m, 5H), 6.81 (d, J = 8.1 Hz, 2H), 6.79 – 6.69 (m, 4H), 6.69-6.67 (m, 2H), 6.52 – 6.41 (m, 2H), 6.26 (t, J = 7.3 Hz, 1H), 3.04 (m, 1H), 2.96 – 2.86 (m, 1H), 2.85 – 2.75 (m, 1H), 2.66 (m, 1H), 2.31 (s, 3H) ppm;

^{13}C NMR (100 MHz, $CDCl_3$) δ_C = 158.3, 148.9, 143.5, 142.5, 138.0, 137.9, 137.1, 136.3, 135.2, 134.8, 132.9, 132.9, 132.5, 131.7, 130.9, 130.9, 130.8, 130.5, 129.9, 129.7, 129.4, 129.1, 129.1, 128.8, 128.7, 128.5, 127.7, 127.6, 127.3, 127.0, 126.9, 126.6, 126.0, 125.9, 125.5, 125.0, 124.5, 124.1, 123.7, 122.9, 121.0, 33.4, 32.7, 21.0 ppm. The carbon attached to boron was not observed due to quadrupolar relaxation;

^{11}B NMR (128 MHz, $CDCl_3$) δ_B = 3.65 ppm.

IR (film): ν_{max} 3668, 3010, 2890, 2758, 1735, 1490, 1303, 999, 824, 748, 703 cm^{-1}.

HRMS (ESI): m/z calculated for $C_{48}H_{39}BNS^+$ $[M+H]^+$, 672.2891, found, 672.2887.

HPLC analysis: HPLC conditions: Chiral column IB, *n*-hexane/isopropanol: 99/1, flow rate = 1.0 mL/min, wavelength = 254 nm, t_R = 10.305 min for major isomer, t_R = 13.503 min for minor isomer, 86% ee.

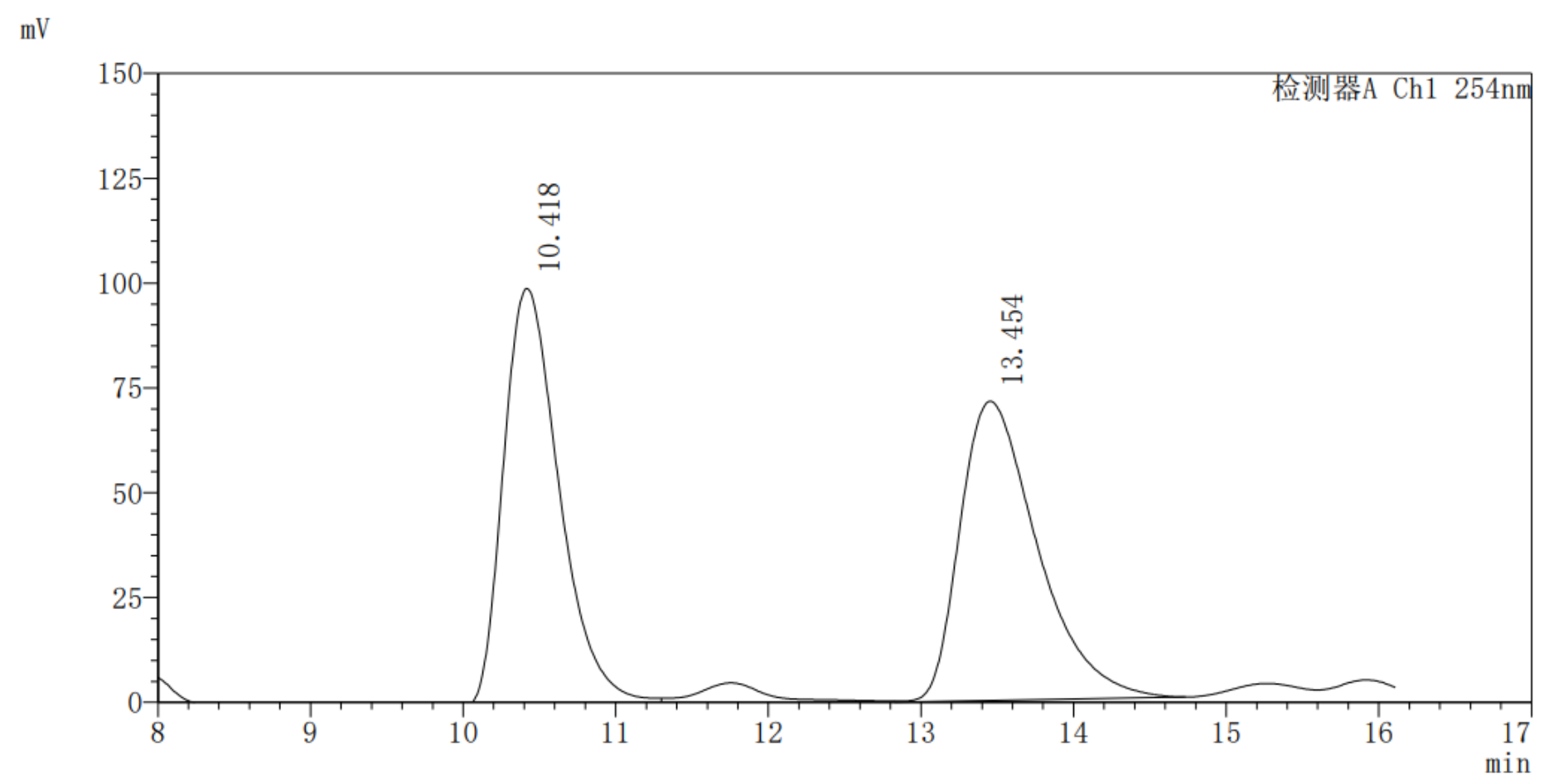

检测器A Ch1 254nm

No.	R.Time	Height	Height%	Area	Area%
1	10.418	100404	58.437	2603743	50.785
2	13.454	71411	41.563	2523277	49.215

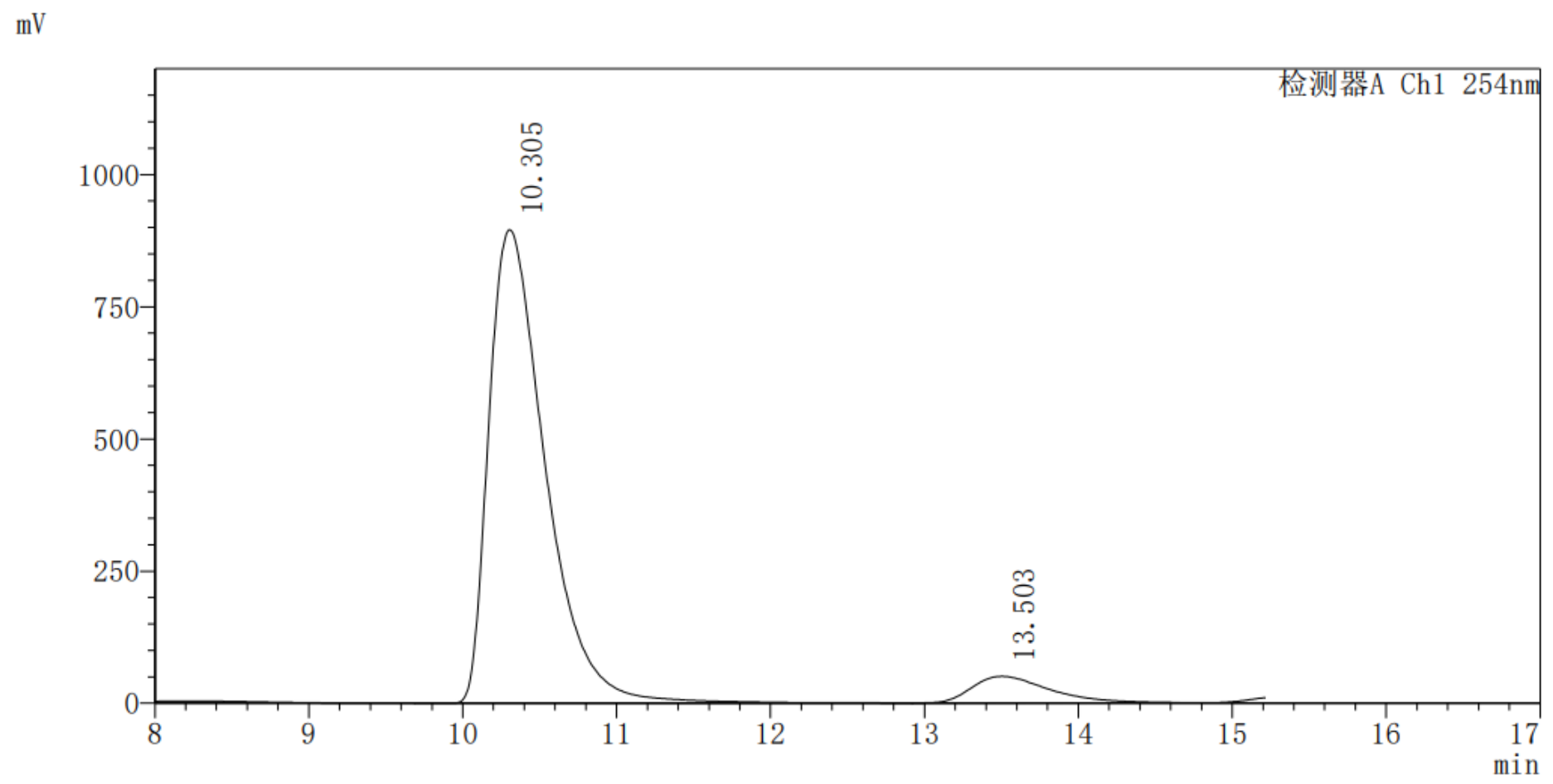

检测器A Ch1 254nm

No.	R.Time	Height	Height%	Area	Area%
1	10.305	896032	94.705	22899986	92.955
2	13.503	50098	5.295	1735582	7.045

(*R*)-6-[2-(Naphthalen-2-ylthio)ethyl]-4,4,5-triphenyl-4*H*-3l4,4l4-naphtho[2',1':5,6][1,2] azabore pino [7,1-a]isoquinoline (45)

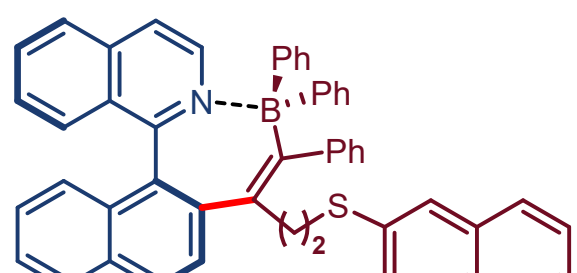

45

Prepared following **Procedure D**, using 1-(isoquinolin-1-yl)naphthalen-2-yl trifluoromethanesulfonate (80.6 mg, 0.2 mmol, 1.0 equiv.), tetramethylammonium [4-(naphthalen-2-ylthio)but-1-yn-1-yl]triphenylborate (158.1 mg, 0.3 mmol, 1.5 equiv.), $Pd_2(dba)_3$ (9.16 mg, 0.001 mmol, 5.0 mol%) and

(*R*)-**L1** (18.3 mg, 0.03 mmol, 15 mol%). Purification by flash column chromatography (Petroleum ether/EtOAc: 50/1) to afford the title compound (131.5 mg, 93%) as a yellow solid.

TLC: R_f = 0.5 (Petroleum ether/EtOAc: 50/1, $KMnO_4$ stain).

M. p.: 85 – 86 °C.

$[\alpha]^{18}_{D}$: +469.51 (c 0.20, CH_2Cl_2).

NMR Spectroscopy (*see spectra*):

^{1}H NMR (400 MHz, $CDCl_3$) δ_H = 9.06 (d, *J* = 6.8 Hz, 1H), 8.02 (d, *J* = 8.3 Hz, 1H), 7.88 (d, *J* = 6.9 Hz, 1H), 7.80 – 7.73 (m, 2H), 7.68 – 7.63 (m, 2H), 7.59 (d, *J* = 8.9 Hz, 3H), 7.50-7.39 (m, 3H), 7.31 – 7.25 (m, 2H), 7.23 (t, *J* = 11.6 Hz, 1H), 7.19 (d, *J* = 7.2 Hz, 1H), 7.08 (t, *J* = 7.8 Hz, 1H), 7.02 – 6.88 (m, 7H), 6.80 – 6.70 (m, 4H), 6.69-6.61 (m, 2H), 6.50 (d, *J* = 8.3 Hz, 1H), 6.45 (t, *J* = 7.3 Hz, 1H), 6.31 – 6.22 (m, 1H), 3.19 – 3.07 (m, 1H), 3.04 – 2.92 (m, 2H), 2.86 – 2.75 (m, 1H) ppm;.

^{13}C NMR (100 MHz, $CDCl_3$) δ_C = 158.3, 148.8, 142.4, 138.0, 137.9, 136.9, 136.3, 134.5, 133.7, 132.9, 132.6, 131.7, 131.4, 130.9, 130.8, 130.5, 130.1, 129.8, 129.1, 128.7, 128.0, 127.7, 127.7, 127.3, 127.0, 126.9, 126.8, 126.6, 126.5, 126.4, 126.0, 125.9, 125.3, 125.3, 125.0, 125.0, 124.5, 124.2, 123.6, 122.9, 121.0, 32.7, 32.1 ppm. The carbon attached to boron was not observed due to quadrupolar relaxation;

^{11}B NMR (128 MHz, $CDCl_3$) δ_B = 3.65 ppm.

IR (film): ν_{max} 3673, 2998, 2889, 2757, 1748, 1502, 1302, 998, 823, 742, 703 cm^{-1}.

HRMS (ESI): m/z calculated for $C_{51}H_{39}BNS^+$ $[M+H]^+$, 708.2891, found, 708.2899.

HPLC analysis: HPLC conditions: Chiral column IB, *n*-hexane/isopropanol: 99/1, flow rate = 1.0 mL/min, wavelength = 254 nm, t_R = 15.913 min for major isomer, t_R = 21.183 min for minor isomer, 85% ee.

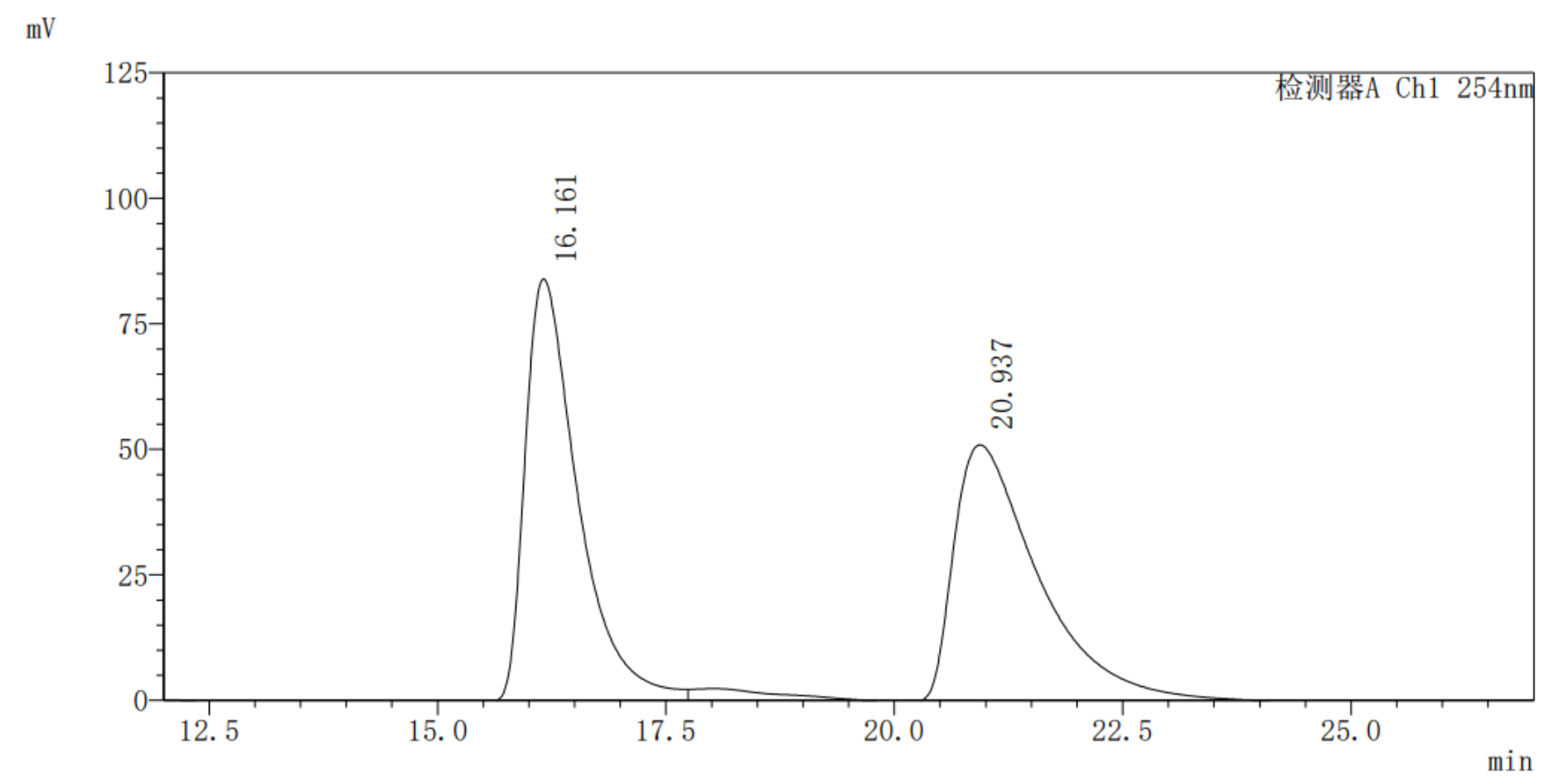

检测器A Ch1 254nm

No.	R.Time	Height	Height%	Area	Area%
1	16.161	84346	62.163	3424896	50.270
2	20.937	51339	37.837	3388045	49.730

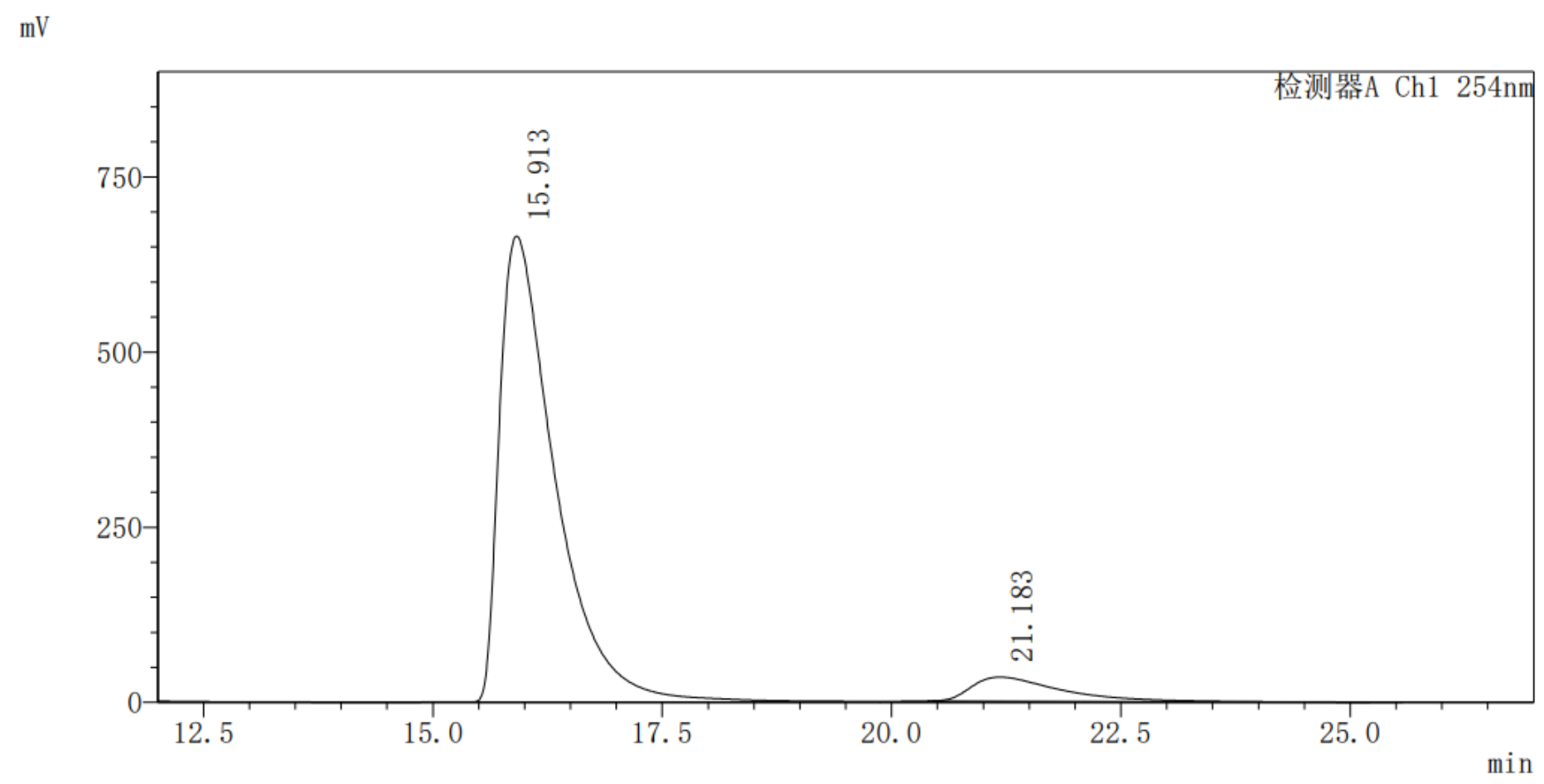

检测器A Ch1 254nm

No.	R.Time	Height	Height%	Area	Area%
1	15.913	666024	95.070	28400773	92.342
2	21.183	34539	4.930	2355188	7.658

(*R*)-4,4,5-Triphenyl-6-[2-(thiophen-2-ylthio)ethyl]-4*H*-3l4,4l4-naphtho[2',1':5,6][1,2]azaborepino [7,1-a]isoquinoline (46)

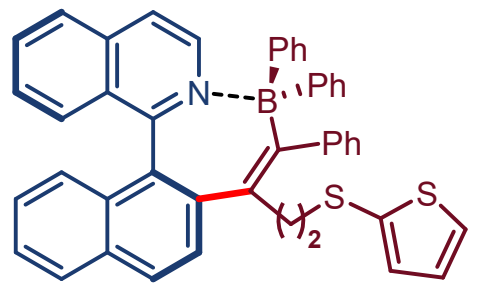

46

Prepared following **Procedure D**, using 1-(isoquinolin-1-yl)naphthalen-2-yl trifluoromethanesulfonate (80.6 mg, 0.2 mmol, 1.0 equiv.), tetramethylammonium triphenyl[4-(thiophen-2-ylthio)but-1-yn-1-

yl]borate (145.0 mg, 0.3 mmol, 1.5 equiv.), $Pd_2(dba)_3$ (9.16 mg, 0.001 mmol, 5.0 mol%) and (*R*)-**L1** (18.3 mg, 0.03 mmol, 15 mol%). Purification by flash column chromatography (Petroleum ether/EtOAc: 50/1) to afford the title compound (120.7 mg, 91%) as a yellow solid.

TLC: R_f = 0.5 (Petroleum ether/EtOAc: 50/1, $KMnO_4$ stain).

M. p.: 231 – 232 °C.

$[\alpha]^{18}_D$: +503.59 (c 0.20, CH_2Cl_2).

NMR Spectroscopy (*see spectra*):

^{1}H NMR (400 MHz, $CDCl_3$) δ_H = 8.88 (d, *J* = 7.0 Hz, 1H), 7.91 (d, *J* = 8.5 Hz, 1H), 7.76 – 7.68 (m, 2H), 7.49 (d, *J* = 8.6 Hz, 2H), 7.36 – 7.25 (m, 3H), 7.17 – 7.10 (m, 2H), 6.99 (d, *J* = 7.9 Hz, 1H), 6.93 (t, *J* = 7.8 Hz, 2H), 6.89 – 6.71 (m, 6H), 6.65 (s, 1H), 6.58 (d, *J* = 8.0 Hz, 3H), 6.53 (d, *J* = 7.5 Hz, 1H), 6.49 (d, *J* = 7.6 Hz, 1H), 6.43 (d, *J* = 7.9 Hz, 1H), 6.29 (d, *J* = 8.0 Hz, 2H), 6.10 (t, *J* = 7.8 Hz, 1H), 2.90 (t, *J* = 13.3 Hz, 1H), 2.76 (dt, *J* = 8.3, 14.4 Hz, 1H), 2.60 – 2.40 (m, 2H) ppm;

^{13}C NMR (100 MHz, $CDCl_3$) δ_C = 158.4, 148.8, 142.5, 138.1, 138.1, 136.9, 136.4, 134.8, 133.0, 132.6, 132.6, 131.8, 130.9, 130.6, 129.8, 129.8, 129.1, 128.9, 128.5, 127.7, 127.6, 127.4, 127.3, 127.1, 126.9, 126.6, 126.0, 126.0, 125.9, 125.0, 124.6, 124.2, 123.6, 123.0, 121.0, 38.6, 33.2 ppm. The carbon attached to boron was not observed due to quadrupolar relaxation;

^{11}B NMR (128 MHz, $CDCl_3$) δ_B = 3.97 ppm.

IR (film): ν_{max} 3606, 3050, 2999, 2900, 2767, 1713, 1487, 1408, 1272, 1163, 1129, 961, 705 cm^{-1}.

HRMS (ESI): m/z calculated for $C_{45}H_{35}BNS_2^+$ $[M+H]^+$, 664.2298, found, 664.2304.

HPLC analysis: HPLC conditions: Chiral column IB, *n*-hexane/isopropanol: 99/1, flow rate = 1.0 mL/min, wavelength = 254 nm, t_R = 11.958 min for major isomer, t_R = 15.180 min for minor isomer, 92% ee.

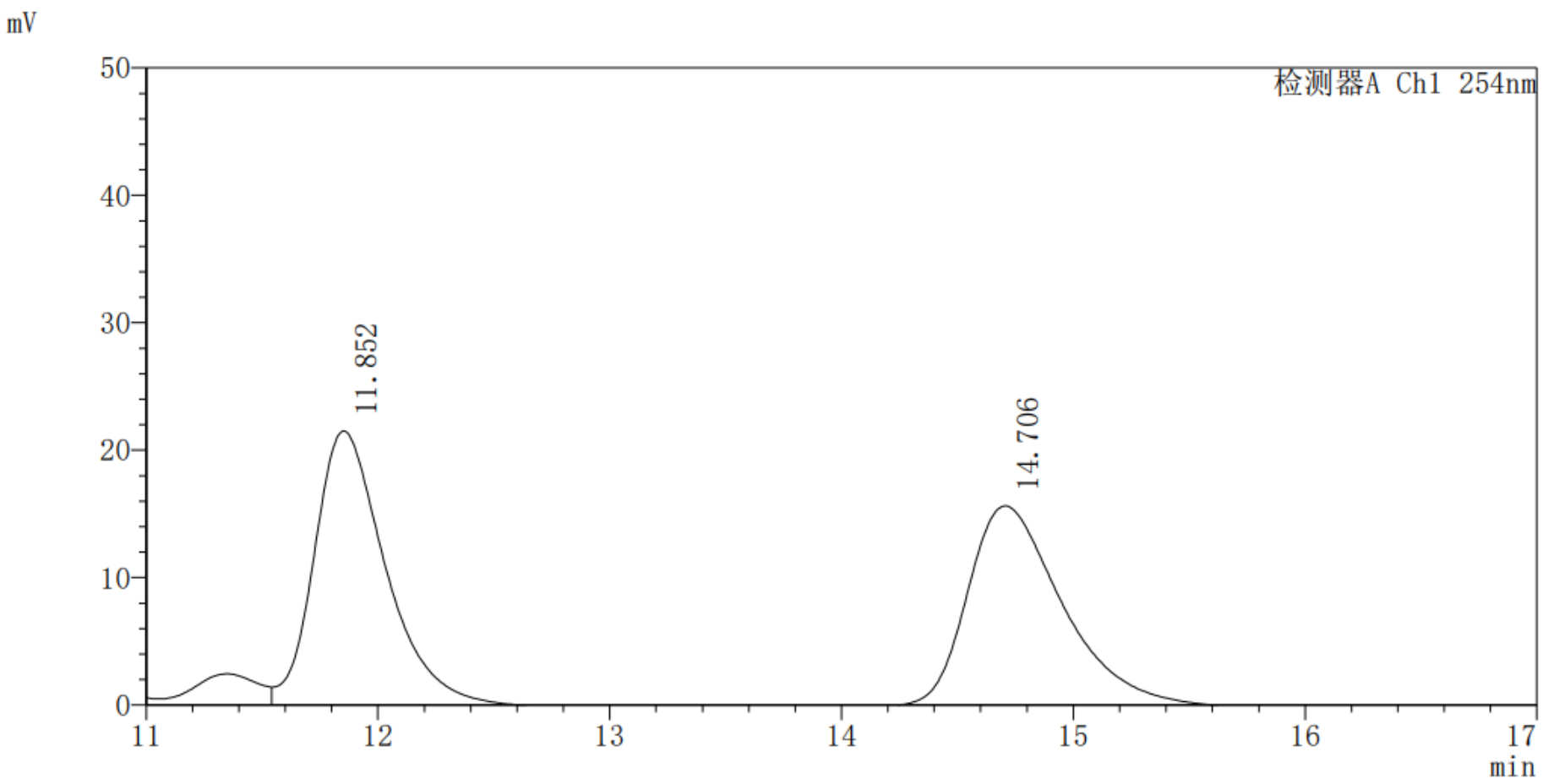

检测器A Ch1 254nm

No.	R.Time	Height	Height%	Area	Area%
1	11.852	21862	57.686	485205	50.195
2	14.706	16037	42.314	481434	49.805

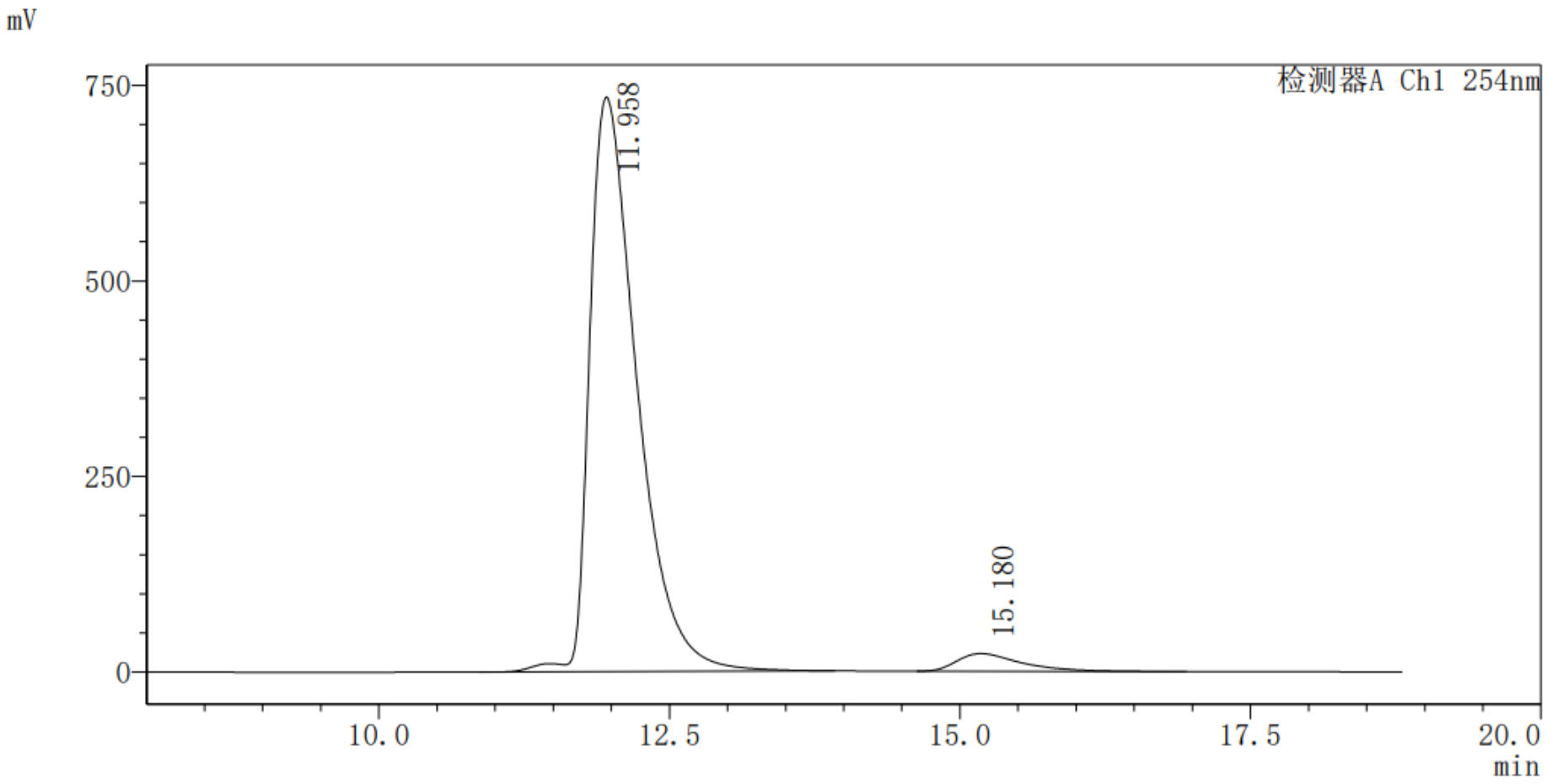

检测器A Ch1 254nm

No.	R.Time	Height	Height%	Area	Area%
1	11.958	734579	97.010	20778116	95.946
2	15.180	22639	2.990	878022	4.054

(*R*)-6-[3-(9*H*-Carbazol-9-yl)propyl]-4,4,5-triphenyl-4*H*-3l4,4l4-naphtho[2',1':5,6][1,2]azaborepino [7,1-a] isoquinoline (47)

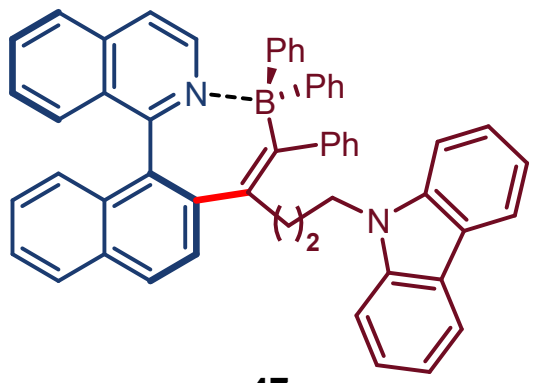

47

Prepared following **Procedure D**, using 1-(isoquinolin-1-yl)naphthalen-2-yl trifluoromethanesulfonate

(80.6 mg, 0.2 mmol, 1.0 equiv.), tetramethylammonium [5-(9*H*-carbazol-9-yl)pent-1-yn-1-yl]triphenylborate (164.5 mg, 0.3 mmol, 1.5 equiv.), $Pd_2(dba)_3$ (9.16 mg, 0.001 mmol, 5.0 mol%) and (*R*)-**L1** (18.3 mg, 0.03 mmol, 15 mol%). Purification by flash column chromatography (Petroleum ether/EtOAc: 50/1) to afford the title compound (100.4 mg, 69%) as a yellow solid.

TLC: R_f = 0.5 (Petroleum ether/EtOAc: 50/1, $KMnO_4$ stain).

M. p.: 89 – 90 °C.

$[\alpha]^{18}_D$: +386.90 (c 0.20, CH_2Cl_2).

NMR Spectroscopy (*see spectra*):

^{1}H NMR (400 MHz, $CDCl_3$) δ_H = 8.99 (d, *J* = 6.8 Hz, 1H), 8.06 (d, *J* = 7.7 Hz, 2H), 7.95 (d, *J* = 8.3 Hz, 1H), 7.81 (d, *J* = 6.9 Hz, 1H), 7.71 (t, *J* = 7.6 Hz, 1H), 7.61 (dd, *J* = 8.4, 12.3 Hz, 2H), 7.51 (d, *J* = 8.8 Hz, 1H), 7.33 (t, *J* = 7.4 Hz, 3H), 7.25 (d, *J* = 4.6 Hz, 1H), 7.20 (q, *J* = 8.5 Hz, 3H), 7.14 (d, *J* = 7.1 Hz, 1H), 7.09 – 7.00 (m, 5H), 6.96 (t, *J* = 7.4 Hz, 2H), 6.90 (t, *J* = 7.3 Hz, 2H), 6.76 – 6.67 (m, 4H), 6.62 (d, *J* = 7.7 Hz, 1H), 6.58 (d, *J* = 7.7 Hz, 1H), 6.44 (t, *J* = 7.3 Hz, 1H), 6.36 (d, *J* = 8.5 Hz, 1H), 6.25 (t, *J* = 7.4 Hz, 1H), 4.08 – 3.96 (m, 2H), 2.87 – 2.78 (m, 1H), 2.66-2.60 (m, 1H), 1.88 – 1.79 (m, 1H), 1.74 – 1.69 (m, 1H) ppm;

^{13}C NMR (100 MHz, $CDCl_3$) δ_C = 158.3, 149.4, 143.0, 140.2, 138.5, 138.2, 138.0, 136.3, 133.0, 132.6, 131.9, 130.9, 130.8, 130.5, 130.4, 129.8, 129.6, 129.2, 129.1, 128.9, 127.7, 127.5, 127.4, 127.1, 126.6, 126.6, 126.0, 125.9, 125.9, 125.5, 125.0, 124.9, 124.6, 124.1, 123.6, 123.0, 122.8, 121.0, 120.3, 118.7, 108.6, 43.1, 31.0, 29.6 ppm. The carbon attached to boron was not observed due to quadrupolar relaxation;

^{11}B NMR (160 MHz, $CDCl_3$) δ_B = 4.14 ppm.

IR (film): ν_{max} 3691, 3044, 2918, 1797, 1484, 1324, 1153, 909, 749, 702 cm^{-1}.

HRMS (ESI): m/z calculated for $C_{54}H_{42}BN_2^+$ $[M+H]^+$, 729.3436, found, 729.3439.

HPLC analysis: HPLC conditions: Chiral column IB, *n*-hexane/isopropanol: 99/1, flow rate = 1.0 mL/min, wavelength = 254 nm, t_R = 26.076 min for major isomer, t_R = 28.722 min for minor isomer, 94% ee.

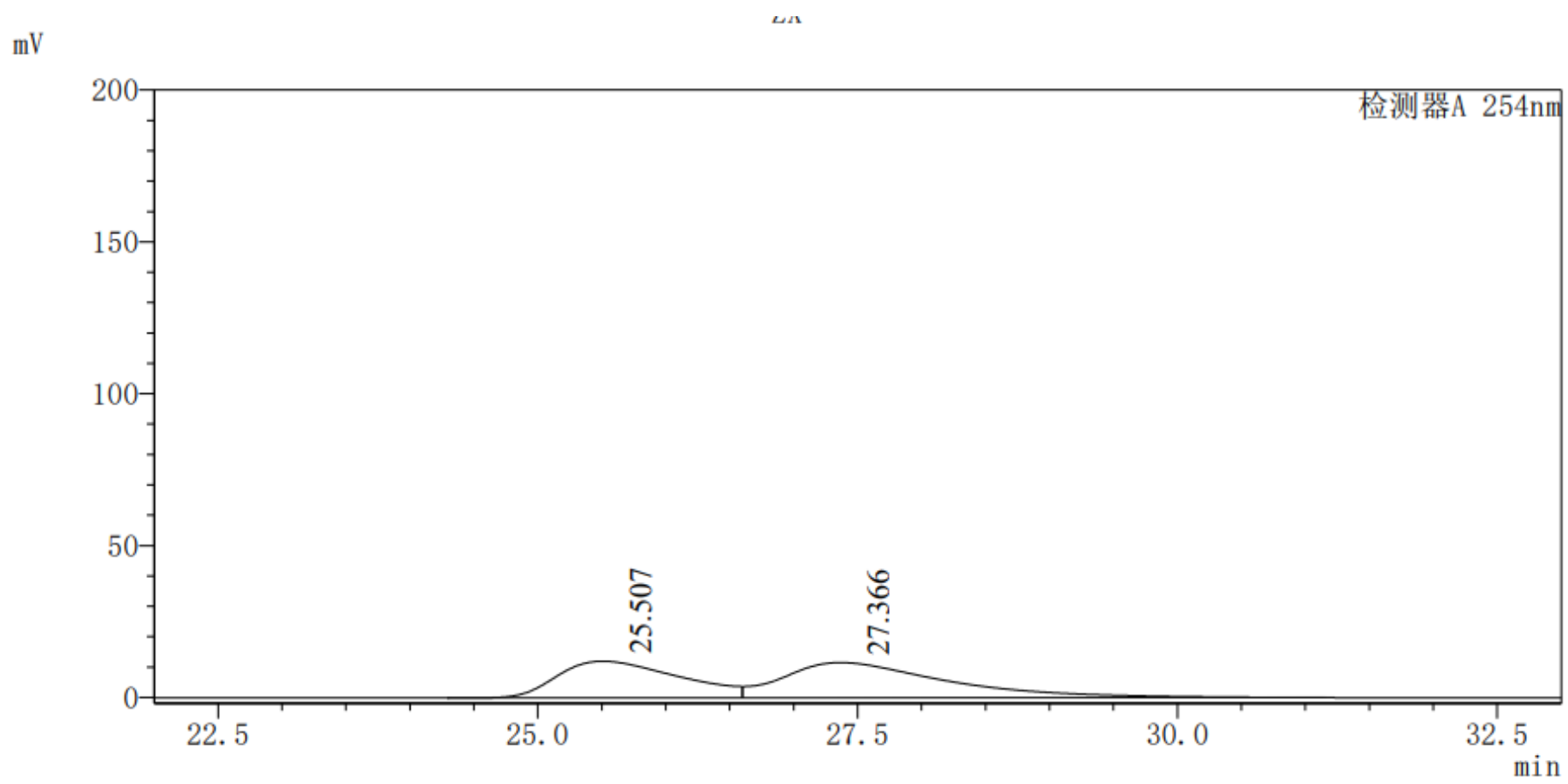

检测器A 254nm

No.	R.Time [min]	Height	Height%	Area	Area%
1	25.507	12087	50.991	803359	43.861
2	27.366	11617	49.009	1028252	56.139
总计		23703	100.000	1831611	100.000

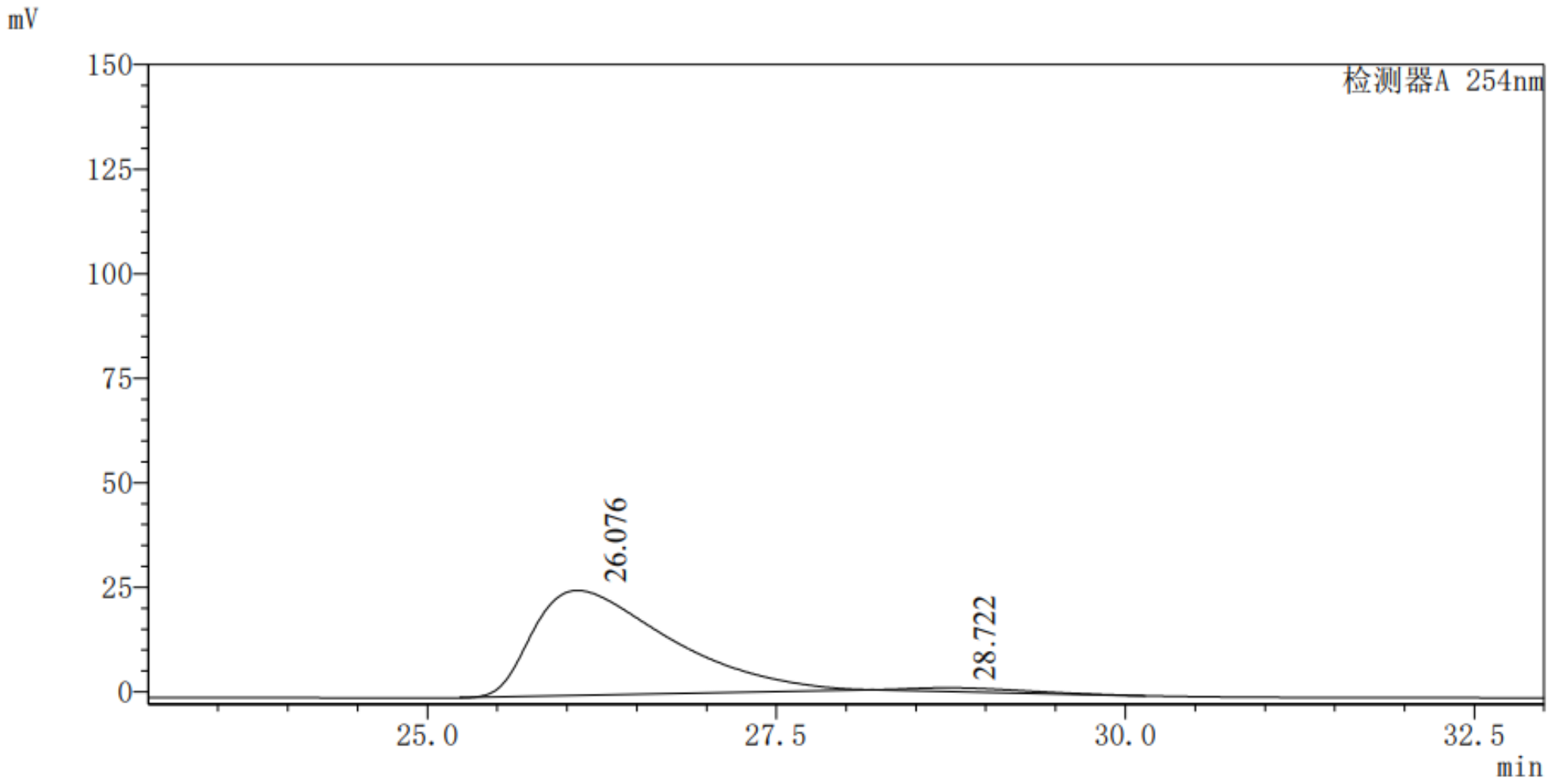

检测器A 254nm

No.	R.Time [min]	Height	Height%	Area	Area%
1	26.076	25125	96.636	1721368	97.119
2	28.722	875	3.364	51063	2.881
总计		25999	100.000	1772431	100.000

(*R,E*)-6-{[(3,7-Dimethylocta-2,6-dien-1-yl)oxy]methyl}-4,4,5-triphenyl-4*H*-3l4,4l4-naphtho [2',1':5,6][1,2] azaborepino [7,1-a] isoquinoline (48)

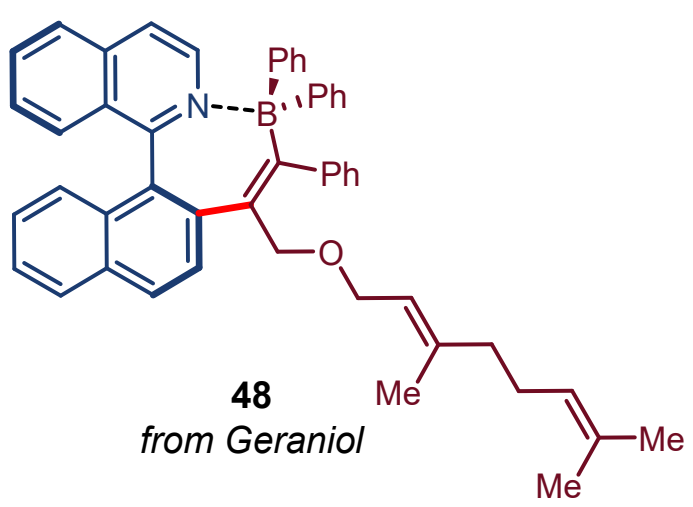

Prepared following **Procedure D**, using 1-(isoquinolin-1-yl)naphthalen-2-yl trifluoromethanesulfonate

(80.6 mg, 0.2 mmol, 1.0 equiv.), tetramethylammonium (*E*)-{3-[(3,7-dimethylocta-2,6-dien-1-yl)oxy]prop-1-yn-1-yl}triphenylborate (152.2 mg, 0.3 mmol, 1.5 equiv.), $Pd_2(dba)_3$ (9.16 mg, 0.001 mmol, 5.0 mol%) and (*R*)-**L1** (18.3 mg, 0.03 mmol, 15 mol%). Purification by flash column chromatography (Petroleum ether/EtOAc: 50/1) to afford the title compound (127.8 mg, 93%) as a yellow oil.

TLC: R_f = 0.4 (Petroleum ether/EtOAc: 20/1, $KMnO_4$ stain).

$[\alpha]^{18}_D$: +304.53 (c 0.20, CH_2Cl_2).

NMR Spectroscopy (*see spectra*):

^{1}H NMR (500 MHz, $CDCl_3$) δ_H = 9.03 (d, *J* = 6.7 Hz, 1H), 7.99 (d, *J* = 8.1 Hz, 1H), 7.85 (d, *J* = 6.9 Hz, 1H), 7.80 – 7.77 (t, *J* = 8.3 Hz, 1H), 7.76 – 7.71 (m, 1H), 7.65 (d, *J* = 9.0 Hz, 1H), 7.61 (d, *J* = 8.2 Hz, 1H), 7.46 – 7.46 (m, 1H), 7.36 (t, *J* = 8.5 Hz, 1H), 7.24 (t, *J* = 7.4 Hz, 1H), 7.15 – 7.08 (m, 2H), 7.04 – 6.97 (m, 2H), 6.95 – 6.89 (m, 2H), 6.86 (t, *J* = 6.4 Hz, 2H), 6.83 (d, *J* = 7.6 Hz, 1H), 6.76 – 6.71 (m, 2H), 6.69 – 6.65 (m, 1H), 6.63 (d, *J* = 8.1 Hz, 1H), 6.58 (d, *J* = 7.6 Hz, 1H), 6.40 (t, *J* = 7.4 Hz, 2H), 6.23 (t, *J* = 7.3 Hz, 1H), 5.05 – 4.93 (m, 2H), 4.67 (d, *J* = 12.4 Hz, 1H), 4.25 (d, *J* = 14.0 Hz, 1H), 3.67 – 3.49 (m, 2H), 1.96 (q, *J* = 7.2 Hz, 2H), 1.90 – 1.81 (m, 2H), 1.65 (s, 3H), 1.56 (s, 3H), 1.26 (s, 3H) ppm;

^{13}C NMR (126 MHz, $CDCl_3$) δ_C = 158.6, 148.3, 143.5, 142.5, 139.2, 138.1, 138.0, 136.9, 136.4, 133.0, 132.6, 131.9, 131.6, 131.2, 130.9, 130.7, 130.6, 129.6, 129.4, 129.3, 129.1, 128.8, 128.5, 127.7, 127.4, 127.3, 127.1, 126.7, 126.6, 126.1, 125.9, 125.7, 125.5, 125.1, 124.9, 124.6, 124.5, 124.3, 124.2, 123.0, 121.5, 121.0, 69.3, 65.7, 39.5, 26.4, 25.8, 17.8, 16.3 ppm. The carbon attached to boron was not observed due to quadrupolar relaxation;

^{11}B NMR (160 MHz, $CDCl_3$) δ_B = 3.61 ppm.

IR (film): ν_{max} 3060, 3045, 2997, 2964, 2914, 2852, 1621, 1596, 1553, 1429, 1062, 750, 702 cm^{-1}.

HRMS (ESI): m/z calculated for $C_{50}H_{47}BNO^+$ $[M+H]^+$ 688.3745, found 688.3737.

HPLC analysis: HPLC conditions: Chiral column IB, *n*-hexane/isopropanol: 99/1, flow rate = 0.4 mL/min, wavelength = 254 nm, t_R = 15.261 min for major isomer, t_R = 21.685 min for minor isomer, 96% ee.

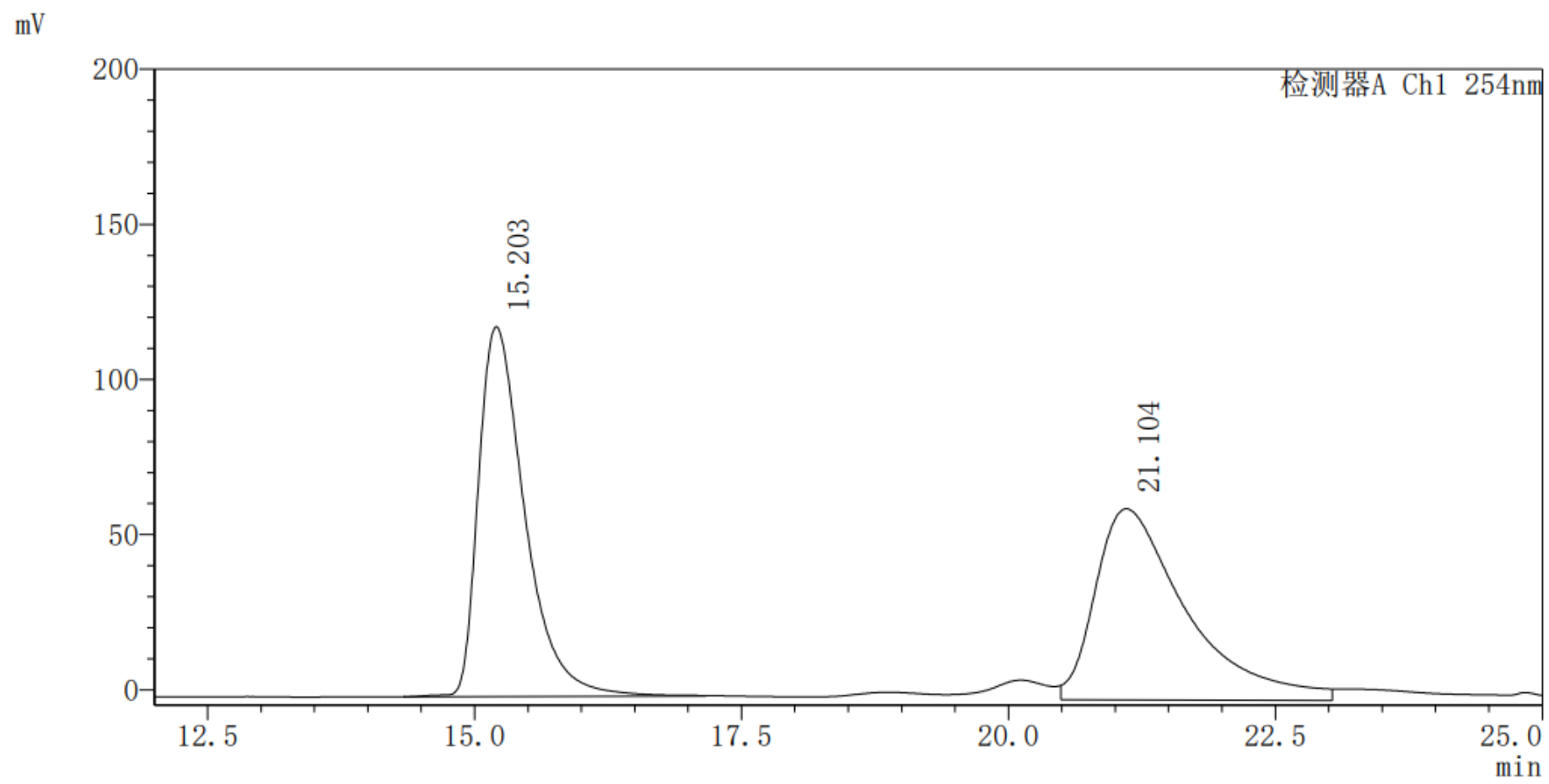

检测器A Ch1 254nm

No.	R.Time	Height	Height%	Area	Area%
1	15.203	119216	65.935	3609638	49.611
2	21.104	61593	34.065	3666268	50.389

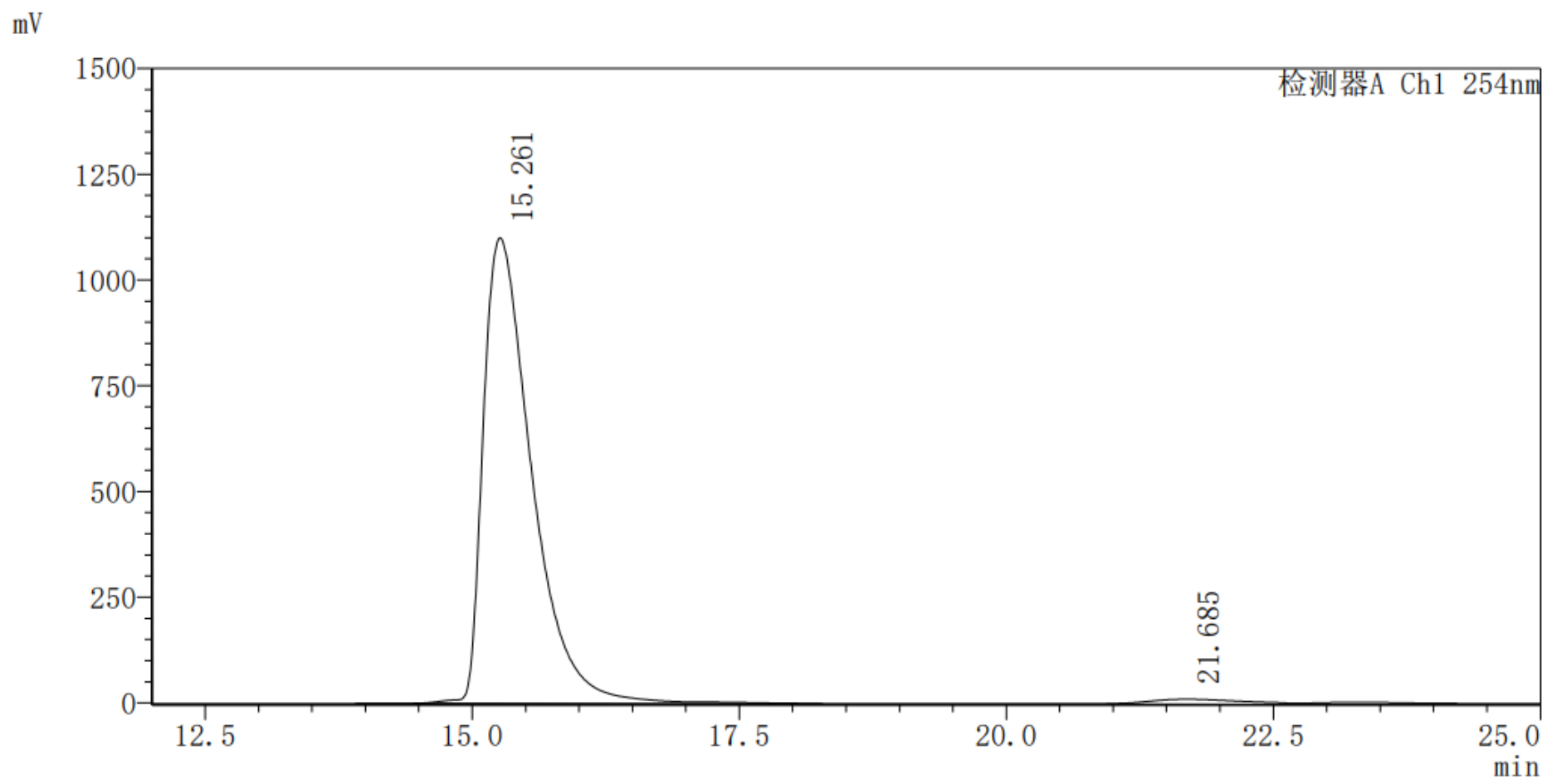

检测器A Ch1 254nm

No.	R.Time	Height	Height%	Area	Area%
1	15.261	1100429	98.929	34831616	97.966
2	21.685	11917	1.071	723011	2.034

(1*R*,5*R*,12*c*'*S*)-6'-Butyl-5'-phenyl-3'l4-aza-9l4-boraspiro[bicyclo[3.3.1]nonane-9,4'-naphtho [2',1':5,6] [1,2]azaborepino[7,1-a]isoquinoline] (49)

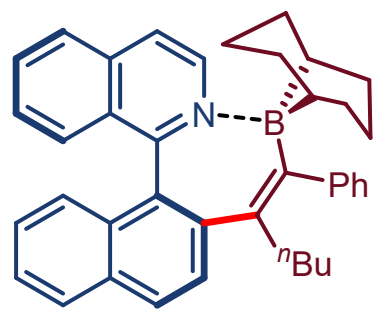

49

Prepared following **Procedure E**, using 1-(isoquinolin-1-yl)naphthalen-2-yl trifluoromethanesulfonate (80.6 mg, 0.2 mmol, 1.0 equiv.), lithium 9-(hex-1-yn-1-yl)-9-phenyl-9-borabicyclo[3.3.1]nonan-9-uide (85.5 mg, 0.3 mmol, 1.5 equiv.), $Pd_2(dba)_3$ (9.16 mg, 0.001 mmol, 5.0 mol%) and (*R*)-**L1** (18.3 mg, 0.03 mmol, 15 mol%). Purification by flash column chromatography (Petroleum ether/EtOAc: 50/1) to afford the title compound (38.4 mg, 36%) as a yellow solid.

TLC: R_f = 0.5 (Petroleum ether/EtOAc: 20/1, $KMnO_4$ stain).

M. p.: 254 – 255 °C.

$[\alpha]^{18}_D$: +1045.47 (c 0.20, CH_2Cl_2).

NMR Spectroscopy (*see spectra*):

^{1}H NMR (500 MHz, $CDCl_3$) δ_H = 8.96 (d, *J* = 6.9 Hz, 1H), 8.06 – 7.90 (m, 4H), 7.80 (d, *J* = 8.5 Hz, 1H), 7.72 (t, *J* = 7.6 Hz, 1H), 7.49 – 7.39 (m, 2H), 7.35 (t, *J* = 7.8 Hz, 1H), 7.24 (d, *J* = 6.9 Hz, 2H), 7.20 – 7.05 (m, 4H), 6.47 (d, *J* = 7.6 Hz, 1H), 2.48 (ddd, *J* = 5.0, 6.7, 14.0 Hz, 1H), 2.16 (ddd, *J* = 6.6, 9.4, 13.9 Hz, 2H), 1.71 – 1.52 (m, 4H), 1.41 (s, 1H), 1.26 – 1.04 (m, 6H), 1.01 – 0.88 (m, 2H), 0.76 – 0.67 (m, 1H), 0.50 – 0.40 (m, 1H), 0.29 – 0.17 (m, 1H), -0.06 (d, *J* = 4.0 Hz, 3H), -0.25 (s, 1H) ppm;

^{13}C NMR (126 MHz, $CDCl_3$) δ_C = 156.8, 149.0, 144.0, 136.8, 136.1, 135.8, 132.4, 132.2, 130.9, 130.4, 129.7, 129.7, 129.5, 129.3, 128.5, 128.4, 127.3, 127.1, 126.7, 126.5, 126.2, 125.3, 125.0, 123.8, 122.2, 33.6, 32.0, 31.5, 31.0, 24.2, 23.1, 21.3, 13.4. ppm. The carbon attached to boron was not observed due to quadrupolar relaxation;

^{11}B NMR (128 MHz, $CDCl_3$) δ_B = 3.31. ppm.

IR (film): ν_{max} 2953, 2918, 2892, 2860, 2823, 1625, 1593, 1556, 1328, 1216, 820, 756, 703 cm^{-1}.

HRMS (ESI): m/z calculated for $C_{39}H_{41}BN^{+}$ $[M+H]^{+}$ 534.3327, found 534.3325.

HPLC analysis: HPLC conditions: Chiral column IB, *n*-hexane/isopropanol: 99/1, flow rate = 1.0mL/min, wavelength = 254 nm, t_R = 5.636 min for major isomer, t_R = 6.315 min for minor isomer, 93% ee.

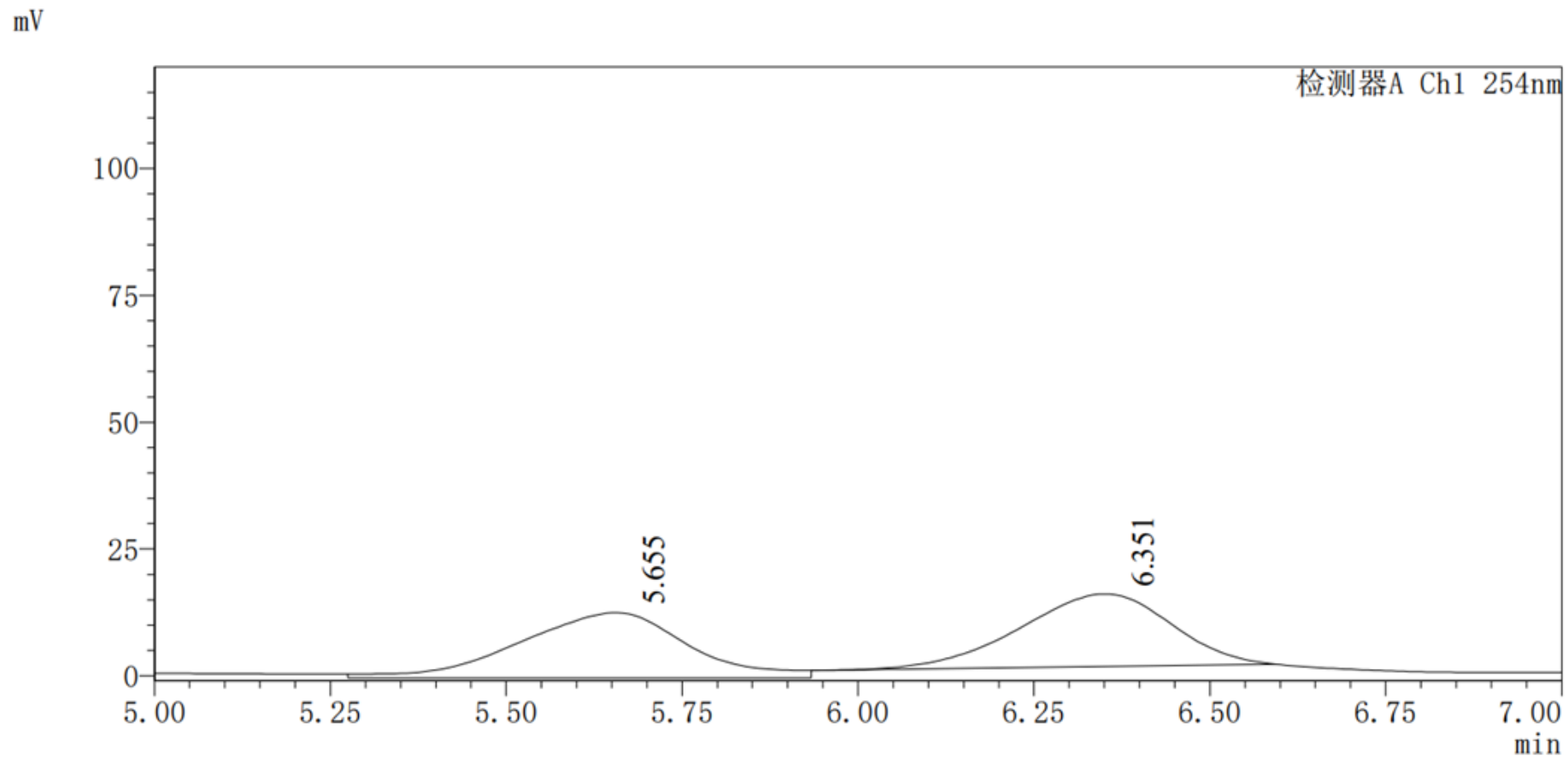

检测器A Ch1 254nm

No.	R.Time [min]	Height	Height%	Area	Area%
1	5.655	12845	47.333	213339	50.058
2	6.351	14292	52.667	212849	49.942
总计		27137	100.000	426188	100.000

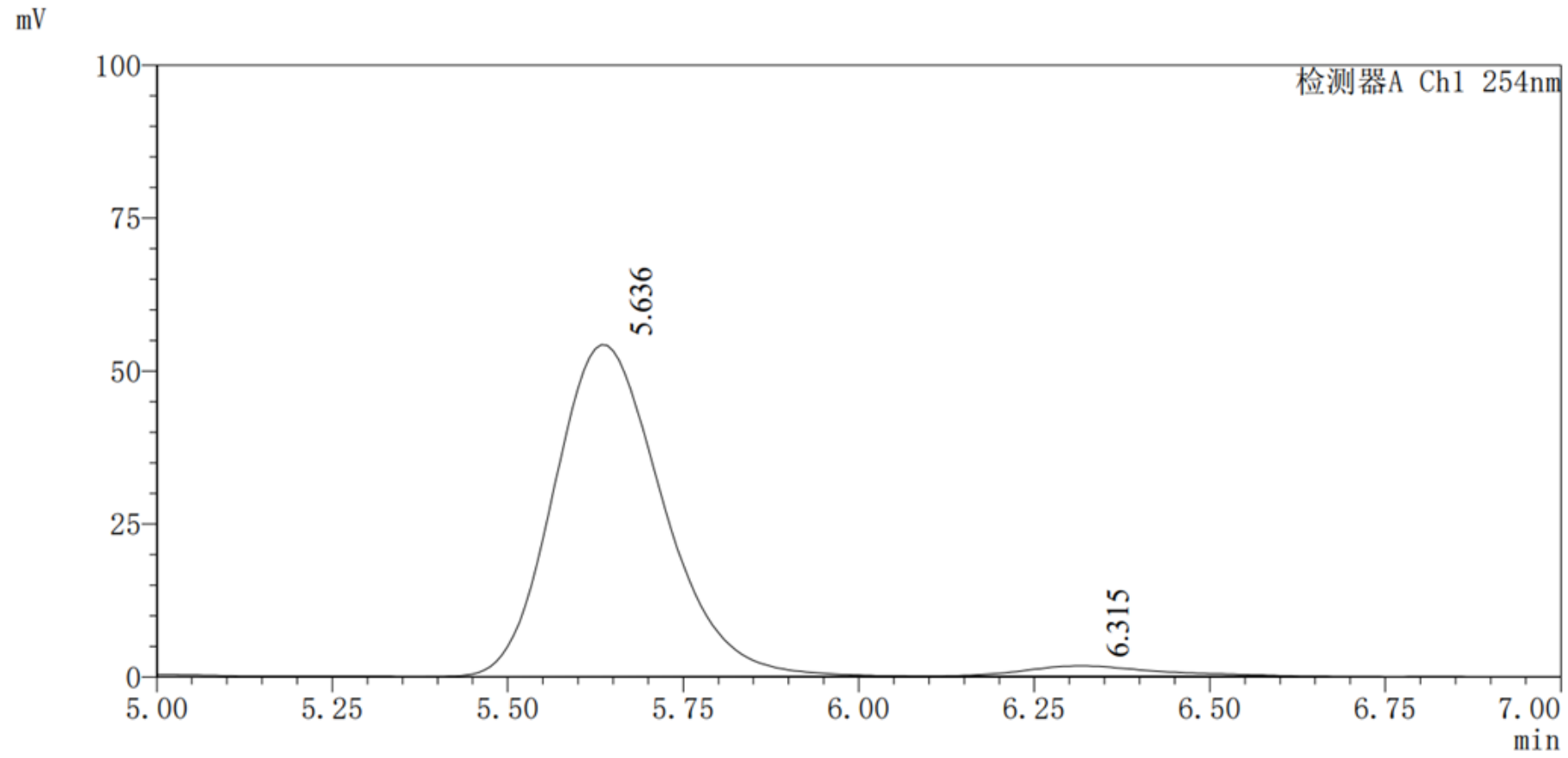

检测器A Ch1 254nm

No.	R.Time [min]	Height	Height%	Area	Area%
1	5.636	54273	97.019	586167	96.458
2	6.315	1668	2.981	21526	3.542
总计		55941	100.000	607693	100.000

3. PHOTOPHYSICAL PROPERTY STUDIES

*Structure of **3** and **43***

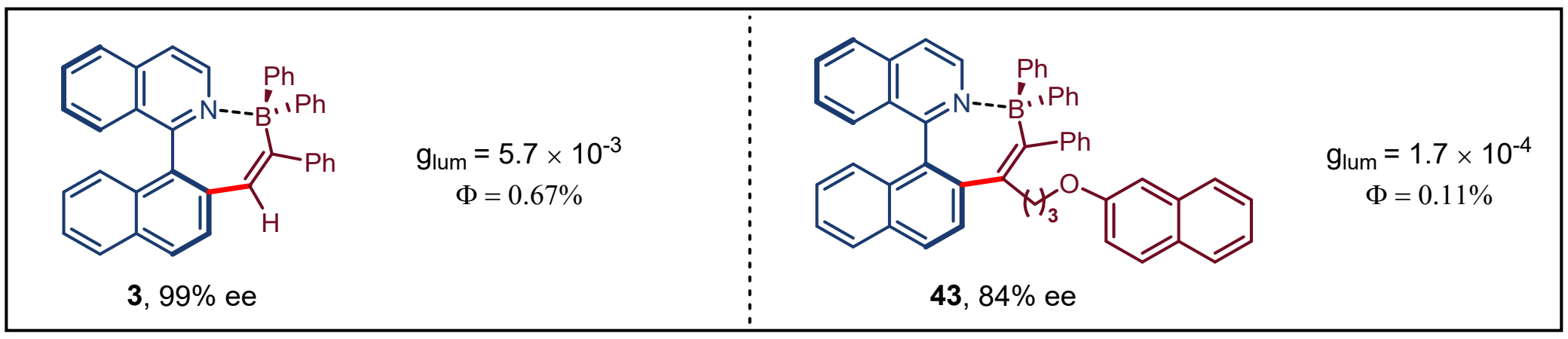

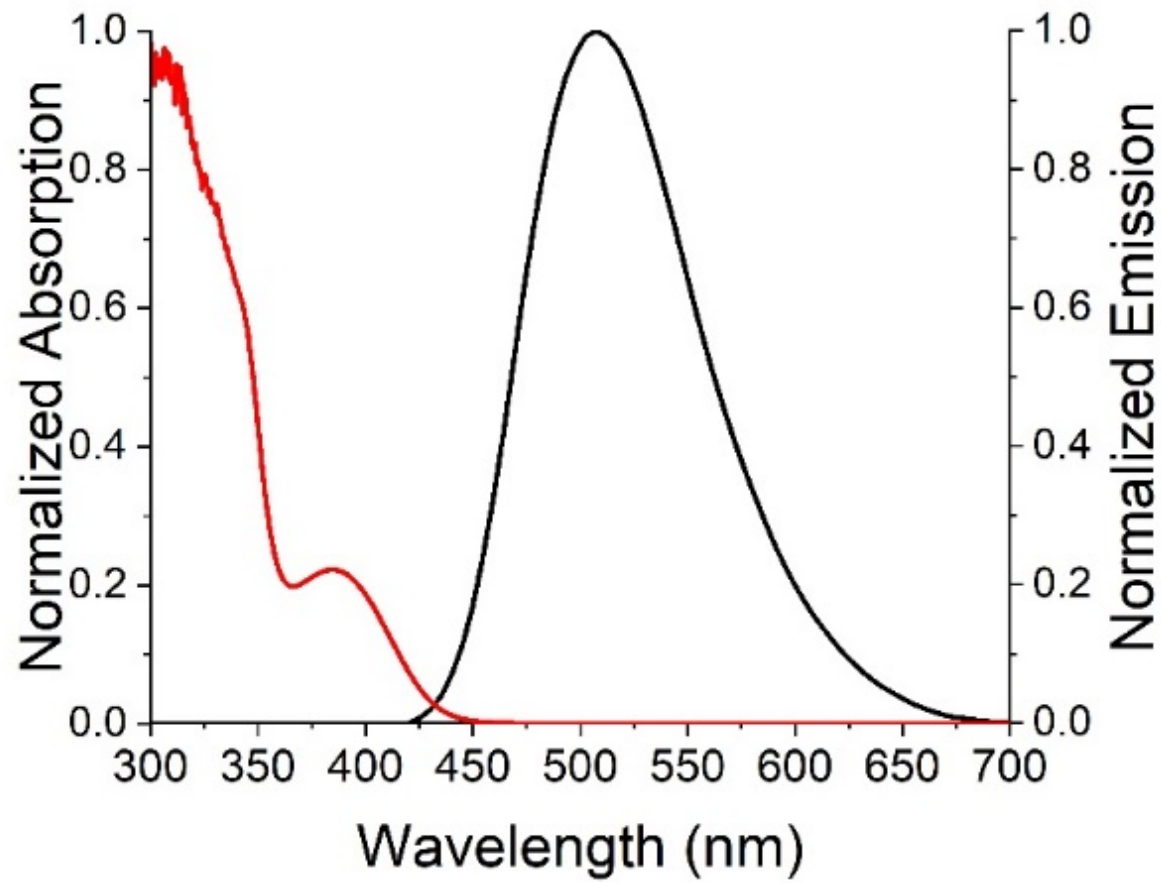

Figure S1. Absorption and fluorescence spectra of **3** in CH_3CN (4 × 10^{-4} M).

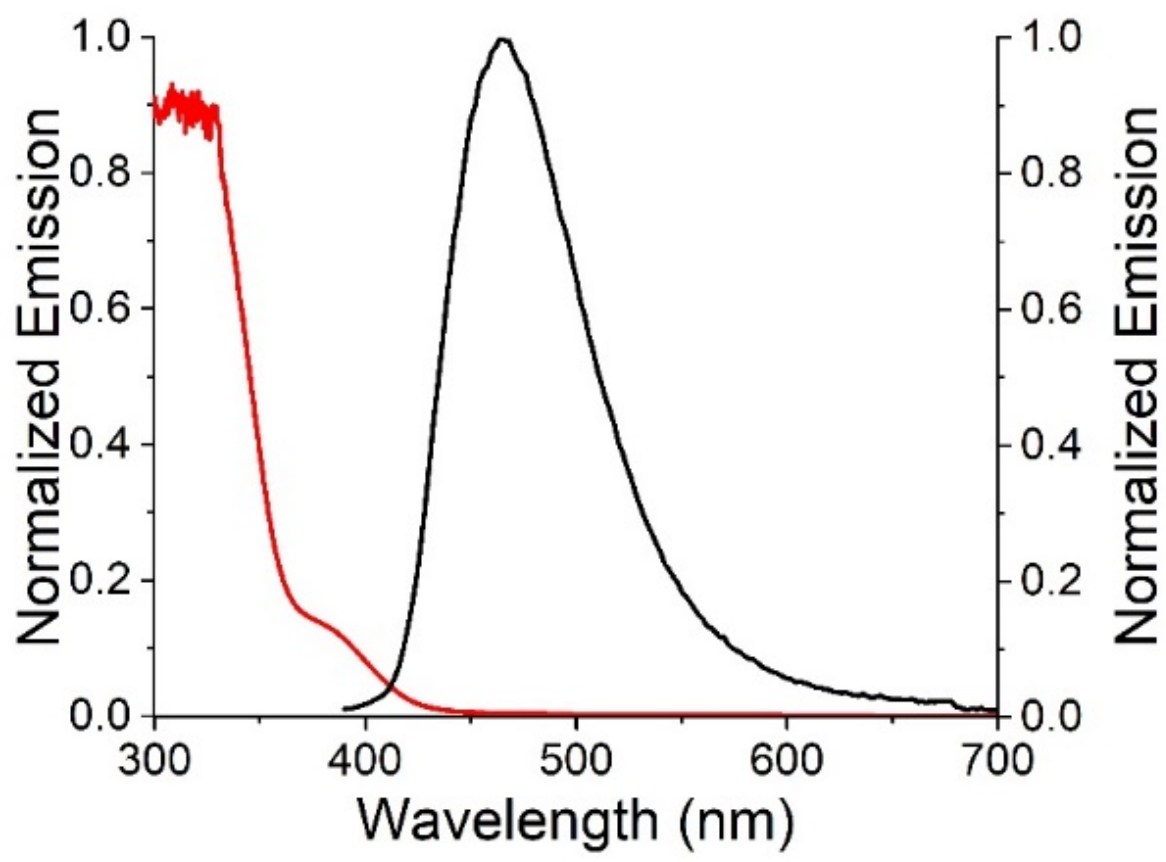

Figure S2. Absorption and fluorescence spectra of **43** in CH_3CN (4 × 10^{-4} M).

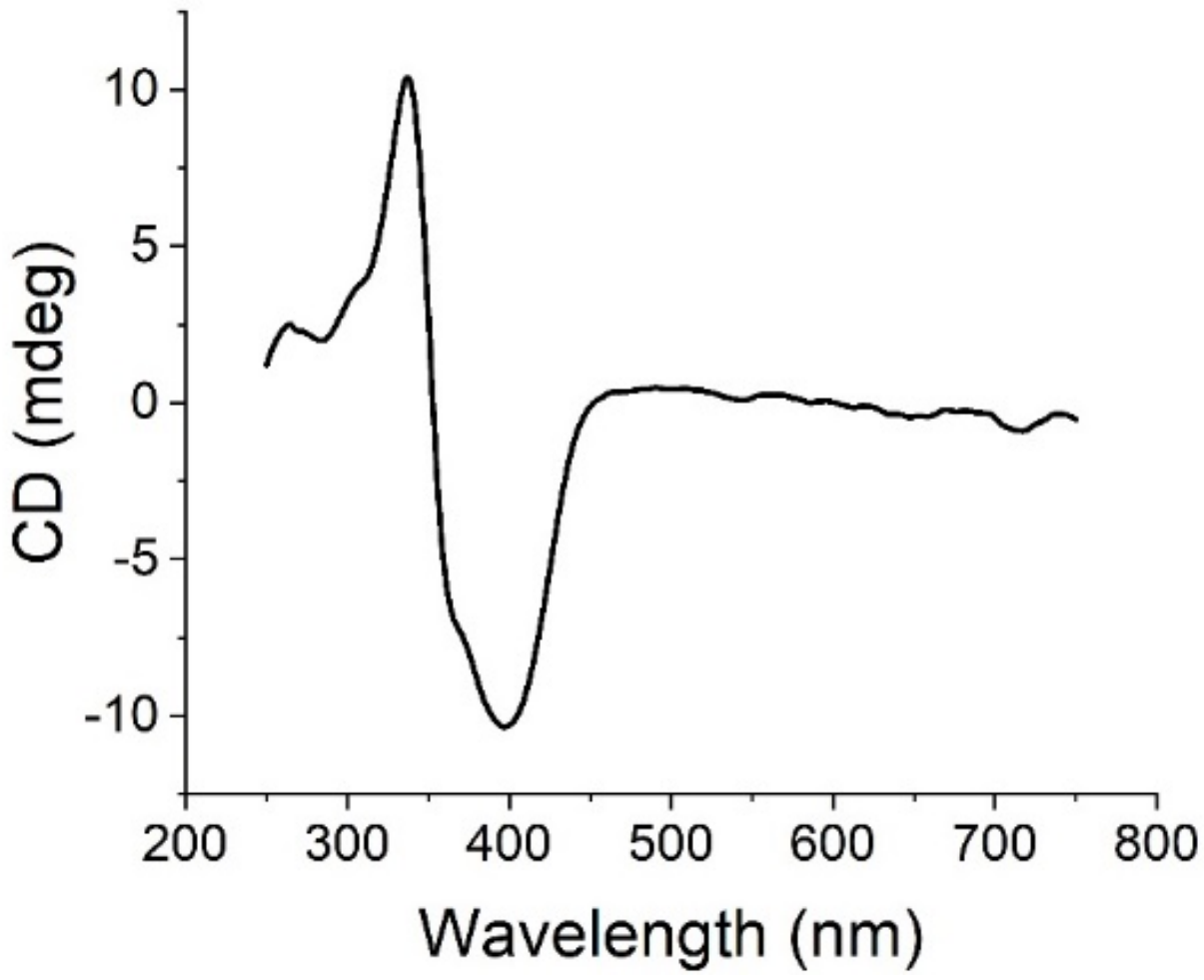

Figure S3. CD spectra of **3** in CH_3CN (2×10^{-4} M)

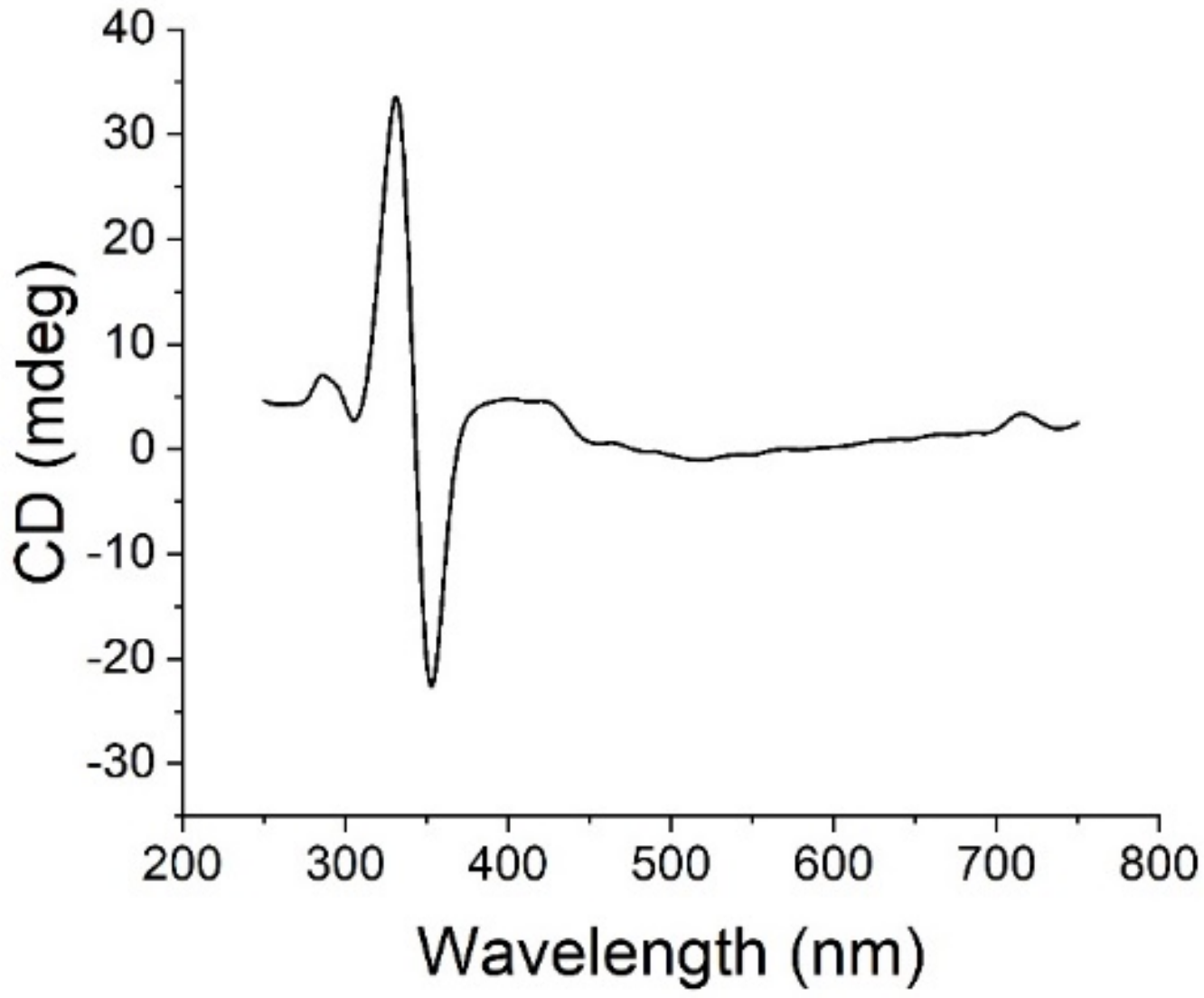

Figure S4. CD spectra of **43** in CH_3CN (2×10^{-4} M).

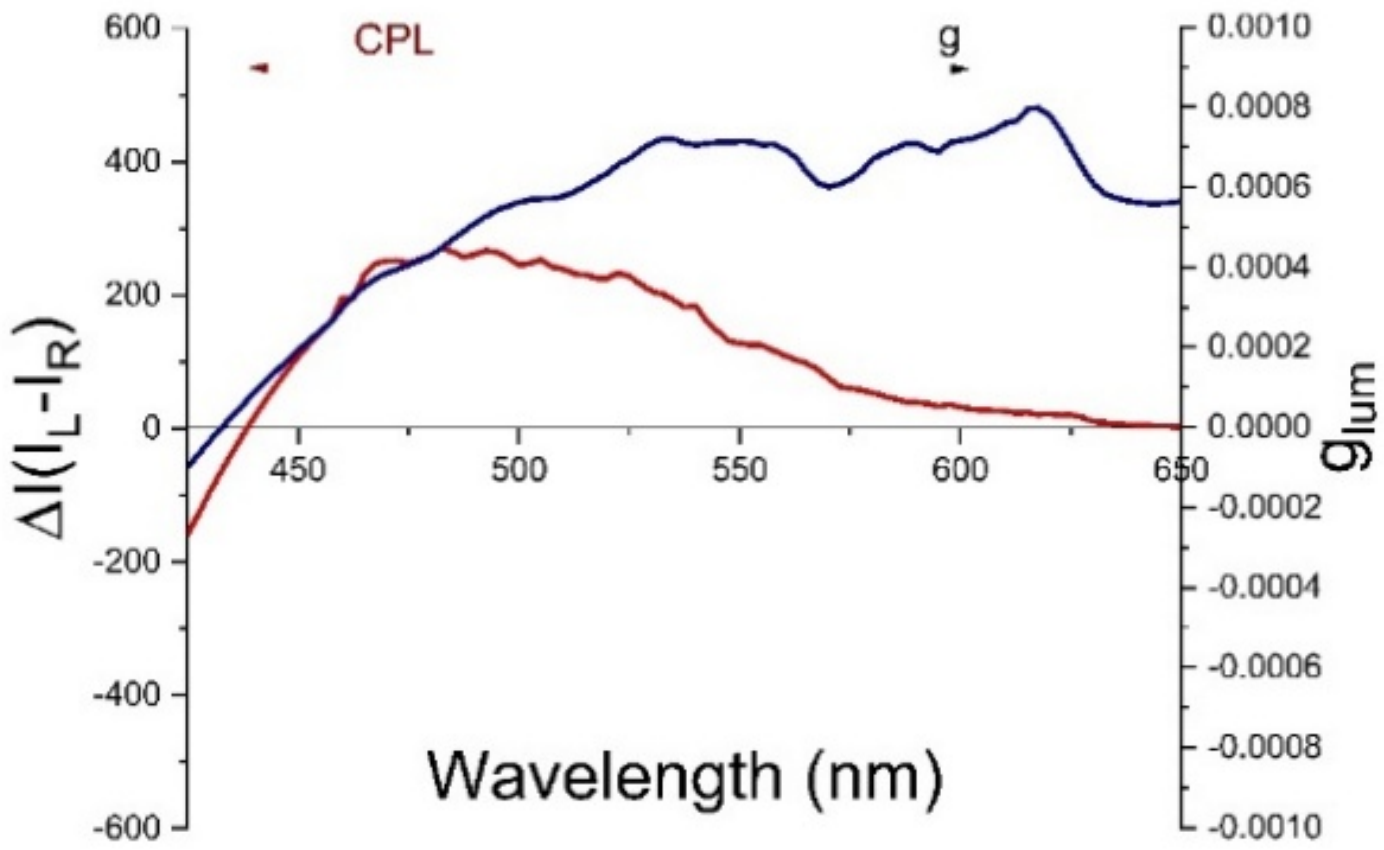

Figure S5. CPL spectra and glum values-wavelength curve of **3** in CH_3CN (2×10^{-4} M).

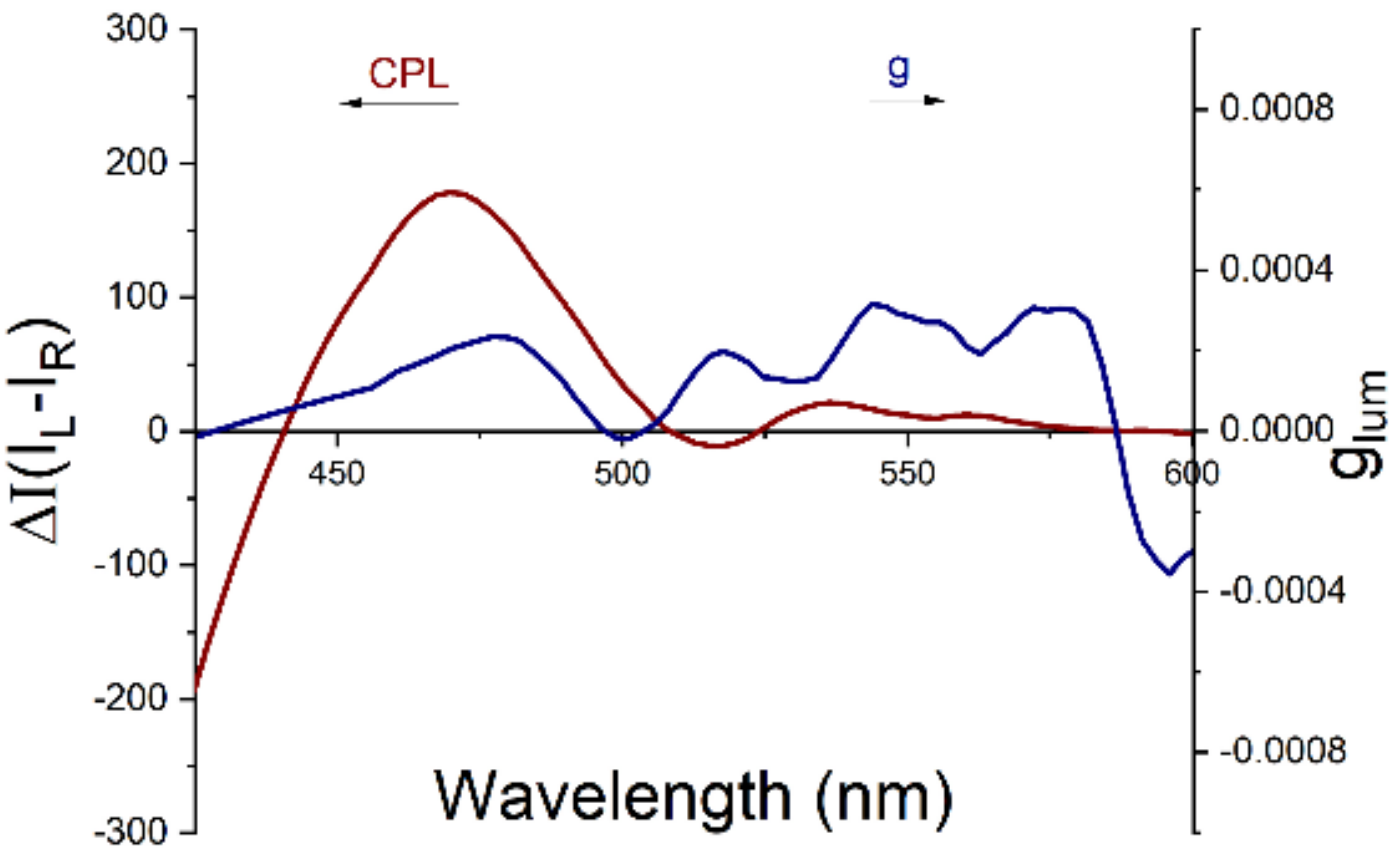

Figure S6. CPL spectra and glum values-wavelength curve of **43** in CH_3CN (2×10^{-4} M).

4. MECHANISTIC EXPERIMENTS

4.1 Control Experiments

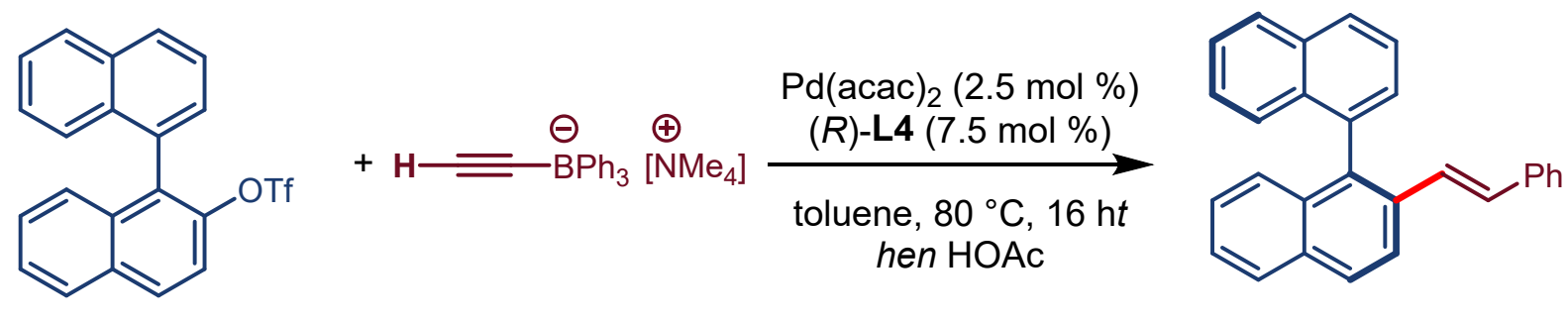

50 **2a** **51**, n.d.

In glovebox, to an oven-dried 8.0 mL vial equipped with a magnetic stir bar was added Pd(acac)$_2$ (0.005 mmol, 2.5 mol%, 1.5 mg) and (*R*)-**L4** (0.0 mmol, 7.5 mol%, 11.0 mg) under N_2 at room temperature. Anhydrous toluene (2.0 mL) was added and the mixture was stirred for 30 min at the same temperature to afford stock solution. [1,1'-Binaphthalen]-2-yl trifluoromethanesulfonate[11](0.2 mmol, 1.0 equiv., 80.4 mg) and tetramethylammonium ethynyltriphenylborate (0.3 mmol, 1.5 equiv., 102.4 mg) were added into a 10 mL Schlenk tube and the stock solution was transferred to this tube. After that, the Schlenk tube was sealed with a septum, removed from the glovebox, and was heated to 80 °C in an oil bath and stirred for 16 h at this temperature. No product was obtained through TLC detection.

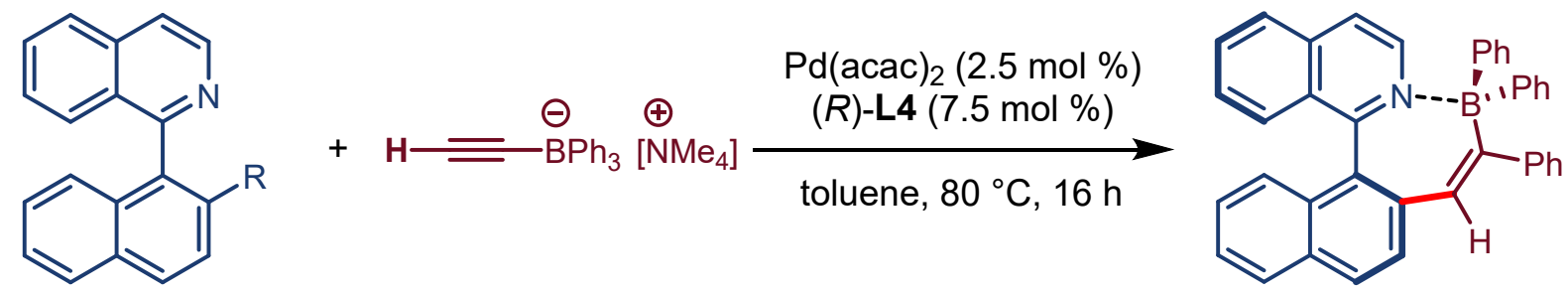

1 (*racemic*) **2a** **3**

R	3
OH	n.d.
Br	90%, 96%ee
ONf	95%, 80% ee

In glovebox, to an oven-dried 8.0 mL vial equipped with a magnetic stir bar was added Pd(acac)$_2$ (0.005 mmol, 2.5 mol%, 1.5 mg) and (*R*)-**L4** (0.0 mmol, 7.5 mol%, 11.0 mg) under N_2 at room temperature. Anhydrous toluene (2.0 mL) was added and the mixture was stirred for 30 min at the same temperature to afford stock solution. 1-(Isoquinolin-1-yl)naphthalen-2-ol (0.2 mmol, 1.0 equiv., 54.2 mg) or 1-(2-bromonaphthalen-1-yl)isoquinoline[12] (0.2 mmol, 1.0 equiv., 66.6 mg) or 1-(isoquinolin-1-yl)naphthalen-2-yl 1,1,2,2,3,3,4,4,4-nonafluorobutane-1-sulfonate[13] (0.2 mmol, 1.0 equiv., 110.6 mg) and tetramethylammonium ethynyltriphenylborate (0.3 mmol, 1.5 equiv., 102.4 mg) were added into a

10 mL Schlenk tube and the stock solution was then transferred to this tube. Finally, the Schlenk tube was sealed with a septum, removed from the glovebox, and was heated to 80 °C in an oil bath, and stirred for 16 h at this temperature.

When R= OH, no product was obtained through TLC detection.

When R = Br, the yield is 90% and ee is 96%.

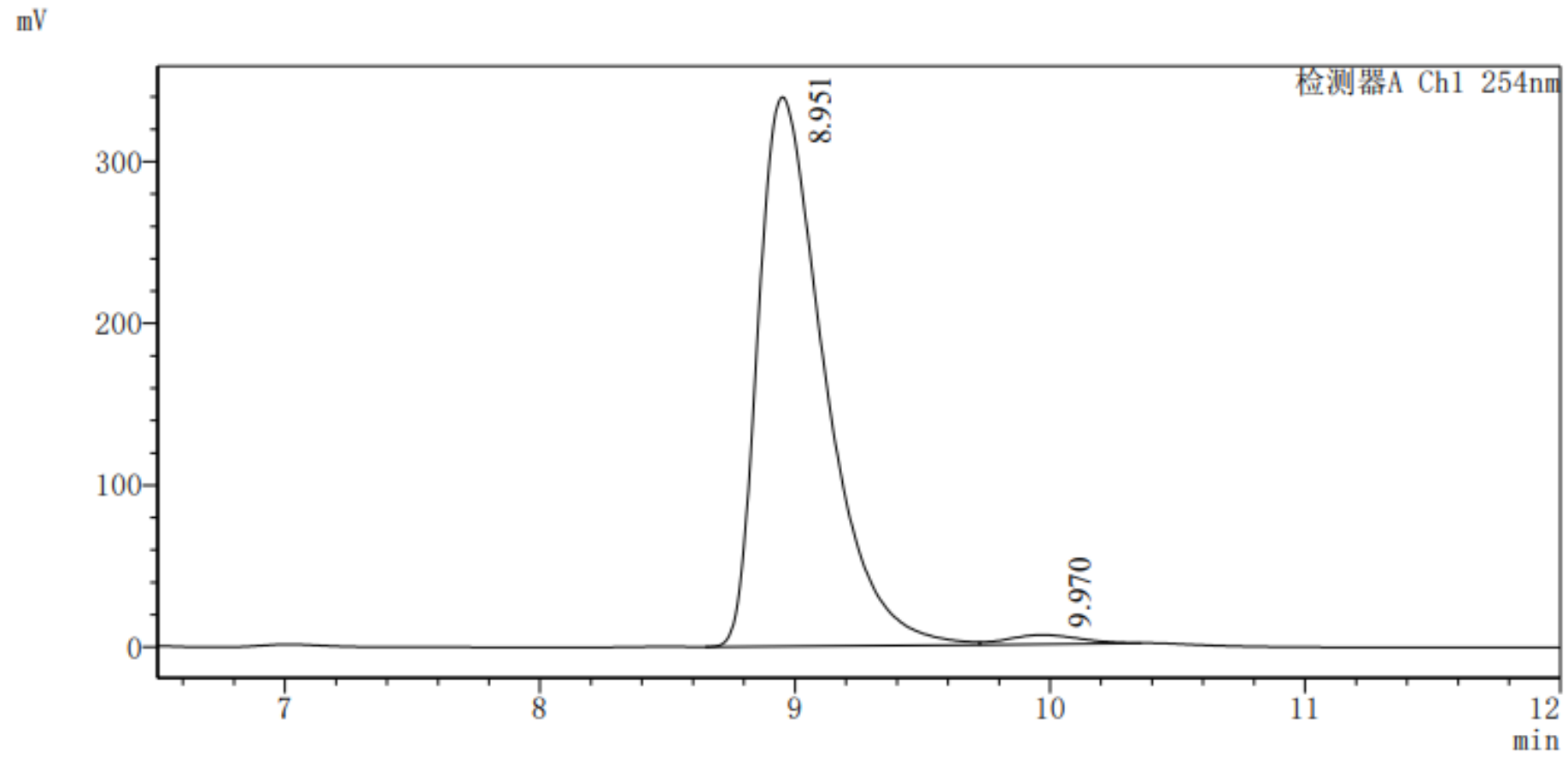

检测器A Ch1 254nm

No.	R.Time [min]	Height	Height%	Area	Area%
1	8.951	339526	98.339	6135171	98.181
2	9.970	5733	1.661	113667	1.819
总计		345260	100.000	6248838	100.000

When R = ONf, the yield is 95% and ee is 80%.

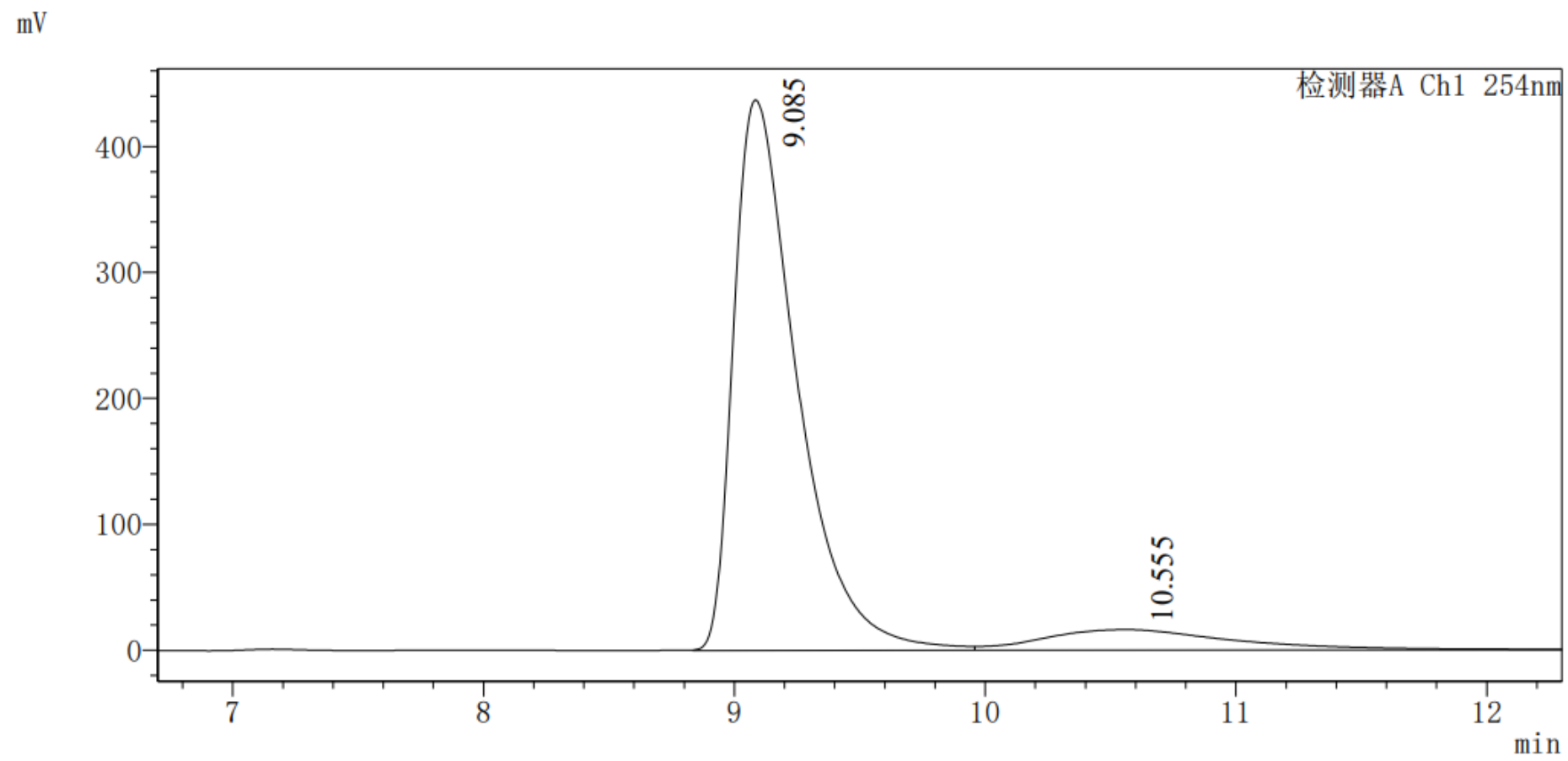

检测器A Ch1 254nm

No.	R.Time [min]	Height	Height%	Area	Area%
1	9.085	437329	96.398	7723319	89.943
2	10.555	16342	3.602	863561	10.057
总计		453671	100.000	8586880	100.000

4.2 Kinetic Experiments

Table S9. Kinetic Experiments Using (*rac*)-1a with 2a.[a]

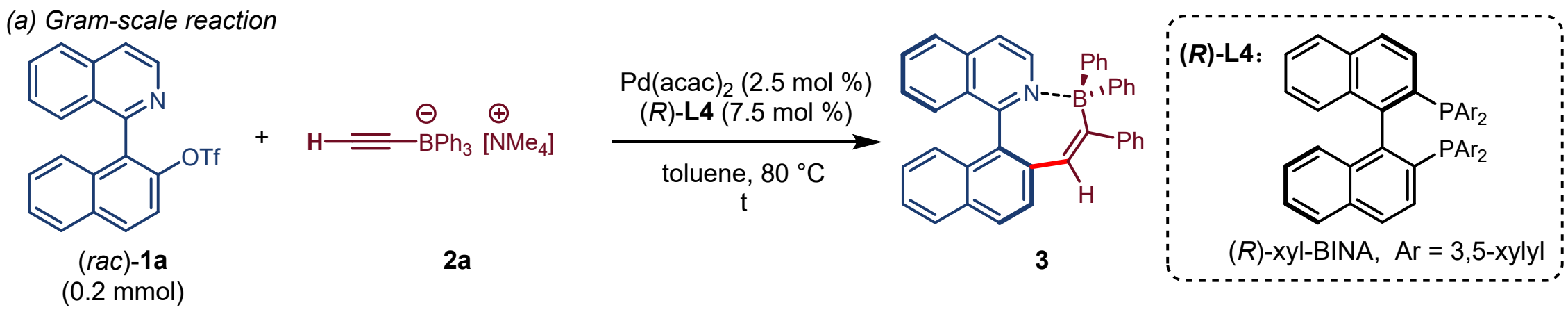

t (min)	Yield of recoved **1a** (%)[b]	*Ee* of recoved **1a** (%)[c]	Yield of **3a** (%)[b]	*Ee* of **3a** (%)[c]
0	100	0	0	-
5	59	21	30	98
10	50	27	46	99
15	35	33	59	99
20	20	43	75	99
40	0	-	98	99
80	0	-	98	99

[a] Reaction conditions: **1a** (0.2 mmol, 1.0 equiv.), **2a** (0.3 mmol, 1.5 equiv.), Pd(acac)$_2$ (2.5 mol%), and (*R*)-**L4** (7.5 mol%) in anhydrous toluene (2.0 mL) at 80°C under N_2. [b] The yield was determined by ^{1}H NMR analysis by using 1,3,5-trimethoxybenzene as an internal standard. [c] The *ee* values were determined by chiral HPLC analysis. *Ee* = Enantiomeric excess.

In glovebox, to an oven-dried 8.0 mL vial equipped with a magnetic stir bar was added Pd(acac)$_2$ (0.005 mmol, 2.5 mol%, 1.5 mg) and (*R*)-**L4** (0.0 mmol, 7.5 mol%, 11.0 mg) under N_2 atmosphere at room temperature. Anhydrous toluene (2.0 mL) was added and the mixture was stirred for 30 min at ambient temperature to afford the stock solution. 1-(Isoquinolin-1-yl)naphthalen-2-yl trifluoromethanesulfonate (0.2 mmol, 1.0 equiv., 80.6 mg) and tetramethylammonium ethynyltriphenylborate (0.3 mmol, 1.5 equiv., 102.4 mg) were added into another 10 mL Schlenk tube and the stock solution was transferred to this tube. Finally, the Schlenk tube was sealed with a septum, removed from the glovebox, and was heated to 80 °C bath in an oil bath for specified time showed in Table S9. After that, the reaction was cooled to room temperature and the crude reaction mixture was quenched by two drops of water and then filtered through a pad of silica gel. The filtrate was concentrated

under reduced pressure. The yield was determined by ^{1}H NMR analysis by using 1,3,5-trimethoxybenzene as an internal standard. The *ee* values were determined by chiral HPLC analysis.

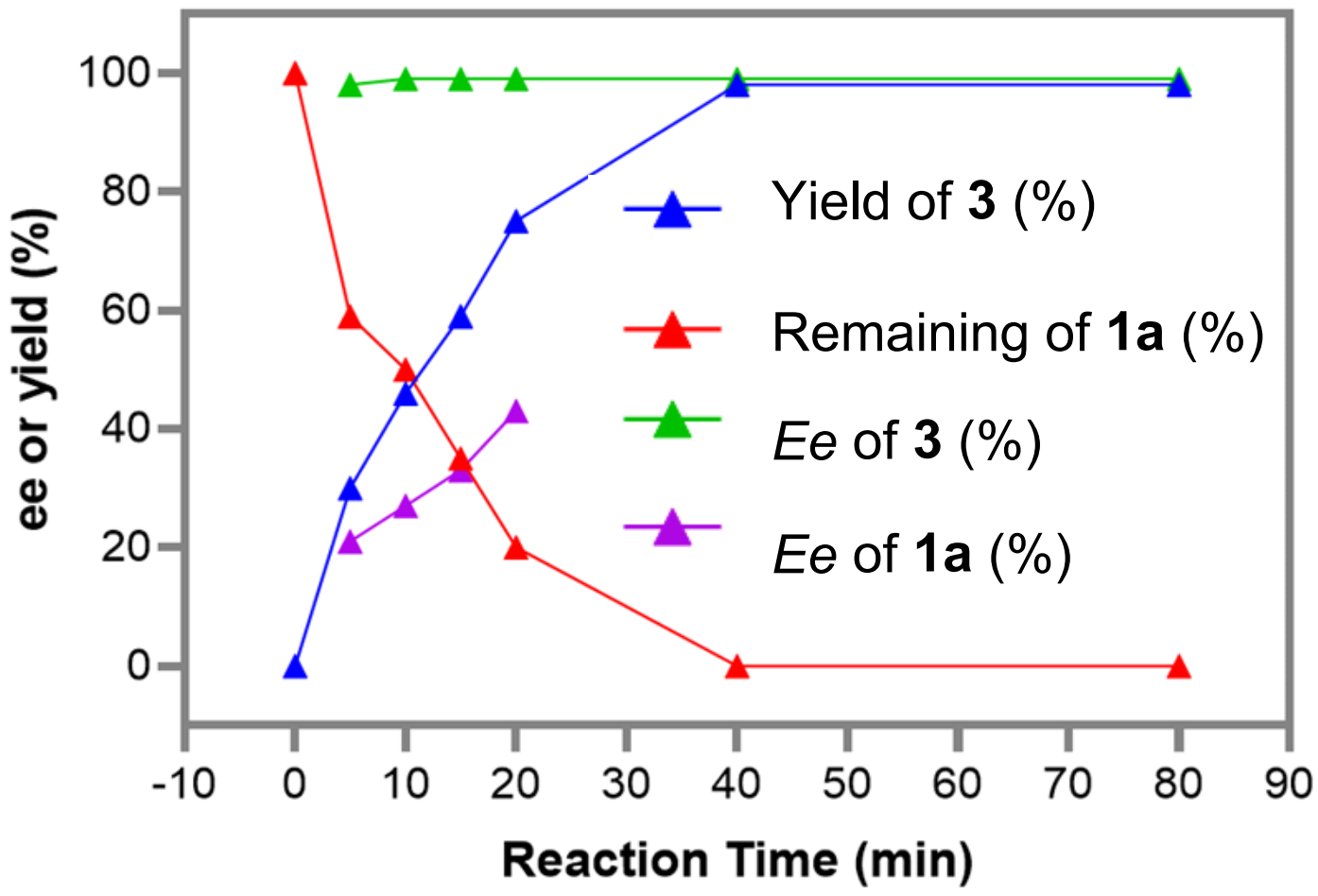

Figure S7. Kinetic Experiments

4.3 Temperature Studies for the Racemization

Table S10. Temperature Studies for the Racemization of 3.

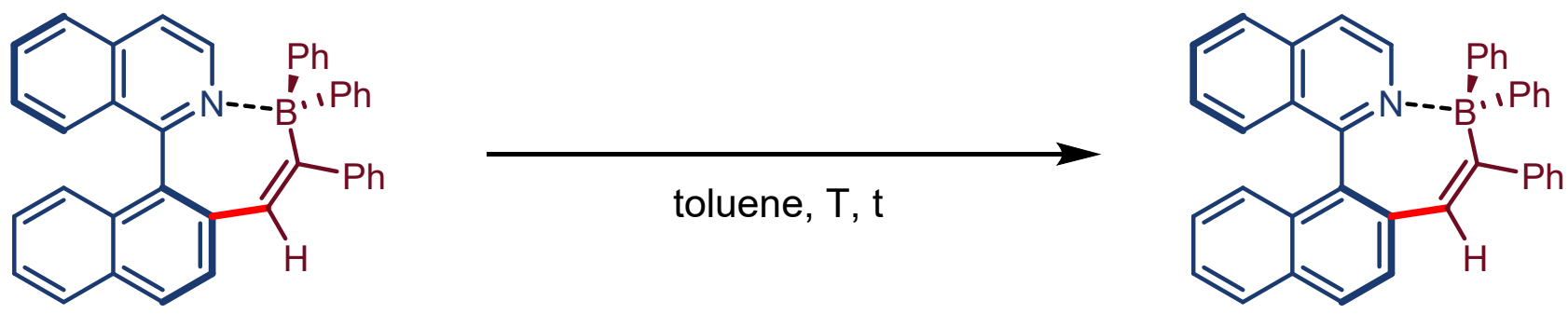

3, 99% *ee*

Entry	T, t	*Ee* of **3** after stirring	Entry	T, t	*Ee* of **3** after stirring
1	90 °C, 1 h	99% ee	4	140 °C, 1 h	99% ee
2	100 °C, 1 h	99% ee	5	140 °C, 16 h	99% ee
3	120 °C, 1 h	99% ee	6	160 °C, 16 h	97% ee

Thermal racemization of compound **3**: A solution of **3** (52.1 mg, 0.1 mmol, 99% ee) in toluene (1.0 mL) was heated at 80 °C, 100 °C, 120 °C, 140 °C, 160 °C. At intervals, small samples (0.5 ml) were taken and the solvent was removed by evaporation. The enantiomeric excess was determined by using HPLC (HPLC conditions: Chiral column IB, isopropanol/hexane = 99:1, flow: 1.0 mL/min, λ= 254 nm).

Racemic of **3**

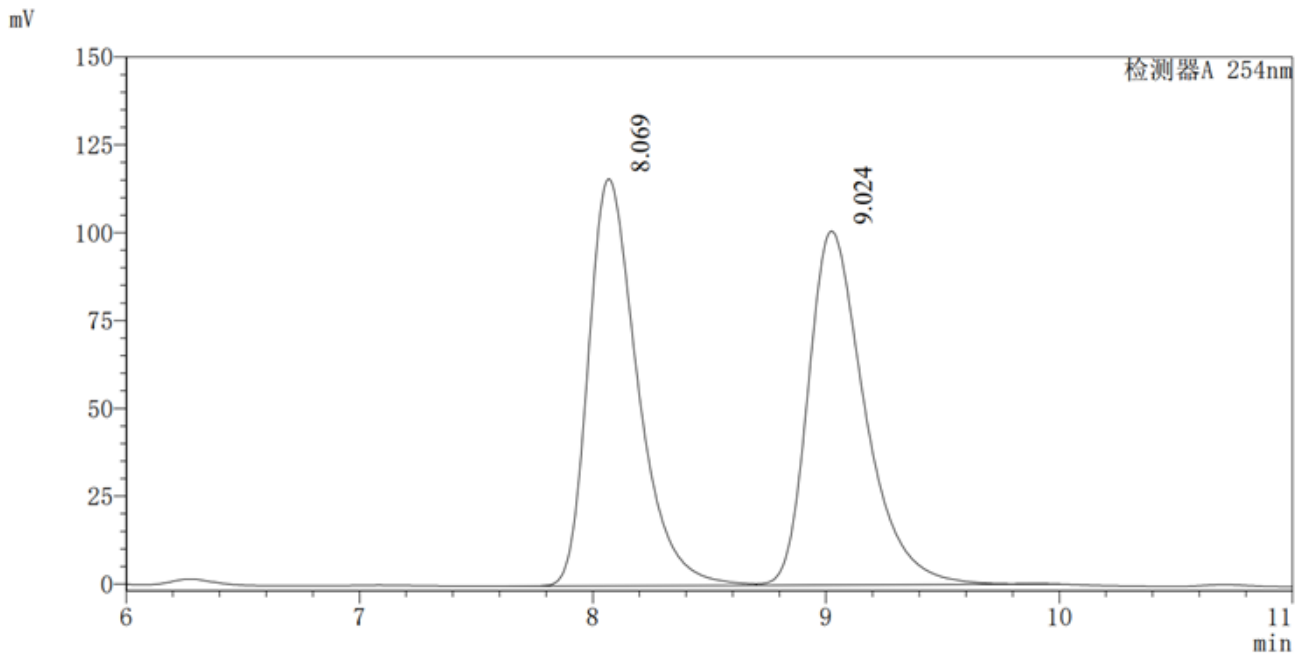

检测器A 254nm

No.	R.Time [min]	Height	Height%	Area	Area%
1	8.069	115883	53.481	1700480	50.025
2	9.024	100797	46.519	1698759	49.975
总计		216681	100.000	3399239	100.000

90 °C, 1 h

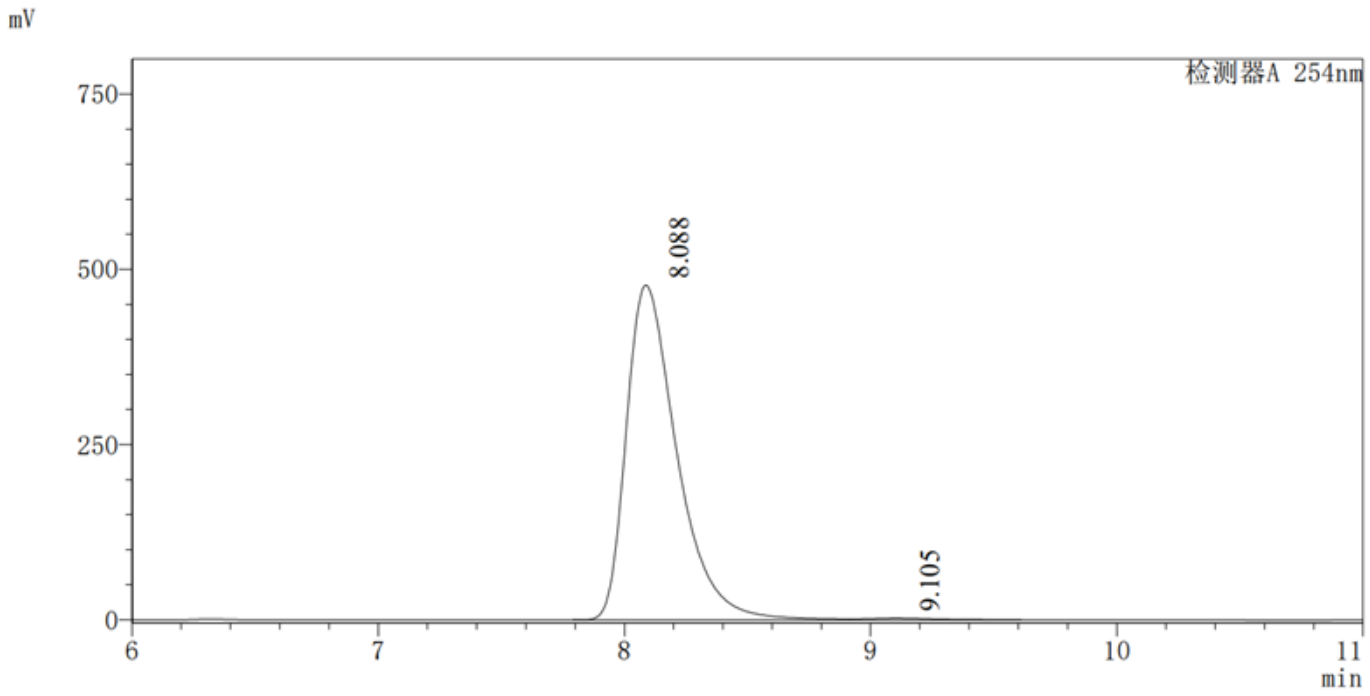

检测器A 254nm

No.	R.Time [min]	Height	Height%	Area	Area%
1	8.088	477219	99.503	6836883	99.349
2	9.105	2385	0.497	44767	0.651
总计		479604	100.000	6881650	100.000

100 °C, 1 h

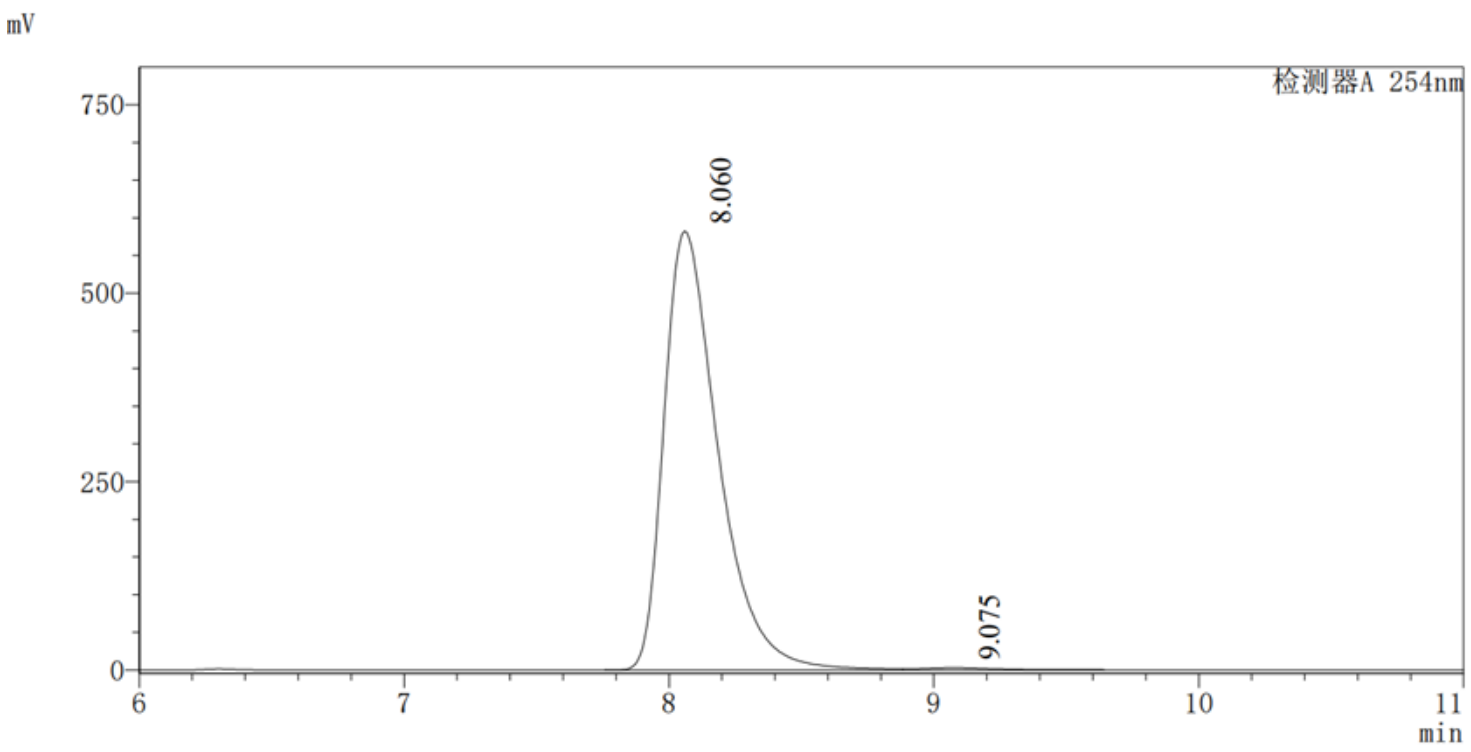

检测器A 254nm

No.	R.Time [min]	Height	Height%	Area	Area%
1	8.060	582225	99.518	8306829	99.358
2	9.075	2820	0.482	53642	0.642
总计		585045	100.000	8360471	100.000

120 °C, 1 h

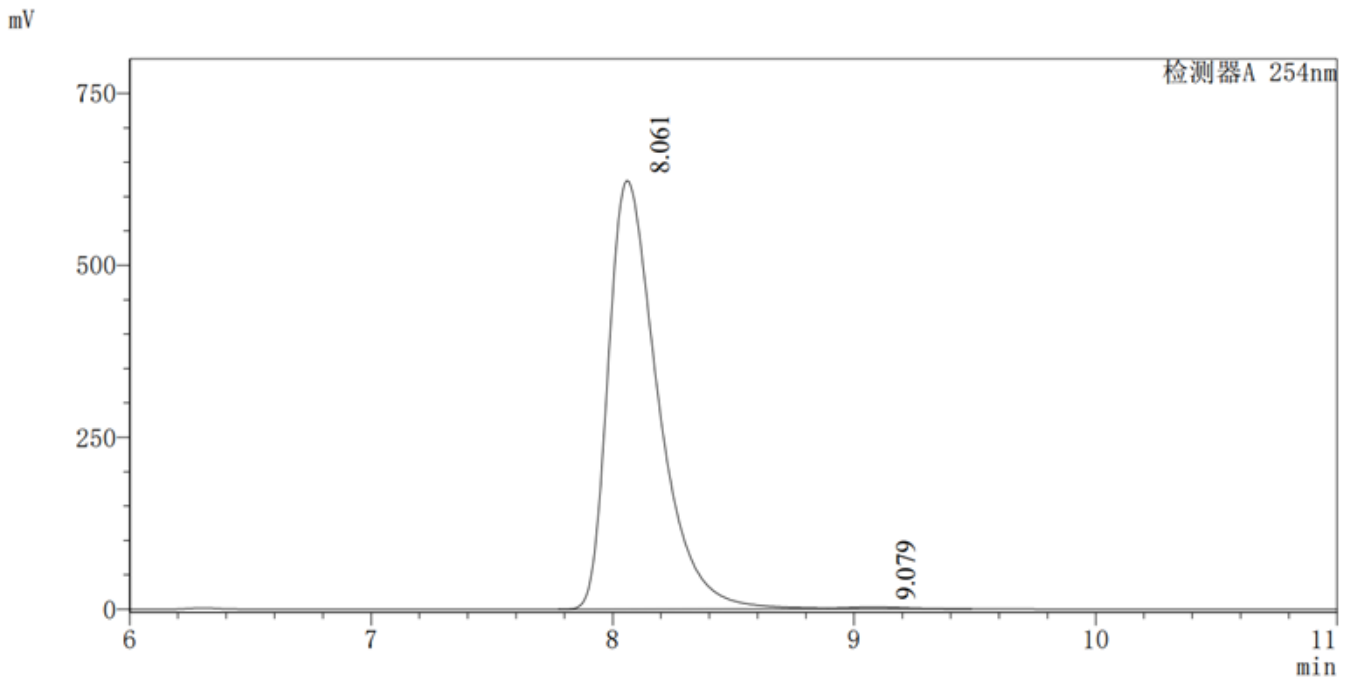

检测器A 254nm

No.	R.Time [min]	Height	Height%	Area	Area%
1	8.061	623341	99.549	8916529	99.435
2	9.079	2827	0.451	50707	0.565
总计		626168	100.000	8967236	100.000

140°C, 1 h

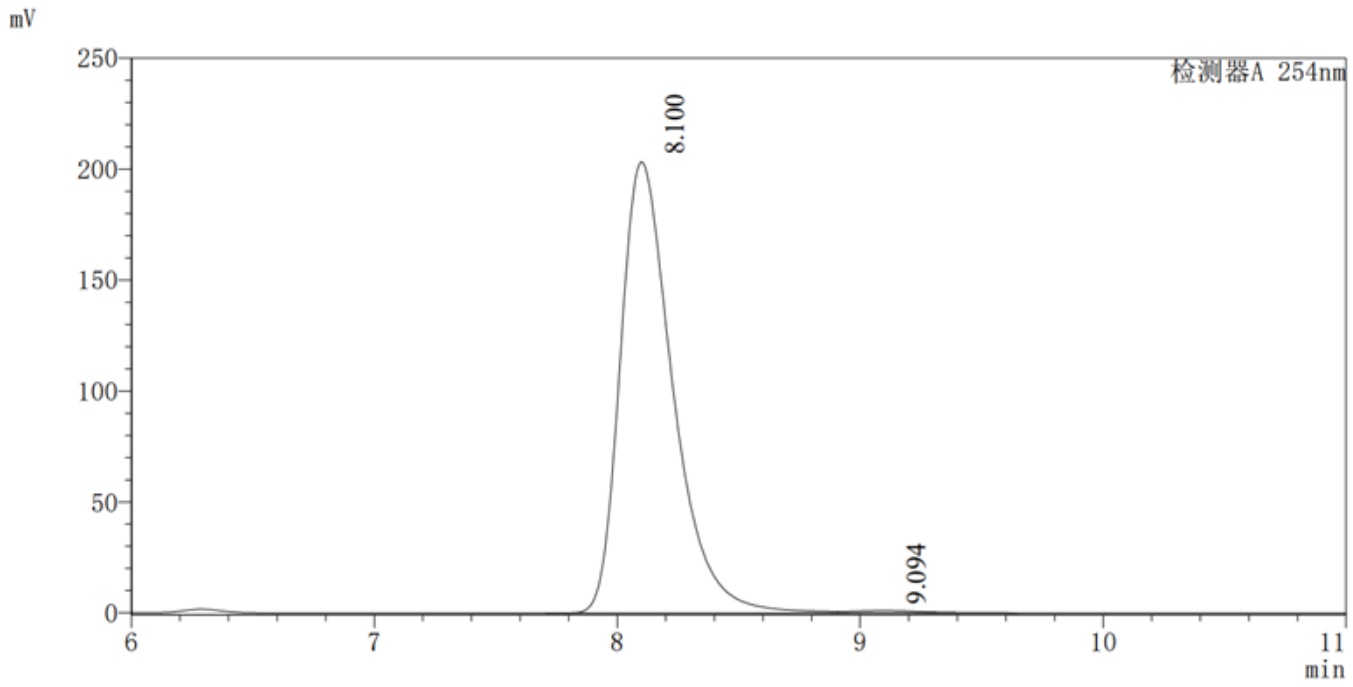

检测器A 254nm

No.	R.Time [min]	Height	Height%	Area	Area%
1	8.100	203480	99.486	3064246	99.315
2	9.094	1052	0.514	21138	0.685
总计		204532	100.000	3085384	100.000

140°C, 16 h

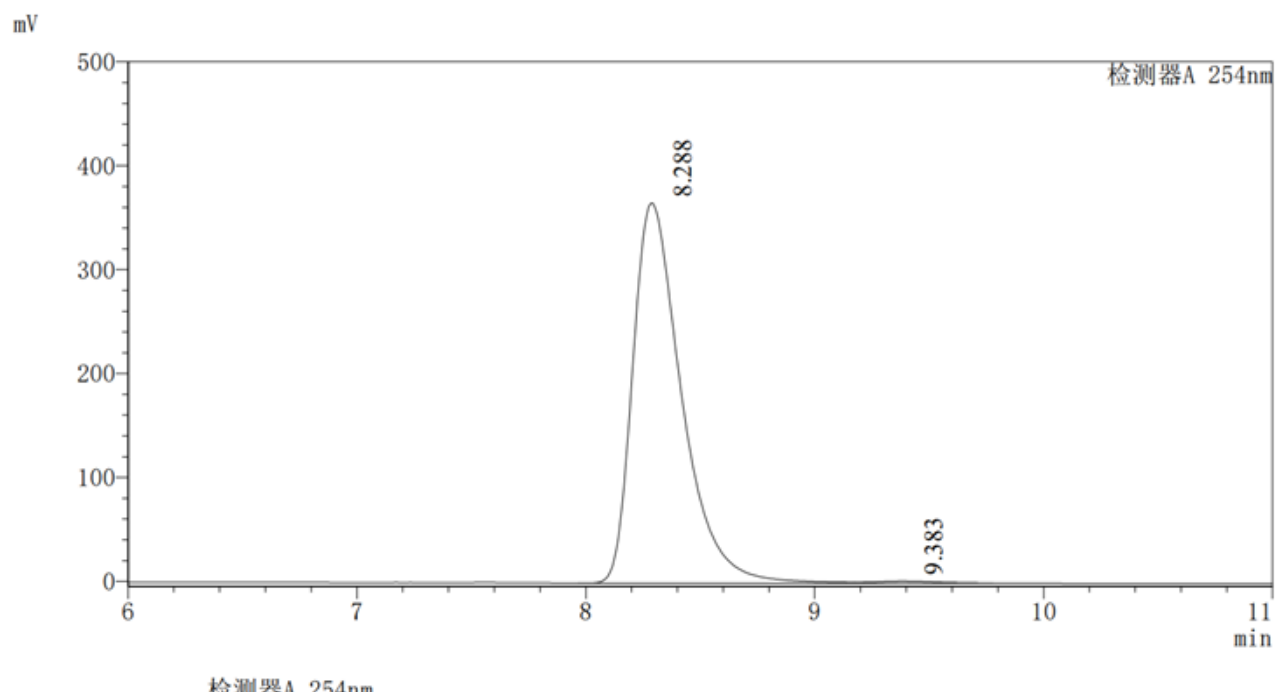

检测器A 254nm

No.	R.Time [min]	Height	Height%	Area	Area%
1	8.288	365516	99.513	5415753	99.387
2	9.383	1790	0.487	33392	0.613
总计		367306	100.000	5449146	100.000

160°C, 16 h

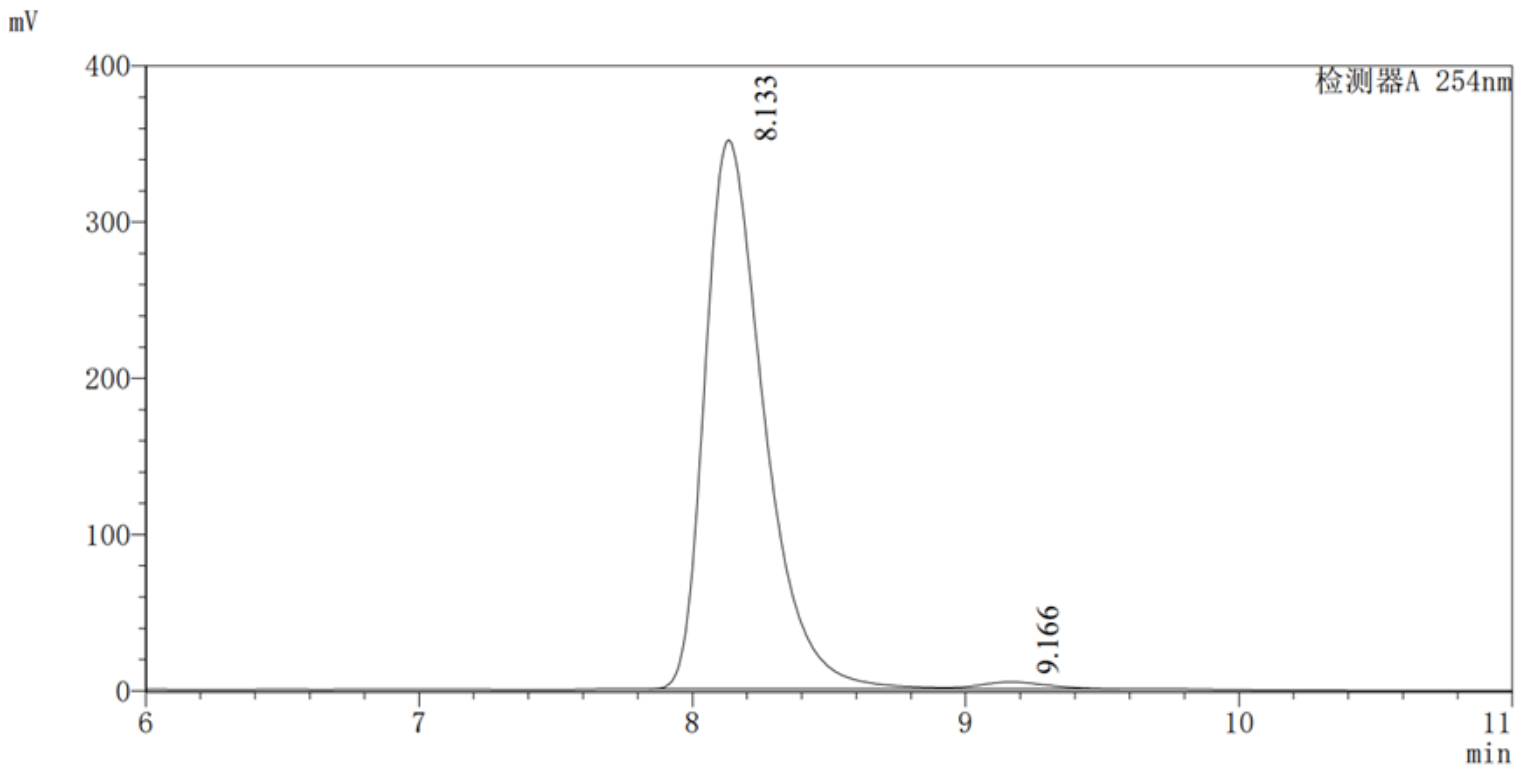

检测器A 254nm

No.	R.Time [min]	Height	Height%	Area	Area%
1	8.133	351477	98.754	5278199	98.559
2	9.166	4435	1.246	77172	1.441
总计		355912	100.000	5355371	100.000

5 SYNTHETIC APPLICATIONS

5.1 Gram-Scale Reaction

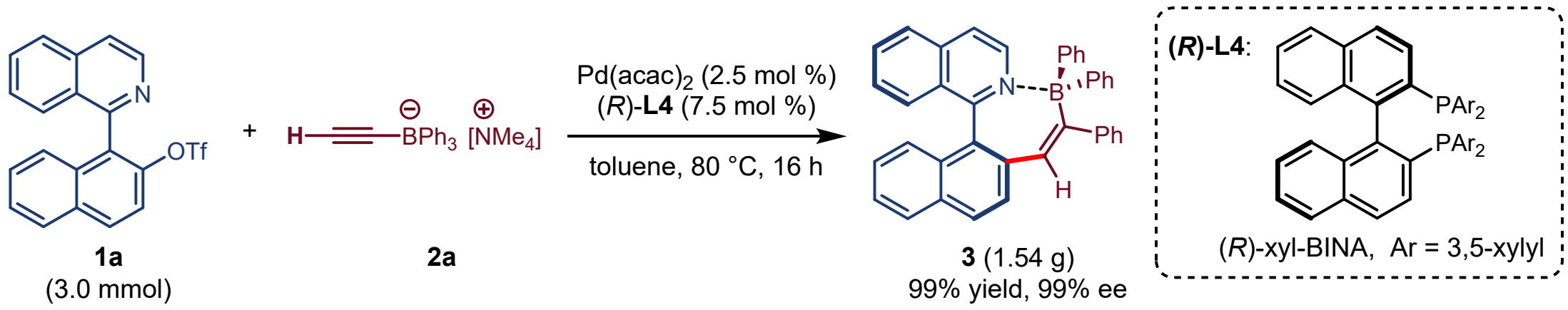

Prepared following **Procedure C**, using 1-(isoquinolin-1-yl)naphthalen-2-yl trifluoromethanesulfonate (1.21 g, 3.0 mmol, 1.0 equiv.), tetramethylammonium ethynyltriphenylborate (1.54 g, 4.5 mmol, 1.5 equiv.), Pd(acac)$_2$ (22.85 mg, 0.075 mmol, 2.5 mol%), and (*R*)-**L4** (165.35 mg, 0.225 mmol, 7.5 mol%). Purification by flash column chromatography (Petroleum ether/EtOAc: 100/1) to afford the title compound (1.54 g, 99%, 99% ee) as a yellow solid.

HPLC analysis: HPLC conditions: Chiral column IB, *n*-hexane/isopropanol: 99/1, flow rate = 1 mL/min, wavelength = 254 nm, t_R = 8.135 min for major isomer, t_R = 9.331 min for minor isomer, 99% ee.

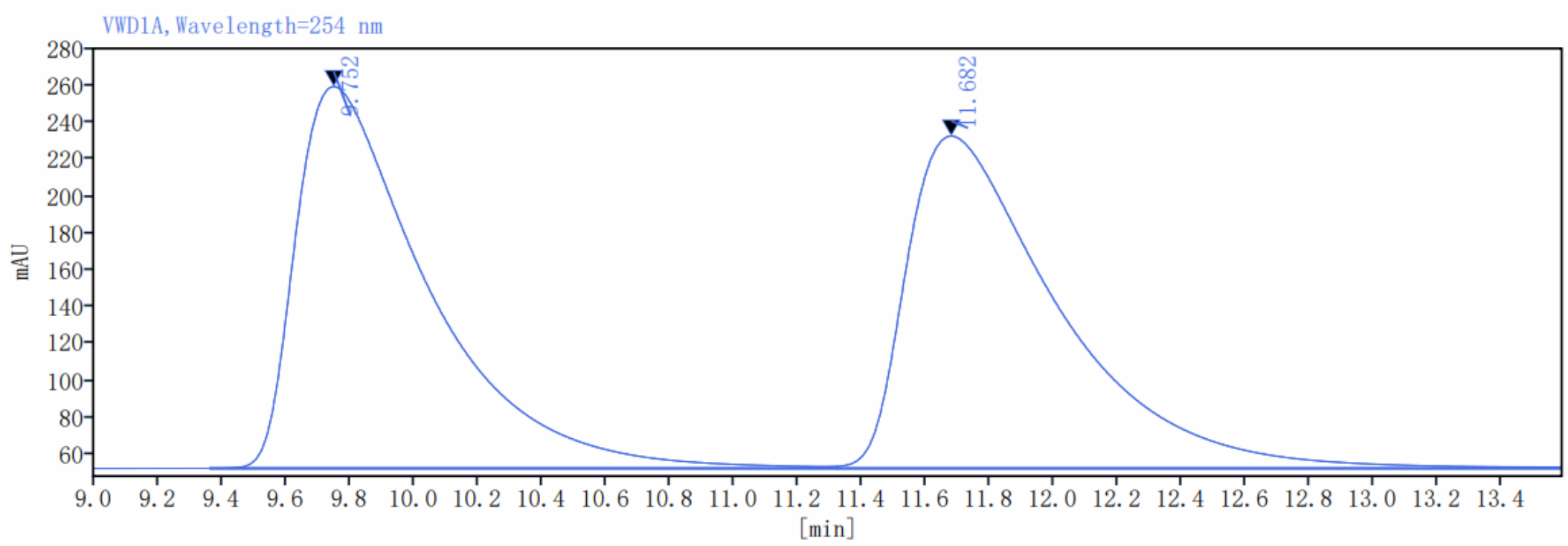

信号: VWD1A, Wavelength=254 nm

Retention Time [min]	Int Type	Width [min]	Area	Height	Area%
9.752	MM m	1.96	5954.05	207.01	49.87
11.682	MB m	2.92	5984.98	180.17	50.13

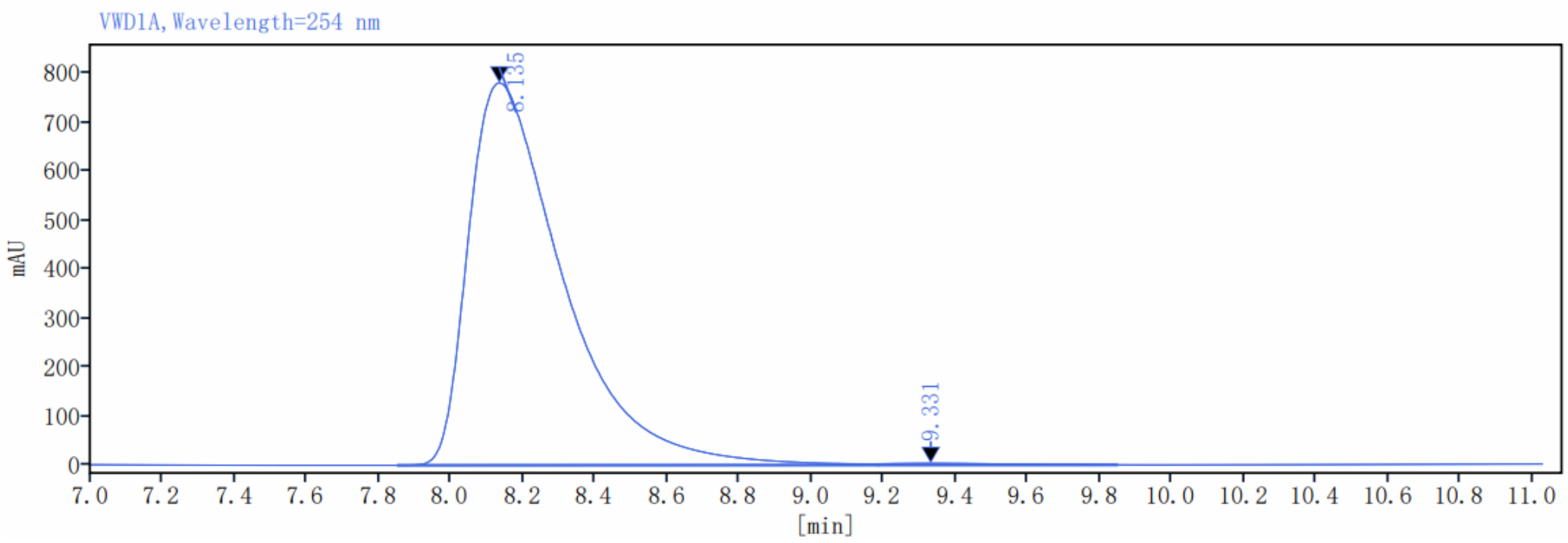

信号: VWD1A, Wavelength=254 nm

Retention Time [min]	Int Type	Width [min]	Area	Height	Area%
8.135	BM m	1.33	14290.73	781.05	99.47
9.331	MM m	0.66	76.56	3.51	0.53

5.2 Transformations of Chiral Axial Seven-Membered Borates

5.2.1 Suzuki-Miyaura Cross-Coupling of Compound 3

General Procedure F:

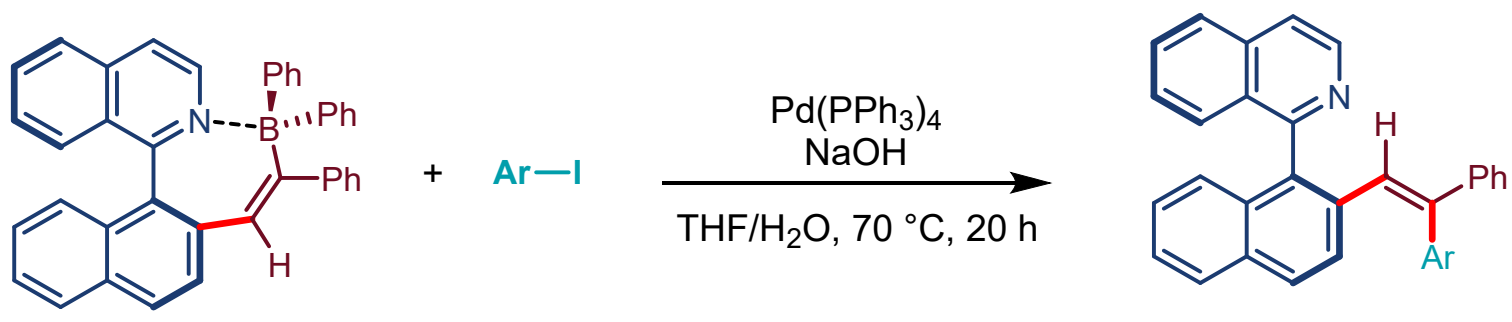

A dried Schlenk tube was charged with $Pd(PPh_3)_4$ (0.01 mmol, 10 mol%, 11.6 mg) and ArI (0.4 mmol, 4.0 equiv.). The anhydrous THF (1.5 mL) was added under an N_2 and the mixture was stirred for 30 min at rt. Then compound **3** (0.1 mmol, 1.0 equiv., 52.1 mg) and NaOH (0.4 mmol, 4.0 equiv., 16.0 mg, dissolved in 0.5 mL H_2O) were sequentially added under argon atmosphere. The tube was heated to 70 ℃ in an oil bath and stirred at the same temperature for 20 hours. The reaction mixture was then cooled to rt, quenched with H_2O (10 mL), and extracted with CH_2Cl_2 (3 × 20 mL). The organic layer was combined and dried over anhydrous $MgSO_4$, filtered, concentrated. The crude material was purified by flash column chromatography (*n*-hexane/EtOAc) to afford the desired product **52–56**.

(*R*,*Z*)-1-{2-[2-Phenyl-2-(*p*-tolyl)vinyl]naphthalen-1-yl}isoquinoline (52)

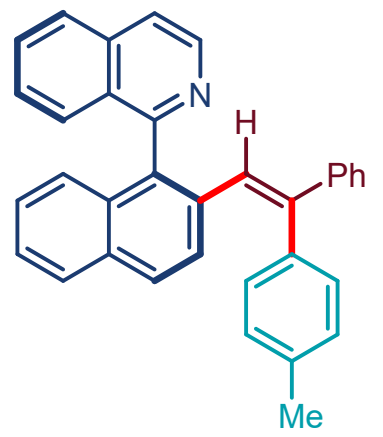

52

Prepared following **Procedure F**, using compound **3** (0.1 mmol, 1.0 equiv., 52.1 mg), 1-iodo-4-methylbenzene (87.2 mg, 0.4 mmol, 4.0 equiv.), $Pd(PPh_3)_4$ (0.01 mmol, 10 mol%, 11.6 mg), NaOH (0.4 mmol, 4.0 equiv., 16.0 mg, dissolved in 0.5 mL H_2O), and anhydrous THF (1.5 mL). Purification by flash column chromatography (Petroleum ether/EtOAc: 10/1) to afford the title compound (44.3 mg, 99%) as a white solid.

TLC: R_f = 0.5 (Petroleum ether/EtOAc: 10/1, $KMnO_4$ stain).

M. p.: 175 – 176 °C.

$[\alpha]^{18}_D$: +292.51 (c 0.20, CH_2Cl_2).

NMR Spectroscopy (*see spectra*):

^{1}H NMR (400 MHz, $CDCl_3$) δ_H = 8.78 (d, J = 5.8 Hz, 1H), 7.94 (d, J = 8.3 Hz, 1H), 7.82 (d, J = 8.1 Hz, 1H), 7.76 (d, J = 5.8 Hz, 1H), 7.70 (t, J = 7.6 Hz, 1H), 7.65 (d, J = 8.8 Hz, 1H), 7.58 (d, J = 8.4 Hz, 1H), 7.50-7.36 (m, 2H), 7.29 – 7.07 (m, 11H), 7.00 (d, J = 8.5 Hz, 1H), 6.64 (s, 1H), 2.40 (s, 3H) ppm;

^{13}C NMR (100 MHz, $CDCl_3$) δ_C = 160.2, 143.9, 143.5, 142.9, 137.4, 137.3, 136.4, 136.4, 135.2, 133.0, 132.4, 130.9, 130.4, 129.1, 128.7, 128.2, 128.0, 127.9, 127.9, 127.6, 127.5, 127.4, 127.4, 127.2, 126.3, 126.3, 126.0, 125.8, 120.4, 21.5 ppm.

IR (film): ν_{max} 2957, 2923, 1737, 1509, 1492, 1463, 1313, 1016, 961, 818, 744, 694 cm^{-1}.

HRMS (ESI): m/z calculated for $C_{34}H_{26}N^+$ $[M+H]^+$ 448.2060, found 448.2052.

HPLC analysis: HPLC conditions: Chiral column IB, *n*-hexane/isopropanol: 95/5, flow rate = 1 mL/min, wavelength = 254 nm, t_R = 6.779 min for major isomer, t_R = 7.871 min for minor isomer, 93% ee.

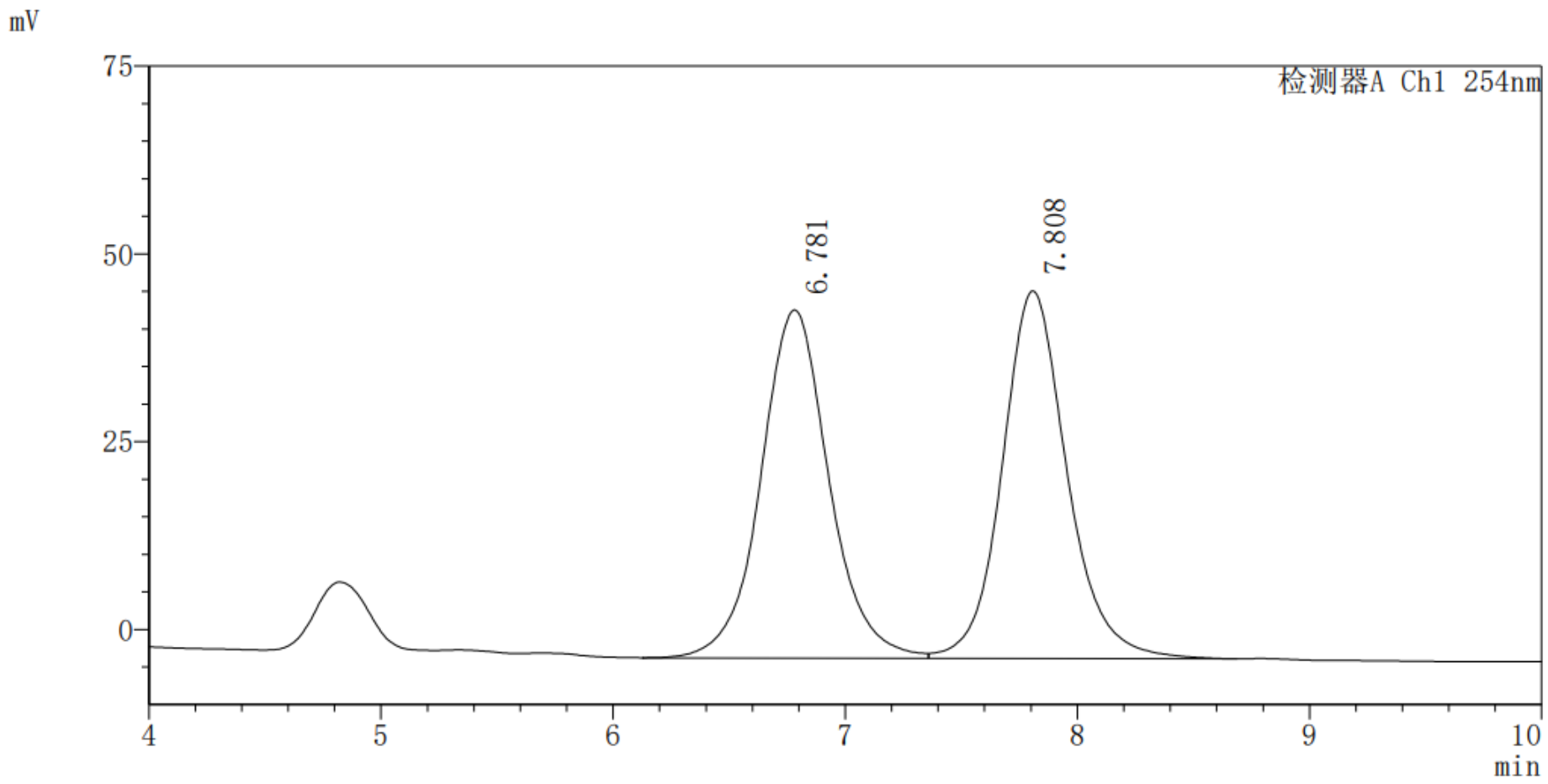

检测器A Ch1 254nm

No.	R.Time	Height	Height%	Area	Area%
1	6.781	46368	48.640	945023	49.859
2	7.808	48961	51.360	950360	50.141

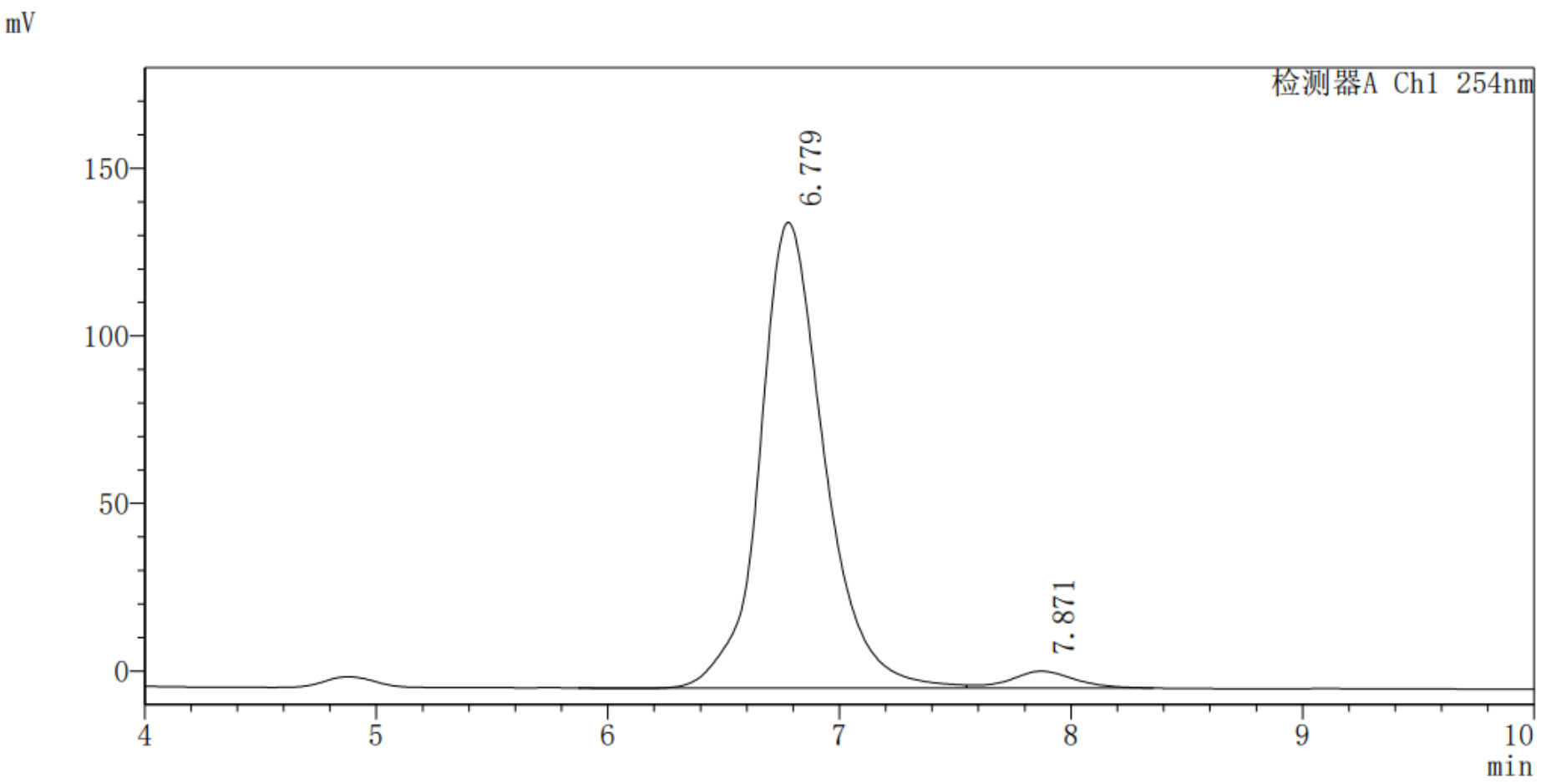

检测器A Ch1 254nm

No.	R.Time	Height	Height%	Area	Area%
1	6.779	138923	96.515	2688974	96.525
2	7.871	5016	3.485	96800	3.475

(*R*,*Z*)-4-{2-[1-(Isoquinolin-1-yl)naphthalen-2-yl]-1-phenylvinyl}benzonitrile (53)

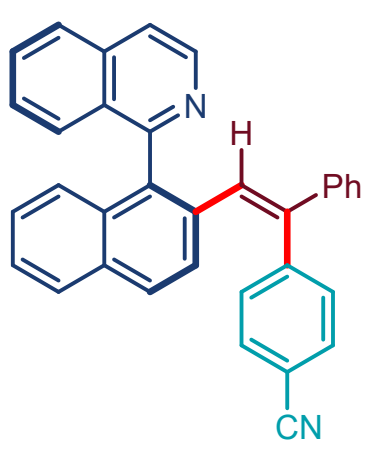

53

Prepared following **Procedure F**, using compound **3** (0.1 mmol, 1.0 equiv., 52.1 mg), 4-iodobenzonitrile (91.6 mg, 0.4 mmol, 4.0 equiv.), $Pd(PPh_3)_4$ (0.01 mmol, 10 mol%, 11.6 mg), NaOH (0.4 mmol, 4.0 equiv.,

16.0 mg, dissolved in 0.5 mL H_2O), anhydrous THF (1.5 mL). Purification by flash column chromatography (Petroleum ether/EtOAc: 5/1) to afford the title compound (40.8 mg, 89%) as a white solid.

TLC: R_f = 0.4 (Petroleum ether/EtOAc: 5/1, $KMnO_4$ stain).

M. p.: 89 – 90 °C.

$[\alpha]^{18}_D$: +141.62 (c 0.20, CH_2Cl_2).

NMR Spectroscopy (*see spectra*):

^{1}H NMR (400 MHz, $CDCl_3$) δ_H = 8.72 (d, J = 5.6 Hz, 1H), 7.92 (d, J = 8.3 Hz, 1H), 7.82 (d, J = 8.3 Hz, 1H), 7.75 – 7.63 (m, 3H), 7.51 (d, J = 8.1 Hz, 2H), 7.47 – 7.39 (m, 3H), 7.30 – 7.17 (m, 6H), 7.06 (d, J = 8.6 Hz, 1H), 7.00 (d, J = 8.6 Hz, 3H), 6.73 (s, 1H) ppm;

^{13}C NMR (100 MHz, $CDCl_3$) δ_C = 159.6, 145.5, 143.0, 142.3, 142.1, 136.8, 136.4, 134.2, 132.9, 132.6, 132.1, 131.8, 130.4, 128.9, 128.4, 128.3, 128.2, 128.1, 128.0, 128.0, 127.7, 127.6, 127.3, 127.1, 126.7, 126.3, 126.1, 120.5, 119.0, 111.2 ppm.

IR (film): ν_{max} 3451, 2956, 2924, 2850, 2226, 1645, 821, 748, 695 cm^{-1}.

HRMS (ESI): m/z calculated for $C_{34}H_{23}N_2^+$ $[M+H]^+$ 459.1856, found 459.1851.

HPLC analysis: HPLC conditions: Chiral column IB, *n*-hexane/isopropanol: 97/3, flow rate = 1 mL/min, wavelength = 254 nm, t_R = 16.152 min for major isomer, t_R = 18.438 min for minor isomer, 97% ee.

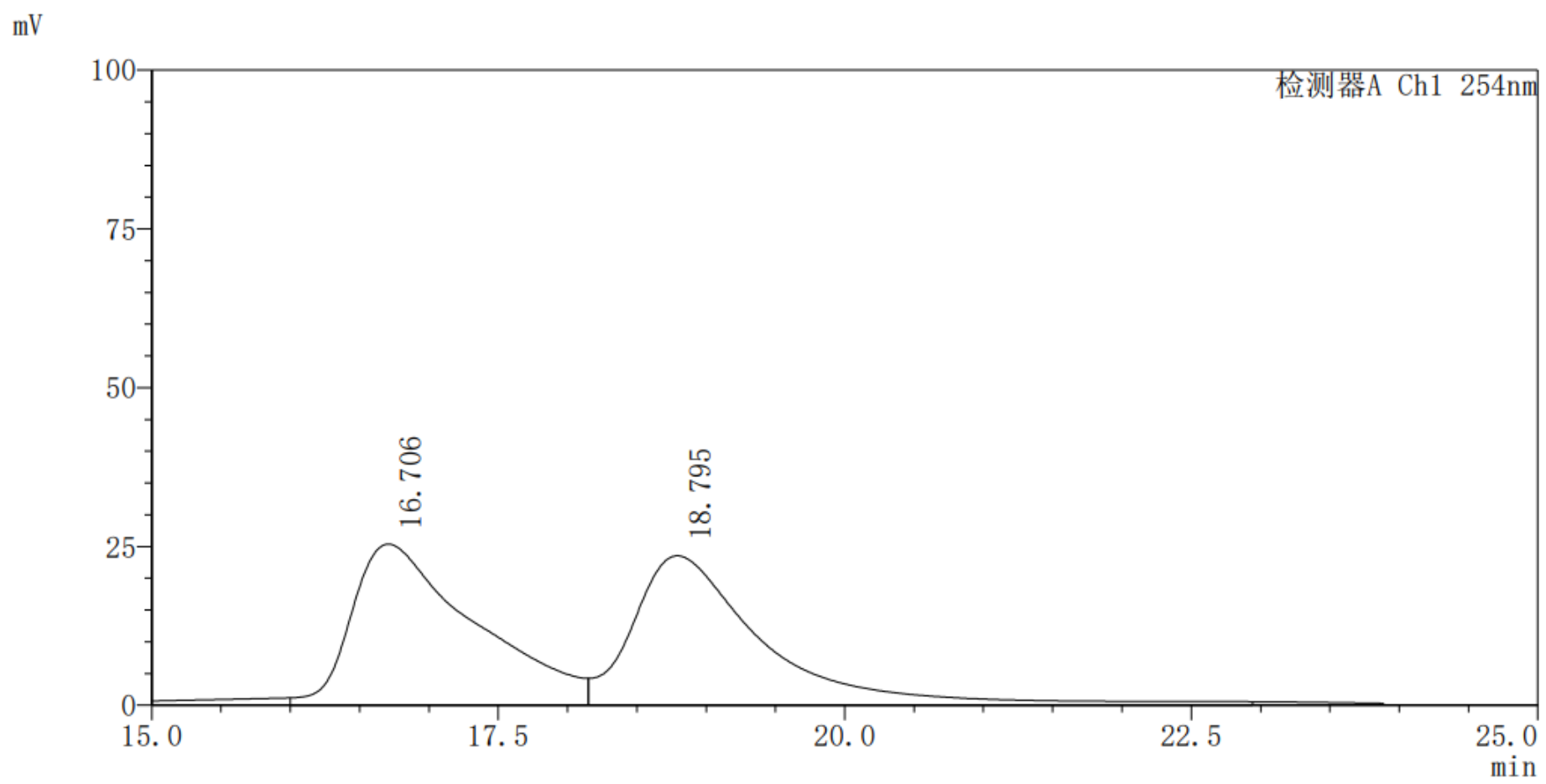

检测器A Ch1 254nm

No.	R.Time	Height	Height%	Area	Area%
1	16.706	25368	51.880	1565739	50.225
2	18.795	23530	48.120	1551717	49.775

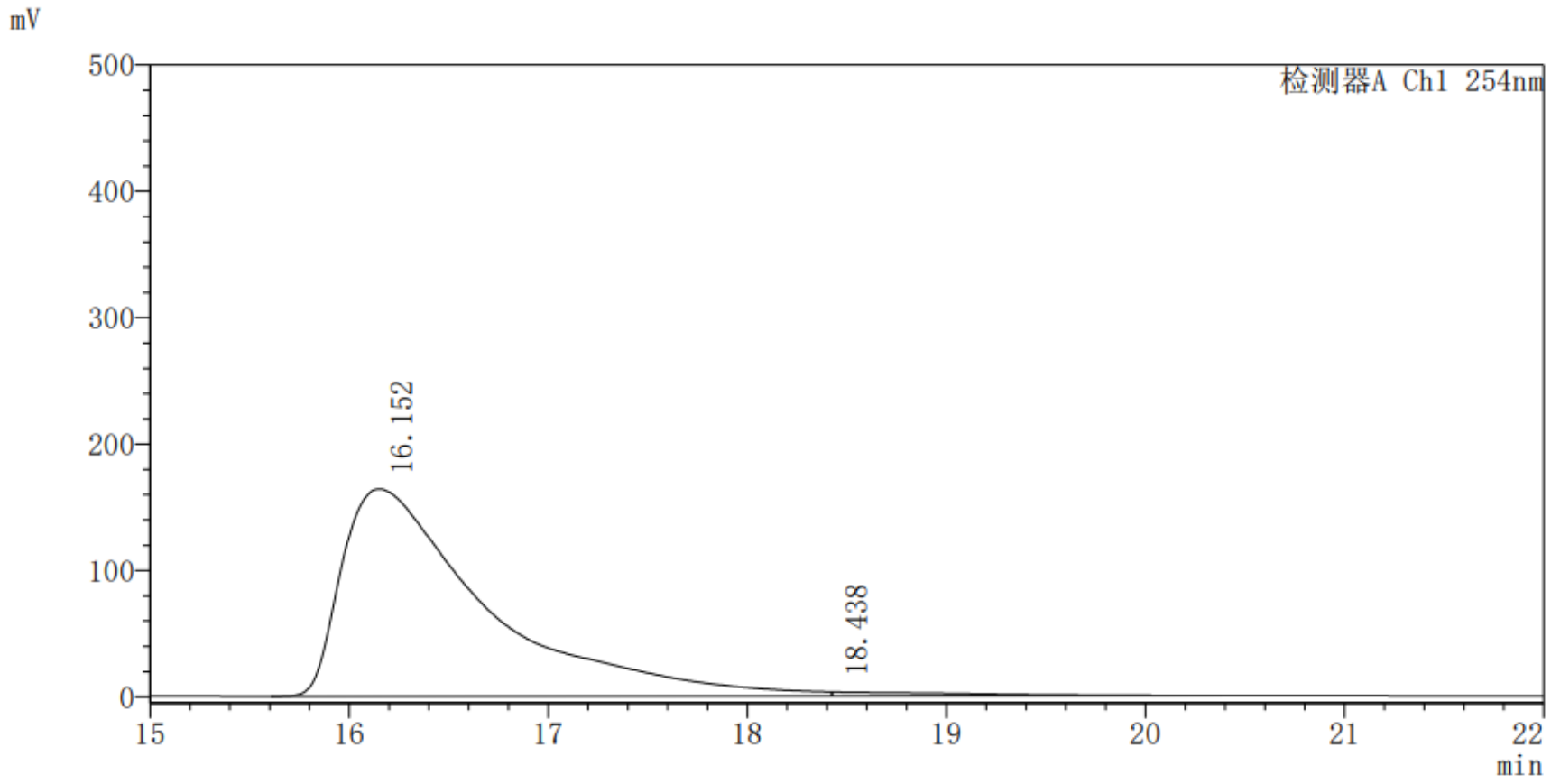

检测器A Ch1 254nm

No.	R. Time	Height	Height%	Area	Area%
1	16. 152	163951	98. 118	8250854	98. 274
2	18. 438	3145	1. 882	144922	1. 726
总计		167096	100. 000	8395776	100. 000

(*R*,*Z*)-1-{4-{2-[1-(Isoquinolin-1-yl)naphthalen-2-yl]-1-phenylvinyl}phenyl}ethan-1-one (54)

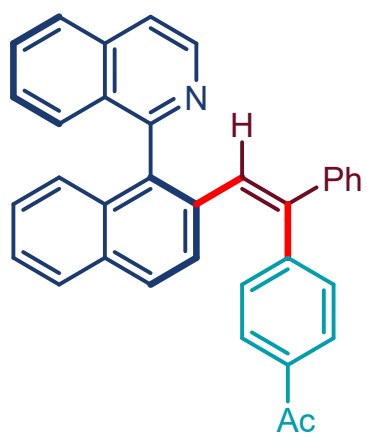

54

Prepared following **Procedure F**, using compound **3** (0.1 mmol, 1.0 equiv., 52.1 mg), 1-(4-iodophenyl)ethan-1-one (98.4 mg, 0.4 mmol, 4.0 equiv.), $Pd(PPh_3)_4$ (0.01 mmol, 10 mol%, 11.6 mg), NaOH (0.4 mmol, 4.0 equiv., 16.0 mg, dissolved in 0.5 mL H_2O), anhydrous THF (1.5 mL). Purification by flash column chromatography (Petroleum ether/EtOAc: 5/1) to afford the title compound (47.1 mg, 99%) as a white solid.

TLC: R_f = 0.5 (Petroleum ether/EtOAc: 5/1, $KMnO_4$ stain).

M. p.: 198 – 199 °C.

$[\alpha]^{18}_D$: +138.02 (c 0.20, CH_2Cl_2).

NMR Spectroscopy (*see spectra*):

^{1}H NMR (400 MHz, $CDCl_3$) δ_H = 8.74 (d, *J* = 5.8 Hz, 1H), 7.92 (d, *J* = 8.3 Hz, 1H), 7.85 (d, *J* = 8.3 Hz, 2H), 7.79 (d, *J* = 7.9 Hz, 1H), 7.73 (d, *J* = 5.6 Hz, 1H), 7.68 (t, *J* = 6.8 Hz, 1H), 7.62 (d, *J* = 8.8 Hz, 1H), 7.50 (d, *J* = 8.1 Hz, 1H), 7.45 – 7.38 (m, 2H), 7.30 (d, *J* = 8.3 Hz, 2H), 7.22 (t, *J* =

7.0 Hz, 1H), 7.19 – 7.13 (m, 3H), 7.10 (d, *J* = 8.7 Hz, 1H), 7.06 – 6.96 (m, 3H), 6.72 (s, 1H), 2.60 (s, 3H) ppm;

^{13}C NMR (100 MHz, $CDCl_3$) δ_C = 197.9, 159.8, 145.7, 142.9, 142.9, 142.5, 136.7, 136.4, 136.1, 134.5, 132.9, 132.5, 131.3, 130.4, 128.6, 128.4, 128.2, 128.1, 128.1, 128.0, 127.9, 127.8, 127.7, 127.6, 127.3, 127.2, 126.5, 126.1, 126.1, 120.5, 26.7 ppm.

IR (film): ν_{max} 2956, 2921, 2851, 1681, 1462, 1401, 1359, 1259, 816, 747, 695 cm^{-1}.

HRMS (ESI): m/z calculated for $C_{35}H_{26}NO^+$ $[M+H]^+$ 476.2009, found 476.2015.

HPLC analysis: HPLC conditions: Chiral column IB, *n*-hexane/isopropanol: 97/3, flow rate = 1 mL/min, wavelength = 254 nm, t_R = 19.410 min for major isomer, t_R = 16.965 min for minor isomer, 96% ee.

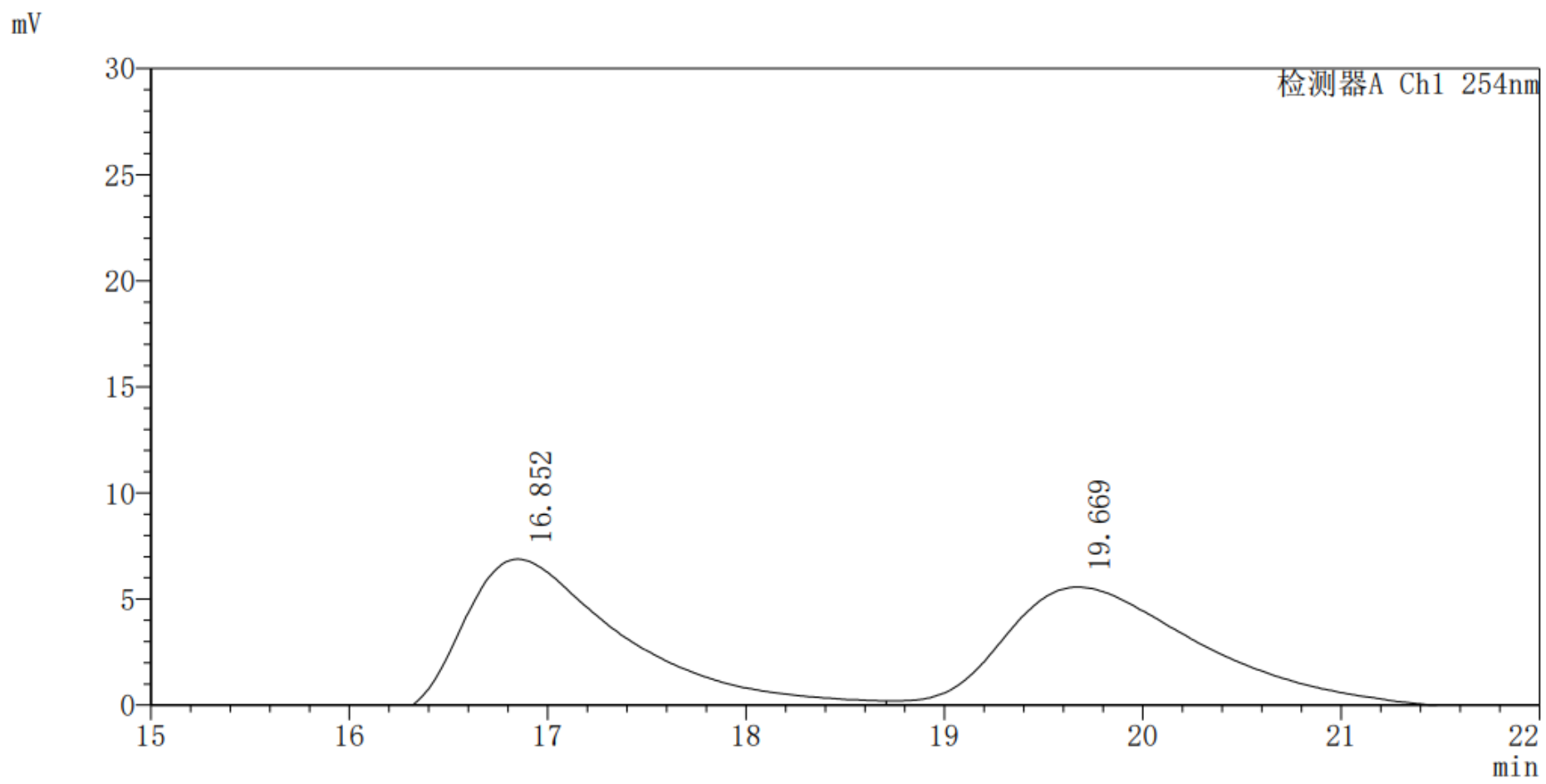

检测器A Ch1 254nm

No.	R.Time	Height	Height%	Area	Area%
1	16.852	7597	55.168	479252	49.714
2	19.669	6174	44.832	484769	50.286

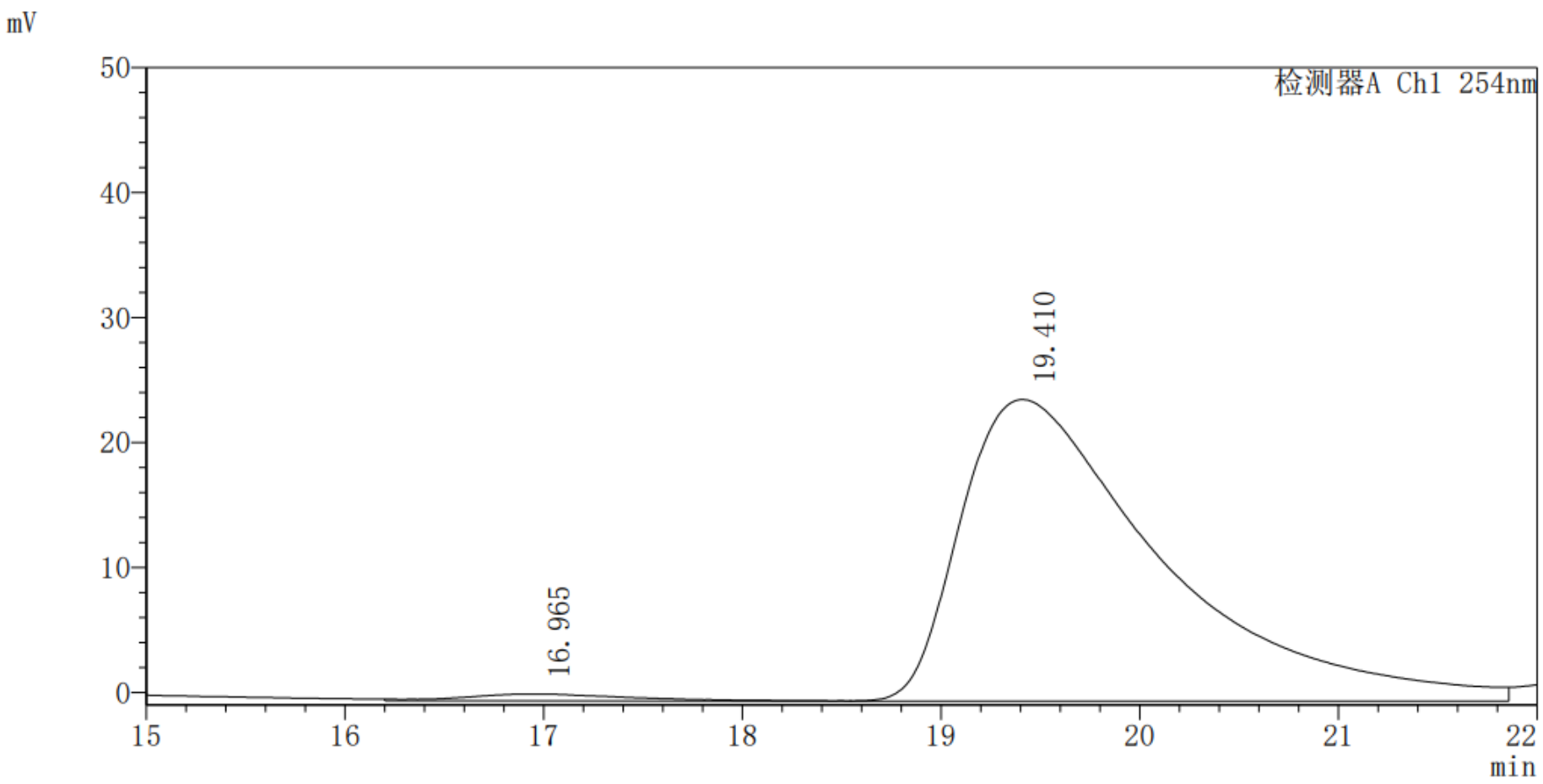

检测器A Ch1 254nm

No.	R.Time	Height	Height%	Area	Area%
1	16.965	549	2.226	31583	1.830
2	19.410	24123	97.774	1694034	98.170

(*R*,*Z*)-1-{2-[2-(6-Methylpyridin-3-yl)-2-phenylvinyl]naphthalen-1-yl}isoquinoline (55)

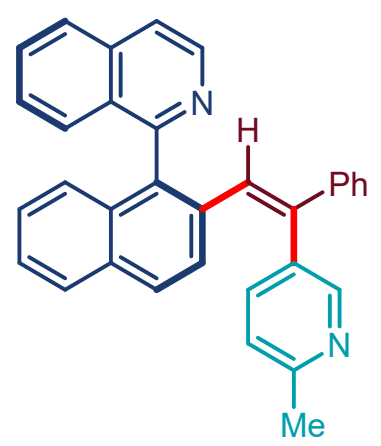

55

Prepared following **Procedure F**, using compound **3** (0.1 mmol, 1.0 equiv., 52.1 mg), 5-bromo-2-methylpyridine (68.8 mg, 0.4 mmol, 4.0 equiv.), $Pd(PPh_3)_4$ (0.01 mmol, 10 mol%, 11.6 mg), NaOH (0.4 mmol, 4.0 equiv., 16.0 mg, dissolved in 0.5 mL H_2O), anhydrous THF (1.5 mL). Purification by flash column chromatography (Petroleum ether/EtOAc: 5/1) to afford the title compound (44.4 mg, 99%) as a white solid.

TLC: R_f = 0.4 (Petroleum ether/EtOAc: 5/1, $KMnO_4$ stain).

M. p.: 112 – 113 °C.

$[\alpha]^{18}_D$: +110.55 (c 0.20, CH_2Cl_2).

NMR Spectroscopy (*see spectra*):

^{1}**H NMR** (400 MHz, $CDCl_3$) δ_H = 8.72 (d, *J* = 5.8 Hz, 1H), 8.27 (d, *J* = 2.4 Hz, 1H), 7.89 (d, *J* = 8.3 Hz, 1H), 7.80 (d, *J* = 8.3 Hz, 1H), 7.73 – 7.63 (m, 3H), 7.50 – 7.36 (m, 4H), 7.23 – 7.13 (m,

5H), 7.06 – 6.95 (m, 4H), 6.76 (s, 1H), 2.56 (s, 3H) ppm;

^{13}C NMR (100 MHz, $CDCl_3$) δ_C = 159.8, 157.3, 150.7, 142.8, 142.5, 140.4, 138.8, 136.6, 136.4, 134.4, 133.2, 132.9, 132.5, 130.4, 128.6, 128.2, 128.2, 128.1, 128.0, 128.0, 127.9, 127.8, 127.7, 127.6, 127.2, 126.5, 126.0, 126.0, 123.0, 120.4, 24.3 ppm.

IR (film): ν_{max} 2954, 2922, 2849, 1492, 1444, 823, 752, 738, 696 cm^{-1}.

HRMS (ESI): m/z calculated for $C_{33}H_{25}N_2^+$ $[M+H]^+$ 449.2012, found 449.2020.

HPLC analysis: HPLC conditions: Chiral column IB, *n*-hexane/isopropanol: 93/7, flow rate = 1 mL/min, wavelength = 254 nm, t_R = 10.920 min for major isomer, t_R = 12.613 min for minor isomer, 96% ee.

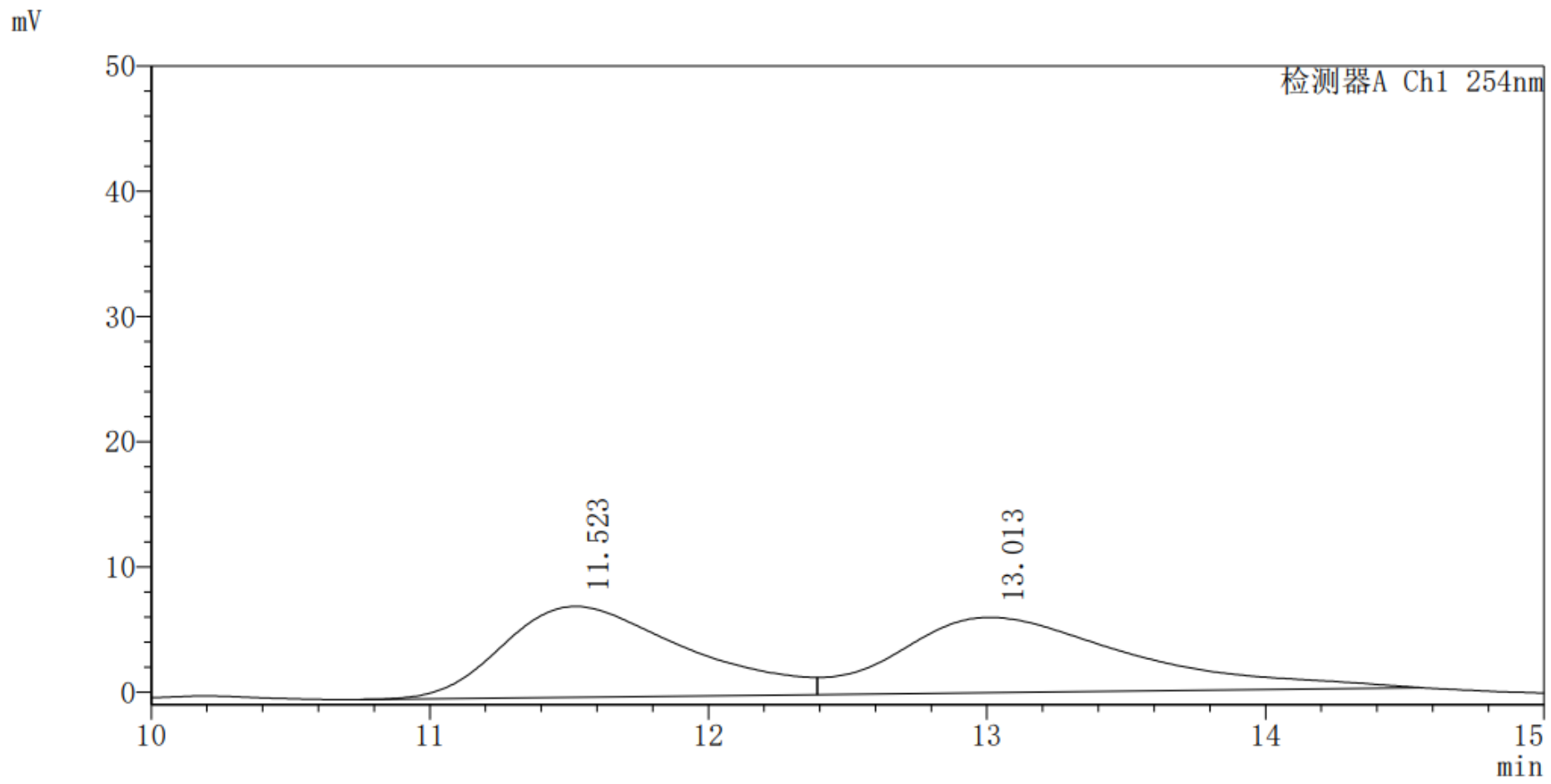

检测器A Ch1 254nm

No.	R. Time	Height	Height%	Area	Area%
1	11.523	7263	54.744	333261	49.116
2	13.013	6005	45.256	345261	50.884

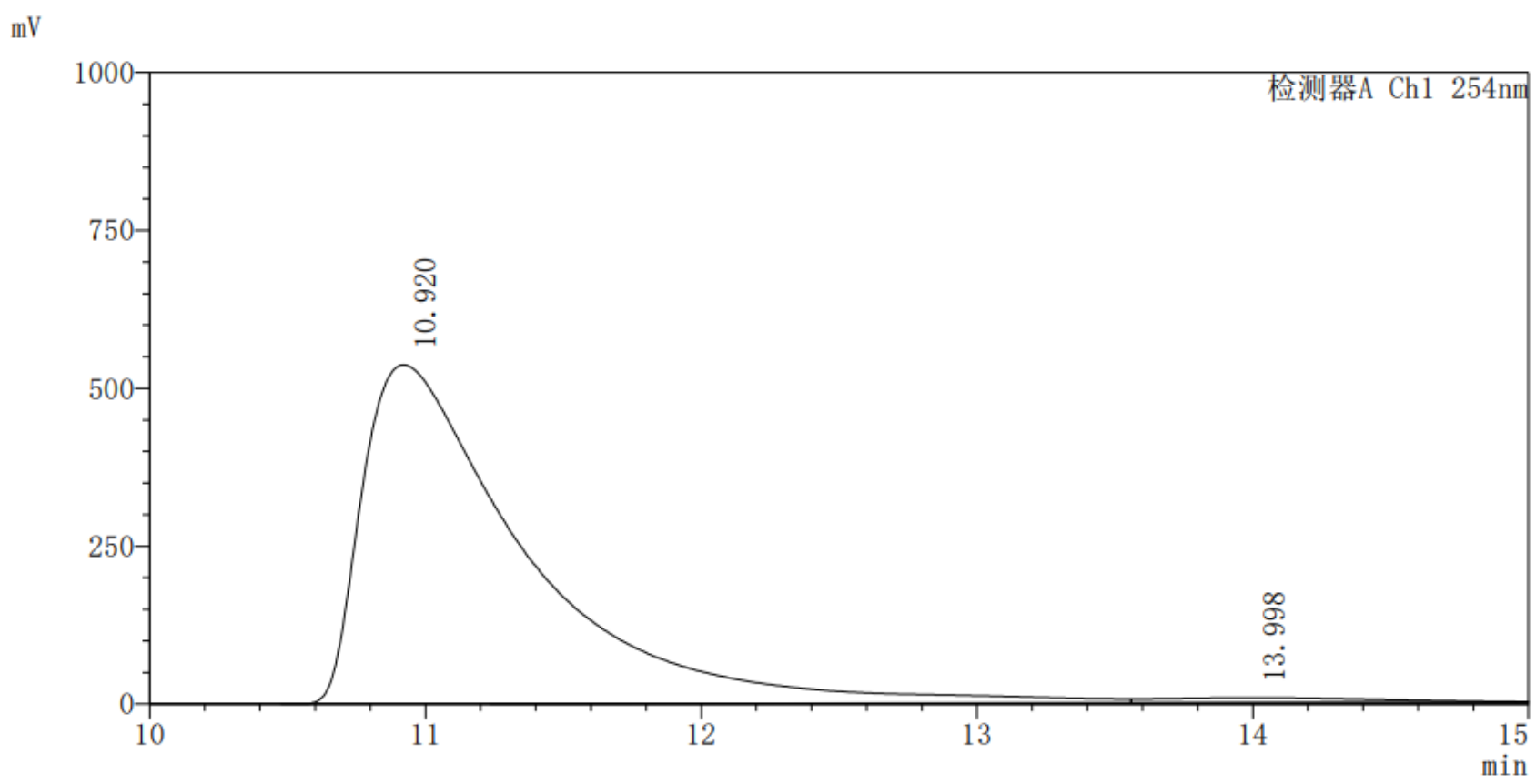

检测器A Ch1 254nm

No.	R. Time	Height	Height%	Area	Area%
1	10.920	537553	98.588	22363845	98.110
2	13.998	7698	1.412	430906	1.890
总计		545252	100.000	22794751	100.000

(*R*,*Z*)-1-{2-[2-(1-Methyl-1*H*-indol-5-yl)-2-phenylvinyl]naphthalen-1-yl}isoquinoline (56)

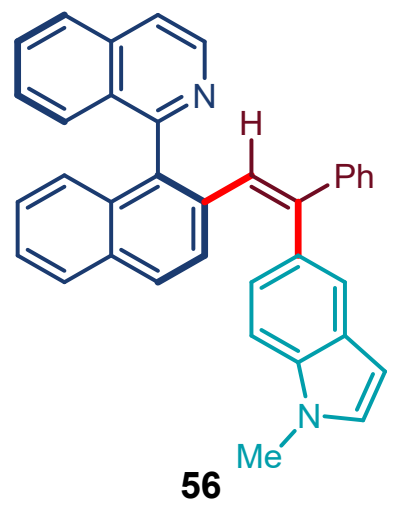

56

Prepared following **Procedure F**, using compound **3** (0.1 mmol, 1.0 equiv., 52.1 mg), 5-bromo-1-methyl-1*H*-indole (84.0 mg, 0.4 mmol, 4.0 equiv.), $Pd(PPh_3)_4$ (0.01 mmol, 10 mol%, 11.6 mg), NaOH (0.4 mmol, 4.0 equiv., 16.0 mg, dissolved in 0.5 mL H_2O), anhydrous THF (1.5 mL). Purification by flash column chromatography (Petroleum ether/EtOAc: 5/1) to afford the title compound (45.7 mg, 94%) as a white solid.

TLC: R_f = 0.4 (Petroleum ether/EtOAc: 5/1, $KMnO_4$ stain).

M. p.: 95 – 96 °C.

$[\alpha]^{18}_D$: +319.63 (c 0.20, CH_2Cl_2).

NMR Spectroscopy (*see spectra*):

^{1}H NMR (400 MHz, $CDCl_3$) δ_H = 8.76 (d, *J* = 5.8 Hz, 1H), 7.91 (d, *J* = 8.4 Hz, 1H), 7.72 (dd, *J* = 6.9, 11.0 Hz, 3H), 7.60 (d, *J* = 8.4 Hz, 1H), 7.52 – 7.43 (m, 3H), 7.35 (t, *J* = 7.5 Hz, 1H), 7.23 – 7.05 (m, 10H), 6.95 (d, *J* = 8.5 Hz, 1H), 6.59 (s, 1H), 6.41 (s, 1H), 3.79 (s, 3H) ppm;

^{13}C NMR (100 MHz, $CDCl_3$) δ_C = 160.4, 145.1, 144.5, 143.0, 136.5, 136.4, 136.2, 135.7, 133.1, 132.3, 131.6, 130.4, 129.2, 128.8, 128.6, 128.4, 128.0, 127.9, 127.8, 127.5, 127.5, 127.5, 127.3, 127.2, 126.2, 126.0, 125.6, 125.6, 125.1, 123.5, 120.3, 109.2, 101.5, 33.0 ppm.

IR (film): ν_{max} 3052, 2923, 2851, 1555, 1510, 1490, 1443, 1365, 1326, 1242, 905, 821, 725, 696 cm^{-1}.

HRMS (ESI): m/z calculated for $C_{36}H_{27}N_2^+$ $[M+H]^+$ 487.2169, found 487.2179.

HPLC analysis: HPLC conditions: Chiral column IB, *n*-hexane/isopropanol: 95/5, flow rate = 1 mL/min, wavelength = 254 nm, t_R = 14.006 min for major isomer, t_R = 16.704 min for minor isomer, 97% ee.

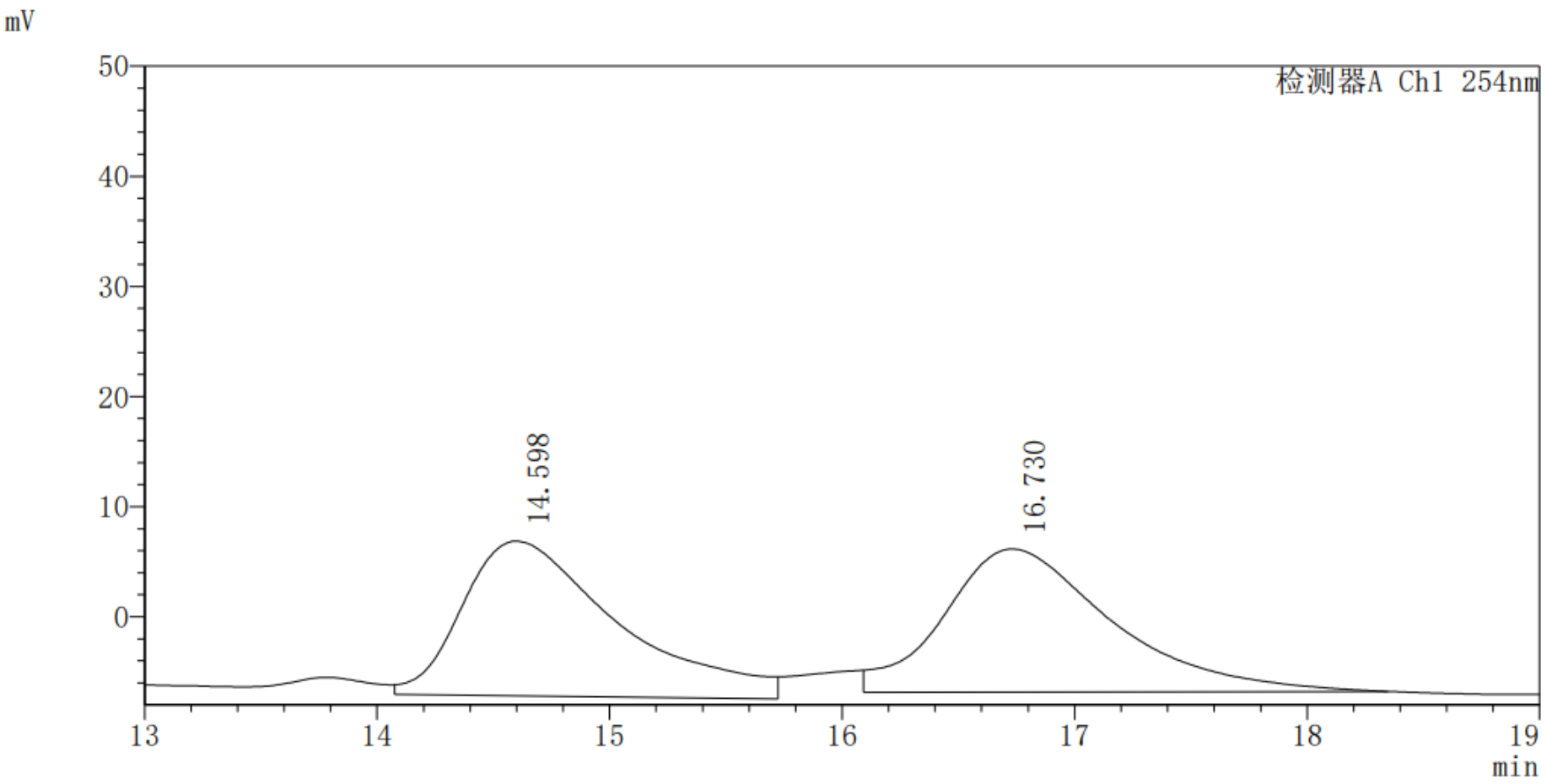

检测器A Ch1 254nm

No.	R.Time	Height	Height%	Area	Area%
1	14.598	14058	51.975	654421	50.087
2	16.730	12989	48.025	652150	49.913

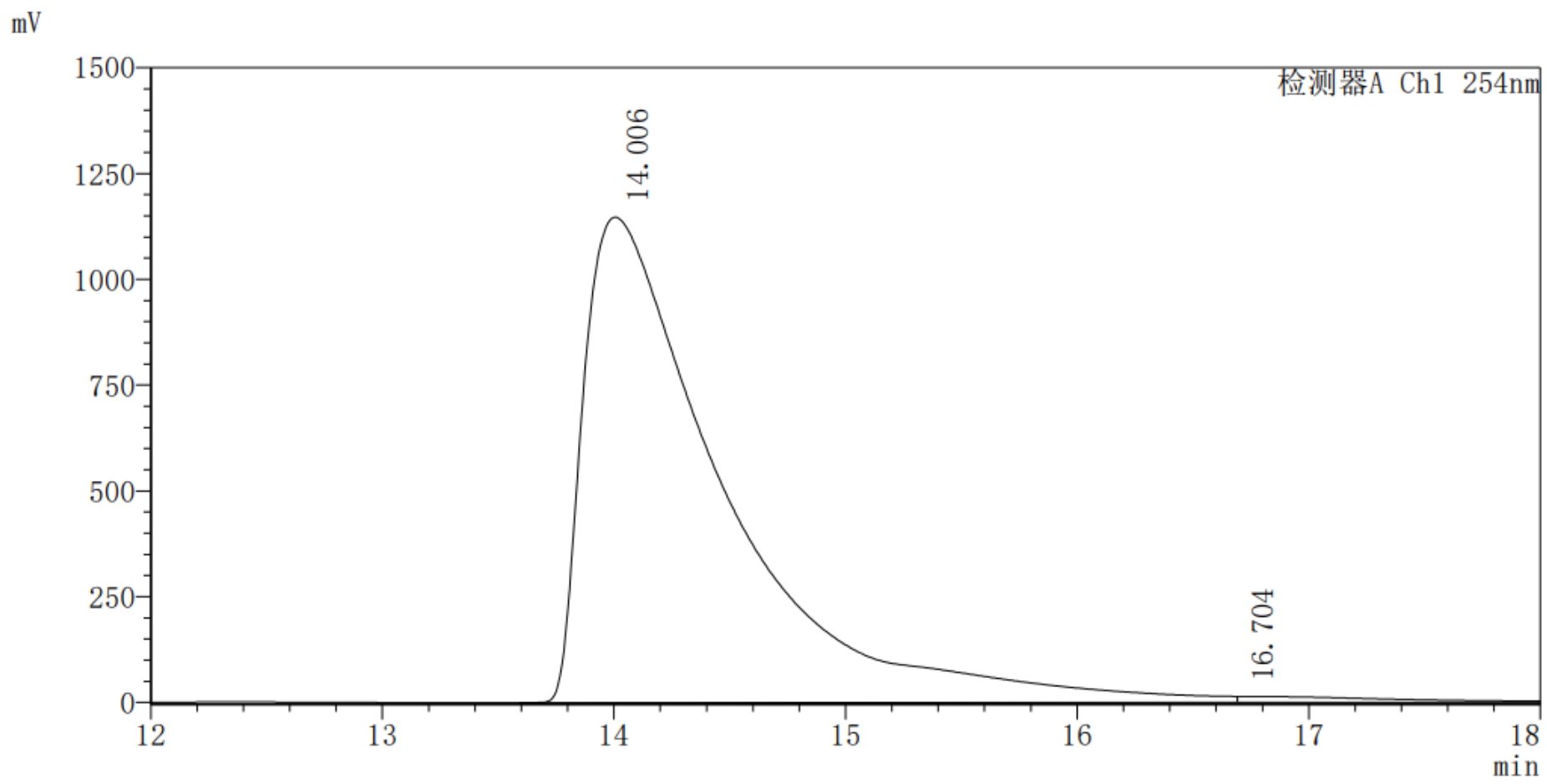

检测器A Ch1 254nm

No.	R.Time	Height	Height%	Area	Area%
1	14.006	1147300	98.764	48651364	98.452
2	16.704	14359	1.236	765206	1.548

5.2.2 Direct Oxidation of Compound 31

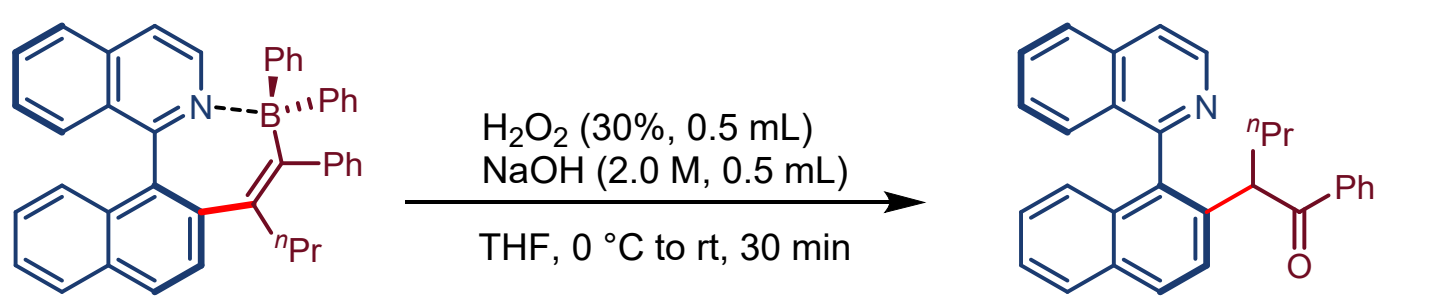

31, 94% *ee* **57**, 95%, 94% *ee*, 1.4:1.0 *dr*

A dried Schlenk tube was charged with **31** (0.1 mmol, 1.0 equiv., 59.1 mg) and NaOH (2.0 M, 0.5 mL). The anhydrous THF (1.0 mL) was added to Schlenk tube at room temperature. Then, H_2O_2 (2.0 M, 0.5

mL) was added to the reaction system at 0 °C. The reaction was then warmed to room temperature and stirred at the same temperature for 30 min. The aqueous layer was extracted with EtOAc (3 × 10 mL), and the combined organic layer was dried over anhydrous $MgSO_4$, filtered, concentrated. The crude material was purified by flash column chromatography (*n*-hexane/EtOAc = 10/1) to afford the desired product **57** (39.5 mg, 95%) as a white solid.

(*R*)-2-[1-(Isoquinolin-1-yl)naphthalen-2-yl]-1-phenylpentan-1-one (57)

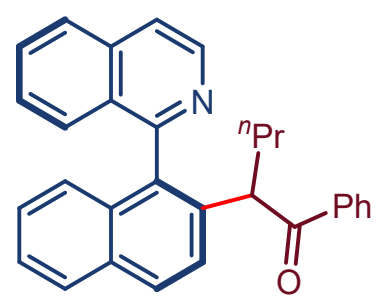

57

$[\alpha]^{18}_{D}$: +196.90 (c 0.20, CH_2Cl_2).

NMR Spectroscopy (*see spectra*):

^{1}H NMR (500 MHz, $CDCl_3$) δ_H = 8.82 (d, *J* = 5.7 Hz, 0.41H), 8.73 (d, *J* = 5.8 Hz, 0.59H), 7.99 (td, *J* = 6.6, 6.1, 3.1 Hz, 2H), 7.94 – 7.80 (m, 3.41H), 7.72 (dt, *J* = 8.1, 4.0 Hz, 0.59H), 7.61 (d, *J* = 8.7 Hz, 0.59H), 7.54 (dt, *J* = 8.1, 3.9 Hz, 0.41H), 7.48 – 7.33 (m, 4H), 7.25 – 7.20 (m, 1.23H), 7.16 – 7.10 (m, 1.77H), 7.07 (d, *J* = 3.4 Hz, 0.59H), 7.03 (d, *J* = 8.5 Hz, 0.41H), 6.98 (d, *J* = 7.8 Hz, 0.41H), 6.93 (t, *J* = 7.9 Hz, 0.59H), 6.85 (d, *J* = 7.5 Hz, 0.41H), 6.68 (d, *J* = 7.5 Hz, 0.59H), 4.36 (ddd, *J* = 15.9, 9.0, 5.0 Hz, 1H), 2.33 – 2.12 (m, 1H), 2.08 – 1.98 (m, 0.41H), 1.46 – 1.36 (m, 0.82H), 1.20 (td, *J* = 14.0, 13.4, 5.9 Hz, 0.59H), 1.06 – 0.93 (m, 0.59H), 0.81 (t, *J* = 7.4 Hz, 1.23H), 0.79 – 0.67 (m, 0.59H), 0.29 (t, *J* = 7.4 Hz, 1.82H) ppm;

^{13}C NMR (100 MHz, $CDCl_3$) δ_C = 201.7, 200.8, 160.0, 159.9, 156.1, 142.7, 142.51, 137.2, 137.1, 137.0, 136.5, 136.4, 136.3, 135.2, 134.6, 133.1, 132.9, 132.8, 132.50, 132.45, 130.7, 130.6, 129.6, 129.3, 129.2, 129.02, 129.01, 129.0, 128.5, 128.08, 128.05, 127.9, 127.8, 127.7, 127.6, 127.2, 126.9, 126.6, 126.5, 126.27, 126.25, 125.8, 125.7, 125.3, 120.8, 120.7, 120.3, 115.5, 49.8, 49.1, 36.5, 35.9, 21.4, 21.1, 14.0, 13.3 ppm.

IR (film): ν_{max} 3055, 2956, 2929, 1679, 1594, 1499, 1447, 1239, 1207, 824, 749, 698 cm^{-1}.

HRMS (ESI): m/z calculated for $C_{30}H_{26}NO^+$ $[M+H]^+$ 416.2009, found 416.2009.

HPLC analysis: HPLC conditions: Chiral column AD-H, *n*-hexane/isopropanol: 95/5, flow rate = 1 mL/min, wavelength = 254 nm, t_R = 10.185 min for major isomer, t_R = 8.357 min for minor isomer, 94% ee.

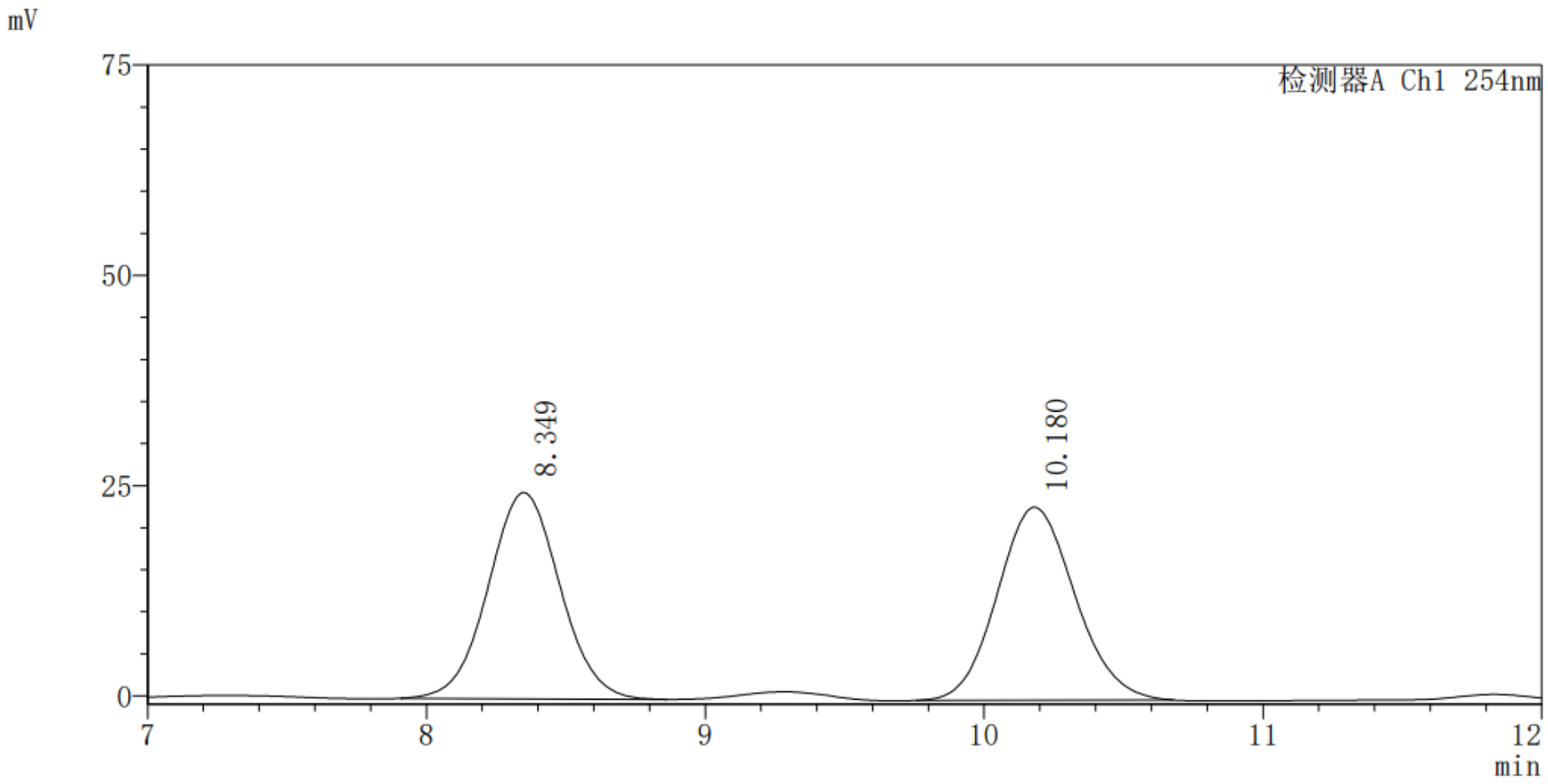

检测器A Ch1 254nm

No.	R.Time	Height	Height%	Area	Area%
1	8.349	24515	51.663	438061	49.699
2	10.180	22937	48.337	443368	50.301

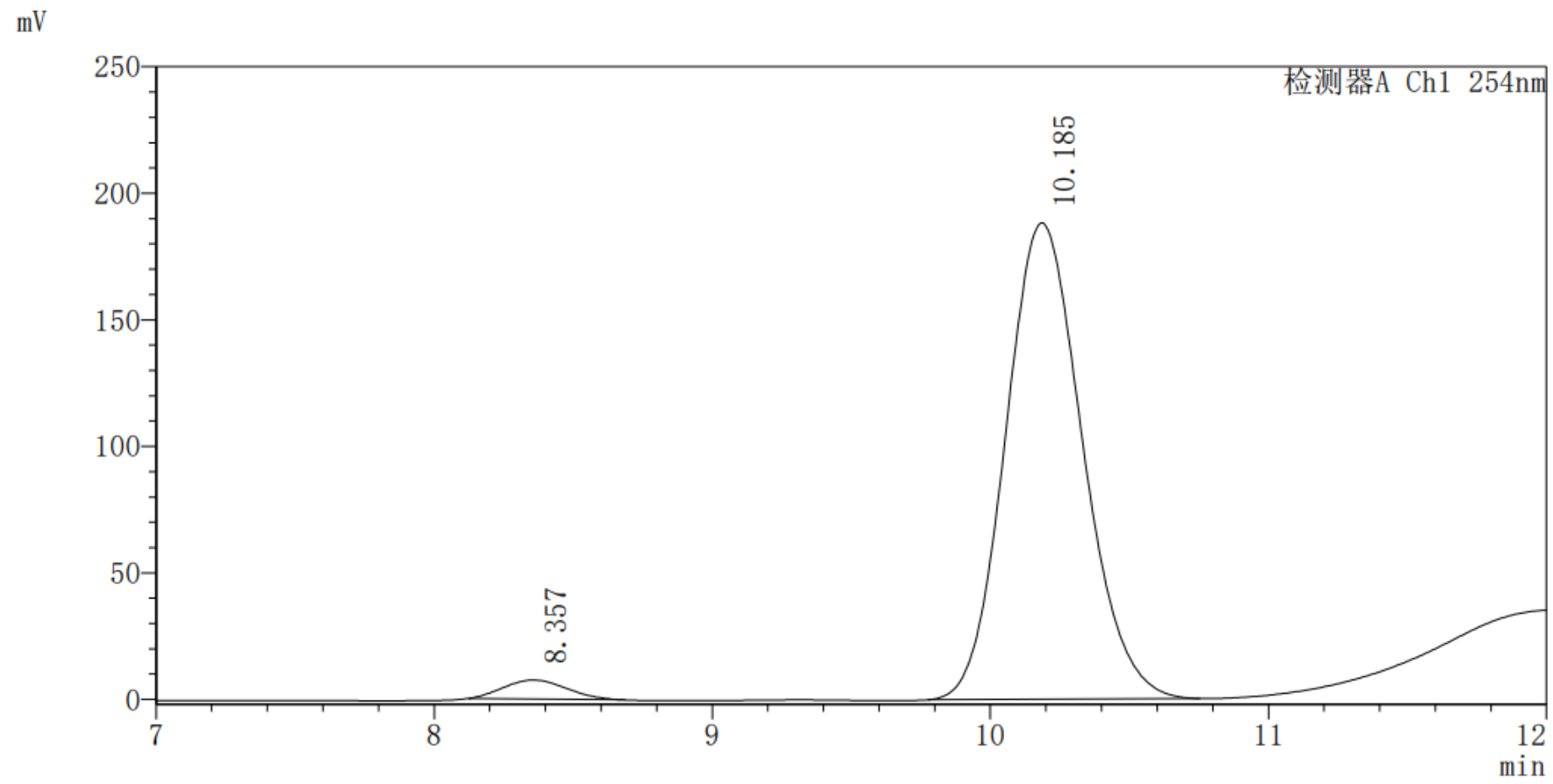

检测器A Ch1 254nm

No.	R.Time	Height	Height%	Area	Area%
1	8.357	7495	3.829	118143	3.170
2	10.185	188262	96.171	3609211	96.830

6. X-RAY CRYSTALLOGRAPHIC DATA

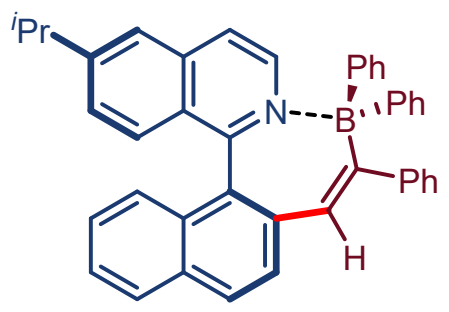

4

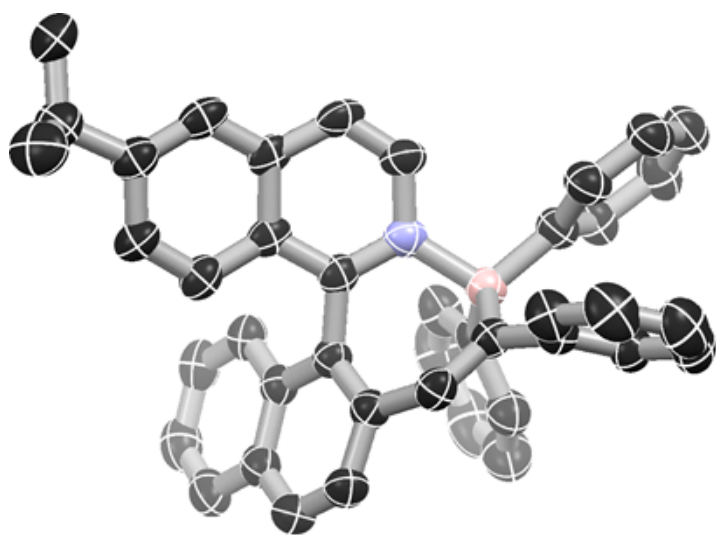

R-Xay of 4
CCDC: 2475423

ORTEP drawing at the 50% probability level.

Table S11: Crystal Data and Structure Refinement for 20250724b.

Identification code	20250724b
Empirical formula	$C_{42}H_{34}BN$
Formula weight	563.51
Temperature/K	298(2)
Crystal system	orthorhombic
Space group	$P2_12_12_1$
a/Å	8.7555(2)
b/Å	14.1862(3)
c/Å	25.5392(6)
α/°	90
β/°	90
γ/°	90
Volume/Å^3	3172.15(12)
Z	4
ρ_{calc}g/cm^3	1.180
μ/mm^{-1}	0.506
F(000)	1192.0

Crystal size/mm^3	0.5265 × 0.4865 × 0.439
Radiation	CuKα (λ = 1.54178)
2Θ range for data collection/°	7.128 to 144.894
Index ranges	-10 ≤ h ≤ 10, -17 ≤ k ≤ 17, -31 ≤ l ≤ 31
Reflections collected	31885
Independent reflections	6182 [R_{int} = 0.0484, R_{sigma} = 0.0347]
Data/restraints/parameters	6182/406/502
Goodness-of-fit on F^2	1.086
Final R indexes [I>=2σ (I)]	R_1 = 0.0409, wR_2 = 0.1075
Final R indexes [all data]	R_1 = 0.0444, wR_2 = 0.1119
Largest diff. peak/hole / e Å^{-3}	0.19/-0.15
Flack parameter	0.1(2)

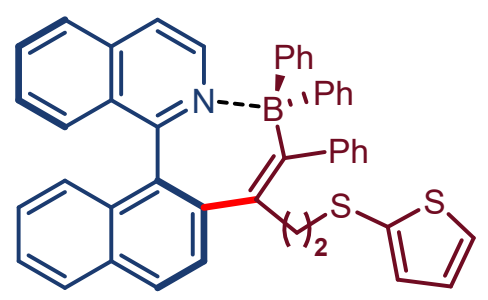

46

≡

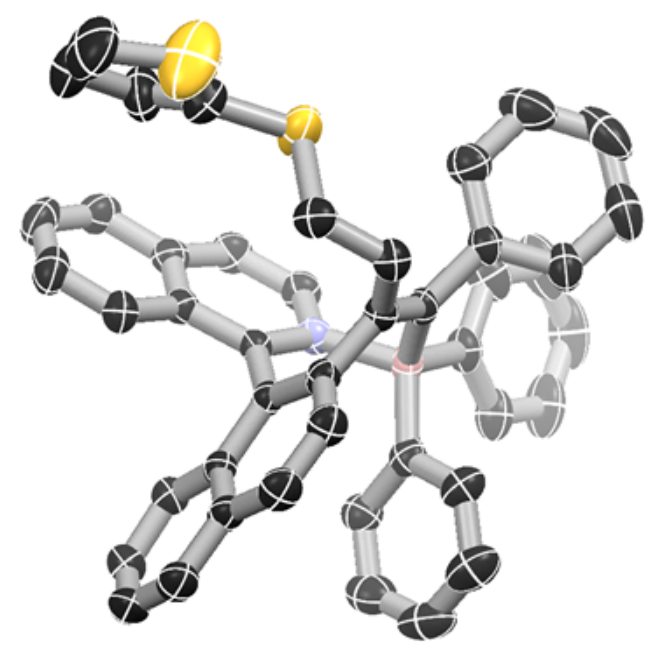

R-Xay of **46**
CCDC: 2477847

ORTEP drawing at the 50% probability level.

Table S12: Crystal Data and Structure Refinement for 20250801b.

Identification code	20250801b
Empirical formula	C45H34BNS2
Formula weight	663.66
Temperature/K	298(2)

Crystal system	orthorhombic
Space group	P212121
a/Å	12.1726(12)
b/Å	13.5587(14)
c/Å	21.354(3)
α/°	90
β/°	90
γ/°	90
Volume/Å3	3524.3(7)
Z	4
ρcalcg/cm3	1.251
μ/mm-1	0.185
F(000)	1392.0
Crystal size/mm3	0.4079 × 0.3885 × 0.3643
Radiation	MoKα (λ = 0.71073)
2Θ range for data collection/°	5.074 to 55
Index ranges	-15 ≤ h ≤ 15, -17 ≤ k ≤ 17, -27 ≤ l ≤ 27
Reflections collected	47886
Independent reflections	8067 [Rint = 0.0628, Rsigma = 0.0521]
Data/restraints/parameters	8067/241/488
Goodness-of-fit on F2	1.022
Final R indexes [I>=2σ (I)]	R1 = 0.0509, wR2 = 0.1020
Final R indexes [all data]	R1 = 0.0874, wR2 = 0.1173
Largest diff. peak/hole / e Å-3	0.30/-0.28
Flack parameter	-0.01(3)

7. COMPUTATIONAL DETAILS

The DFT-calculation was performed at B3LYP/6-311G(d,p) level of theory with GD3BJ dispersion correction for structure optimization.

Table S13: The DFT calculation for the rotational barrier of compound 3.

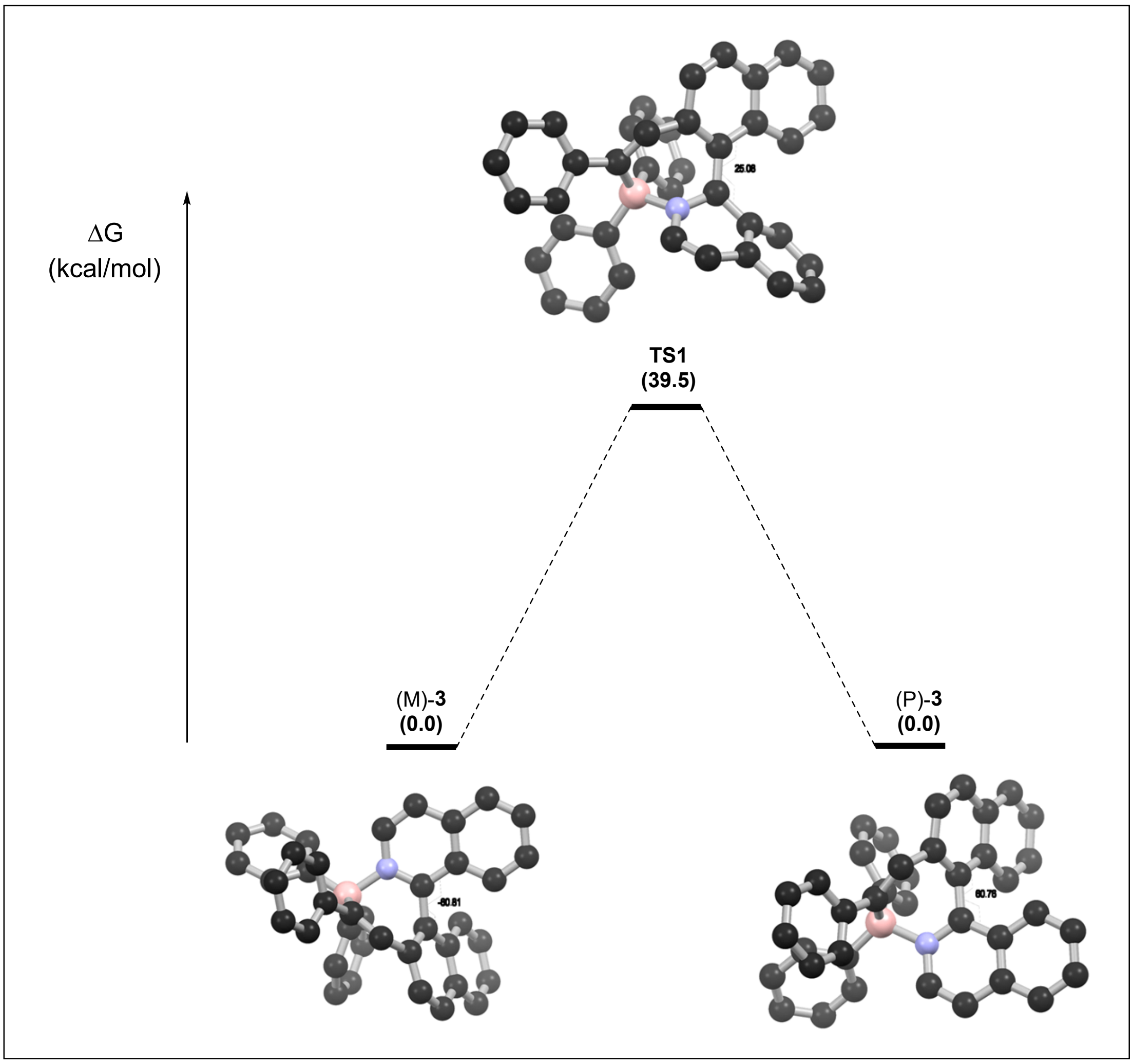

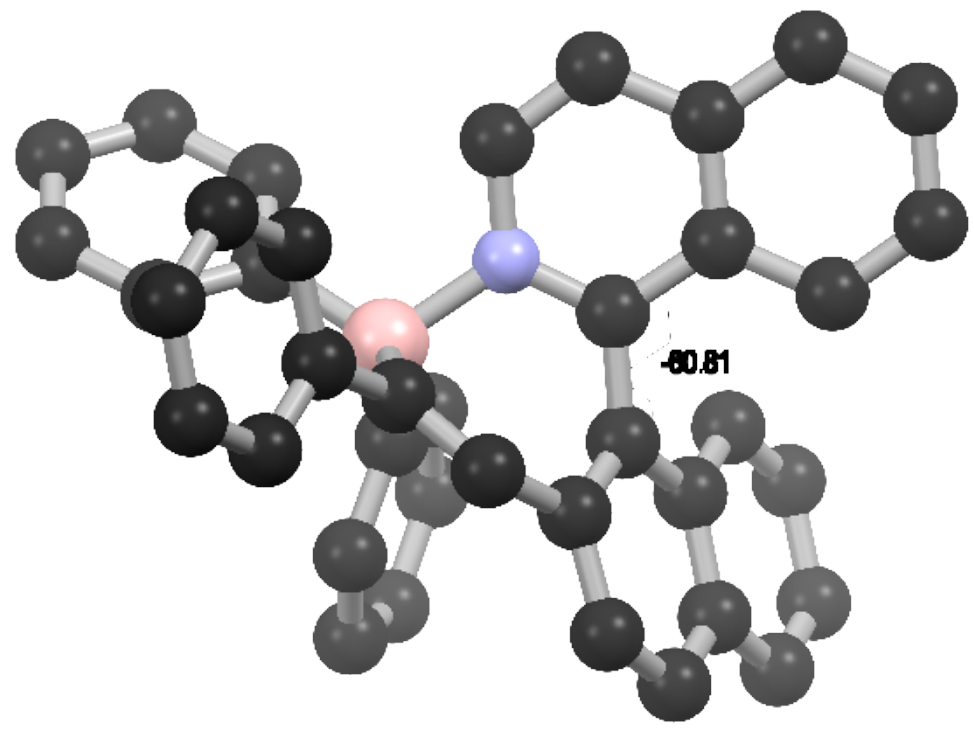

(M)-**3**

C	-1.78372597	2.32186525	0.17338406
C	-1.25645998	3.39571508	0.94754567
C	-1.03895497	1.09961443	0.07502959
N	0.16871714	0.99890685	0.64847085
C	0.69752359	2.06030891	1.33552034
C	0.02220058	3.22412055	1.52567947
C	-1.56557353	-0.00546033	-0.75620198
C	-2.83364464	-0.59065734	-0.43804693
C	-3.42561859	-1.50719775	-1.35613309
C	-2.75897027	-1.78125809	-2.57851778
C	-1.53193818	-1.24385905	-2.83909935
C	-0.87484560	-0.39832976	-1.90300282
C	0.51310565	-0.03804646	-2.16839225
C	1.48712059	-0.00027199	-1.23102578
B	1.12615032	-0.31423580	0.33636075
C	-1.98998329	4.59954176	1.07252866
C	-3.19115143	4.74892717	0.42590588

C	-3.69416265	3.70818733	-0.38590655
C	-3.00843826	2.52640564	-0.51561549
C	-3.49513707	-0.35739354	0.79385346
C	-4.68271573	-0.97986190	1.09128314
C	-5.27436405	-1.87245872	0.17266424
C	-4.65331333	-2.12919318	-1.02493340
C	2.84253989	0.38524088	-1.69443070
C	3.55321000	1.41388846	-1.06064742
C	4.81536119	1.78956108	-1.50333035
C	5.40801760	1.13179122	-2.57991225
C	4.71952720	0.10170807	-3.21460915
C	3.44855000	-0.26338234	-2.77895076
C	0.26340864	-1.67003234	0.57256961
C	-0.57862522	-1.81052538	1.68378273
C	-1.28425346	-2.98351383	1.93139589
C	-1.14808134	-4.07219658	1.07426834
C	-0.29075429	-3.97064618	-0.01890134
C	0.40308724	-2.78774982	-0.25897336
C	2.35583067	-0.43585271	1.38673225
C	2.20312299	-0.20017298	2.76455399
C	3.21448133	-0.46492756	3.68407188
C	4.42396686	-1.00413620	3.25438422
C	4.59253820	-1.28589116	1.90206911
C	3.57567220	-1.00734080	0.99222249

H	1.68851576	1.89921851	1.72656967
H	0.47760827	4.02287190	2.09727362
H	-3.22669199	-2.45437788	-3.28859891
H	-1.00579171	-1.49722369	-3.75229211
H	0.76190130	0.14097525	-3.21335441
H	-1.57934859	5.40111506	1.67539413
H	-3.74886590	5.67316408	0.52027215
H	-4.62700946	3.84789080	-0.91837826
H	-3.39766259	1.74350305	-1.14825277
H	-3.04236500	0.30204385	1.52200345
H	-5.16146839	-0.79653649	2.04609432
H	-6.20888799	-2.36159538	0.42058555
H	-5.08856185	-2.82547572	-1.73333946
H	3.11082563	1.92050561	-0.21306877
H	5.34382088	2.59147552	-1.00023579
H	6.39872477	1.41563848	-2.91543569
H	5.17415034	-0.42391932	-4.04695361
H	2.92410401	-1.07925568	-3.26289295
H	-0.70593254	-0.97644496	2.36708873
H	-1.94624623	-3.04593049	2.78842187
H	-1.69860446	-4.98793500	1.25798878
H	-0.16716096	-4.81393236	-0.69044191
H	1.05223457	-2.72391938	-1.12556648
H	1.26132927	0.18176708	3.14555913

H	3.05205391	-0.26272416	4.73758006
H	5.21594377	-1.21427254	3.96444426
H	5.52003781	-1.72466272	1.55006841
H	3.74026034	-1.25225142	-0.04851820

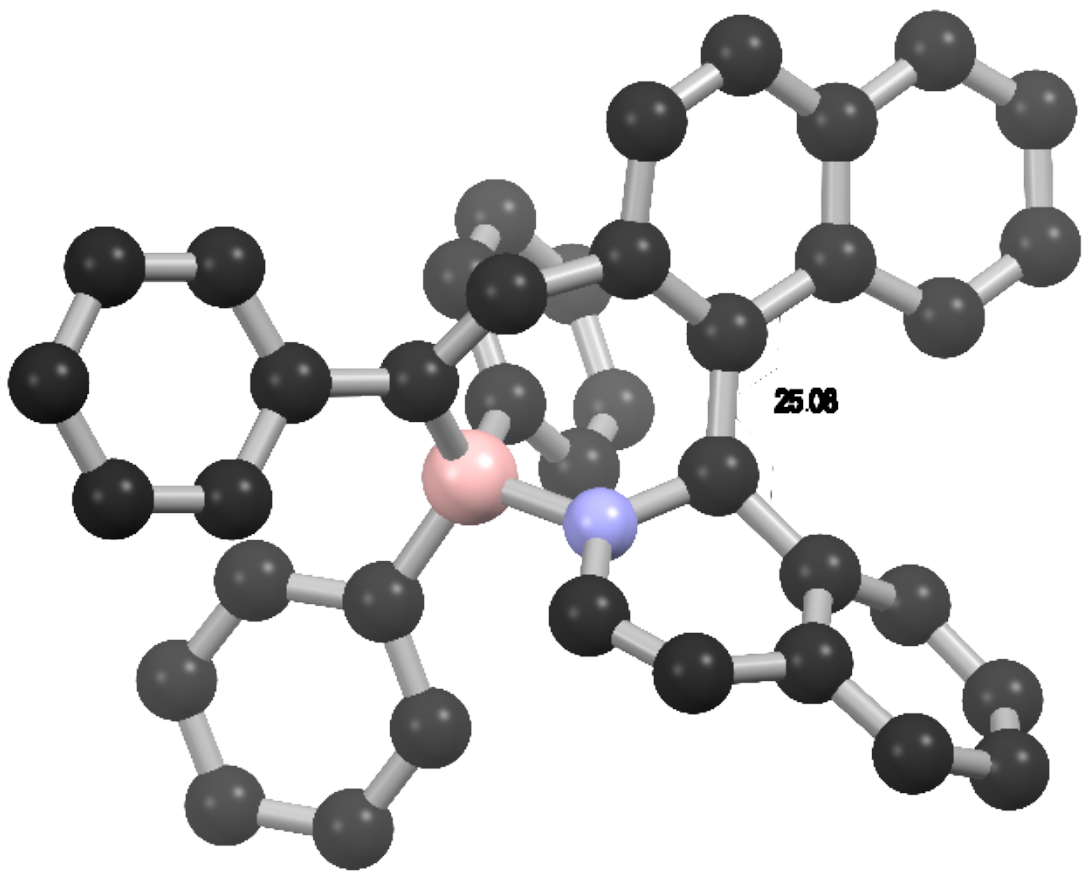

TS

C	1.90590218	1.99116197	-0.81194230
C	1.36476341	2.90272943	-1.76827648
C	1.16554145	0.73031770	-0.61670522
N	-0.17734973	0.93031114	-0.61869830
C	-0.73485227	1.85074414	-1.47345127
C	0.01758622	2.71659062	-2.19317288
C	1.62614633	-0.66667244	-0.64844002
C	2.99708528	-1.12815463	-0.37789598
C	3.34354796	-2.50864897	-0.54870484
C	2.41064095	-3.40129224	-1.12126448
C	1.17929761	-2.96046173	-1.46773021
C	0.71022182	-1.64377731	-1.17500461

C	-0.72055508	-1.58187250	-1.47053535
C	-1.70293260	-0.96027466	-0.77450978
B	-1.17215241	0.08238184	0.33300635
C	2.08059044	4.07383877	-2.09960593
C	3.19307289	4.44665205	-1.37911246
C	3.56912642	3.68584209	-0.26196213
C	2.93593296	2.48925920	0.01347841
C	4.06605157	-0.31446115	0.05416257
C	5.32019608	-0.79947726	0.36006645
C	5.61125256	-2.16553822	0.25511760
C	4.62547649	-2.99849740	-0.21113899
C	-3.10215559	-1.25052274	-1.13996313
C	-4.05129583	-0.22593538	-1.28358301
C	-5.35742883	-0.51003487	-1.66405781
C	-5.75744820	-1.82648017	-1.88322072
C	-4.83515403	-2.85803630	-1.72454593
C	-3.52301254	-2.57228799	-1.36354859
C	-0.33447560	-0.61094527	1.53988410
C	0.44929073	0.18364650	2.39092882
C	1.19900268	-0.36590393	3.42506930
C	1.16214833	-1.74038437	3.65937725
C	0.36250668	-2.54458693	2.85434450
C	-0.37014070	-1.98210364	1.80875214
C	-2.23310099	1.08474942	1.03291747

C	-2.05118425	2.46534793	1.18897957
C	-2.95082724	3.24985160	1.91210374
C	-4.06277018	2.66703369	2.50973115
C	-4.25559677	1.29115319	2.38891933
C	-3.35180556	0.51960226	1.66803325
H	-1.81114876	1.81816015	-1.53294494
H	-0.43334656	3.37671062	-2.92146269
H	2.69990745	-4.43221001	-1.29130483
H	0.47518779	-3.64675716	-1.91982117
H	-1.01282463	-2.27887935	-2.25267261
H	1.68371375	4.71856885	-2.87538190
H	3.71991039	5.36231039	-1.61911302
H	4.34074534	4.04599709	0.40741616
H	3.16665355	1.98193052	0.93698811
H	3.93675184	0.73844022	0.12805793
H	6.08576416	-0.10329978	0.68322412
H	6.59119421	-2.54799702	0.51338768
H	4.81390175	-4.05771865	-0.34624343
H	-3.76621774	0.79786508	-1.08783982
H	-6.06874593	0.29995966	-1.77753058
H	-6.78053987	-2.04683936	-2.16496670
H	-5.13916076	-3.88759219	-1.87625922
H	-2.81586018	-3.38082435	-1.22151316
H	0.47123503	1.25891418	2.24076399

H	1.80525778	0.27558646	4.05623444
H	1.74321922	-2.17482596	4.46506604
H	0.31453061	-3.61361308	3.03330417
H	-0.96589490	-2.63187535	1.17708647
H	-1.18819778	2.95296443	0.74831683
H	-2.77655858	4.31615842	2.01124641
H	-4.76558267	3.27261489	3.07091841
H	-5.11115282	0.81929064	2.86011391
H	-3.51439998	-0.55014913	1.59982462

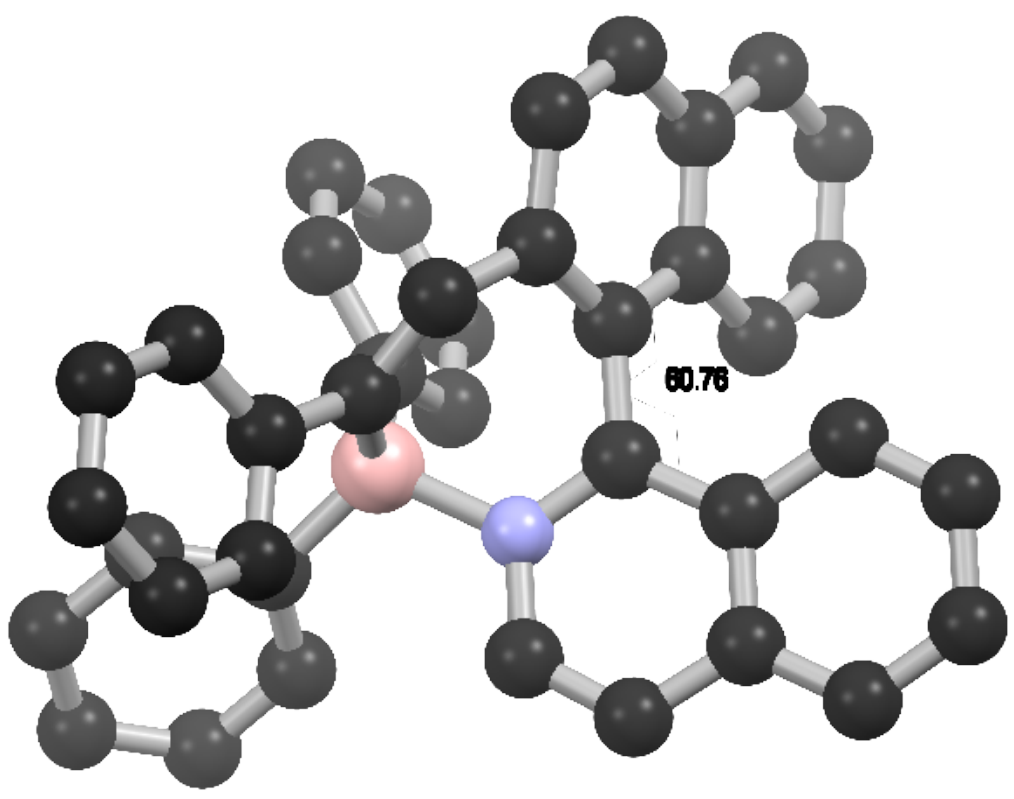

(P)-**3**

C	-1.78408256	2.32177922	-0.17316533
C	-1.25707615	3.39569928	-0.94742562
C	-1.03905246	1.09966131	-0.07480251
N	0.16853131	0.99915140	-0.64838985
C	0.69708524	2.06060070	-1.33555518
C	0.02155445	3.22429319	-1.52568743
C	-1.56548311	-0.00554418	0.75649341

C	-2.83340011	-0.59098142	0.43832073
C	-3.42521117	-1.50770318	1.35636648
C	-2.75836504	-1.78194874	2.57858033
C	-1.53136373	-1.24440198	2.83911096
C	-0.87459627	-0.39848855	1.90315811
C	0.51340477	-0.03808090	2.16856135
C	1.48728644	-0.00031421	1.23110488
B	1.12618443	-0.31416240	-0.33618065
C	-1.99078025	4.59941323	-1.07235380
C	-3.19189503	4.74865640	-0.42560552
C	-3.69469305	3.70784624	0.38624486
C	-3.00878332	2.52616579	0.51590732
C	-3.49504200	-0.35768914	-0.79348230
C	-4.68264997	-0.98015870	-1.09081364
C	-5.27418886	-1.87283574	-0.17220166
C	-4.65294243	-2.12968831	1.02527156
C	2.84277637	0.38528312	1.69446460
C	3.55305797	1.41448089	1.06121595
C	4.81524744	1.79012926	1.50386798
C	5.40830256	1.13175744	2.57985721
C	4.72017591	0.10108480	3.21400298
C	3.44917035	-0.26398320	2.77837365
C	0.26341807	-1.66980755	-0.57253562
C	-0.57861063	-1.80998865	-1.68378950

C	-1.28403044	-2.98302019	-1.93187937
C	-1.14760093	-4.07205027	-1.07524719
C	-0.29030493	-3.97076769	0.01797757
C	0.40331458	-2.78783769	0.25852091
C	2.35569875	-0.43553109	-1.38677549
C	2.20268400	-0.19994896	-2.76458468
C	3.21387889	-0.46458731	-3.68429785
C	4.42356156	-1.00355887	-3.25484092
C	4.59250144	-1.28510654	-1.90253904
C	3.57577125	-1.00667002	-0.99248719
H	1.68808471	1.89970328	-1.72666963
H	0.47679722	4.02308767	-2.09735964
H	-3.22582091	-2.45539615	3.28852924
H	-1.00504017	-1.49794849	3.75214972
H	0.76213940	0.14102140	3.21351256
H	-1.58033371	5.40104304	-1.67527681
H	-3.74973157	5.67282721	-0.51991609
H	-4.62752300	3.84741517	0.91878430
H	-3.39788243	1.74323368	1.14857766
H	-3.04238276	0.30175688	-1.52170737
H	-5.16151436	-0.79669544	-2.04554642
H	-6.20872569	-2.36197785	-0.42005858
H	-5.08803533	-2.82608613	1.73366430
H	3.11040587	1.92157194	0.21404118

H	5.34341278	2.59248839	1.00116726
H	6.39902101	1.41560862	2.91534451
H	5.17507837	-0.42503682	4.04588474
H	2.92503596	-1.08032265	3.26186892
H	-0.70622606	-0.97562363	-2.36668859
H	-1.94600295	-3.04518026	-2.78894532
H	-1.69784781	-4.98787200	-1.25938209
H	-0.16656795	-4.81428567	0.68920200
H	1.05239288	-2.72421972	1.12519134
H	1.26076844	0.18188104	-3.14542537
H	3.05118754	-0.26245157	-4.73777962
H	5.21537722	-1.21368166	-3.96508679
H	5.52015798	-1.72364742	-1.55066255
H	3.74070839	-1.25140718	0.04823326

8. NMR SPECTRA OF ALL COMPOUNDS

^{1}H NMR (500 MHz, $CDCl_3$) of **1b** (*see procedure*)

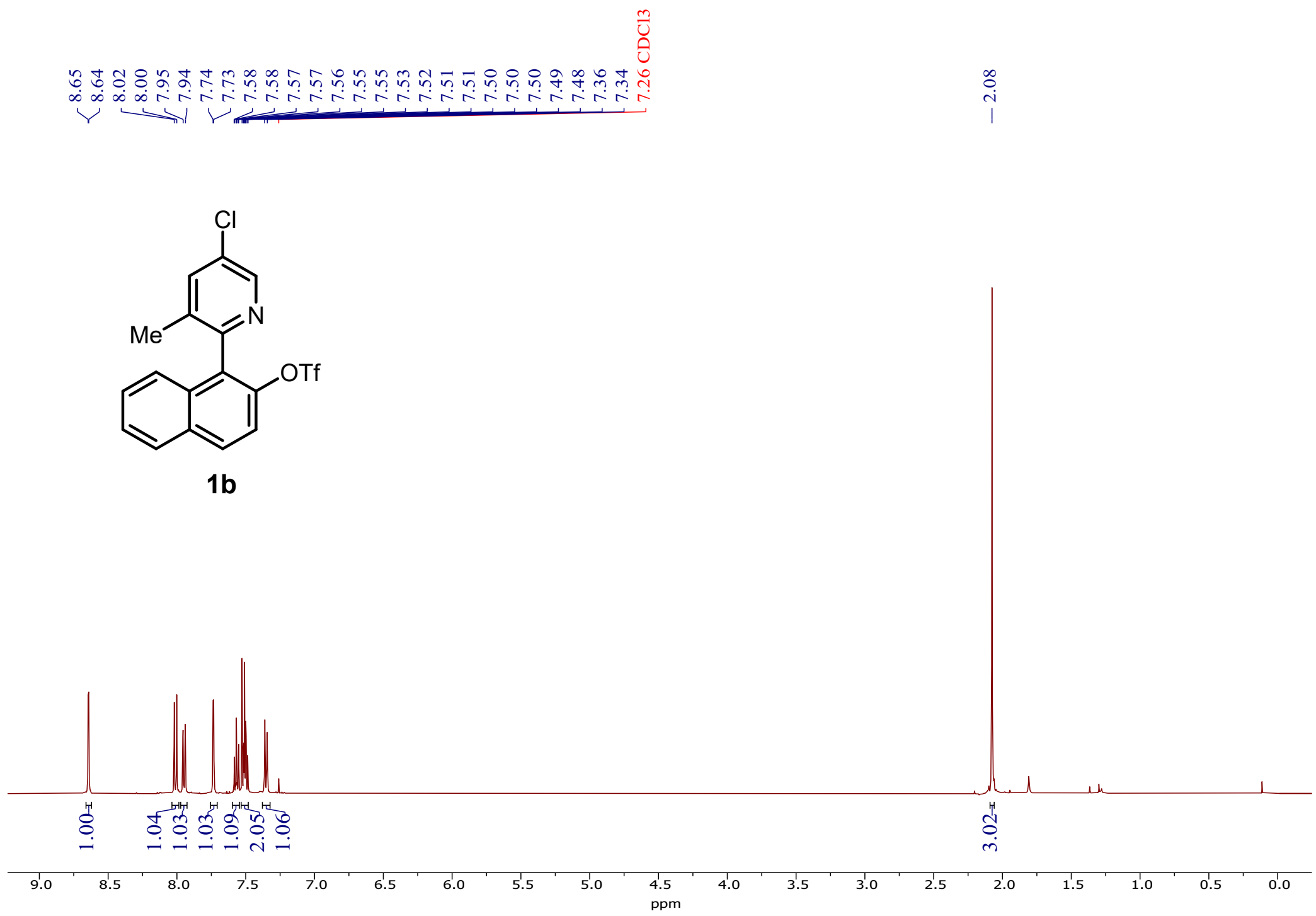

^{13}C NMR (125 MHz, $CDCl_3$) of **1b**

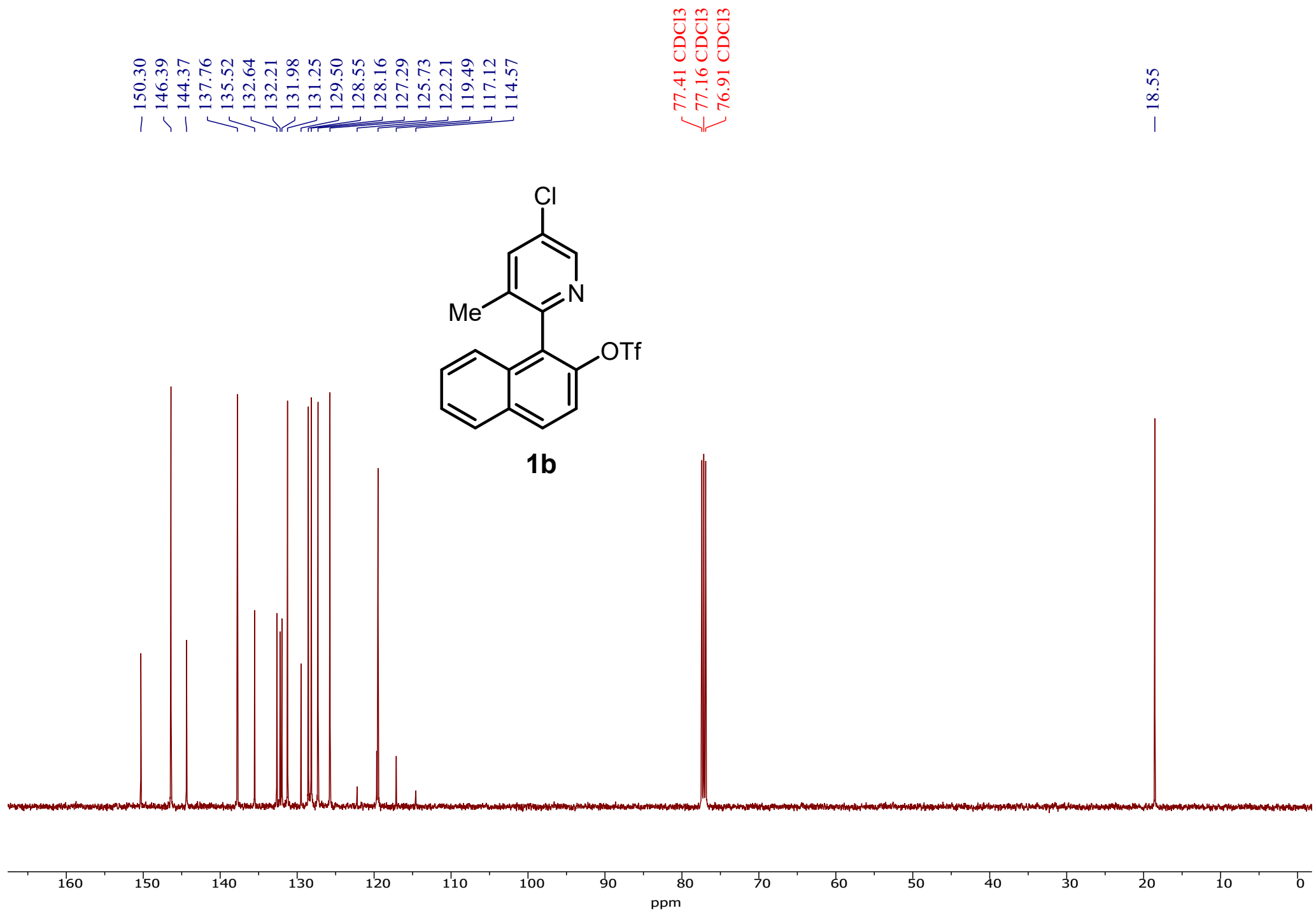

^{19}F NMR (376 MHz, $CDCl_3$) of **1b**

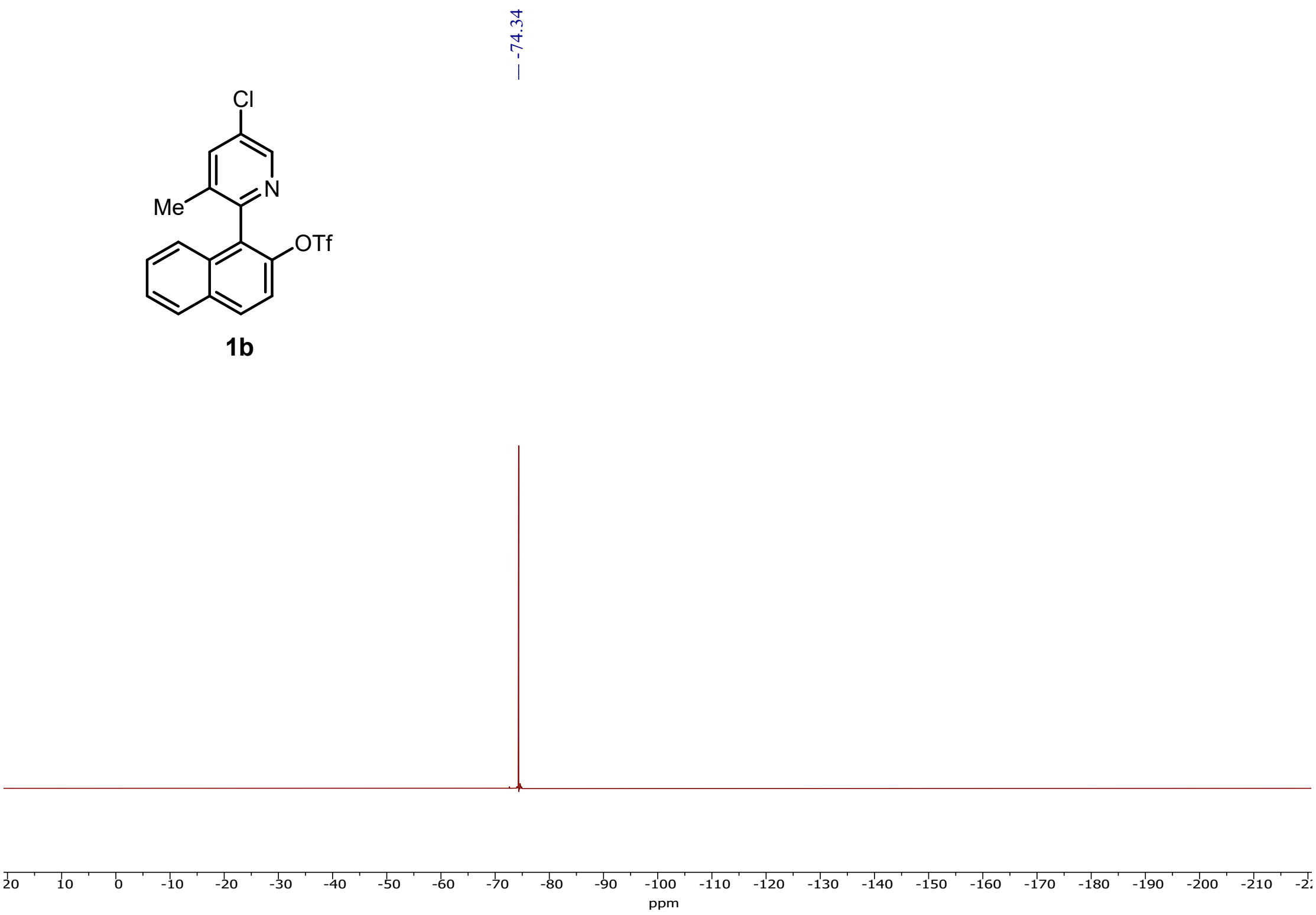

^{1}H NMR (400 MHz, $CDCl_3$) of **1c** (*see procedure*)

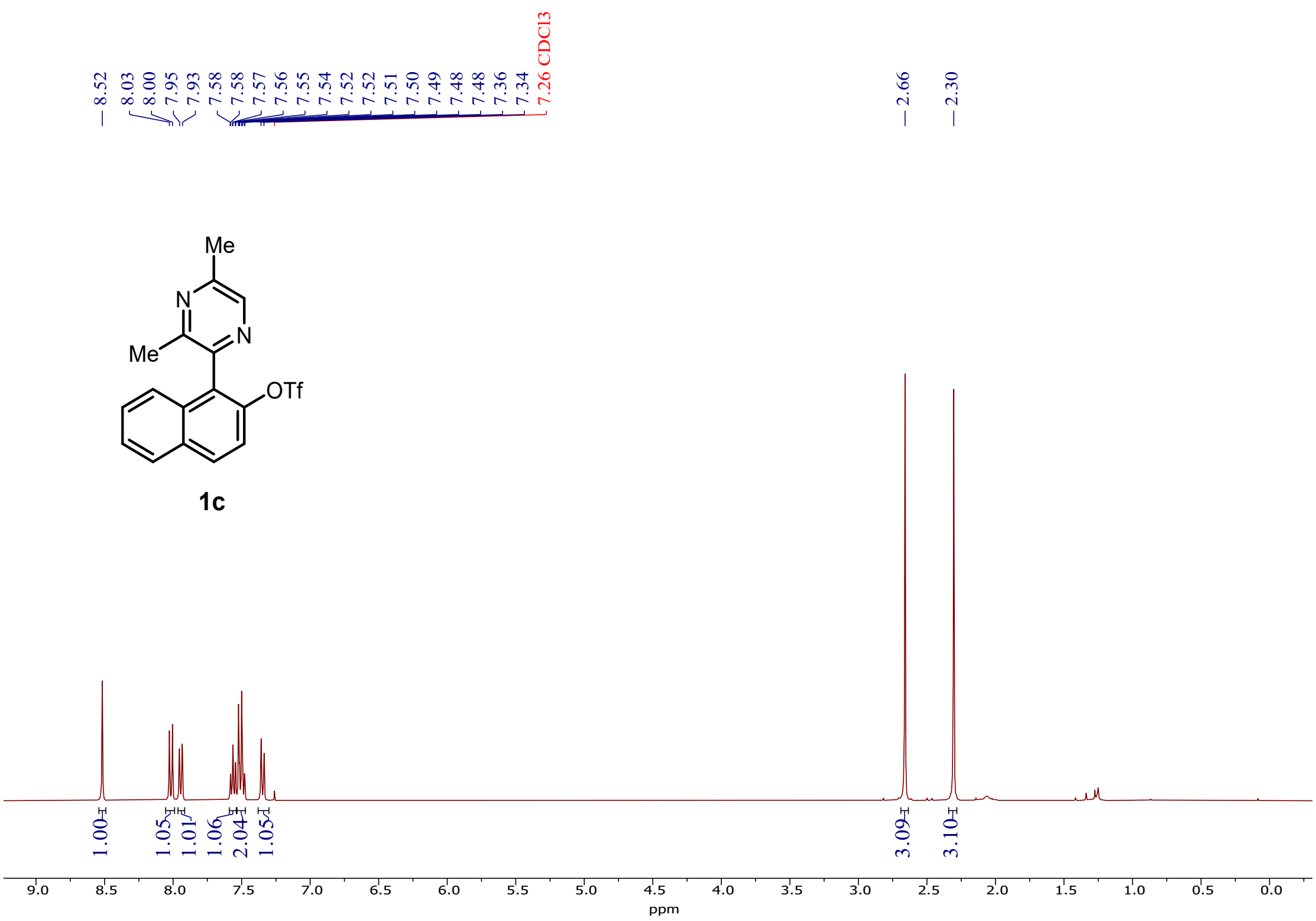

^{13}C NMR (125 MHz, $CDCl_3$) of **1c**

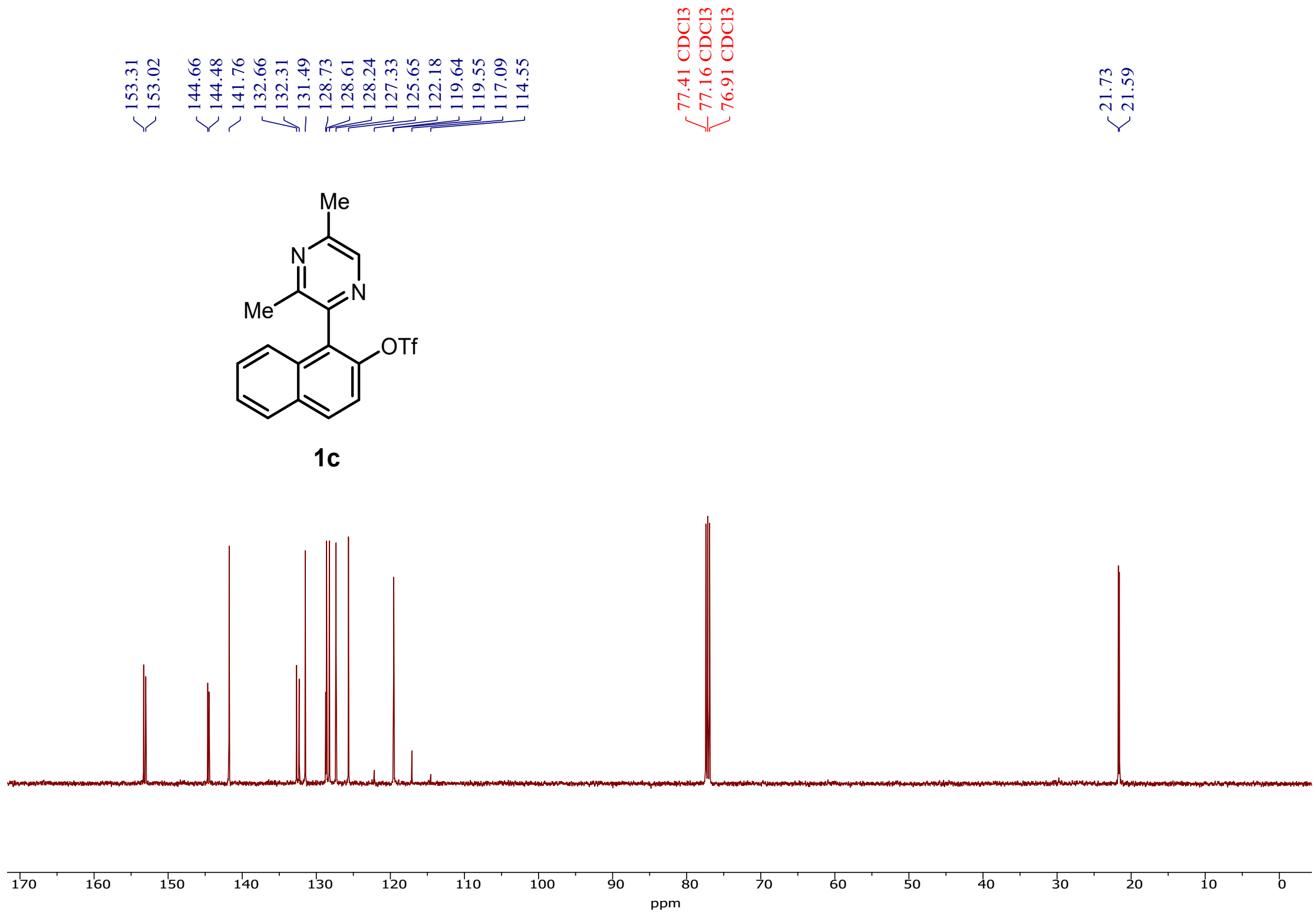

^{19}F NMR (376 MHz, $CDCl_3$) of **1c**

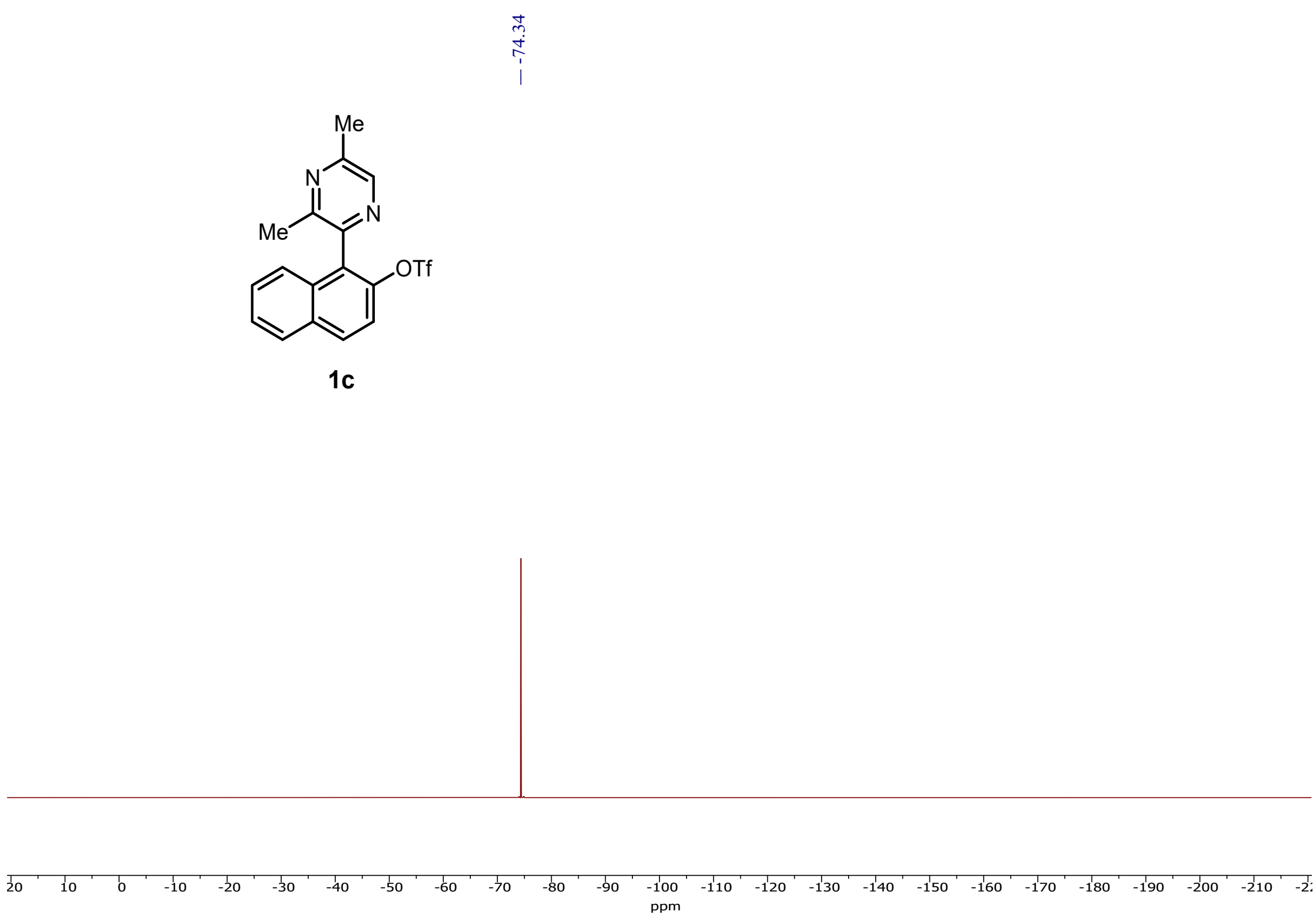

^{1}H NMR (400 MHz, $CDCl_3$) of **1d** (*see procedure*)

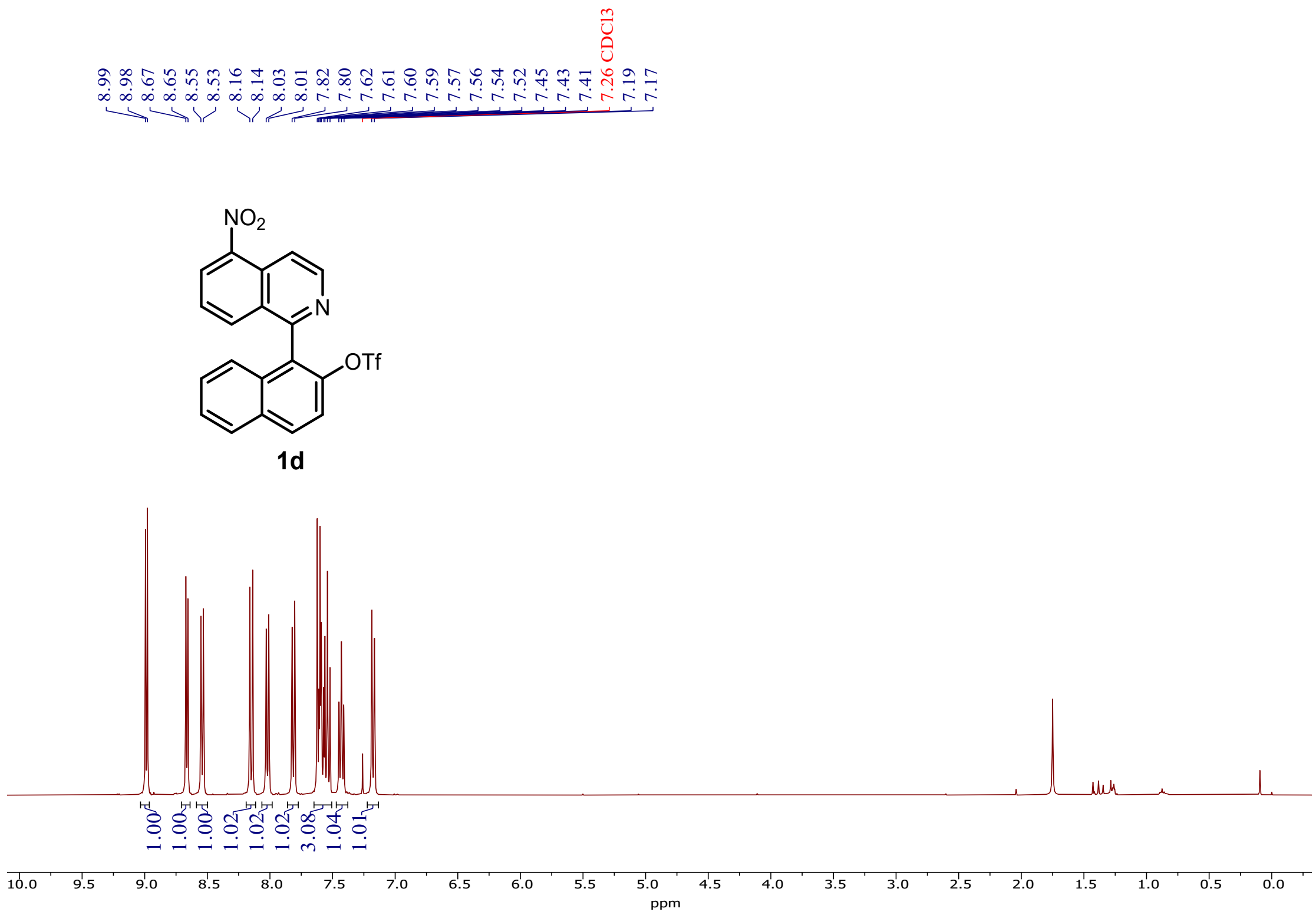

^{13}C NMR (100 MHz, $CDCl_3$) of **1d**

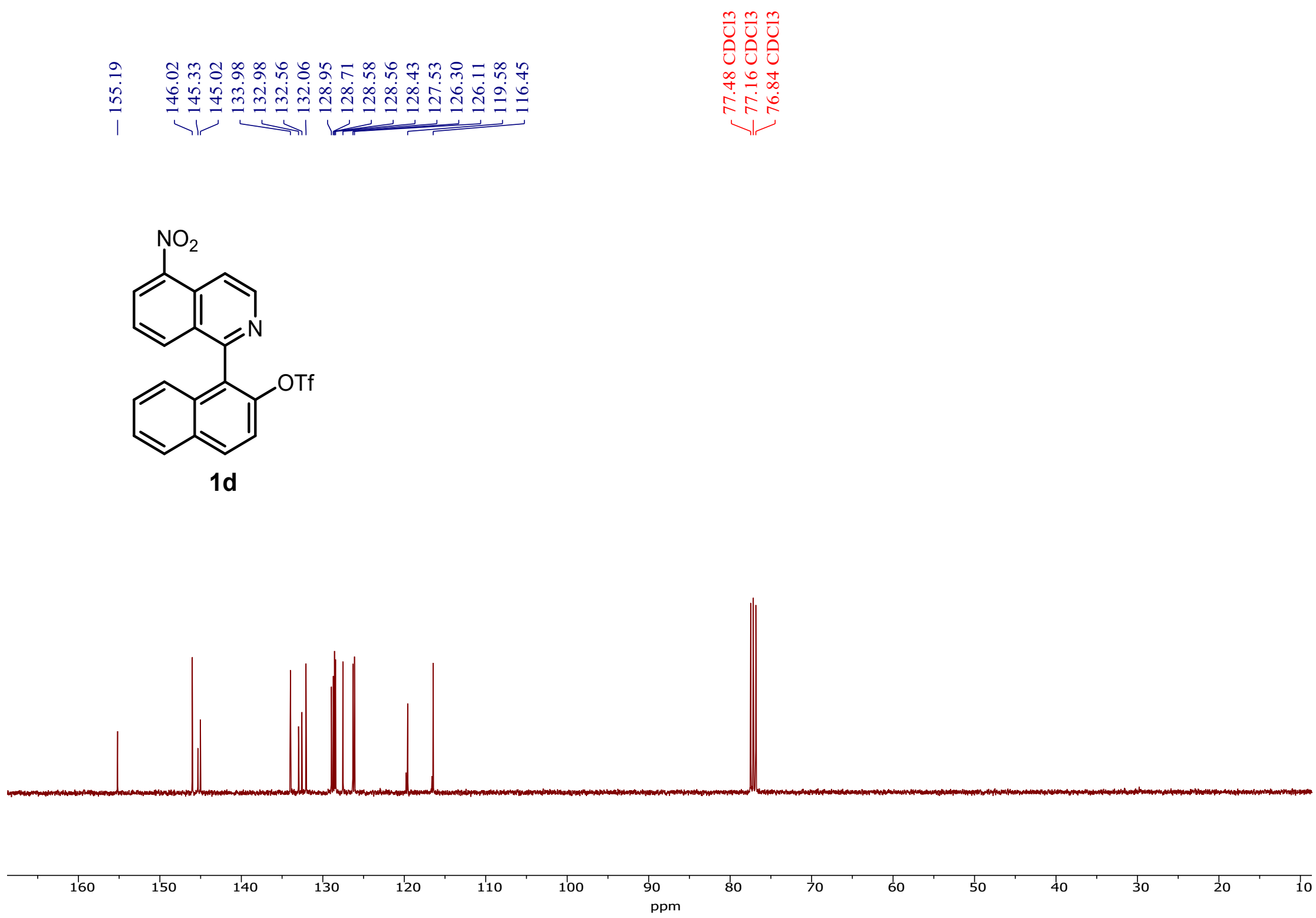

^{19}F NMR (376 MHz, $CDCl_3$) of **1d**

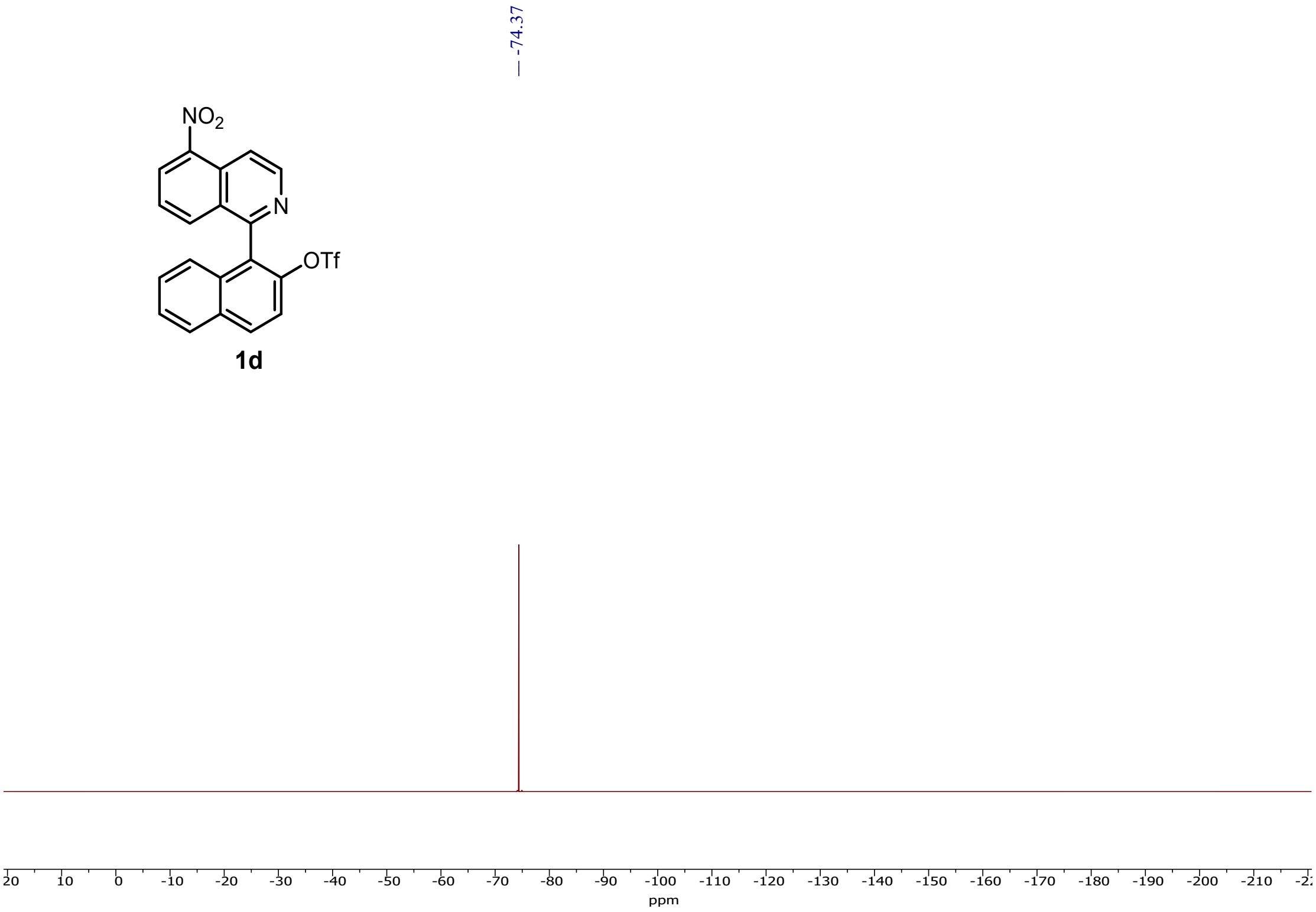

^{1}H NMR (400 MHz, $CDCl_3$) of **1e** (*see procedure*)

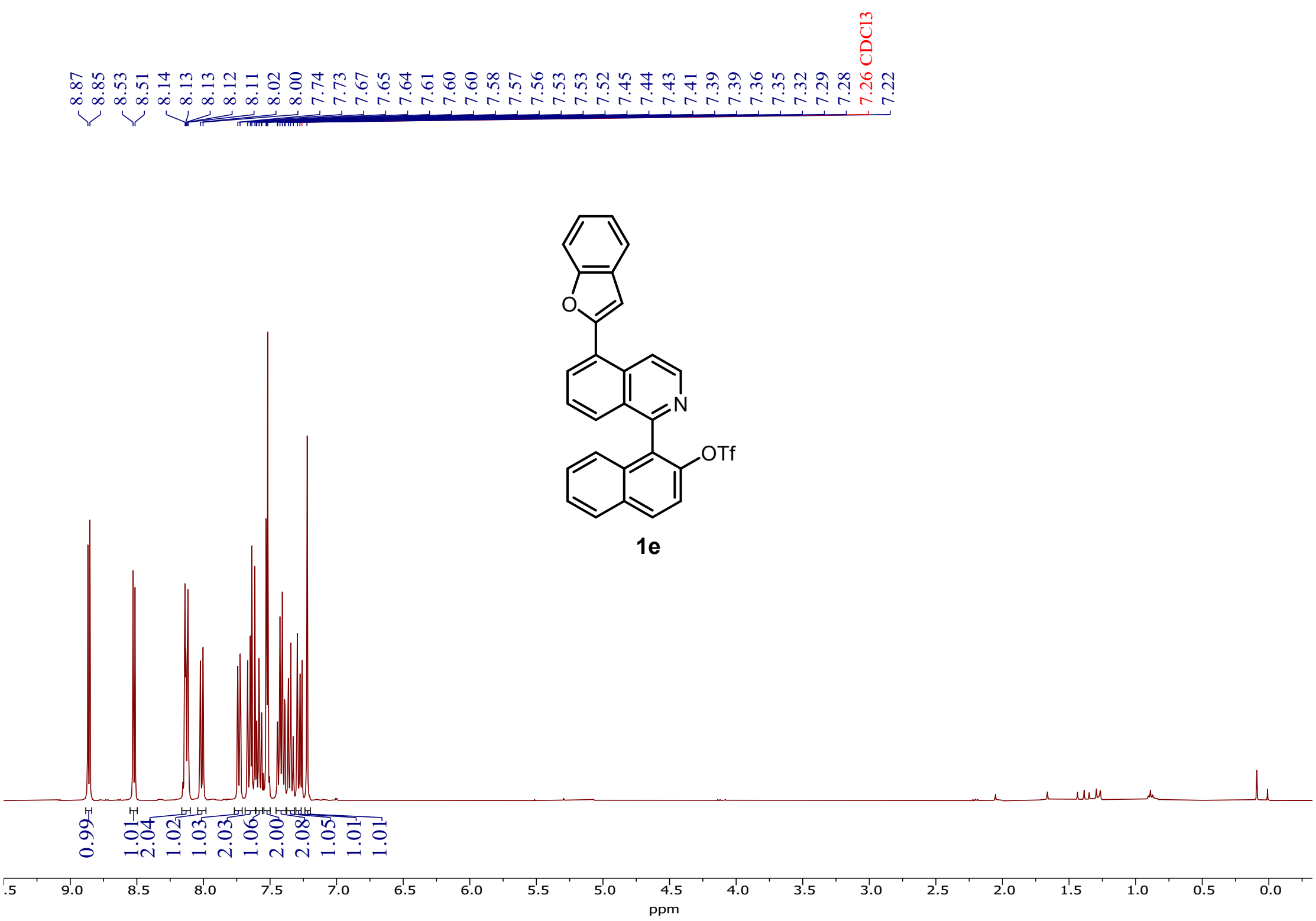

^{13}C NMR (100 MHz, $CDCl_3$) of **1e**

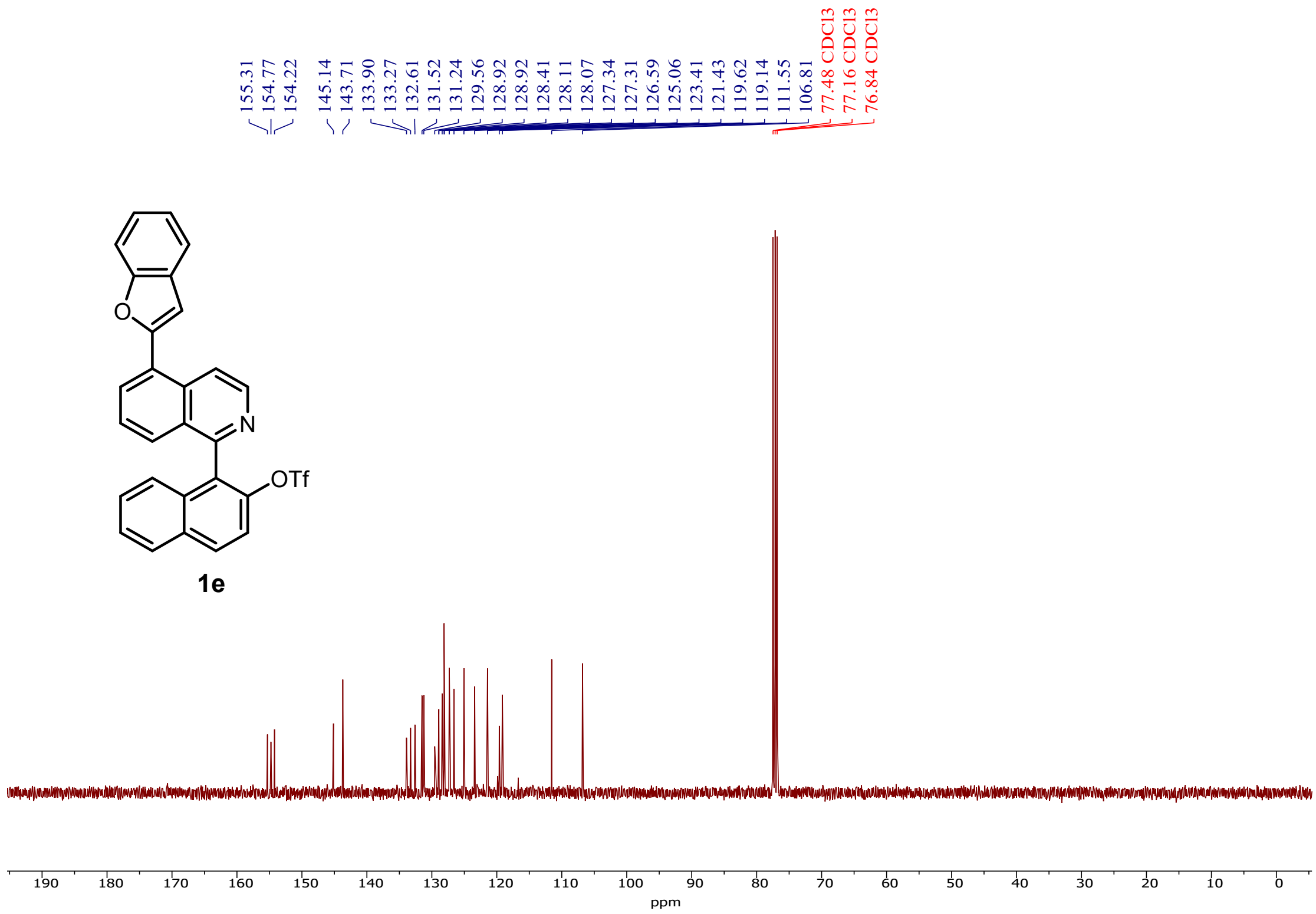

^{19}F NMR (376 MHz, $CDCl_3$) of **1e**

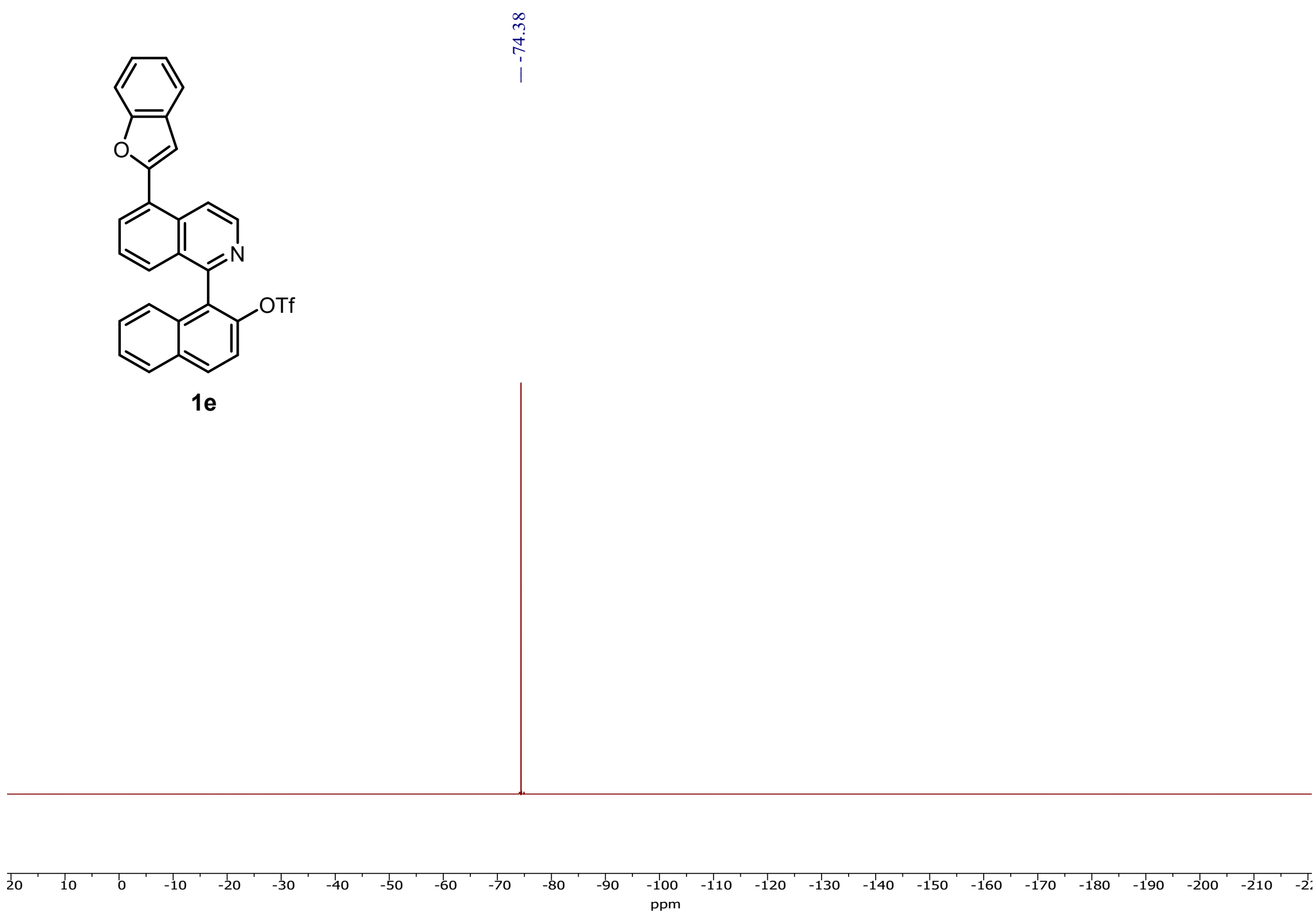

^1H NMR (400 MHz, $CDCl_3$) of **1f** (*see procedure*)

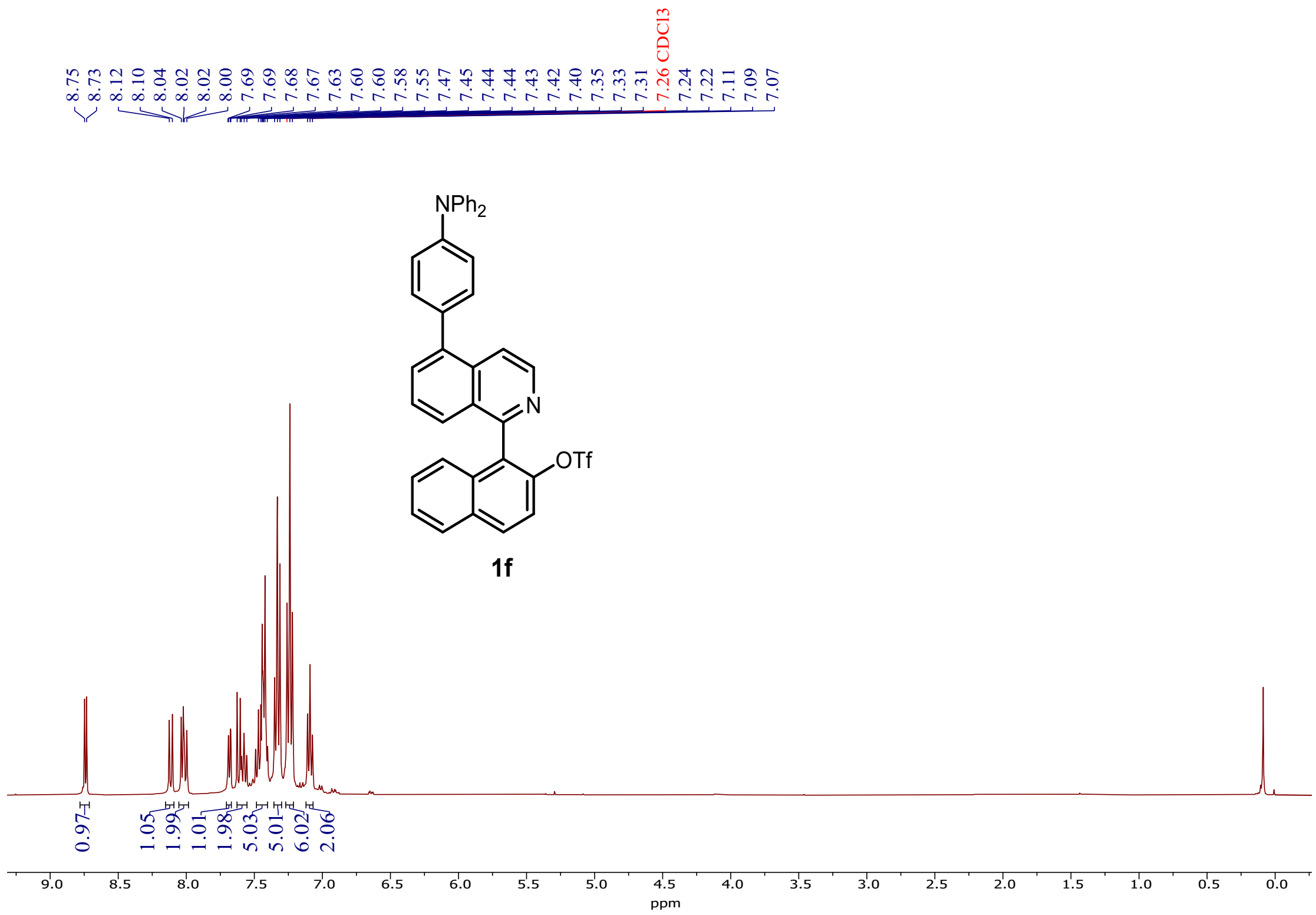

^{13}C NMR (100 MHz, $CDCl_3$) of **1f**

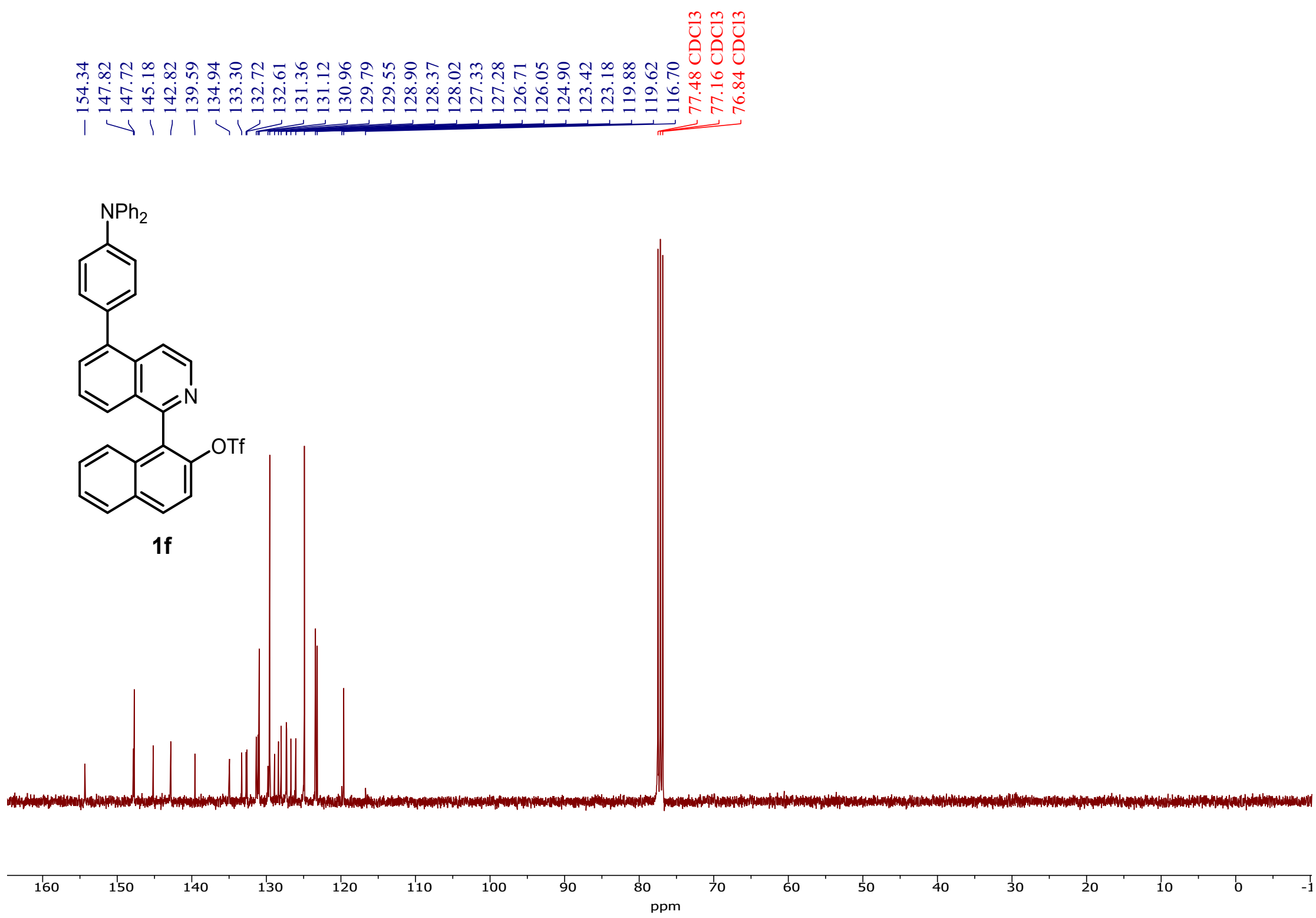

^{19}F NMR (376 MHz, $CDCl_3$) of **1f**

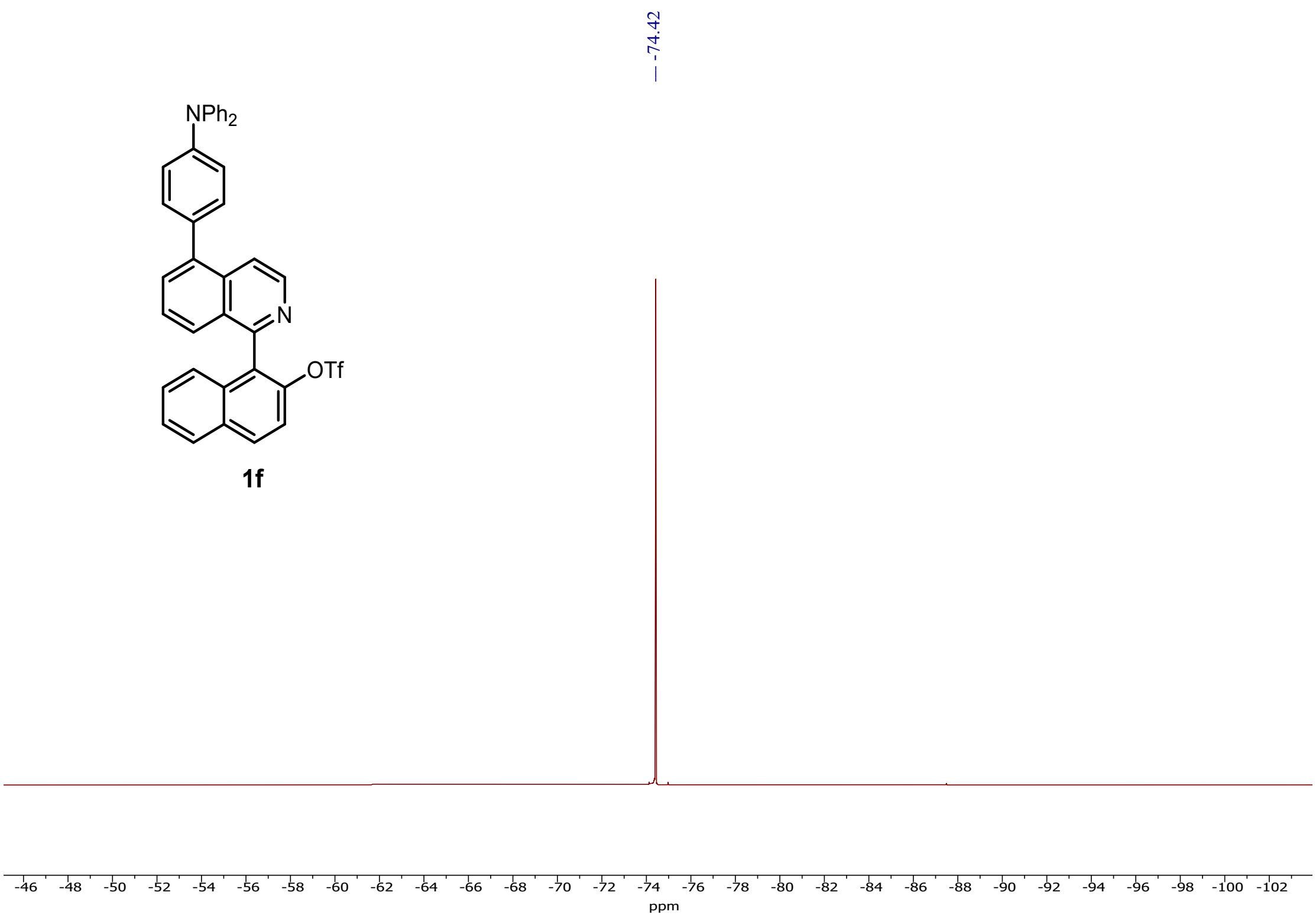

^{1}H NMR (400 MHz, $CDCl_3$) of **1g** (*see procedure*)

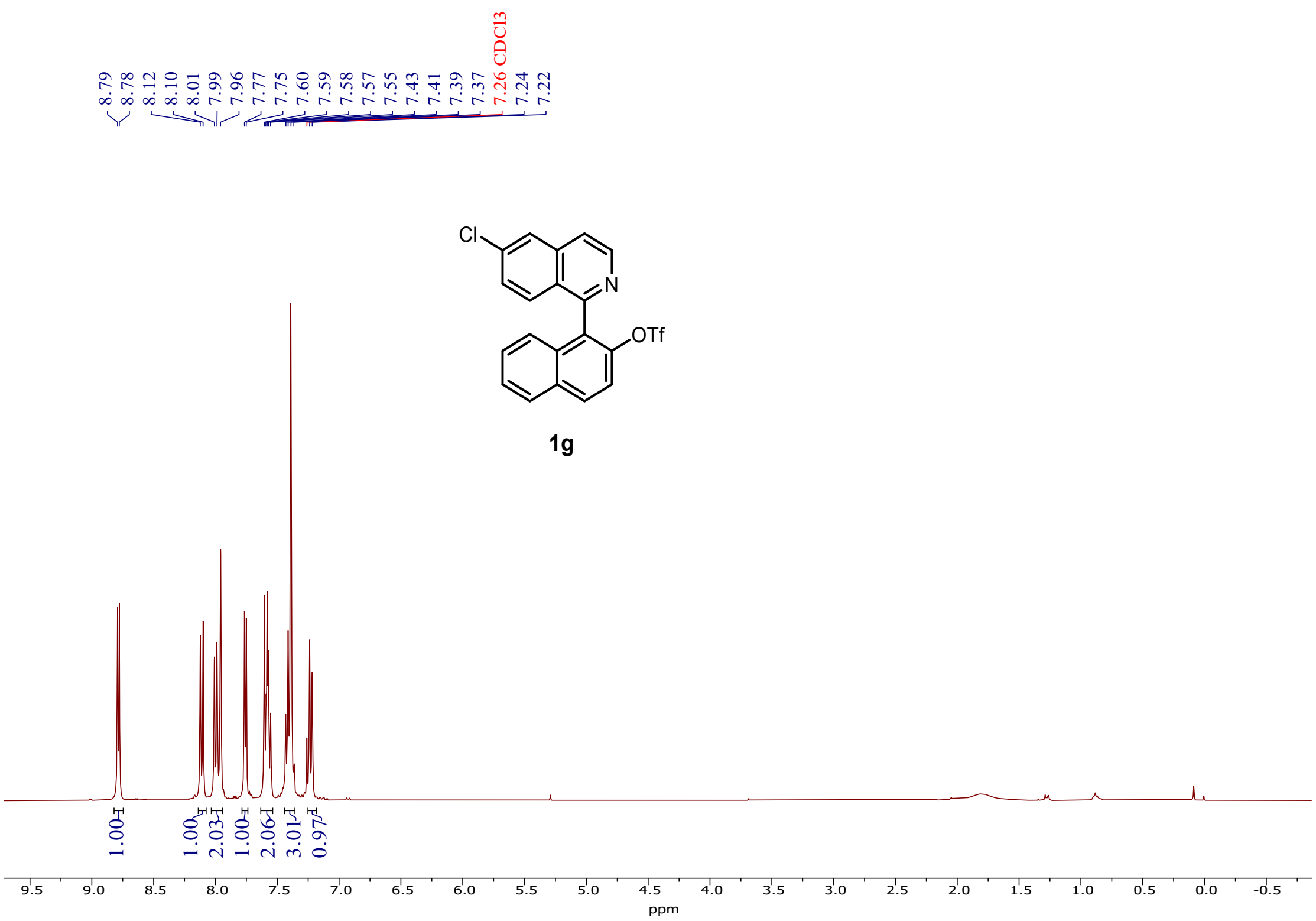

^{13}C NMR (125 MHz, $CDCl_3$) of **1g**

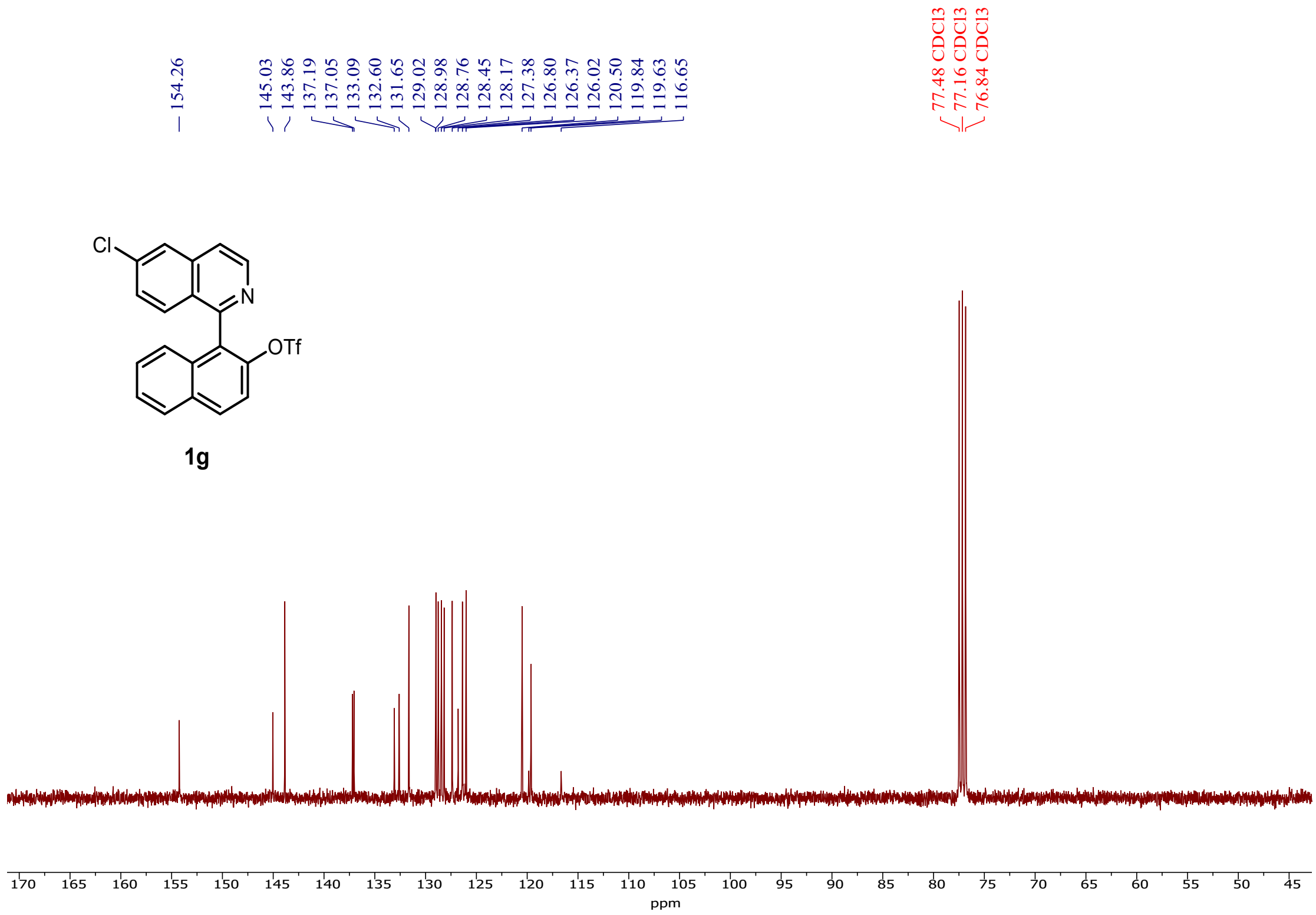

^{19}F NMR (376 MHz, $CDCl_3$) of **1g**

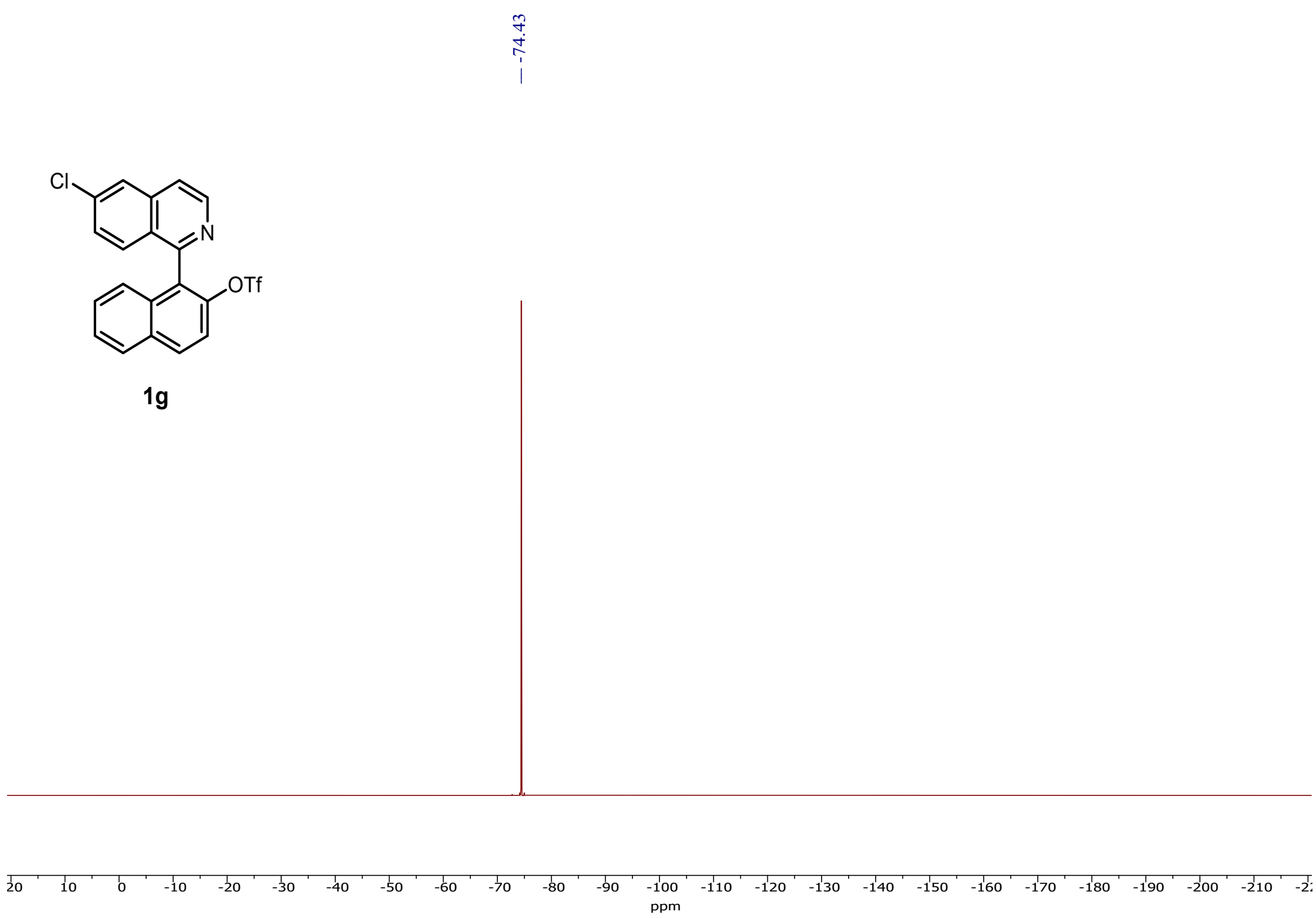

^{1}H NMR (400 MHz, $CDCl_3$) of **1h** (*see procedure*)

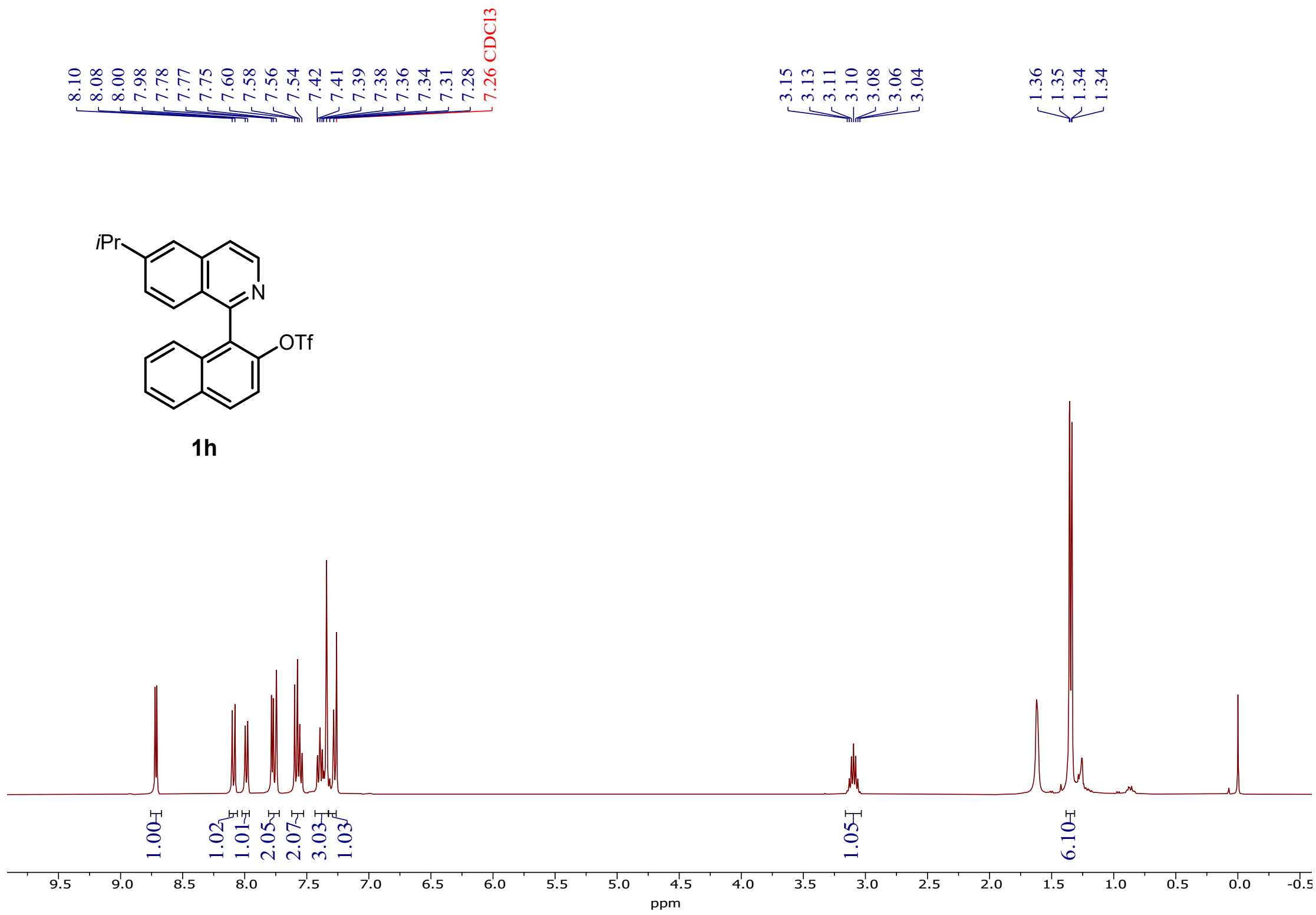

^{13}C NMR (125 MHz, $CDCl_3$) of **1h**

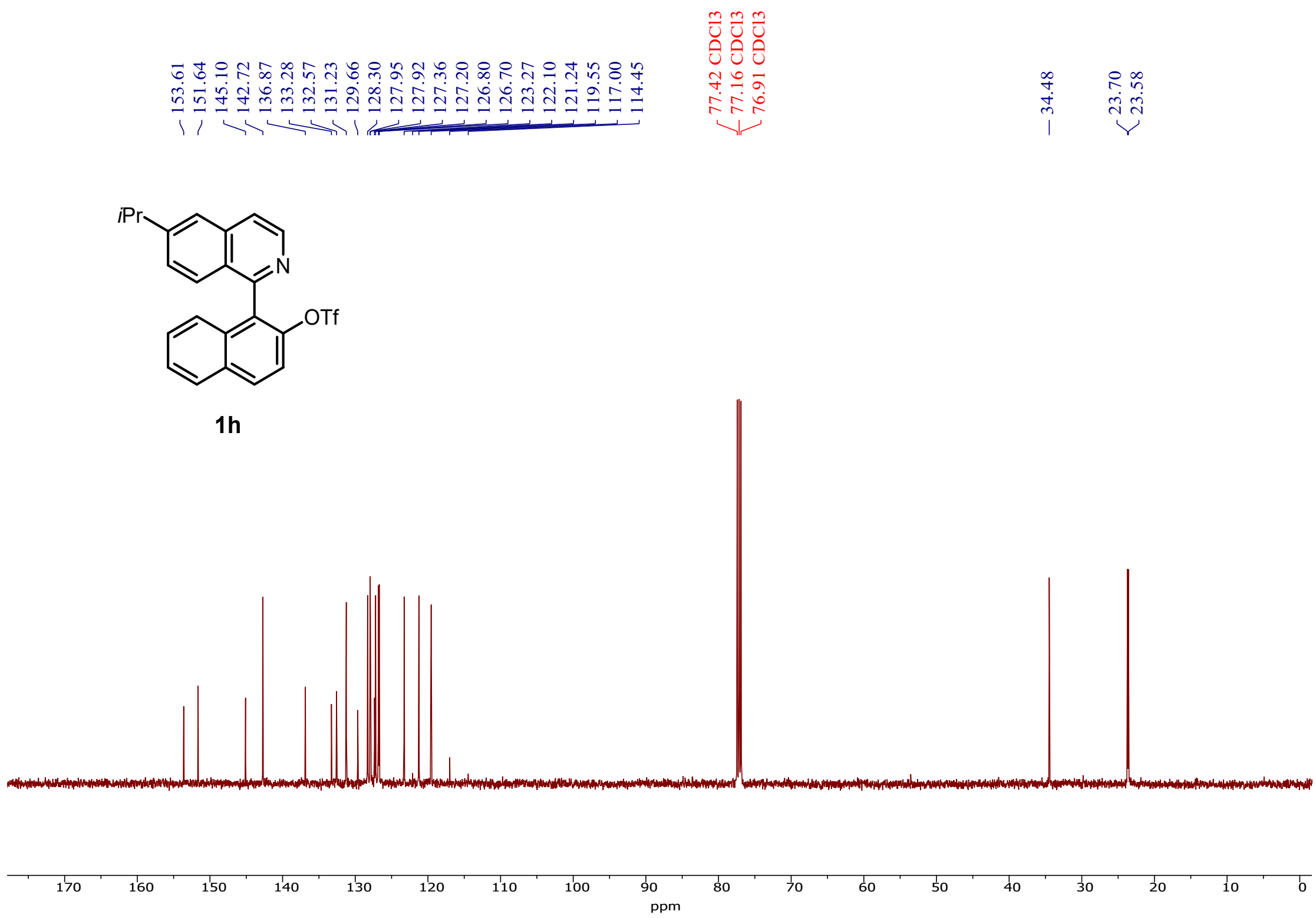

^{19}F NMR (376 MHz, $CDCl_3$) of **1h**

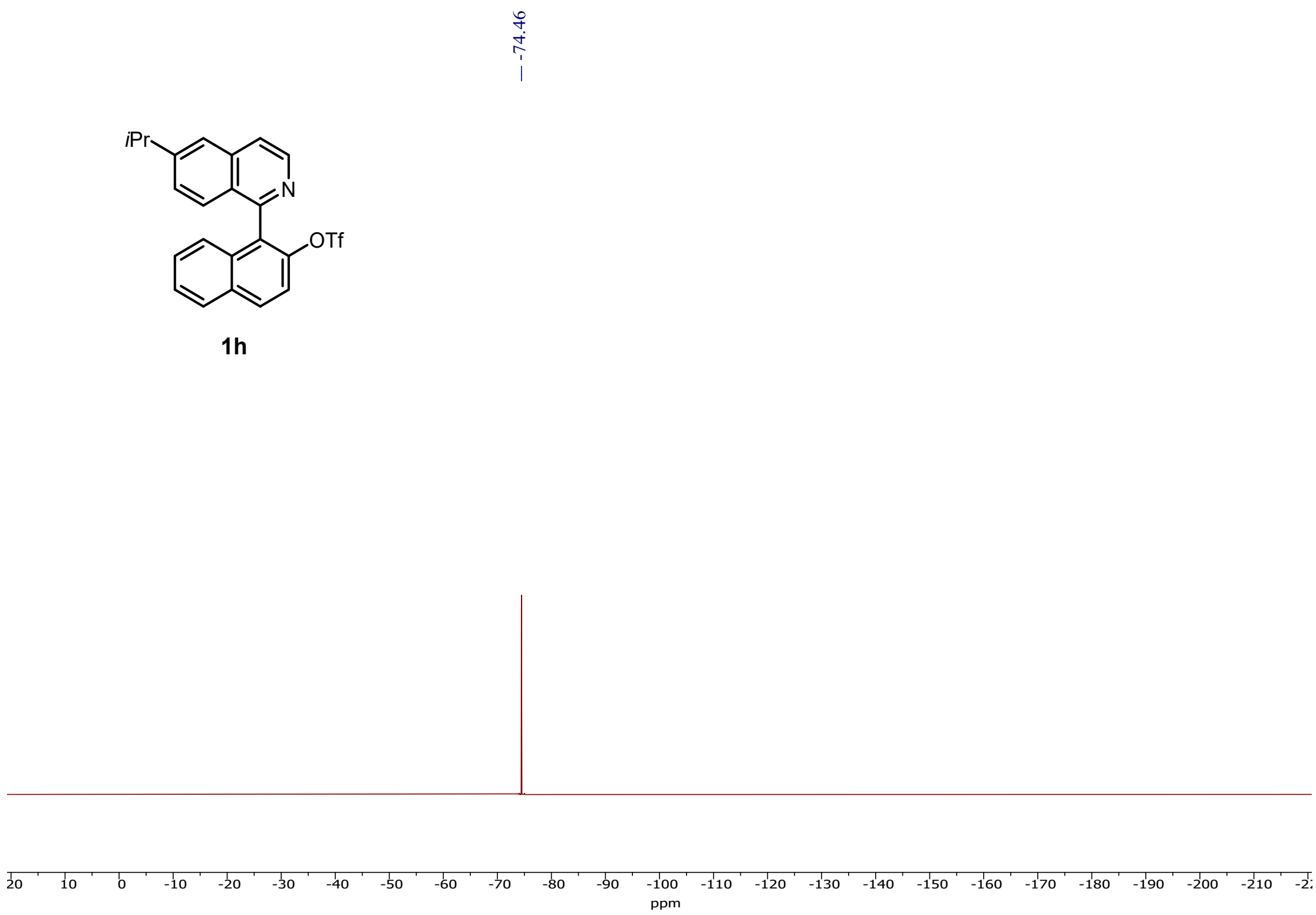

^{1}H NMR (400 MHz, $CDCl_3$) of **1i** (*see procedure*)

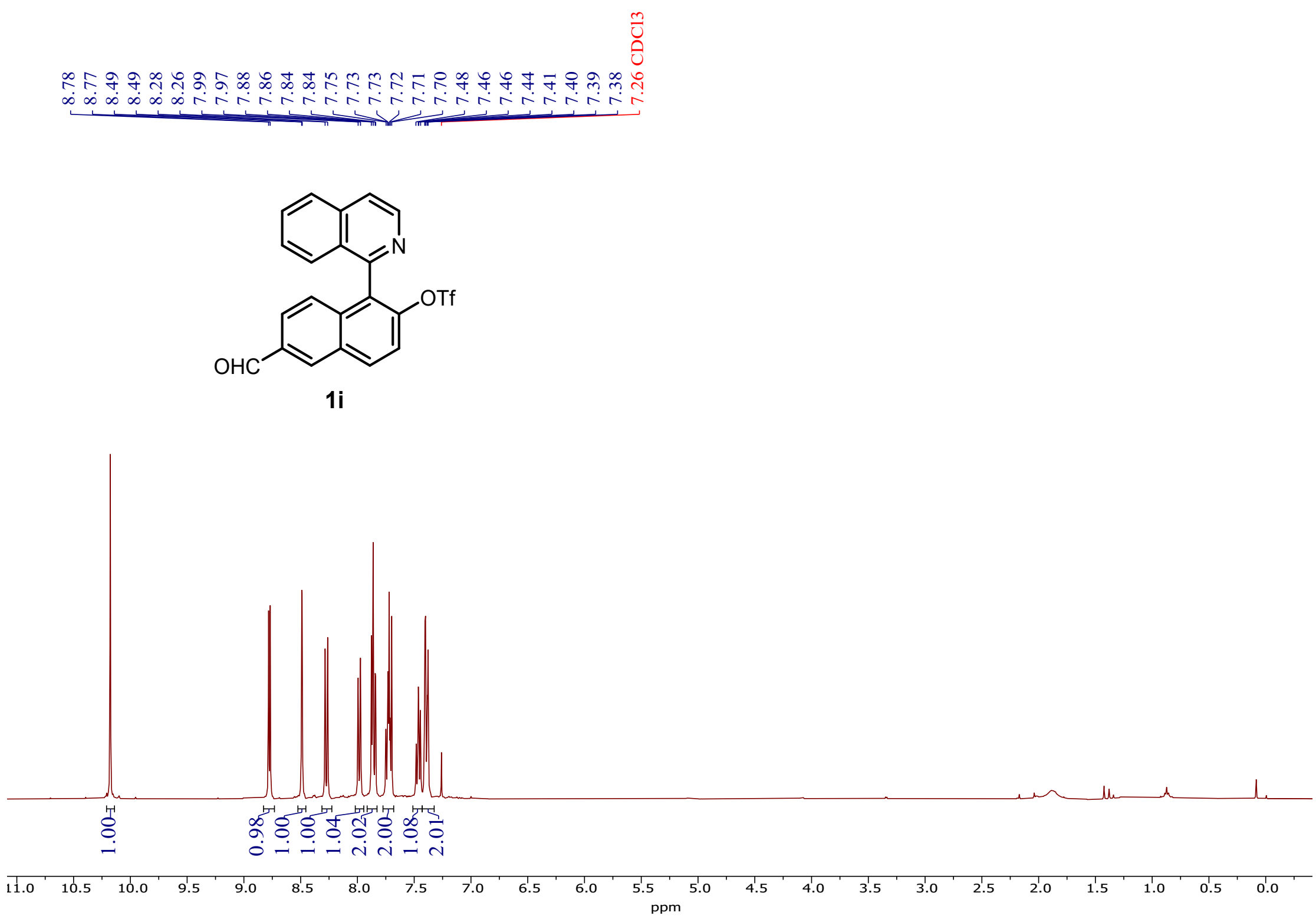

^{13}C NMR (125 MHz, $CDCl_3$) of **1i**

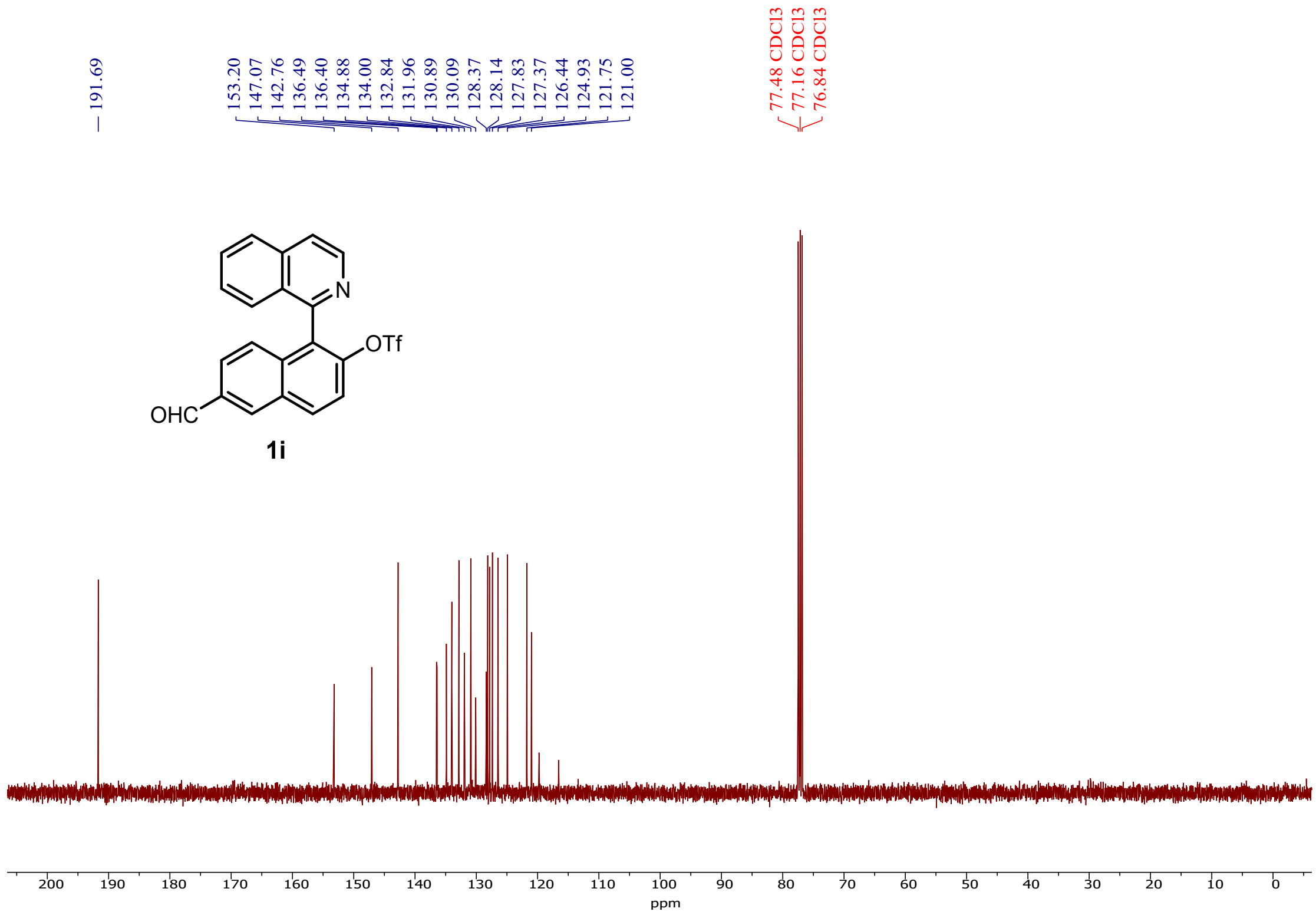

^{19}F NMR (376 MHz, $CDCl_3$) of **1i**

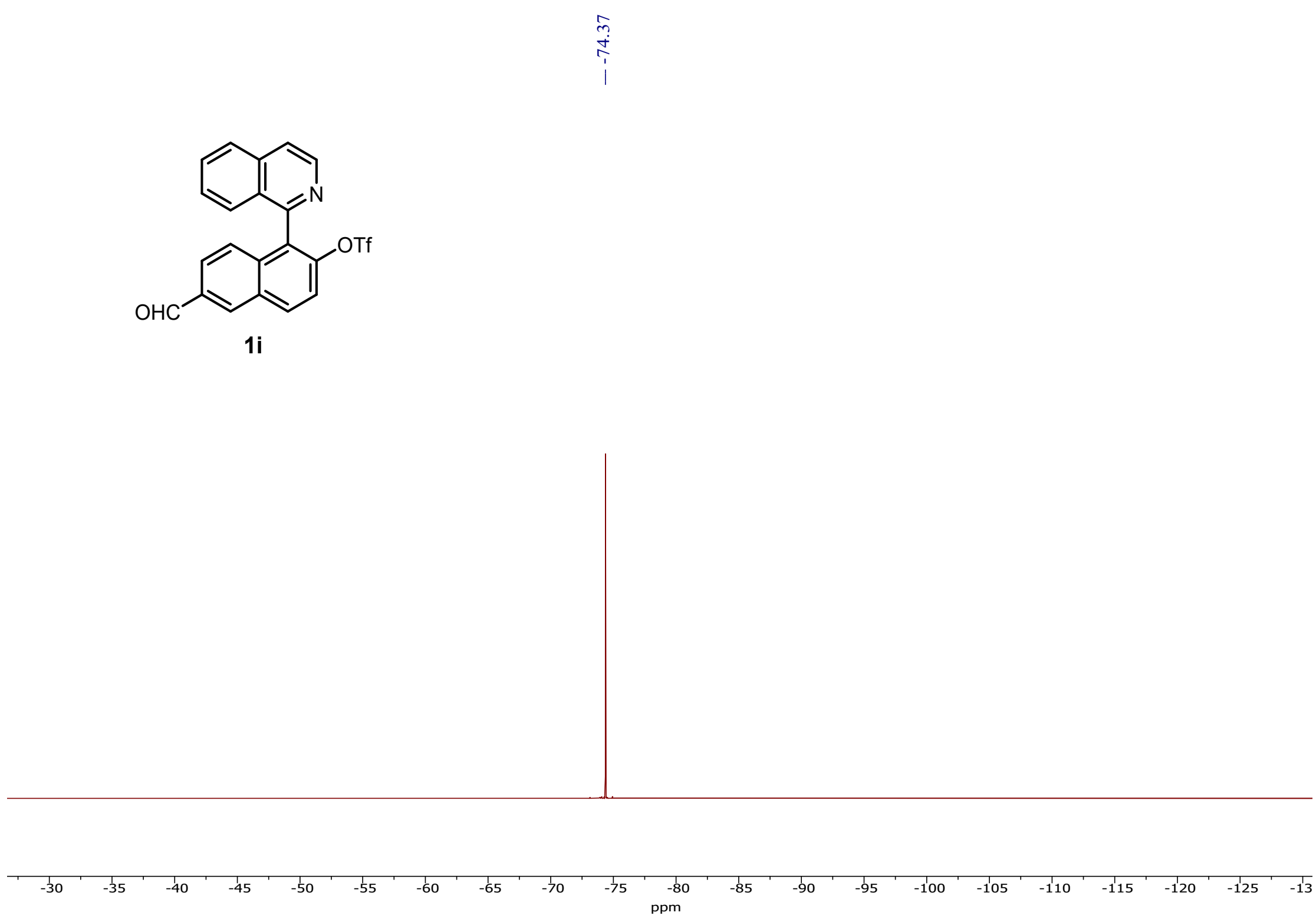

^{1}H NMR (400 MHz, $CDCl_3$) of **1j** (*see procedure*)

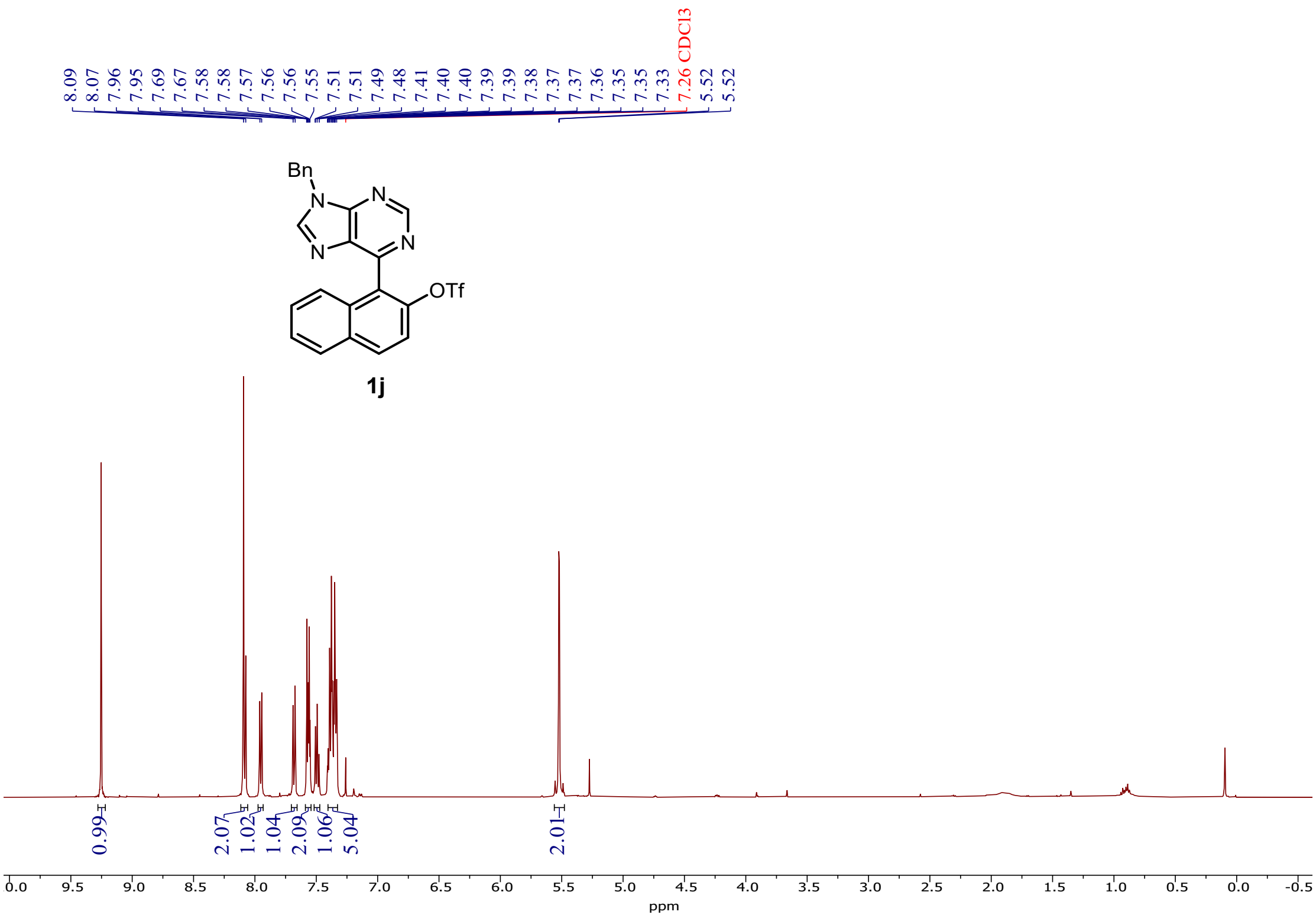

^{13}C NMR (125 MHz, $CDCl_3$) of **1j**

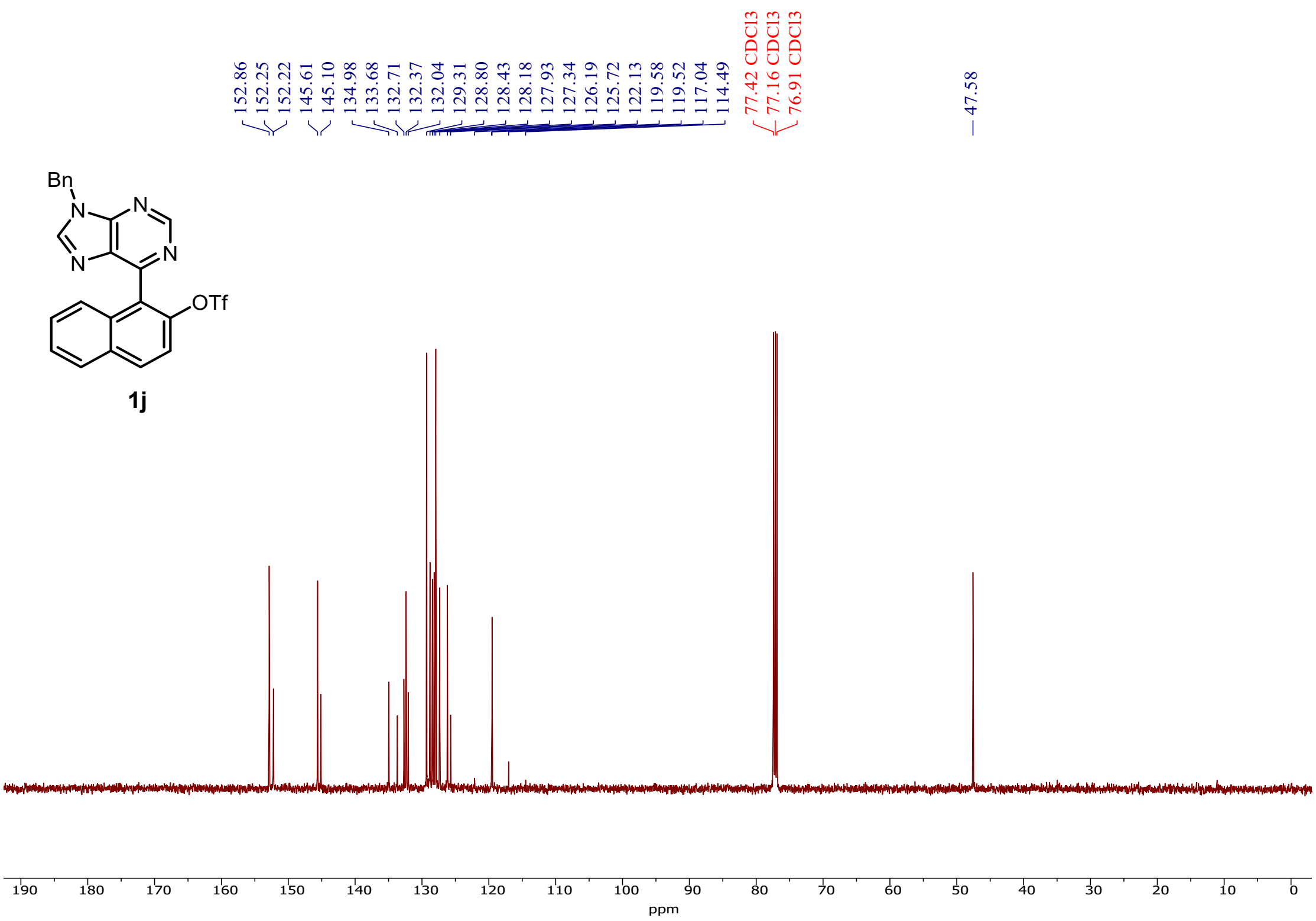

^{19}F NMR (376 MHz, $CDCl_3$) of **1j**

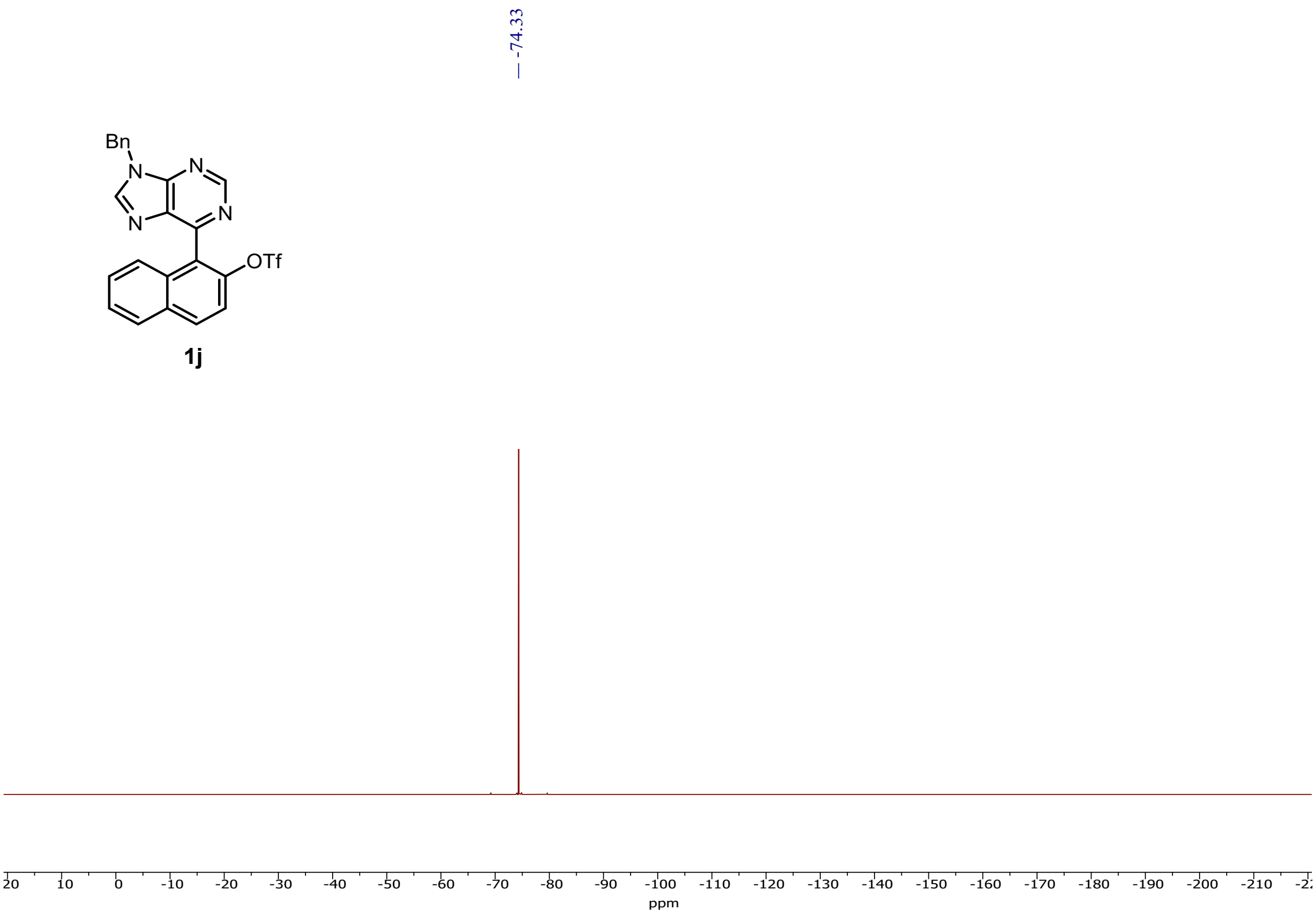

^{1}H NMR (400 MHz, d_6-DMSO) of **2c** (*see procedure*)

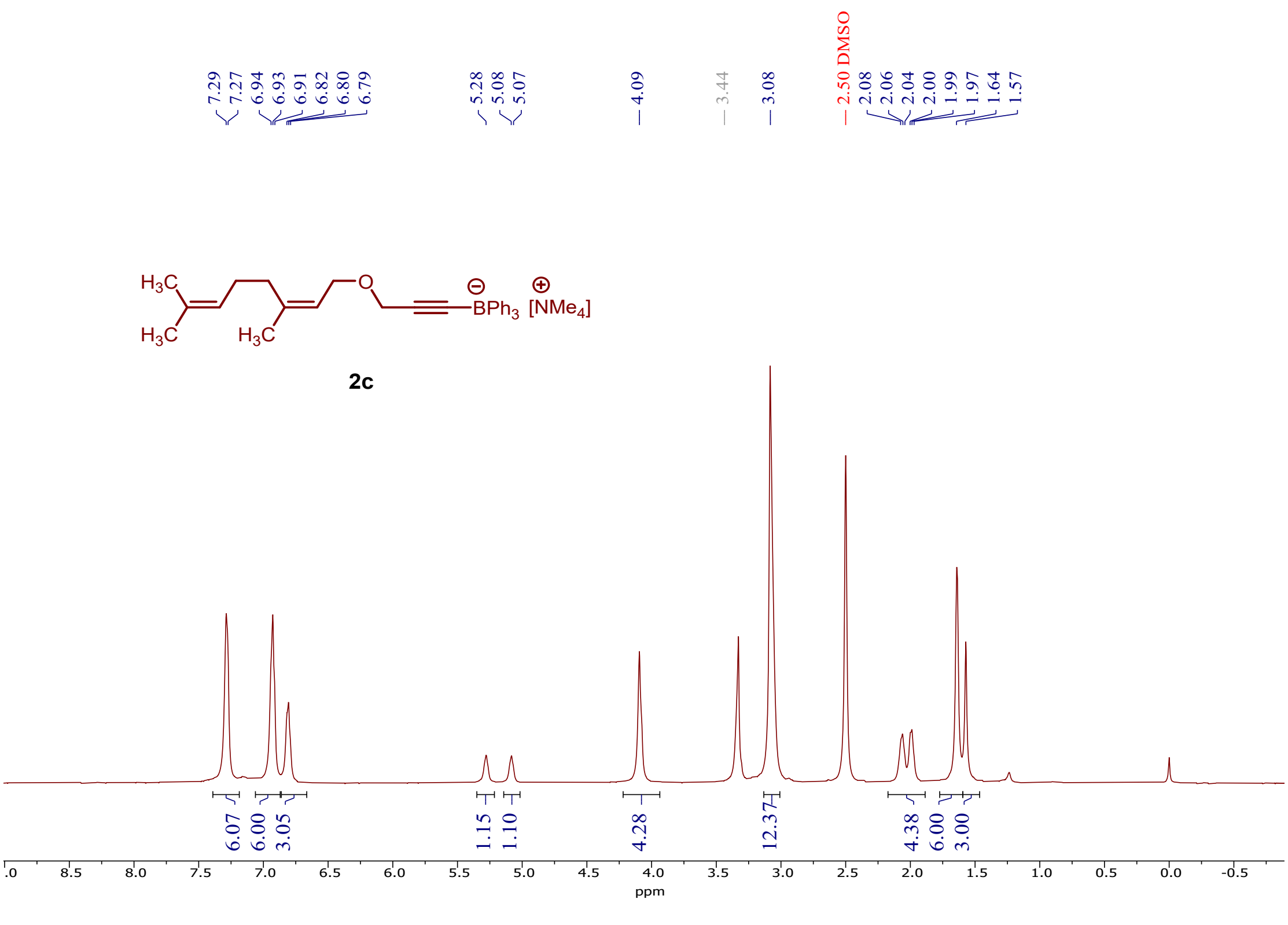

^{13}C NMR (125 MHz, d_6-DMSO) of **2c**

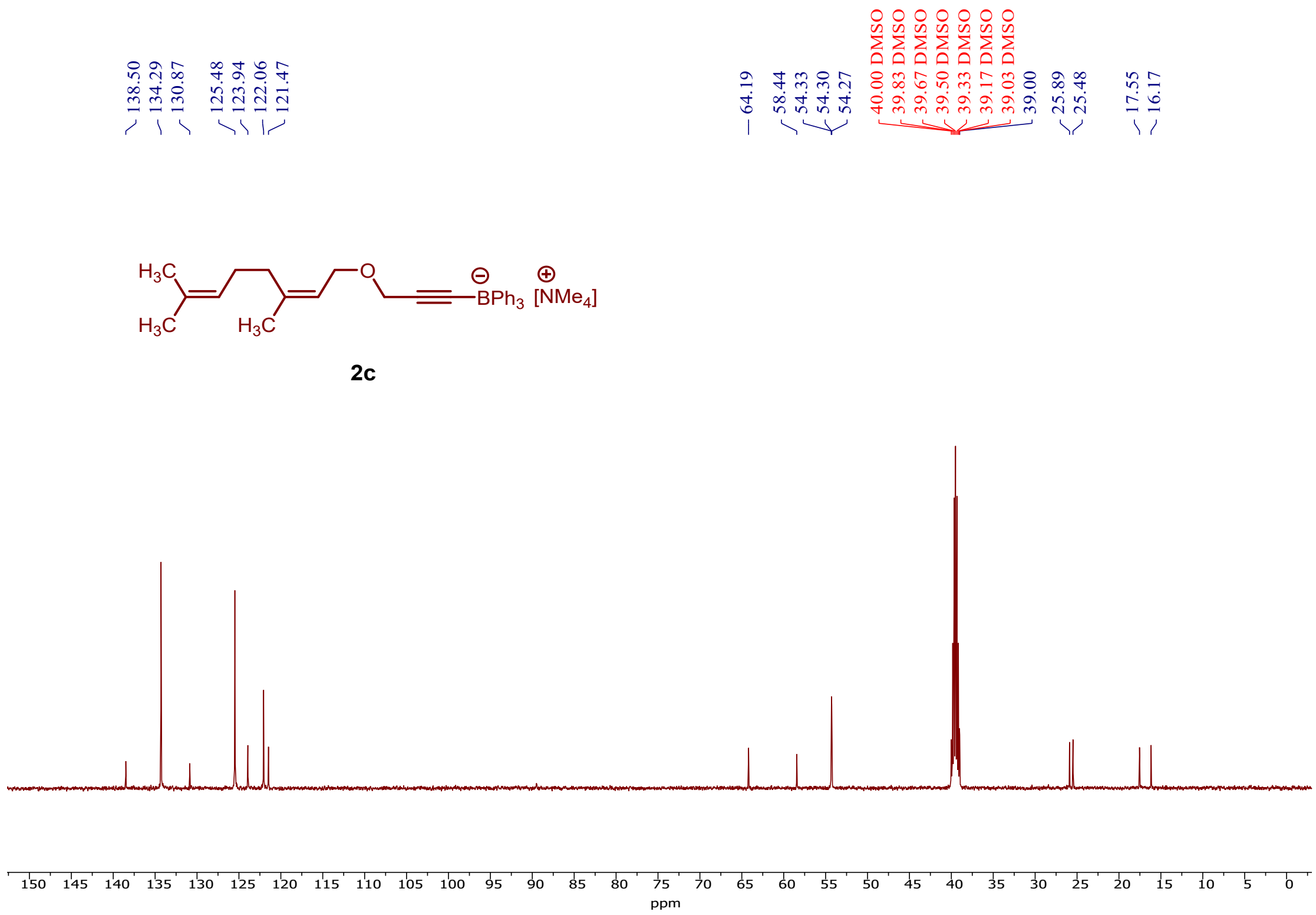

^{11}B NMR (160 MHz, d_6-DMSO) of **2c**

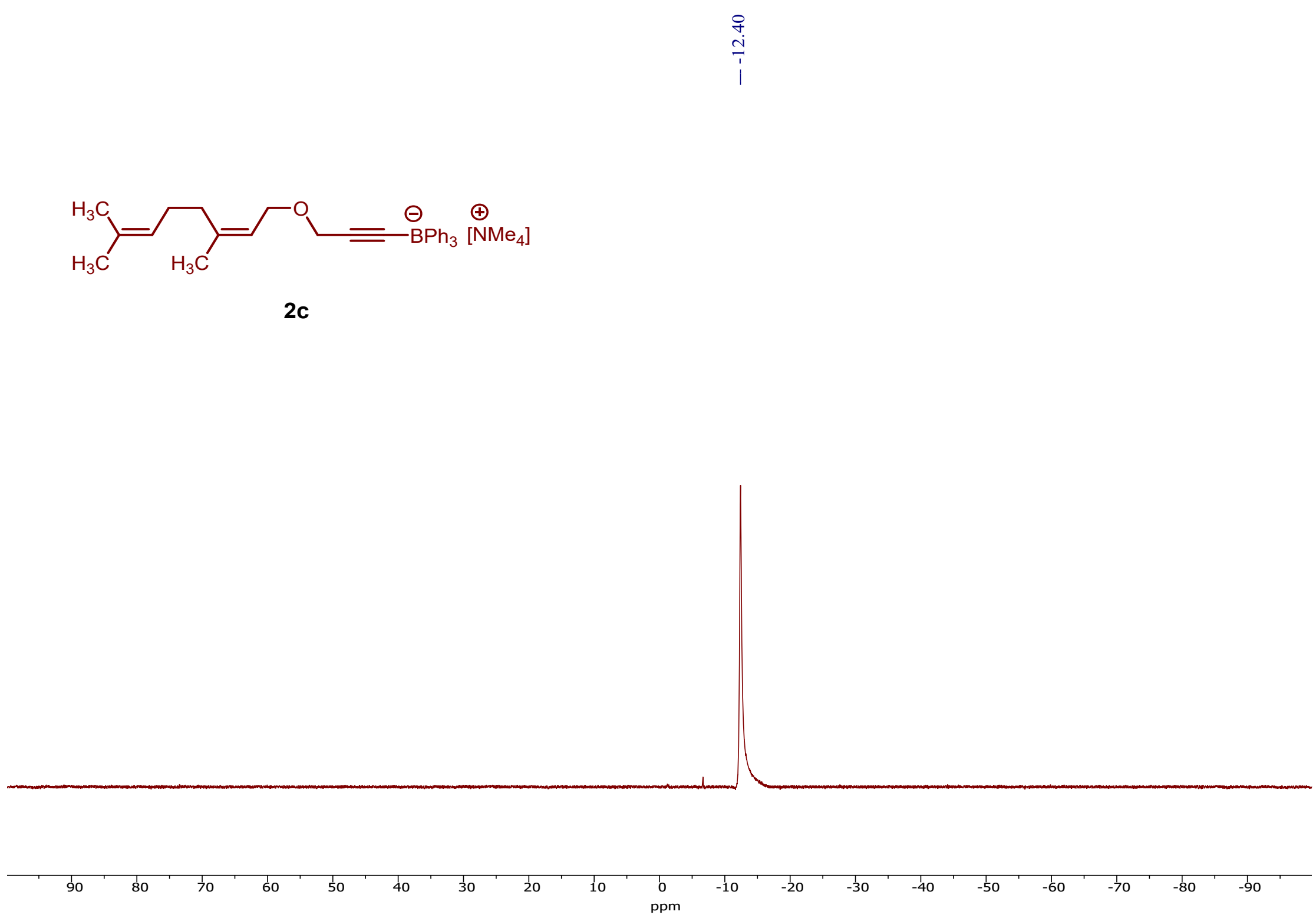

^{1}H NMR (400 MHz, $CDCl_3$) of **3** (*see procedure*)

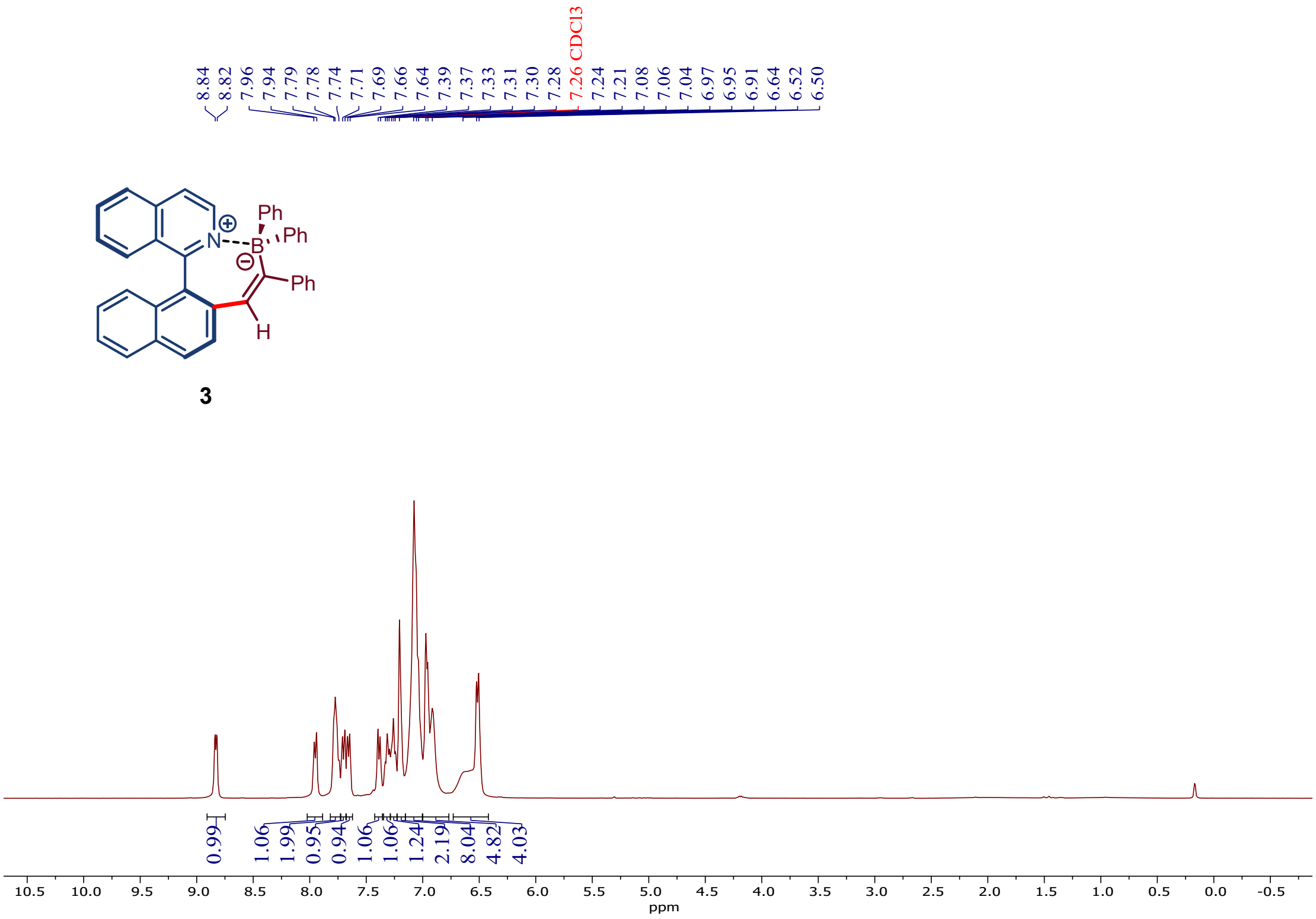

^{13}C NMR (100 MHz, $CDCl_3$) of **3**

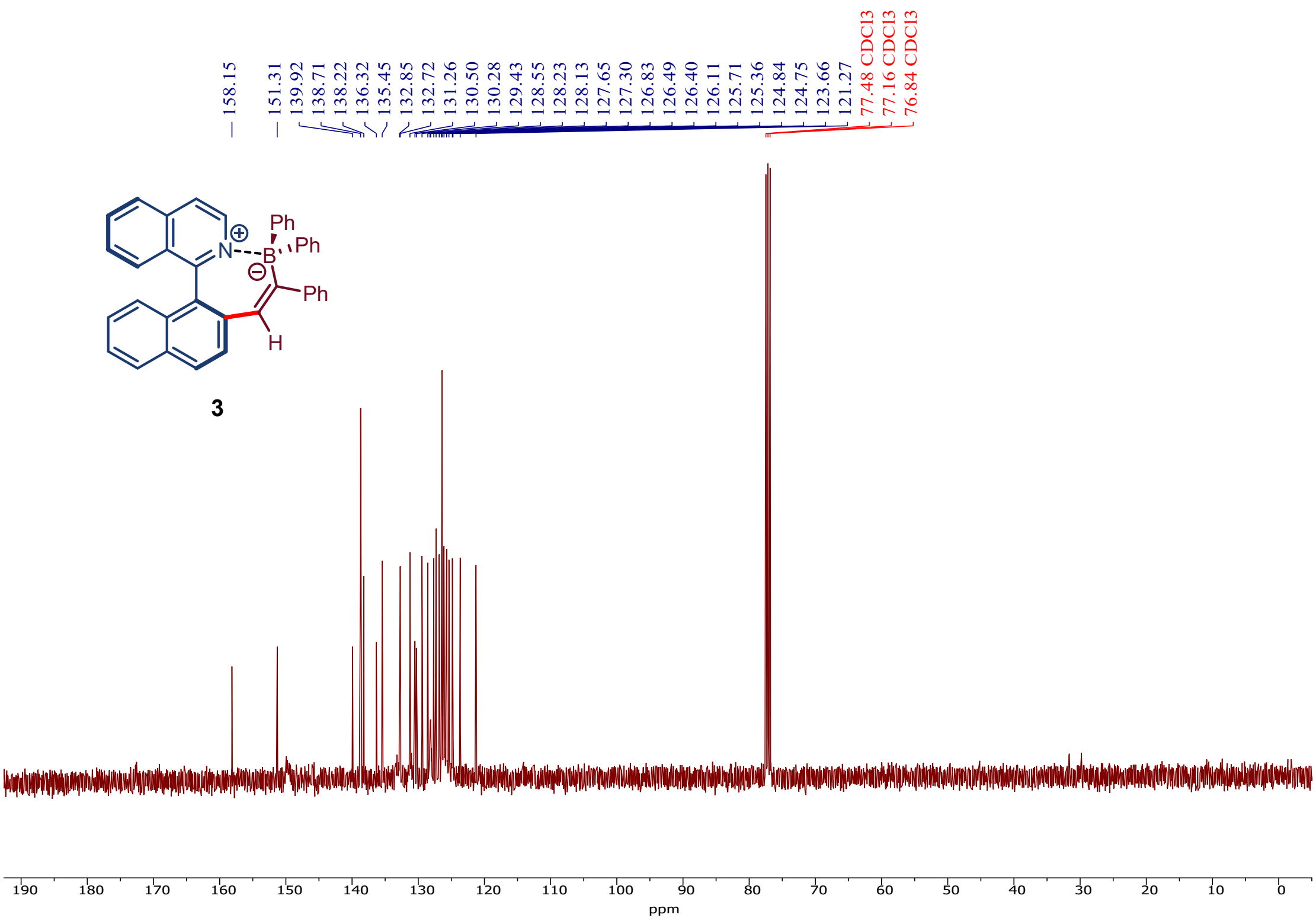

^{11}B NMR (128 MHz, CDCl$_3$) of **3**

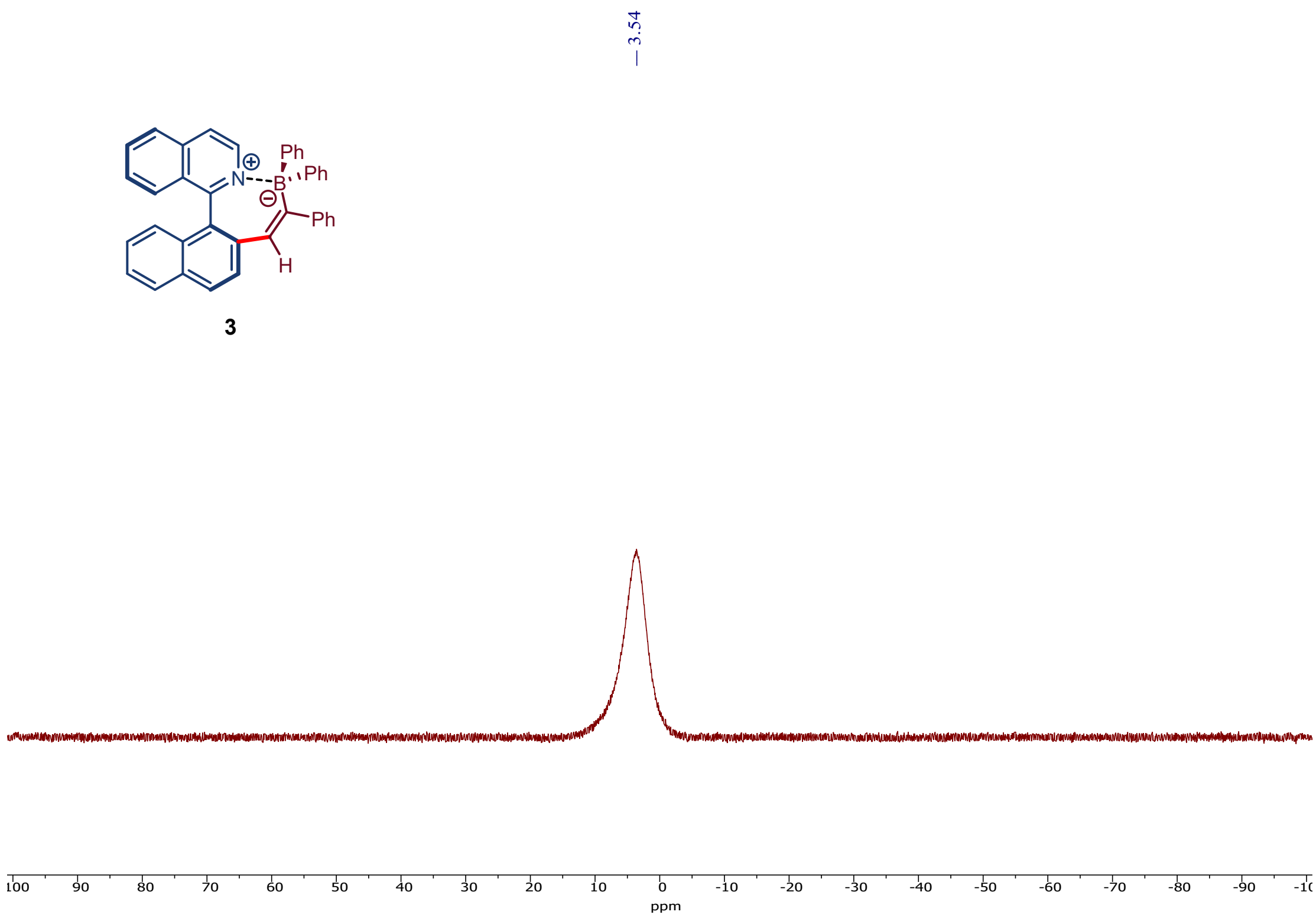

^{1}H NMR (400 MHz, CDCl$_3$) of **4** (*see procedure*)

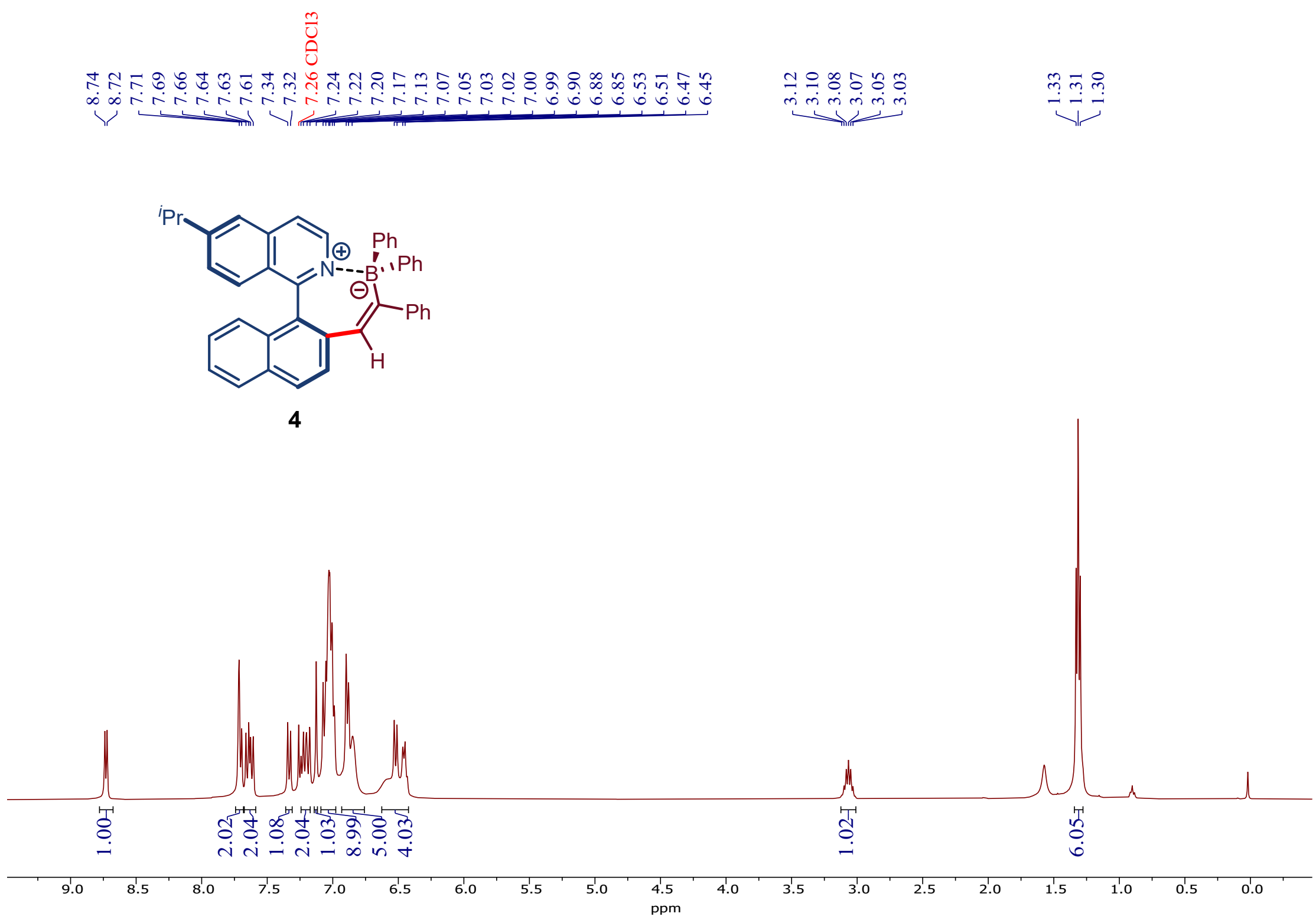

^{13}C NMR (100 MHz, $CDCl_3$) of **4**

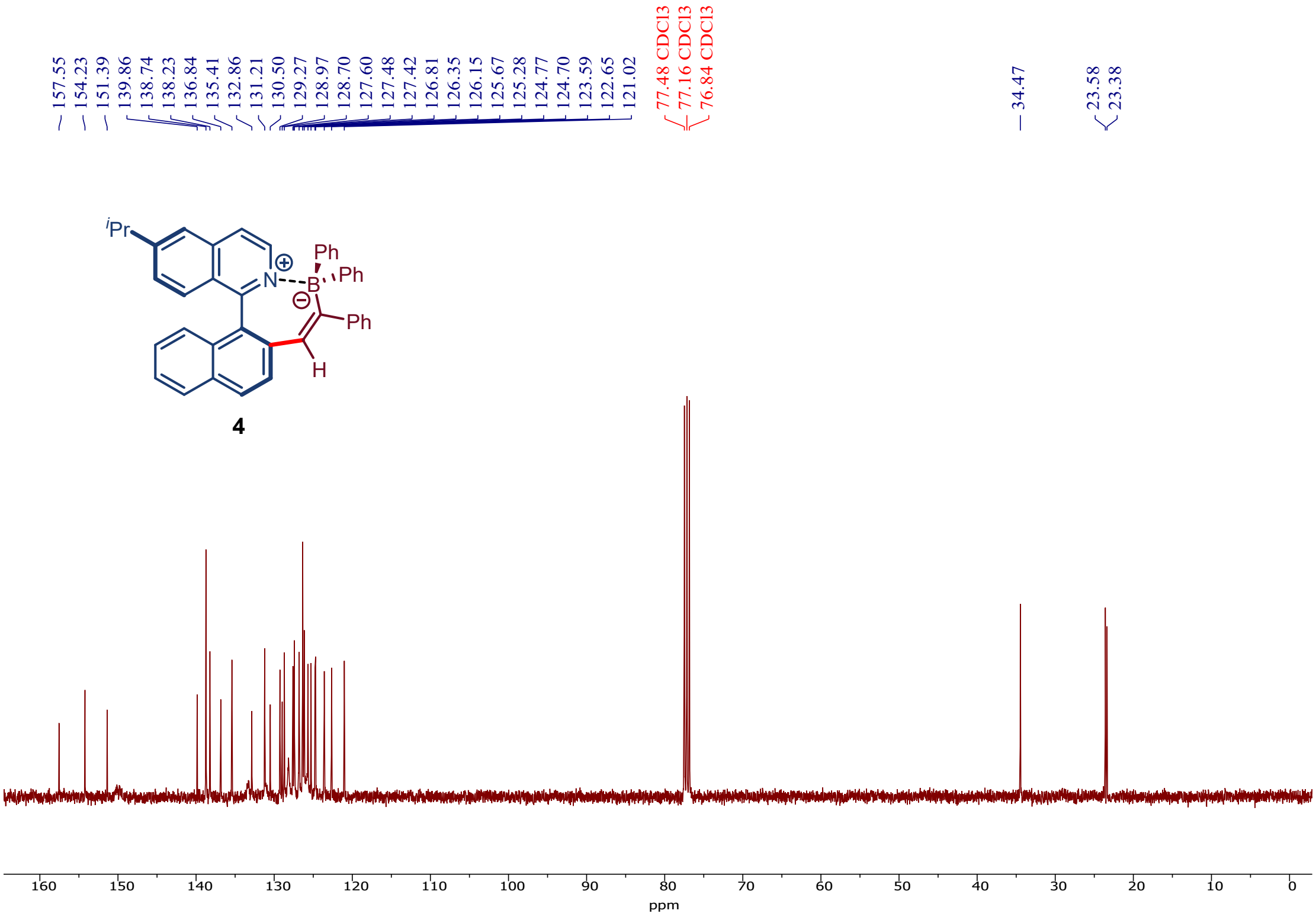

^{11}B NMR (128 MHz, $CDCl_3$) of **4**

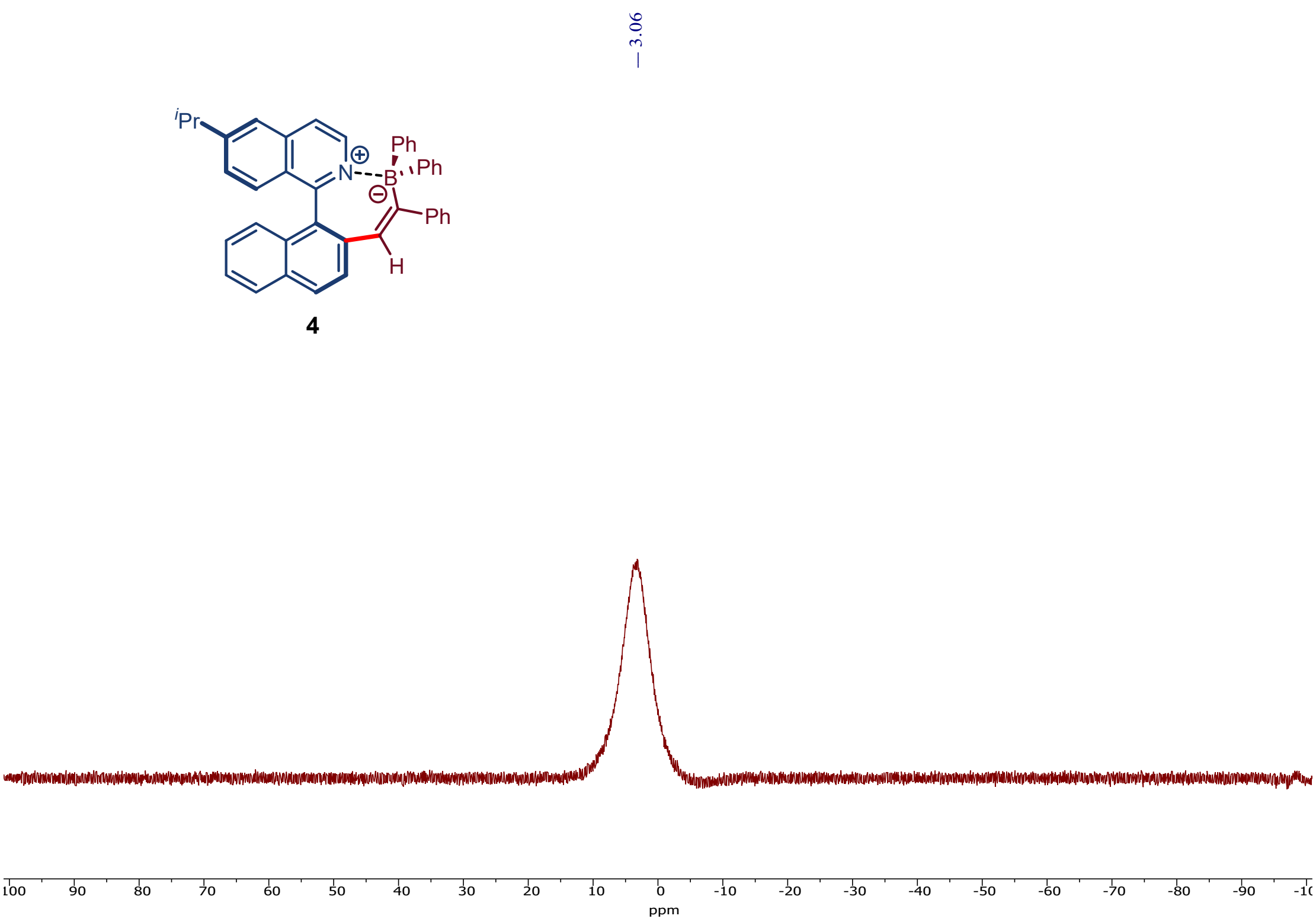

^{1}H NMR (400 MHz, $CDCl_3$) of **5** (*see procedure*)

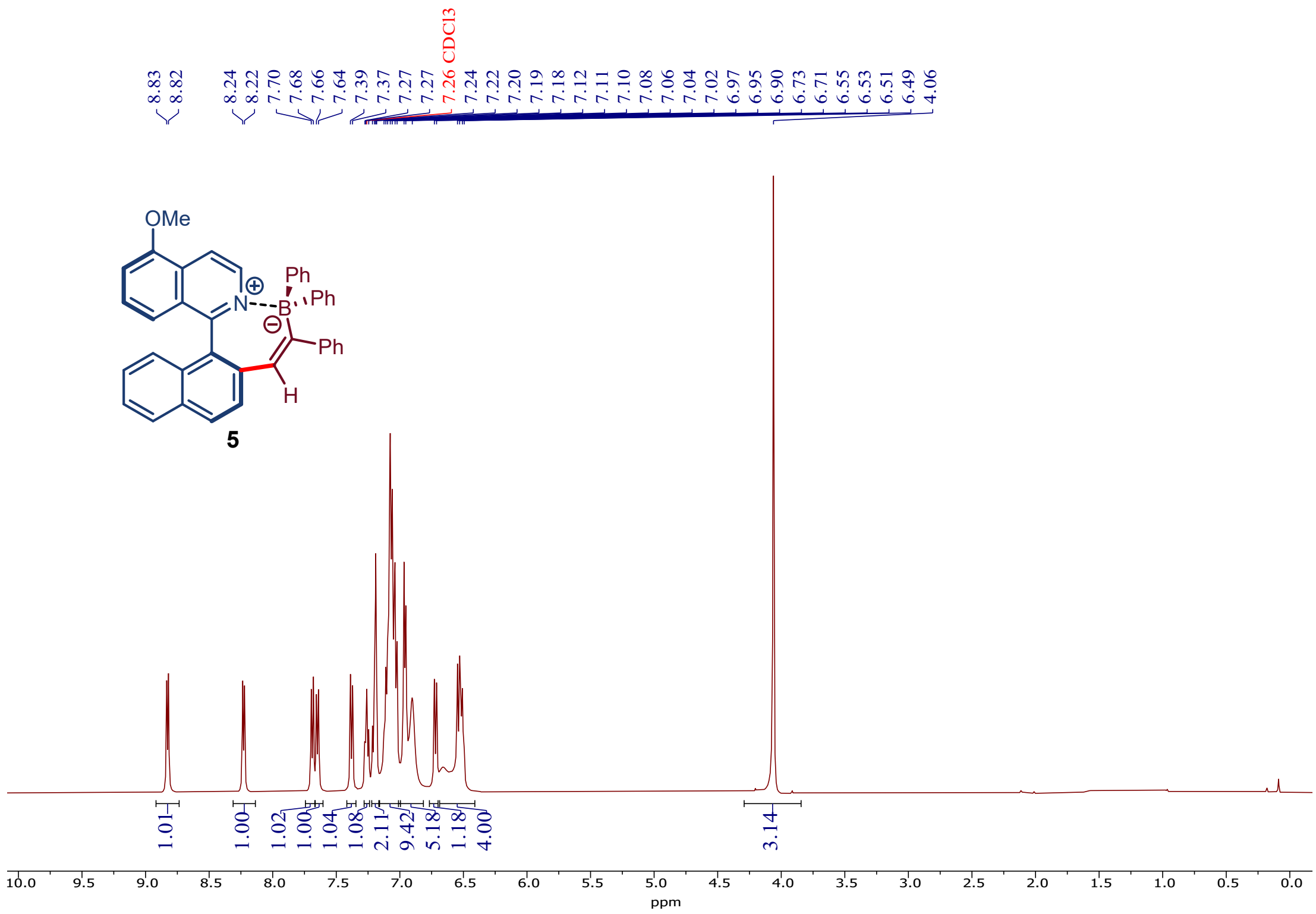

^{13}C NMR (100 MHz, $CDCl_3$) of **5**

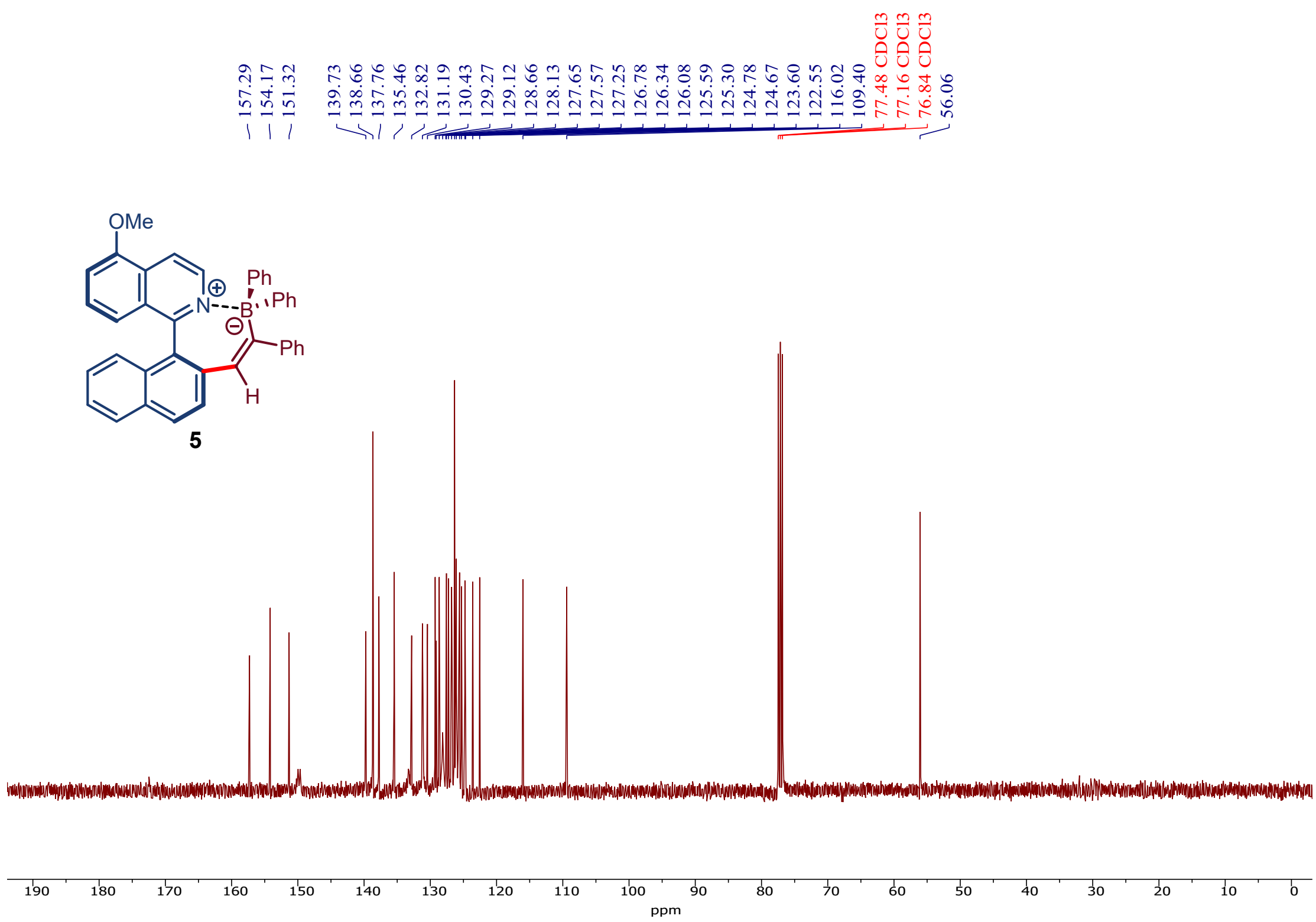

^{11}B NMR (128 MHz, $CDCl_3$) of **5**

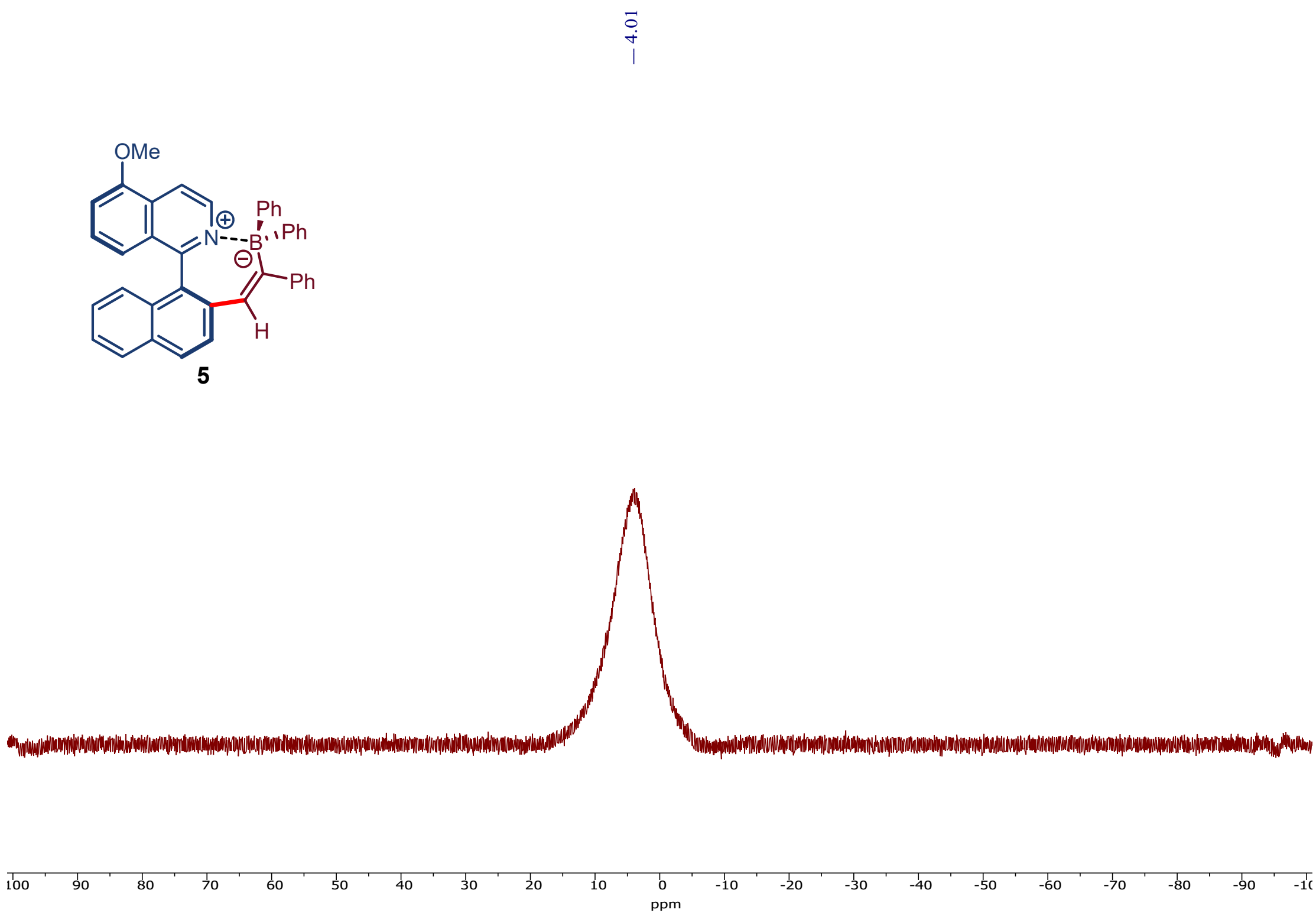

^{1}H NMR (500 MHz, $CDCl_3$) of **6** (*see procedure*)

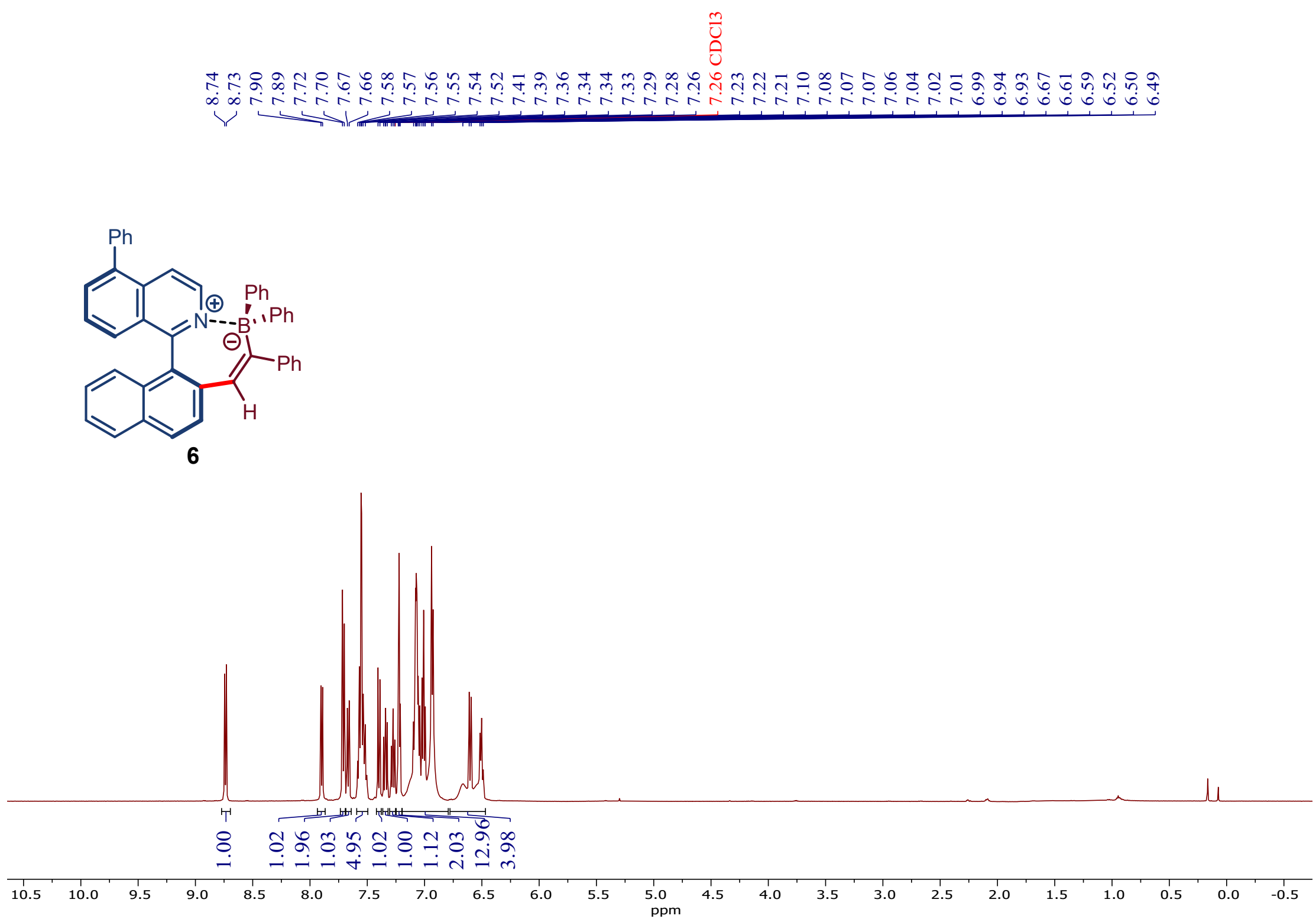

^{13}C NMR (125 MHz, $CDCl_3$) of **6**

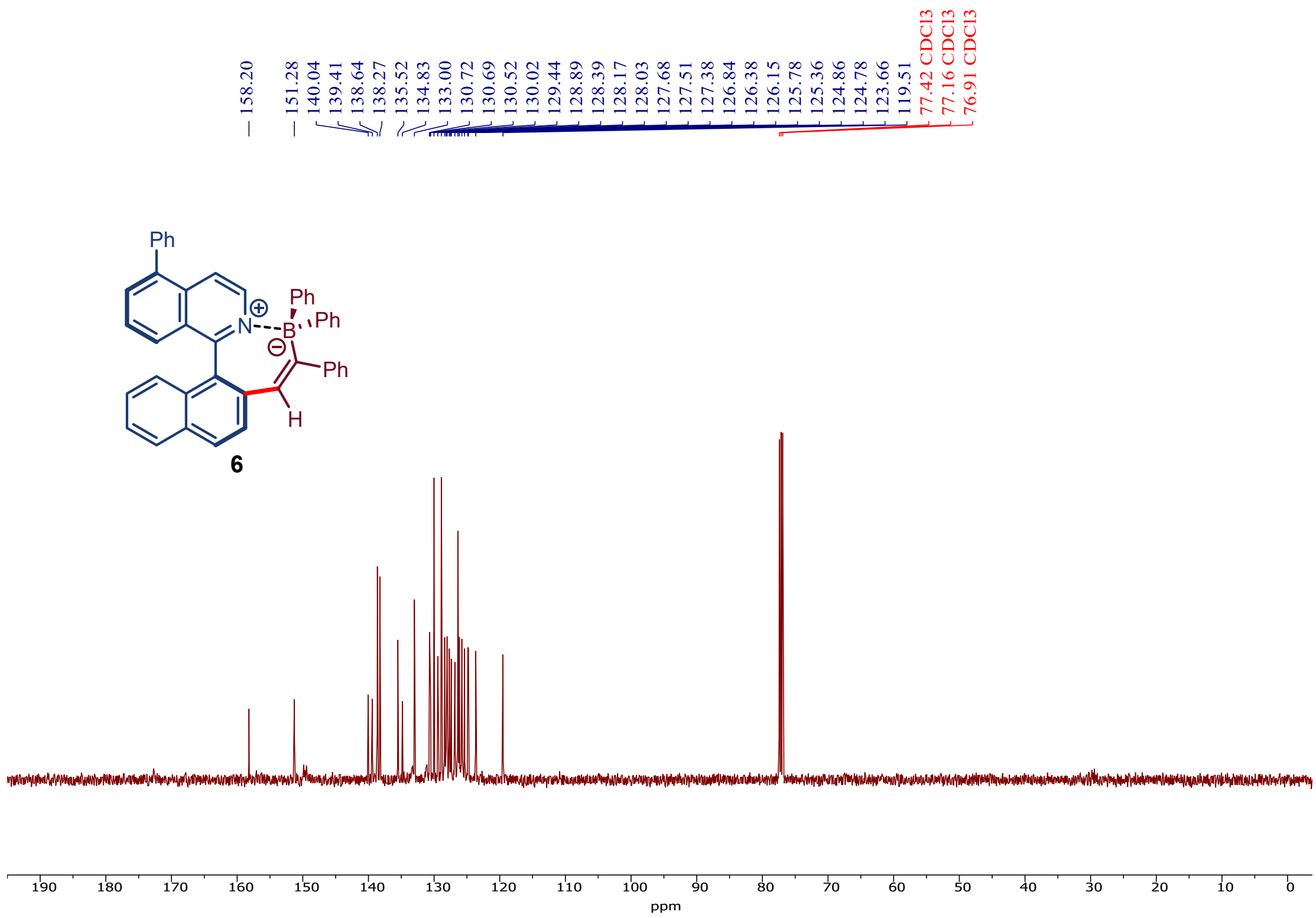

^{11}B NMR (128 MHz, $CDCl_3$) of **6**

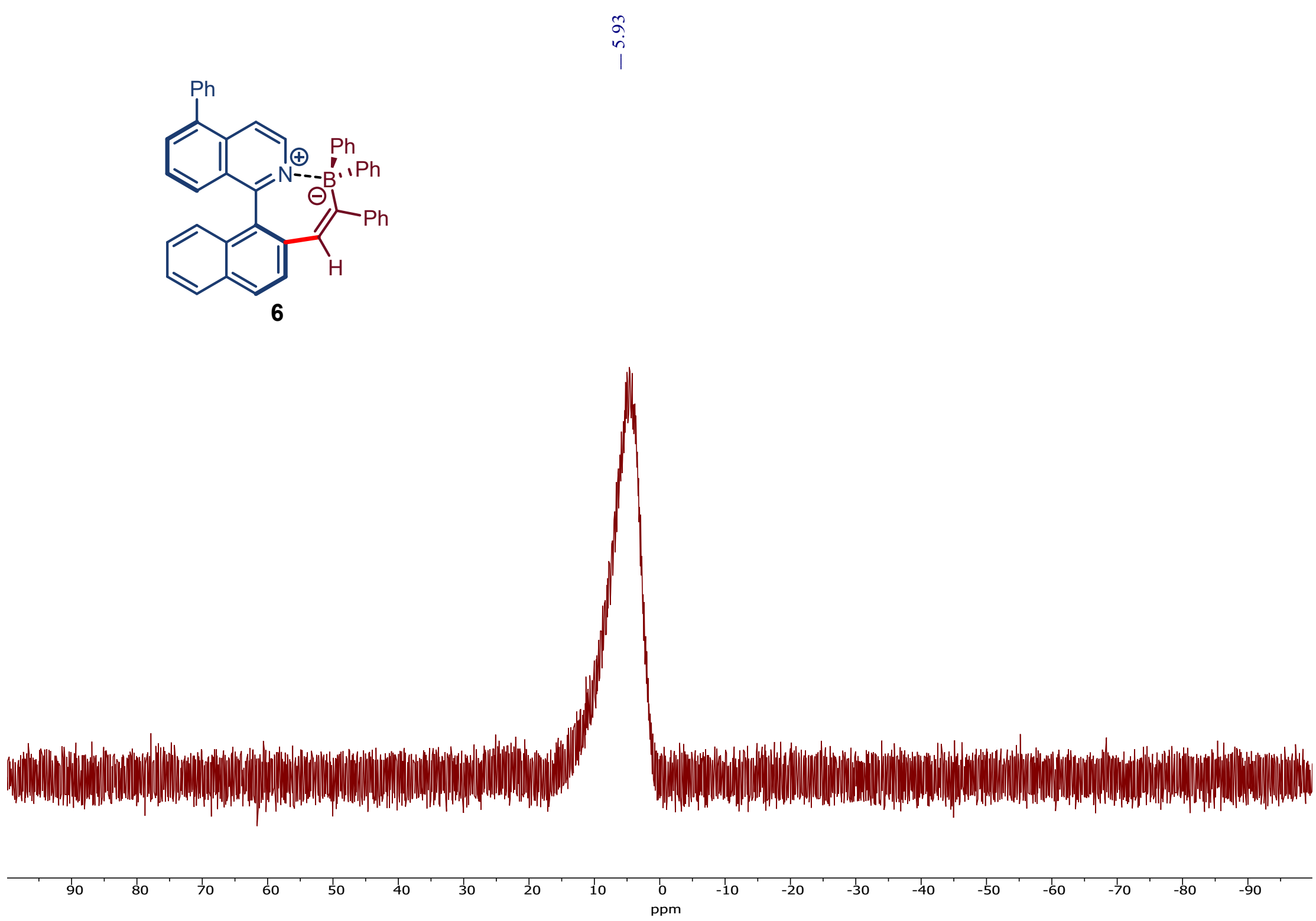

^1H NMR (400 MHz, $CDCl_3$) of **7** (*see procedure*)

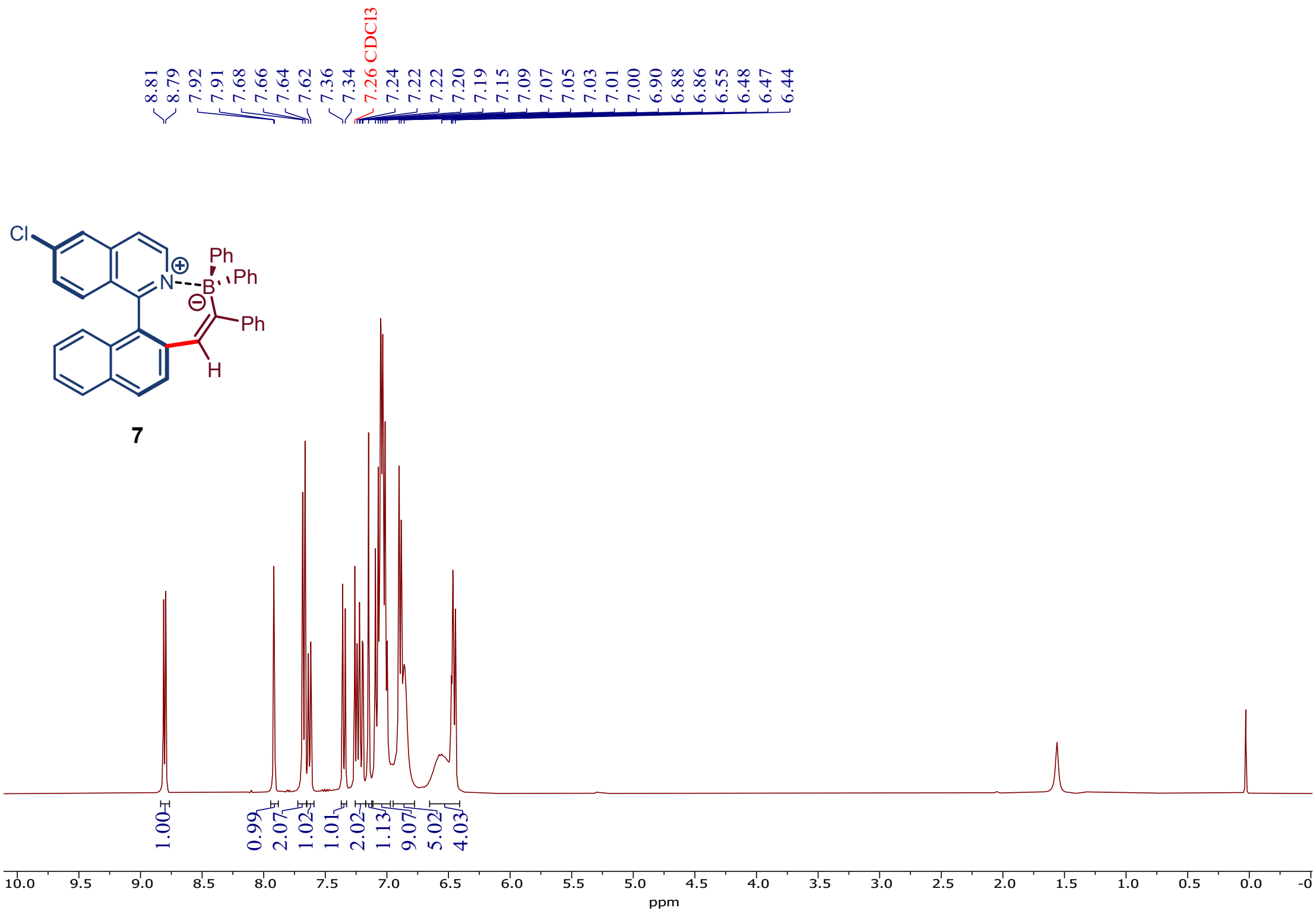

^{13}C NMR (100 MHz, $CDCl_3$) of **7**

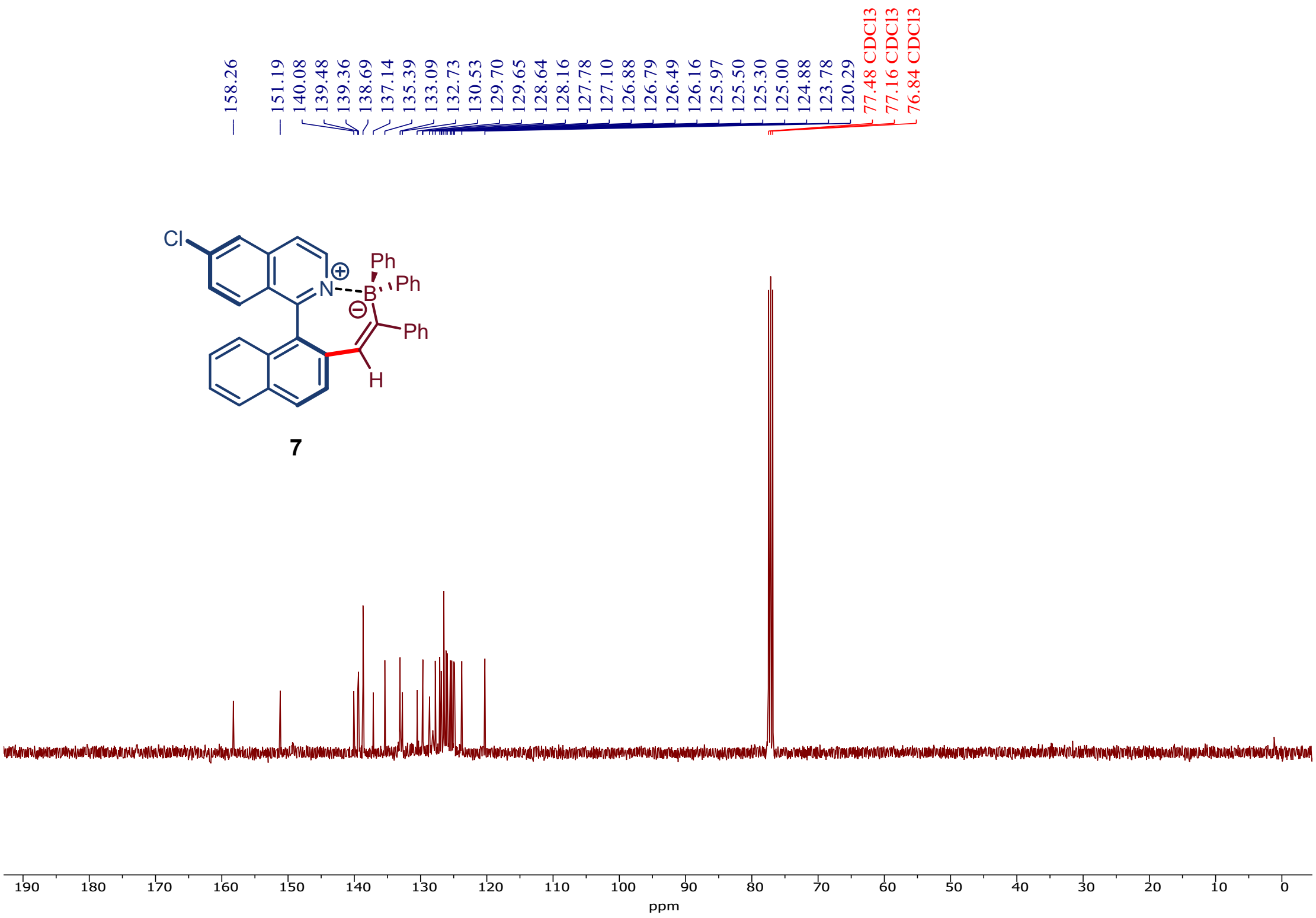

^{11}B NMR (128 MHz, $CDCl_3$) of **7**

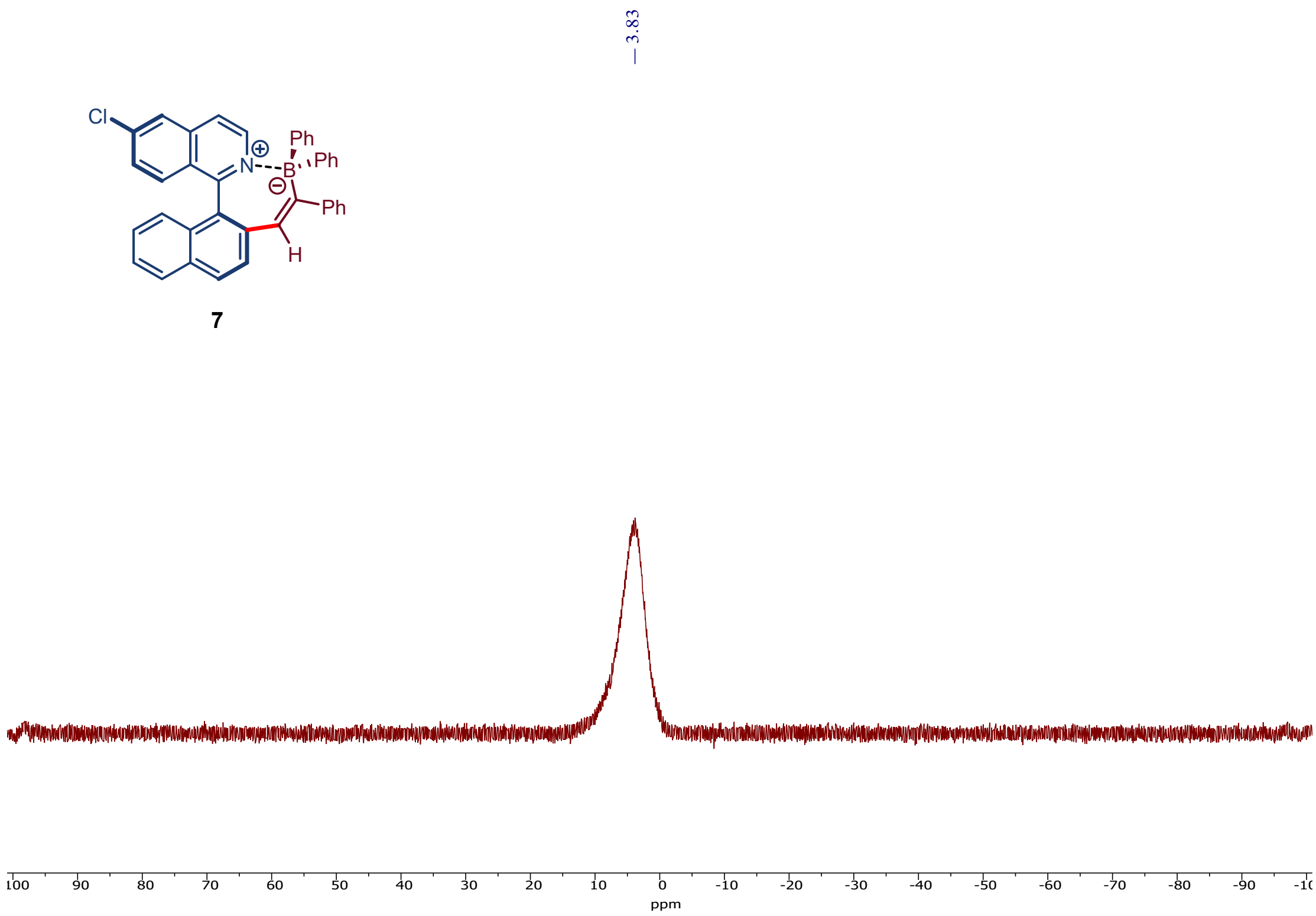

^{1}H NMR (400 MHz, $CDCl_3$) of **8** (*see procedure*)

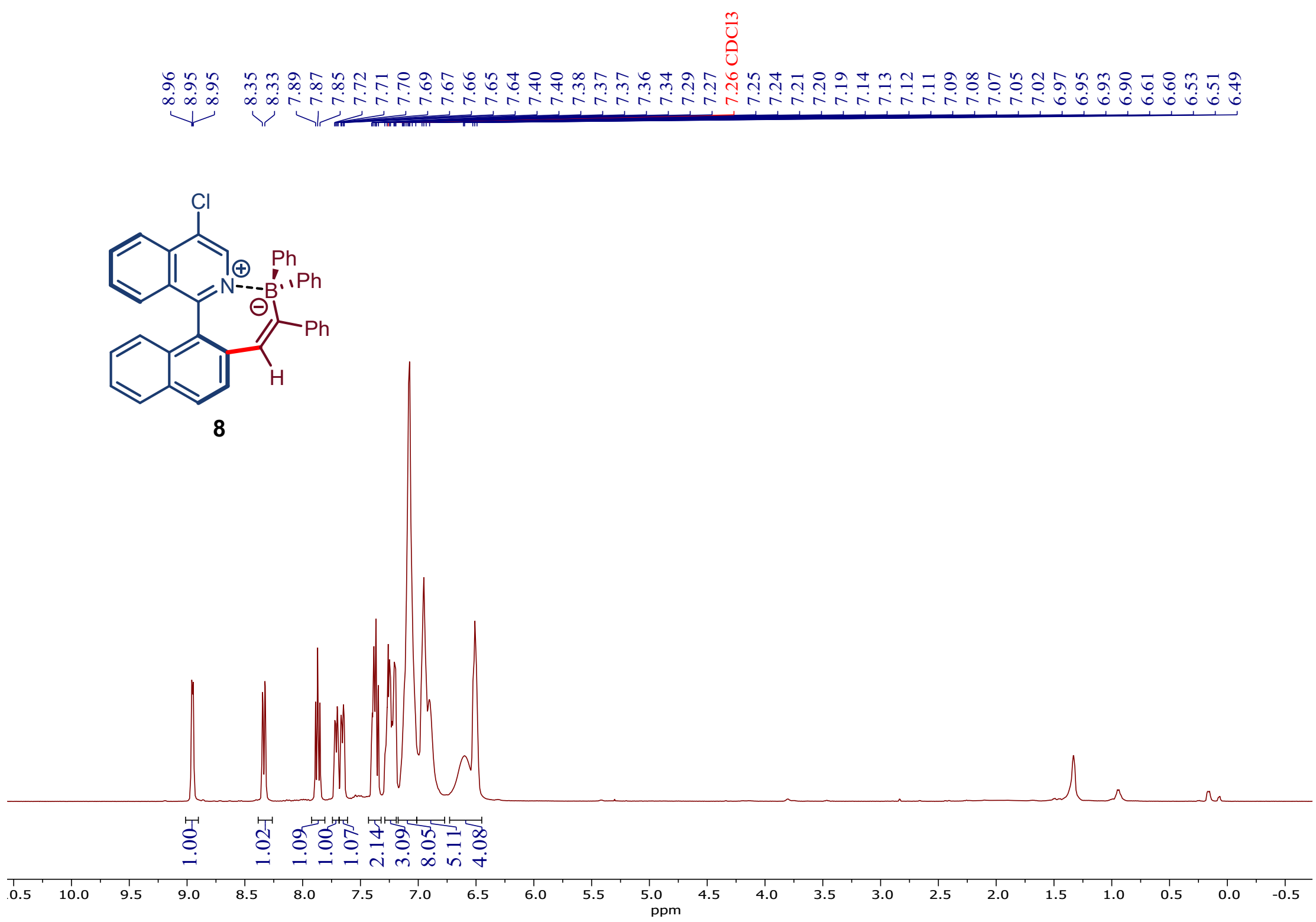

^{13}C NMR (100 MHz, $CDCl_3$) of **8**

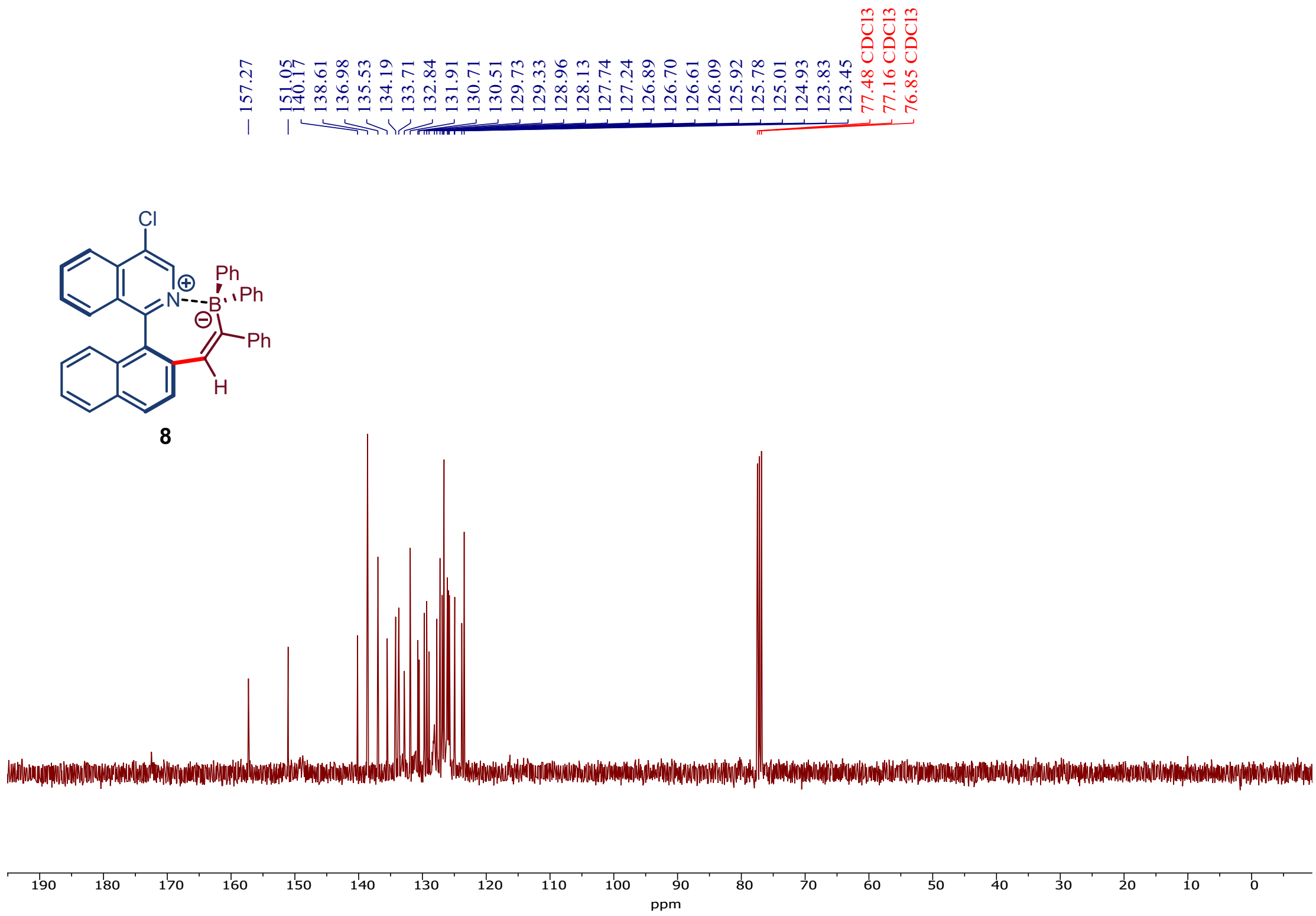

^{11}B NMR (128 MHz, $CDCl_3$) of **8**

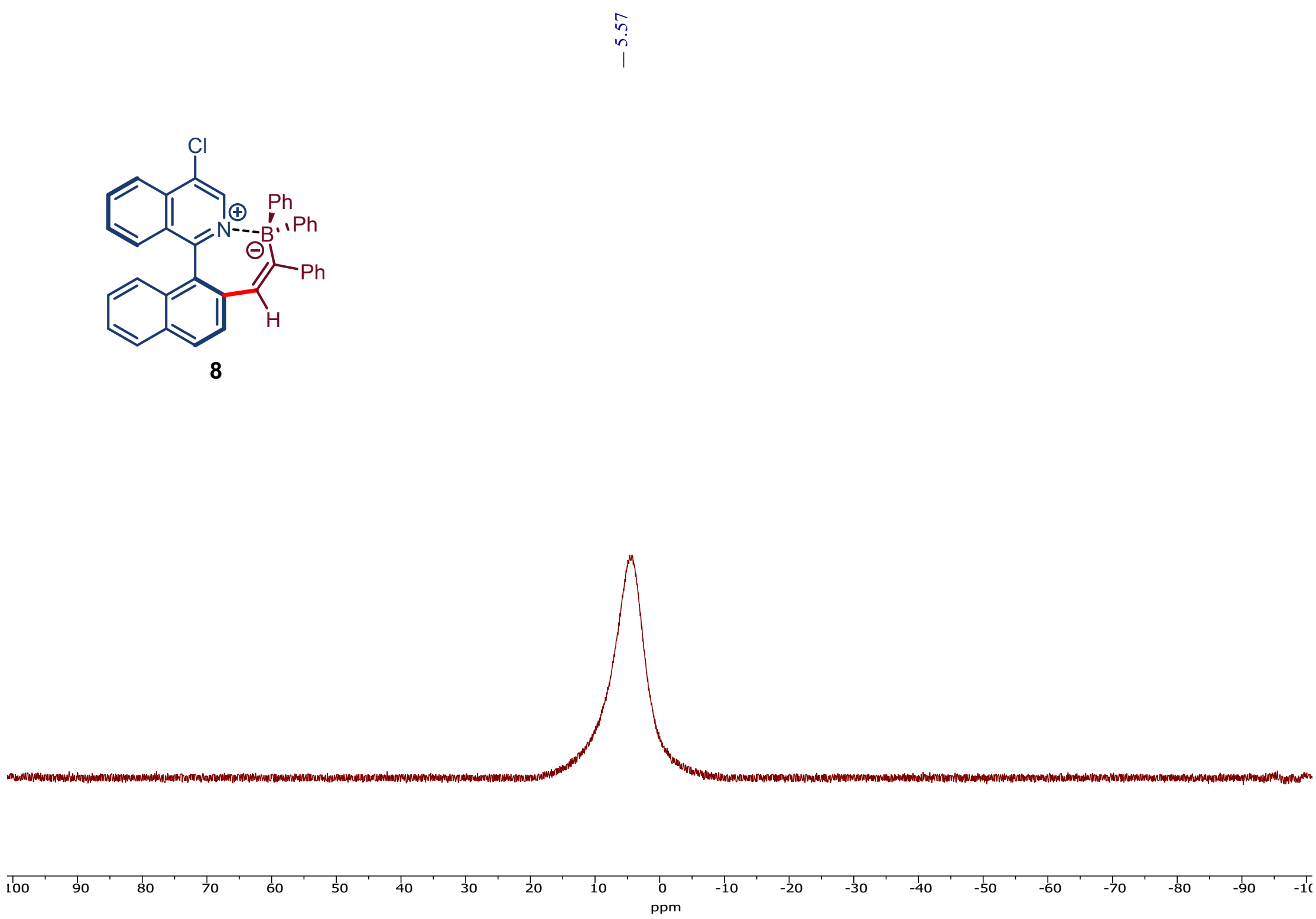

^{1}H NMR (400 MHz, $CDCl_3$) of **9** (*see procedure*)

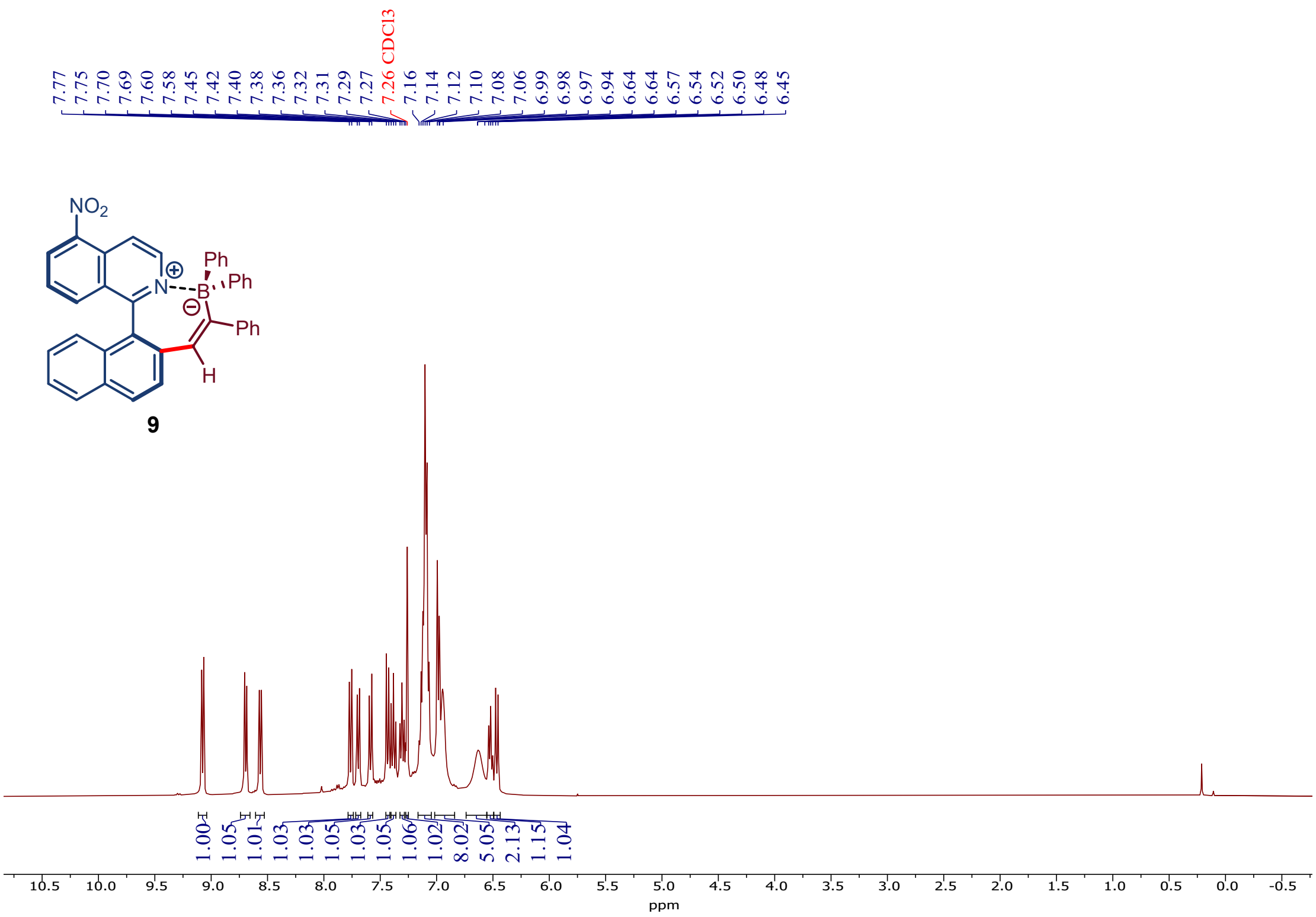

^{13}C NMR (100 MHz, $CDCl_3$) of **9**

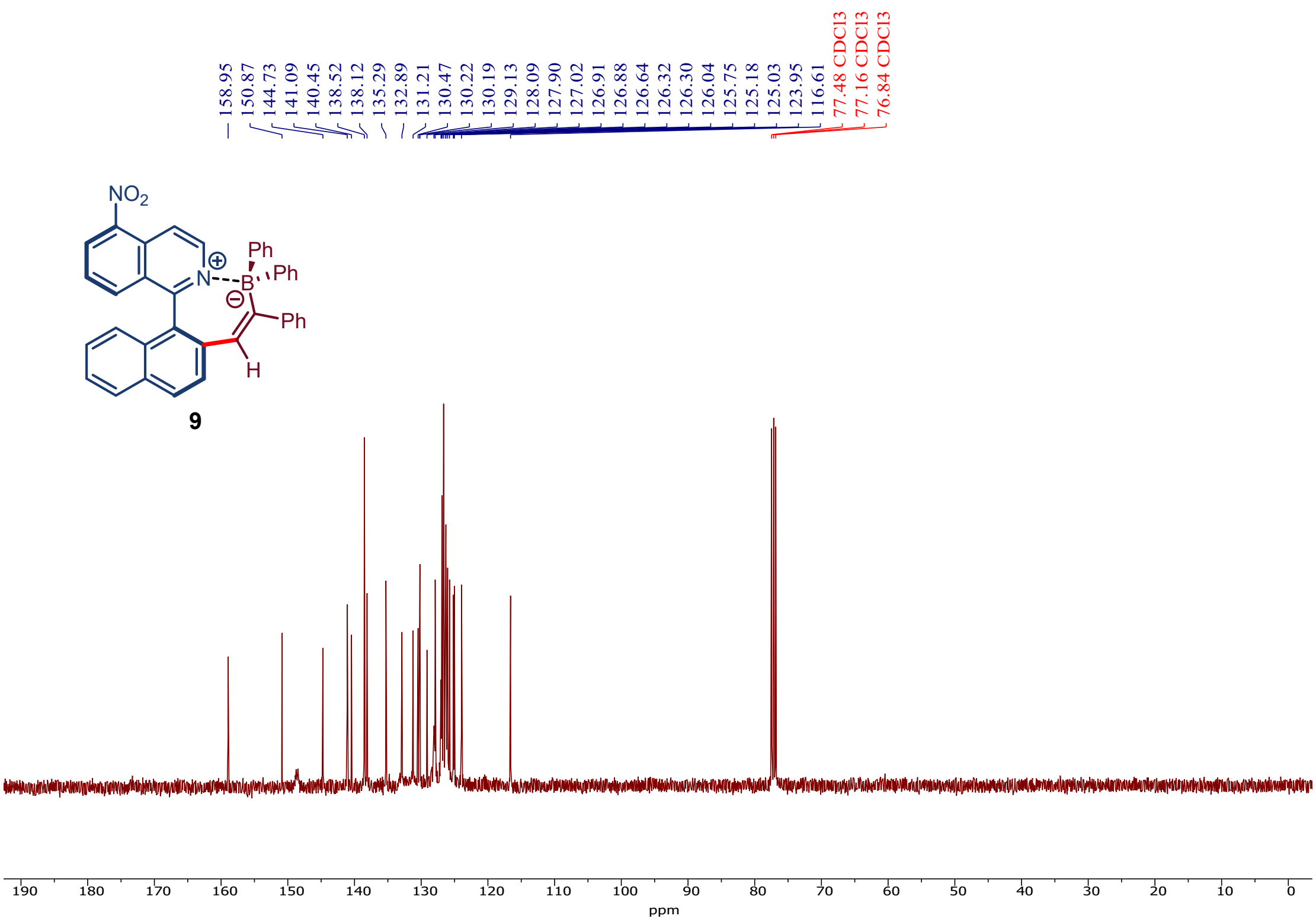

^{11}B NMR (128 MHz, $CDCl_3$) of **9**

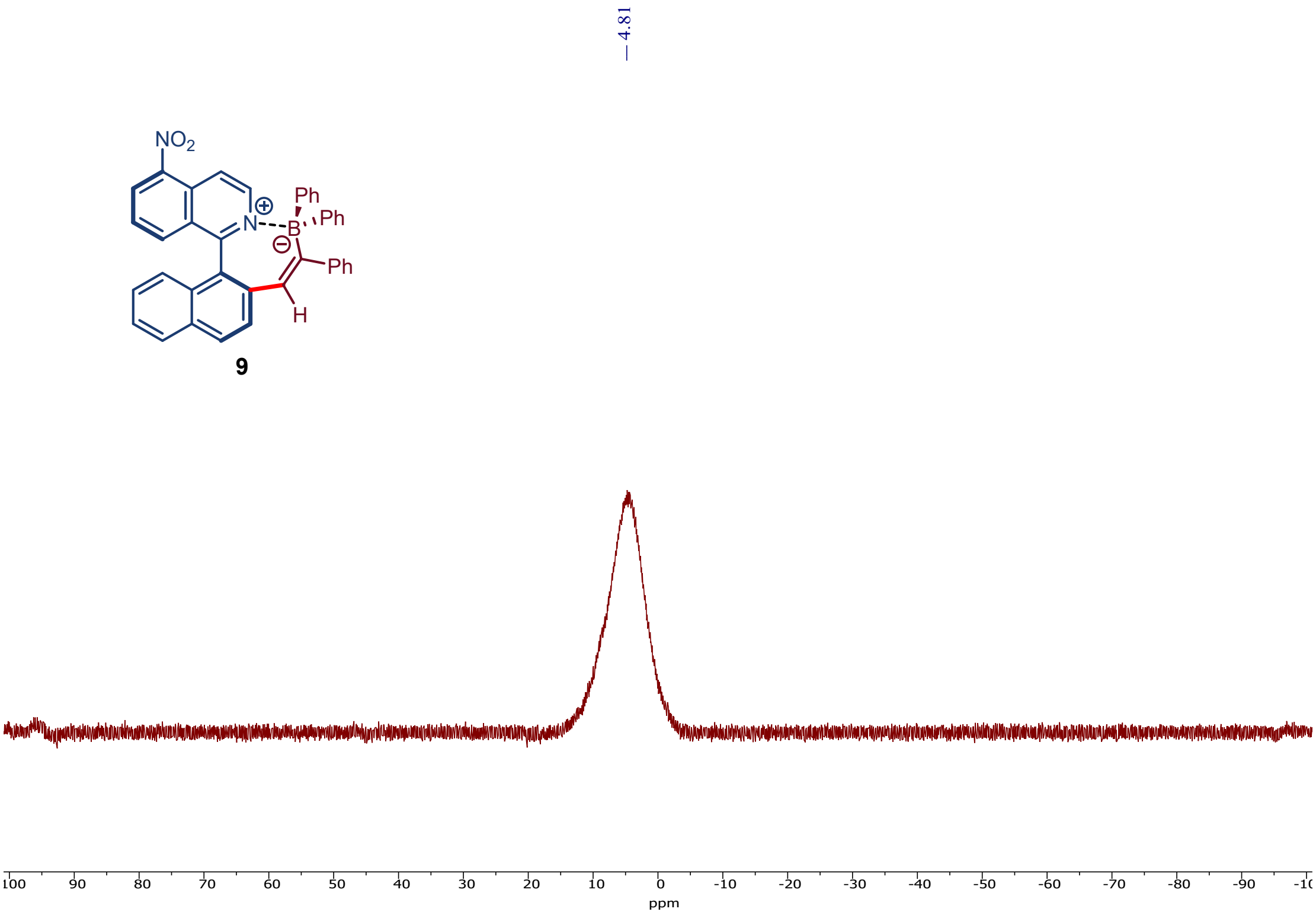

^{1}H NMR (400 MHz, $CDCl_3$) of **10** (*see procedure*)

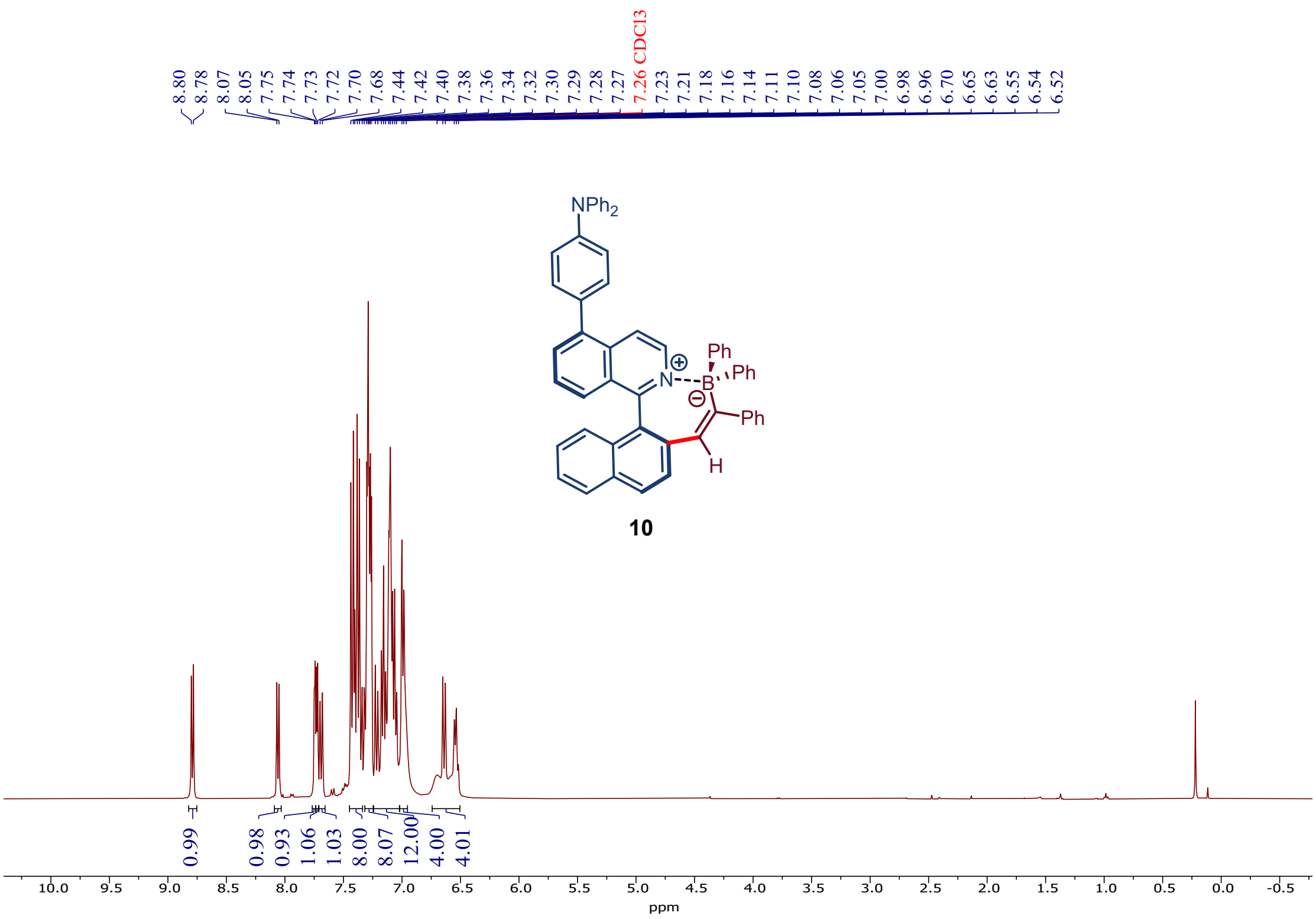

^{13}C NMR (100 MHz, $CDCl_3$) of **10**

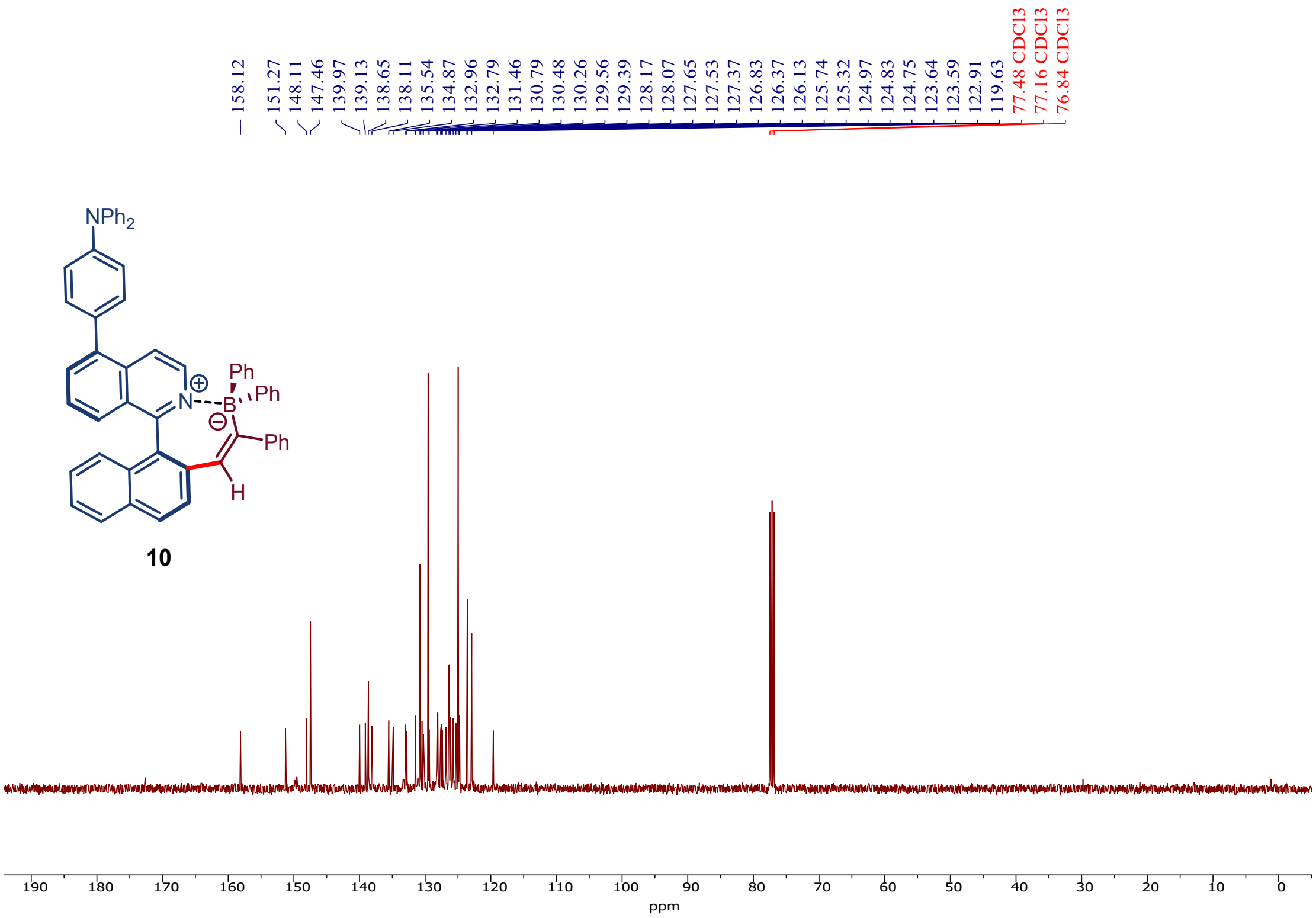

^{11}B NMR (128 MHz, $CDCl_3$) of **10**

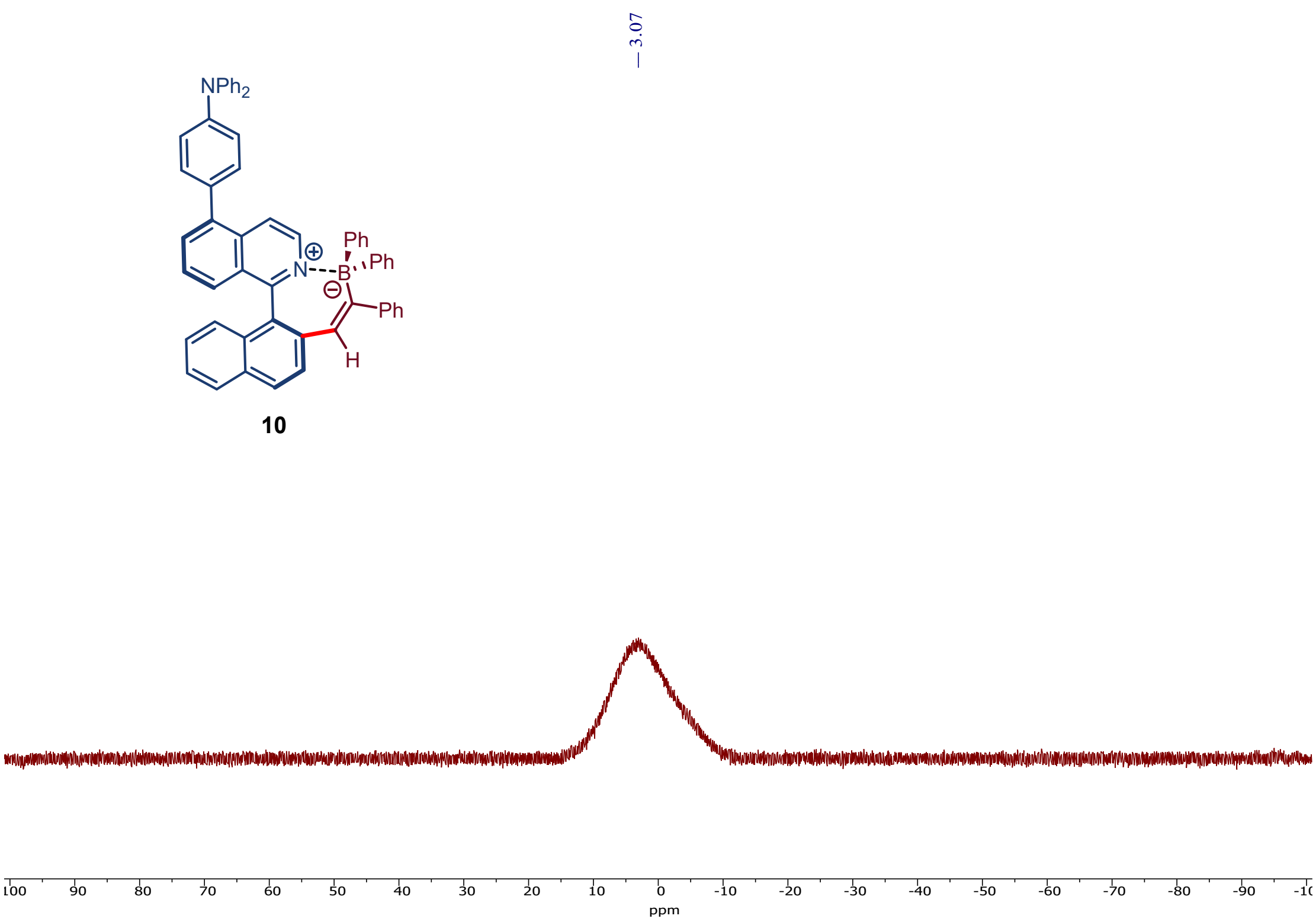

^{1}H NMR (400 MHz, $CDCl_3$) of **11** (*see procedure*)

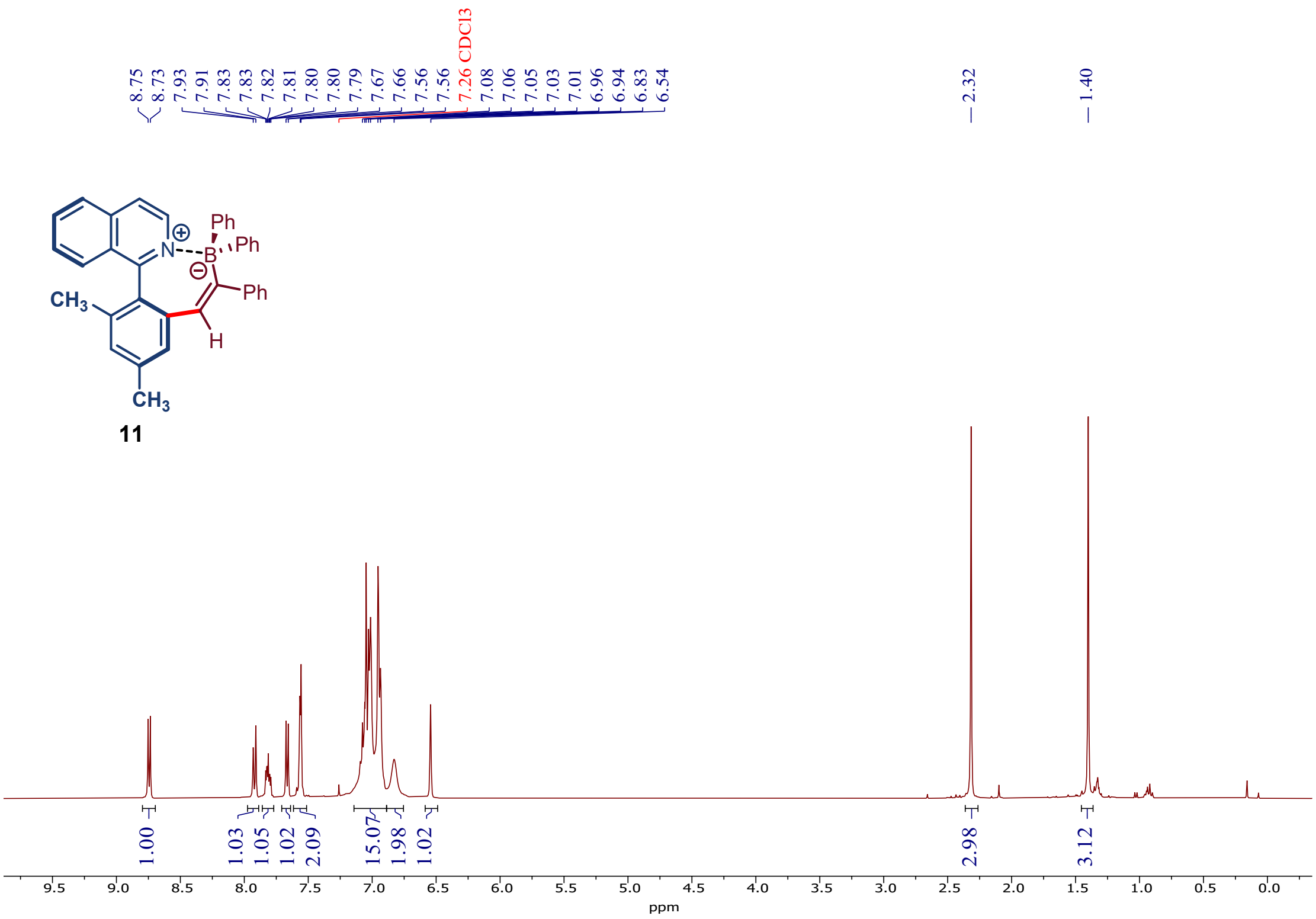

^{13}C NMR (100 MHz, $CDCl_3$) of **11**

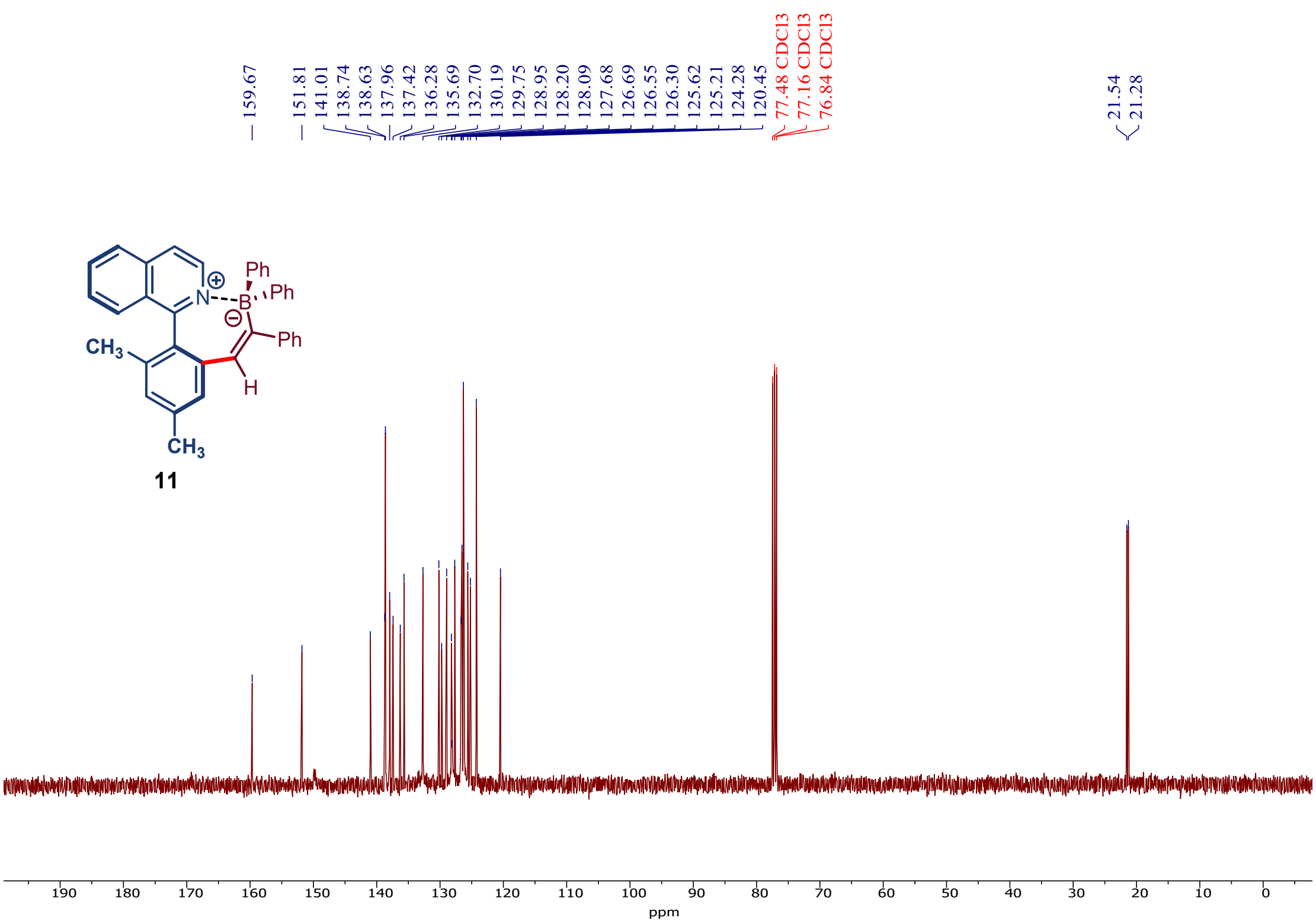

^{11}B NMR (128 MHz, $CDCl_3$) of **11**

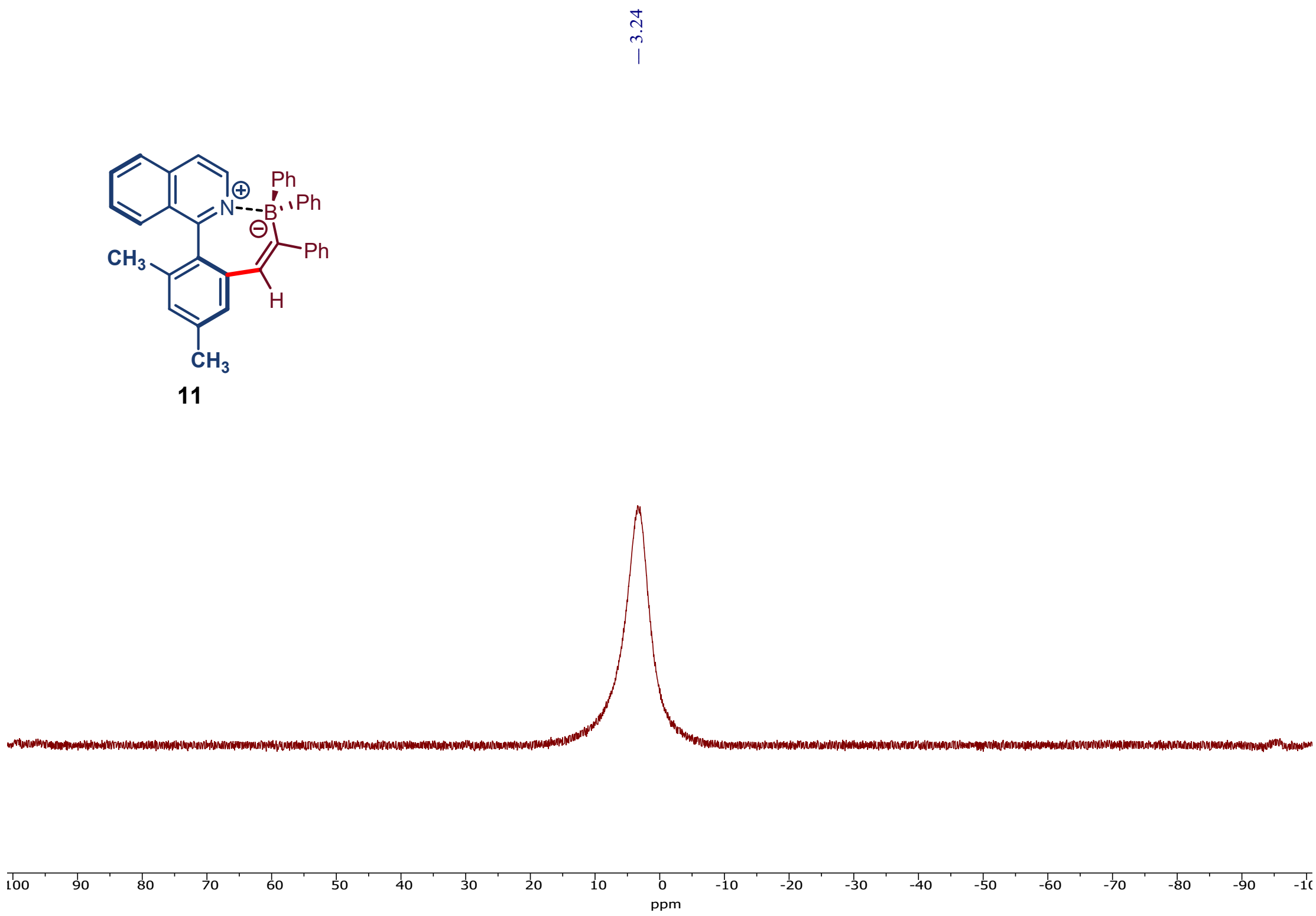

^{1}H NMR (400 MHz, $CDCl_3$) of **12** (*see procedure*)

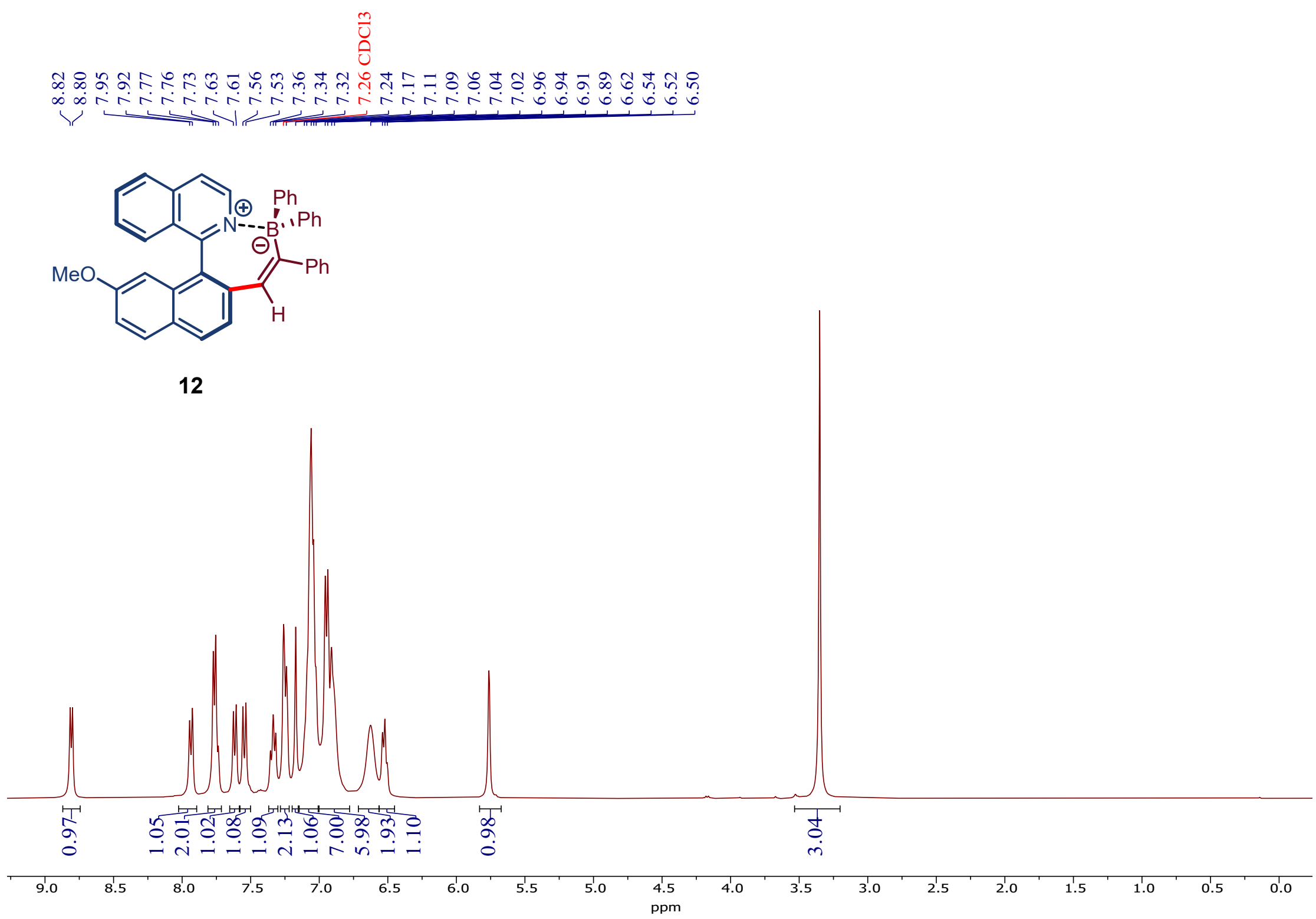

^{13}C NMR (100 MHz, $CDCl_3$) of **12**

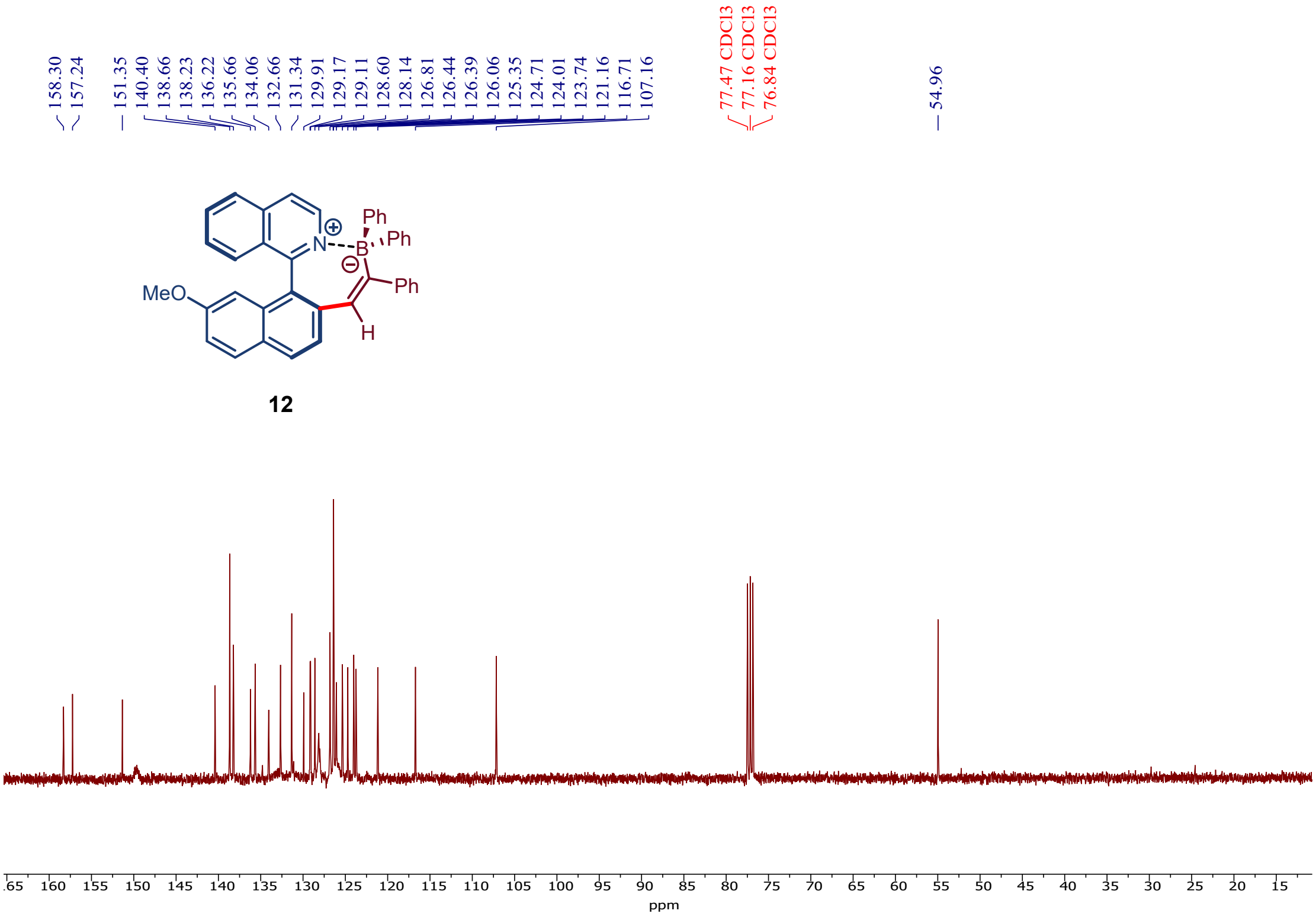

^{11}B NMR (128 MHz, $CDCl_3$) of **12**

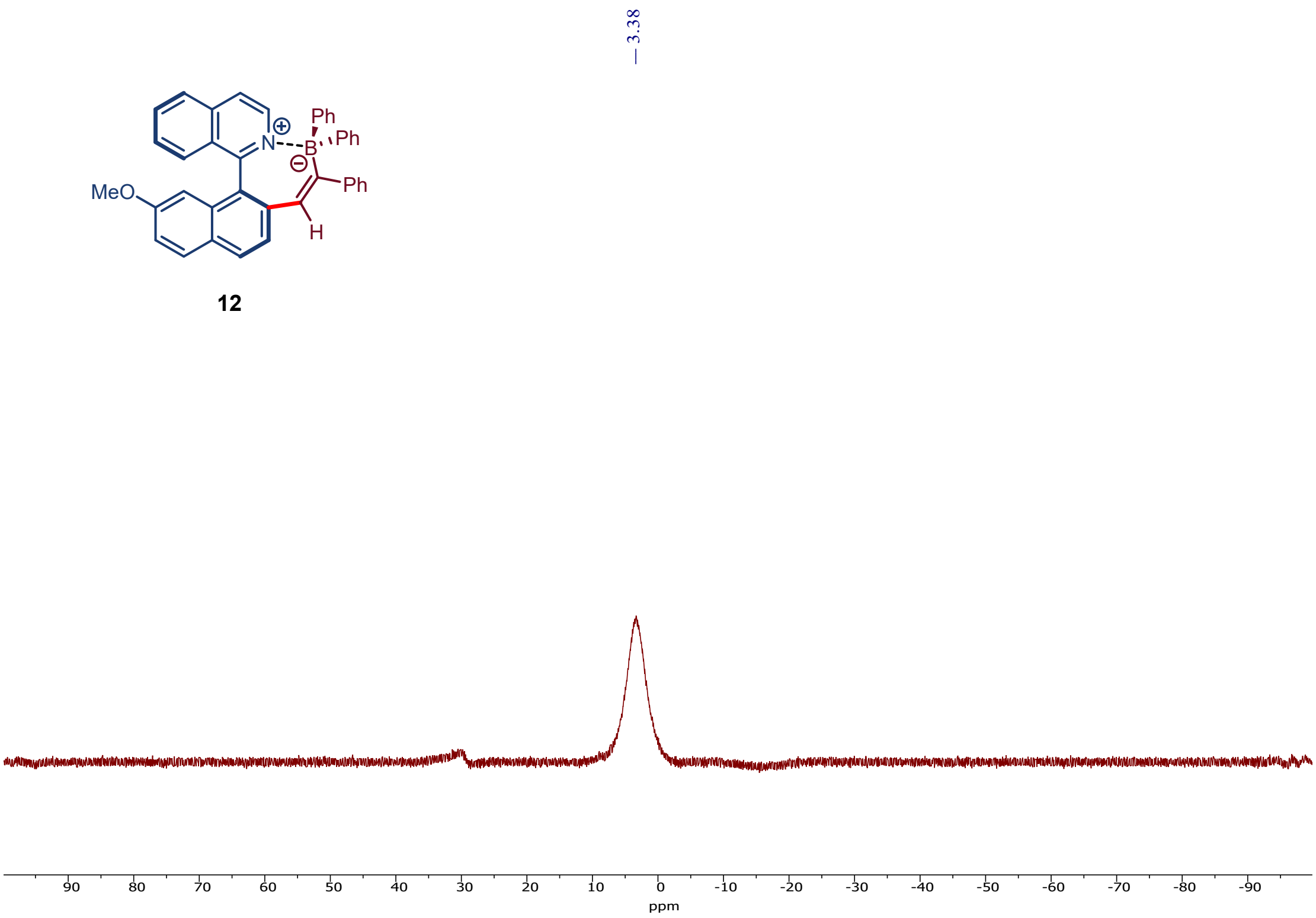

^{1}H NMR (400 MHz, $CDCl_3$) of **13** (see procedure)

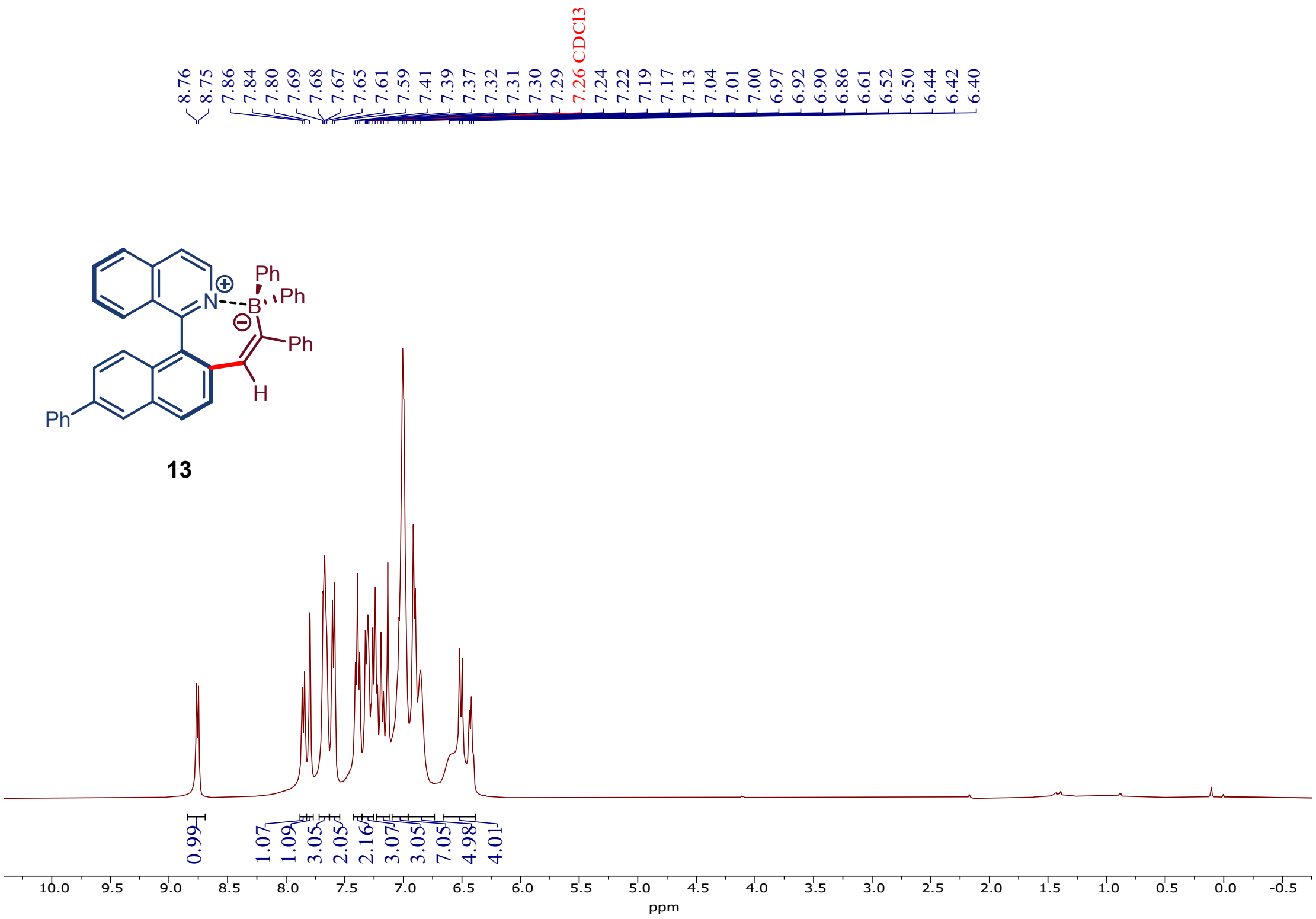

^{13}C NMR (100 MHz, $CDCl_3$) of **13**

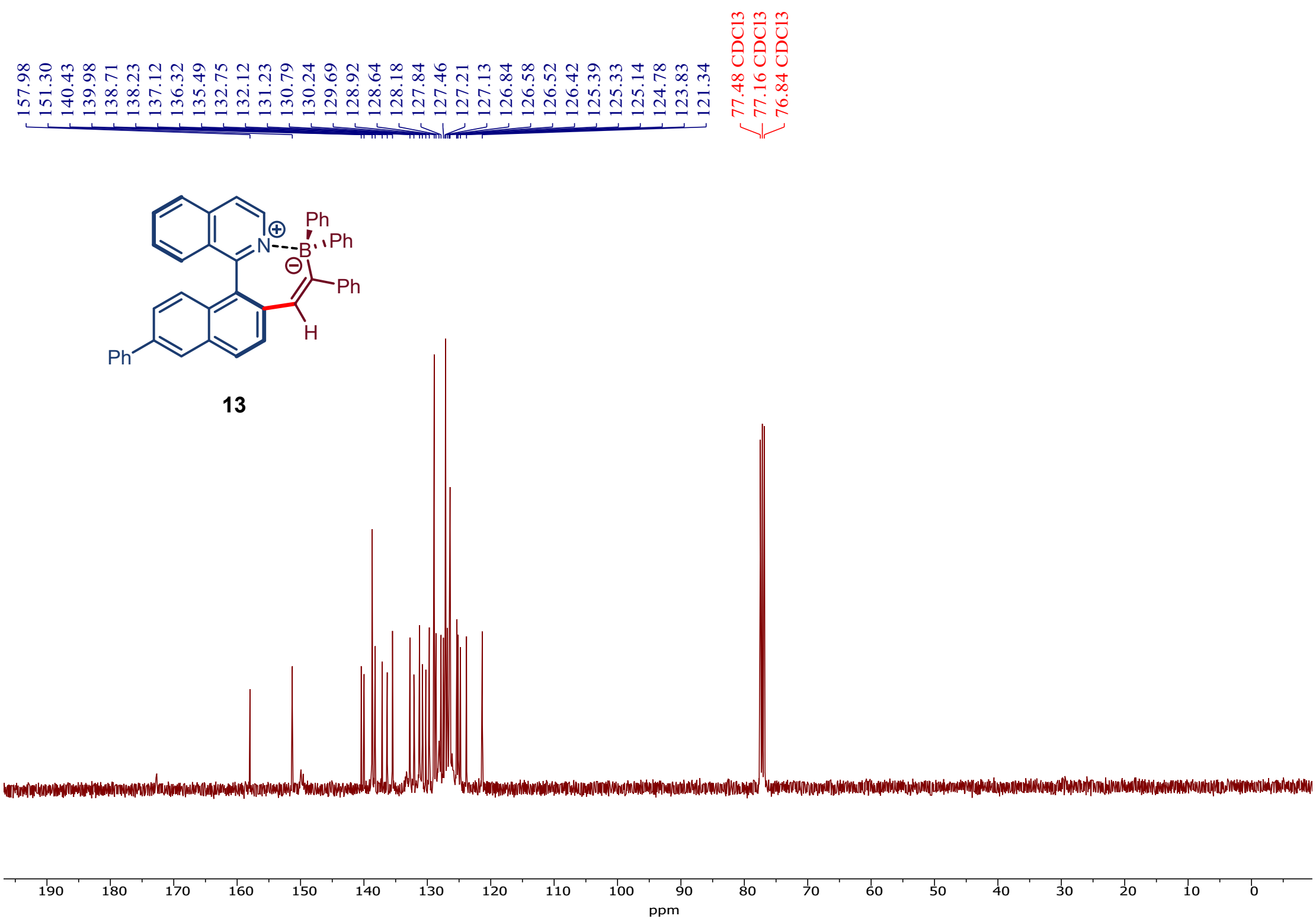

^{11}B NMR (128 MHz, $CDCl_3$) of **13**

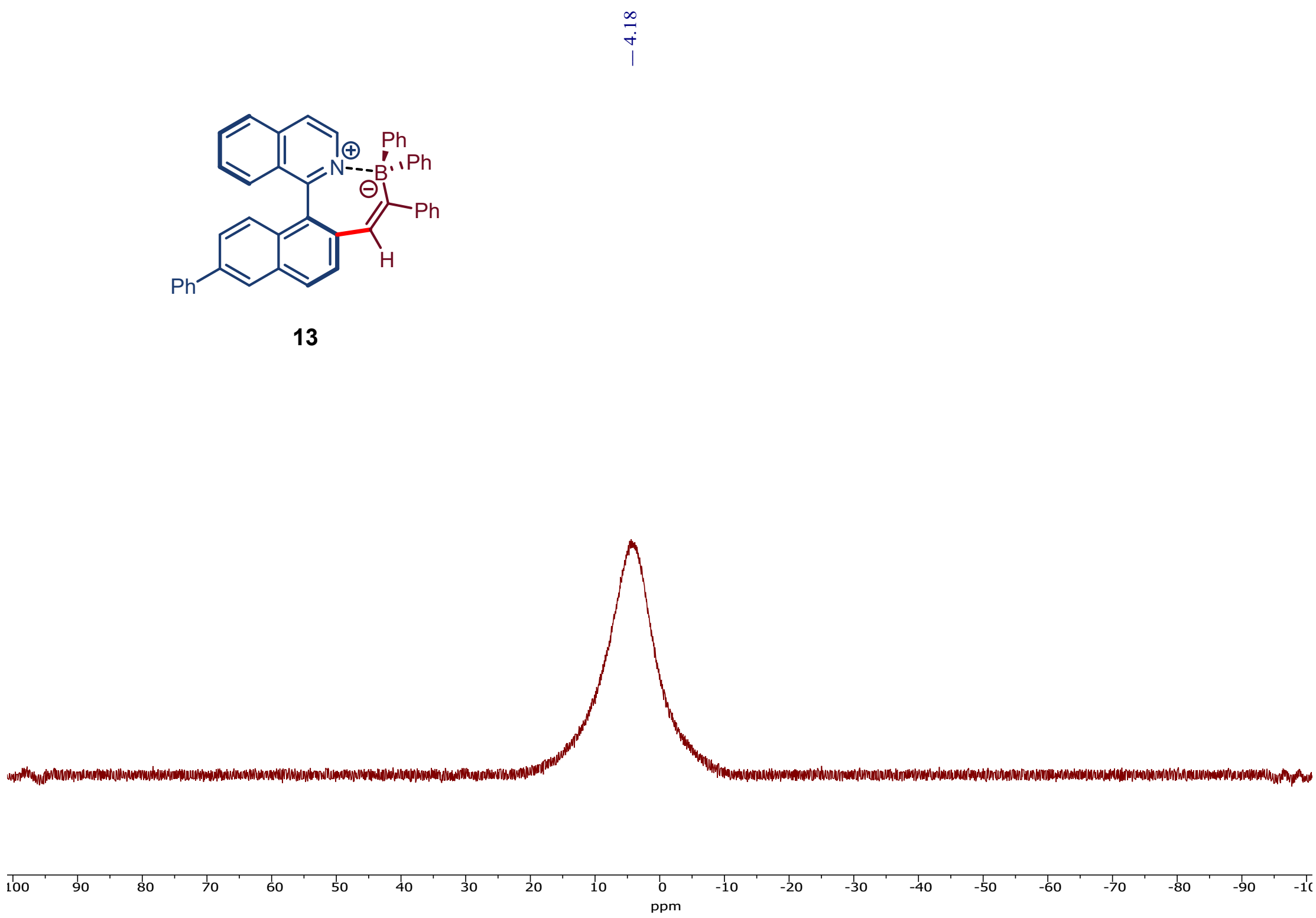

^{1}H NMR (400 MHz, $CDCl_3$) of **14** (*see procedure*)

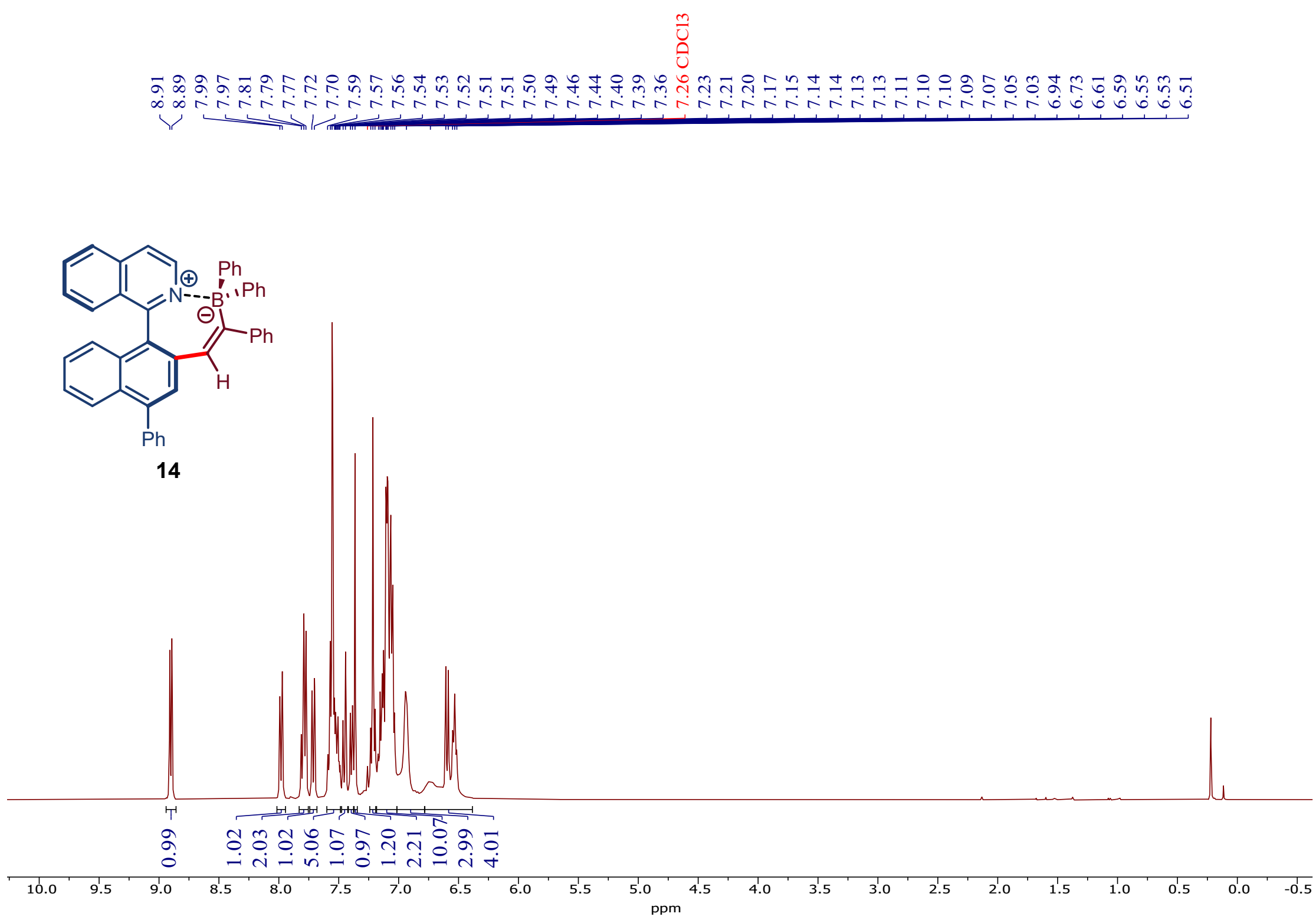

^{13}C NMR (100 MHz, $CDCl_3$) of **14**

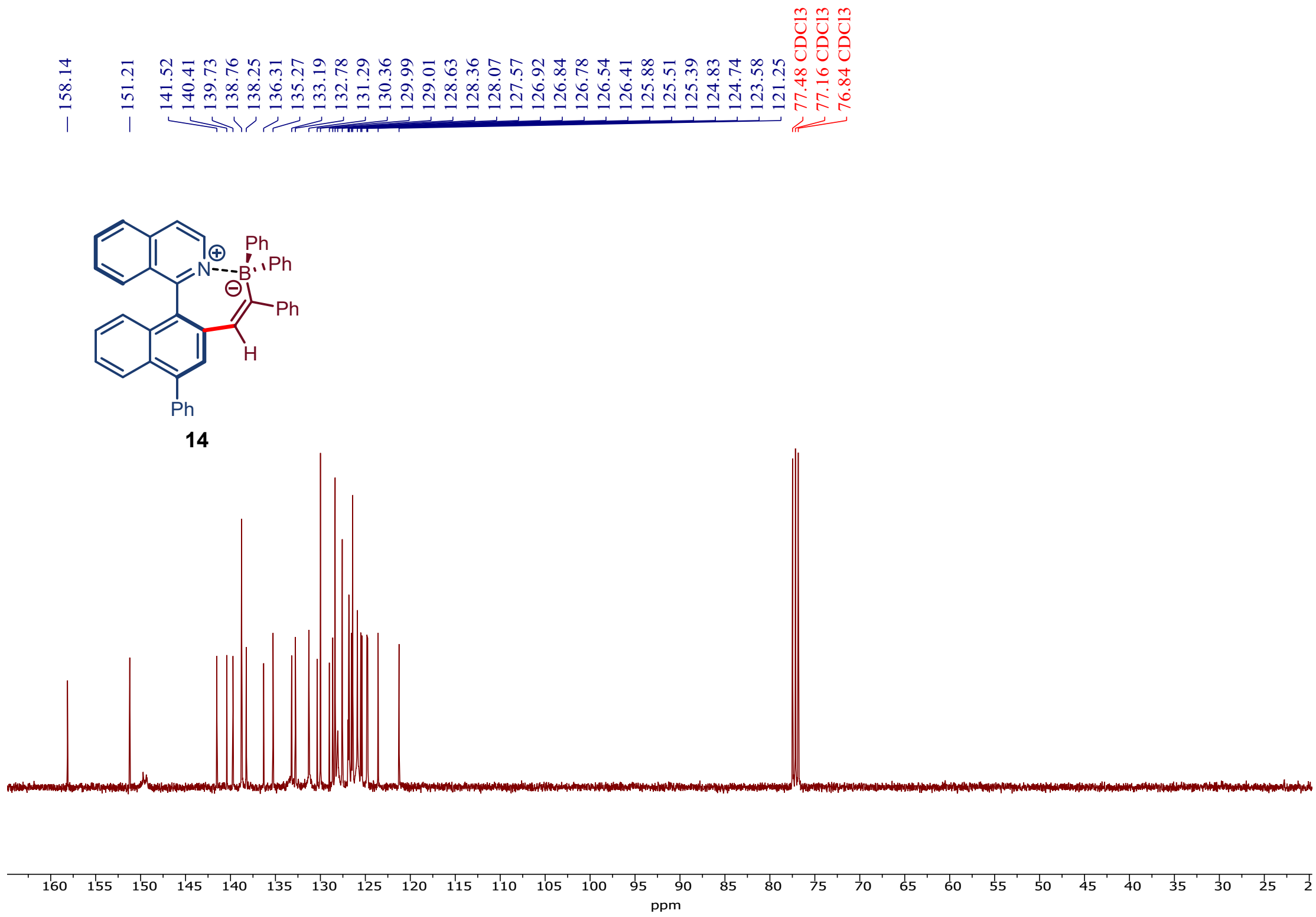

^{11}B NMR (128 MHz, $CDCl_3$) of **14**

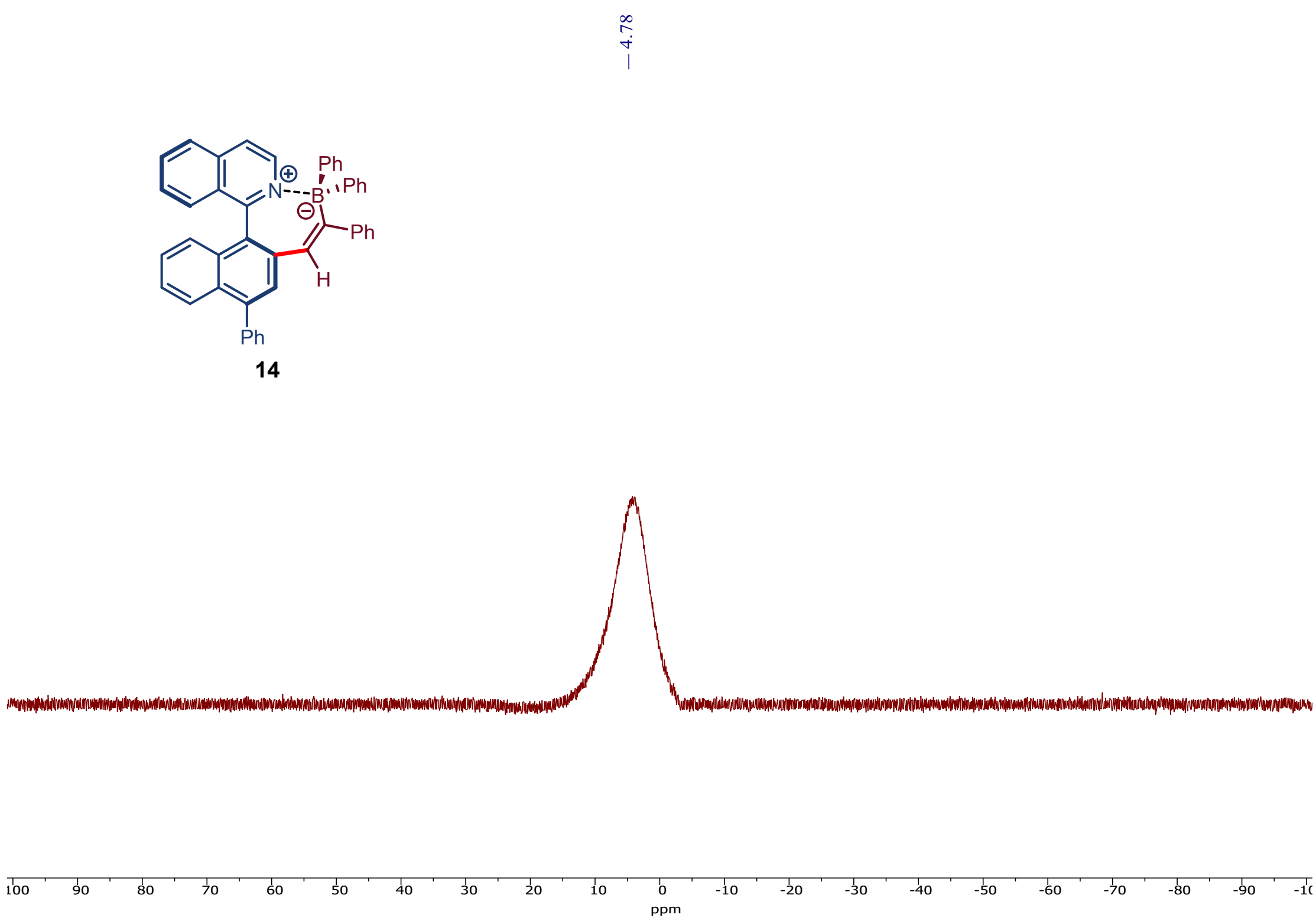

^{1}H NMR (400 MHz, $CDCl_3$) of **15** (*see procedure*)

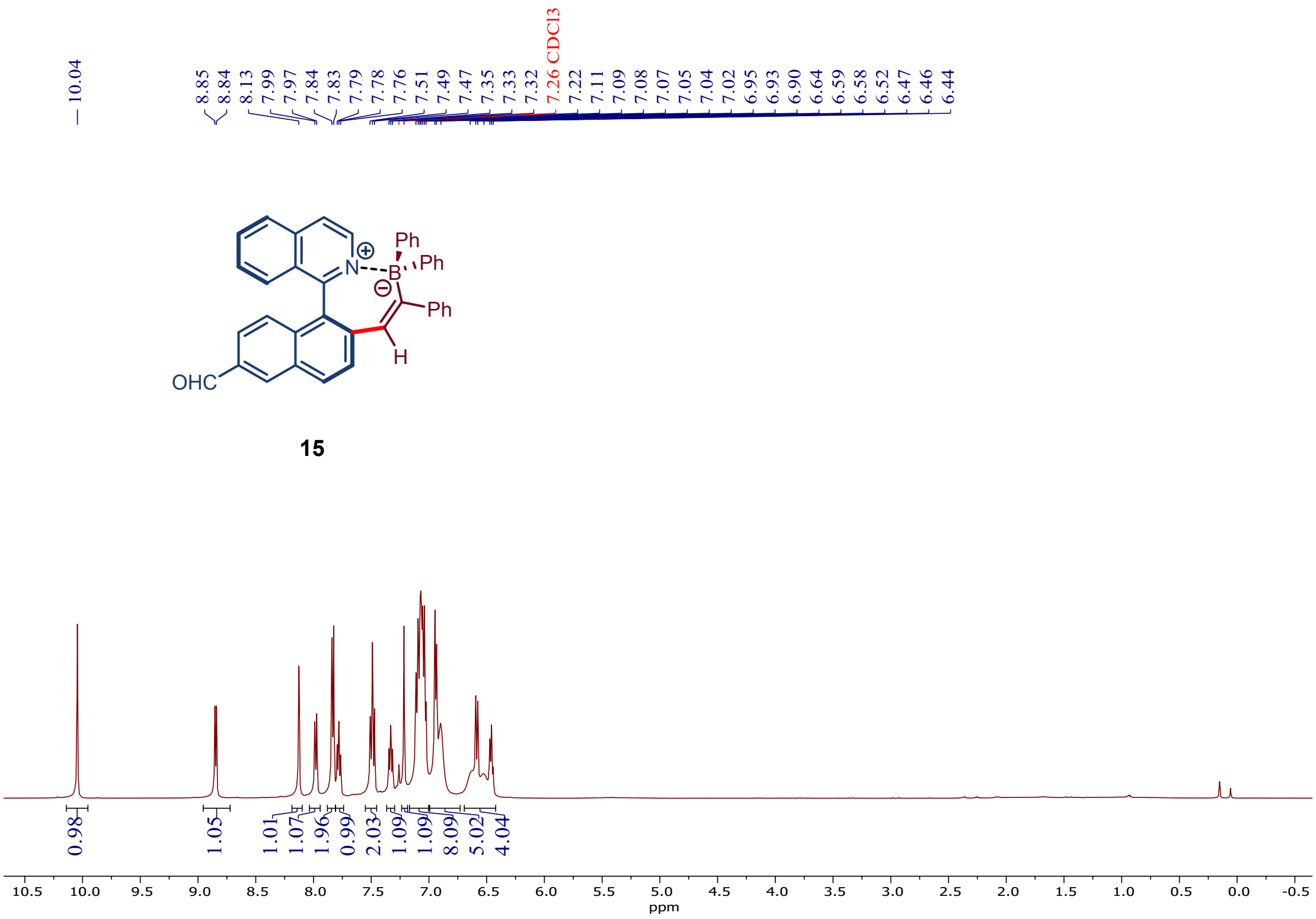

^{13}C NMR (100 MHz, $CDCl_3$) of **15**

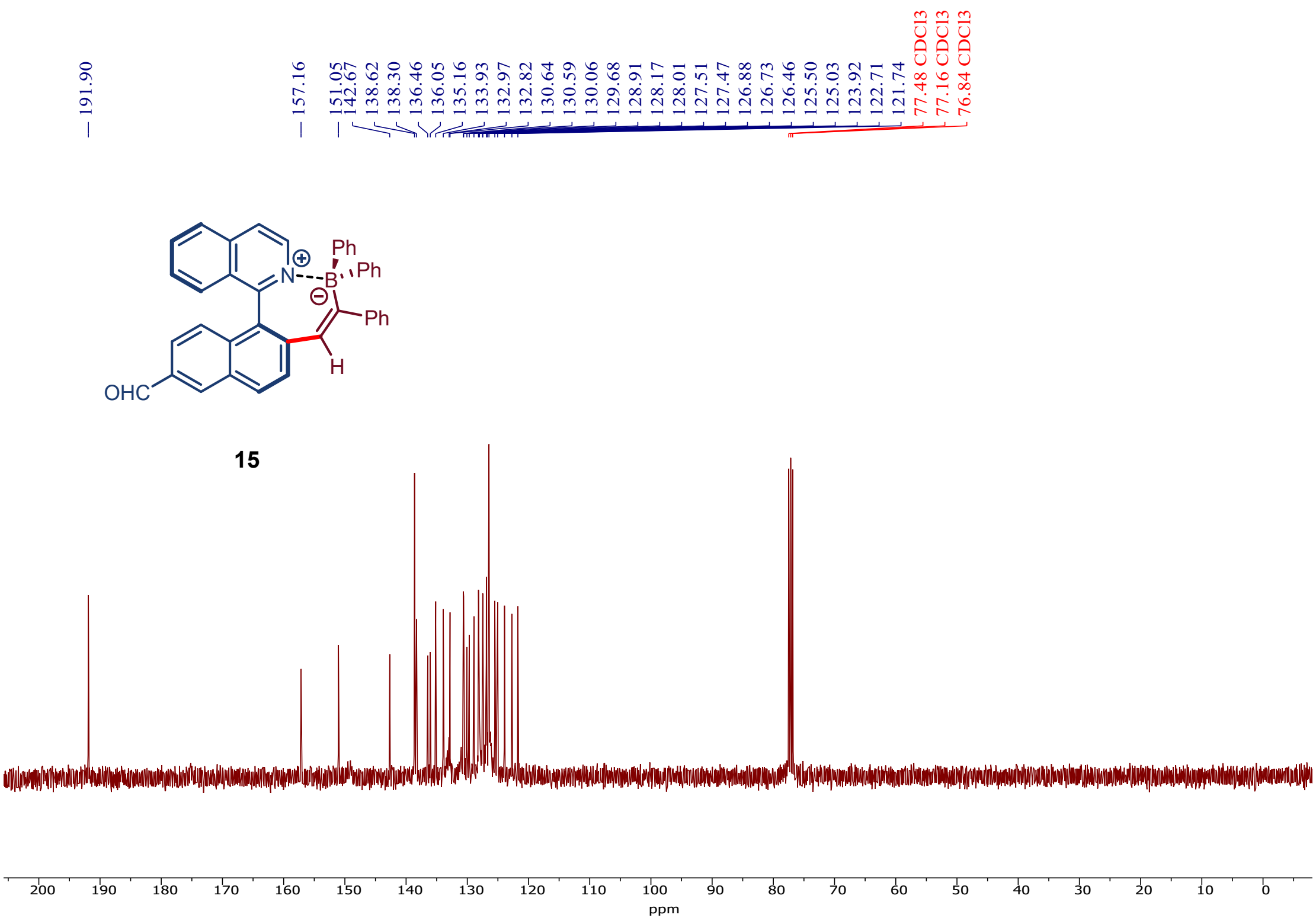

^{11}B NMR (128 MHz, $CDCl_3$) of **15**

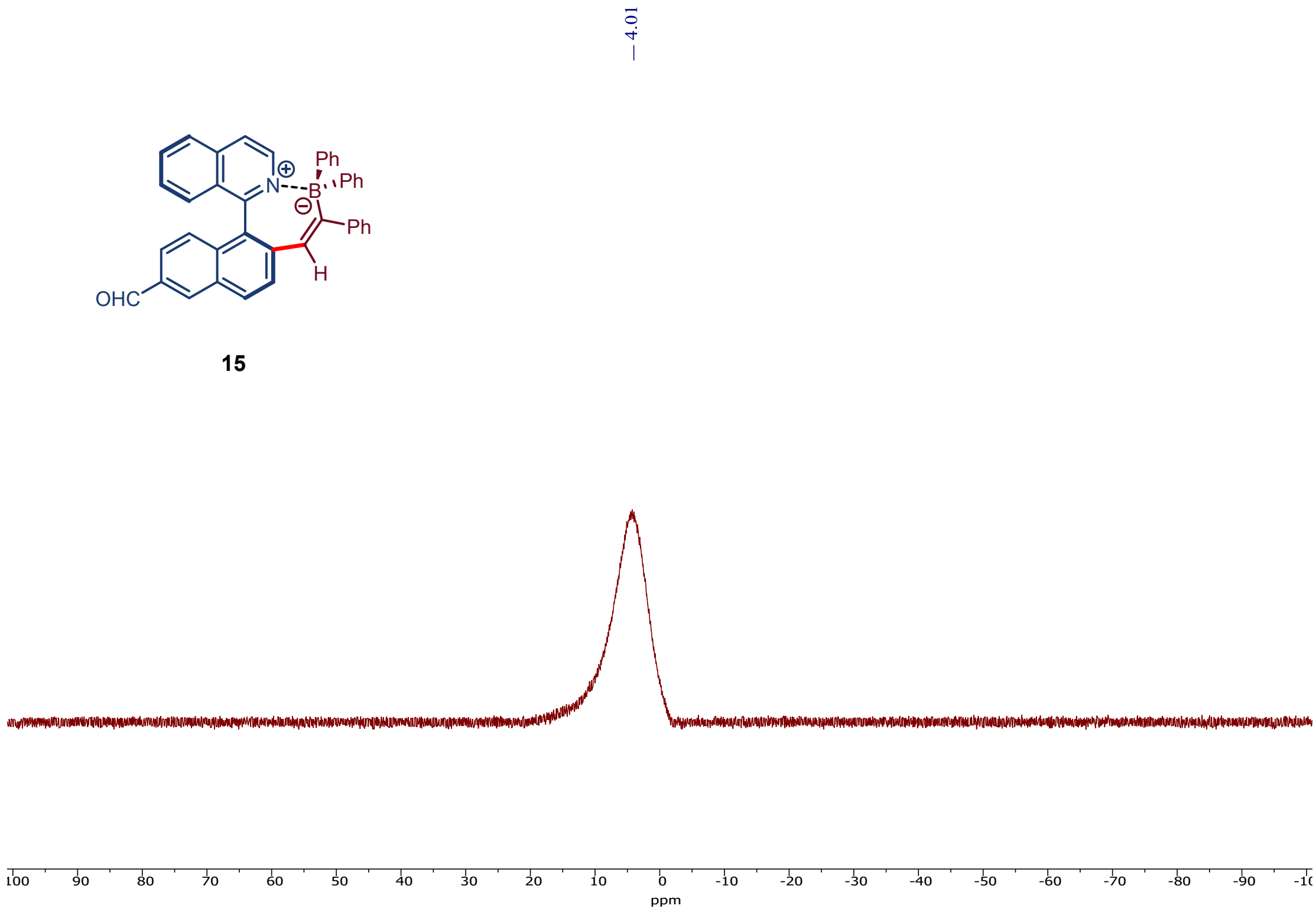

^{1}H NMR (400 MHz, $CDCl_3$) of **16** (*see procedure*)

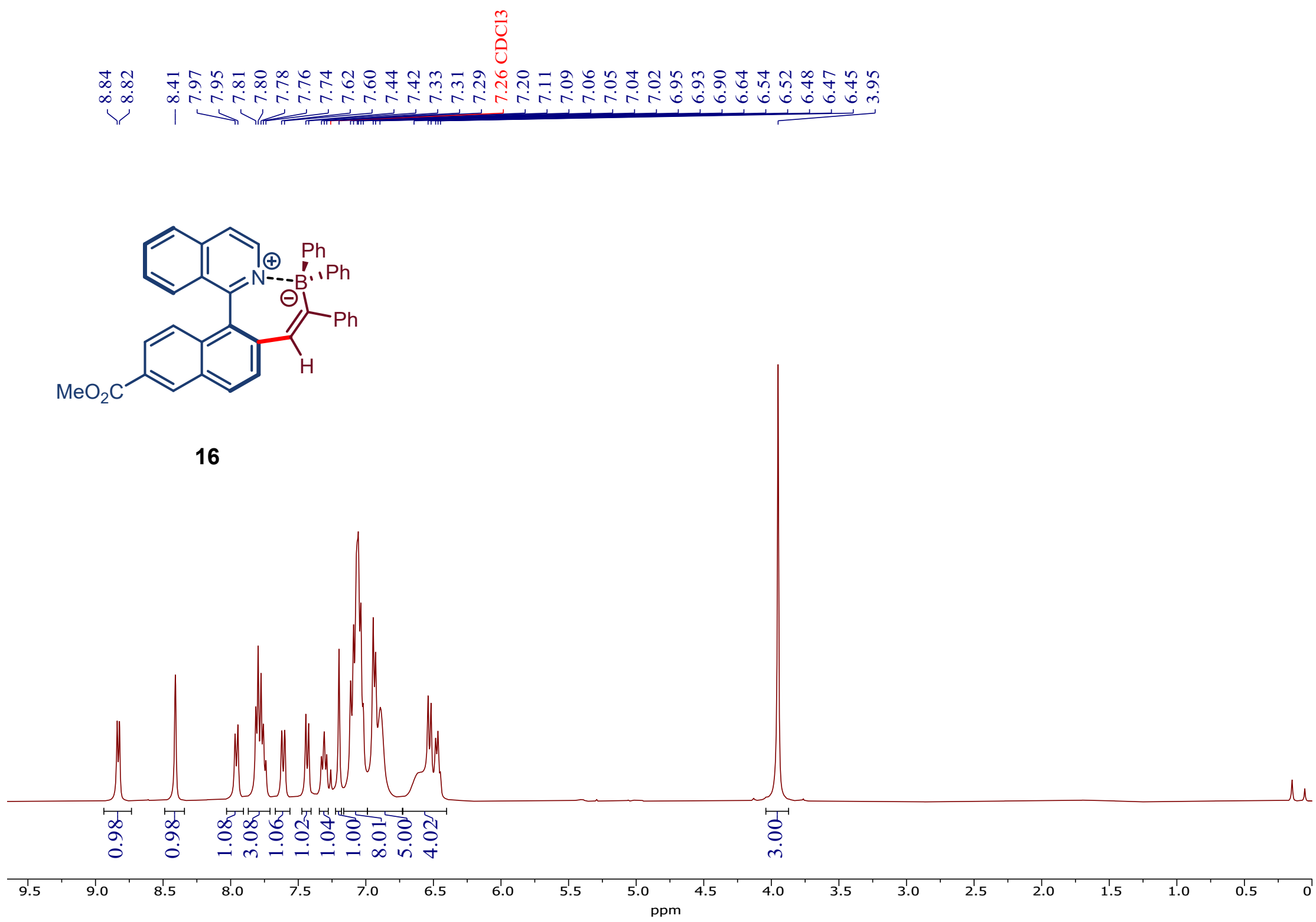

^{13}C NMR (100 MHz, $CDCl_3$) of **16**

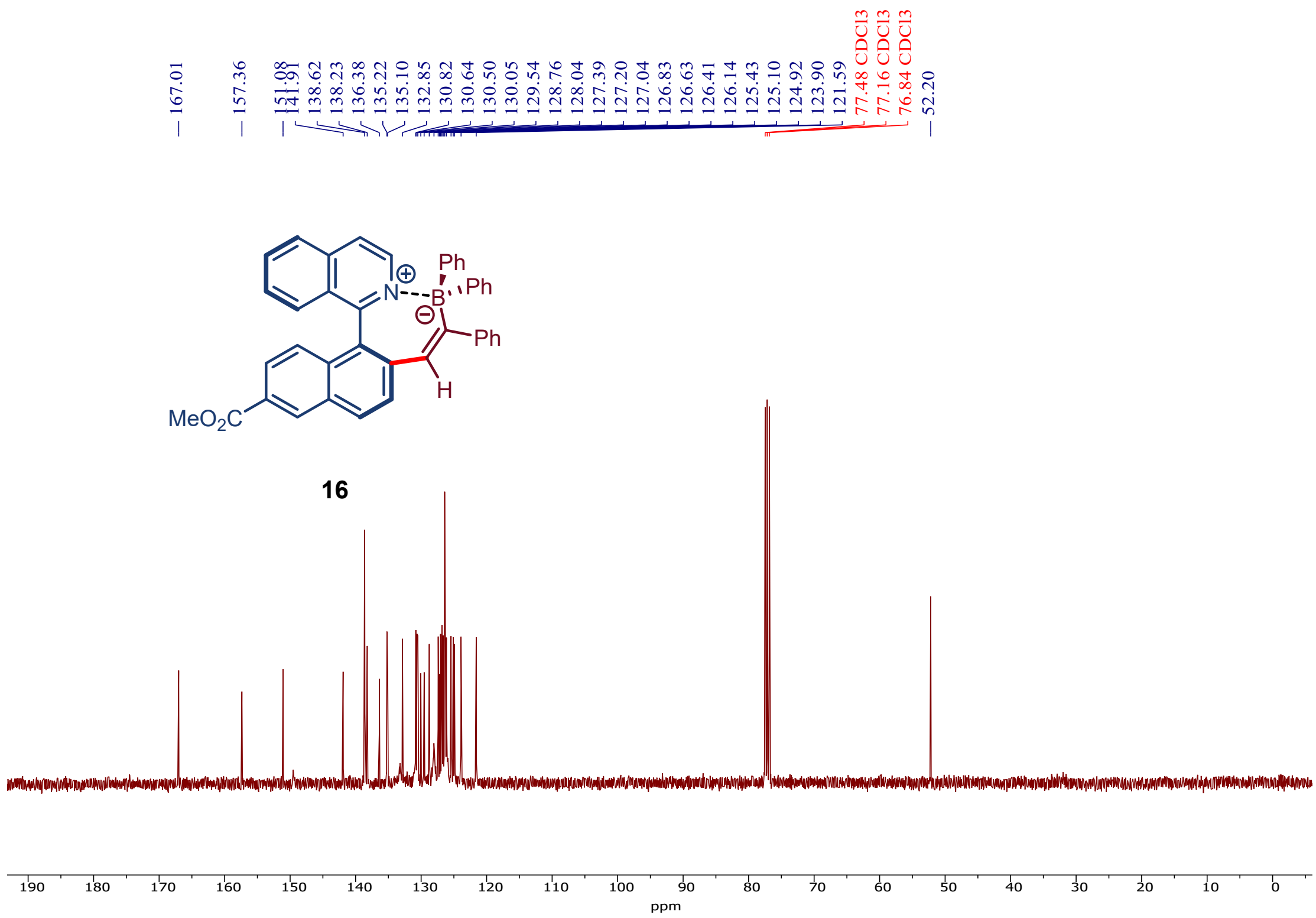

^{11}B NMR (128 MHz, $CDCl_3$) of **16**

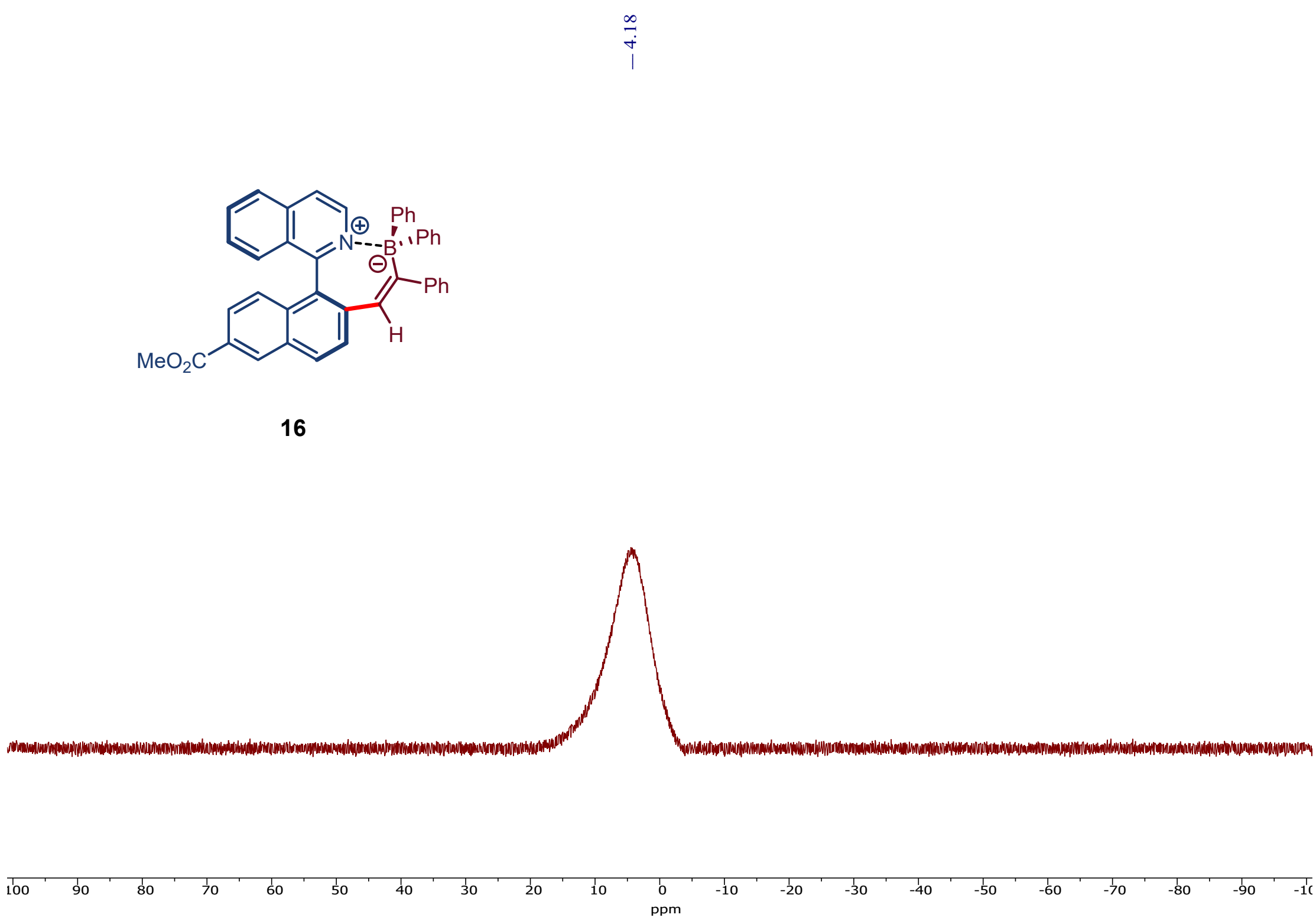

^{1}H NMR (400 MHz, $CDCl_3$) of **17** (*see procedure*)

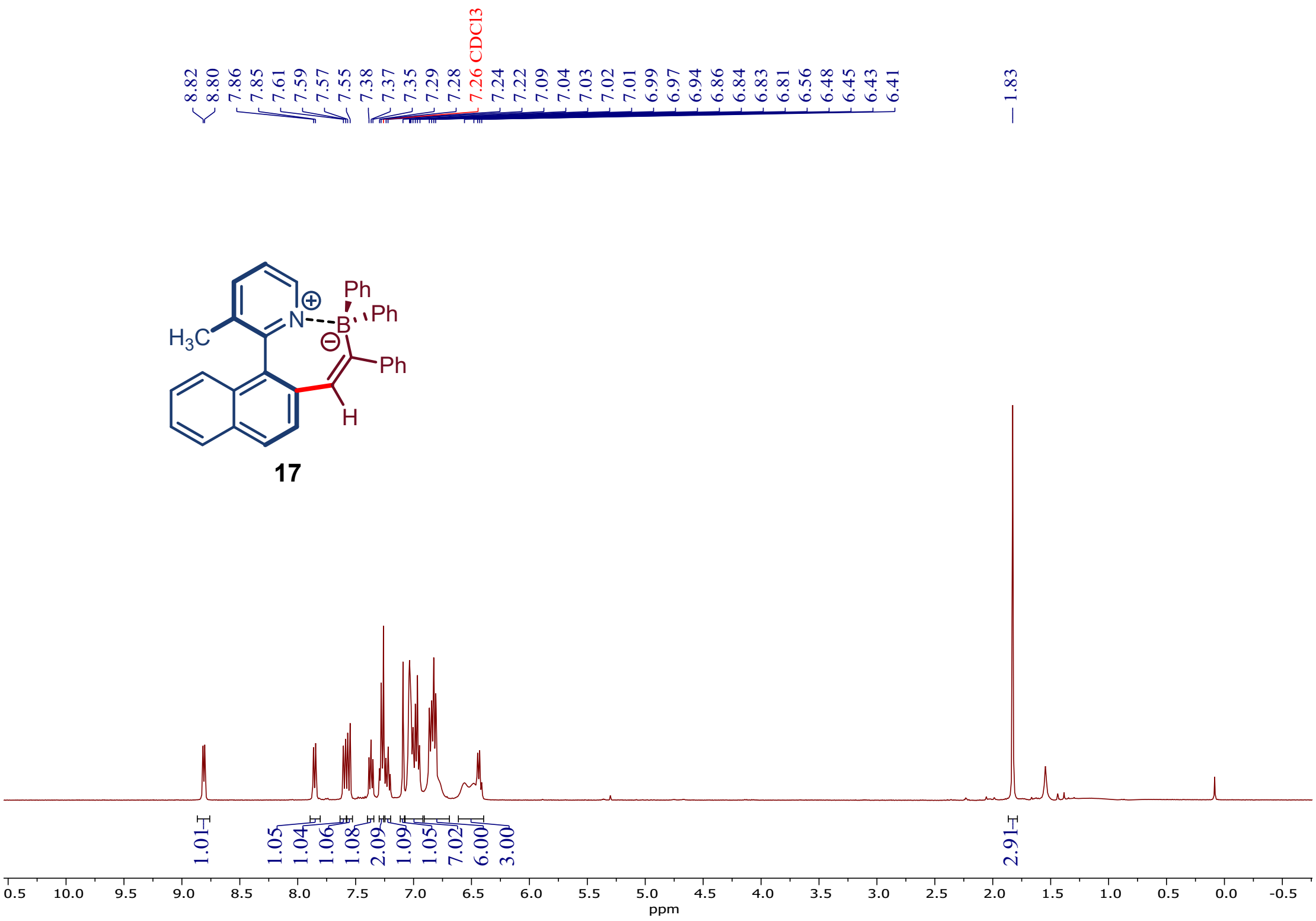

^{13}C NMR (100 MHz, $CDCl_3$) of **17**

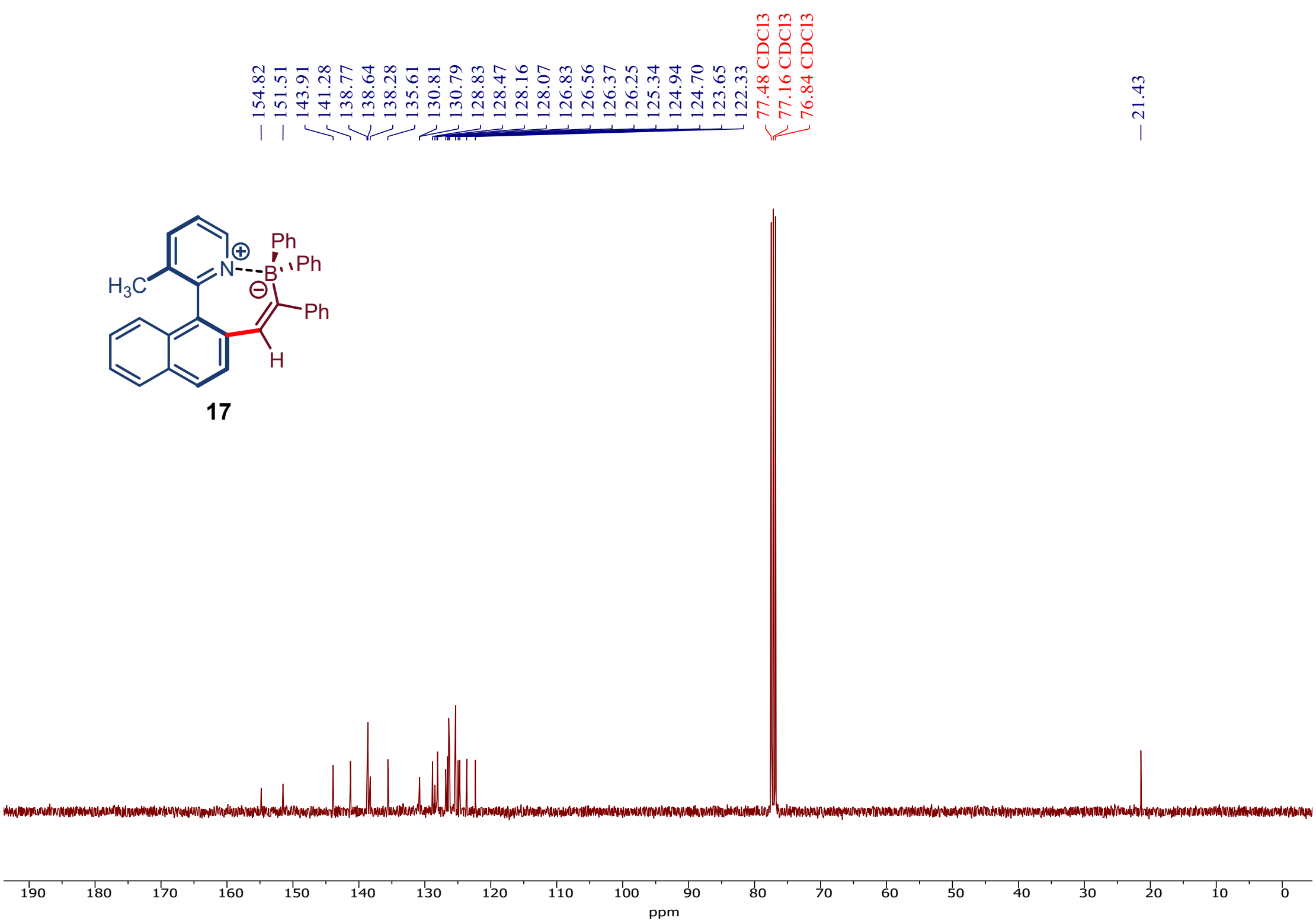

^{11}B NMR (128 MHz, $CDCl_3$) of **17**

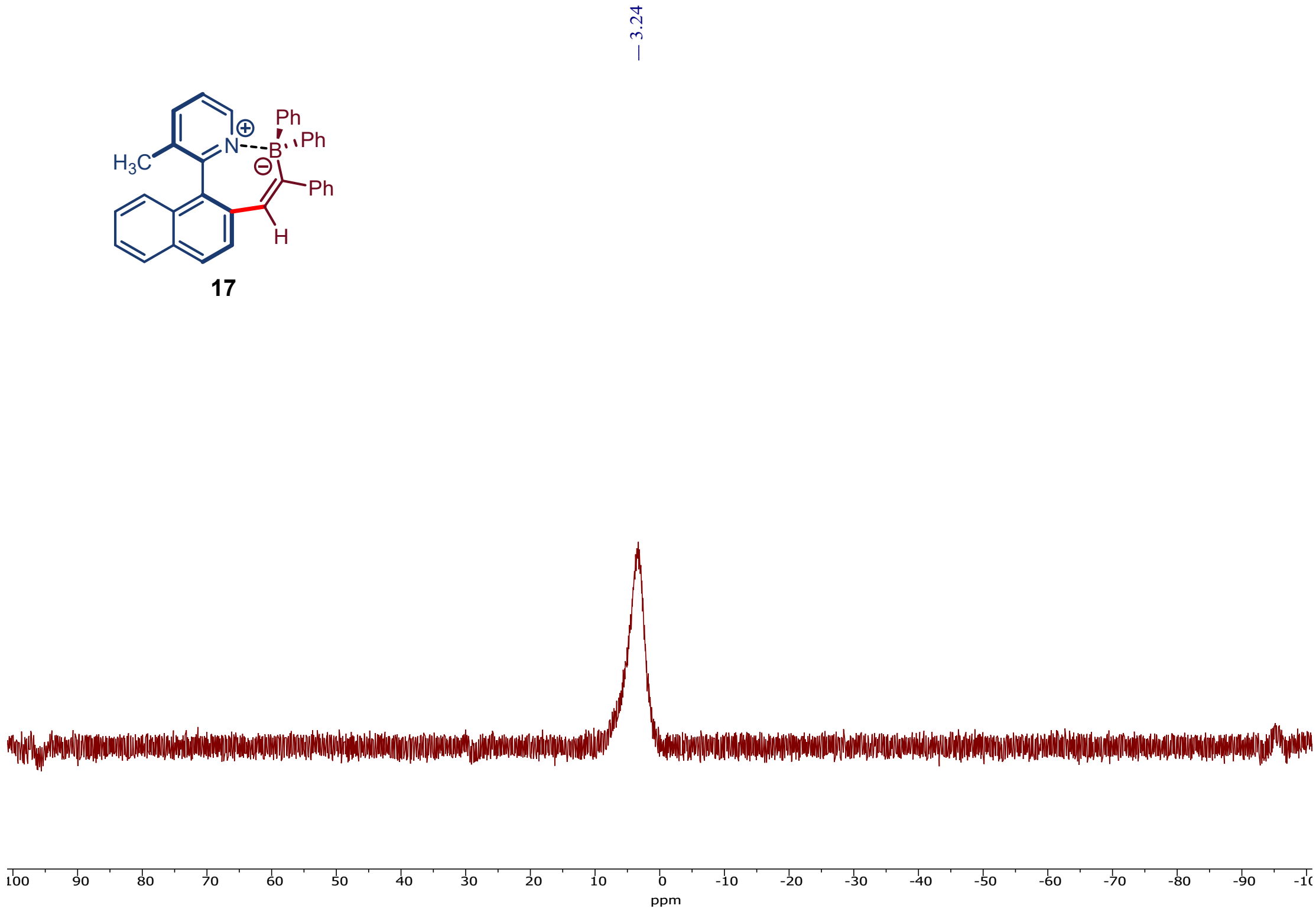

^{1}H NMR (500 MHz, $CDCl_3$) of **18** (*see procedure*)

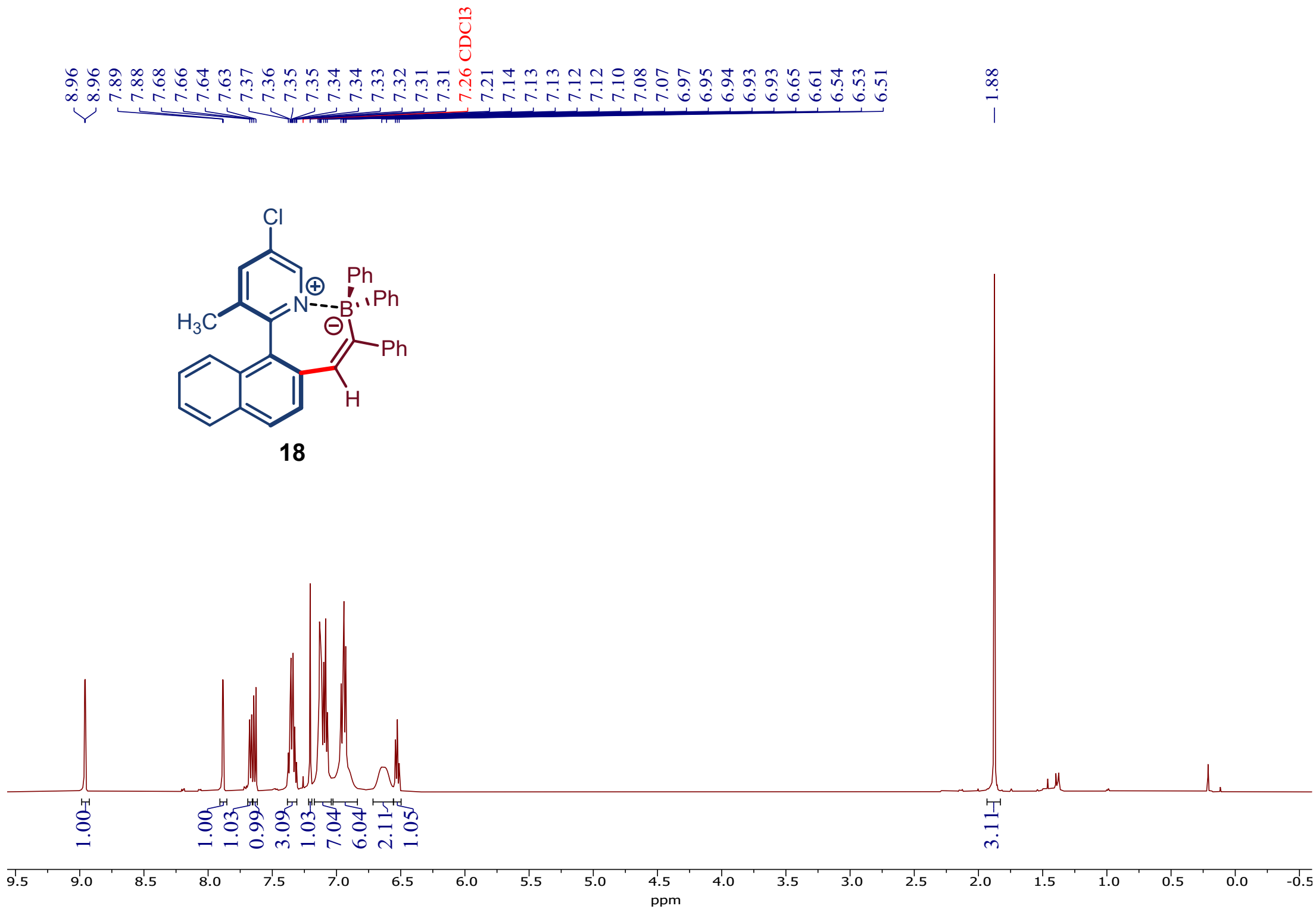

^{13}C NMR (126 MHz, $CDCl_3$) of **18**

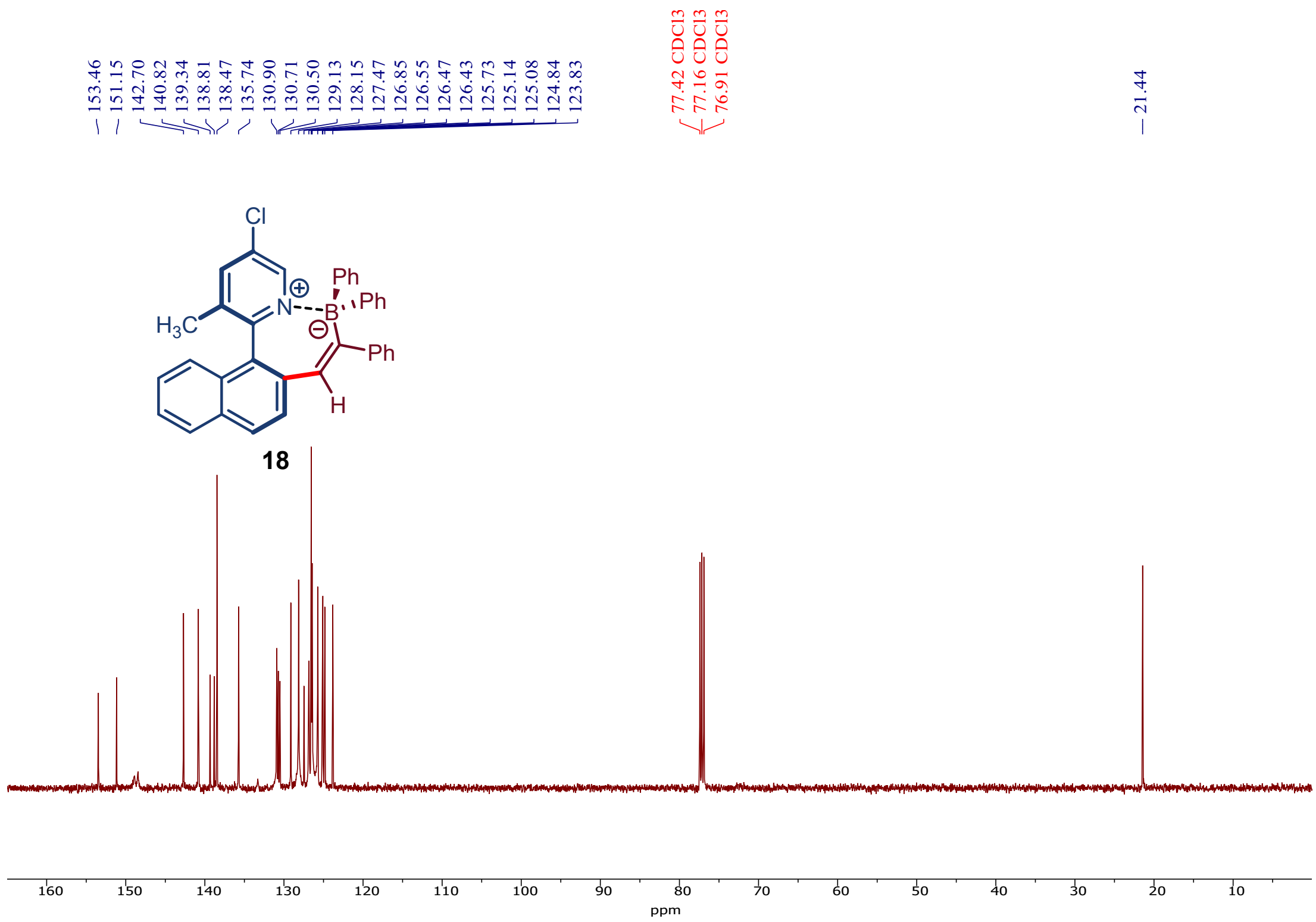

^{11}B NMR (160 MHz, $CDCl_3$) of **18**

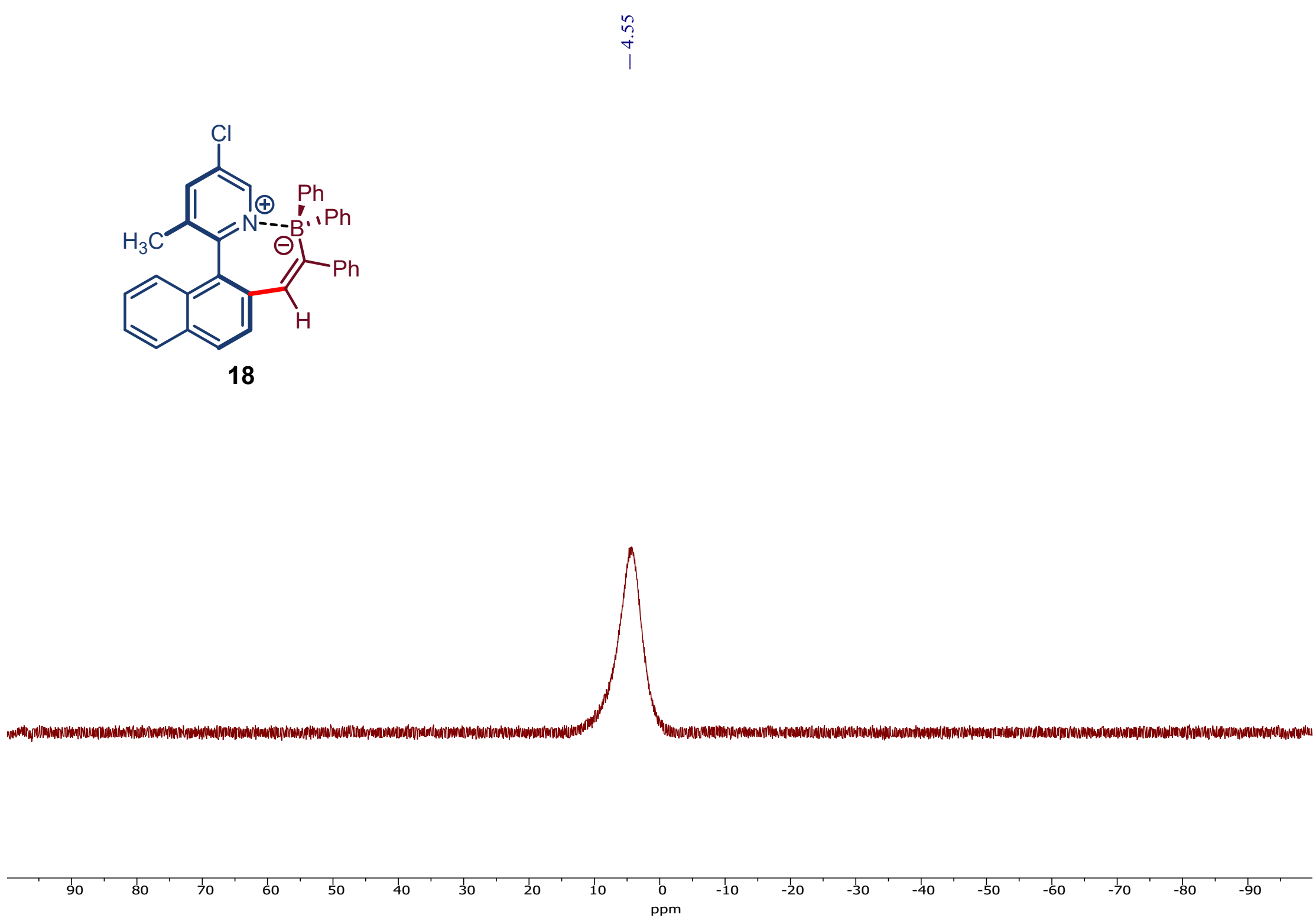

^{1}H NMR (400 MHz, $CDCl_3$) of **19** (*see procedure*)

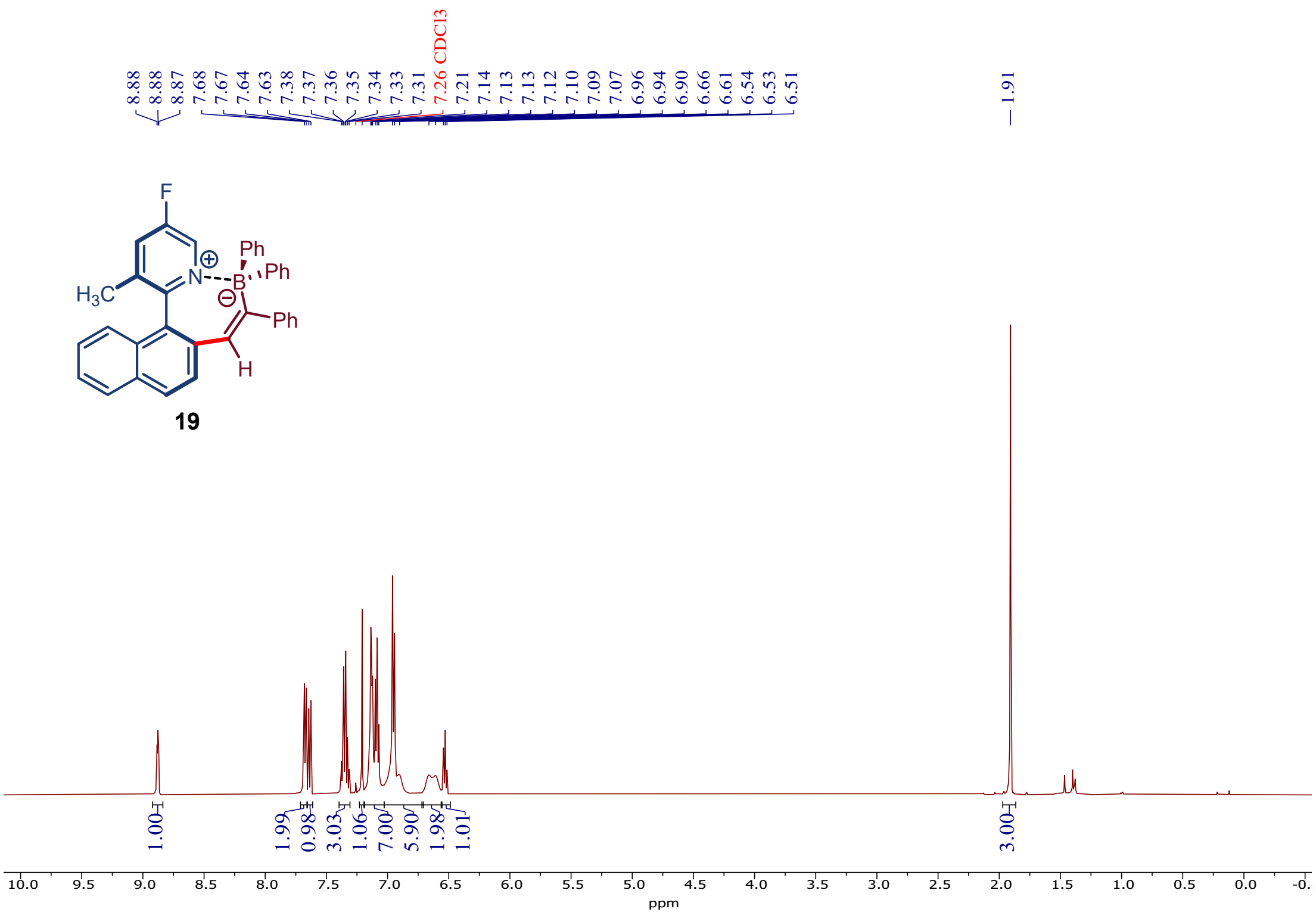

^{13}C NMR (100 MHz, $CDCl_3$) of **19**

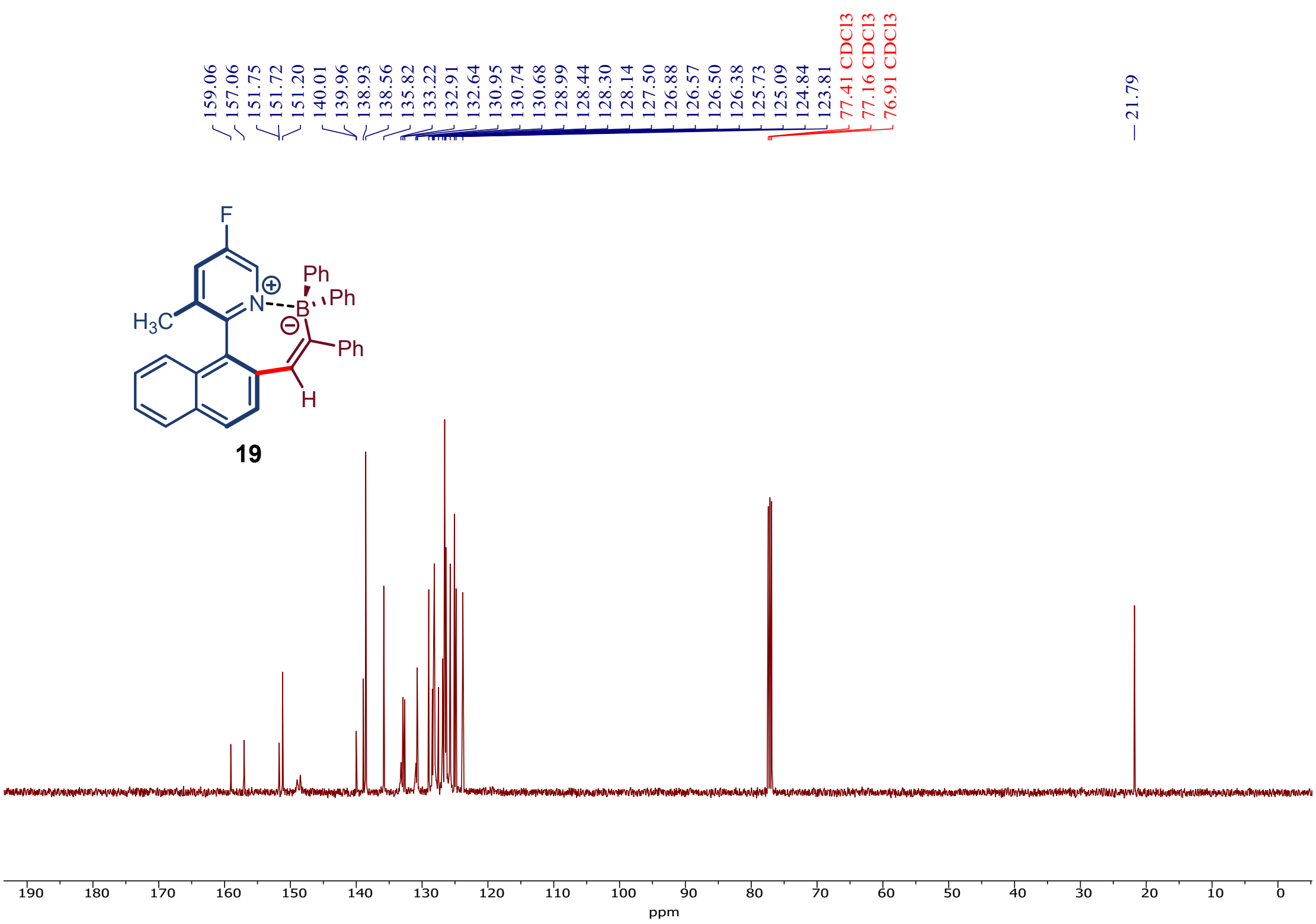

^{11}B NMR (128 MHz, $CDCl_3$) of **19**

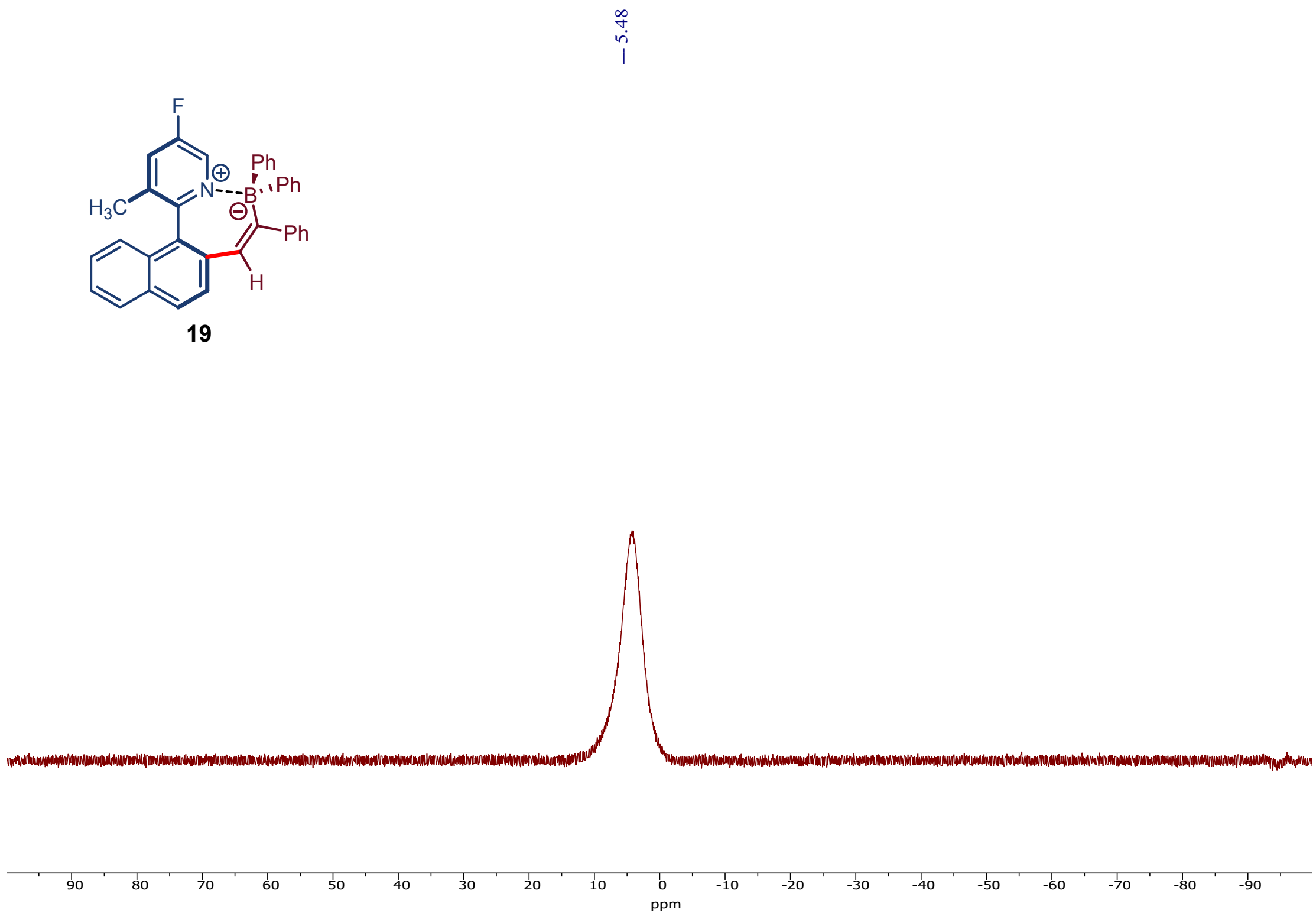

^{11}F NMR (471 MHz, $CDCl_3$) of **19**

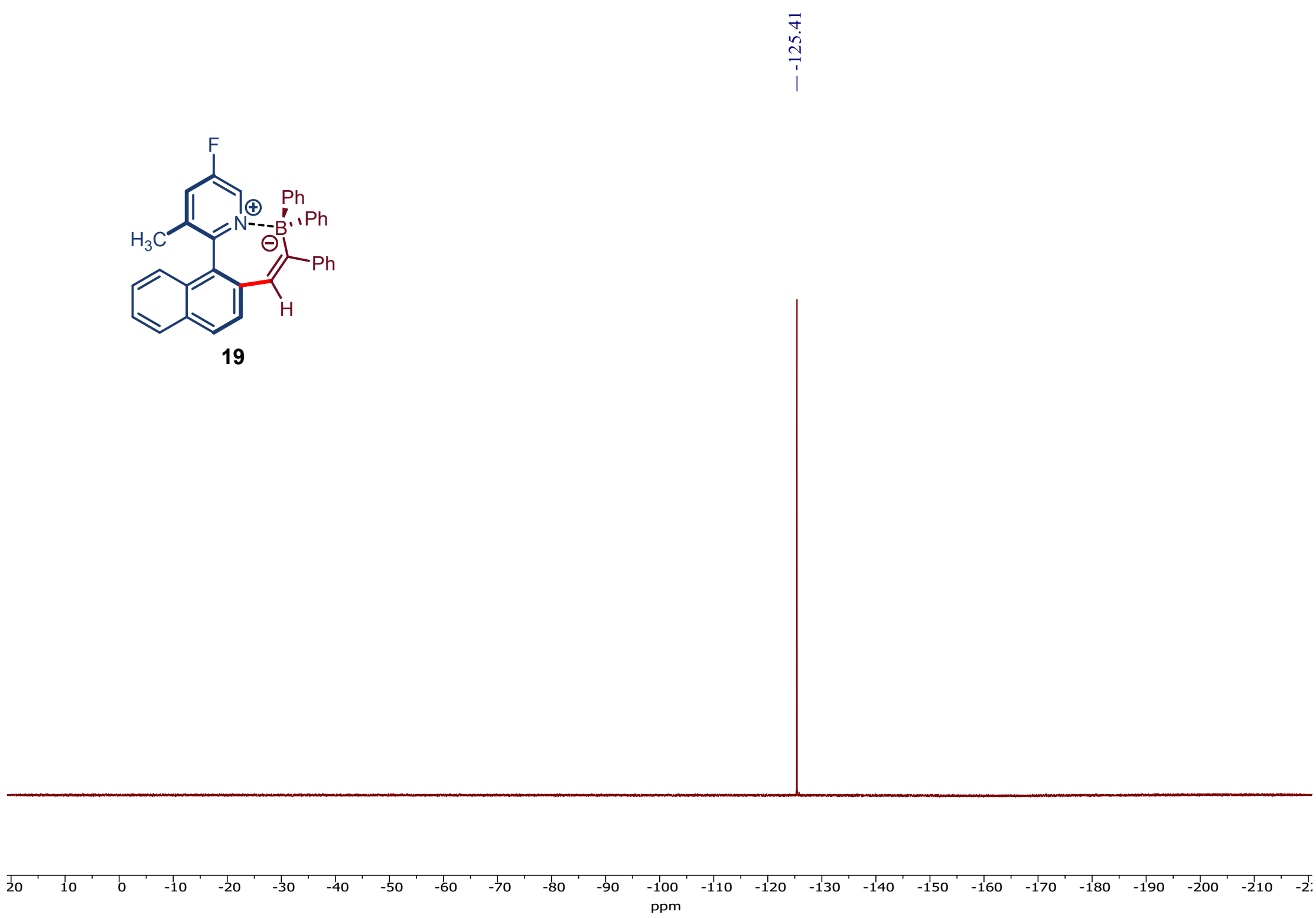

^{1}H NMR (400 MHz, $CDCl_3$) of **20** (*see procedure*)

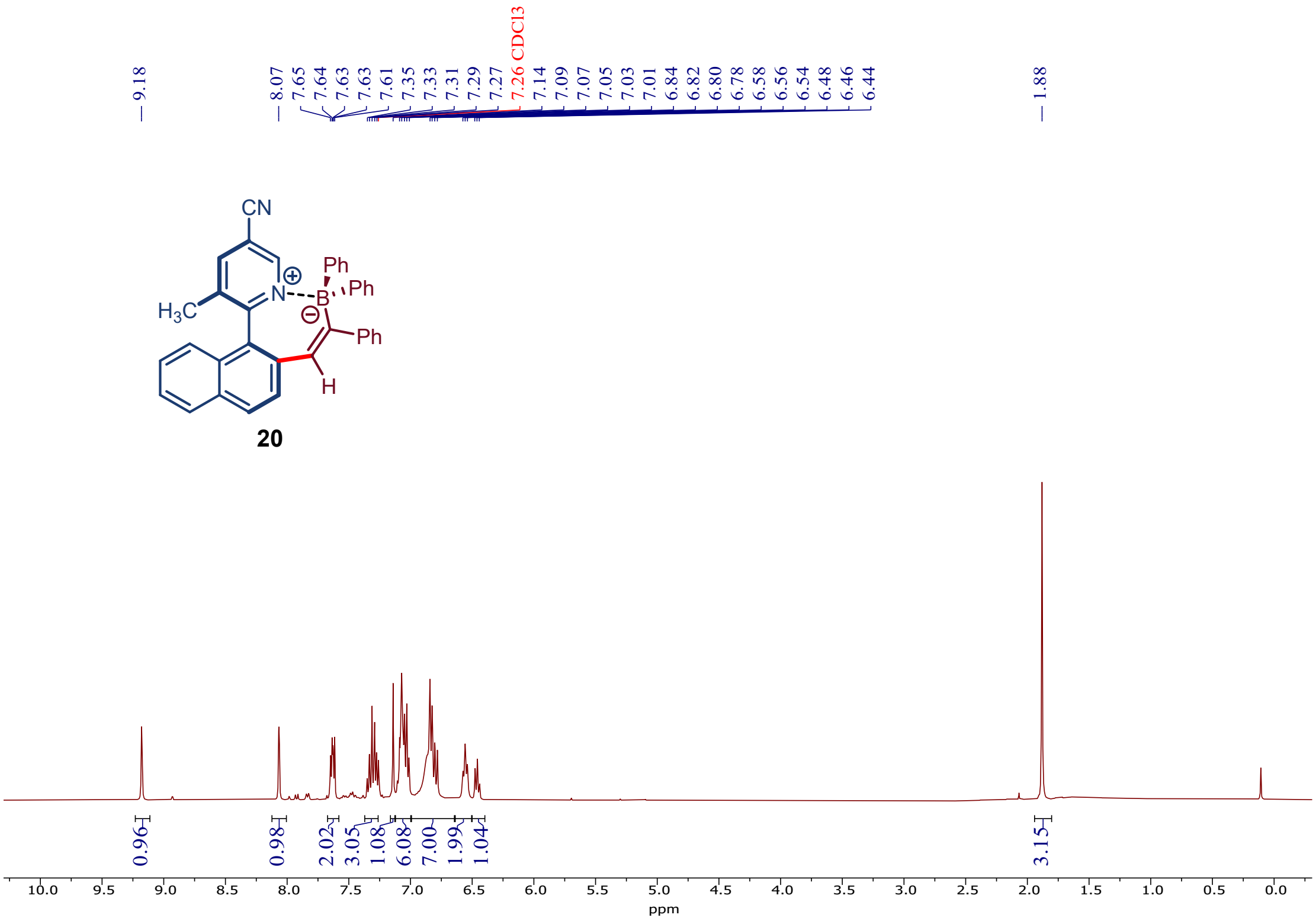

^{13}C NMR (100 MHz, $CDCl_3$) of **20**

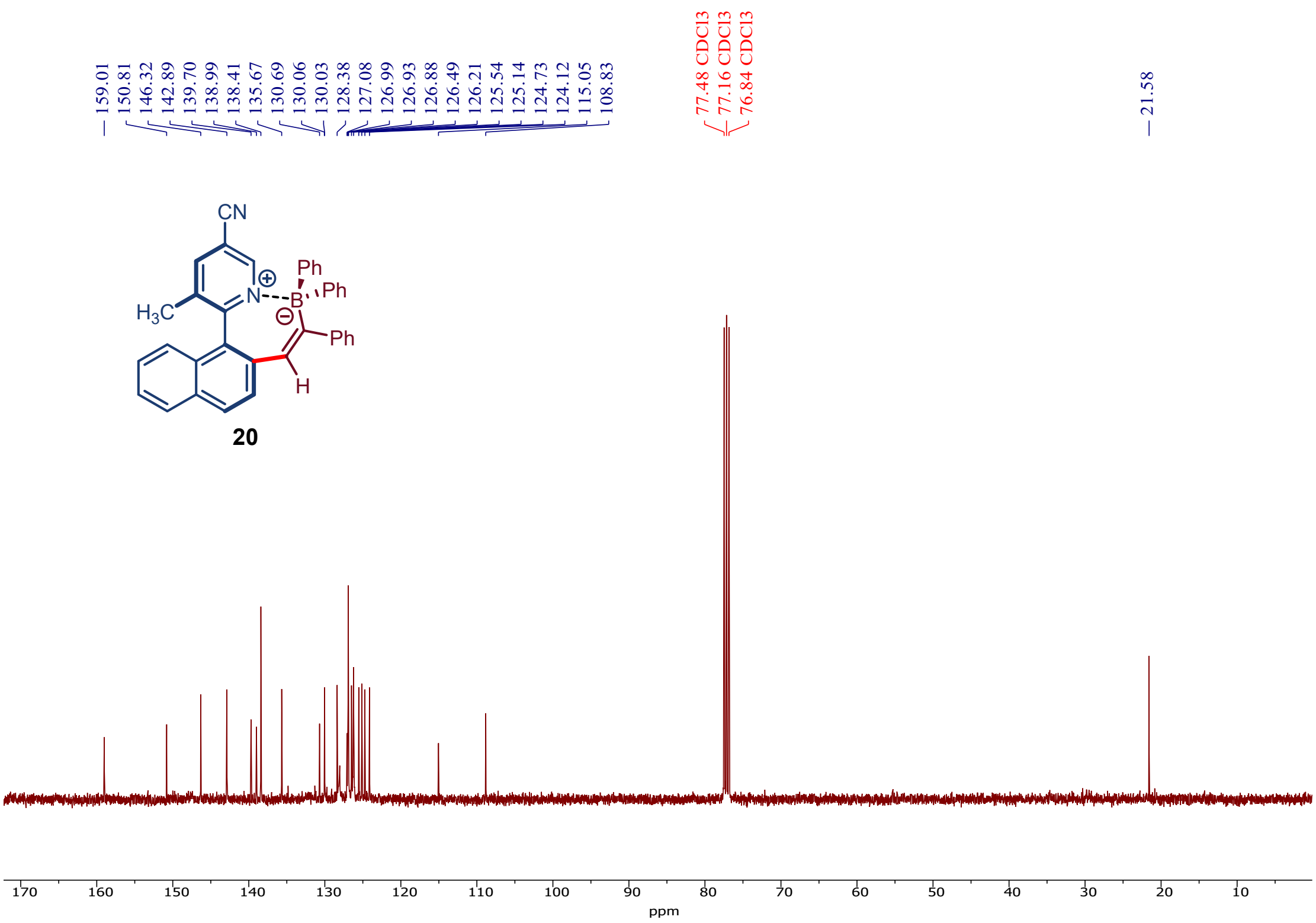

^{11}B NMR (128 MHz, $CDCl_3$) of **20**

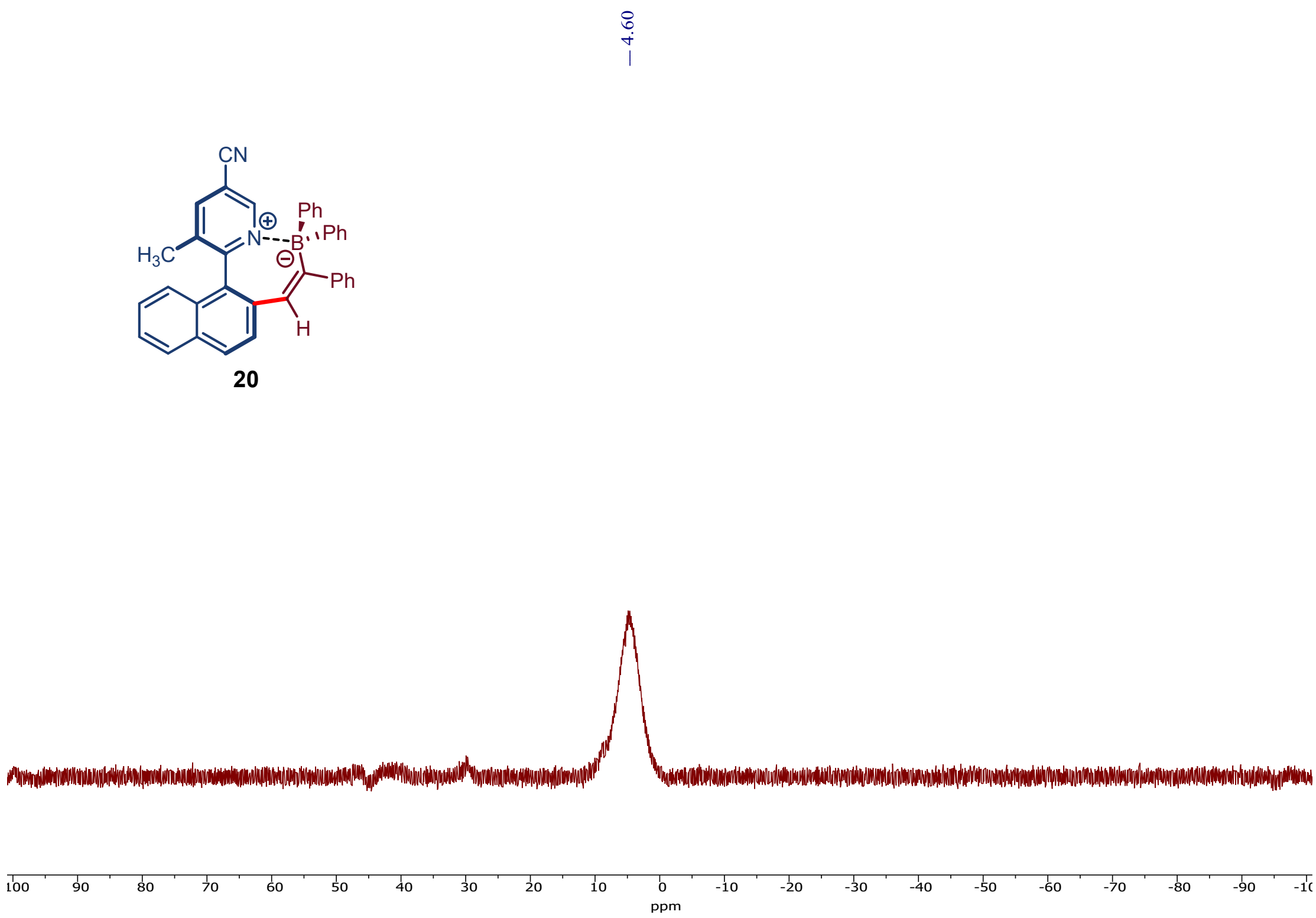

^{1}H NMR (500 MHz, $CDCl_3$) of **21** (*see procedure*)

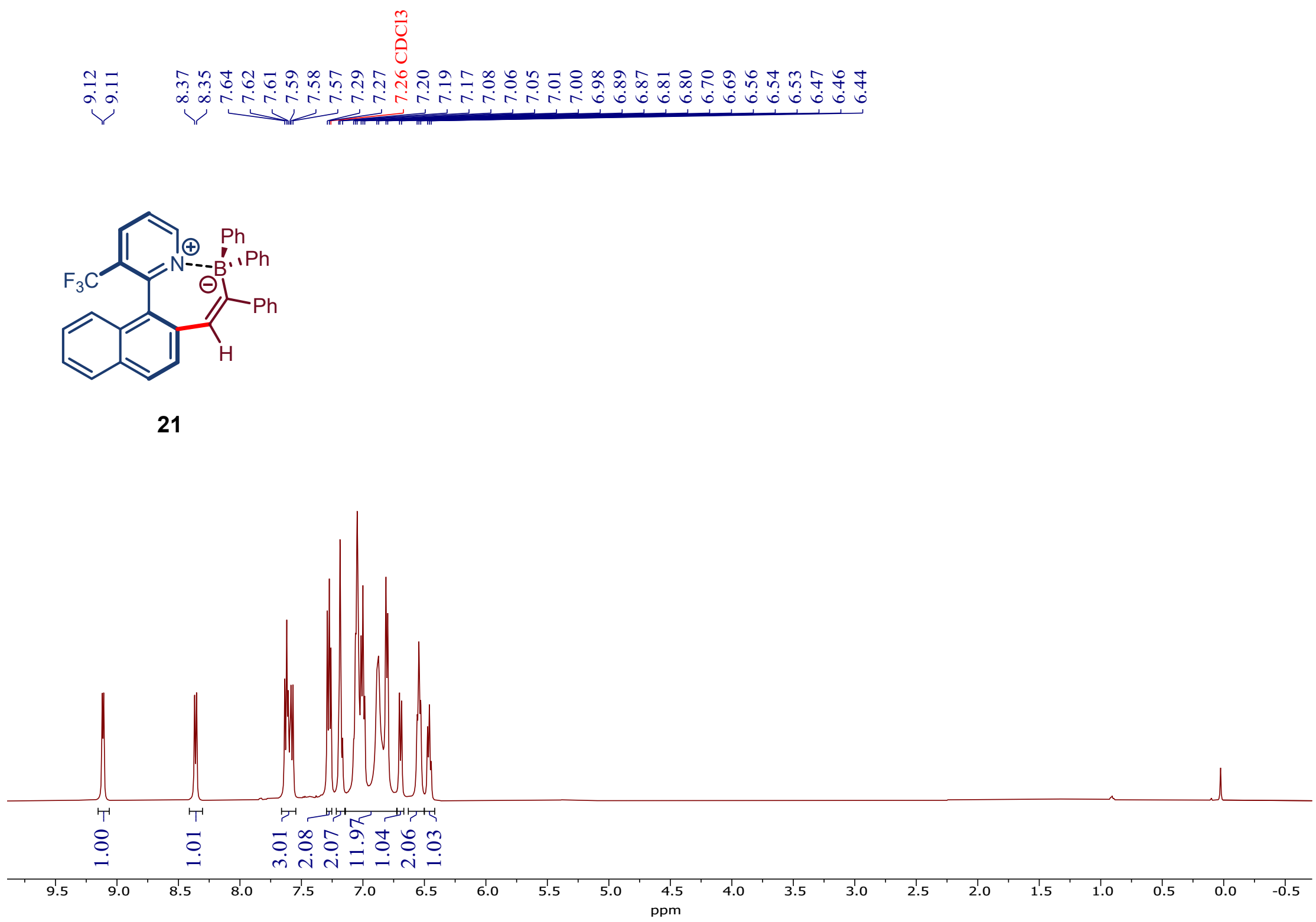

^{13}C NMR (100 MHz, $CDCl_3$) of **21**

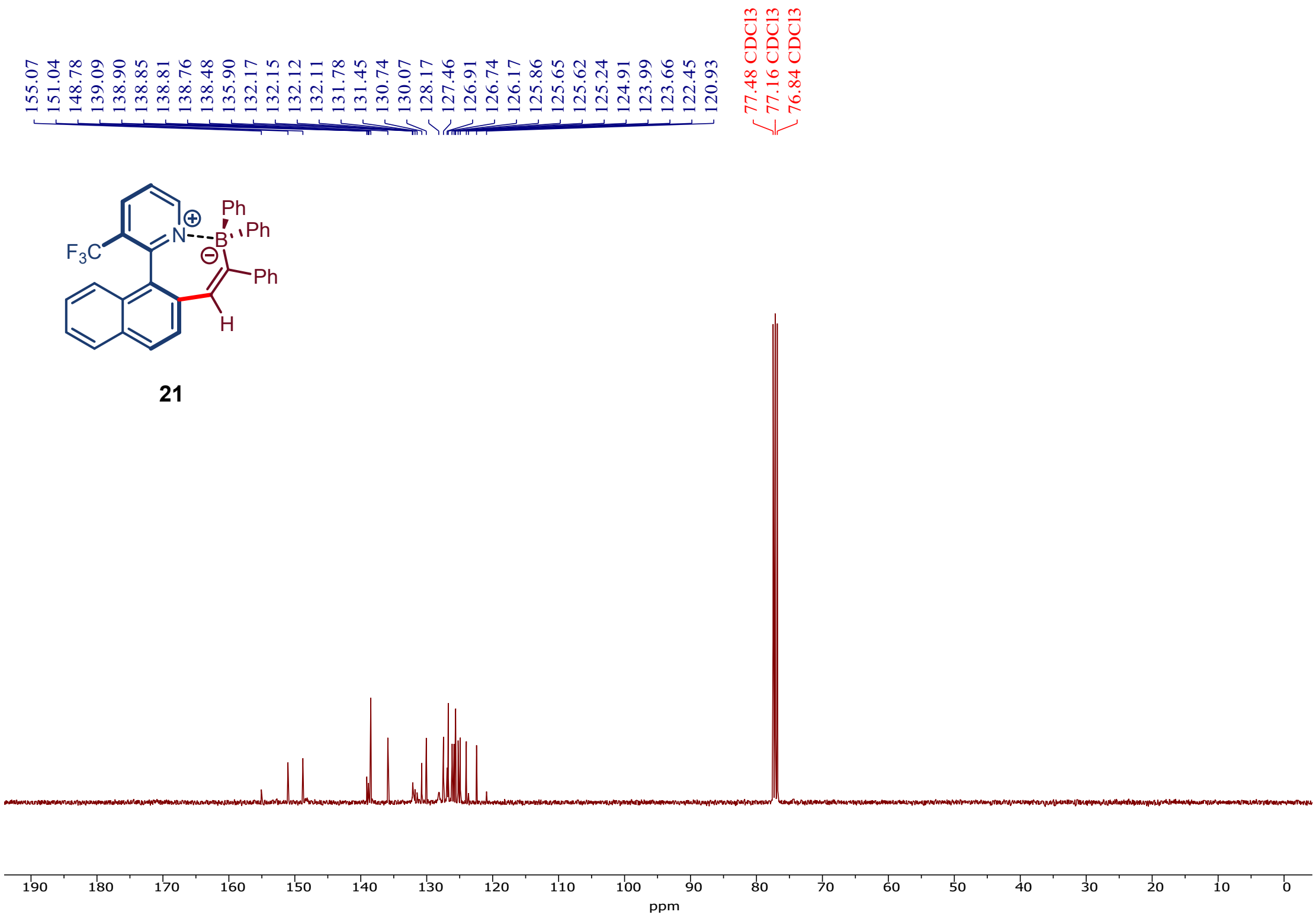

^{11}B NMR (128 MHz, $CDCl_3$) of **21**

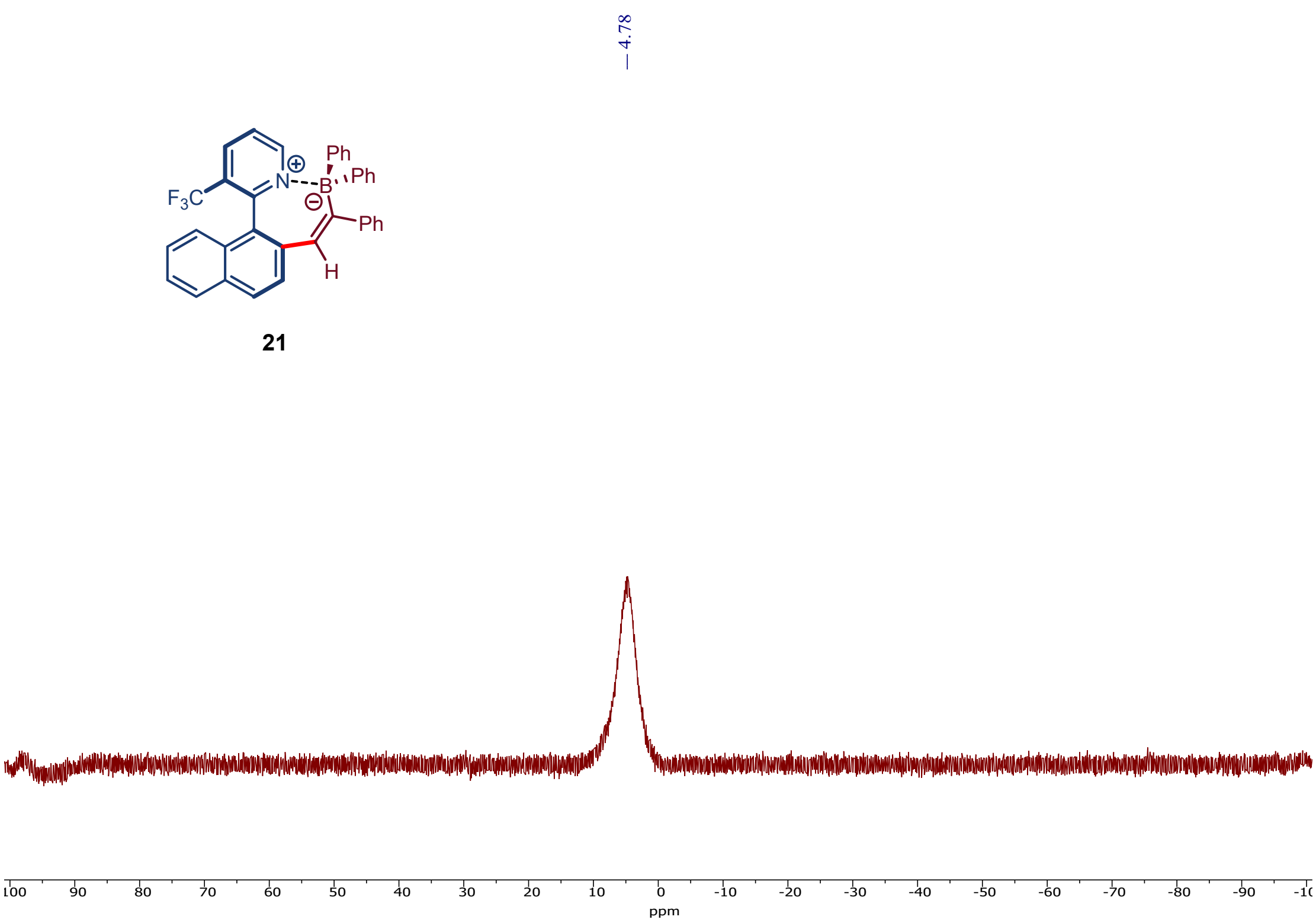

^{19}F NMR (471 MHz, $CDCl_3$) of **21**

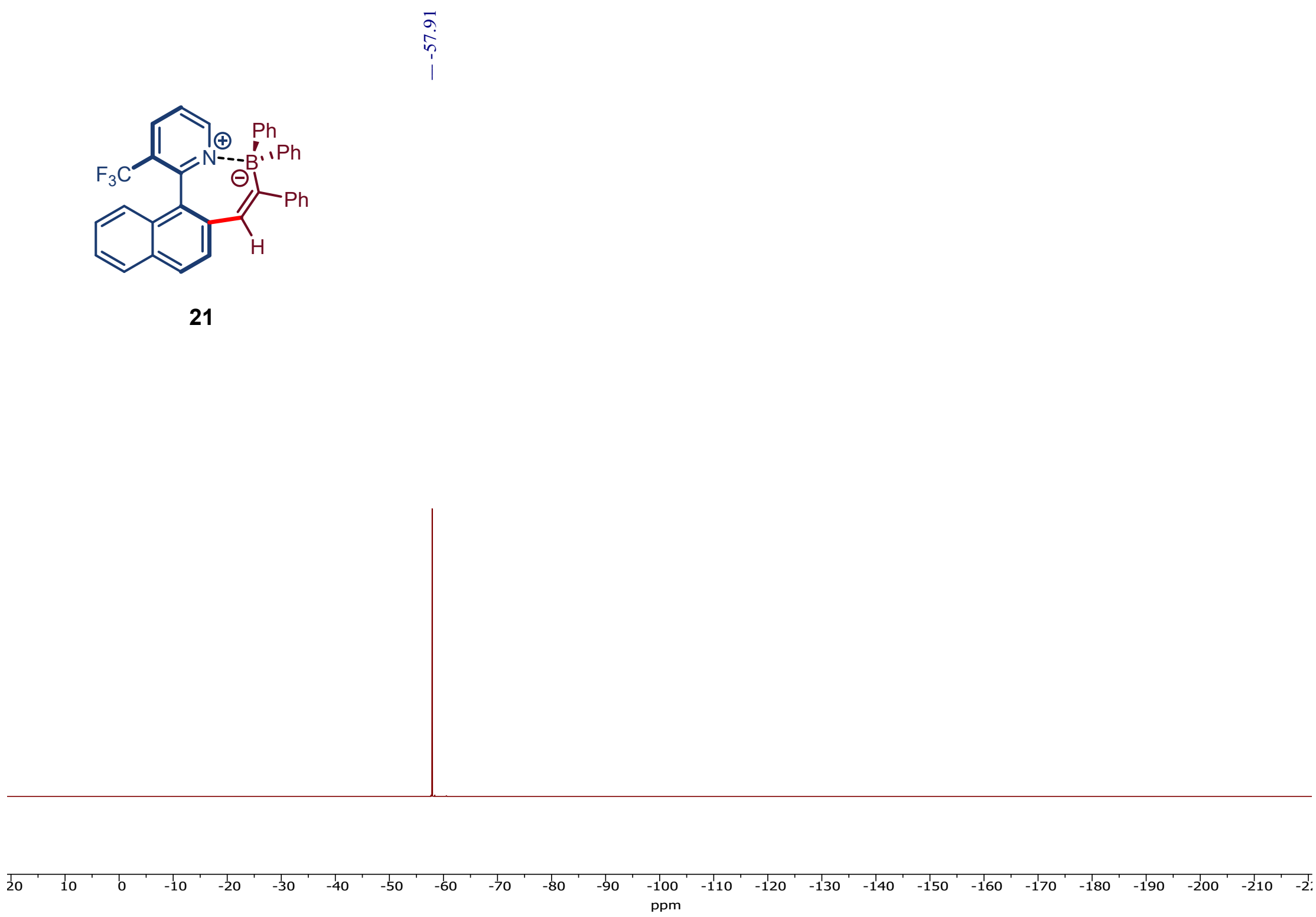

^{1}H NMR (400 MHz, $CDCl_3$) of **22** (*see procedure*)

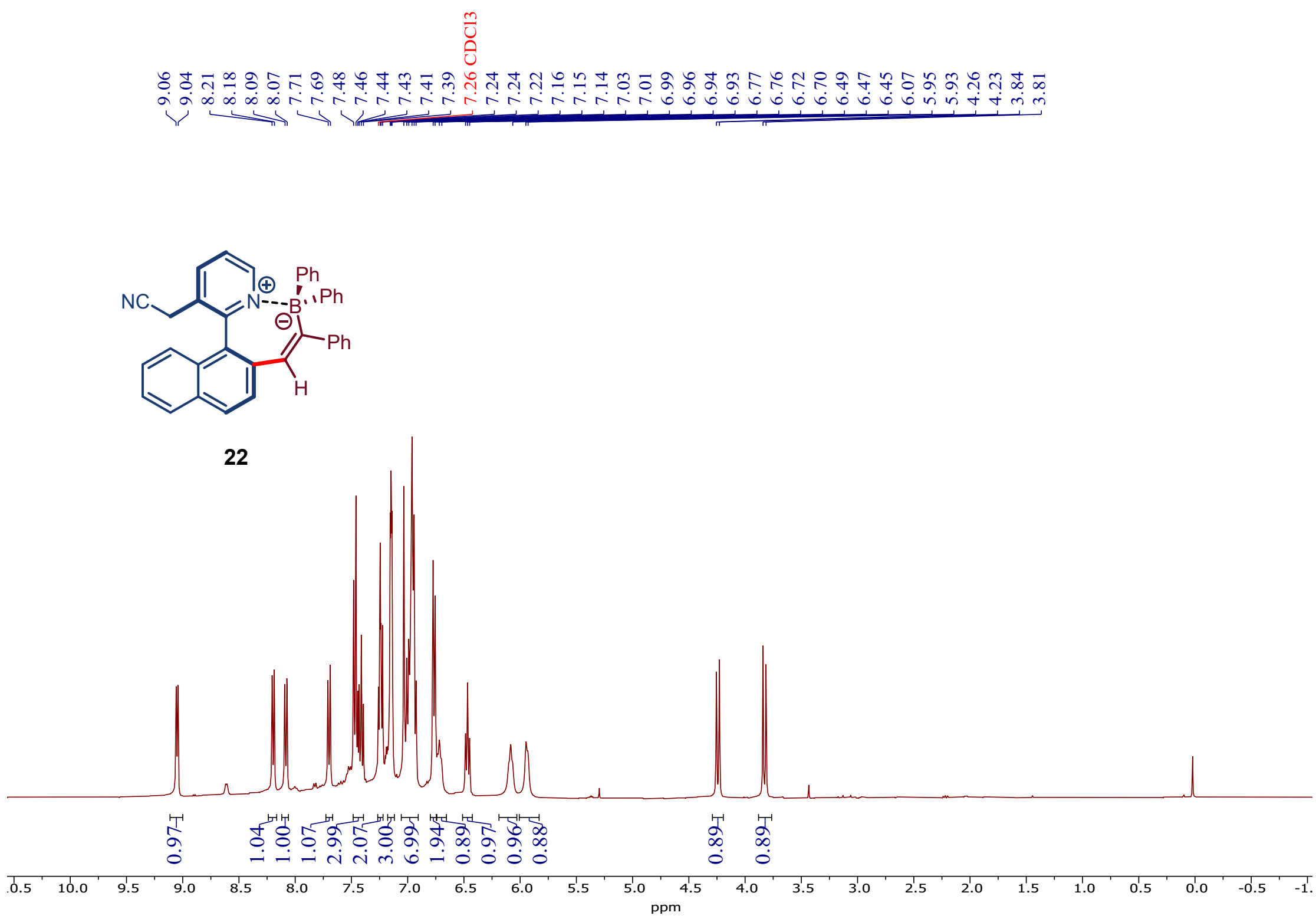

^{13}C NMR (100 MHz, $CDCl_3$) of **22**

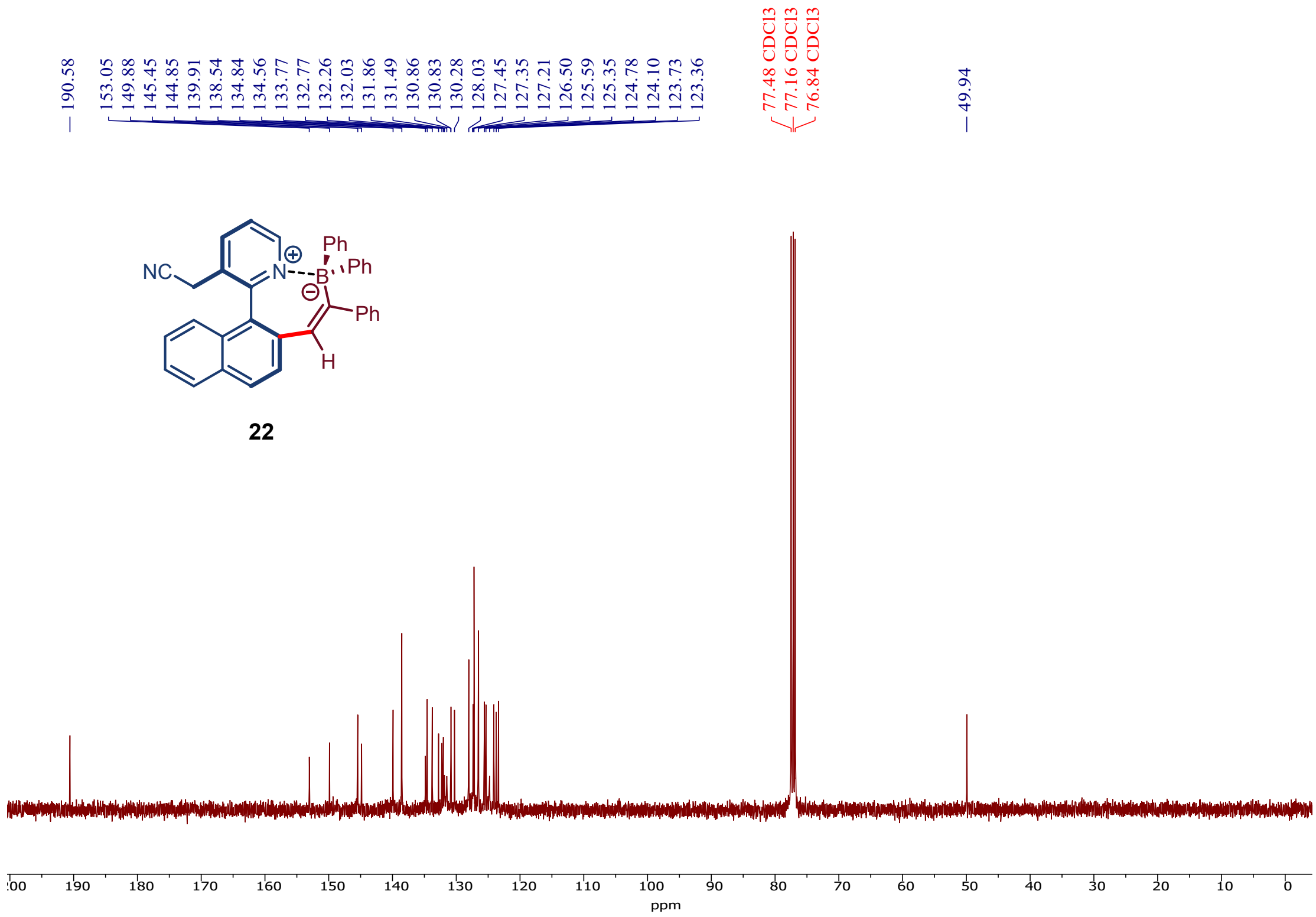

^{11}B NMR (128 MHz, $CDCl_3$) of **22**

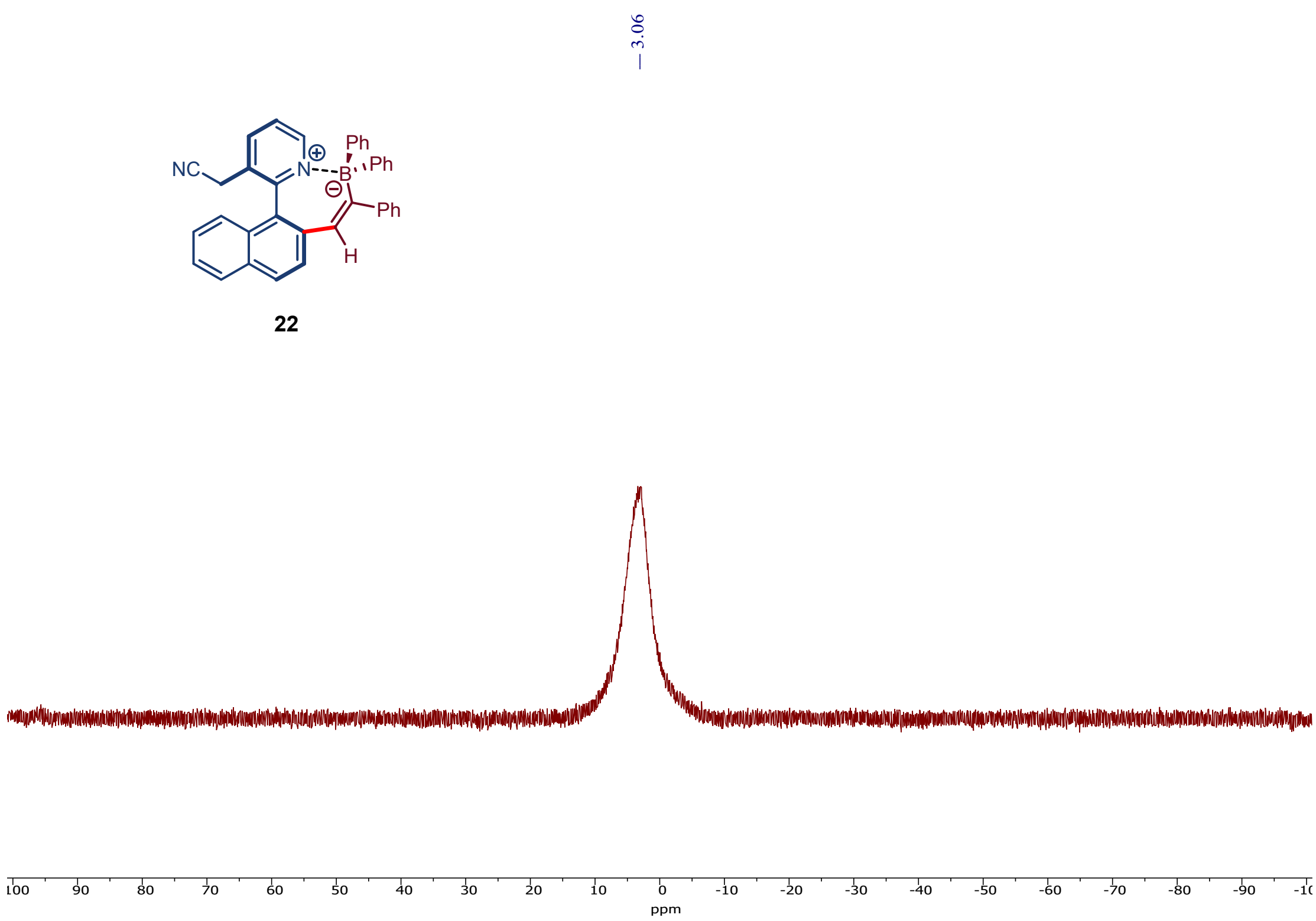

^{1}H NMR (400 MHz, $CDCl_3$) of **23** (*see procedure*)

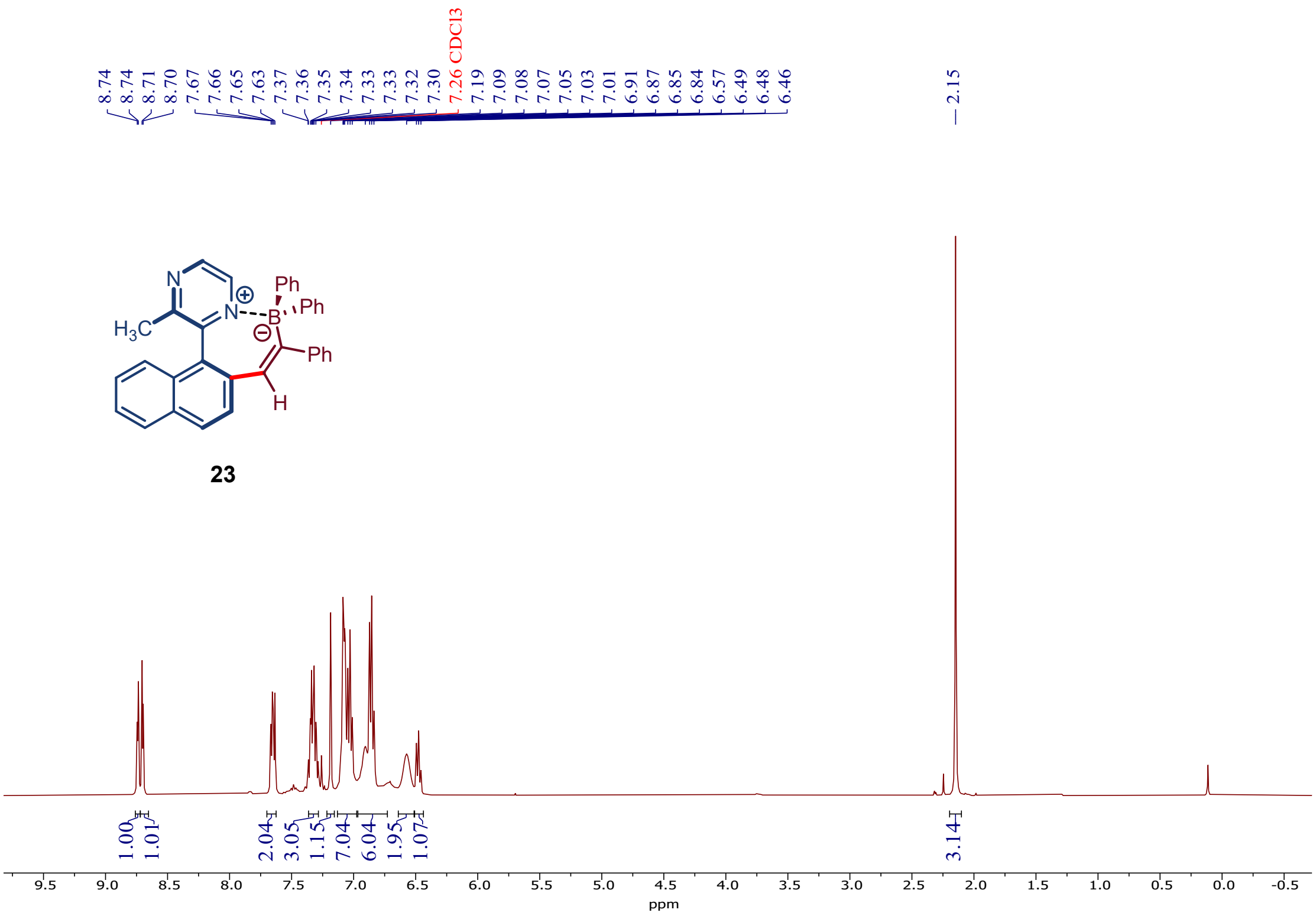

^{13}C NMR (100 MHz, $CDCl_3$) of **23**

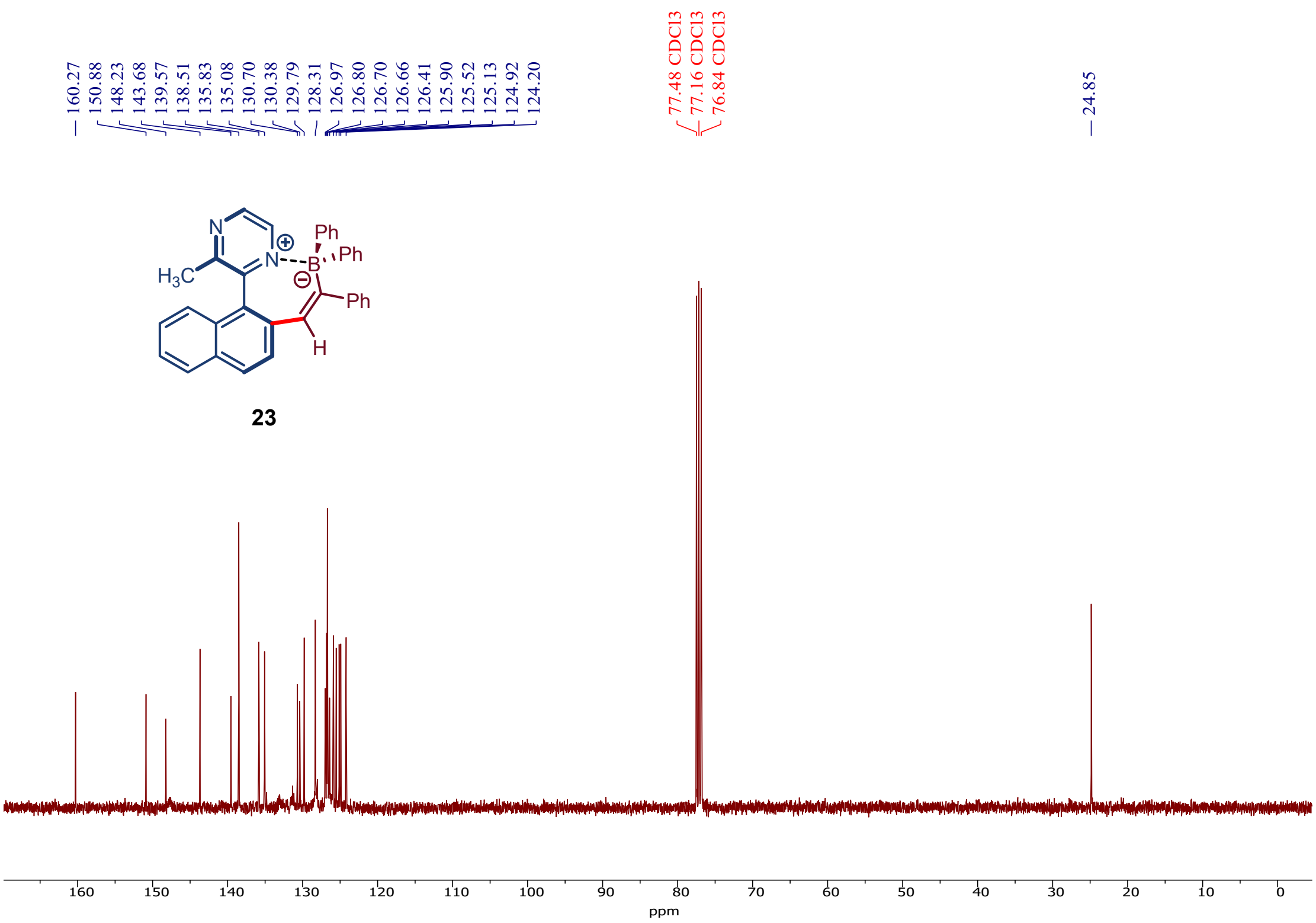

^{11}B NMR (128 MHz, $CDCl_3$) of **23**

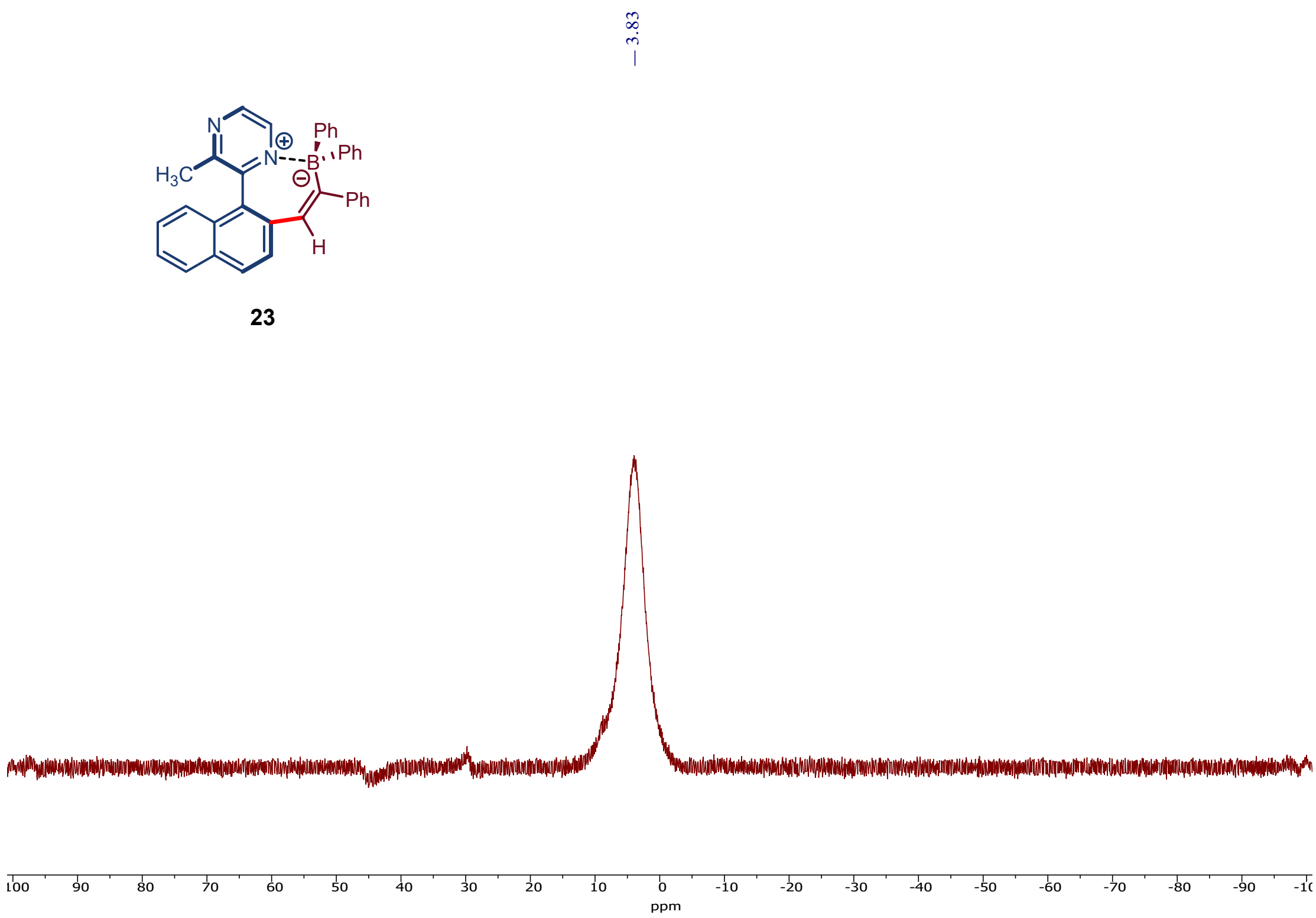

^{1}H NMR (400 MHz, $CDCl_3$) of **24** (*see procedure*)

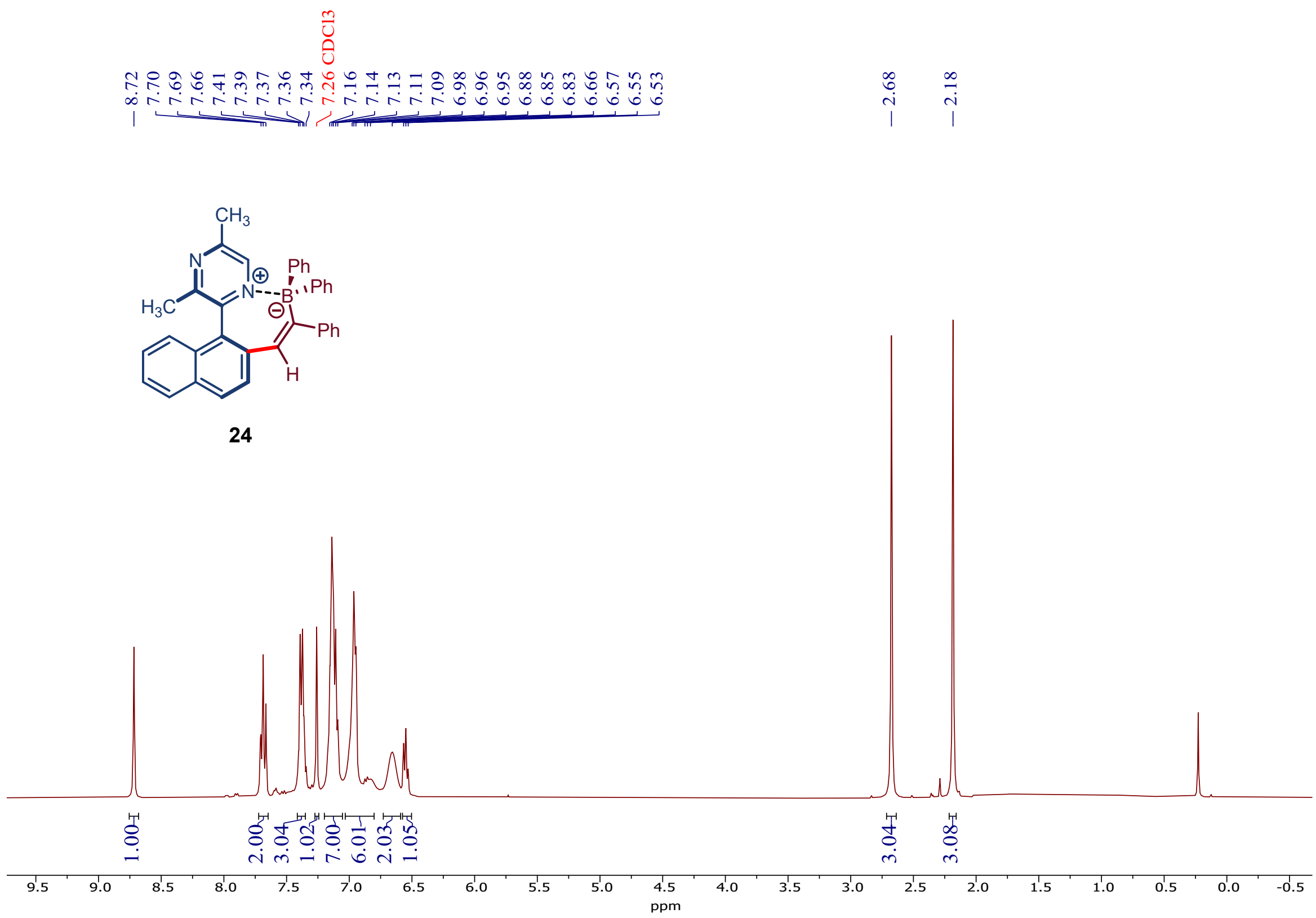

^{13}C NMR (100 MHz, $CDCl_3$) of **24**

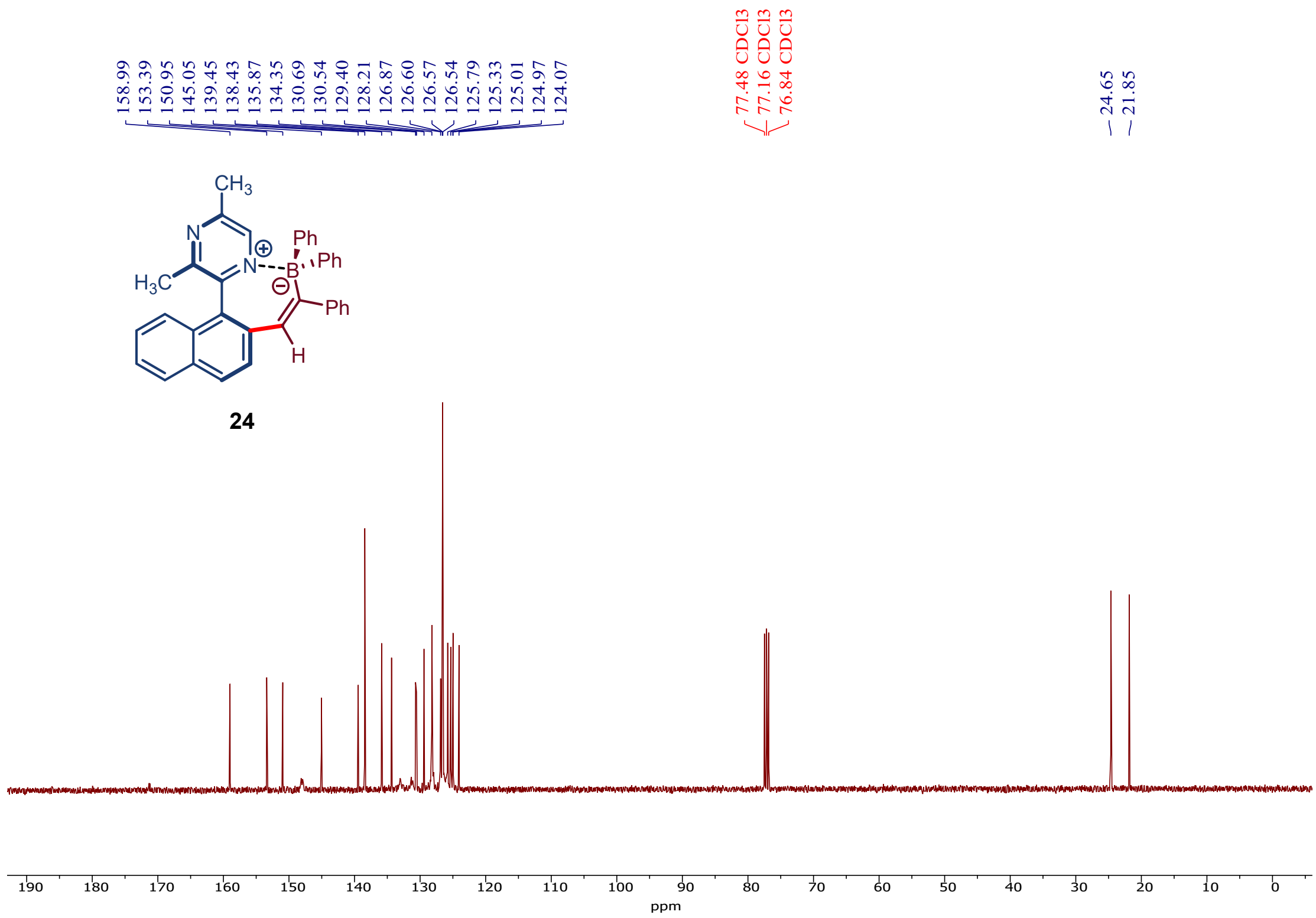

^{11}B NMR (128 MHz, $CDCl_3$) of **24**

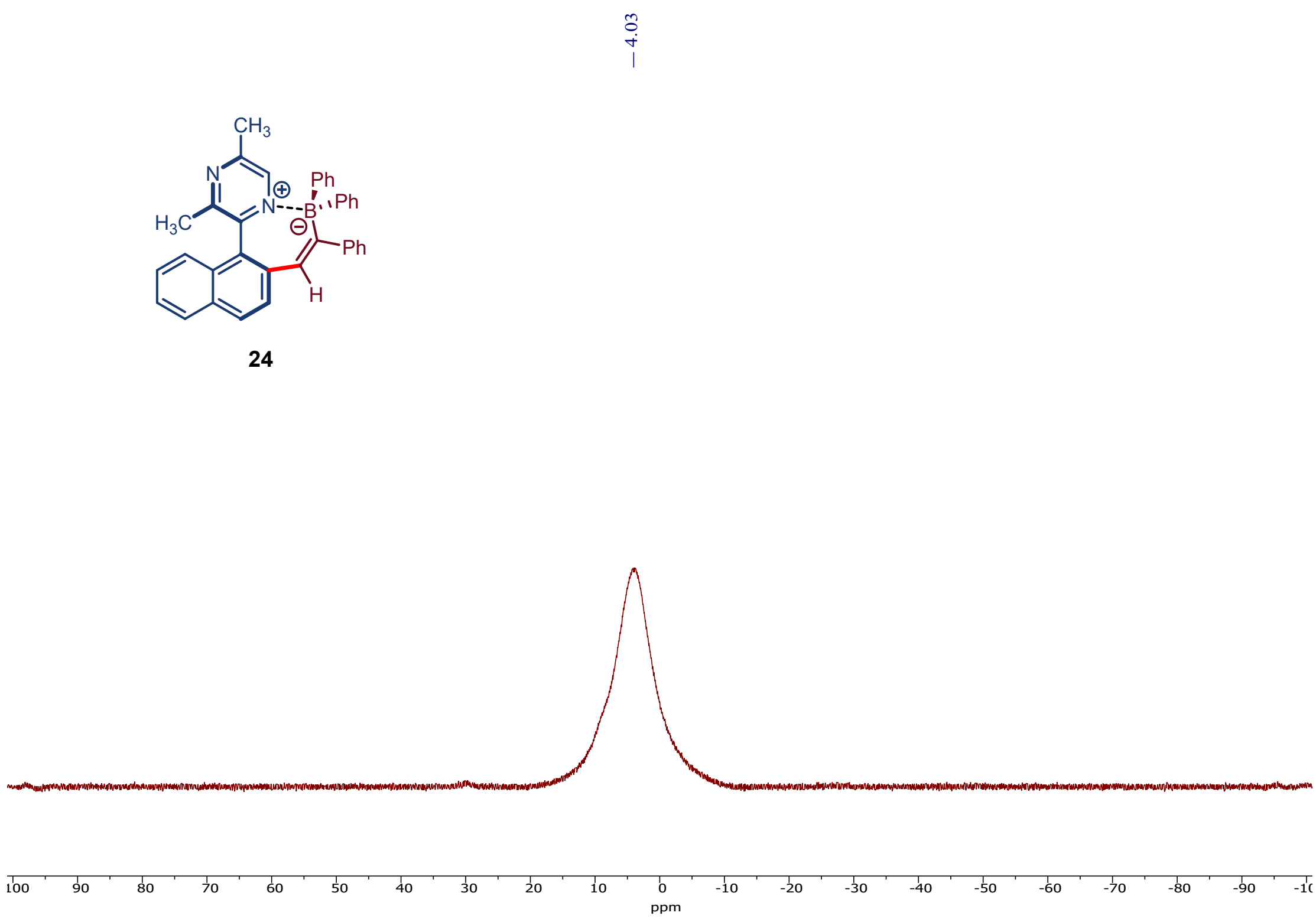

^{1}H NMR (400 MHz, $CDCl_3$) of **25** (*see procedure*)

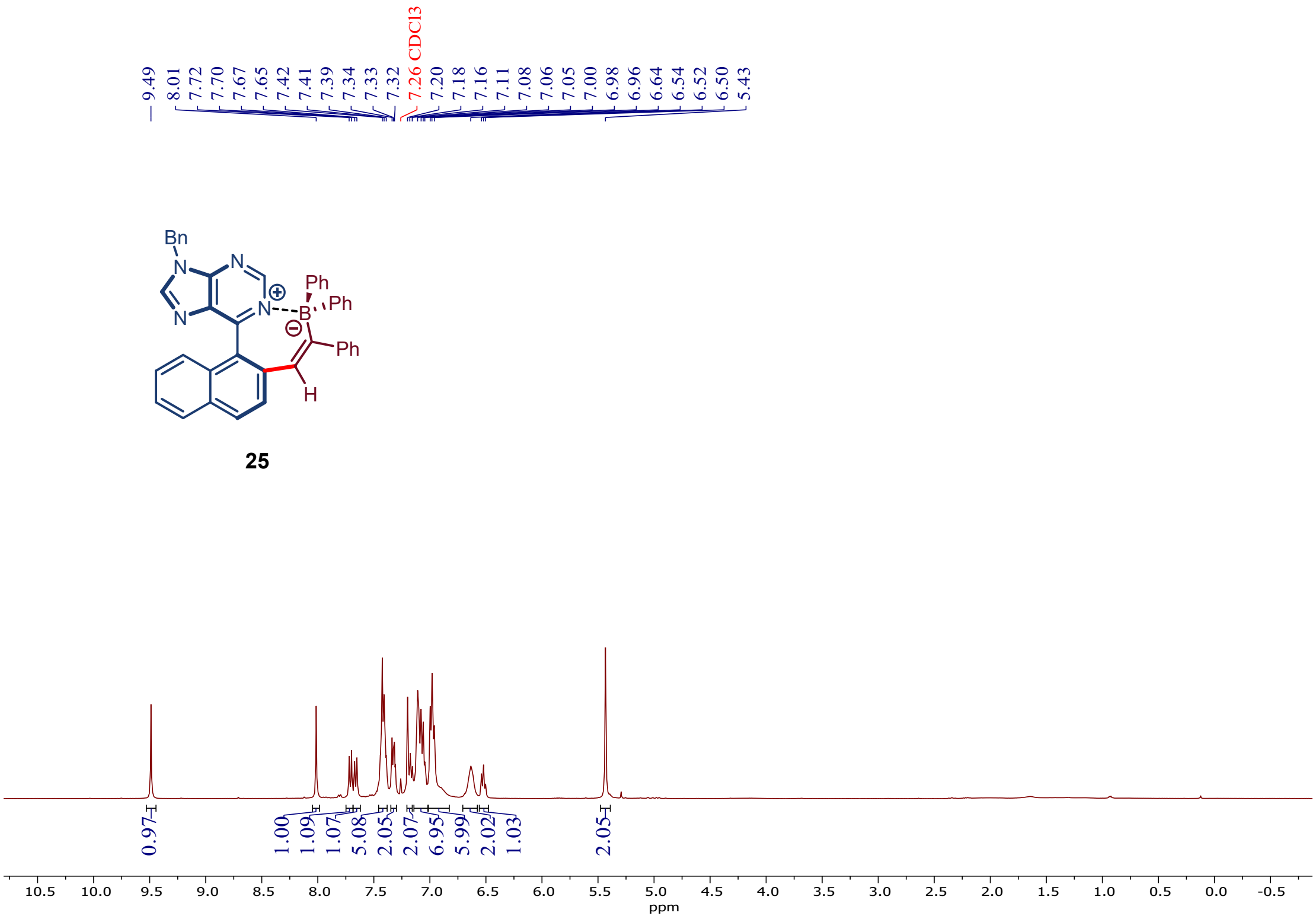

^{13}C NMR (100 MHz, $CDCl_3$) of **25**

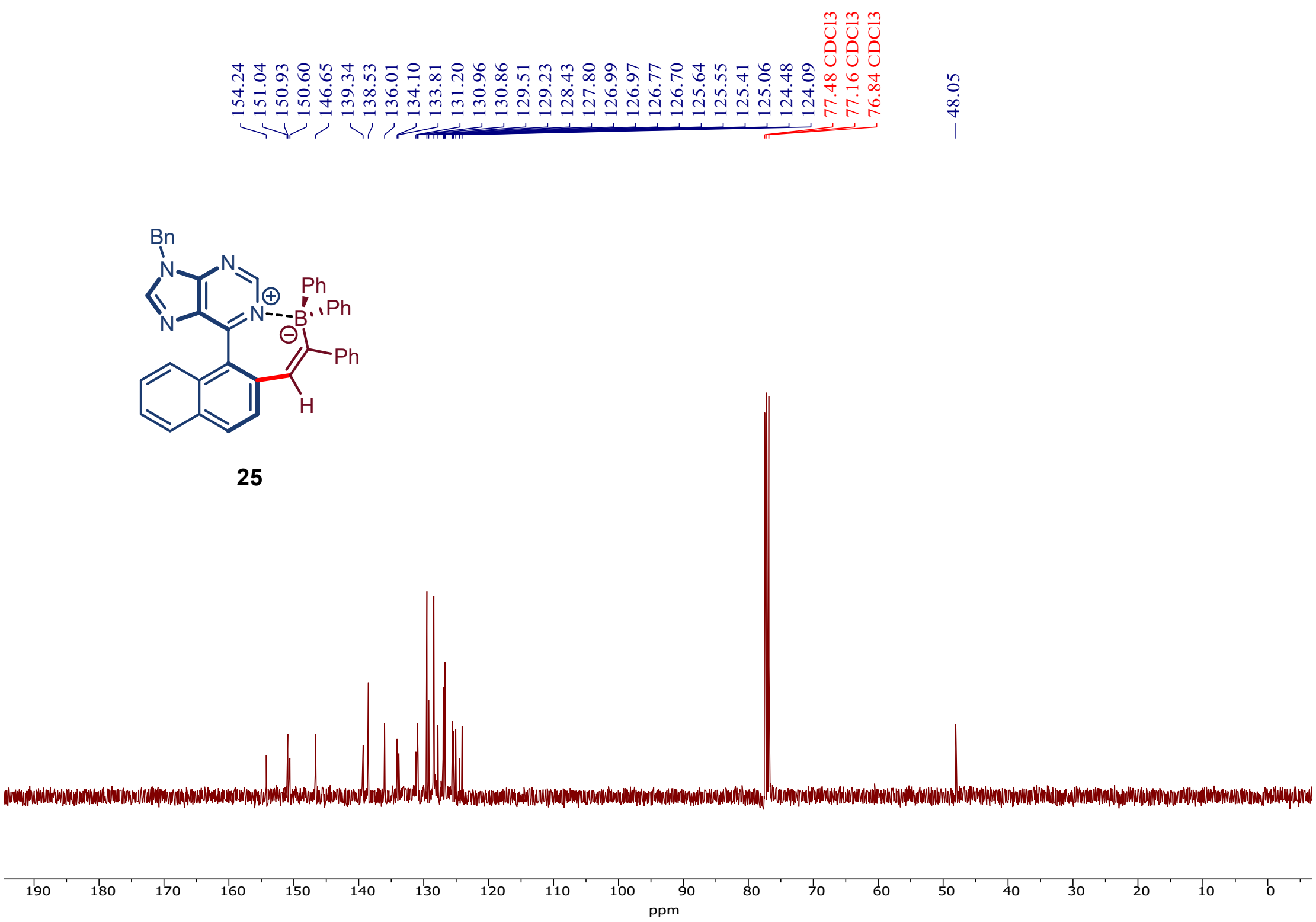

^{11}B NMR (128 MHz, $CDCl_3$) of **25**

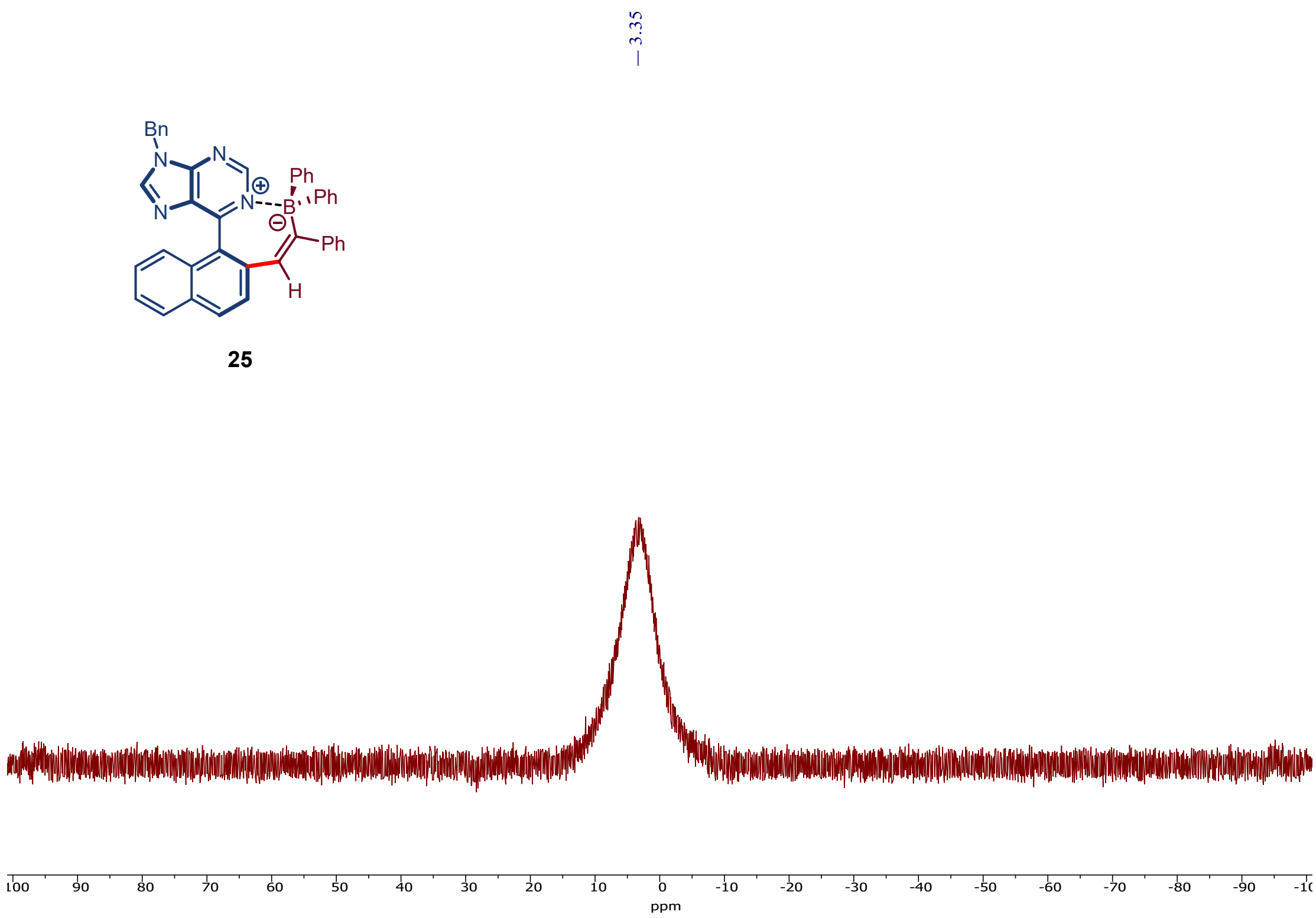

^{1}H NMR (400 MHz, $CDCl_3$) of **26** (*see procedure*)

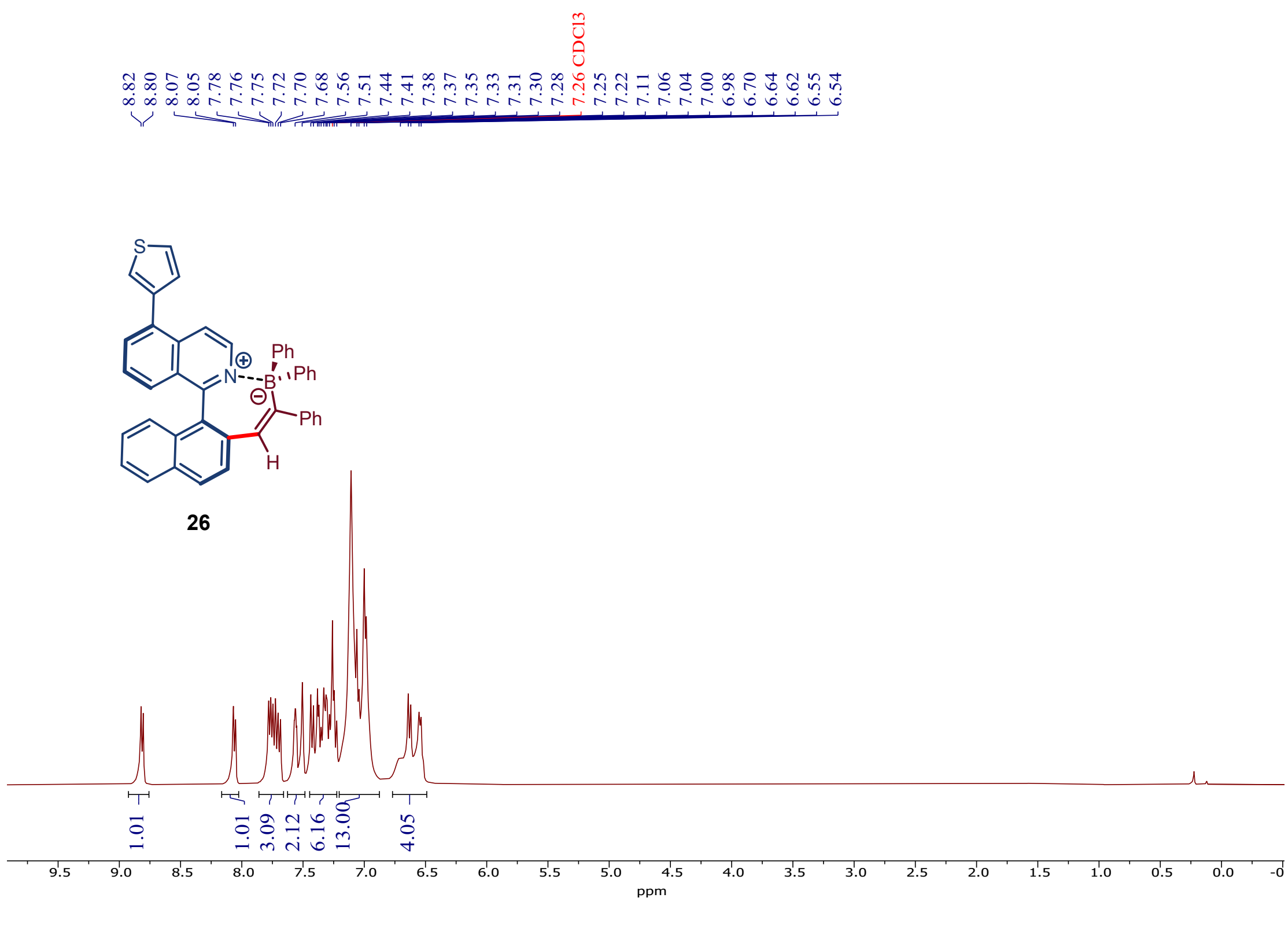

^{13}C NMR (100 MHz, $CDCl_3$) of **26**

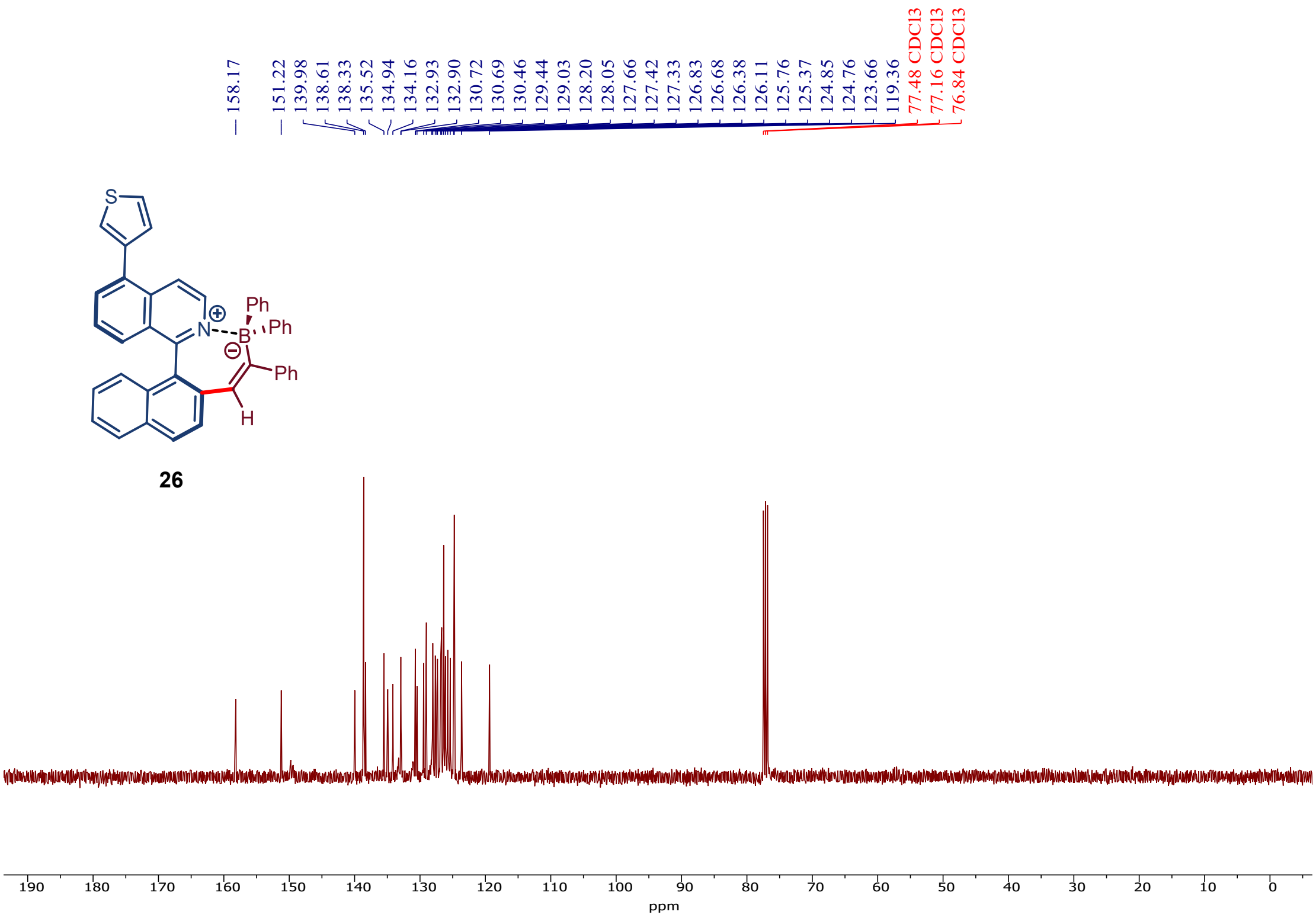

^{11}B NMR (128 MHz, $CDCl_3$) of **26**

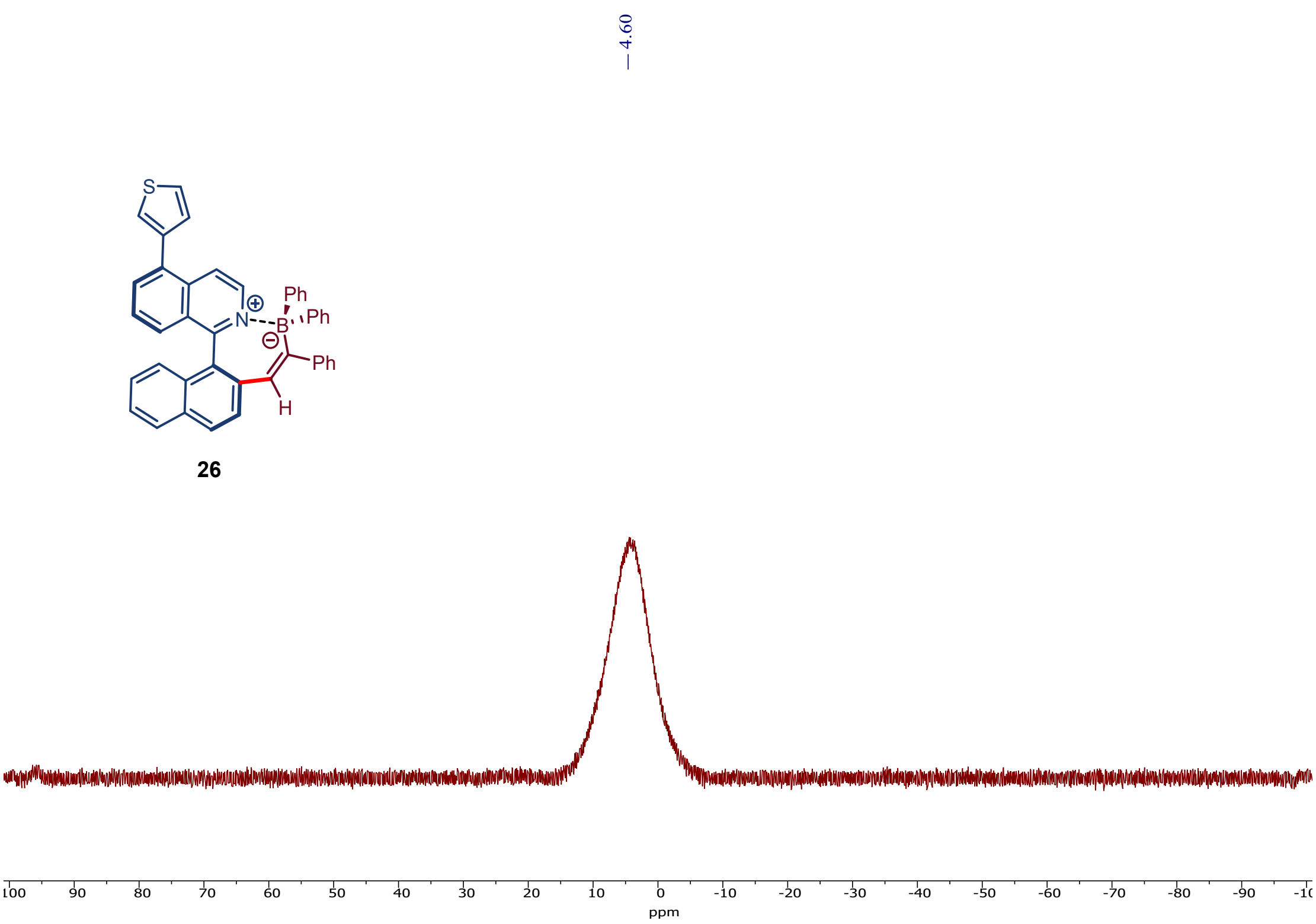

^{1}H NMR (400 MHz, $CDCl_3$) of **27** (*see procedure*)

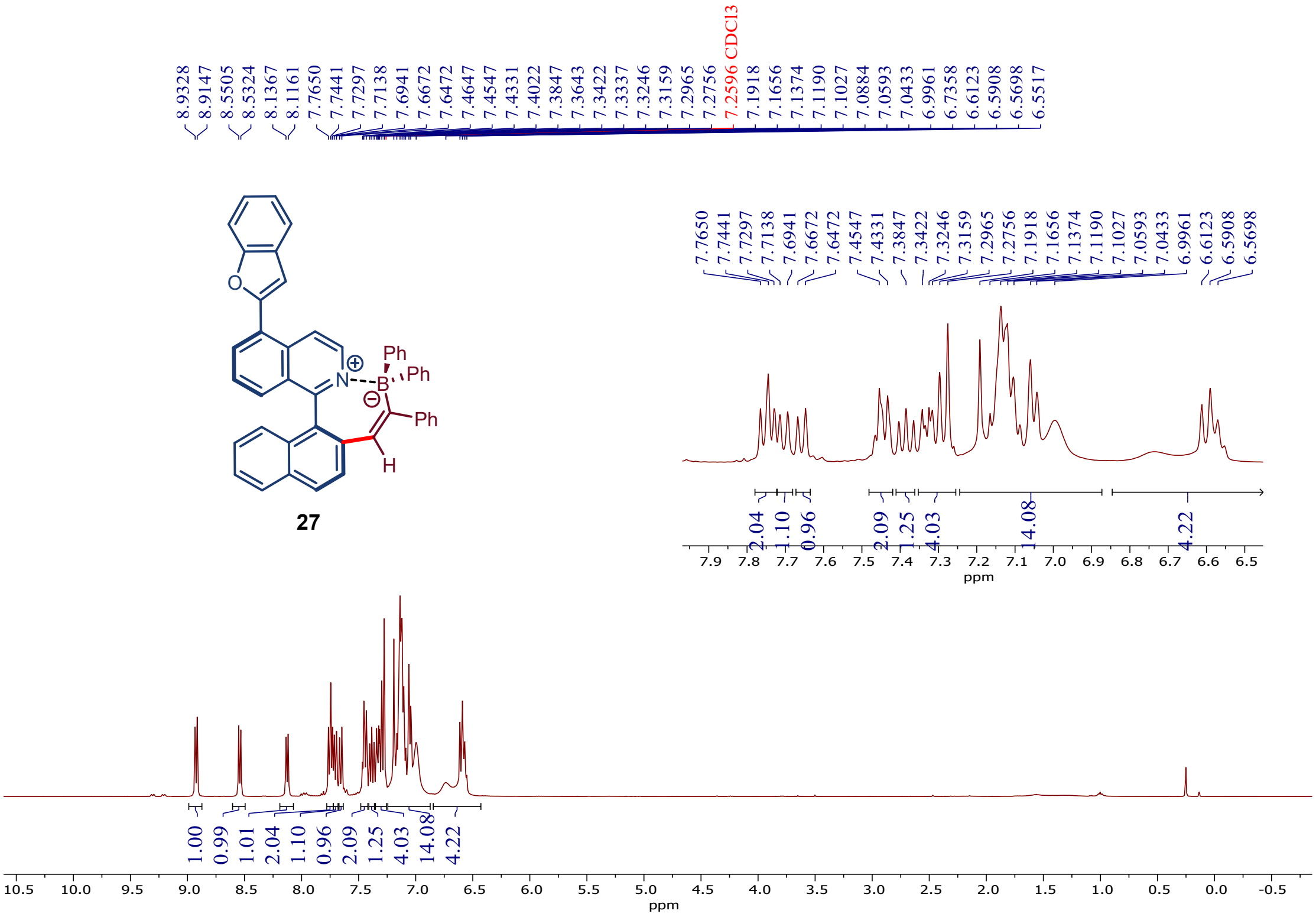

^{13}C NMR (100 MHz, $CDCl_3$) of **27**

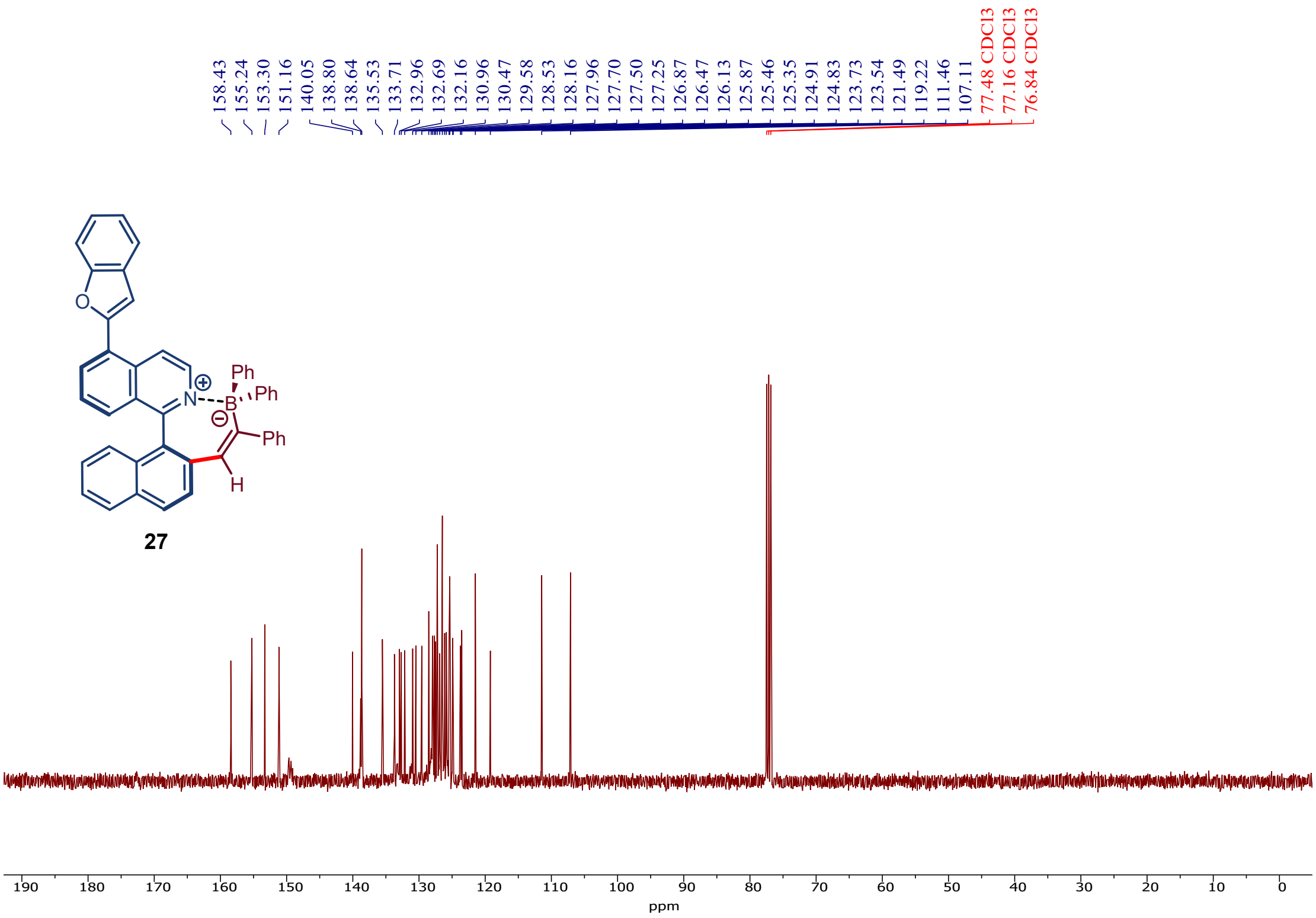

^{11}B NMR (128 MHz, $CDCl_3$) of **27**

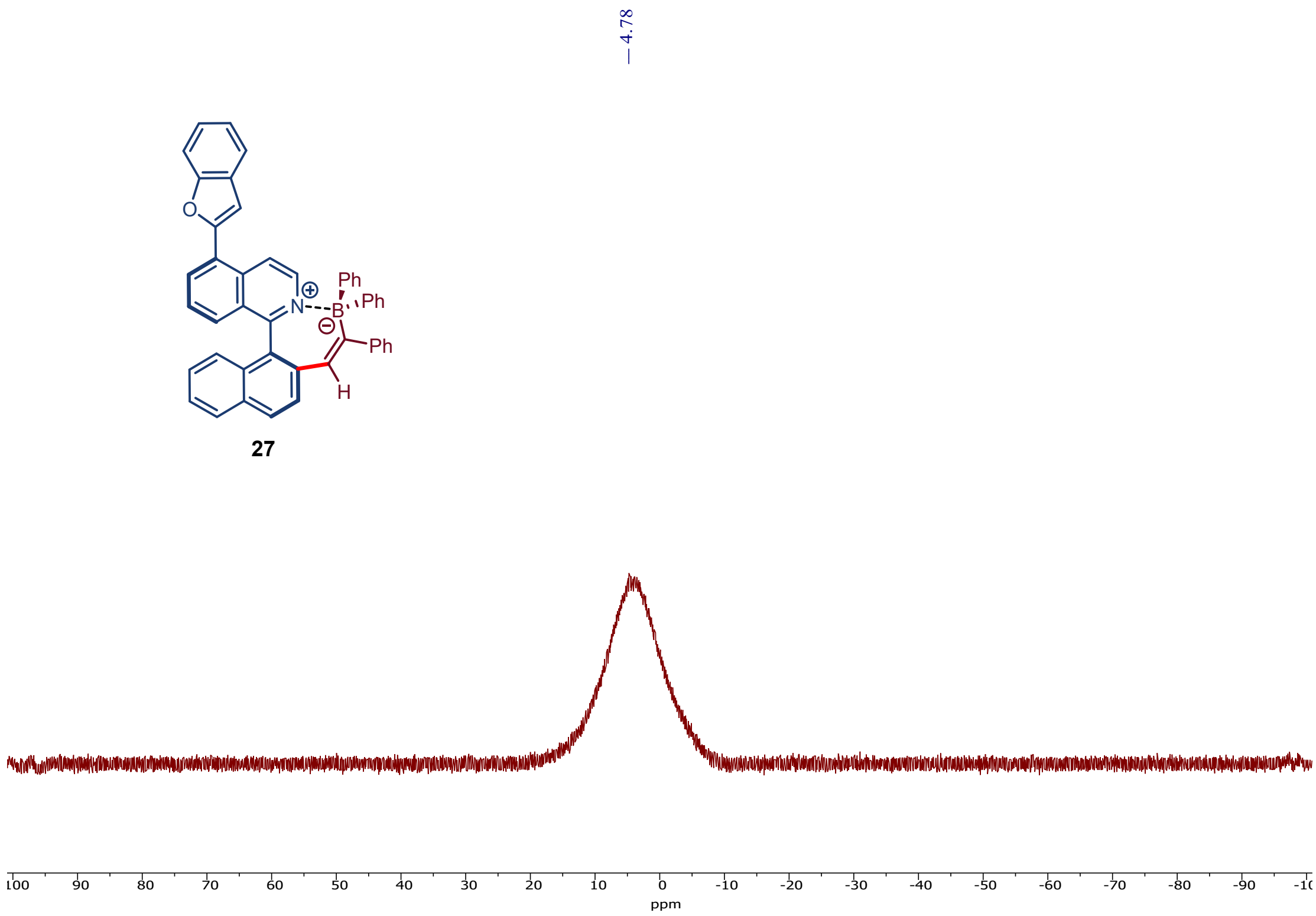

^{1}H NMR (400 MHz, $CDCl_3$) of **28** (*see procedure*)

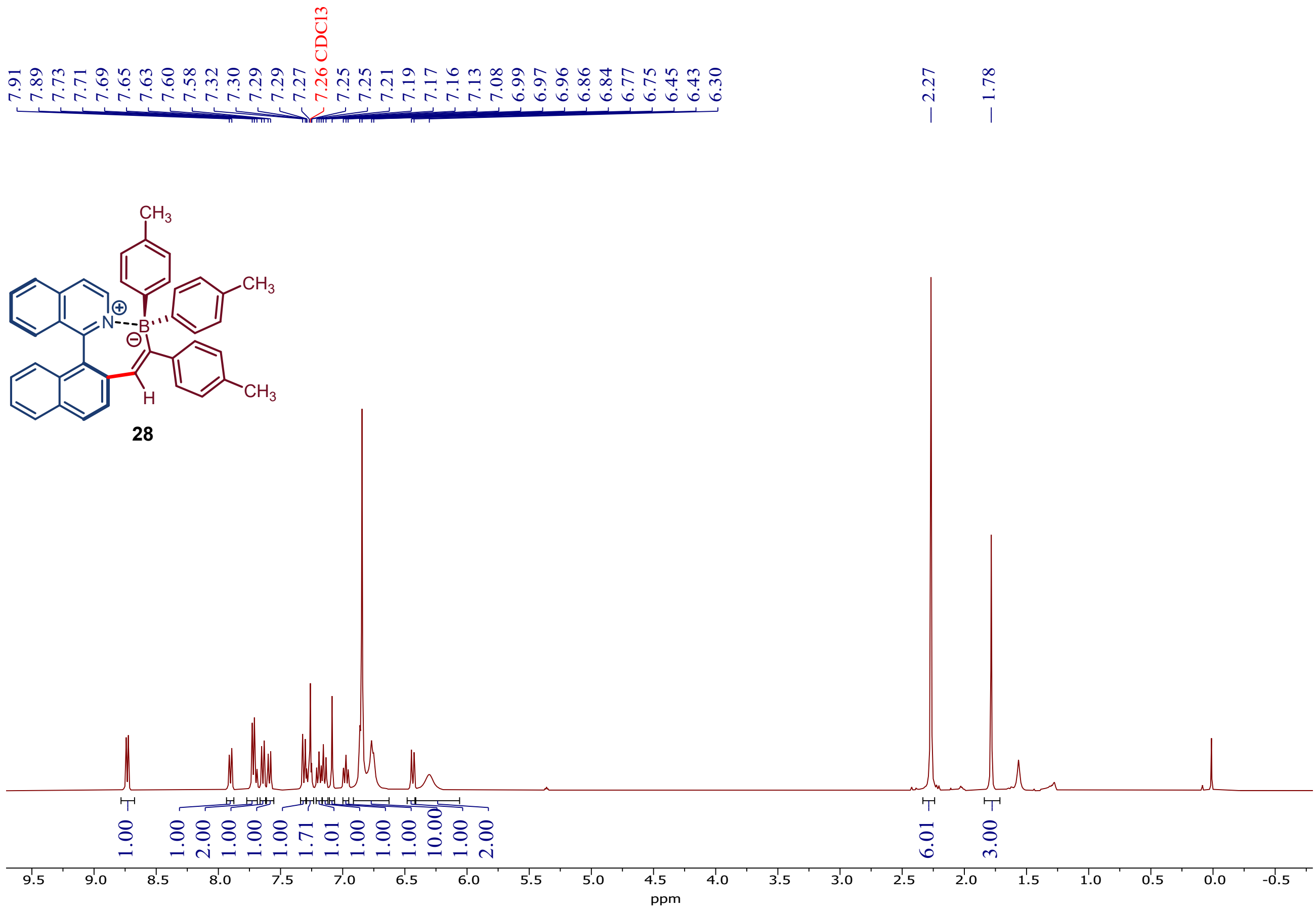

^{13}C NMR (100 MHz, $CDCl_3$) of **28**

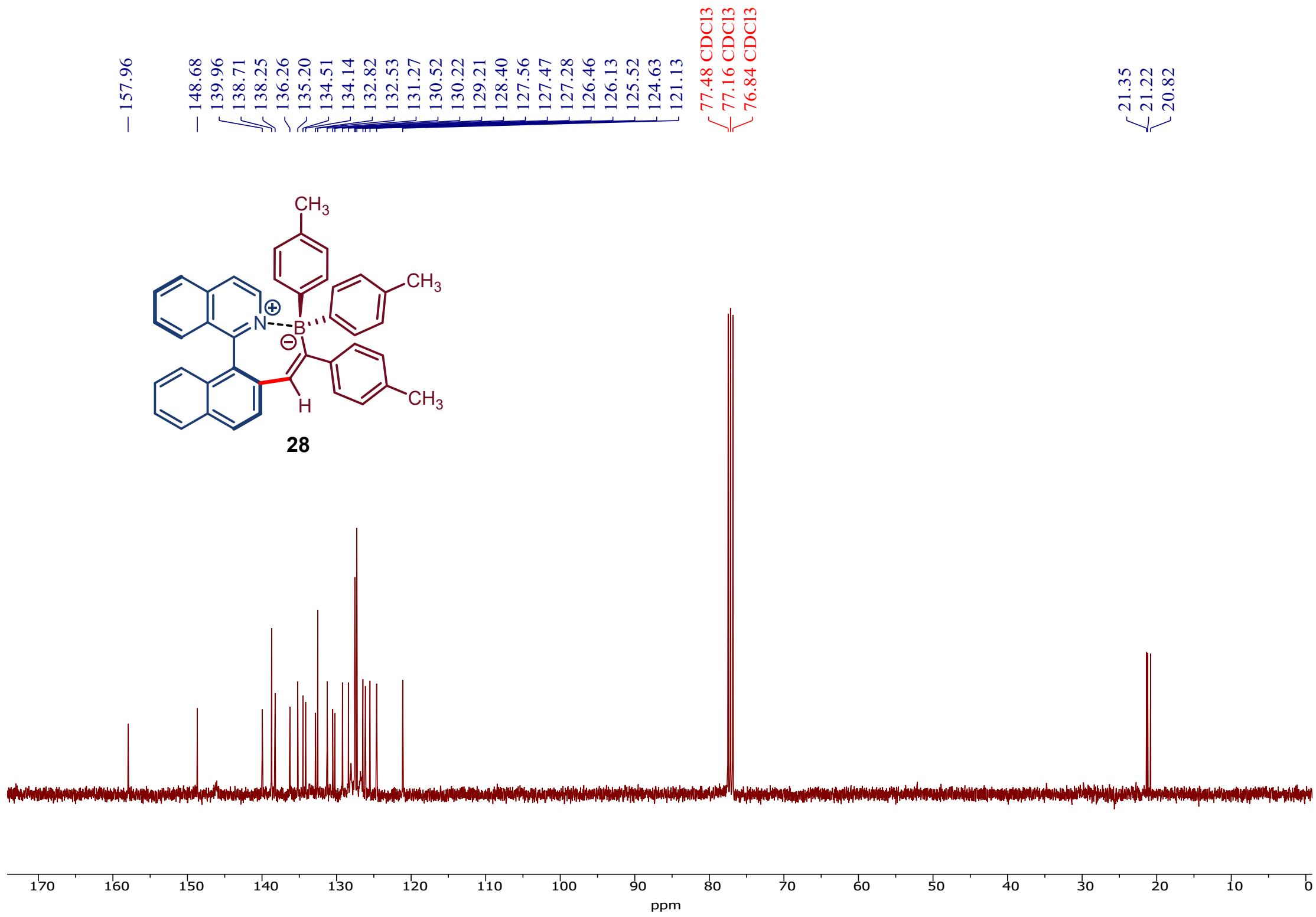

^{11}B NMR (128 MHz, $CDCl_3$) of **28**

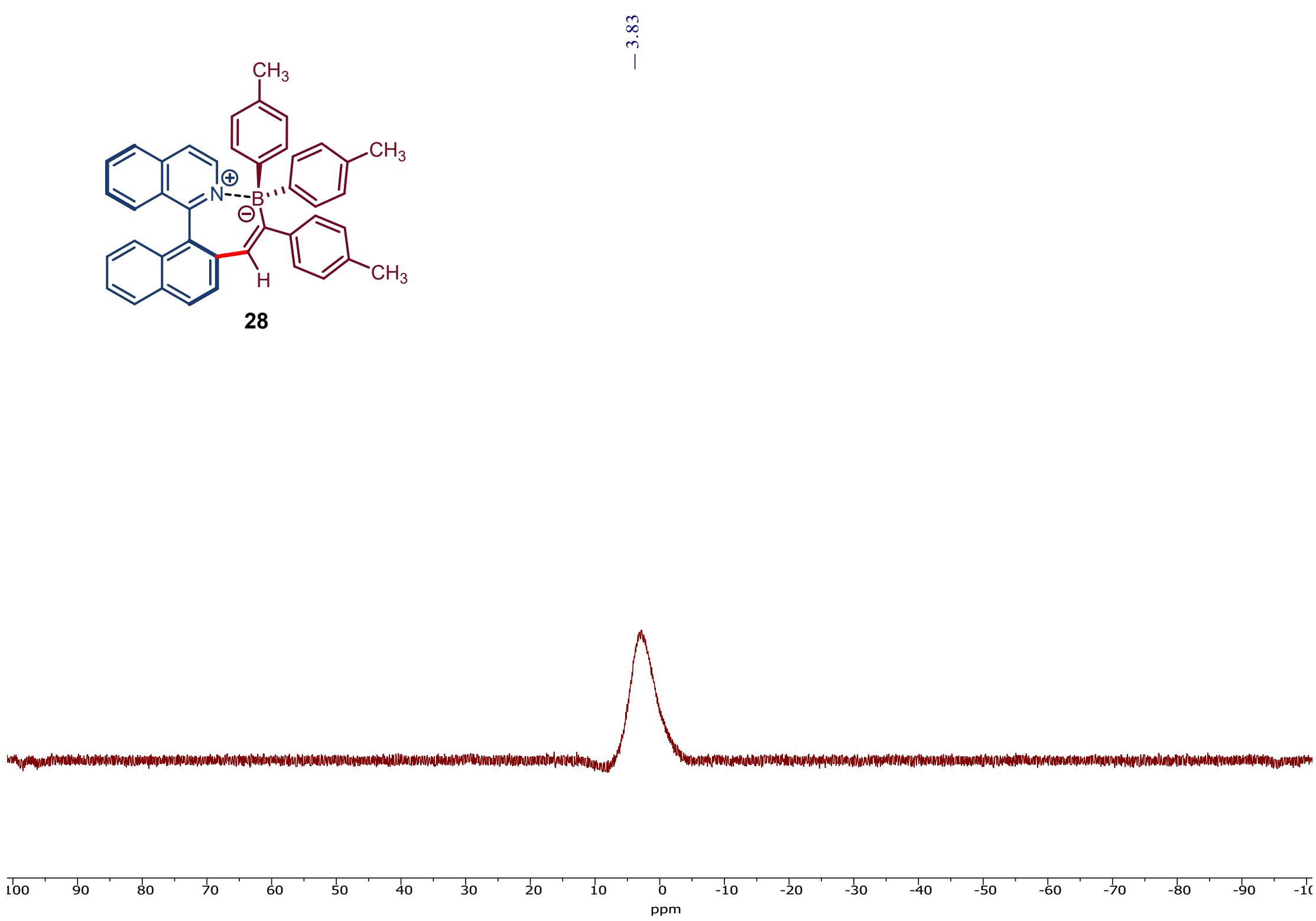

^{1}H NMR (400 MHz, $CDCl_3$) of **29** (*see procedure*)

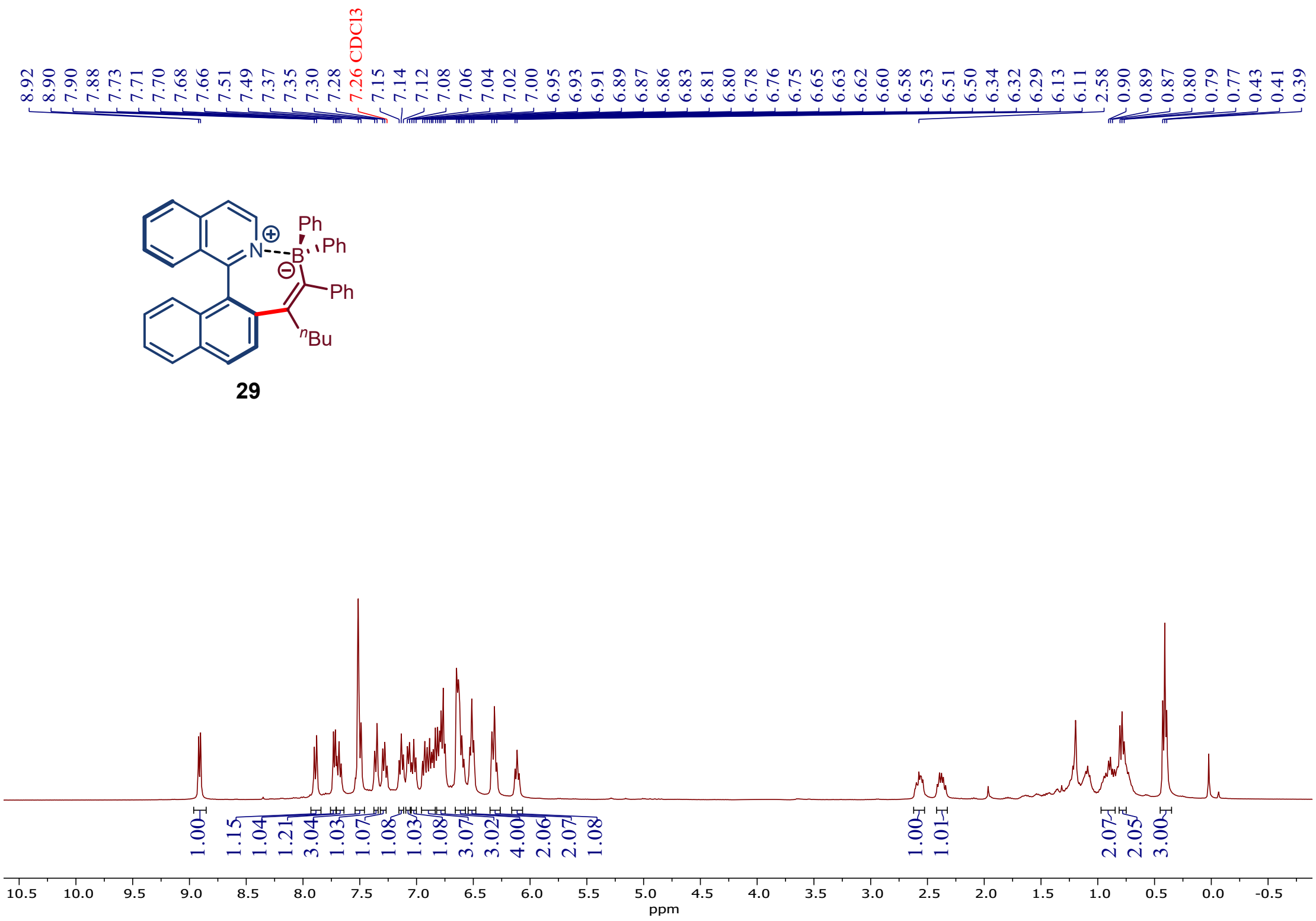

^{13}C NMR (100 MHz, $CDCl_3$) of **29**

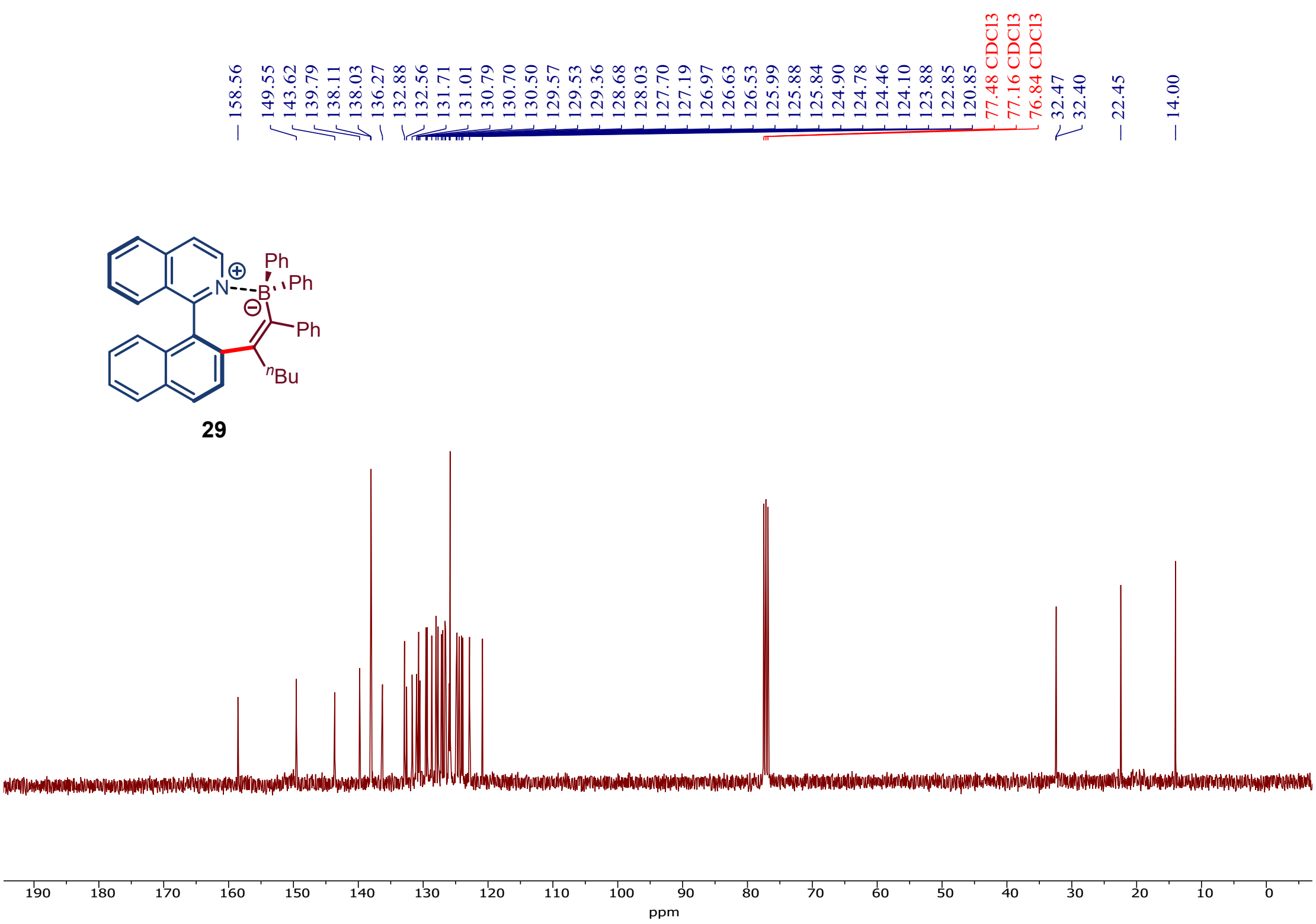

^{11}B NMR (128 MHz, $CDCl_3$) of **29**

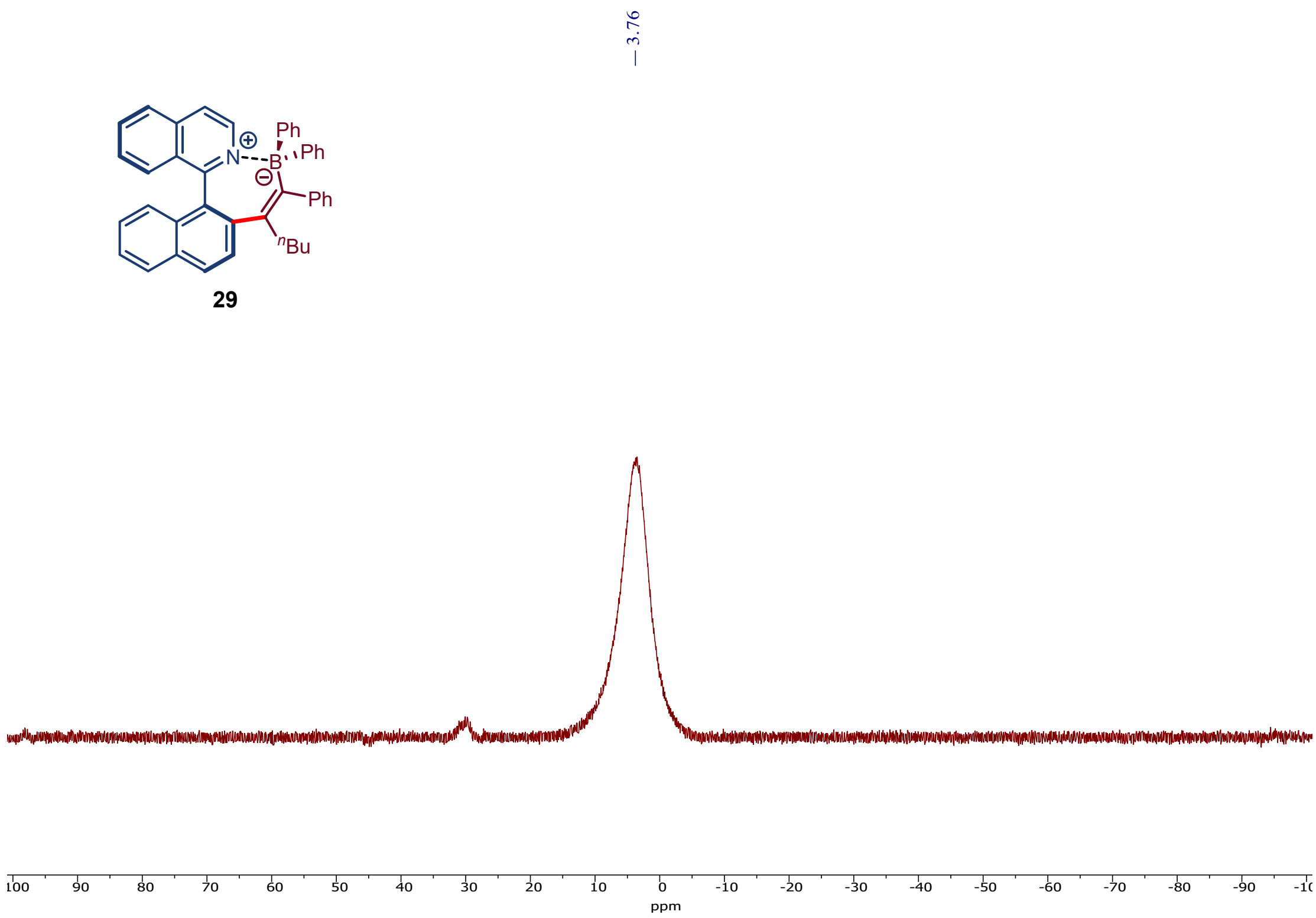

^{1}H NMR (400 MHz, $CDCl_3$) of **30** (*see procedure*)

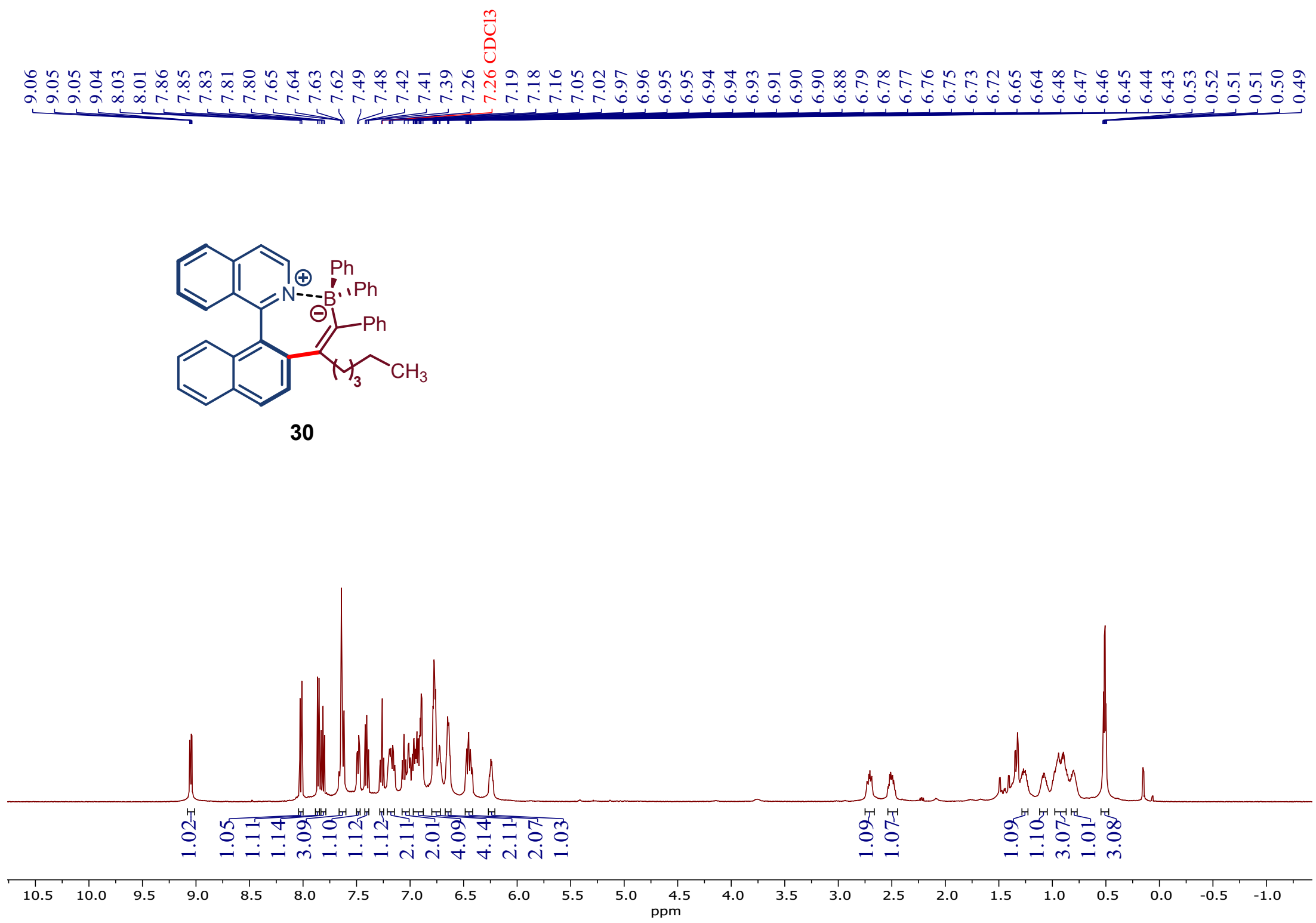

^{13}C NMR (100 MHz, $CDCl_3$) of **30**

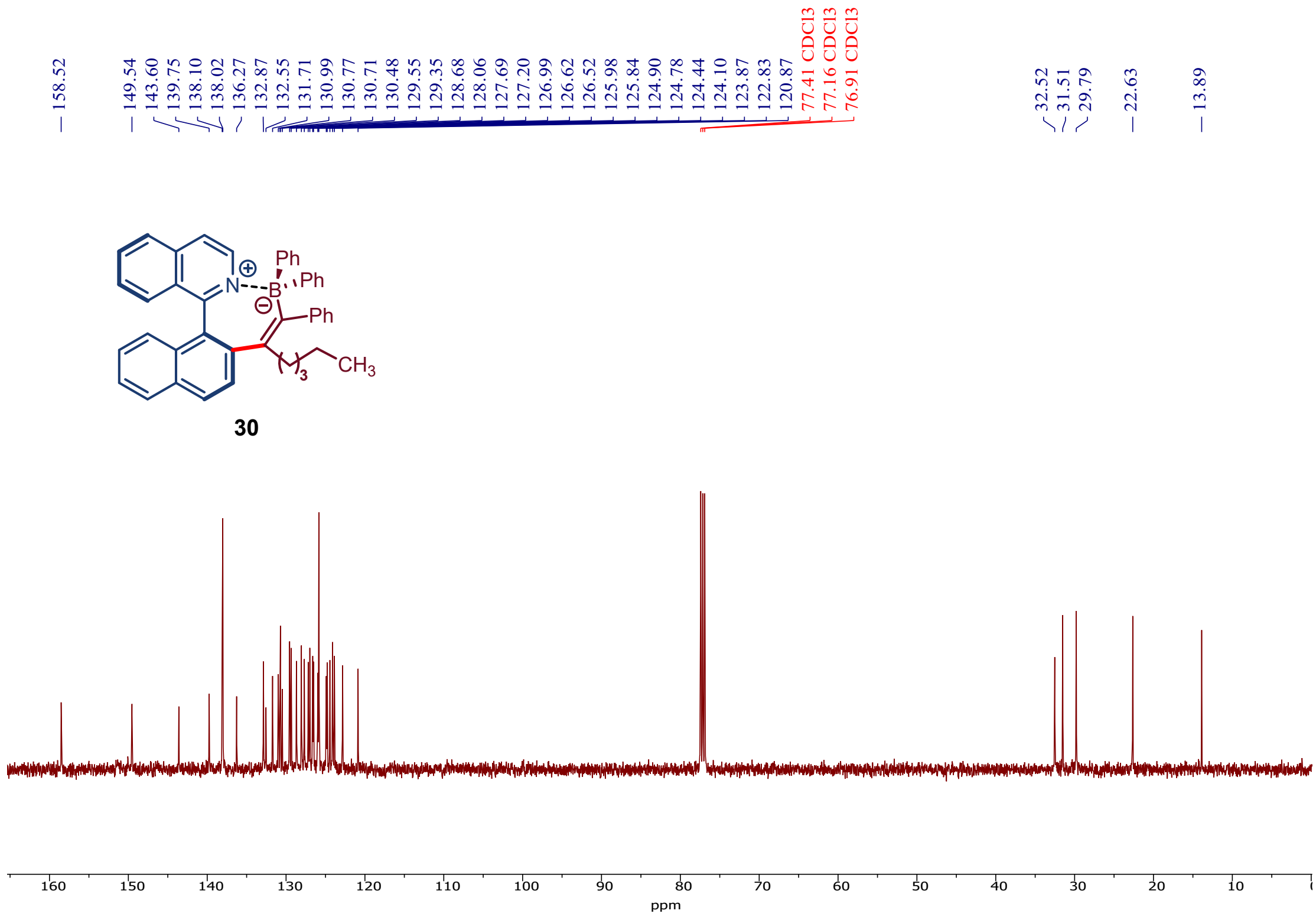

^{11}B NMR (128 MHz, $CDCl_3$) of **30**

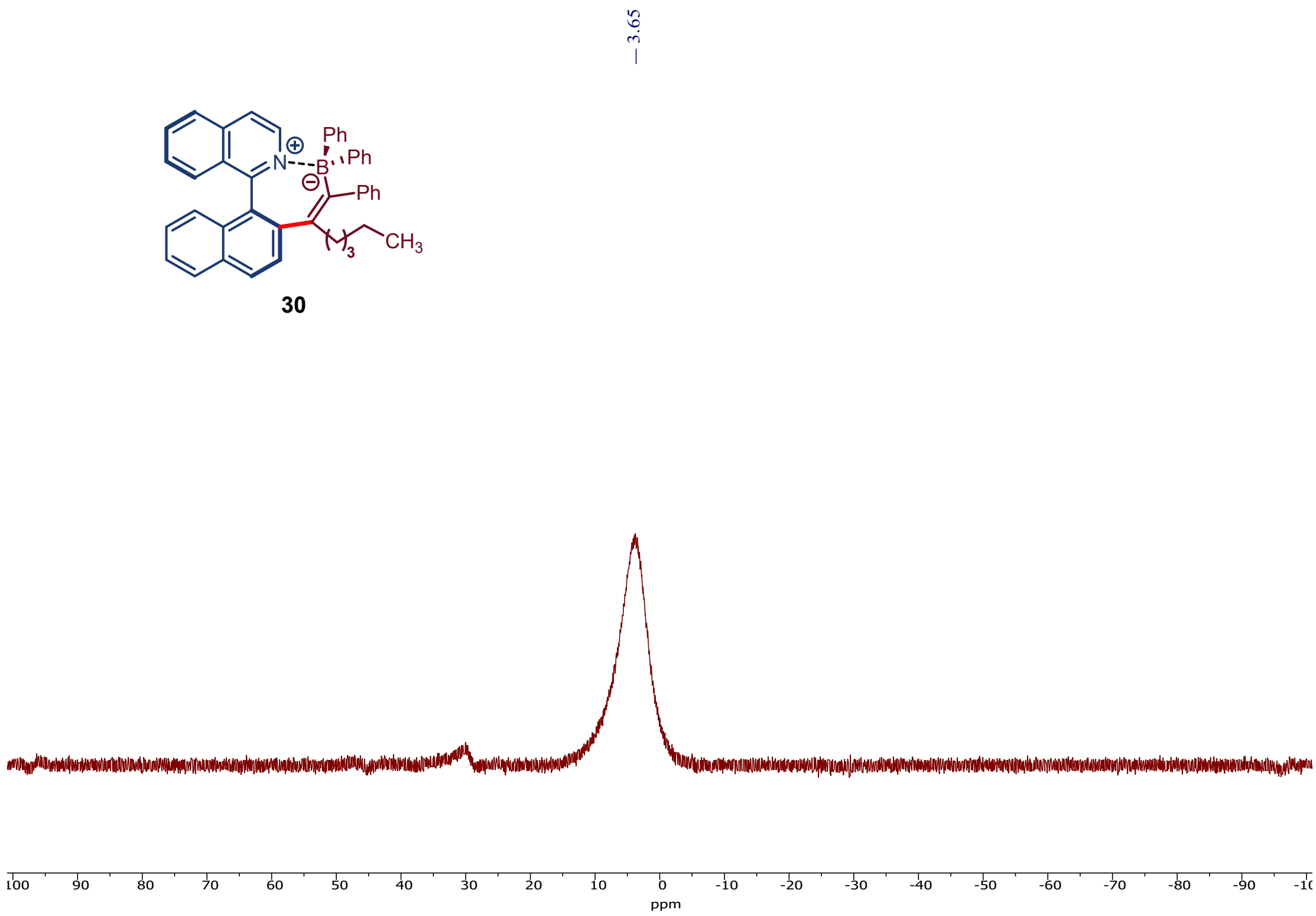

^{1}H NMR (400 MHz, $CDCl_3$) of **31** (*see procedure*)

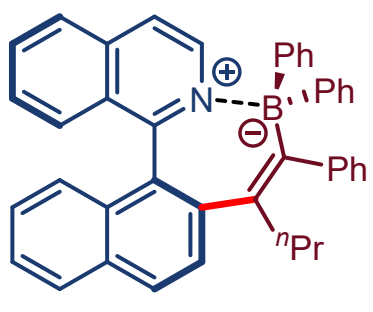

31

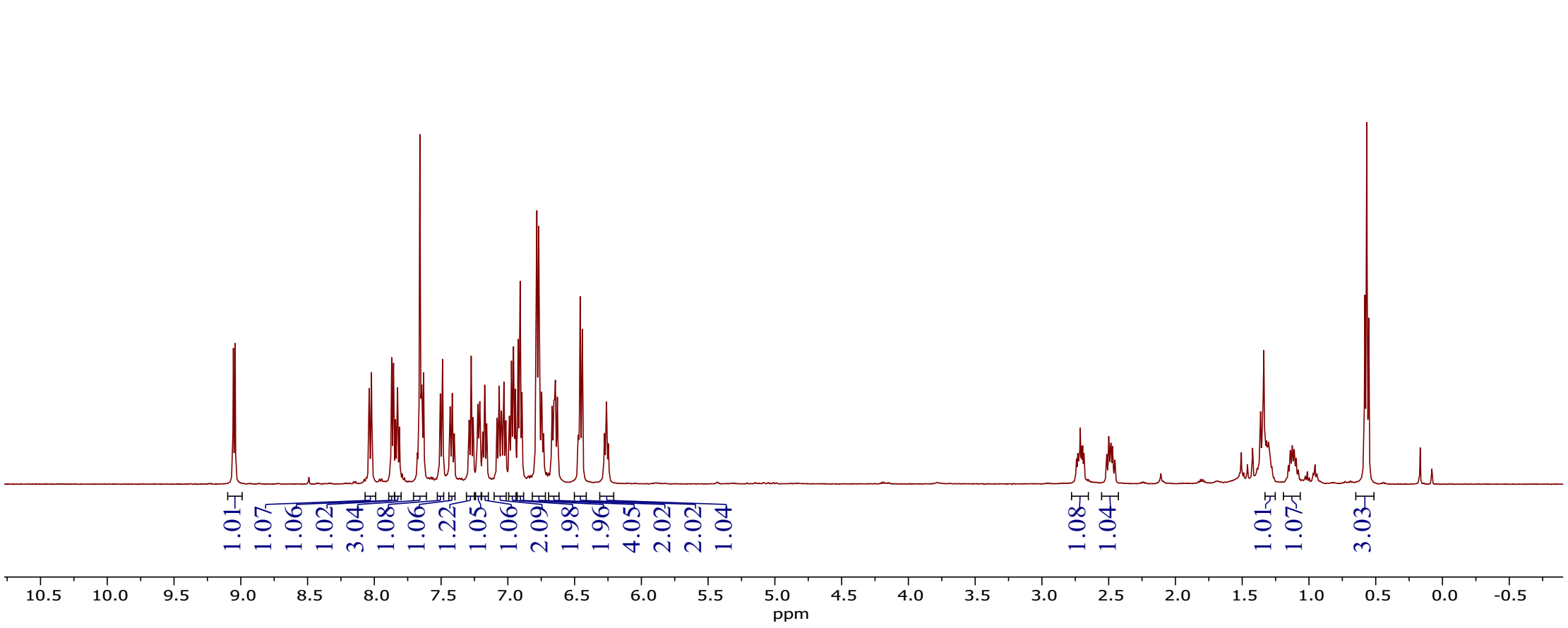

^{13}C NMR (100 MHz, $CDCl_3$) of **31**

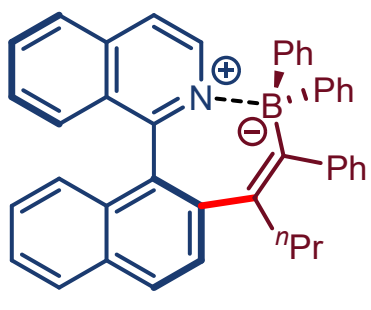

31

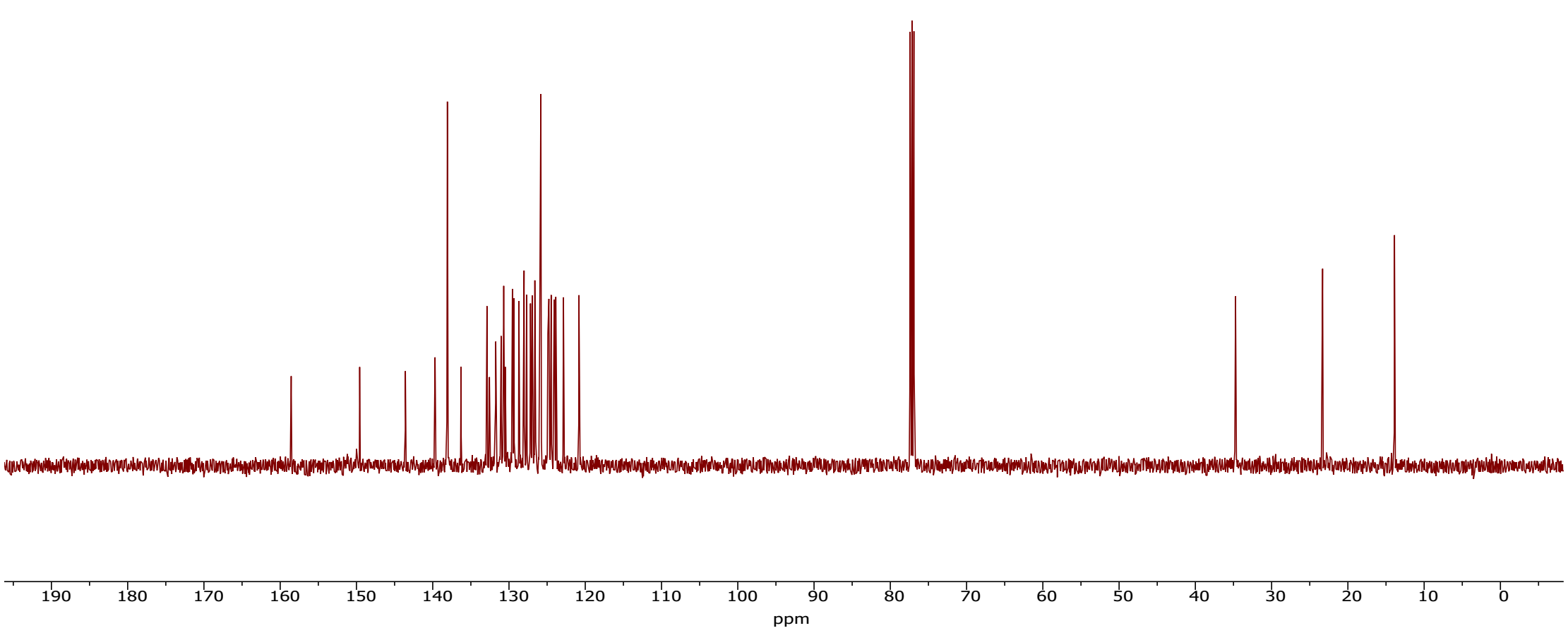

^{11}B NMR (128 MHz, $CDCl_3$) of **31**

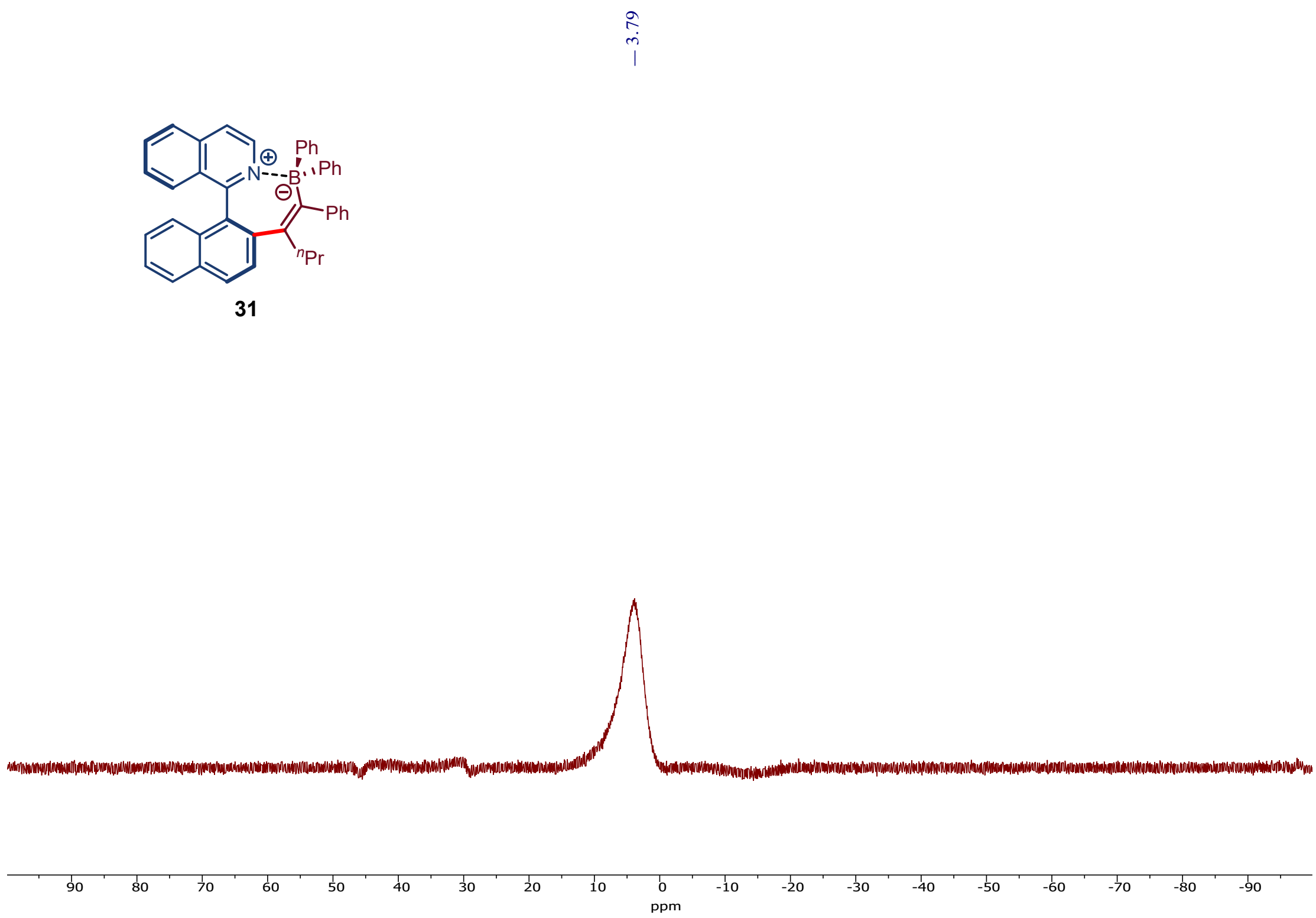

^{1}H NMR (400 MHz, $CDCl_3$) of **32** (*see procedure*)

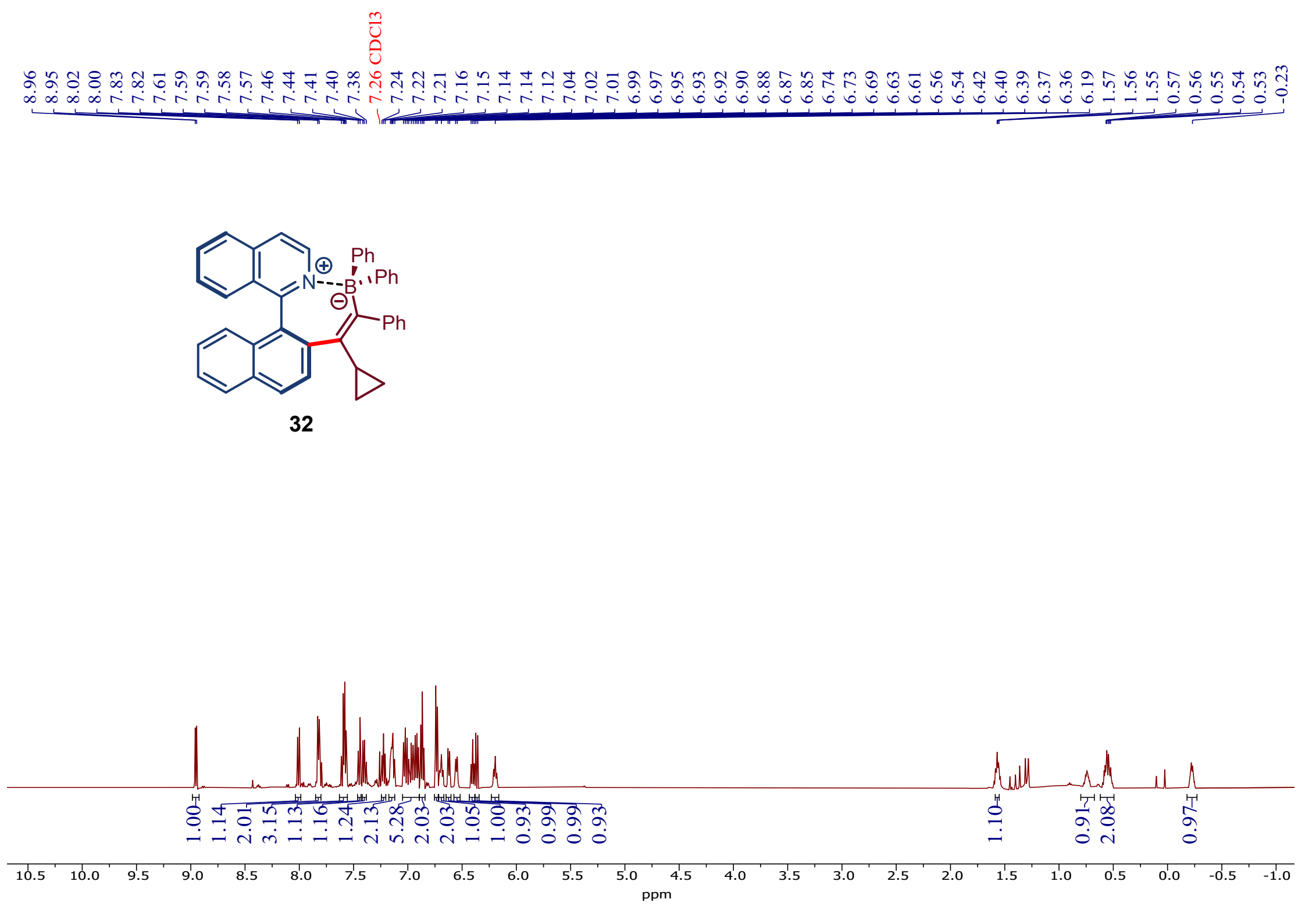

^{13}C NMR (100 MHz, $CDCl_3$) of **32**

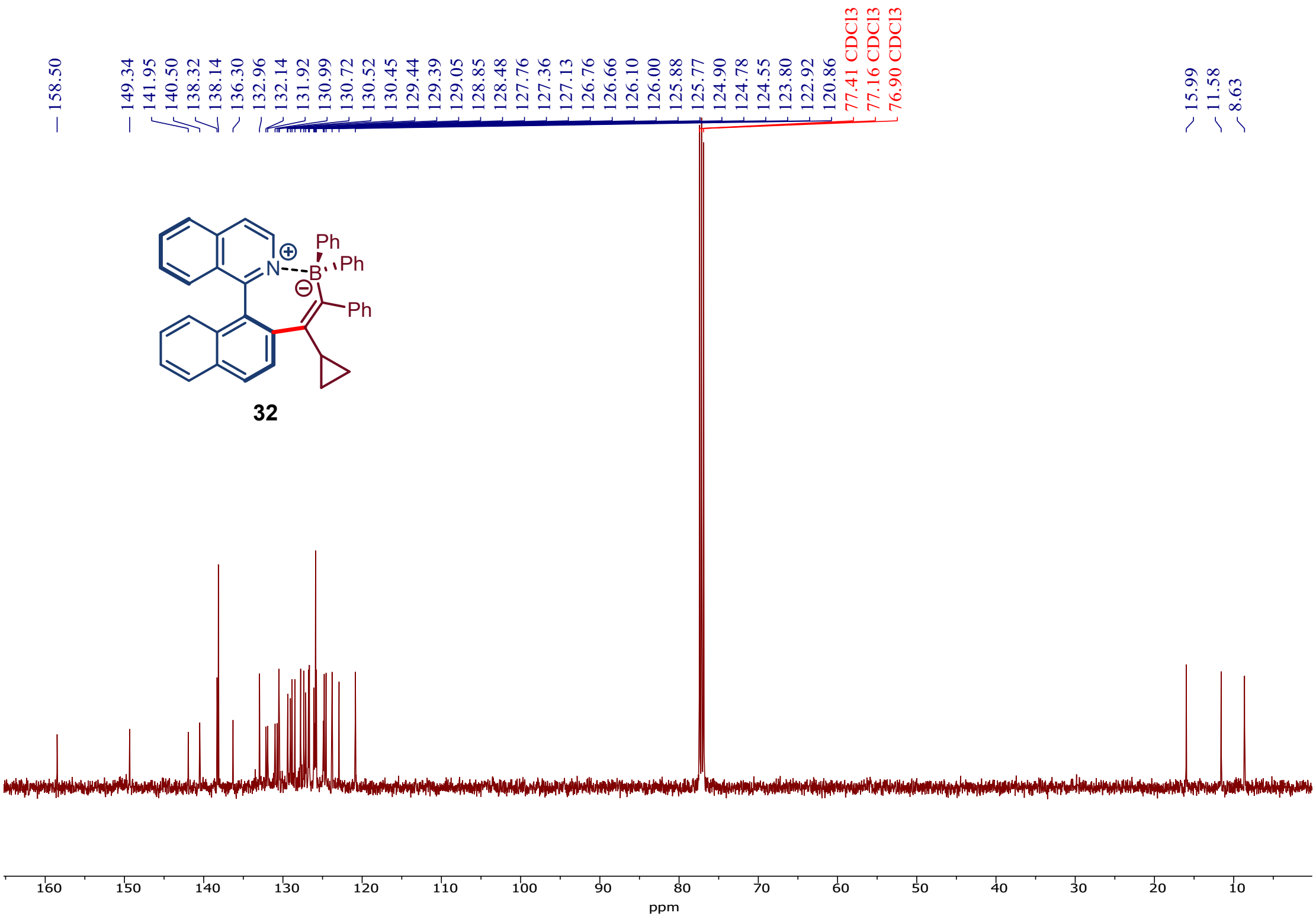

^{11}B NMR (128 MHz, $CDCl_3$) of **32**

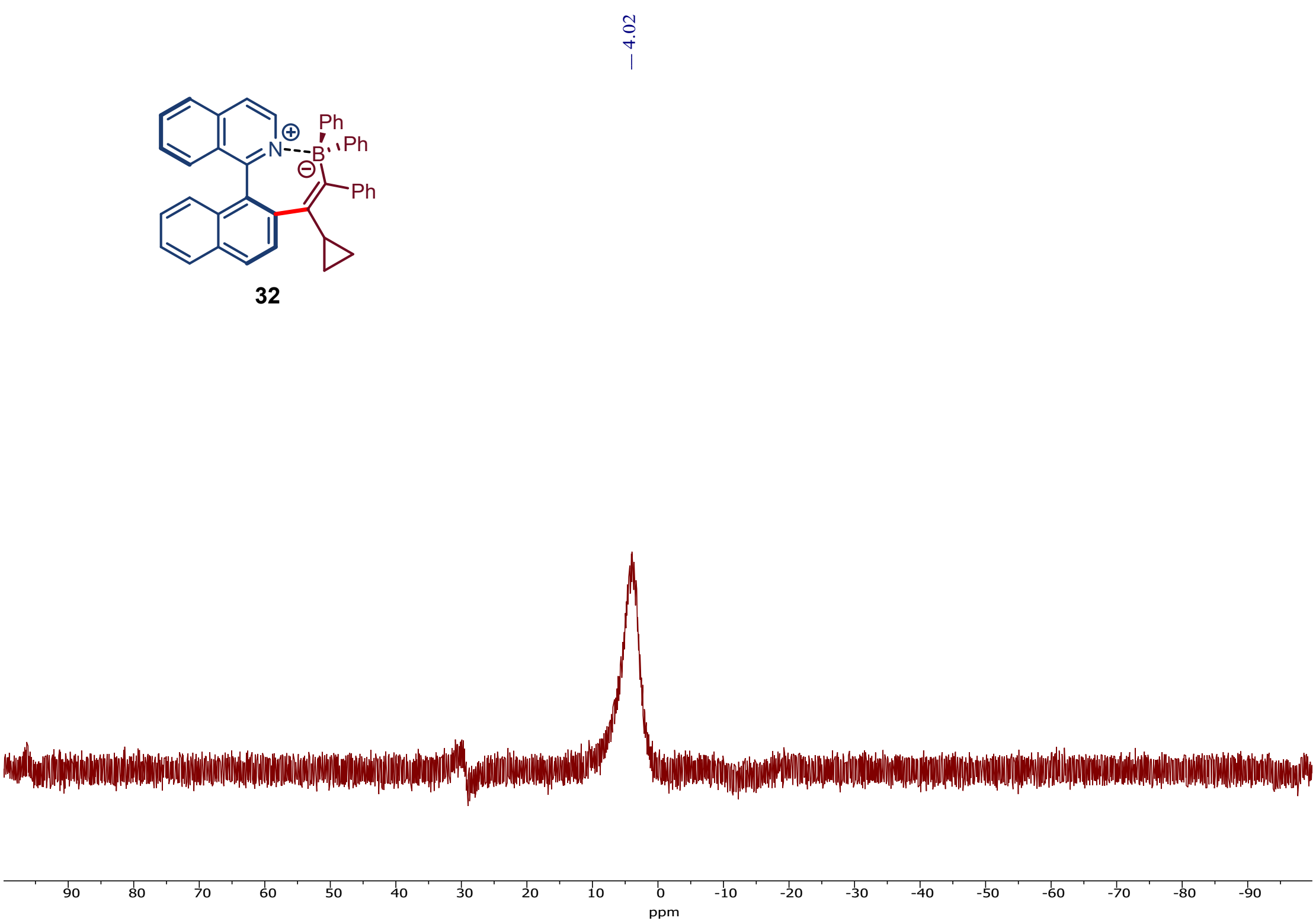

^{1}H NMR (400 MHz, $CDCl_3$) of **33** (*see procedure*)

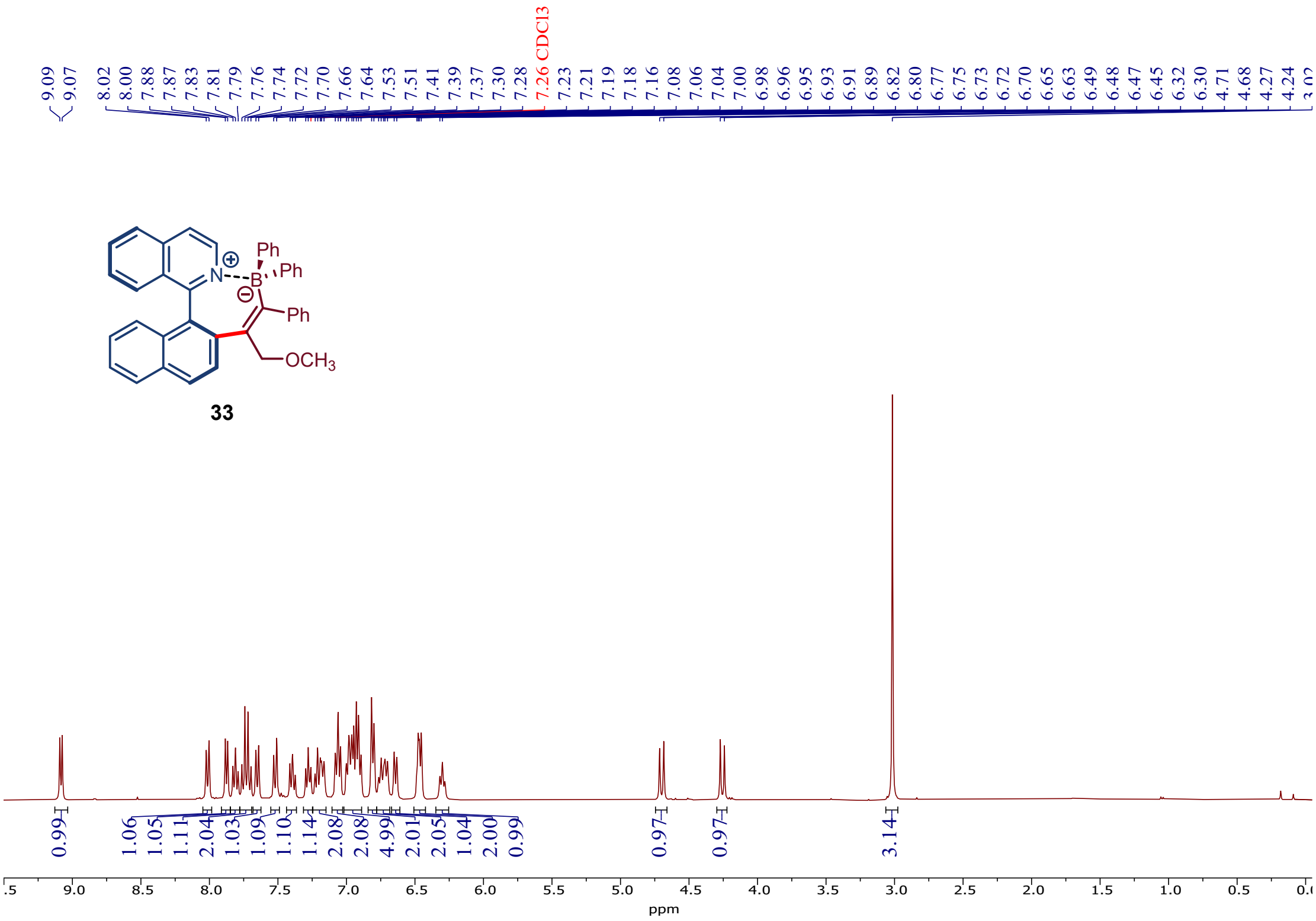

^{13}C NMR (100 MHz, $CDCl_3$) of **33**

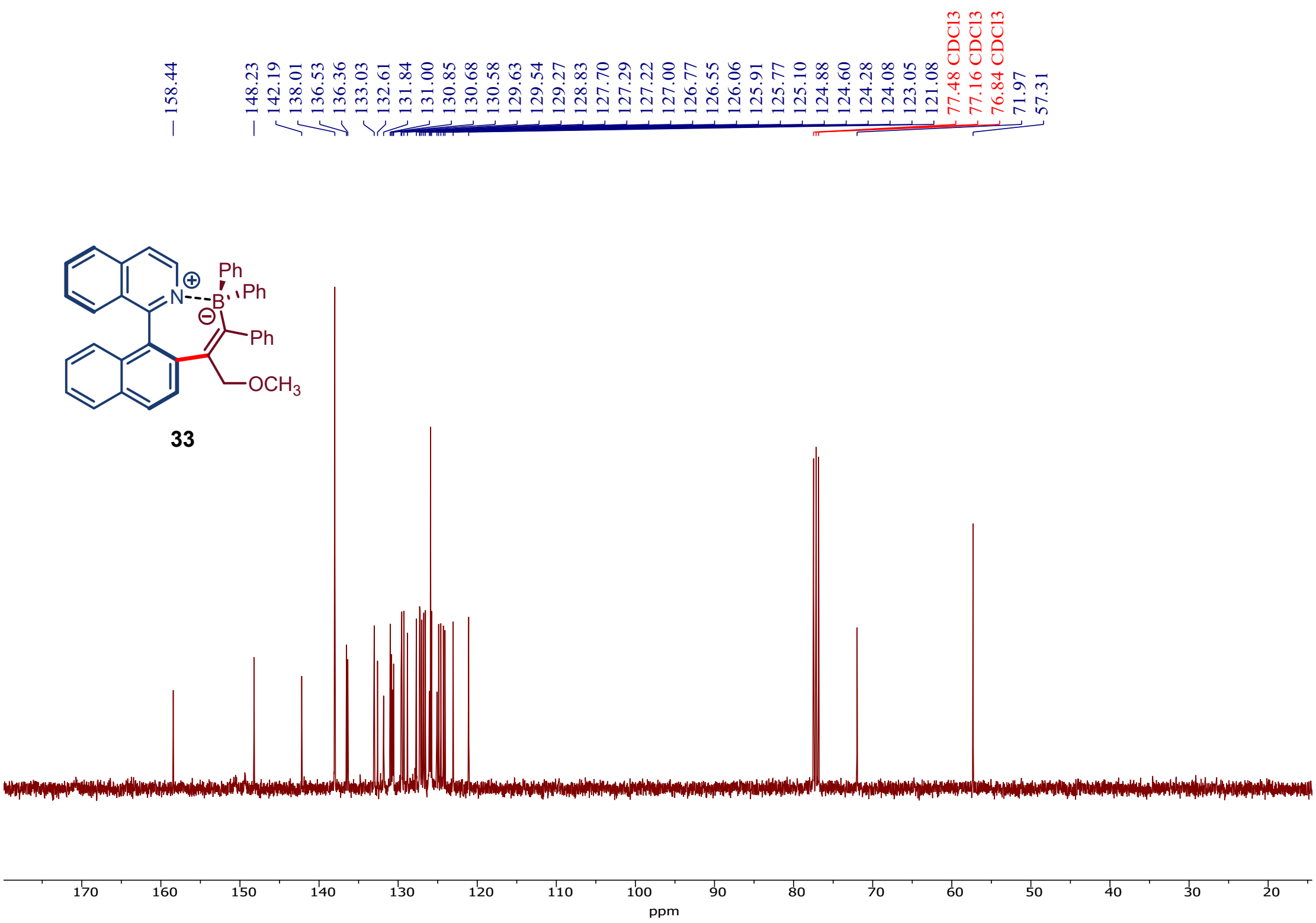

^{11}B NMR (128 MHz, $CDCl_3$) of **33**

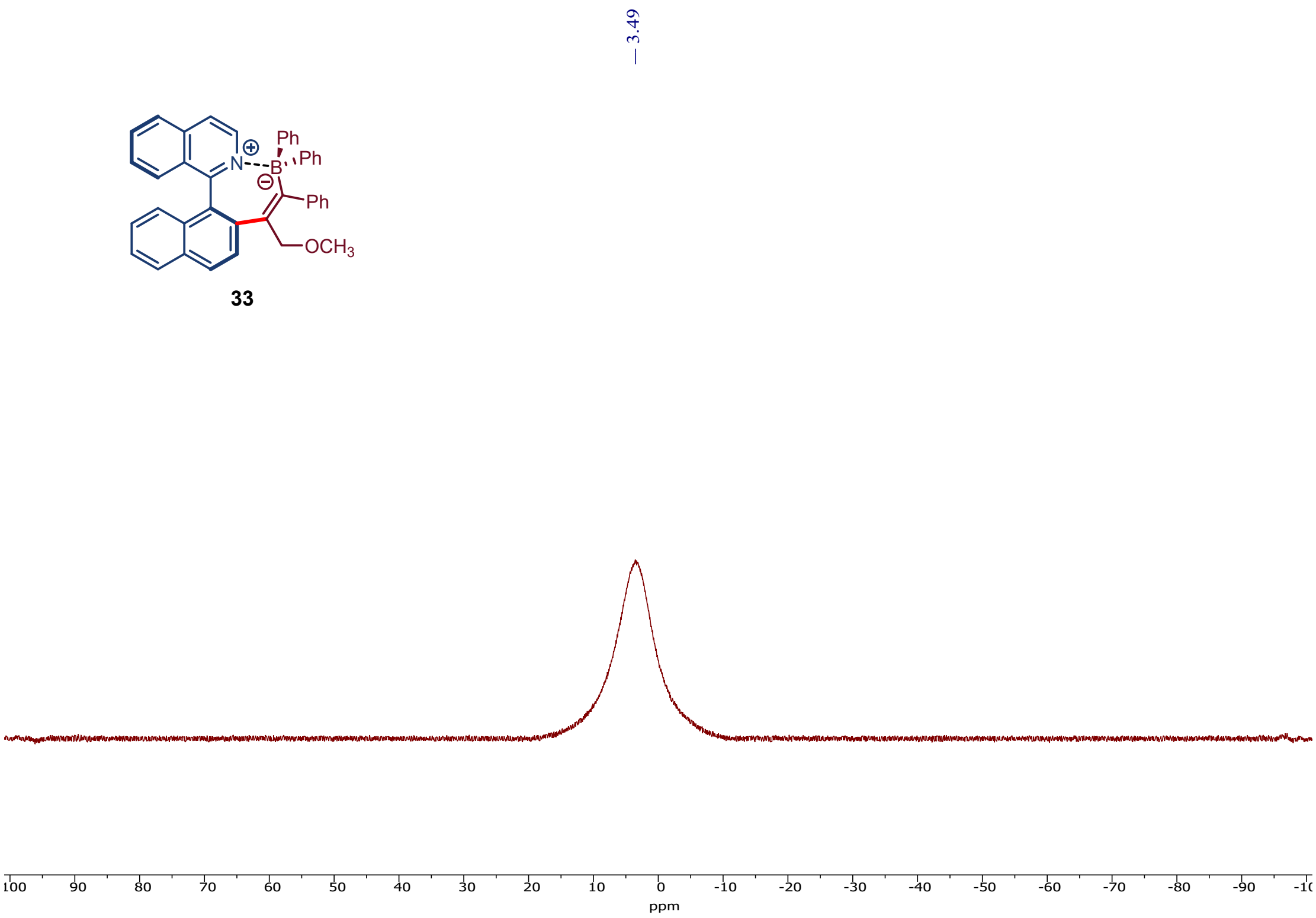

^{1}H NMR (500 MHz, $CDCl_3$) of **34** (*see procedure*)

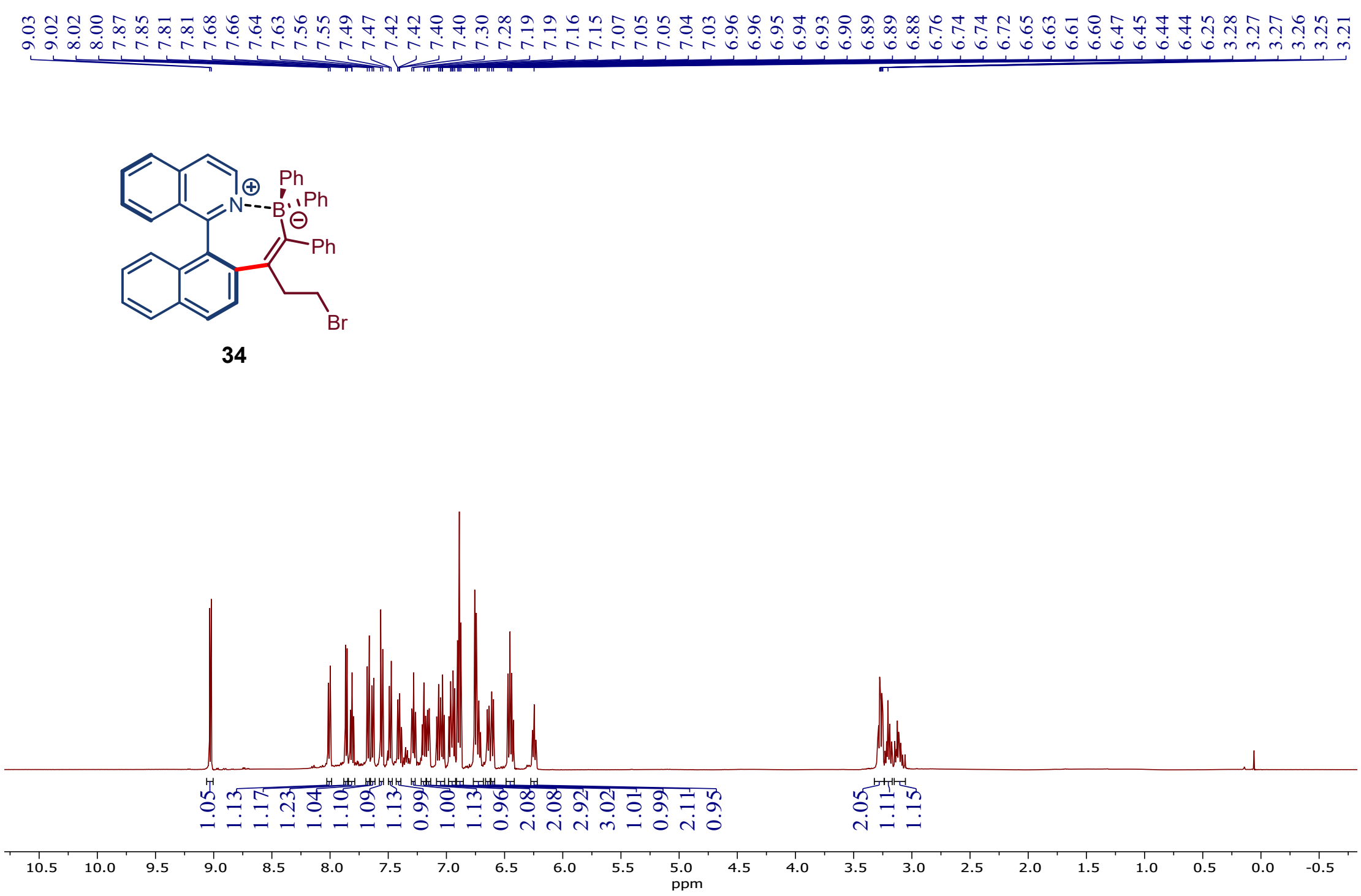

^{13}C NMR (126 MHz, $CDCl_3$) of **34**

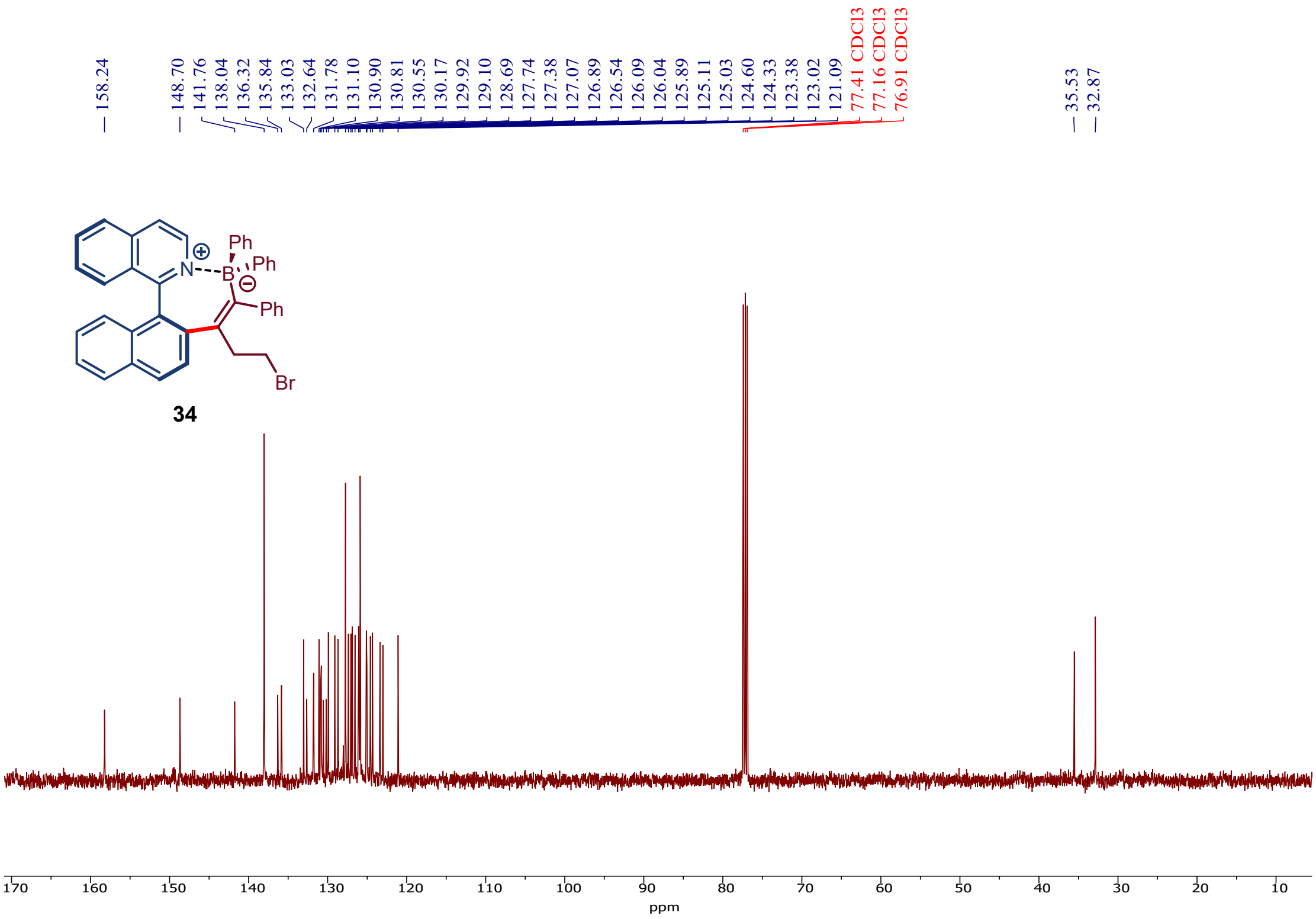

^{11}B NMR (128 MHz, $CDCl_3$) of **34**

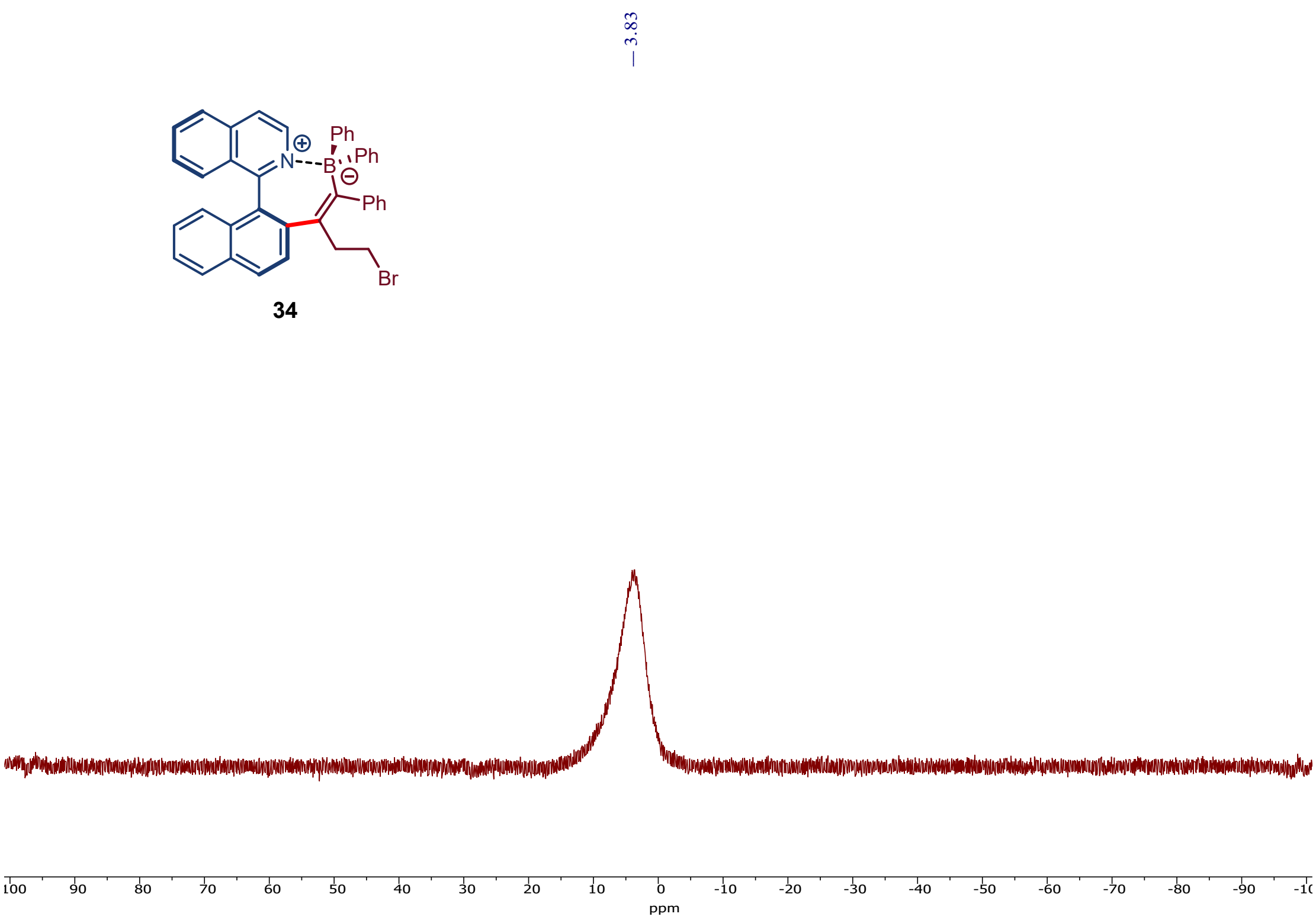

^{1}H NMR (400 MHz, $CDCl_3$) of **35** (see procedure)

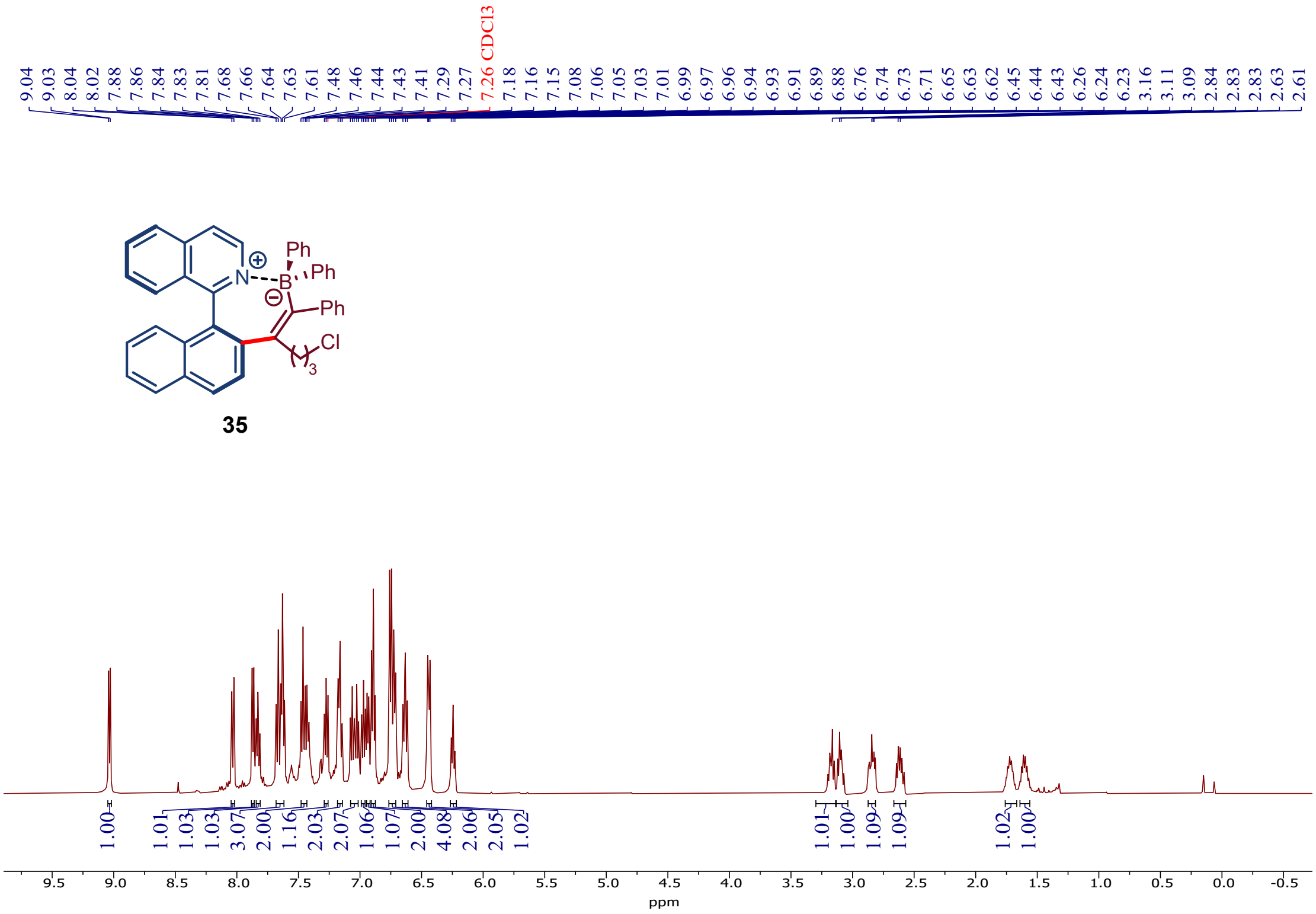

^{13}C NMR (100 MHz, $CDCl_3$) of **35**

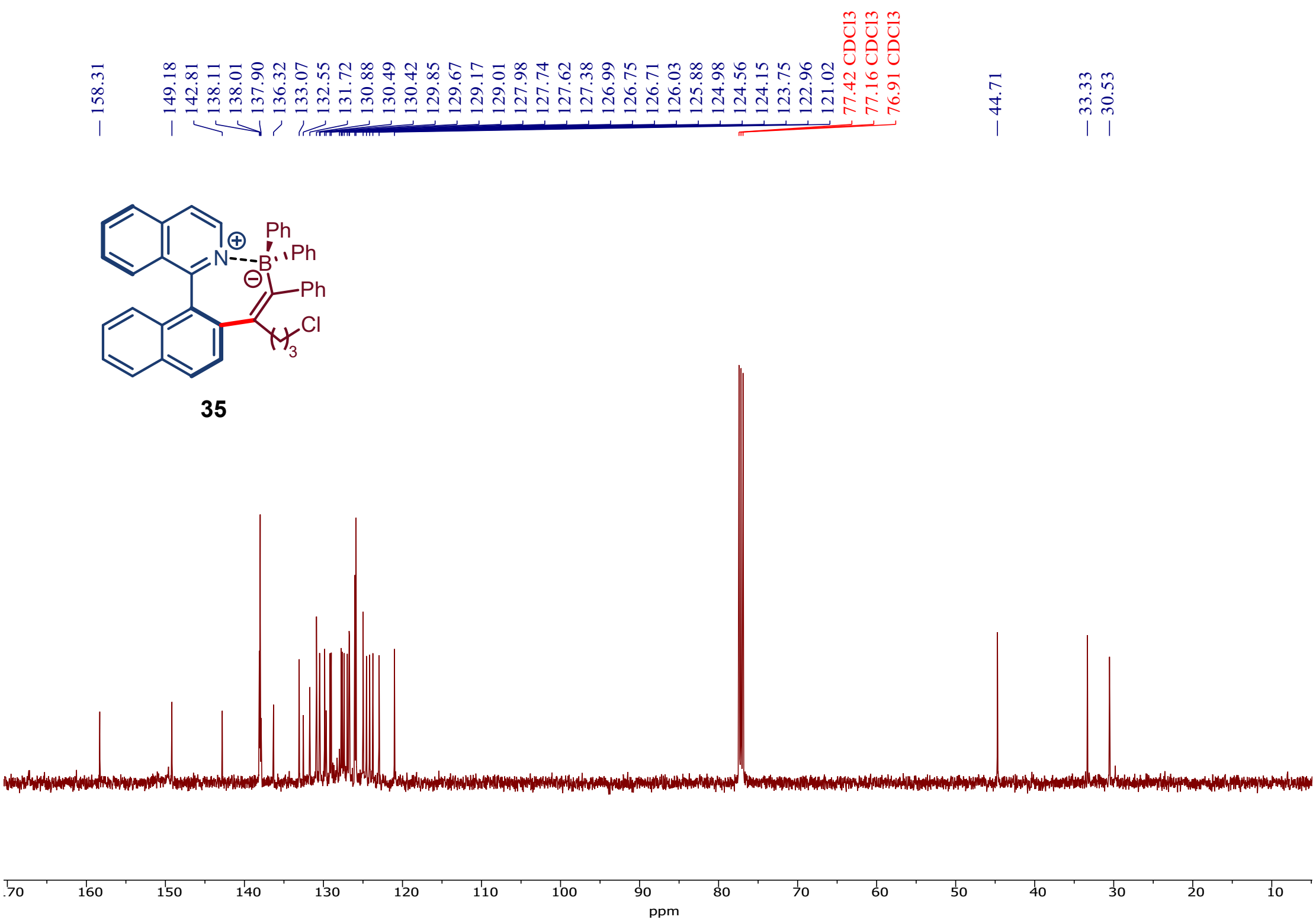

^{11}B NMR (128 MHz, $CDCl_3$) of **35**

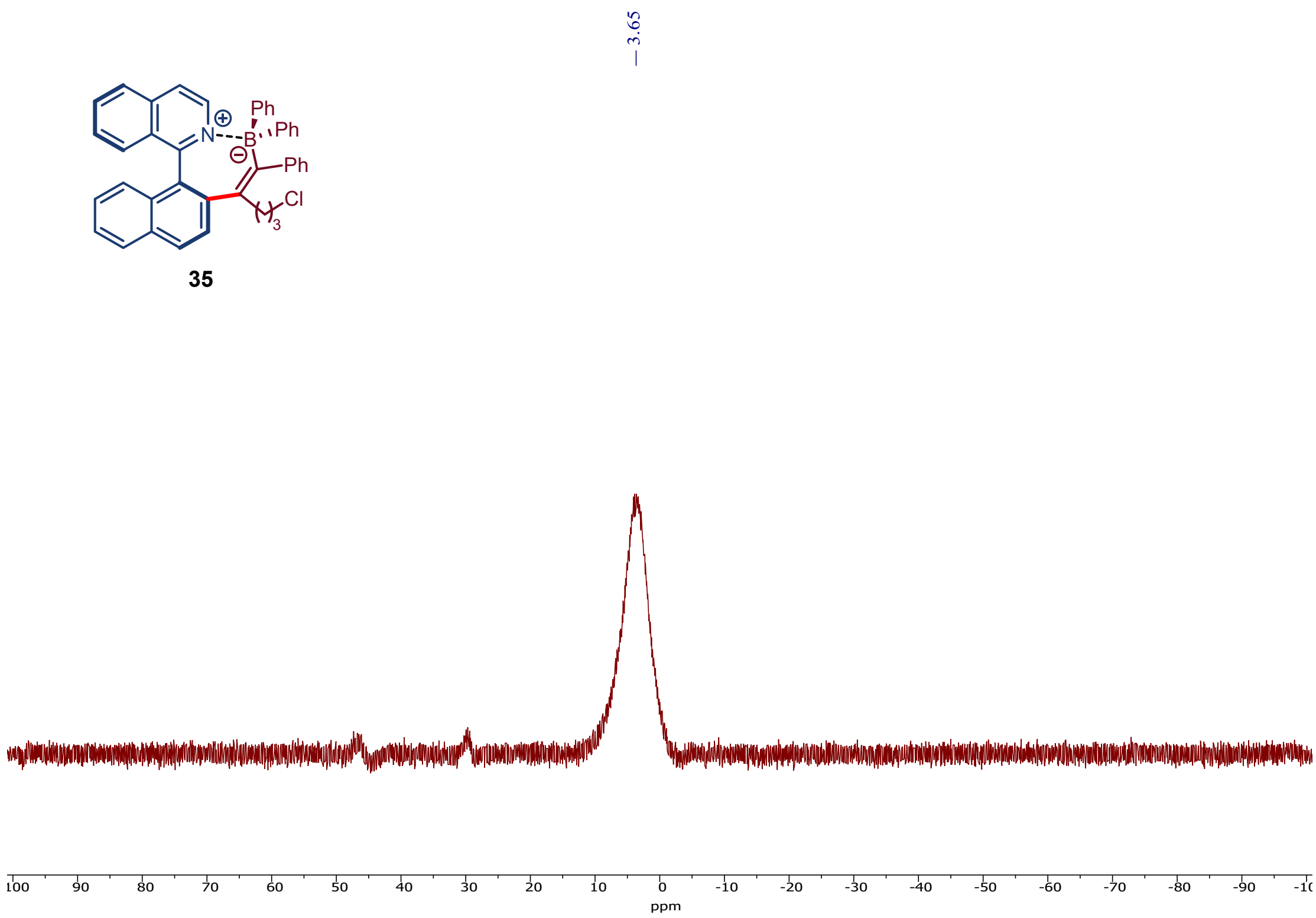

^{1}H NMR (400 MHz, $CDCl_3$) of **36** (*see procedure*)

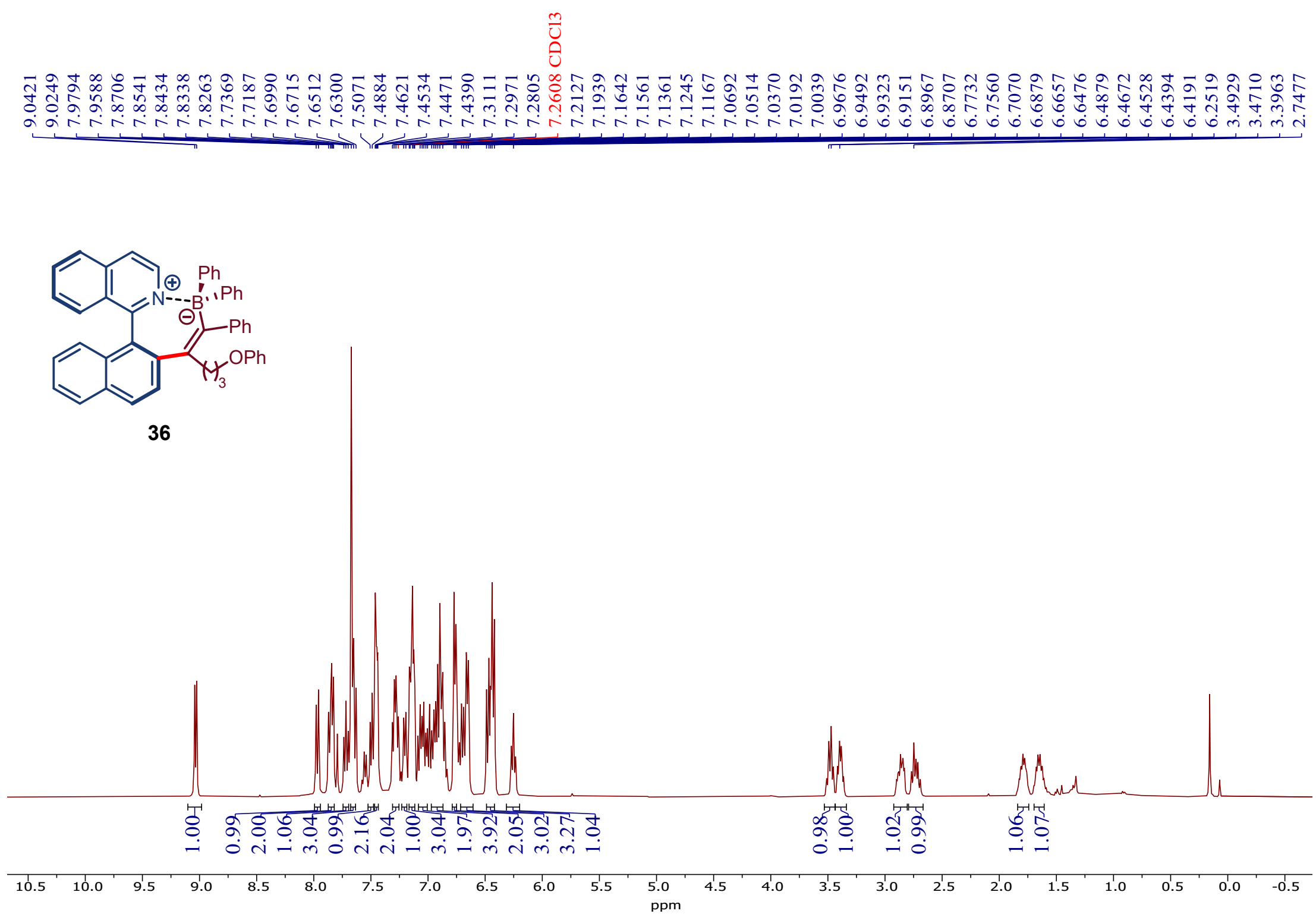

^{13}C NMR (100 MHz, $CDCl_3$) of **36**

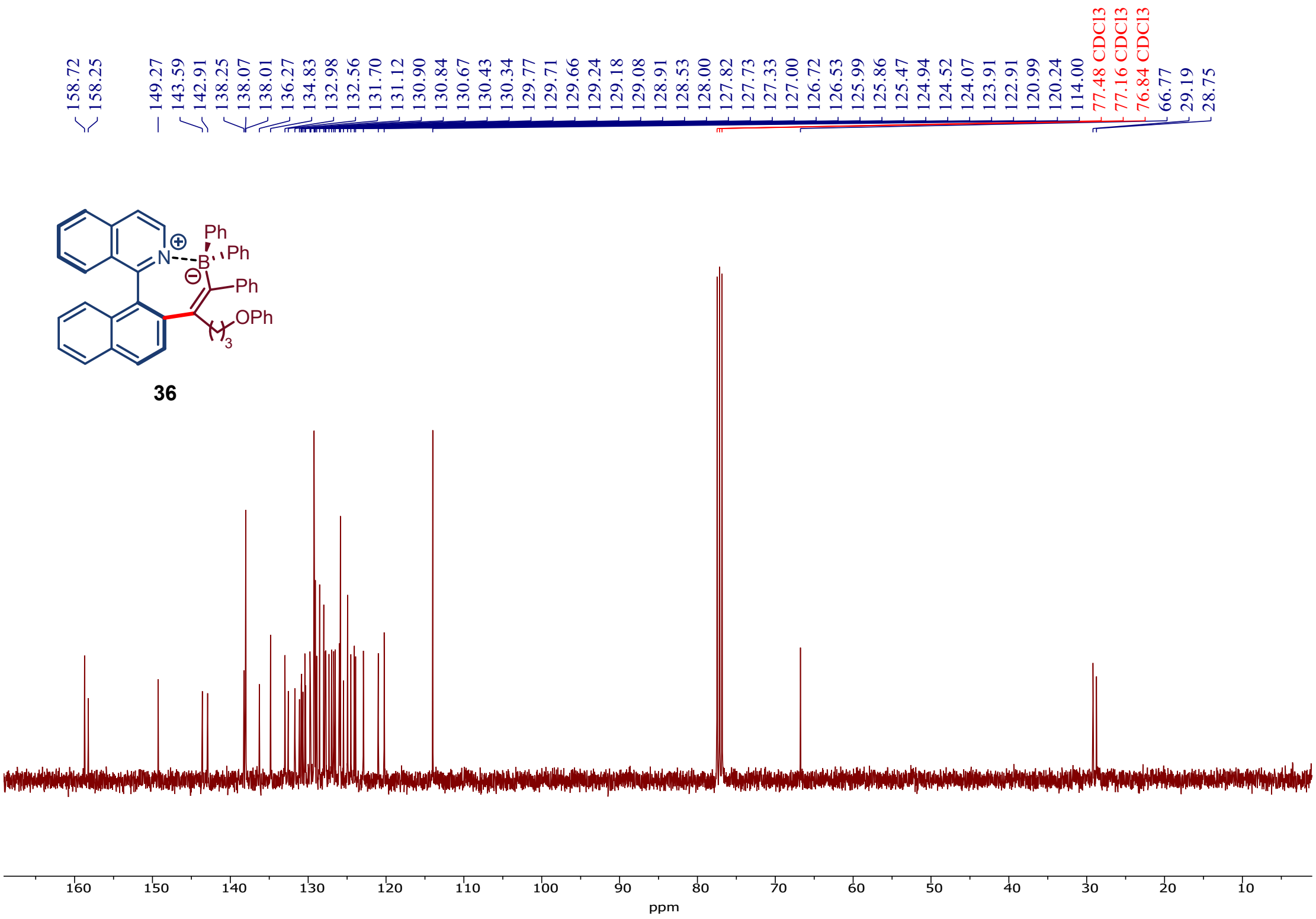

^{11}B NMR (128 MHz, $CDCl_3$) of **36**

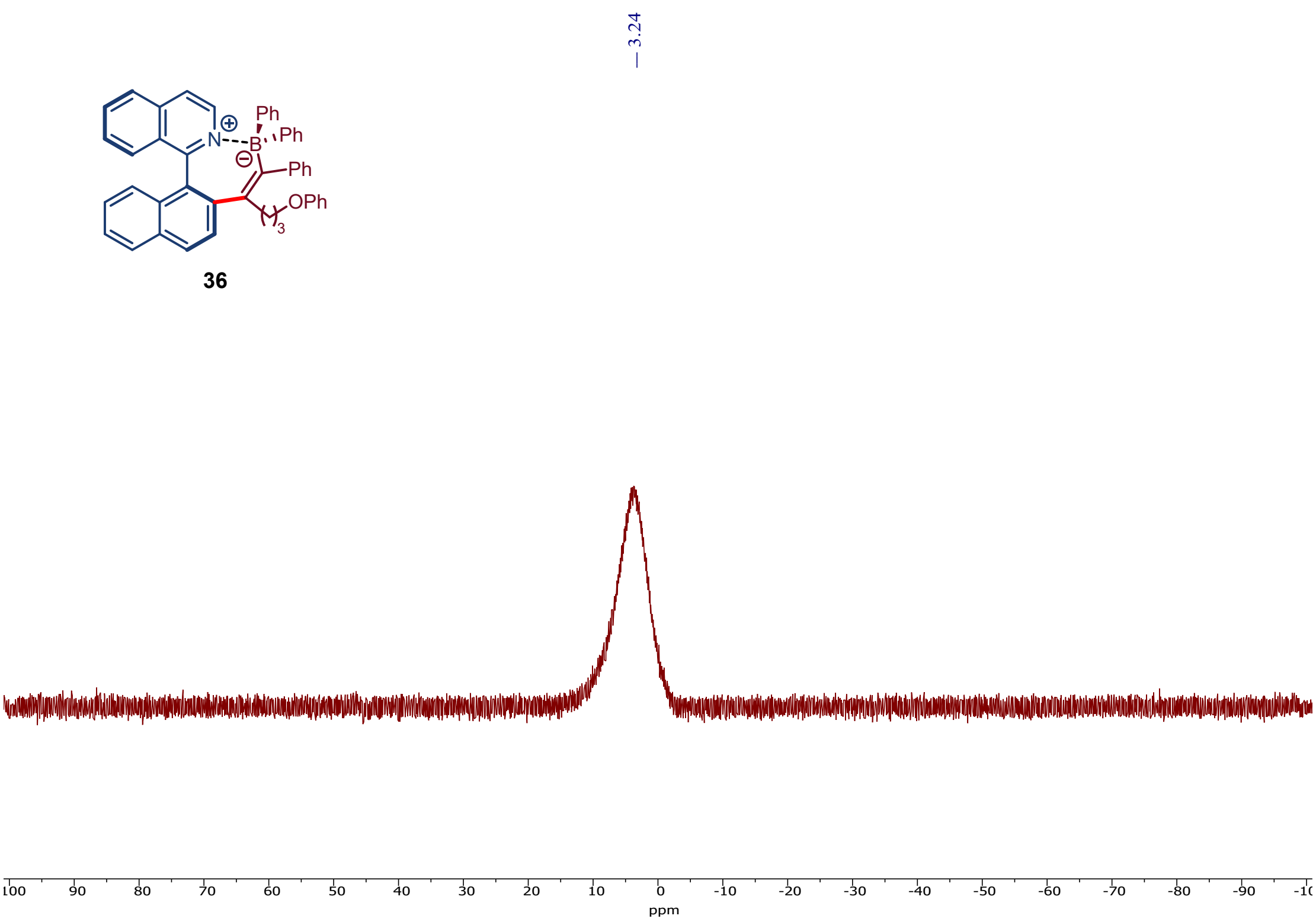

^{1}H NMR (400 MHz, $CDCl_3$) of **37** (*see procedure*)

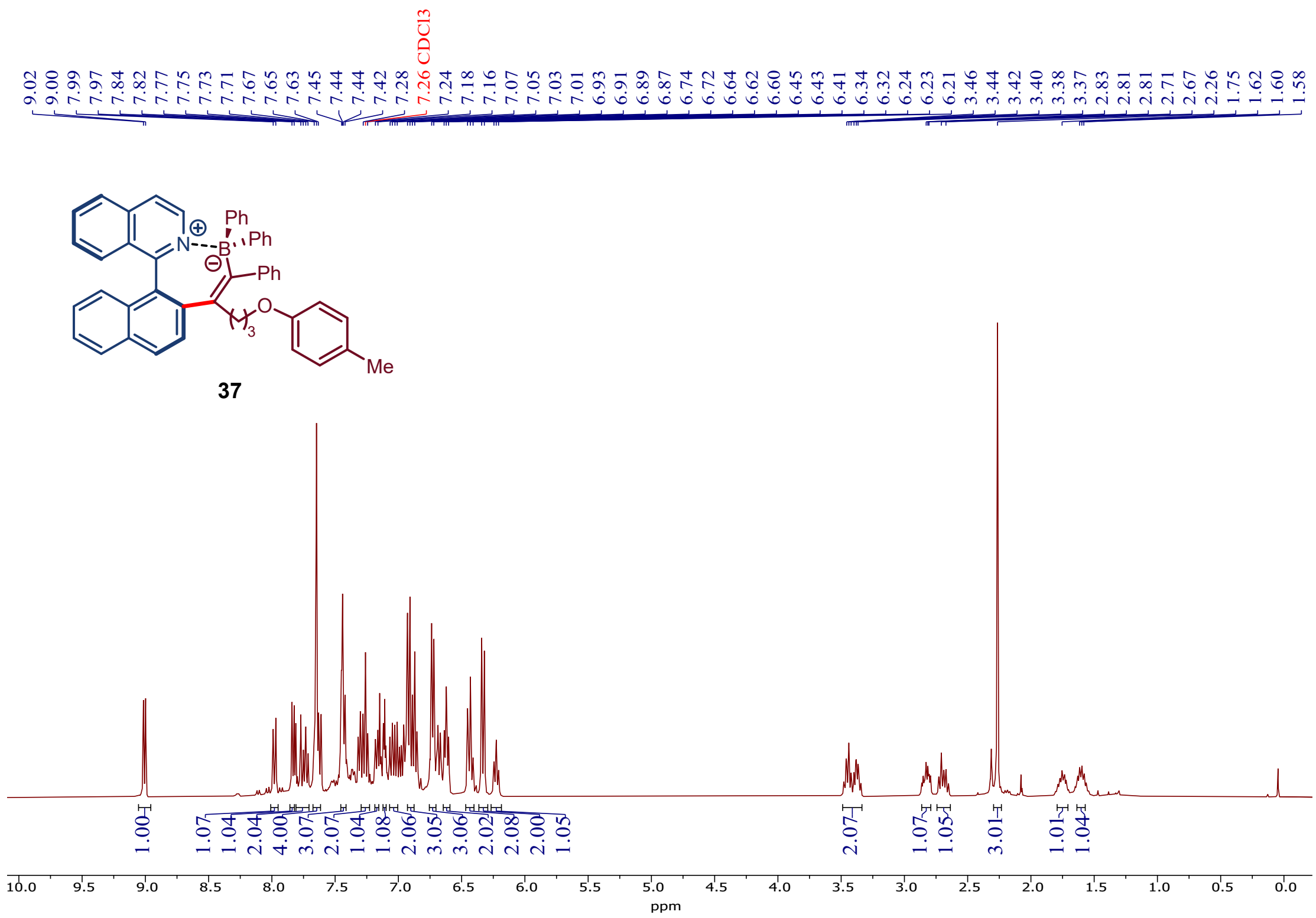

^{13}C NMR (100 MHz, $CDCl_3$) of **37**

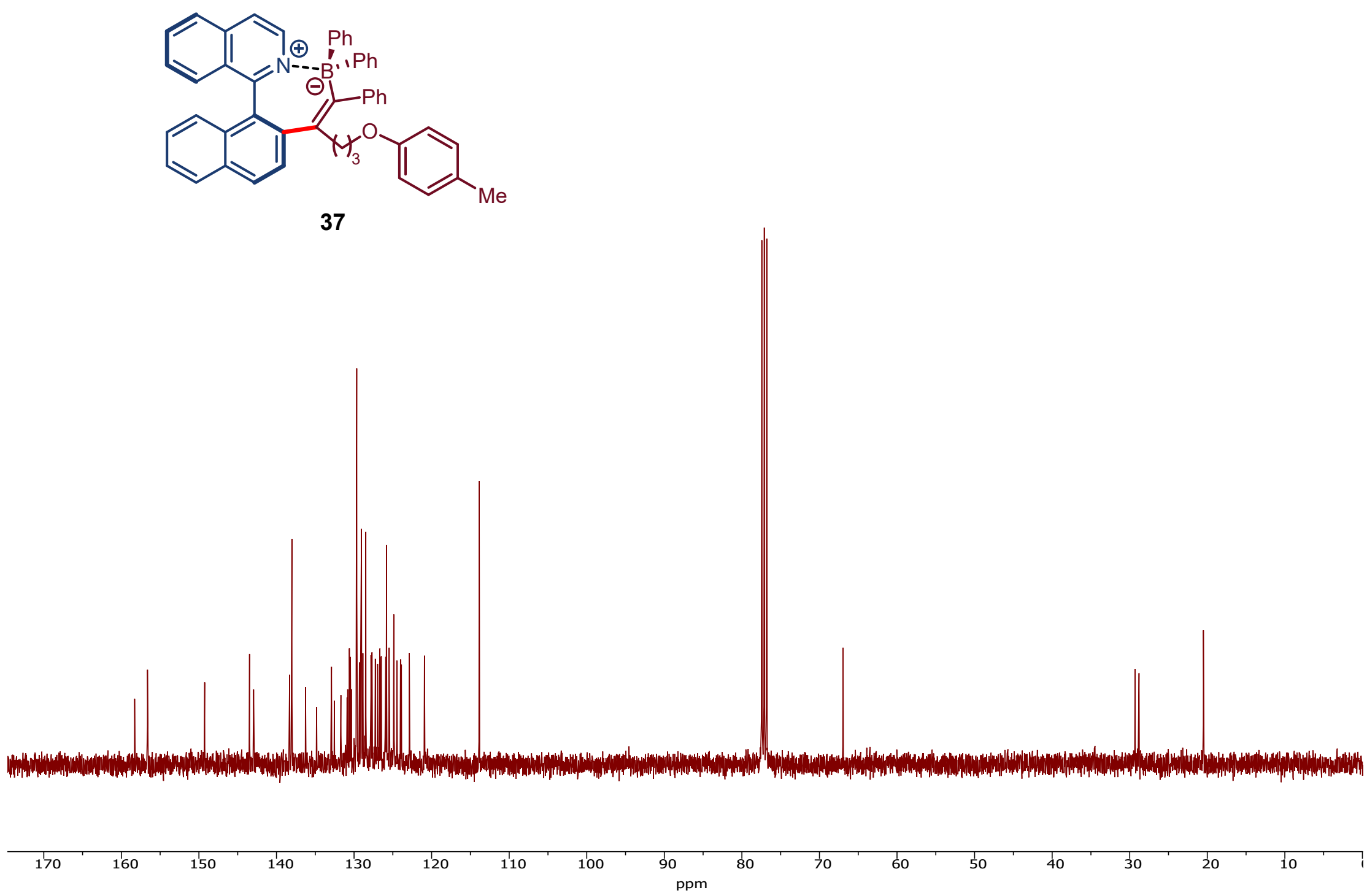

^{11}B NMR (128 MHz, $CDCl_3$) of **37**

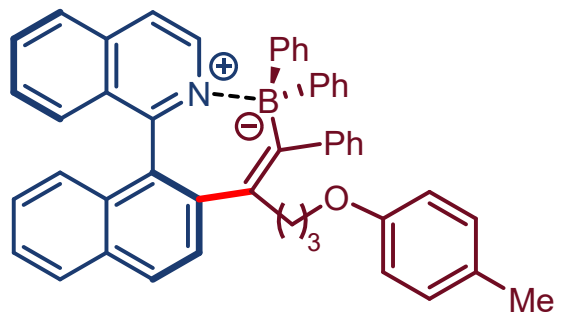

37

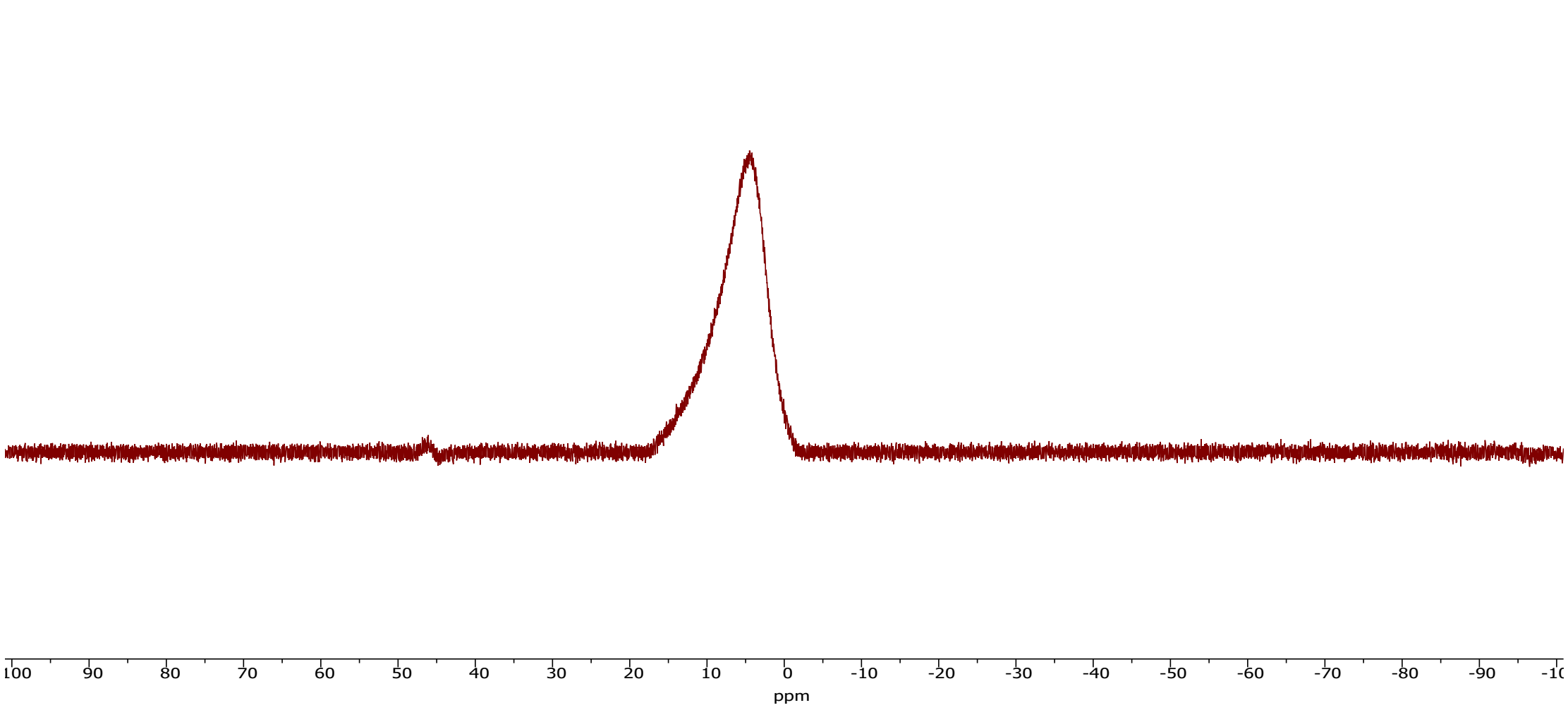

^{1}H NMR (400 MHz, $CDCl_3$) of **38** (*see procedure*)

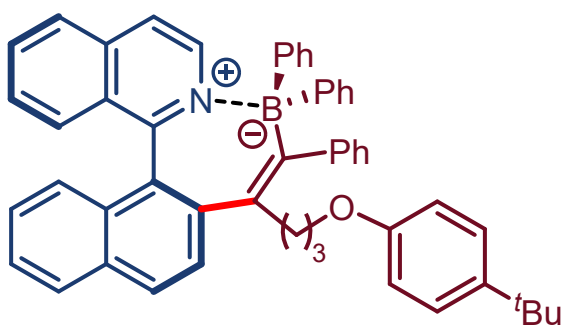

38

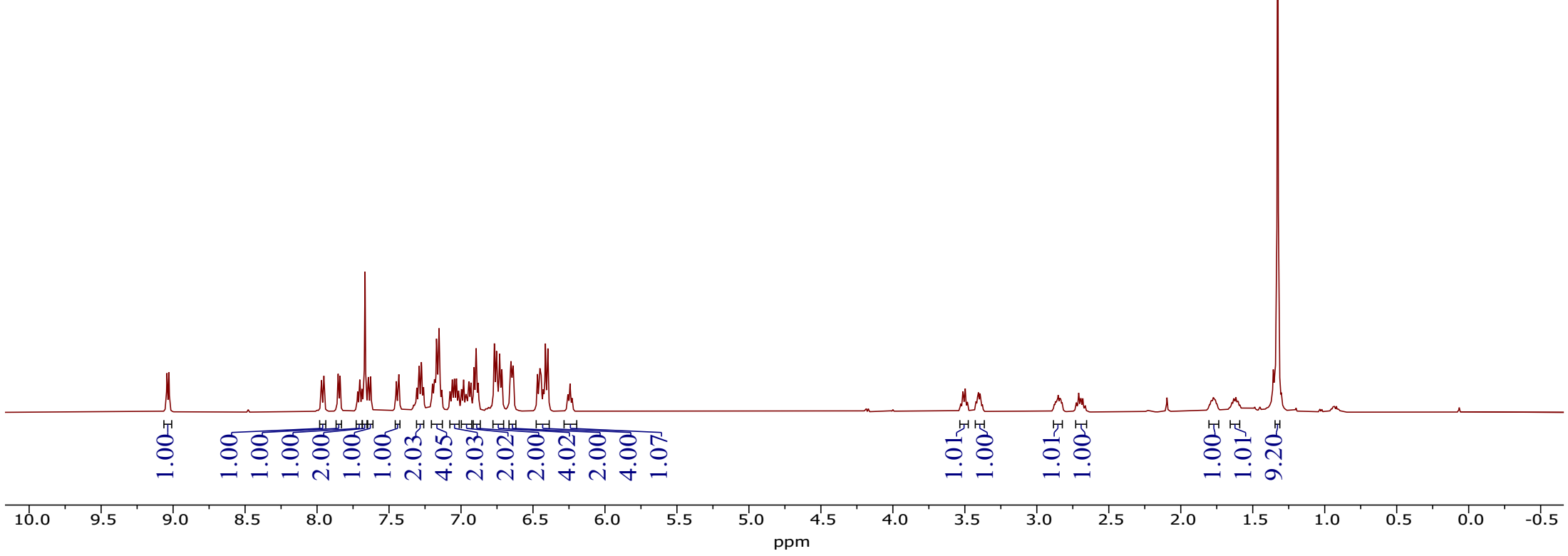

^{13}C NMR (100 MHz, $CDCl_3$) of **38**

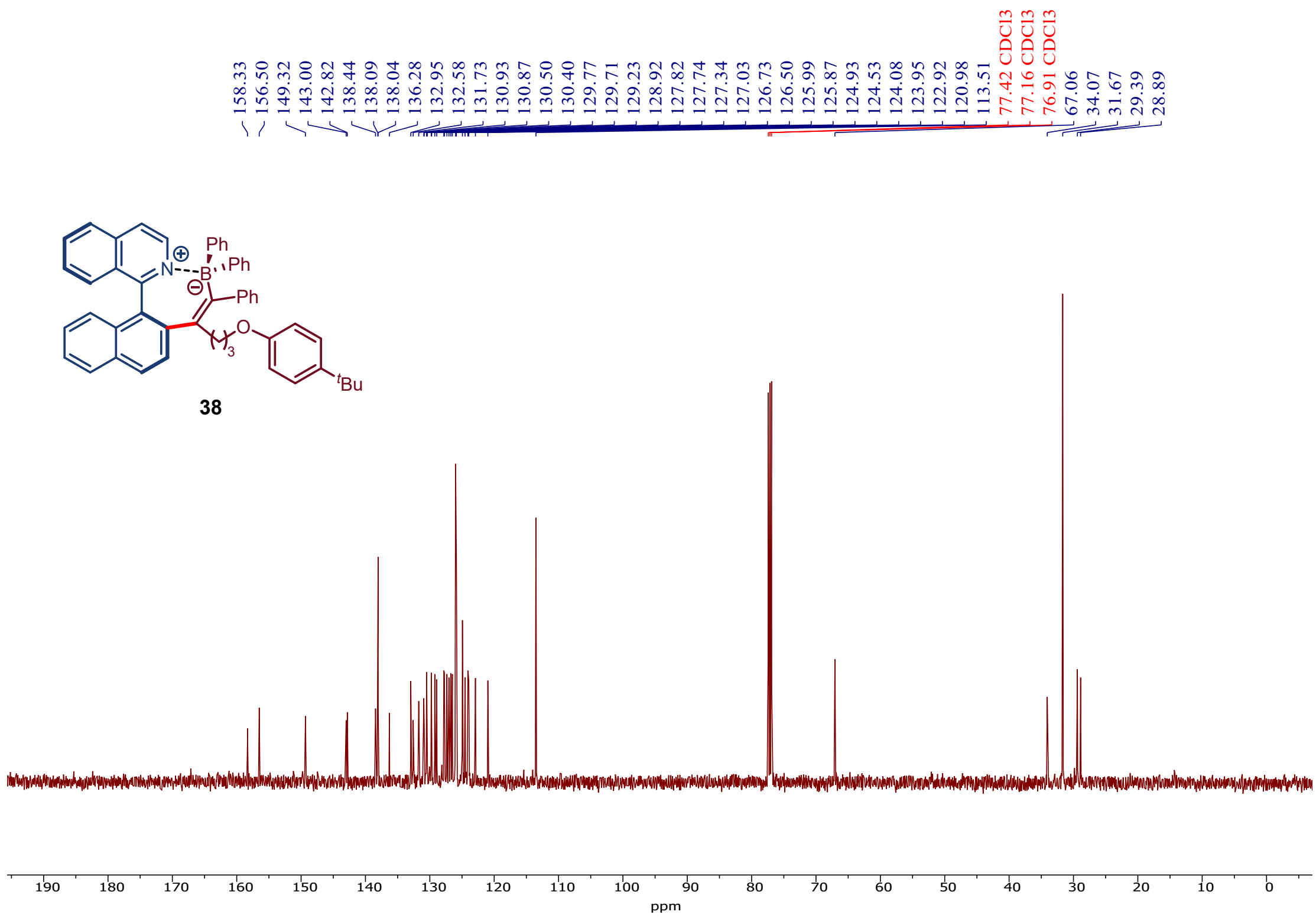

^{11}B NMR (128 MHz, $CDCl_3$) of **38**

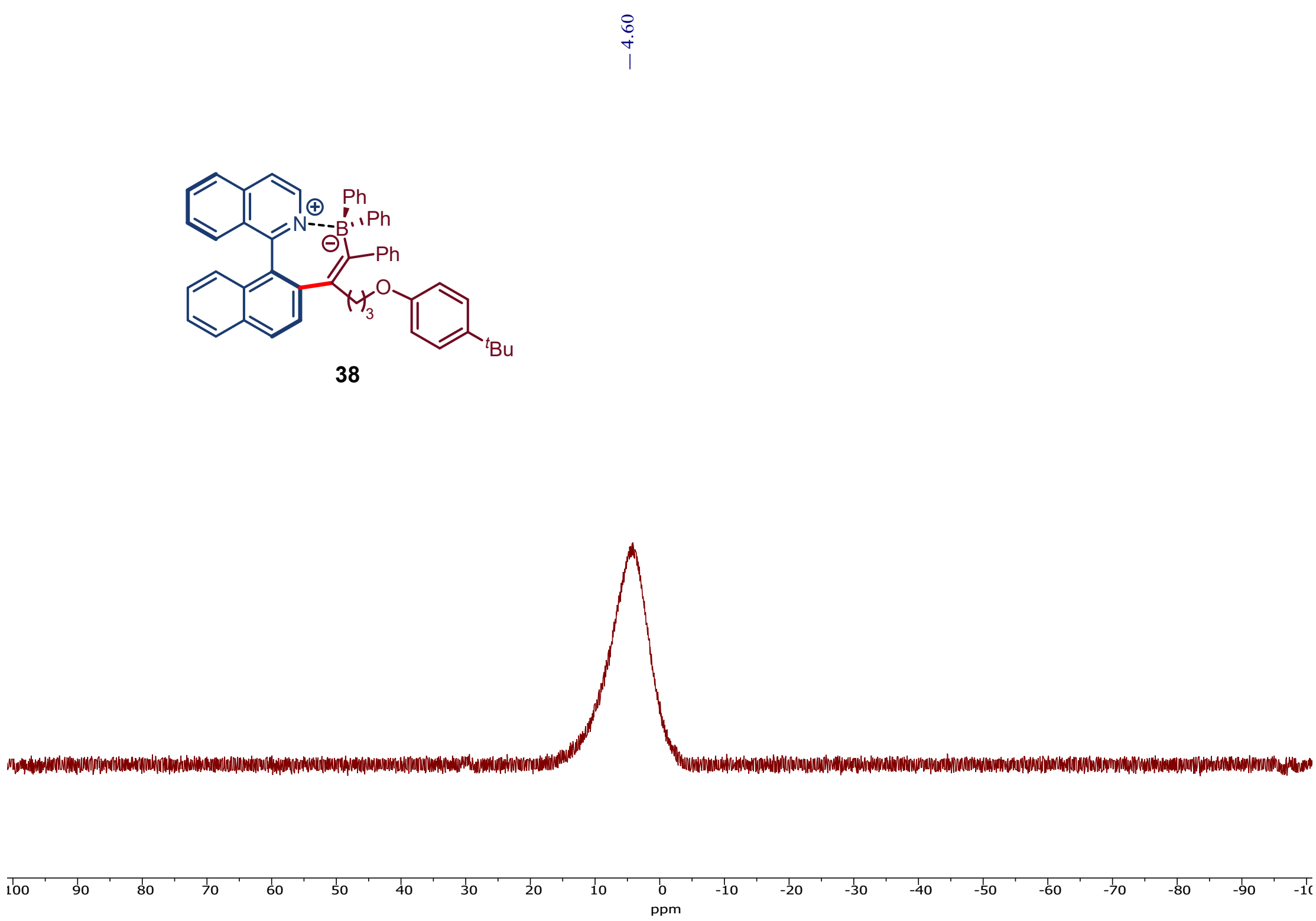

^{1}H NMR (400 MHz, $CDCl_3$) of **39** (*see procedure*)

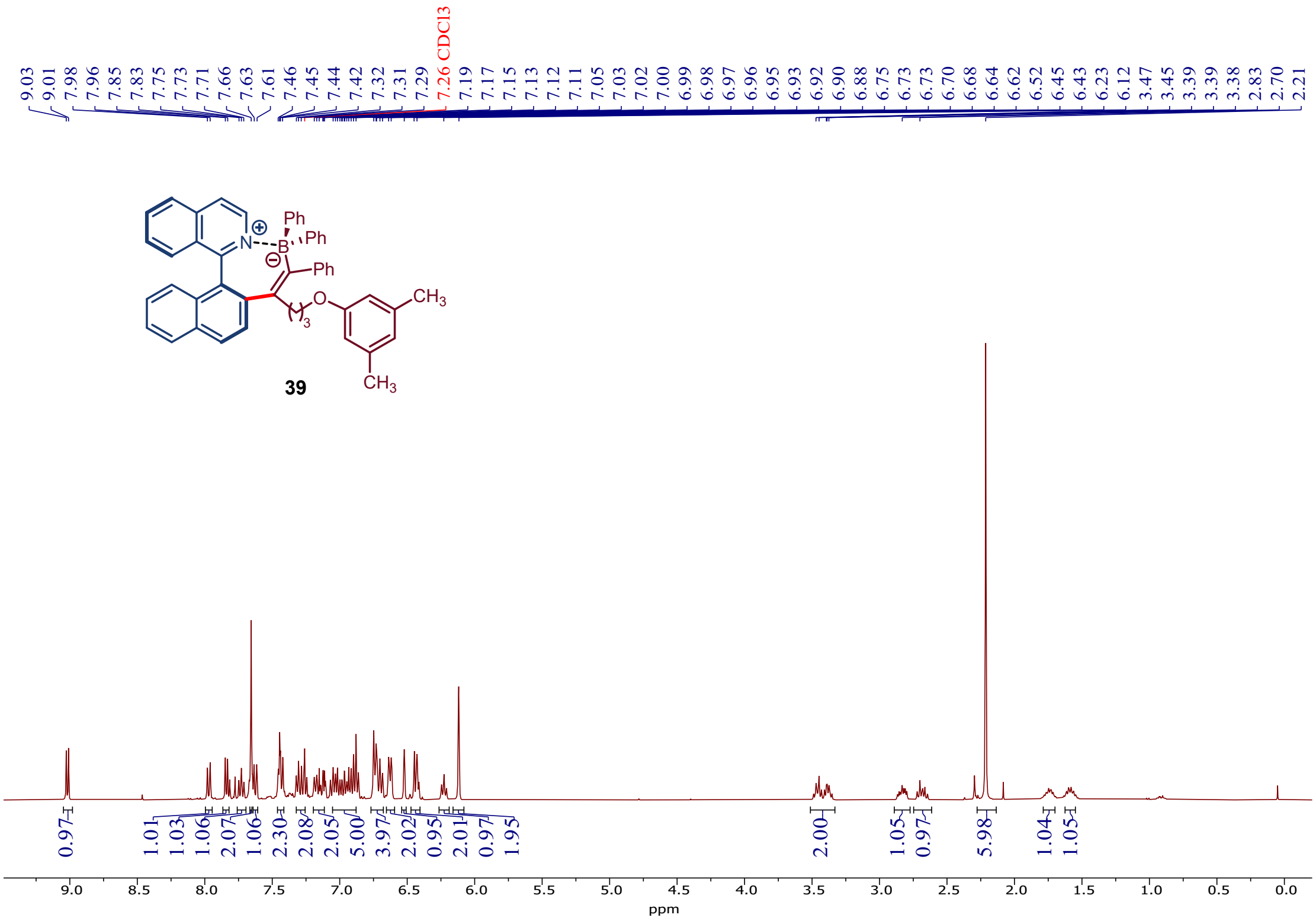

^{13}C NMR (100 MHz, $CDCl_3$) of **39**

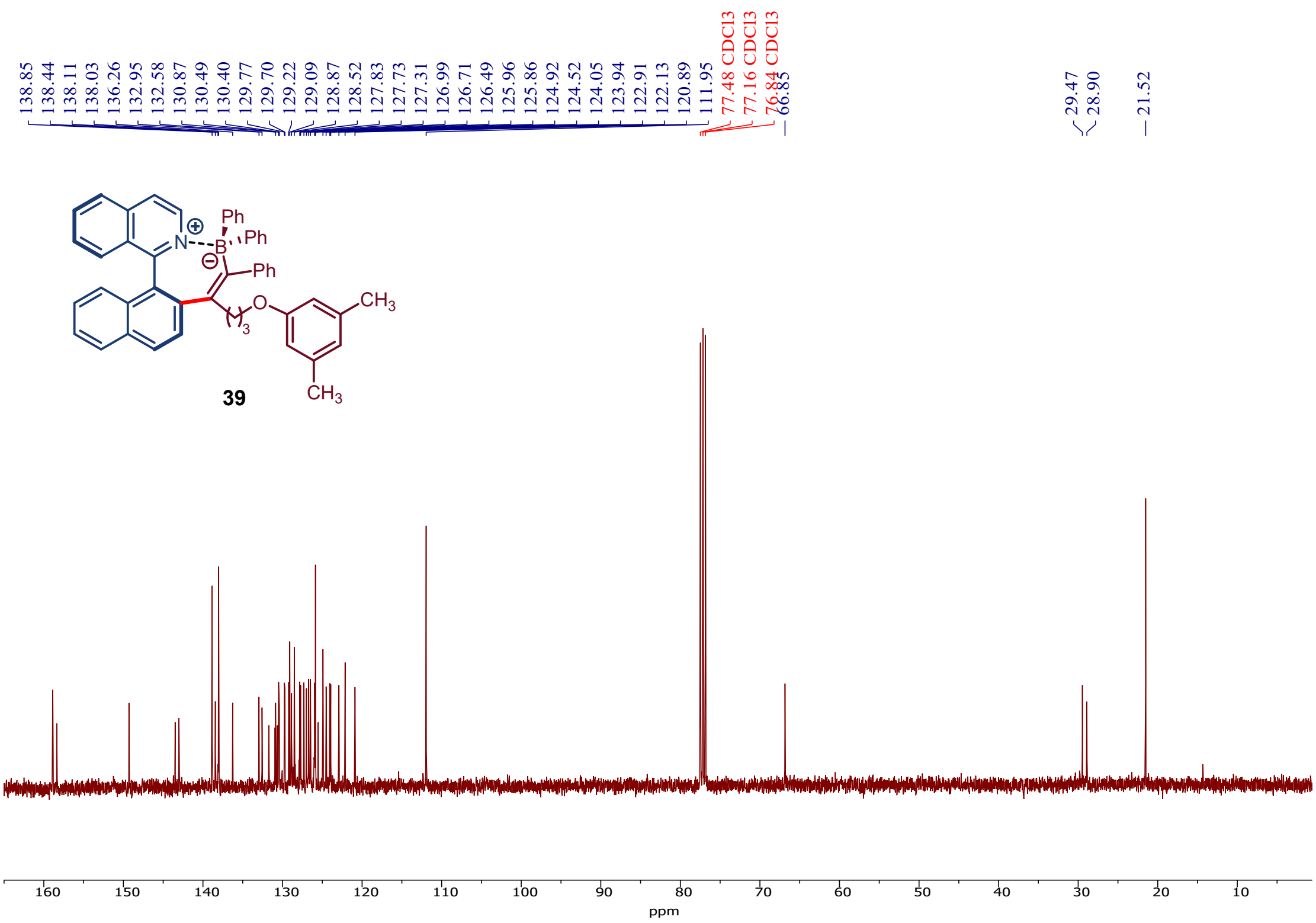

^{11}B NMR (128 MHz, $CDCl_3$) of **39**

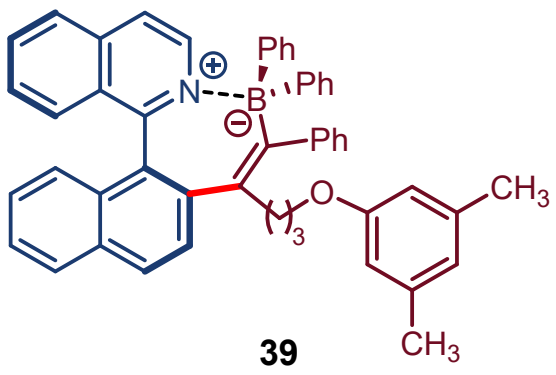

39

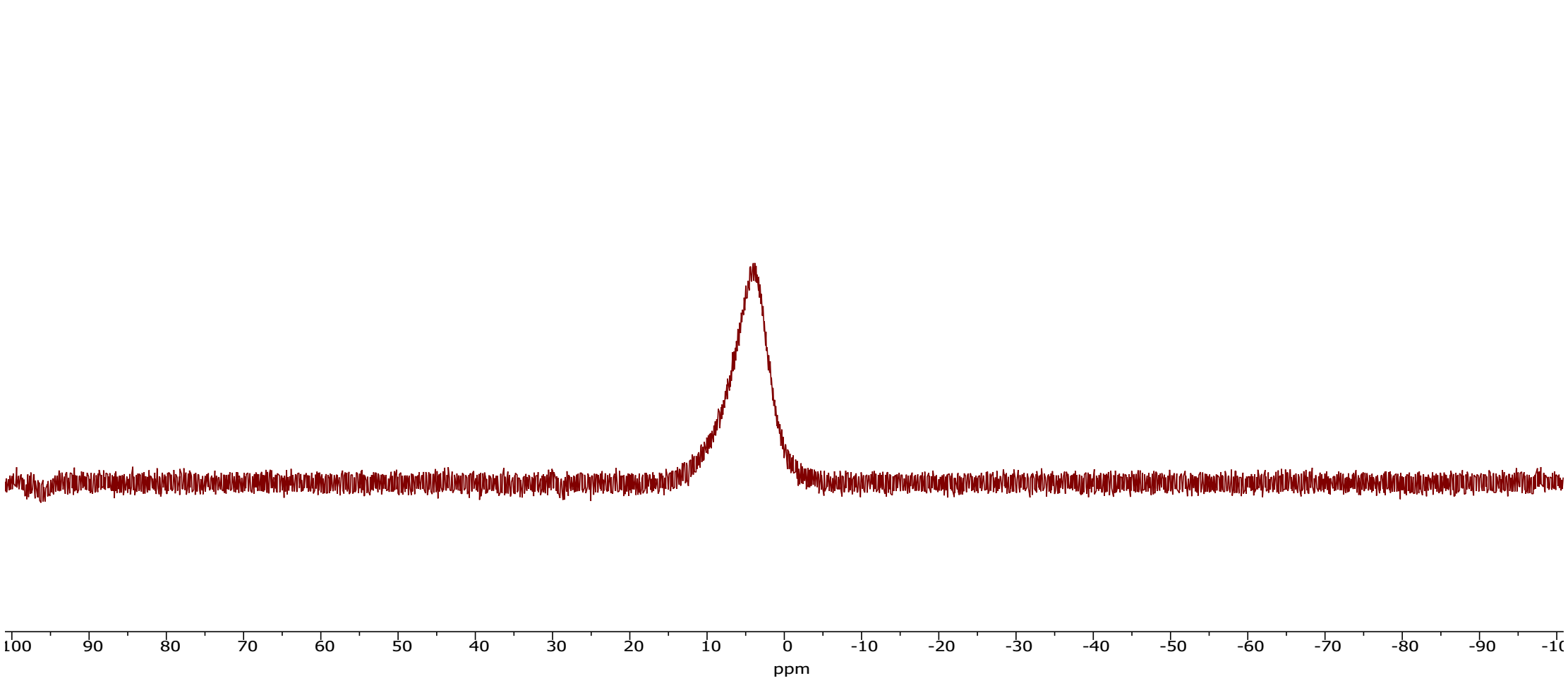

^{1}H NMR (400 MHz, $CDCl_3$) of **40** (*see procedure*)

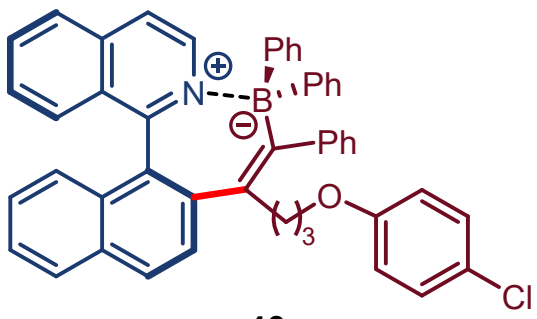

40

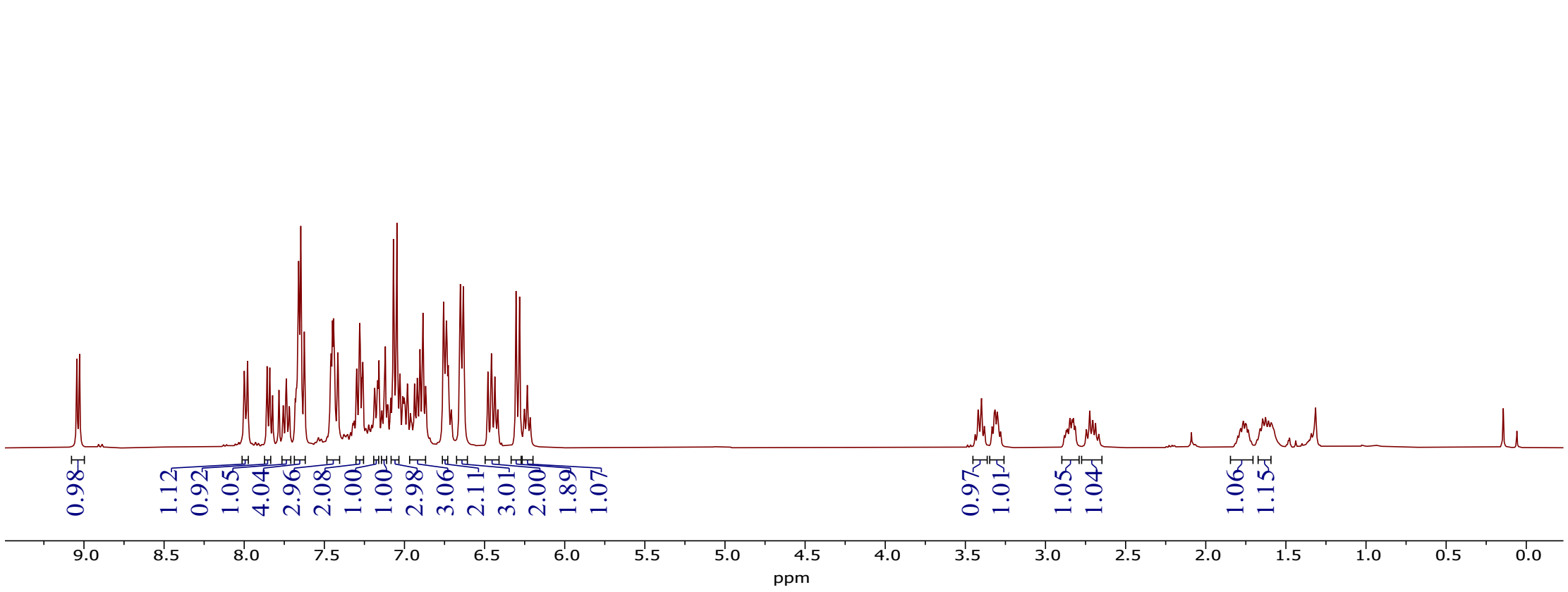

^{13}C NMR (100 MHz, $CDCl_3$) of **40**

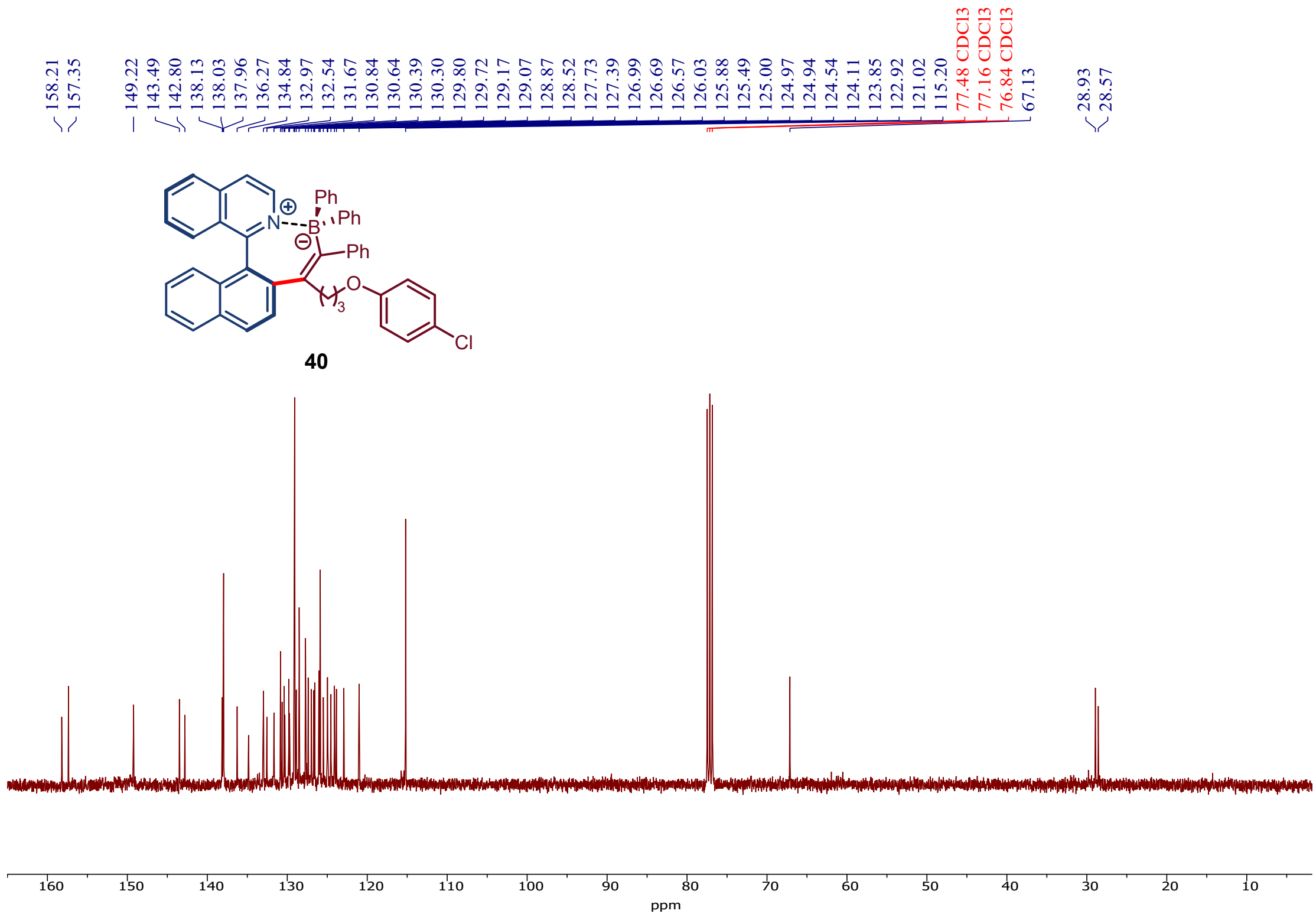

^{11}B NMR (128 MHz, $CDCl_3$) of **40**

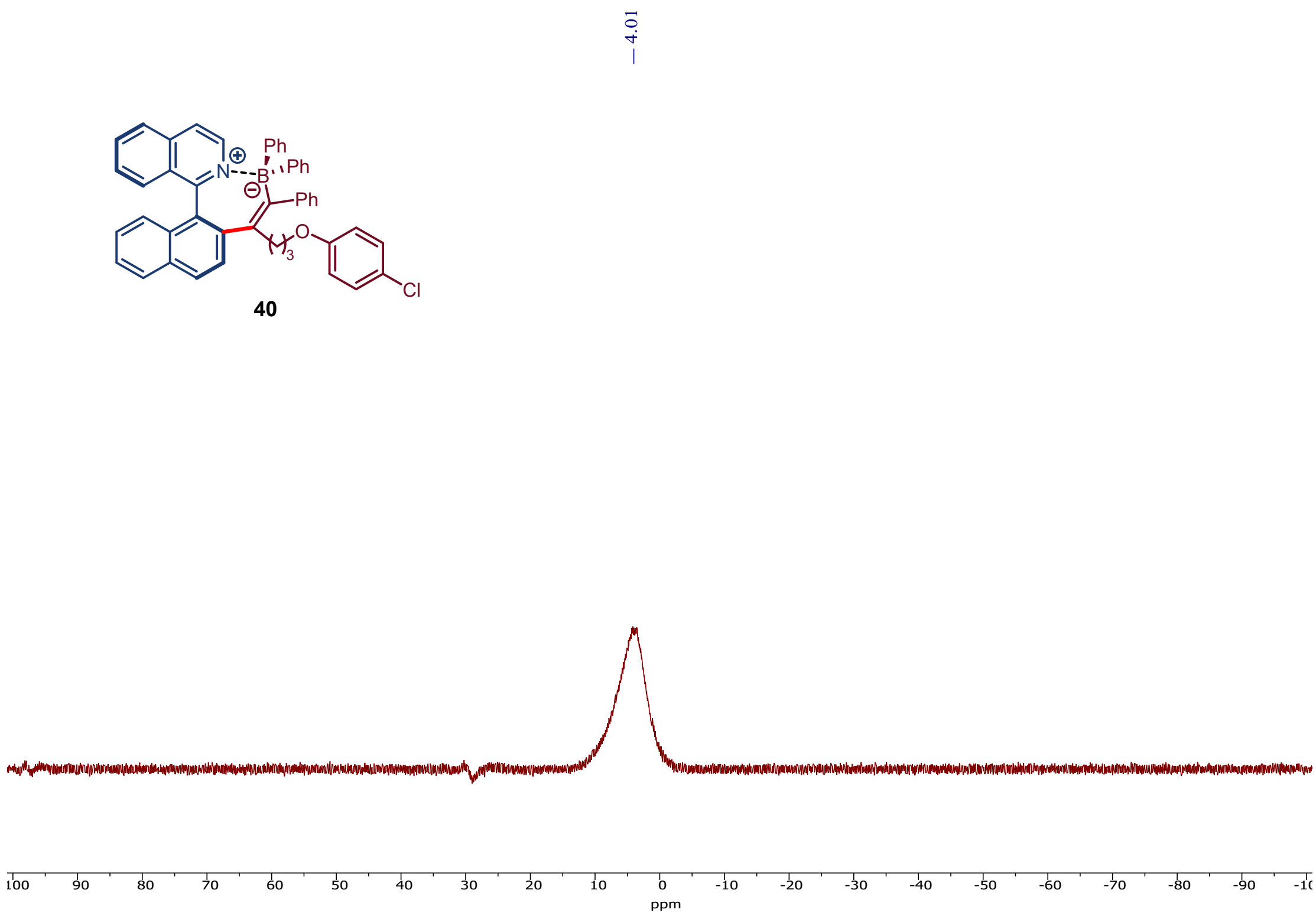

^{1}H NMR (400 MHz, $CDCl_3$) of **41** (*see procedure*)

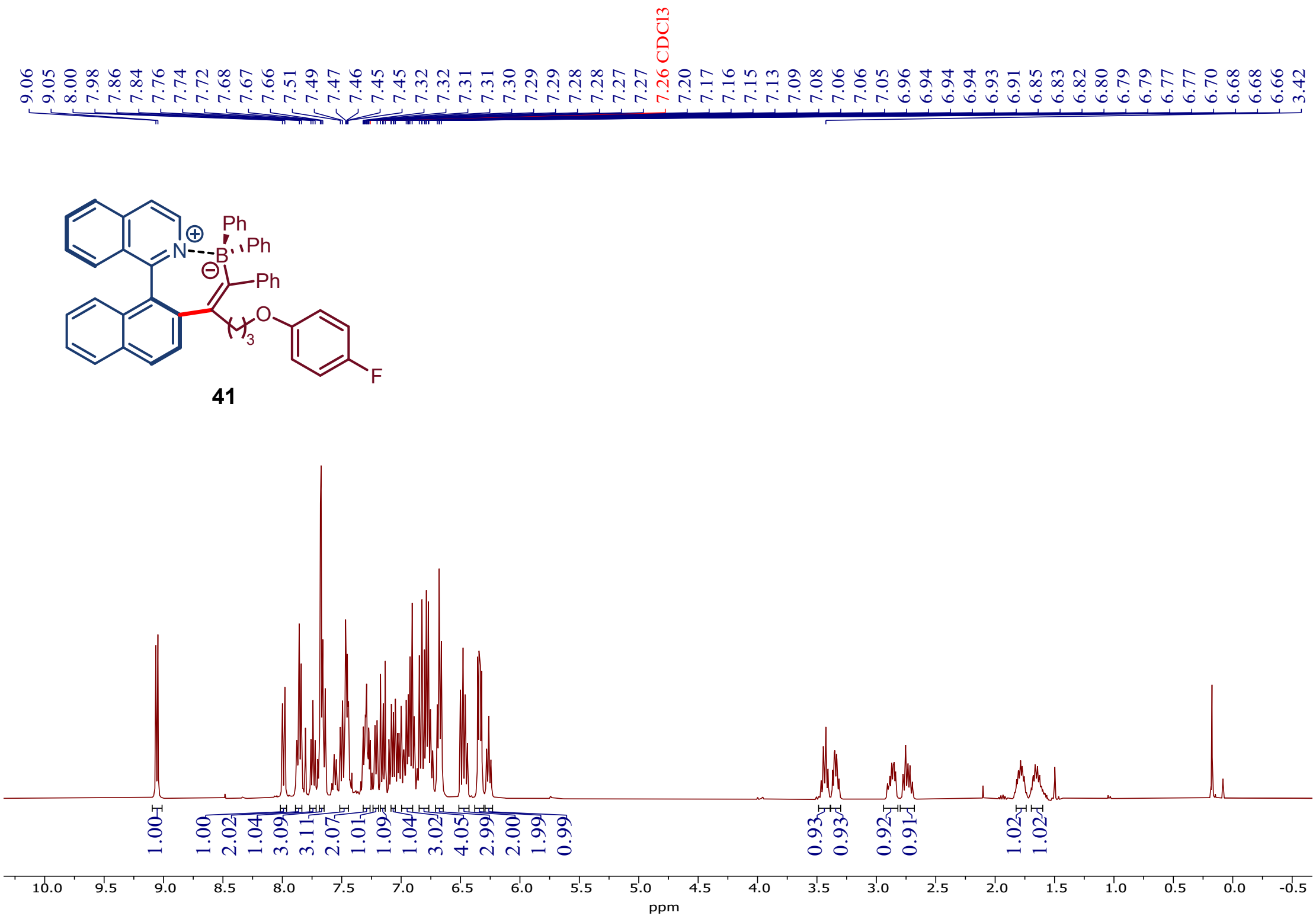

^{13}C NMR (100 MHz, $CDCl_3$) of **41**

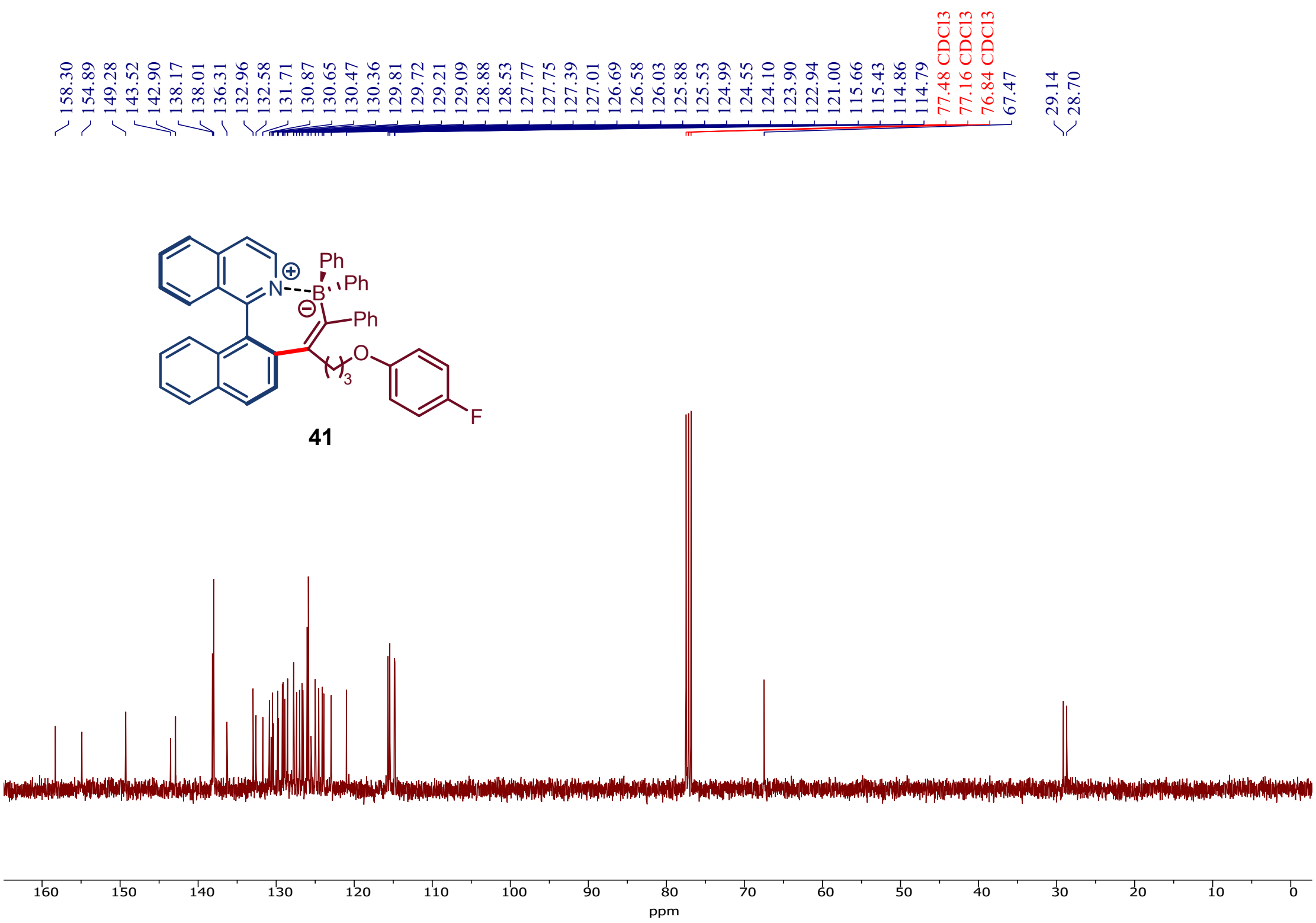

^{11}B NMR (128 MHz, $CDCl_3$) of **41**

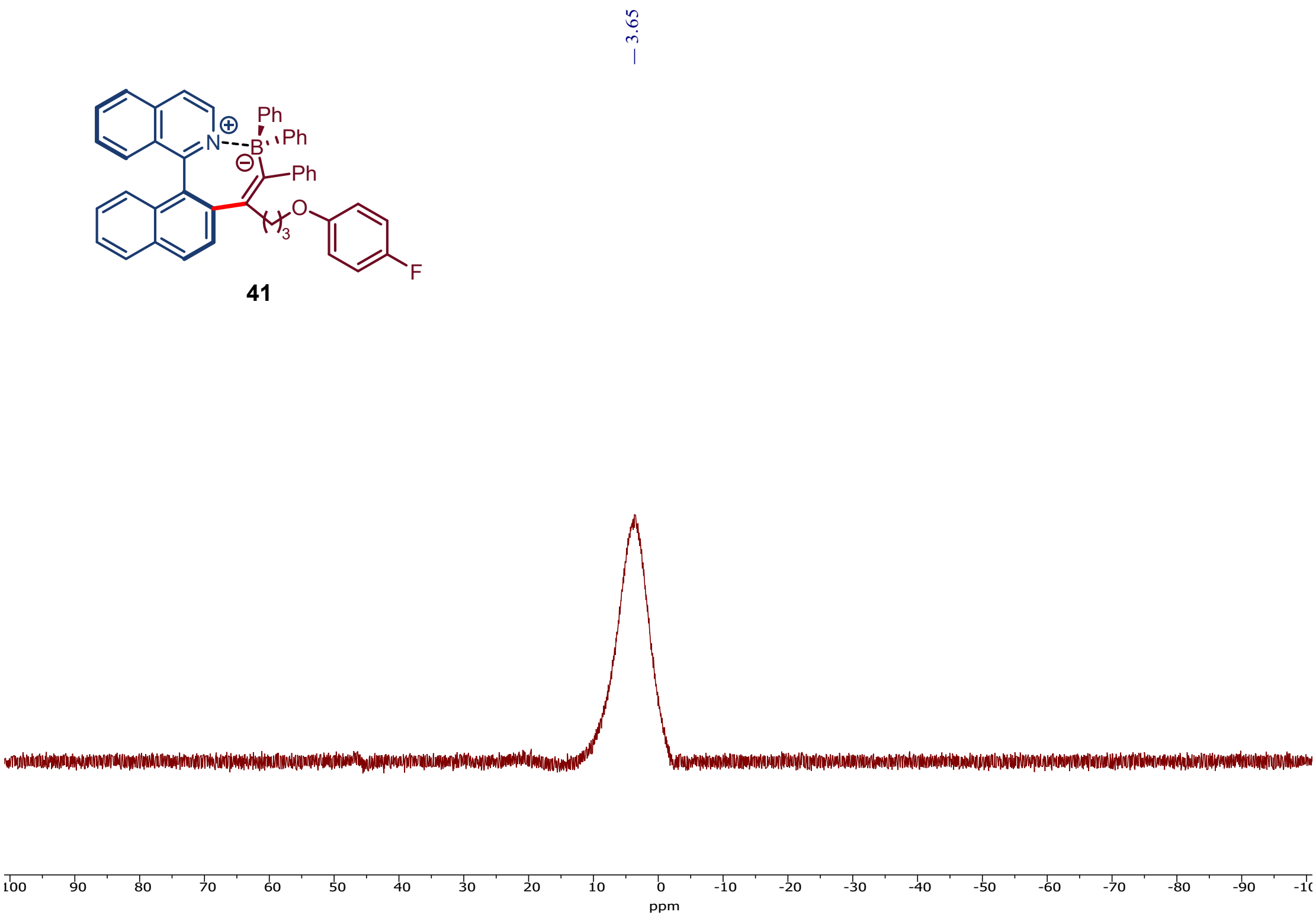

^{19}F NMR (376 MHz, $CDCl_3$) of **41**

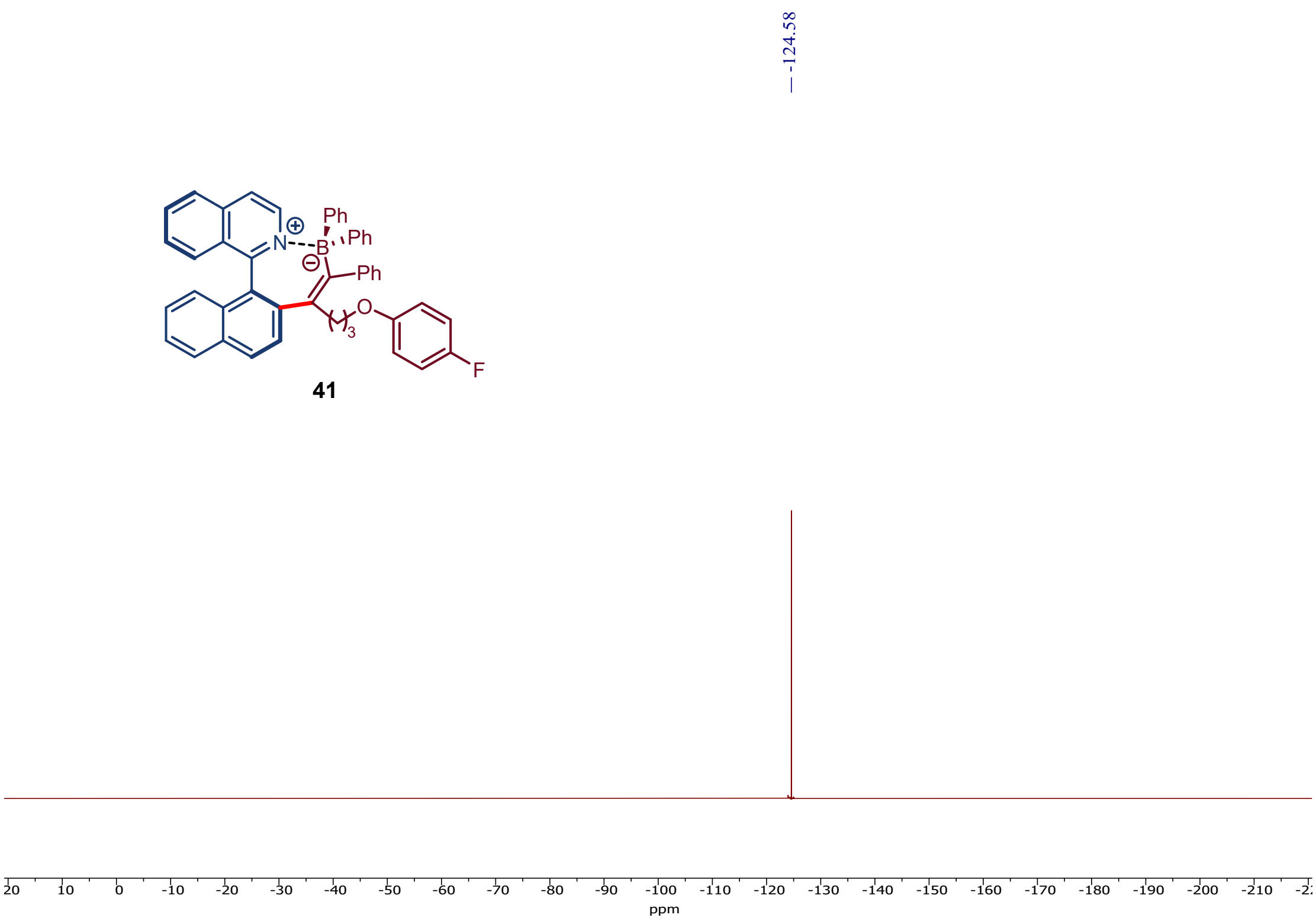

^{1}H NMR (400 MHz, $CDCl_3$) of **42** (*see procedure*)

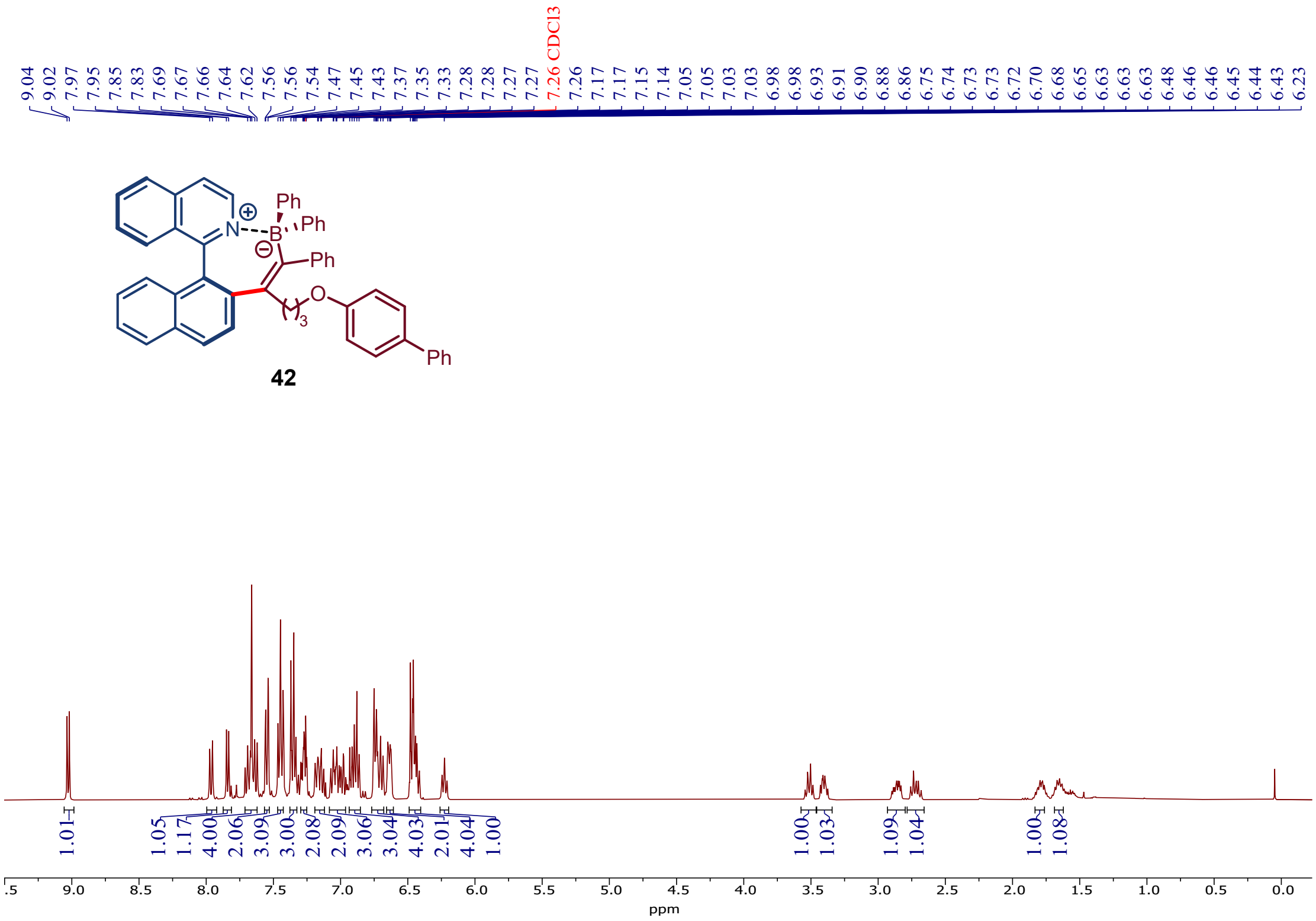

^{13}C NMR (100 MHz, $CDCl_3$) of **42**

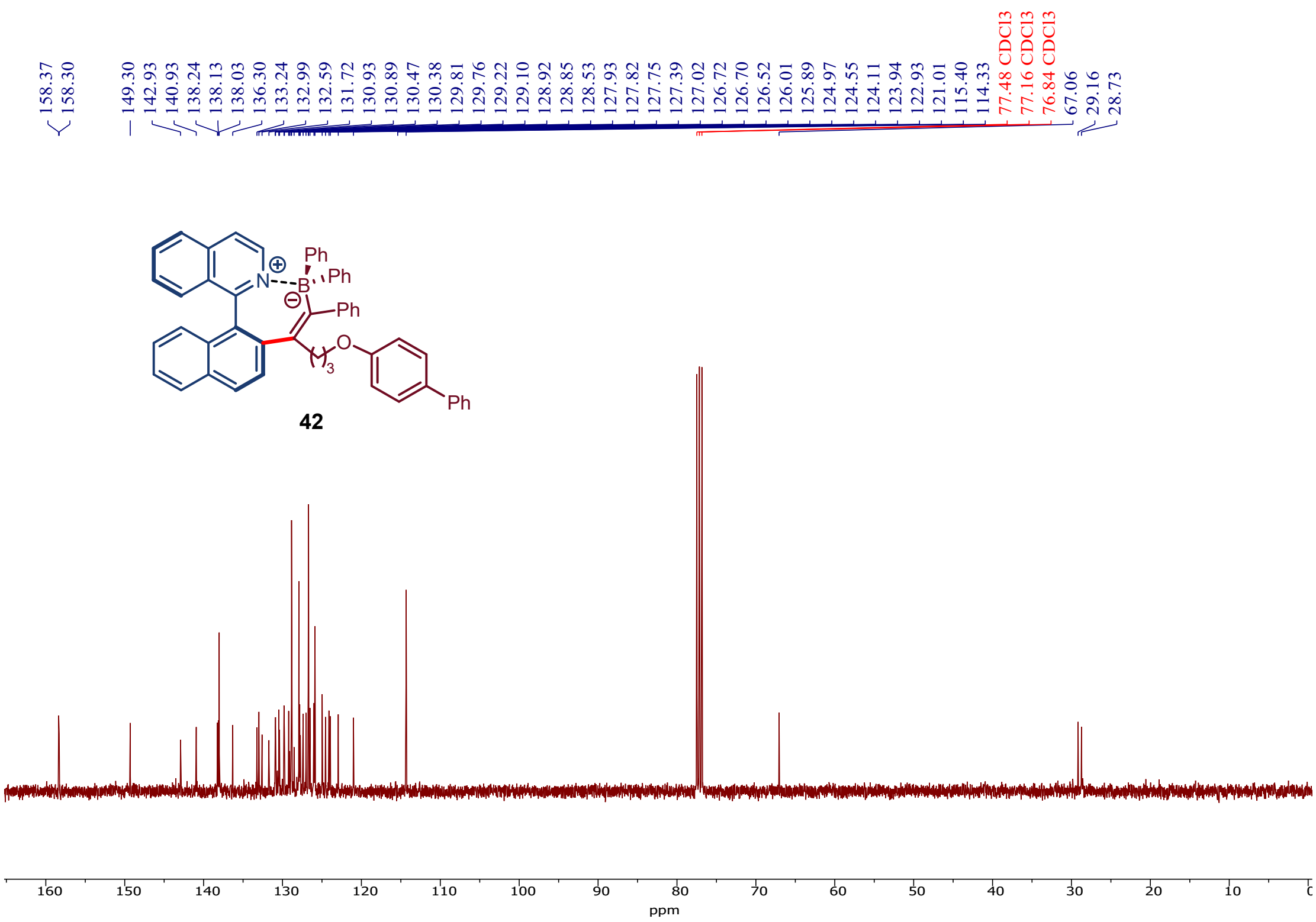

^{11}B NMR (128 MHz, $CDCl_3$) of **42**

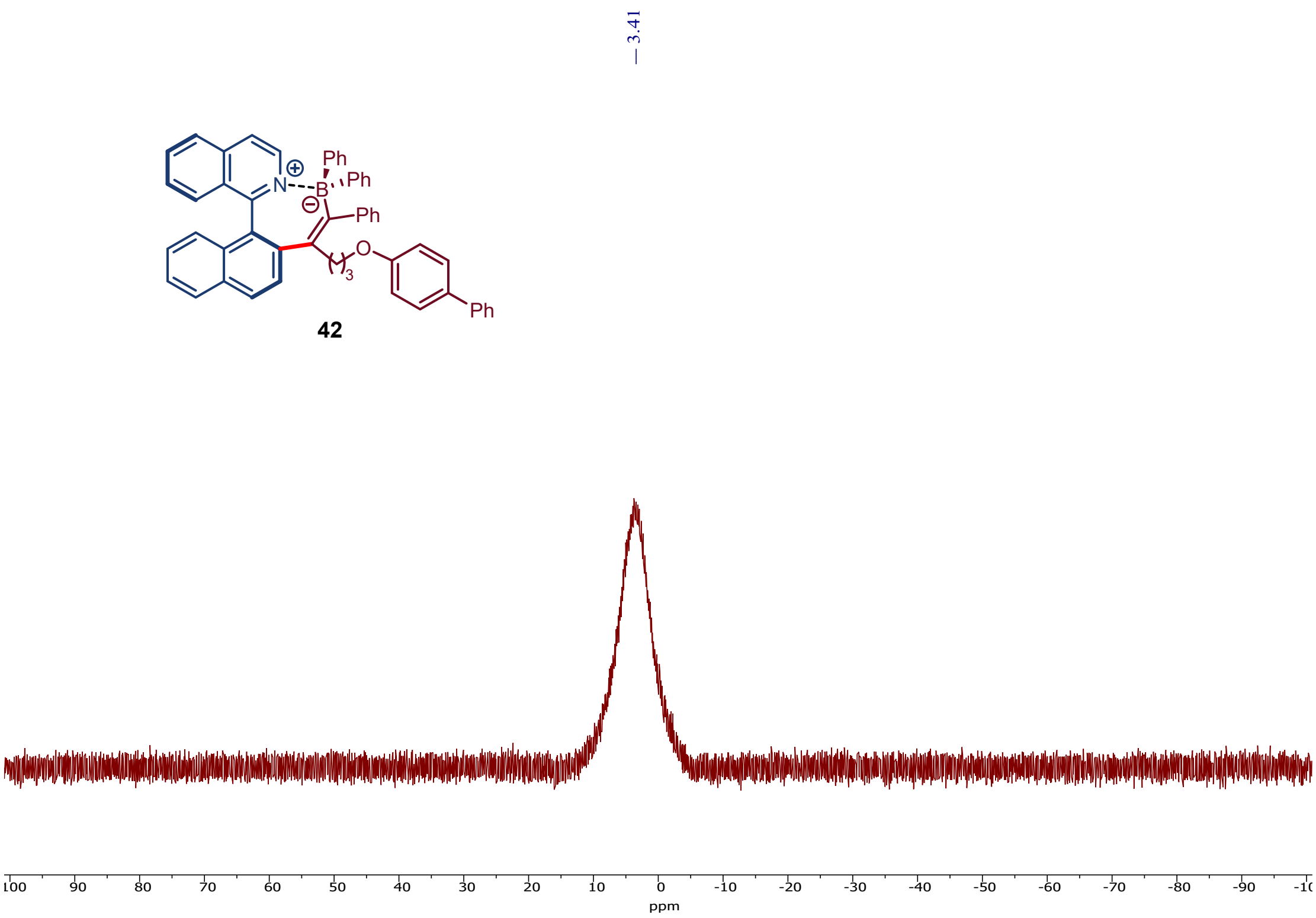

^{1}H NMR (400 MHz, $CDCl_3$) of **43** (*see procedure*)

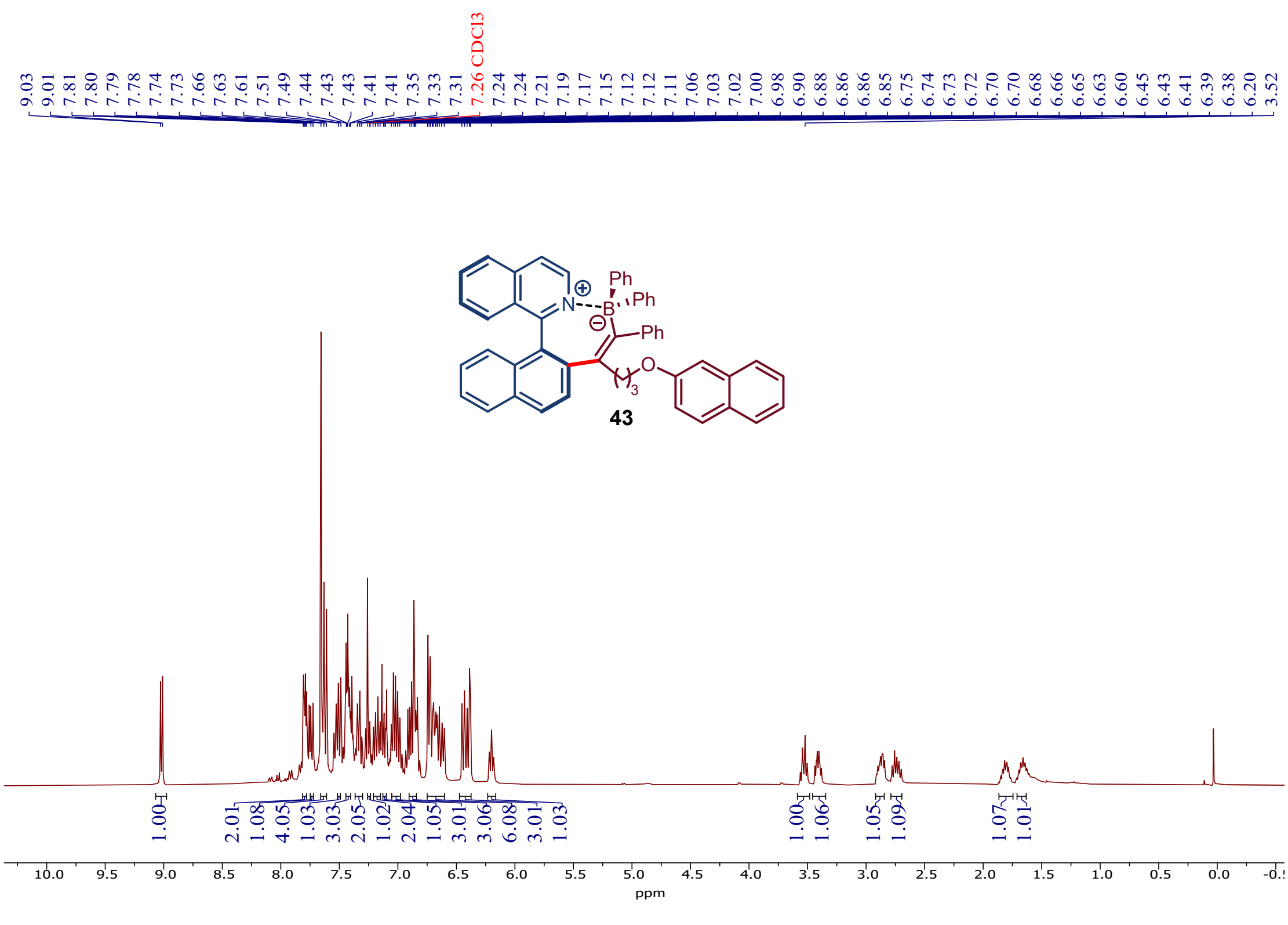

^{13}C NMR (100 MHz, $CDCl_3$) of **43**

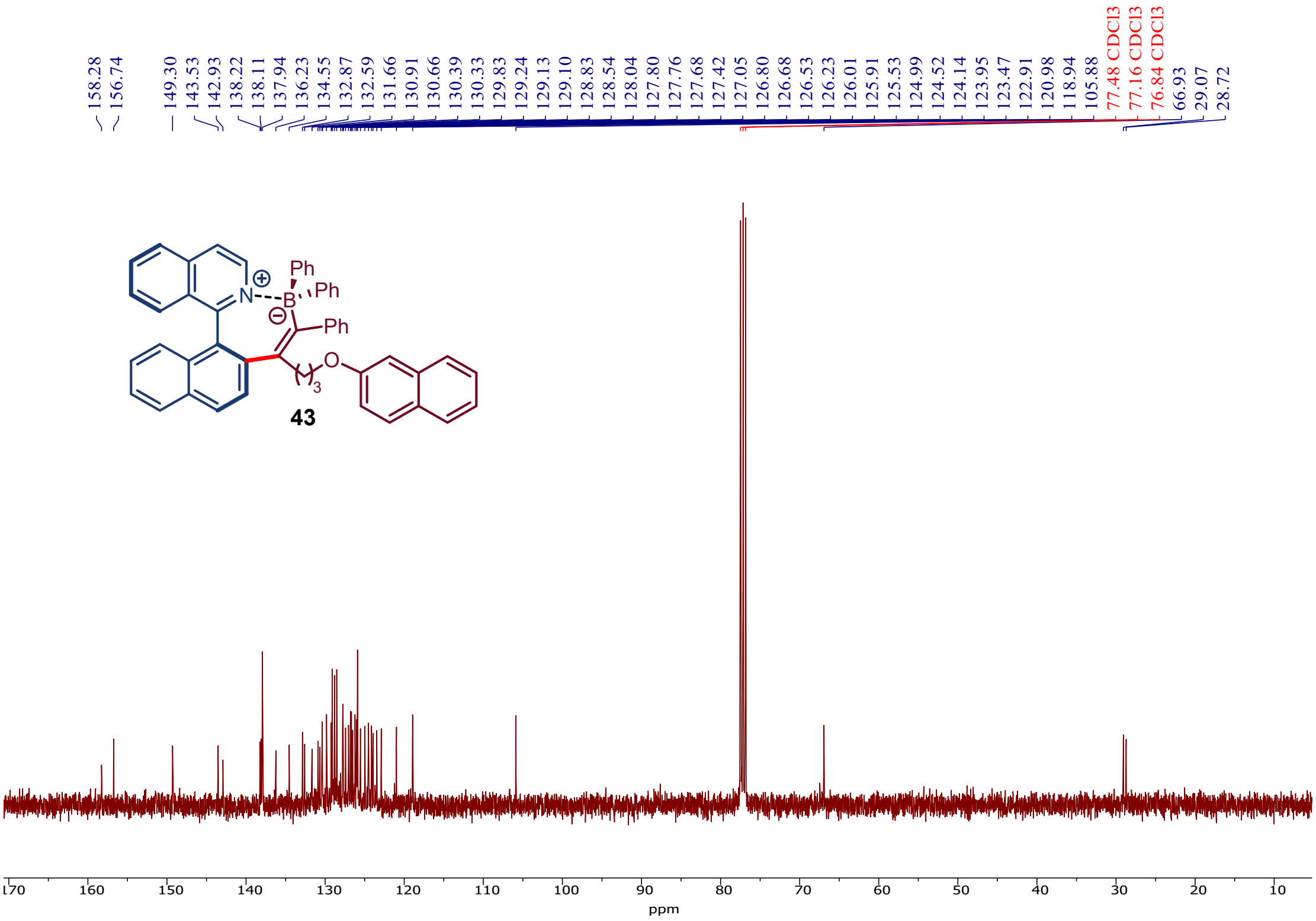

^{11}B NMR (128 MHz, $CDCl_3$) of **43**

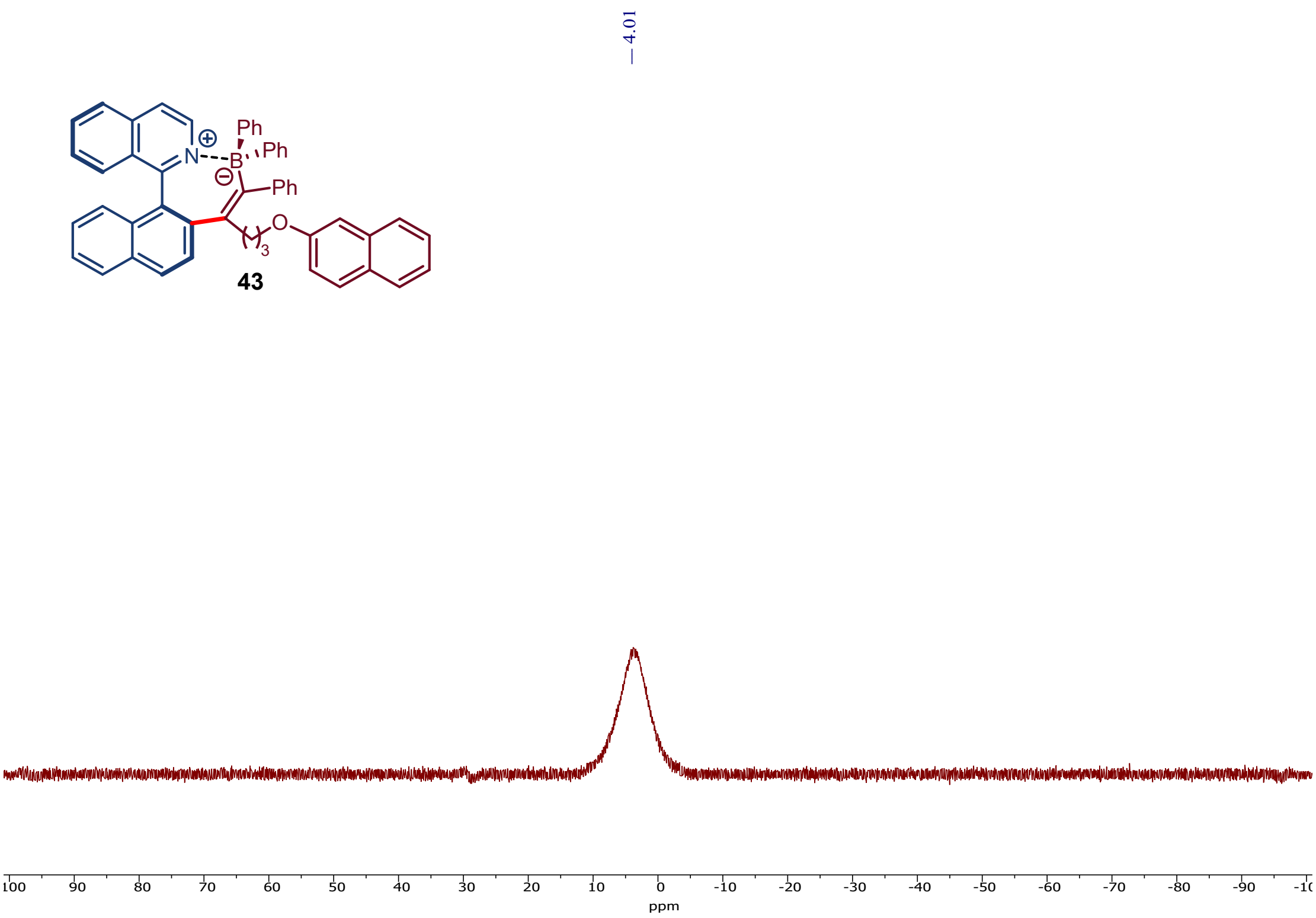

^{1}H NMR (400 MHz, $CDCl_3$) of **44** (*see procedure*)

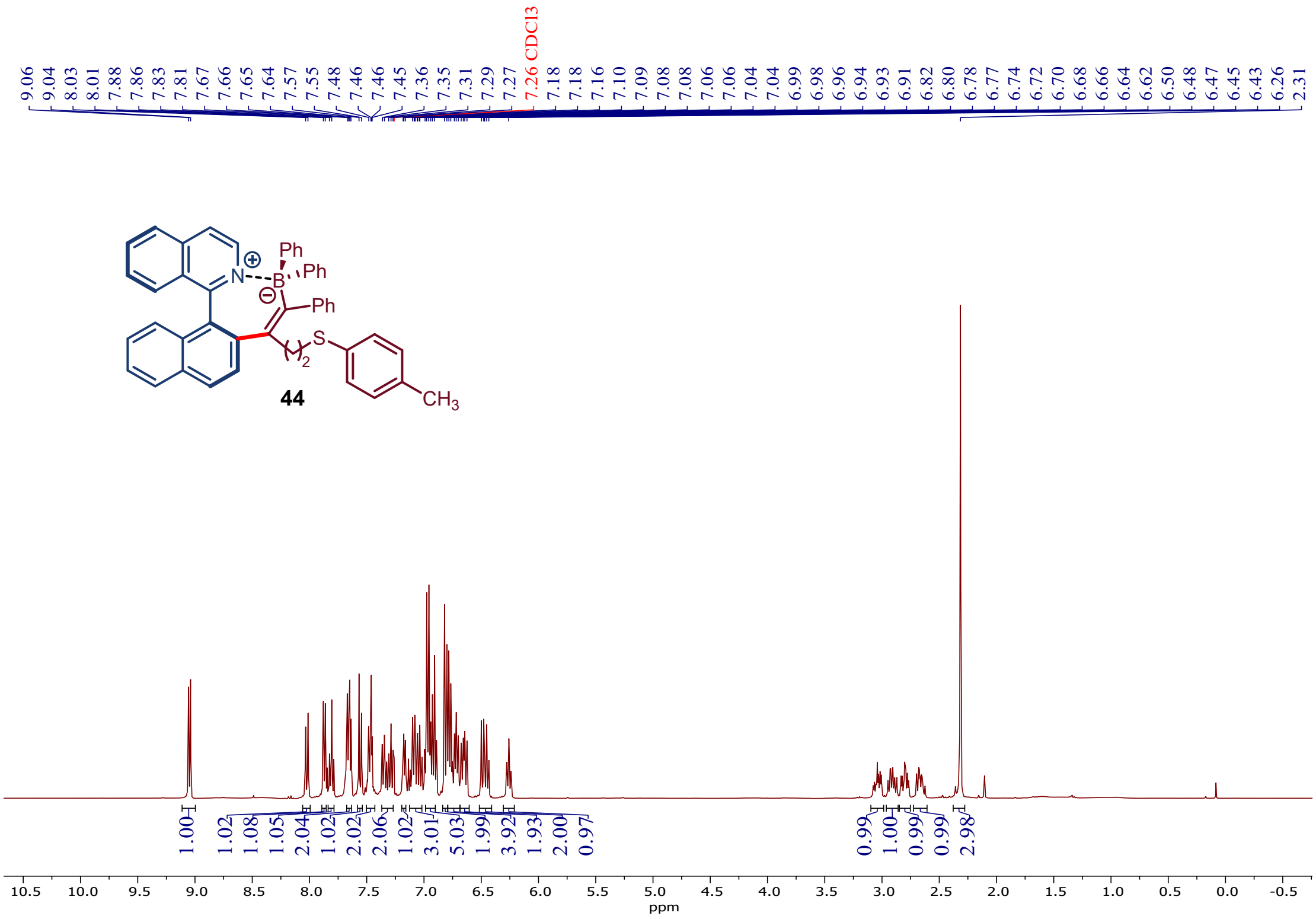

^{13}C NMR (100 MHz, $CDCl_3$) of **44**

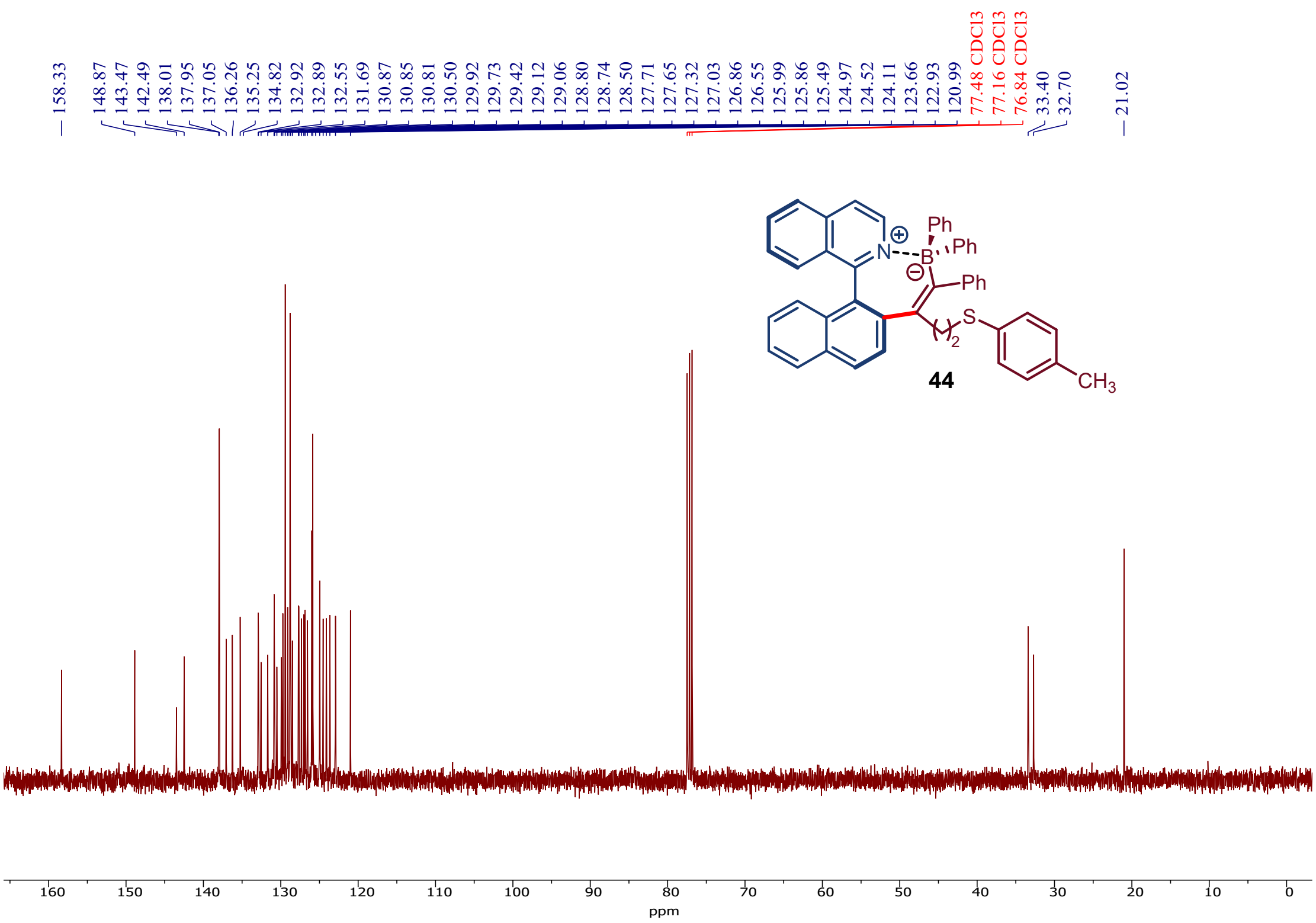

^{11}B NMR (128 MHz, $CDCl_3$) of **44**

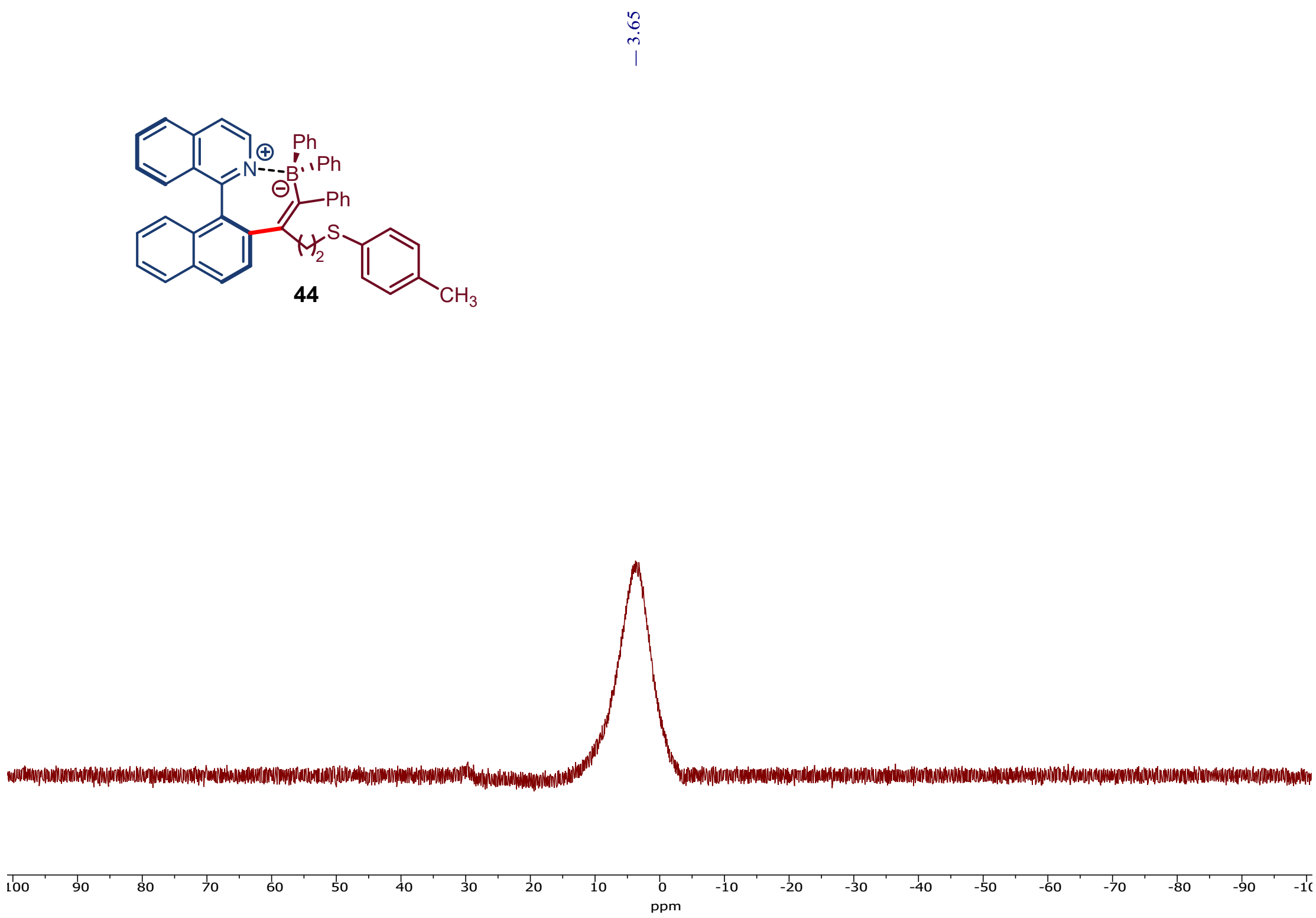

^{1}H NMR (400 MHz, $CDCl_3$) of **45** (*see procedure*)

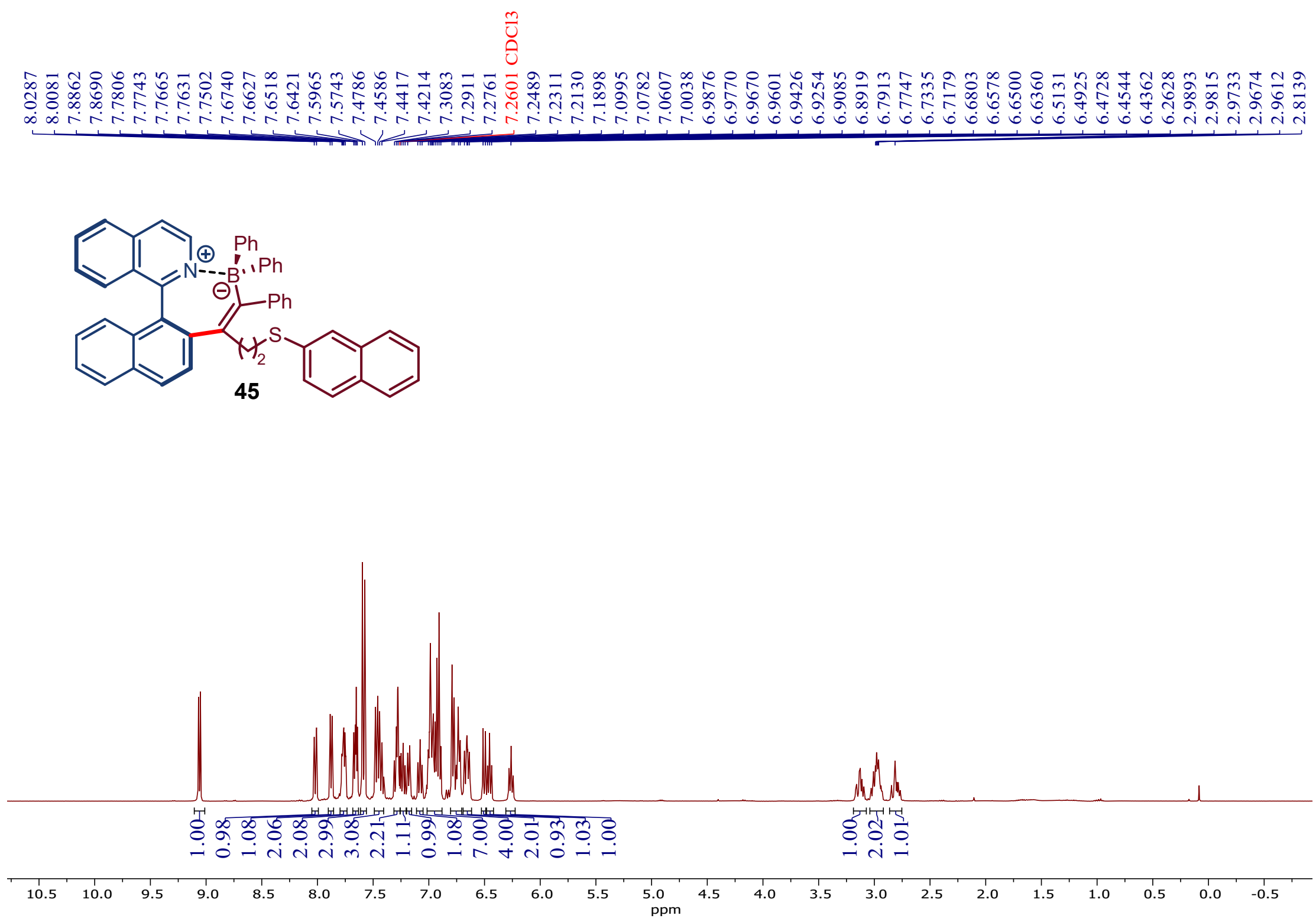

^{13}C NMR (100 MHz, $CDCl_3$) of **45**

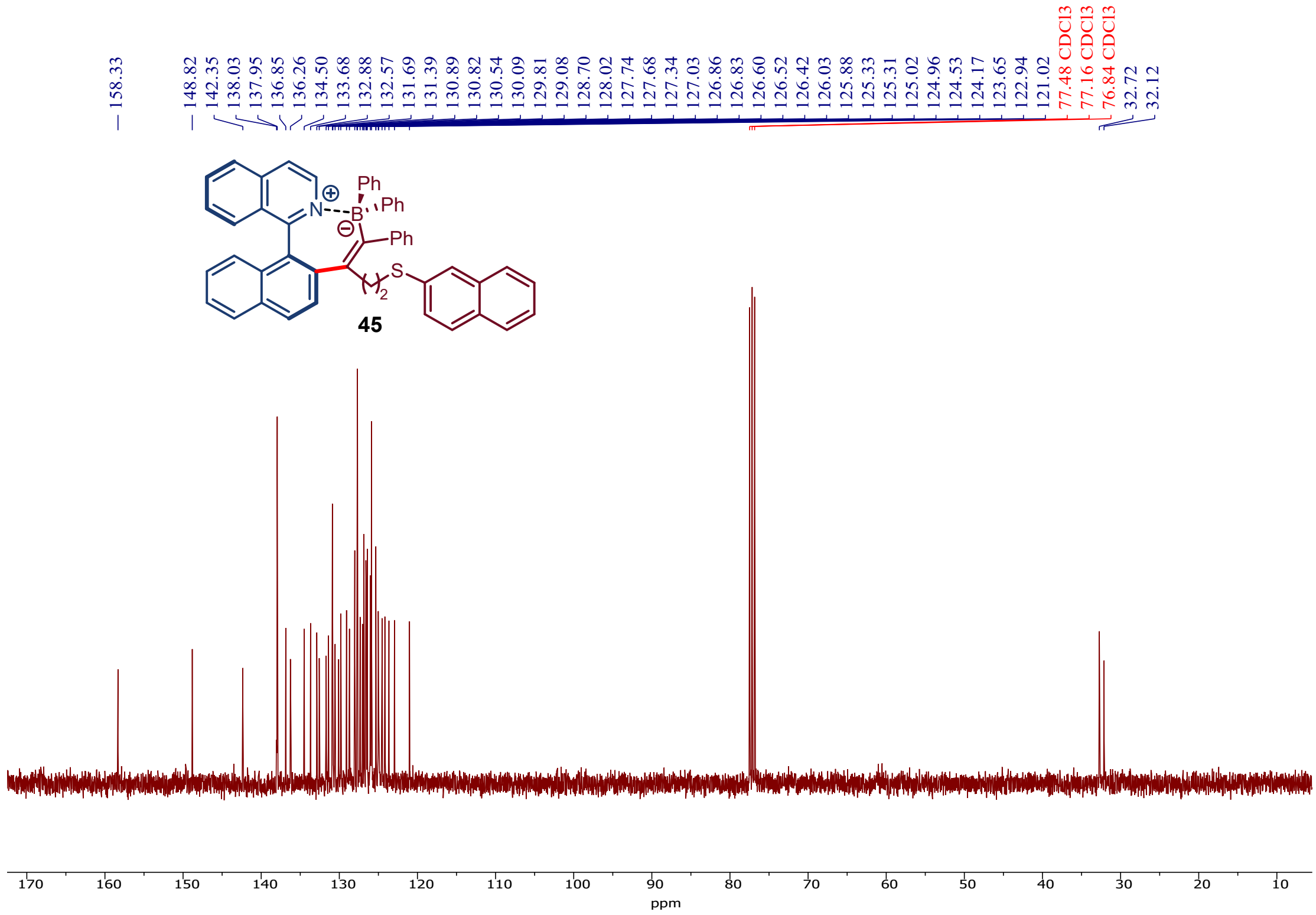

^{11}B NMR (128 MHz, $CDCl_3$) of **45**

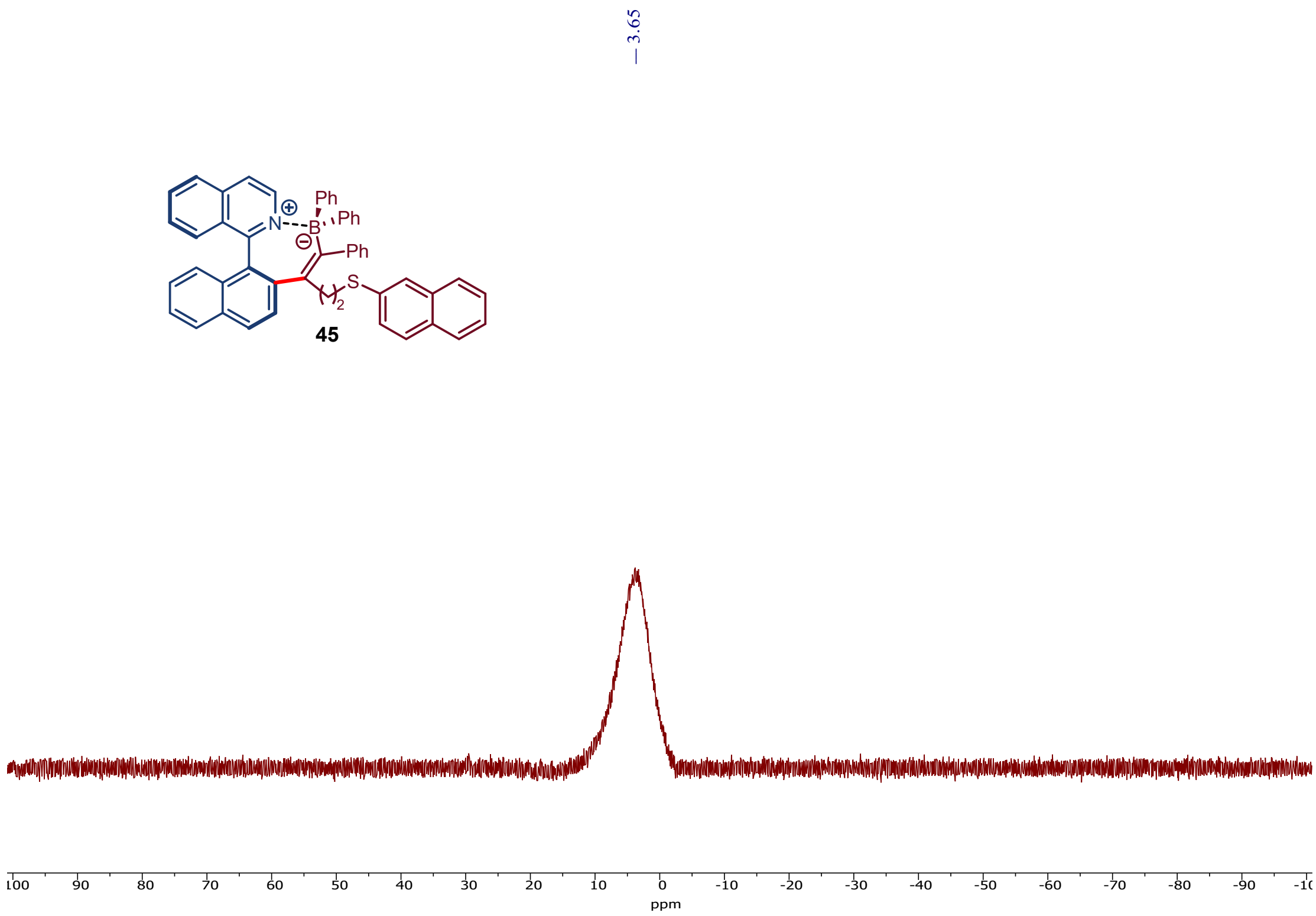

^{1}H NMR (400 MHz, $CDCl_3$) of **46** (*see procedure*)

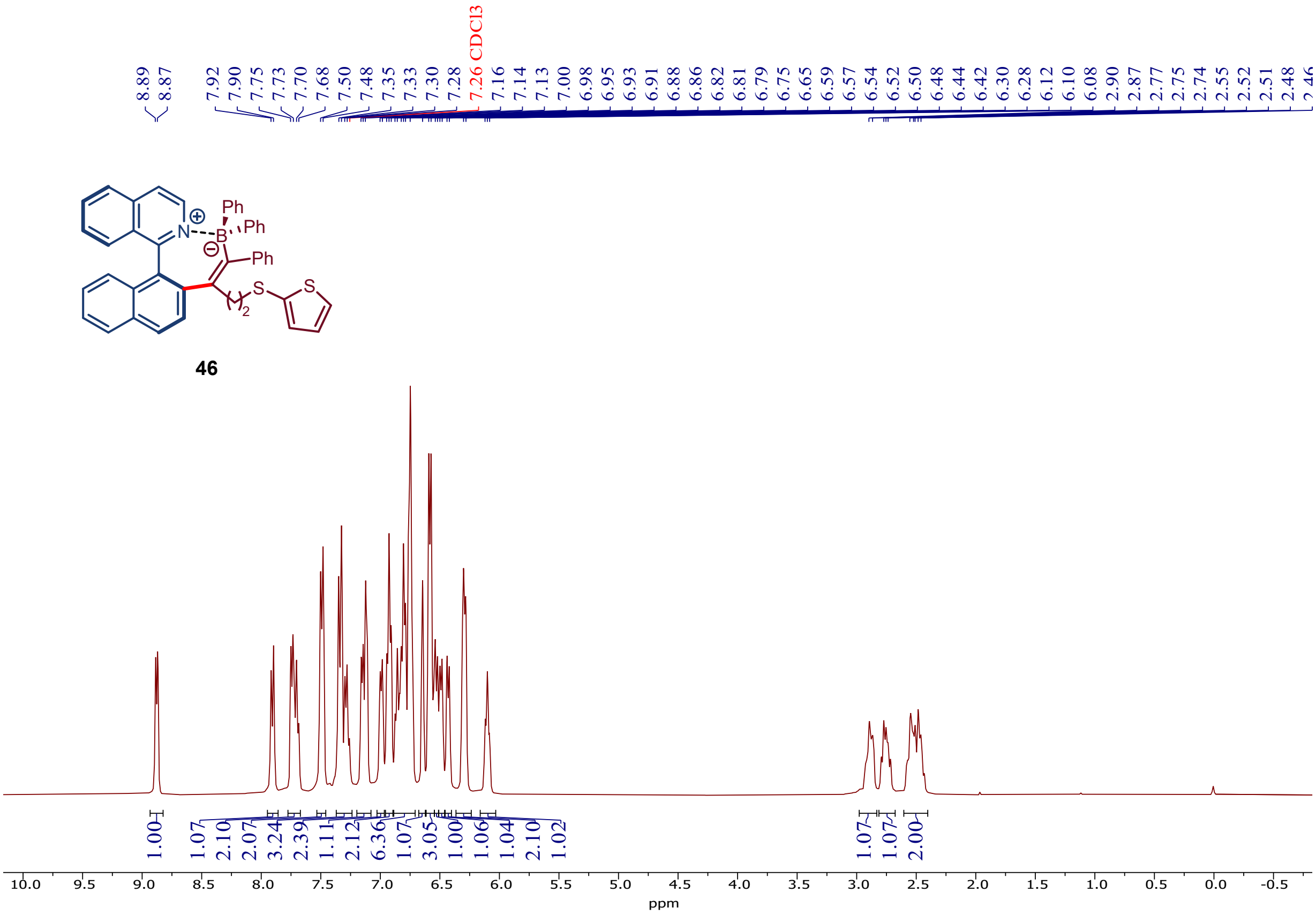

^{13}C NMR (100 MHz, $CDCl_3$) of **46**

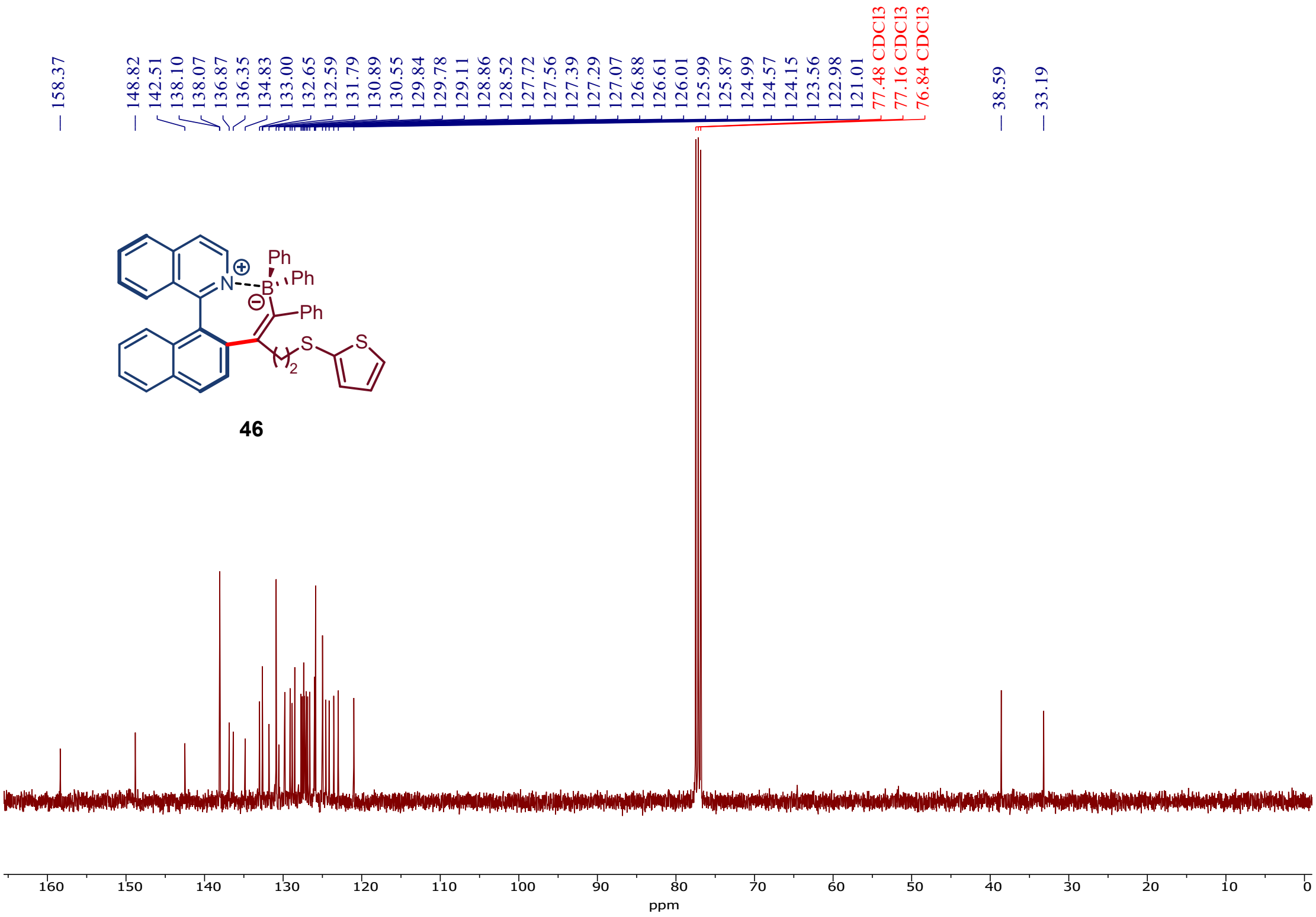

^{11}B NMR (128 MHz, $CDCl_3$) of **46**

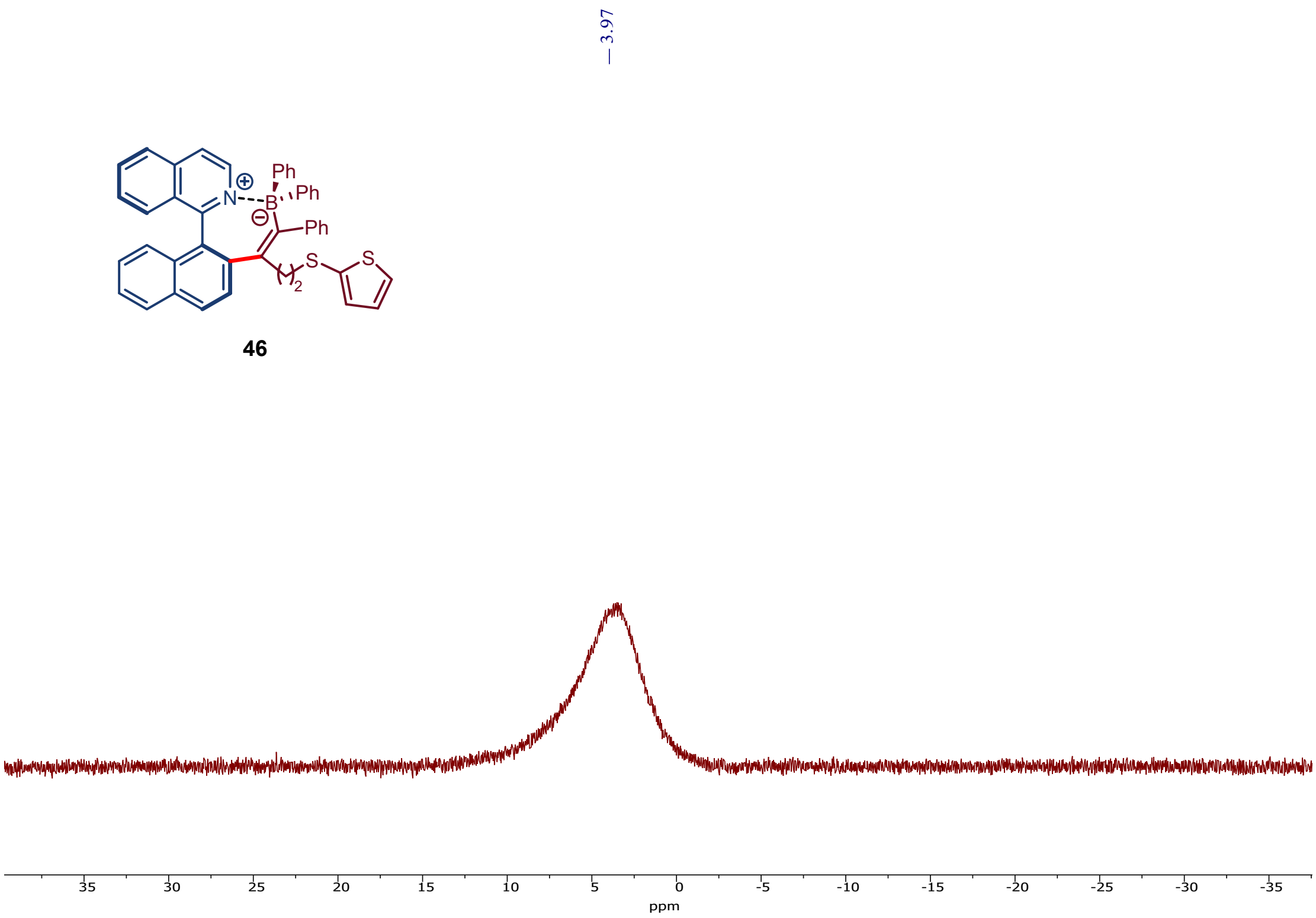

^{1}H NMR (400 MHz, $CDCl_3$) of **47** (*see procedure*)

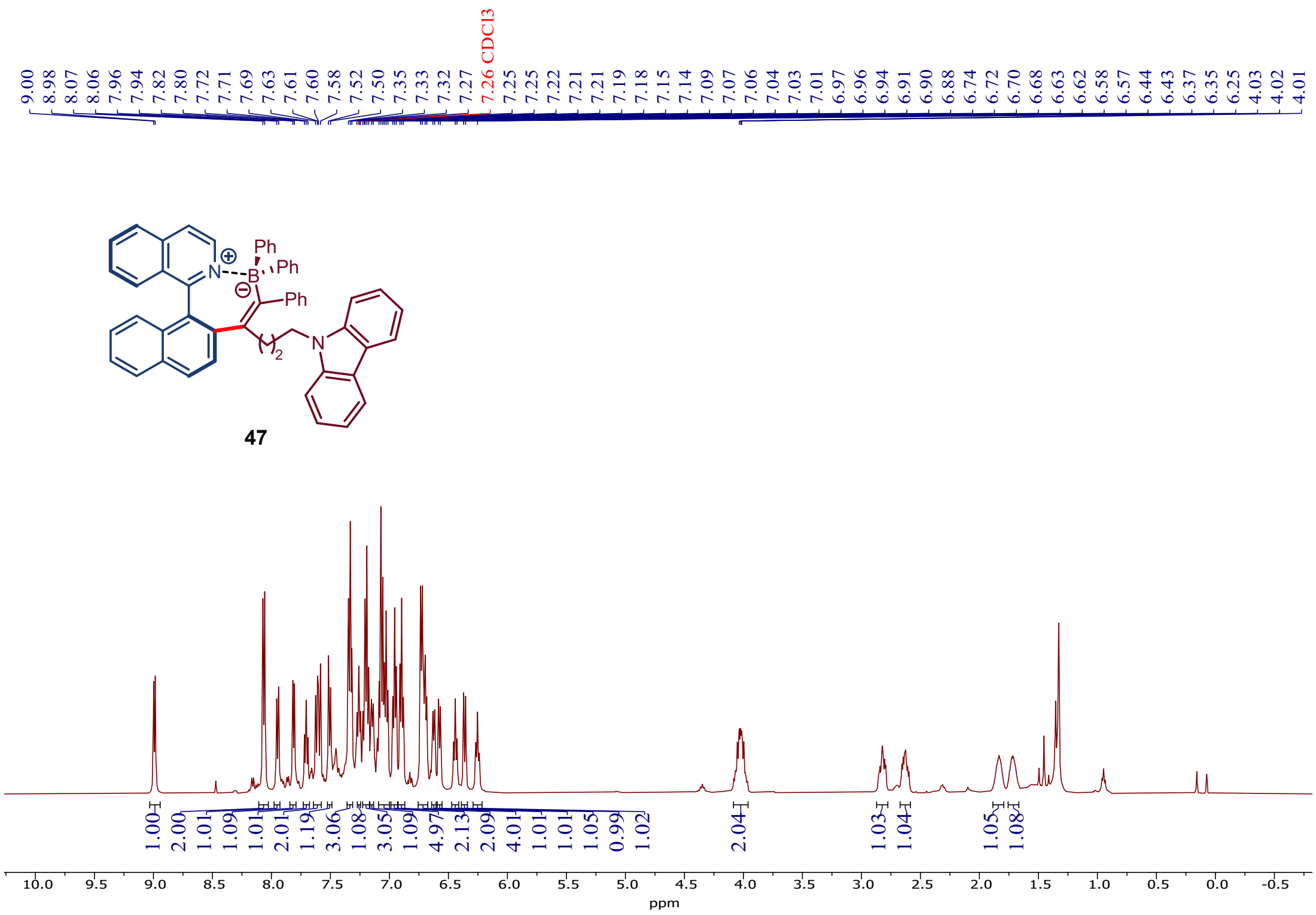

^{13}C NMR (100 MHz, $CDCl_3$) of **47**

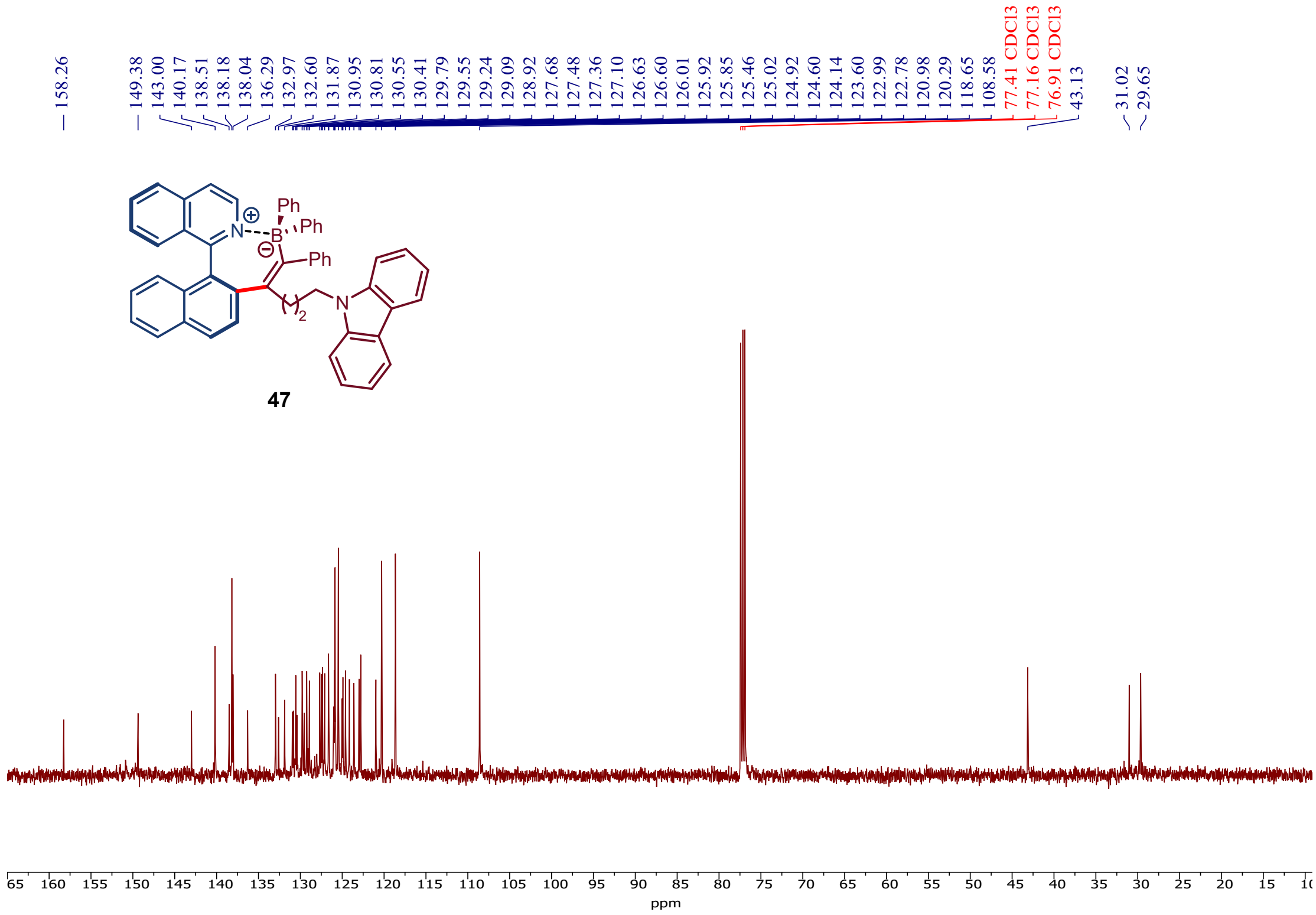

^{11}B NMR (128 MHz, $CDCl_3$) of **47**

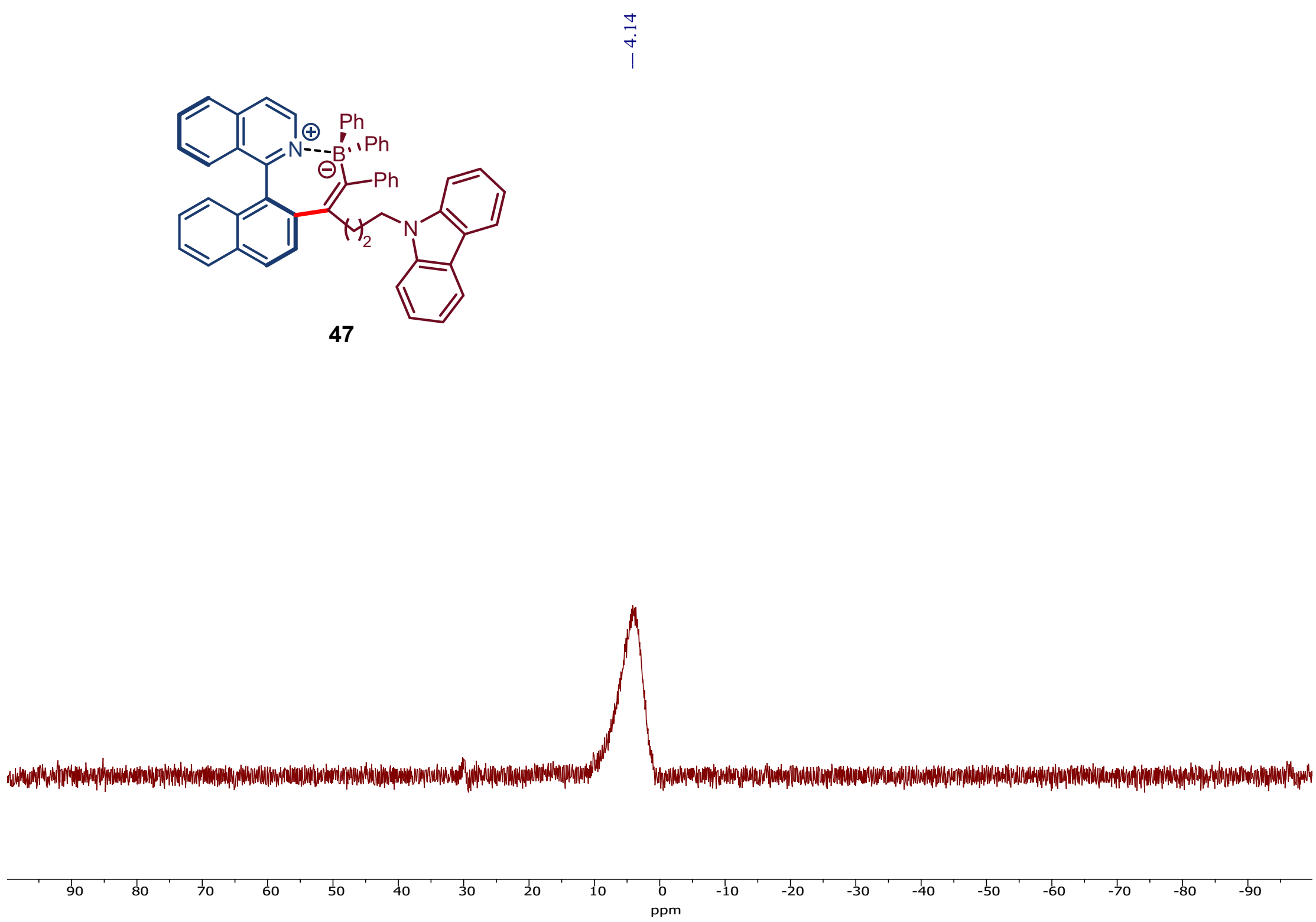

^{1}H NMR (400 MHz, $CDCl_3$) of **48** (*see procedure*)

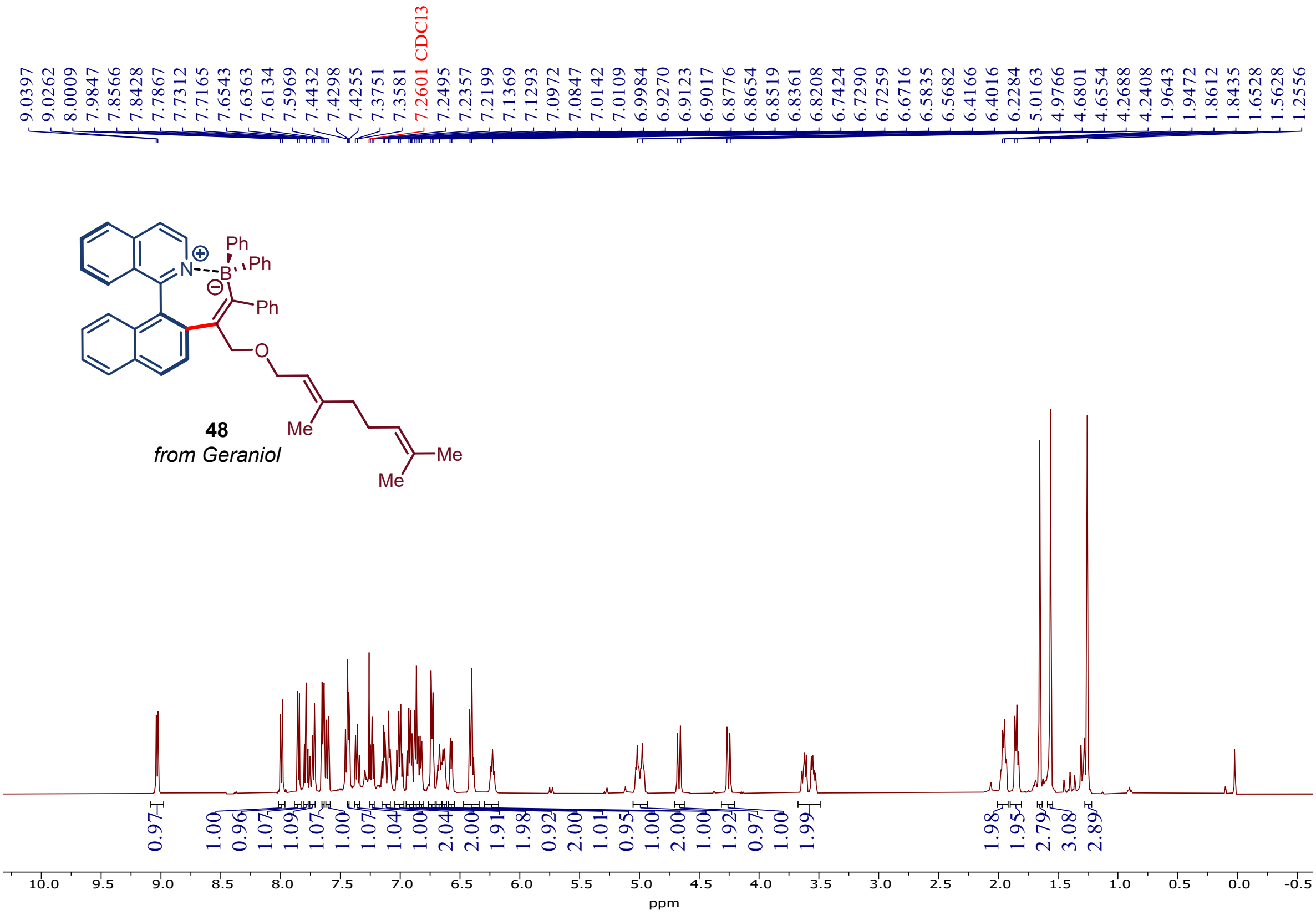

^{13}C NMR (100 MHz, $CDCl_3$) of **48**

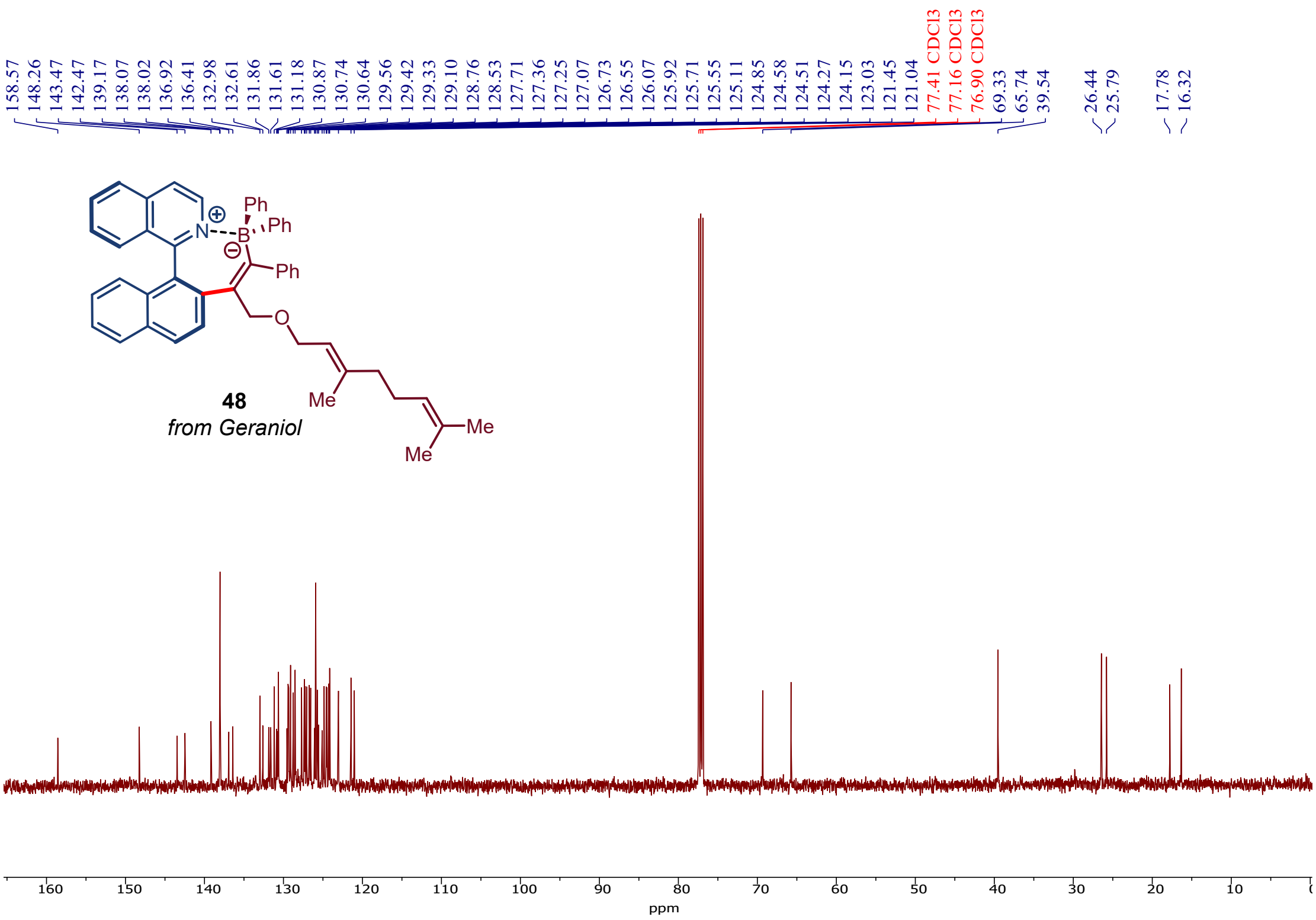

^{11}B NMR (128 MHz, $CDCl_3$) of **48**

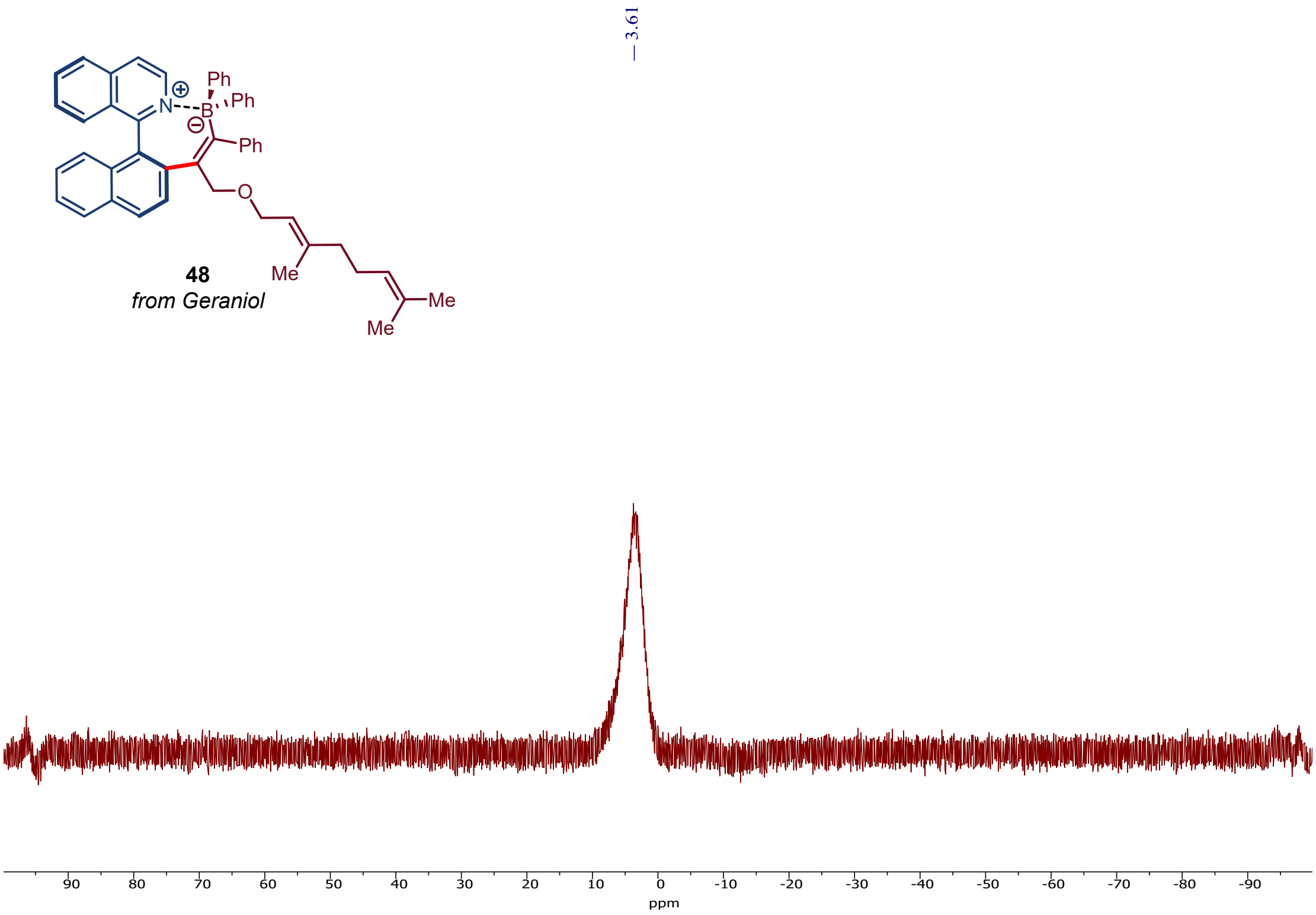

^{1}H NMR (500 MHz, $CDCl_3$) of **49** (*see procedure*)

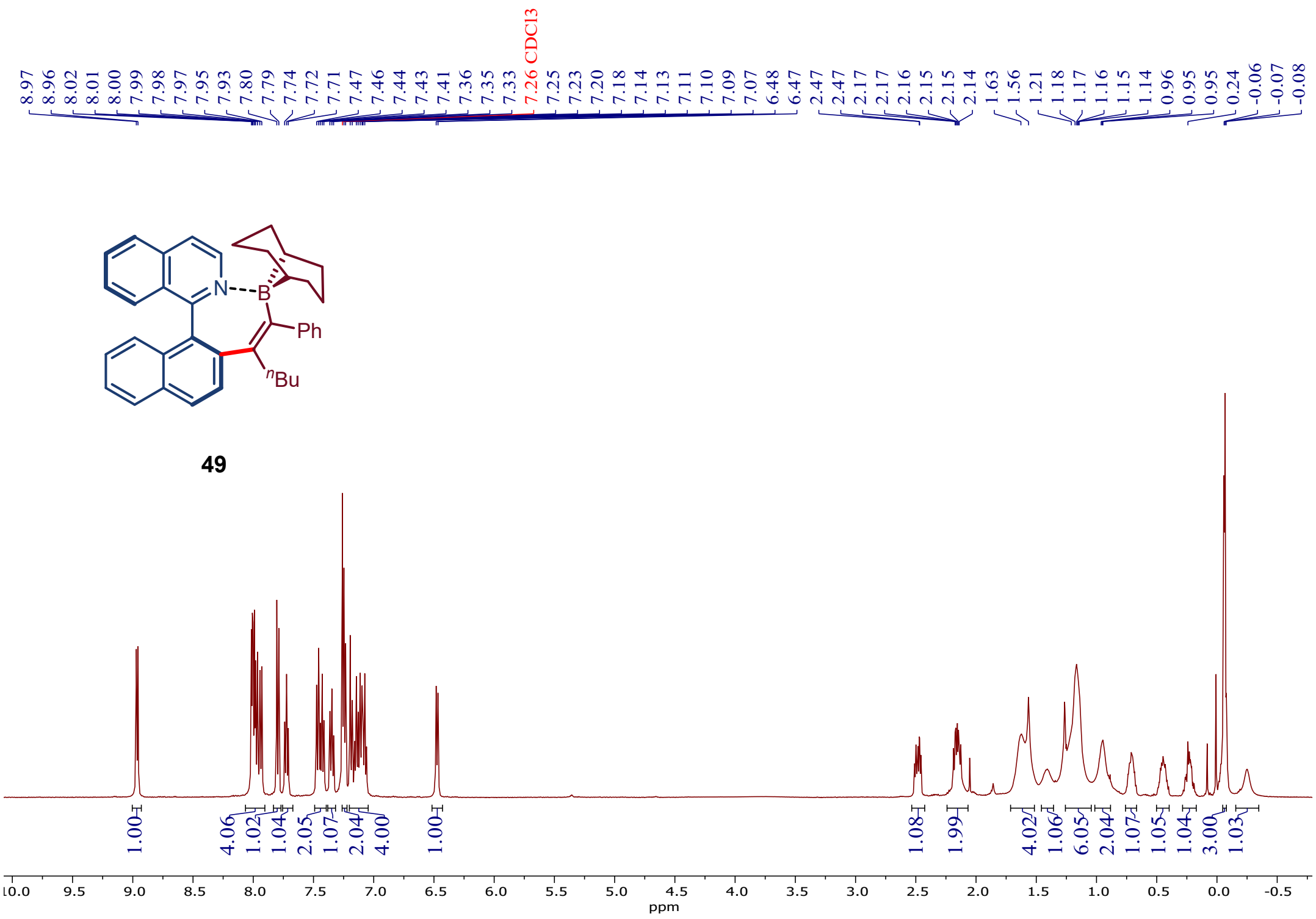

^{13}C NMR (126 MHz, $CDCl_3$) of **49**

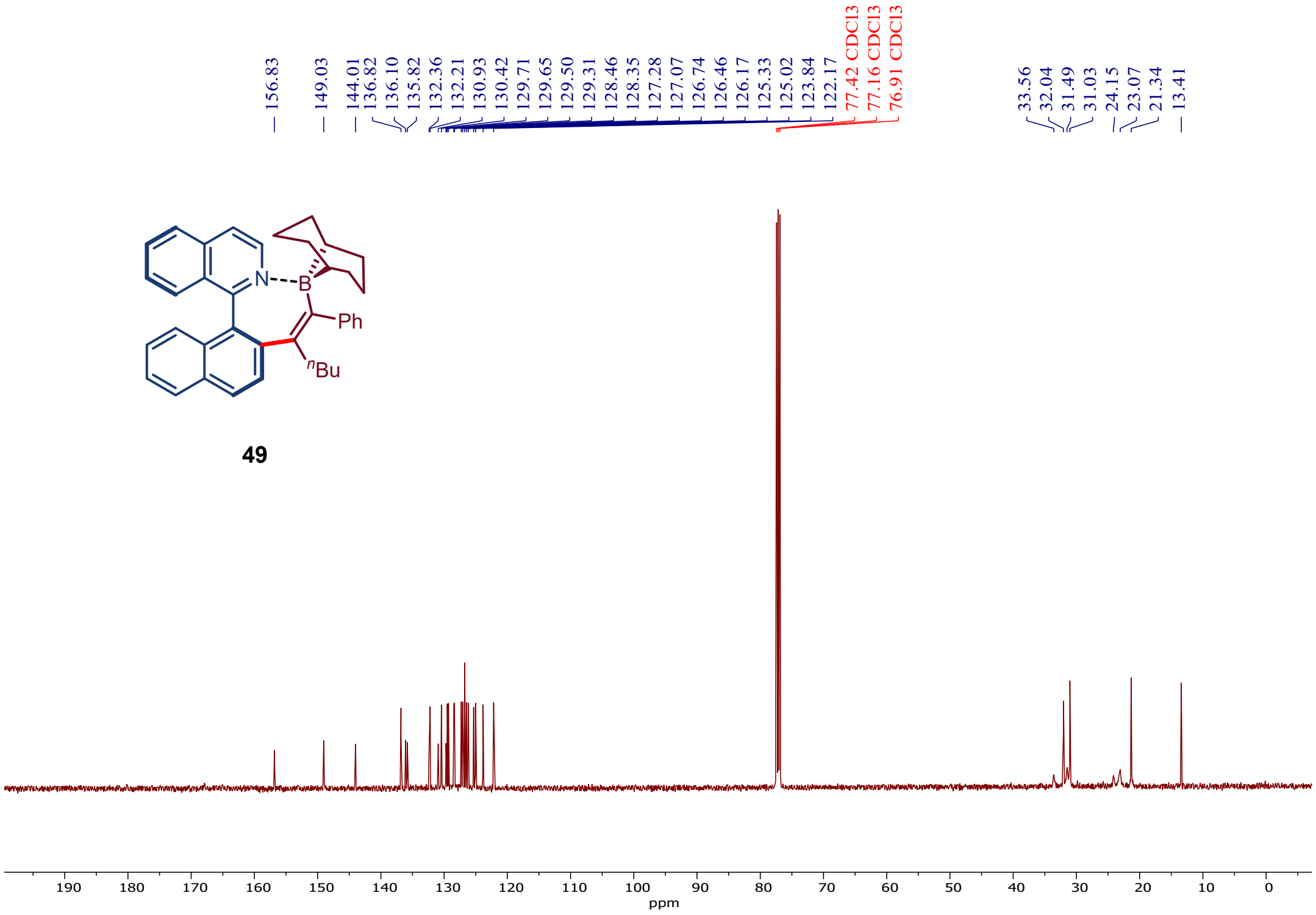

^{11}B NMR (128 MHz, $CDCl_3$) of **49**

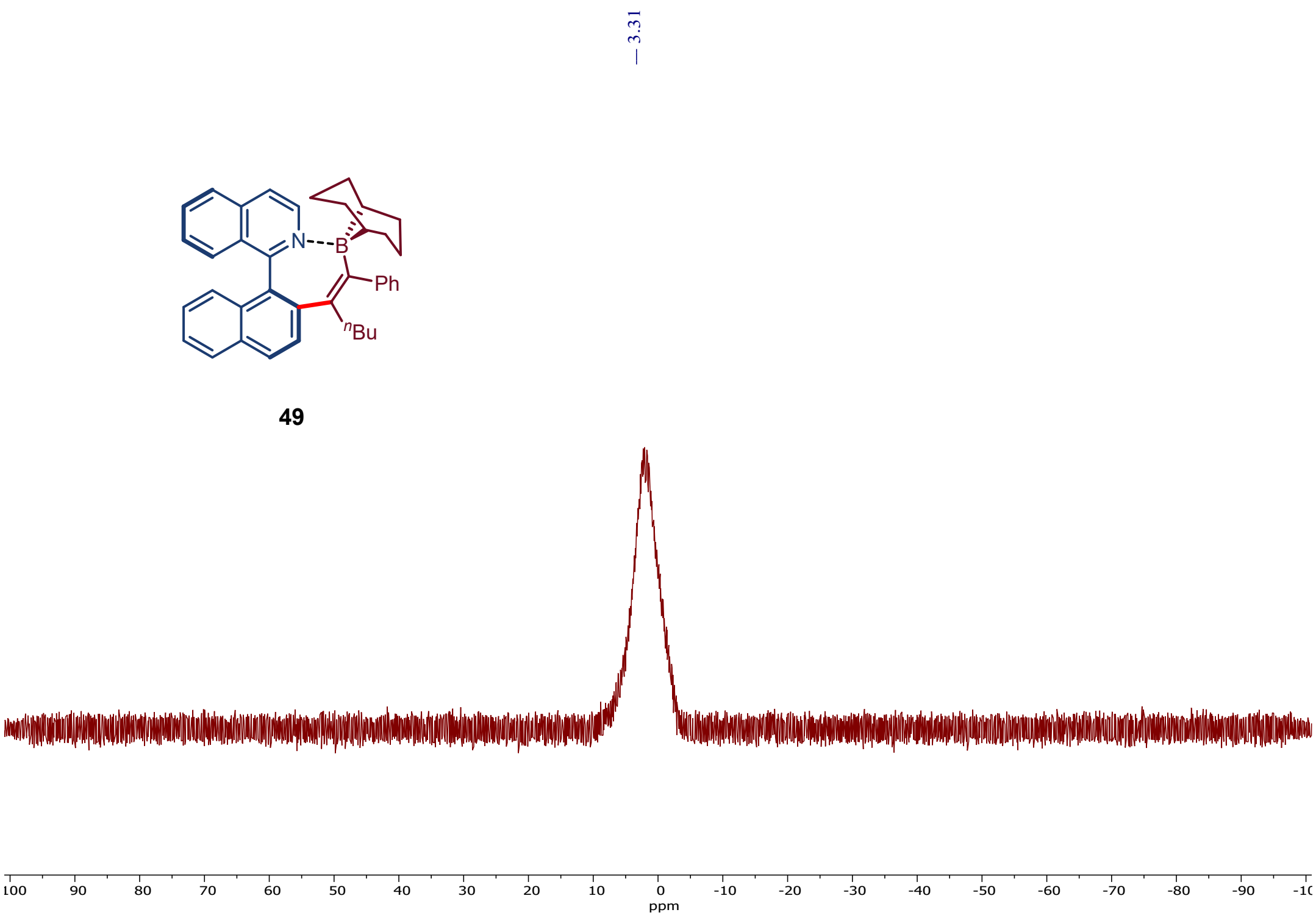

^{1}H NMR (400 MHz, $CDCl_3$) of **52** (*see procedure*)

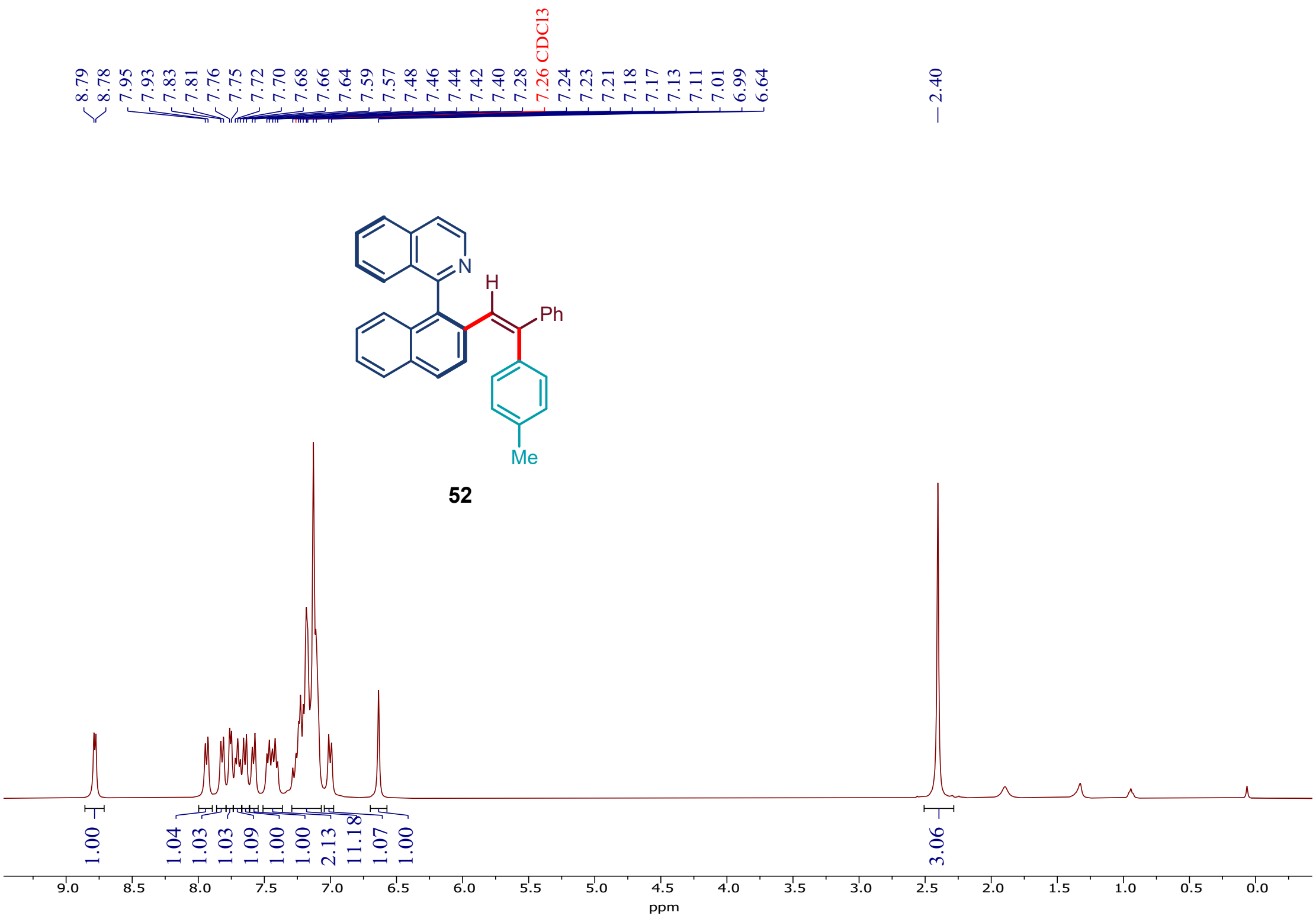

^{13}C NMR (100 MHz, $CDCl_3$) of **52**

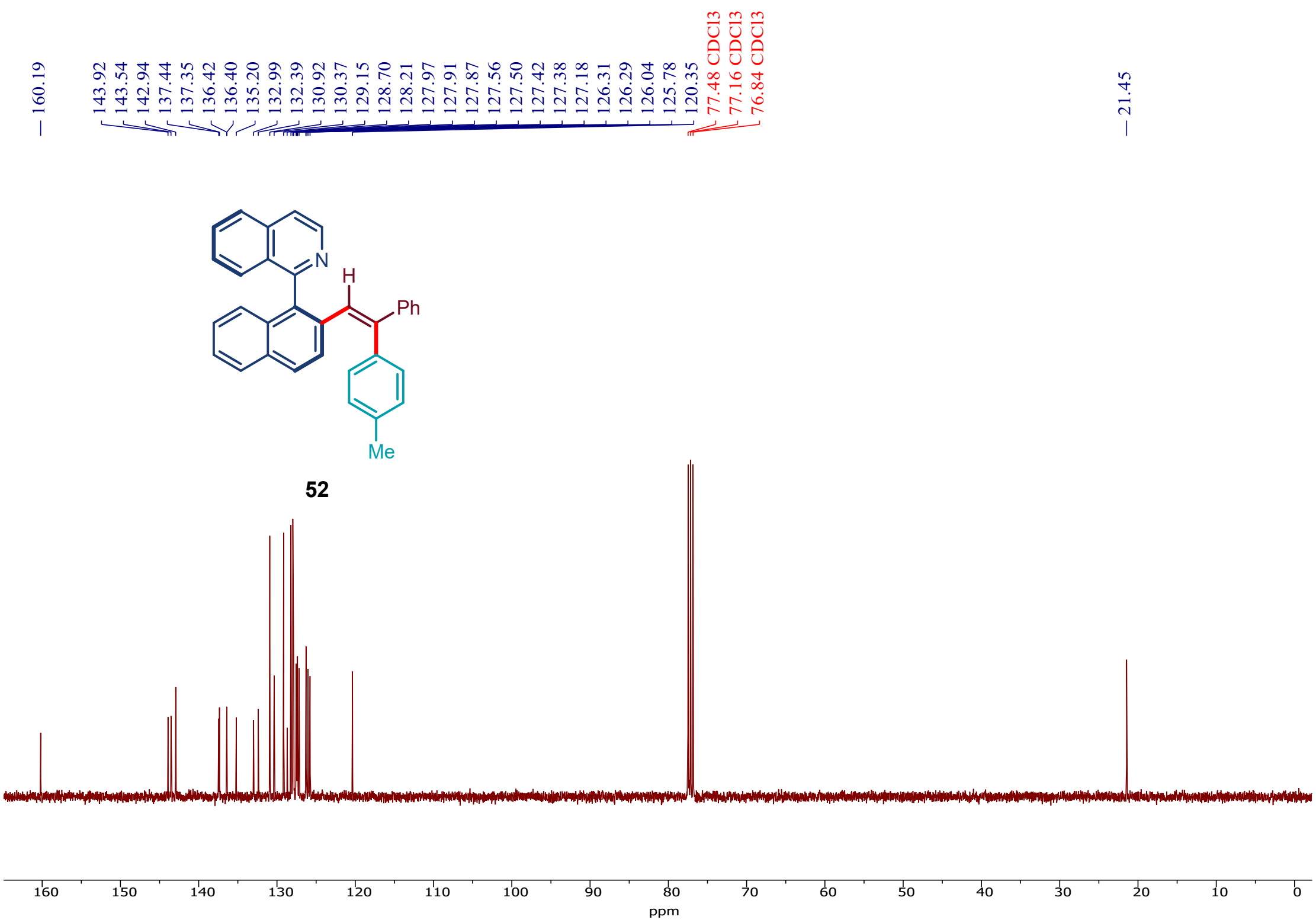

^{1}H NMR (400 MHz, $CDCl_3$) of **53** (*see procedure*)

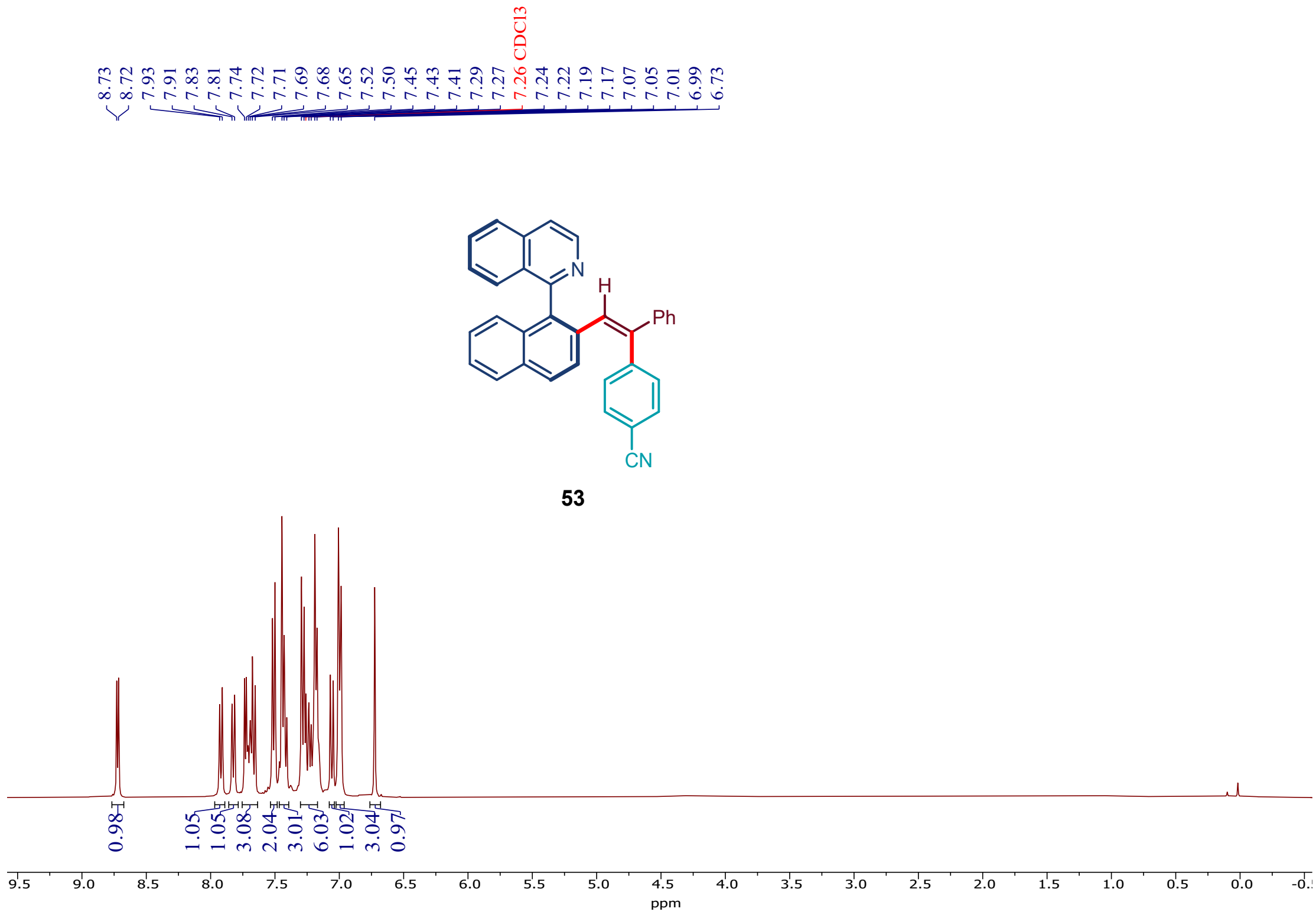

^{13}C NMR (100 MHz, $CDCl_3$) of **53**

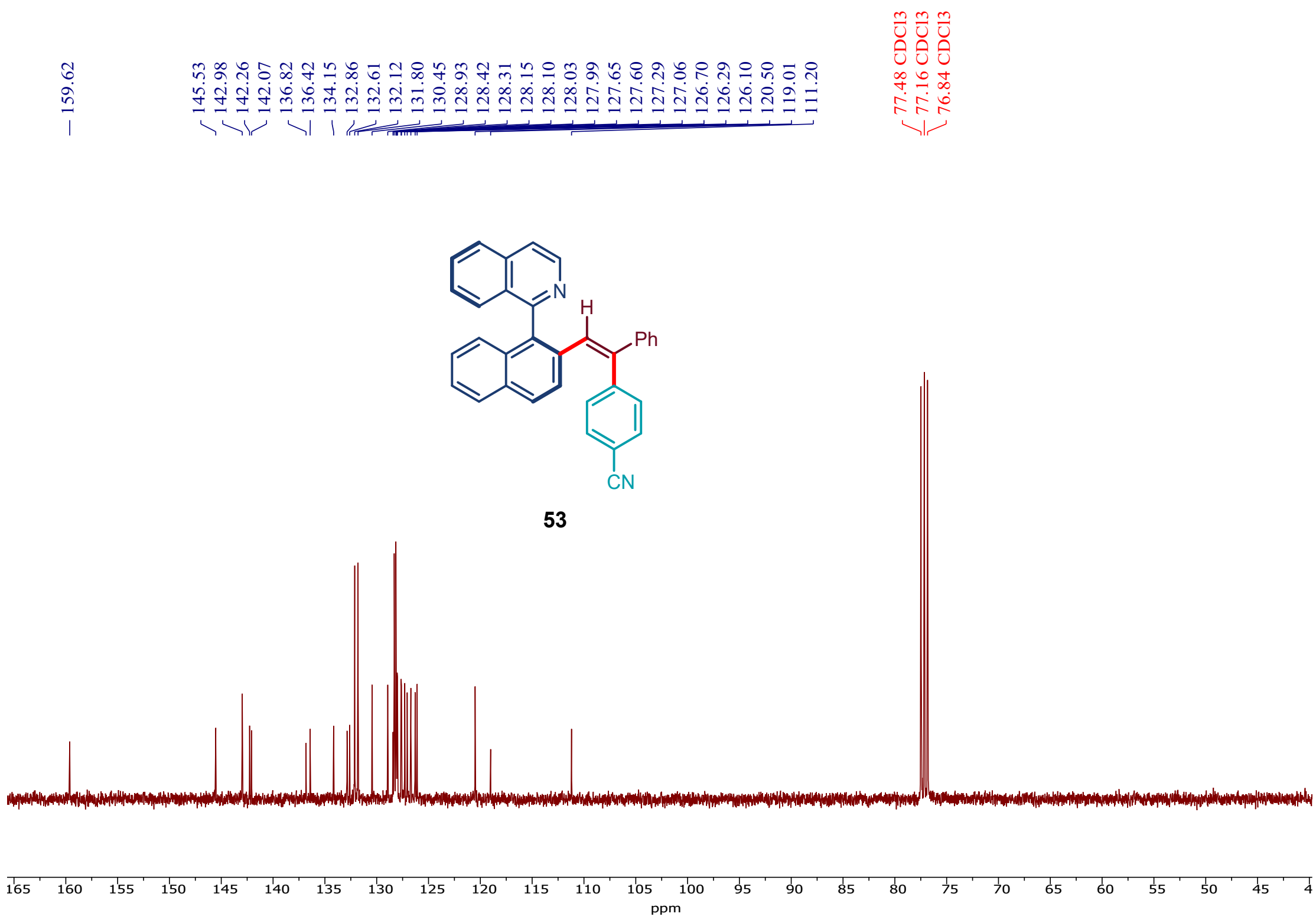

^{1}H NMR (400 MHz, $CDCl_3$) of **54** (see procedure)

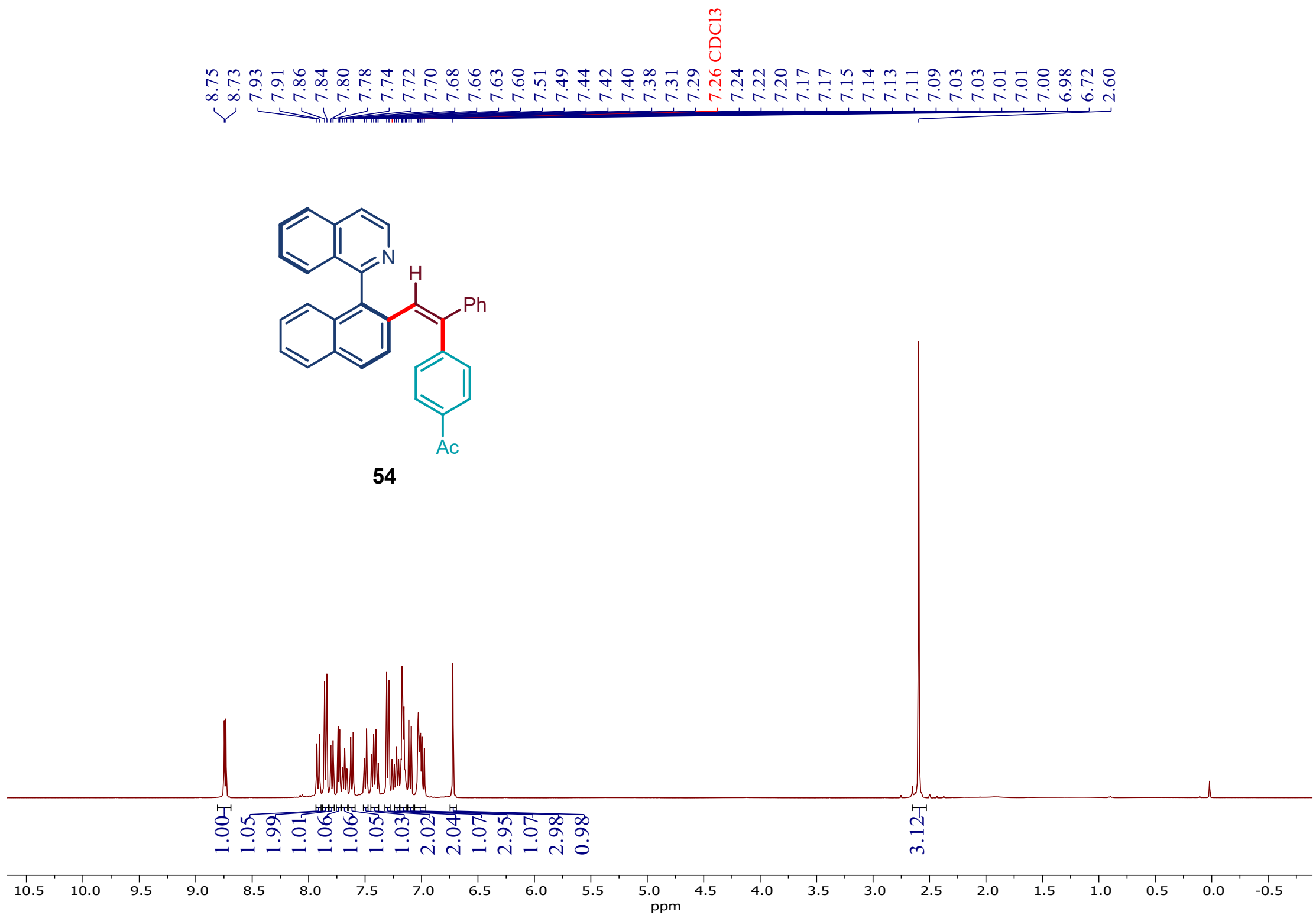

^{13}C NMR (100 MHz, $CDCl_3$) of **54**

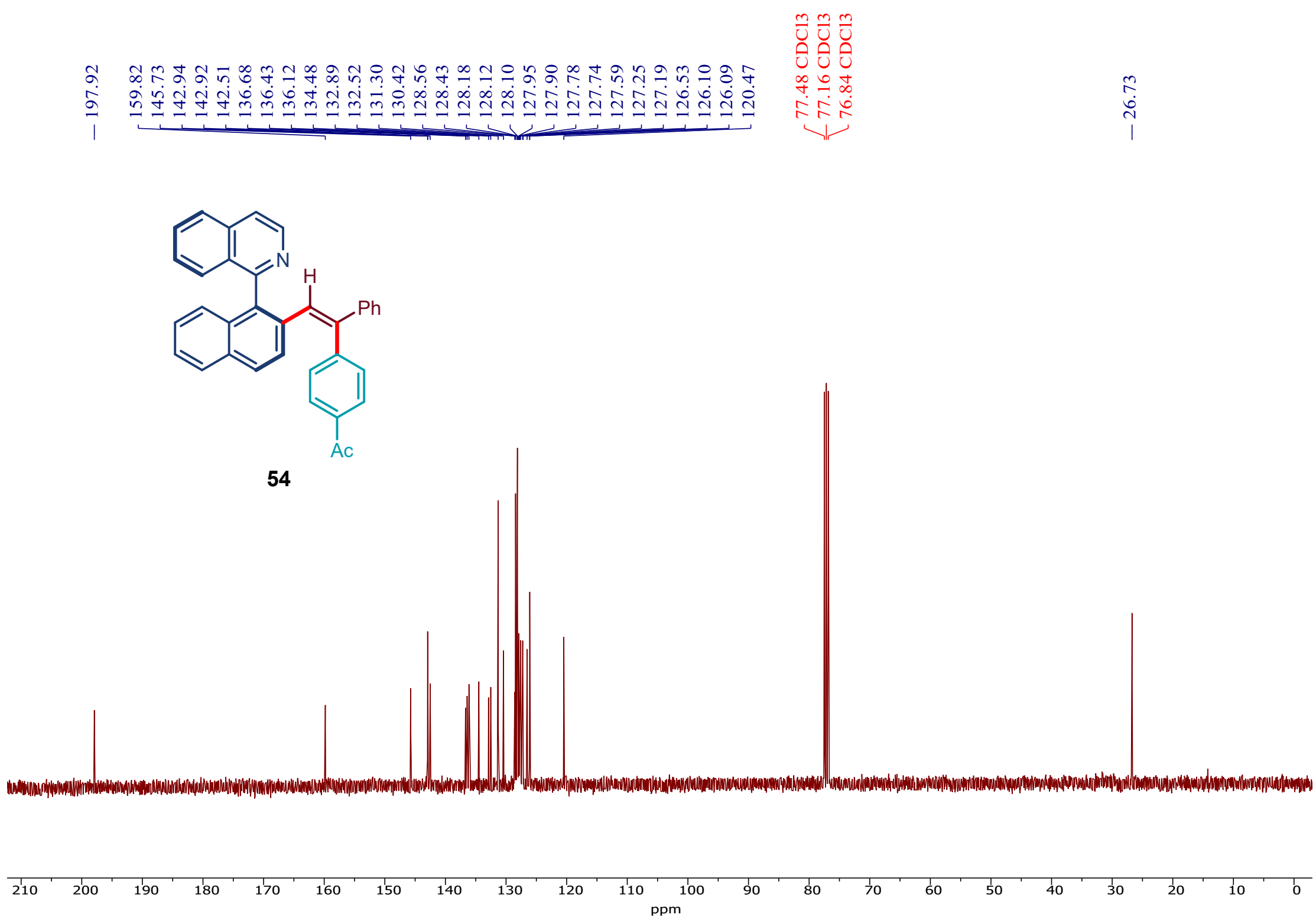

^{1}H NMR (400 MHz, $CDCl_3$) of **55** (*see procedure*)

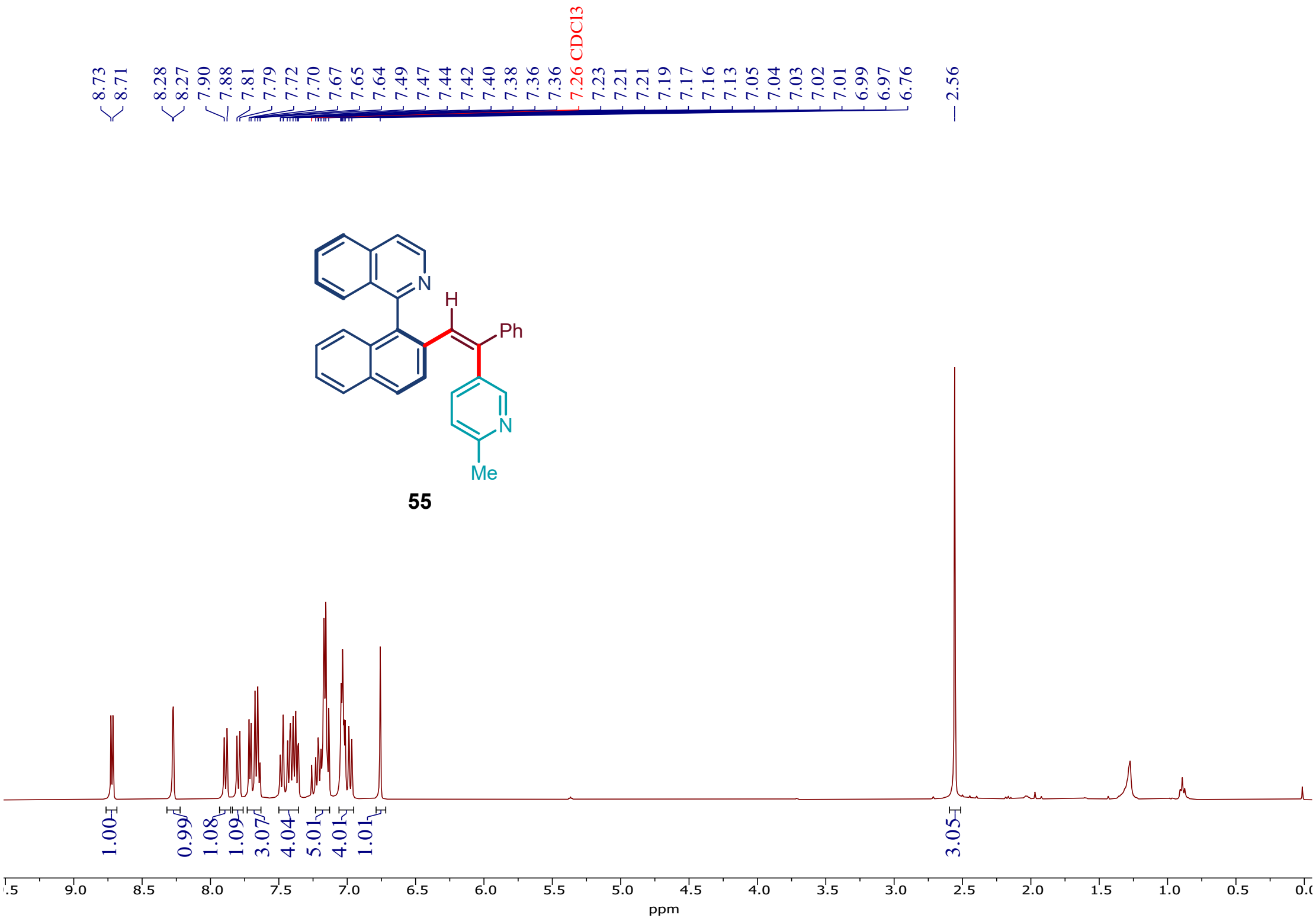

^{13}C NMR (100 MHz, $CDCl_3$) of **55**

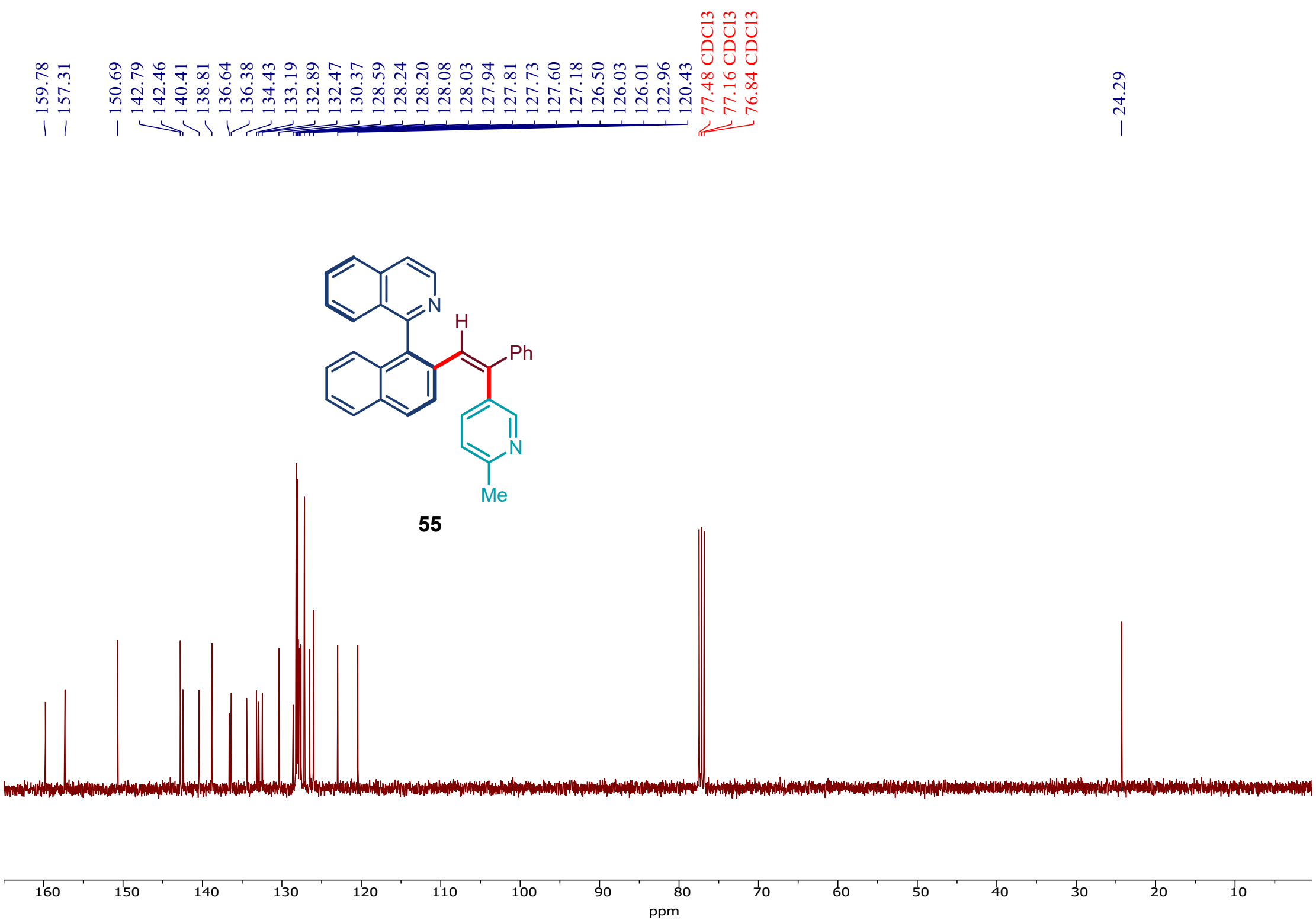

NOESY Analysis of **55**

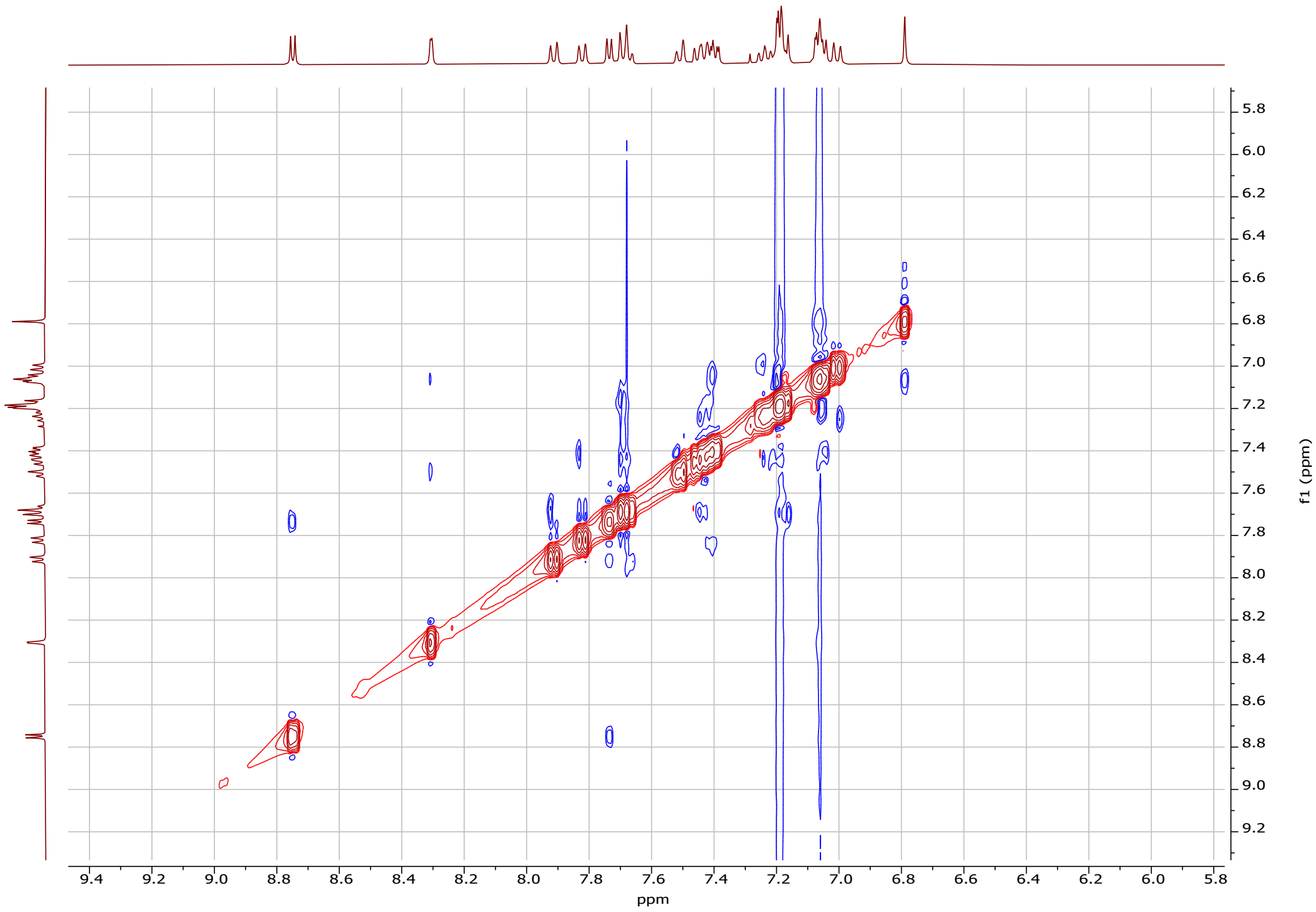

^{1}H NMR (400 MHz, $CDCl_3$) of **56** (*see procedure*)

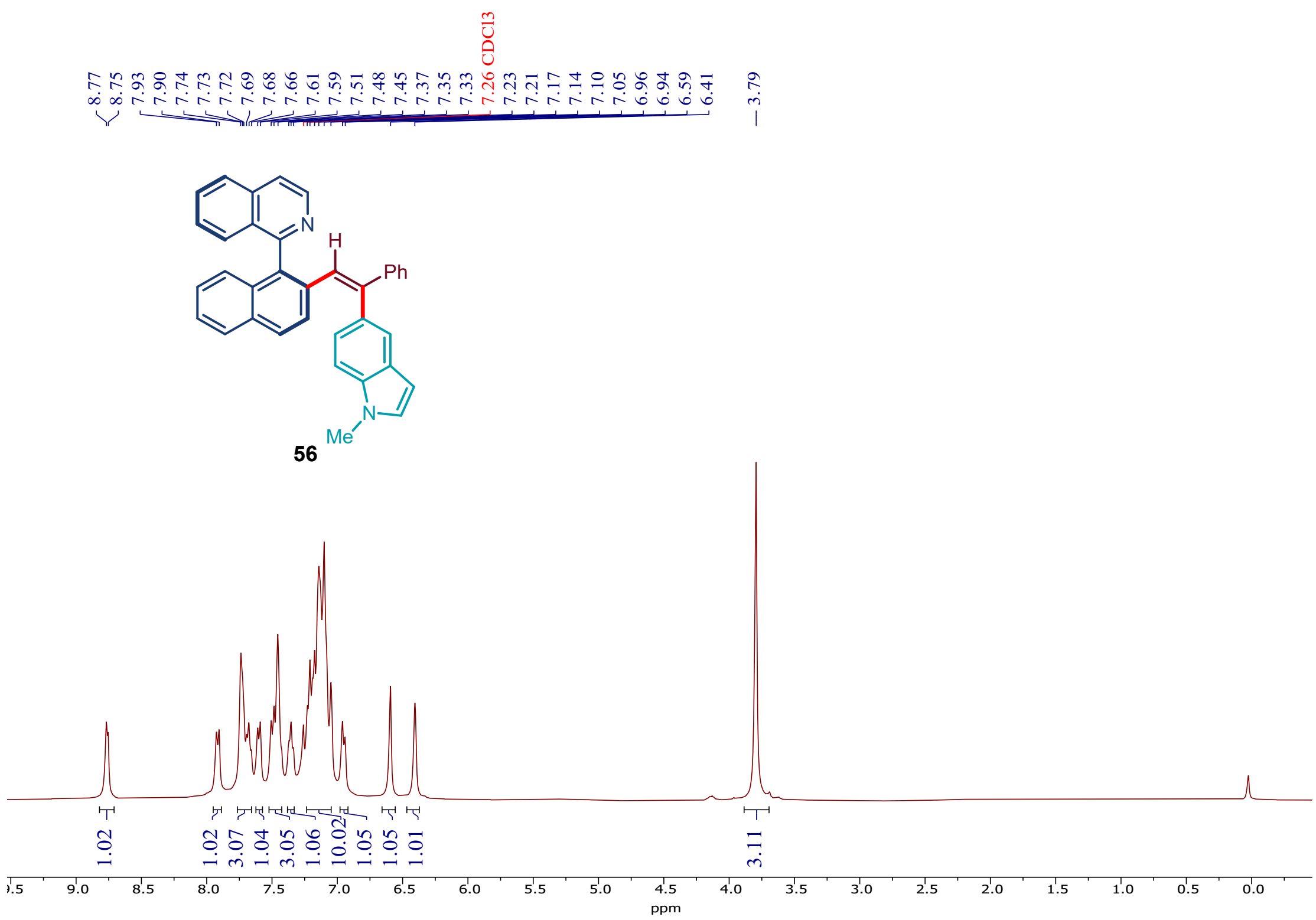

^{13}C NMR (100 MHz, $CDCl_3$) of **56**

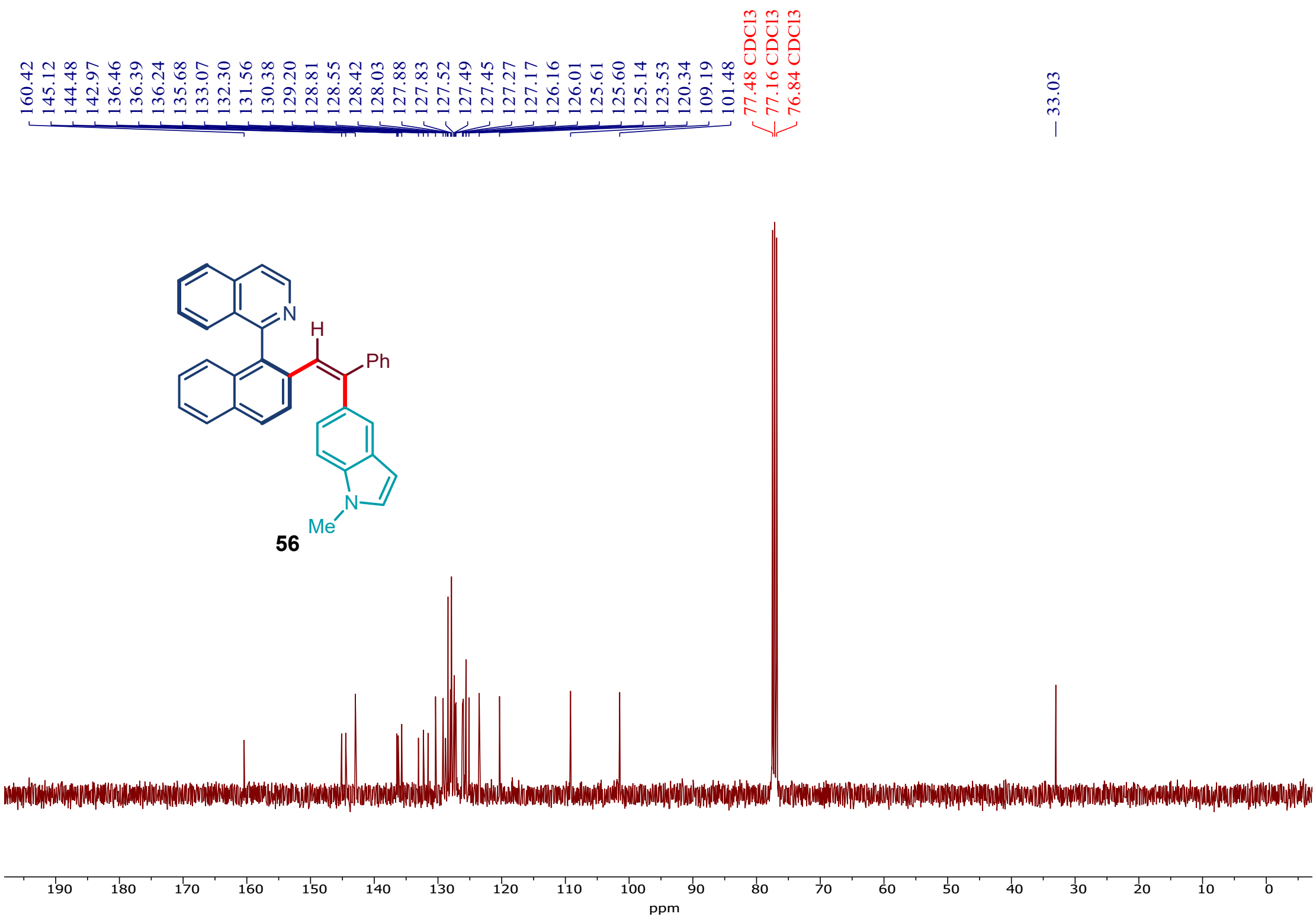

^{1}H NMR (400 MHz, $CDCl_3$) of **57** (*see procedure*)

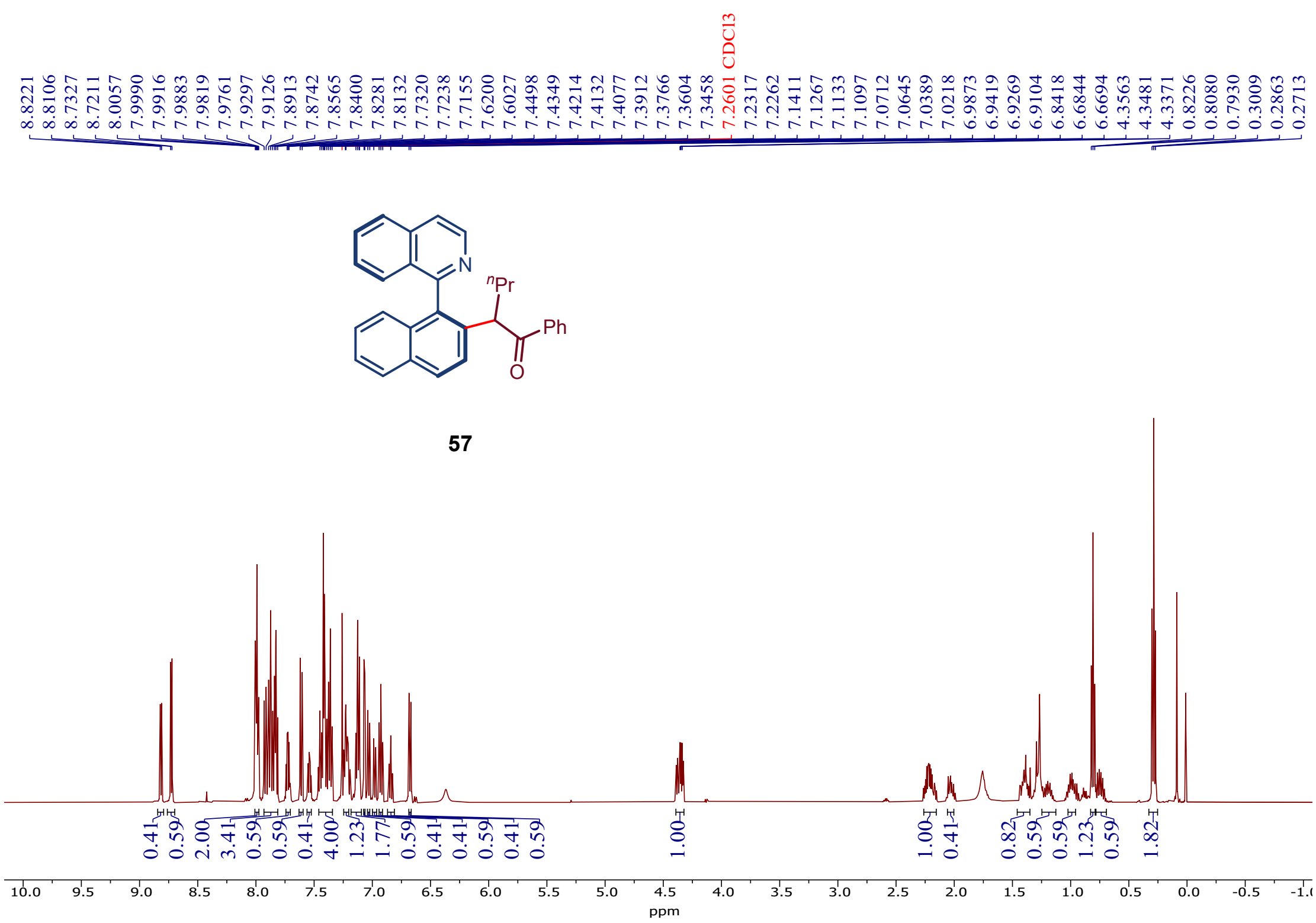

^{13}C NMR (100 MHz, $CDCl_3$) of **57**

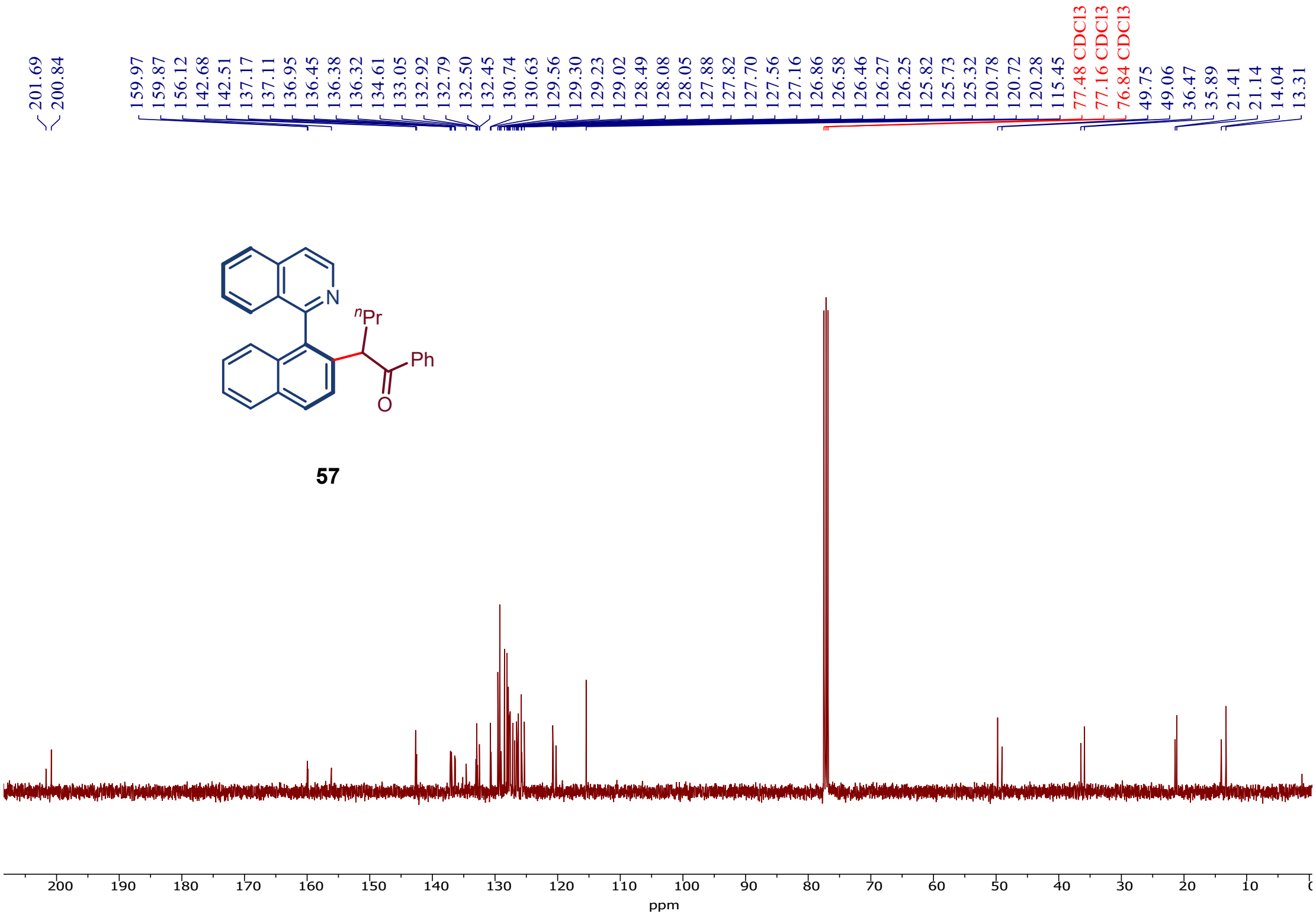

9. SUPPLEMENTARY REFERENCES

[1] P.-Y. Jiang, K.-F. Fan, S. Li, S.-H. Xiang, B. Tan, "Metal-free oxidative cross-coupling enabled practical synthesis of atropisomeric QUINOL and its derivatives" *Nat. Commun.* ***12***, (2021): 2384, https://doi.org/10.1038/s41467-021-22621-2.

[2] L. Su, S. Gao, J. Liu, "Enantioconvergent synthesis of axially chiral amides enabled by Pd-catalyzed dynamic kinetic asymmetric aminocarbonylation" *Nat. Commun.* ***15***, (2024): 7248, https://doi.org/10.1038/s41467-024-51717-8.

[3] Y.-P. Shao, Y.-M. Liang, "Dynamic Kinetic Reductive Grignard-Type Addition for the Construction of Axial and Central Chirality" *ACS Catal.* ***15***, (2025): 1147–1157, https://doi.org/10.1021/acscatal.4c07172.

[4] W. Xiong, X. Jiang, W.-C. Wang, Y. Cheng, L.-Q. Lu, K. Gao, W.-J. Xiao, "Dynamic Kinetic Reductive Conjugate Addition for Construction of Axial Chirality Enabled by Synergistic Photoredox/Cobalt Catalysis" *J. Am. Chem. Soc.* ***145***, (2023): 7983–7991, https://doi.org/10.1021/jacs.2c13538.

[5] P.-Y. Jiang, S. Wu, G.-J. Wang, S.-H. Xiang, B. Tan, "Synthesis of Axially Chiral QUINAP Derivatives by Ketone-Catalyzed Enantioselective Oxidation" *Angew. Chem. Int. Ed.* ***62***, (2023): e202309272, https://onlinelibrary.wiley.com/doi/abs/10.1002/anie.202309272.

[6] X. W. Chen, C. Li, Y. Y. Gui, J. P. Yue, Q. Zhou, L. L. Liao, J. W. Yang, J. H. Ye, D. G. Yu, "Atropisomeric Carboxylic Acids Synthesis via Nickel-Catalyzed Enantioconvergent Carboxylation of Aza-Biaryl Triflates with CO2" *Angew. Chem. Int. Ed.* ***63***, (2024): e202403401, https://doi.org/10.1002/anie.202403401.

[7] X. Ma, M. Tan, L. Li, Z. Zhong, P. Li, J. Liang, Q. Song, "Ni-catalysed assembly of axially chiral alkenes from alkynyl tetracoordinate borons via 1,3-metallate shift" *Nat. Chem.* ***16***, (2024): 42–53, https://doi.org/10.1038/s41557-023-01396-7.

[8] N. Ishida, M. Narumi, M. Murakami, "Synthesis of Azaaromatic□Borane Intramolecular Complexes by Palladium-Catalyzed Reaction of Azaaromatic Halides with Alkynyl(triaryl)borates" *HeIv. Chim. Acta* ***95***, (2012): 2474–2480, https://doi.org/10.1002/hlca.201200554.

[9] F. He, Z. Sun, X. Zhang, Z. Long, Q. Zhao, L. Hu, L. Fu, H. Wang, "Iridium-Catalyzed Enantioselective Allylation of Alkynylboronates to Access Chiral 1,4-Dienes" *Angew. Chem. Int. Ed.* ***65***, (2026): e23810, https://doi.org/10.1002/anie.202523810.

[10] L. Wei, Z. Guo, J. L. Tyler, V. K. Aggarwal, "Catalytic Asymmetric 1,2-Migration/Allylation of

Alkynyl Boronate Complexes: A Modular Route to Enantioenriched Skipped 1,4 Dienes" *J. Am. Chem. Soc.* ***148***, (2026), 106–113, https://doi.org/10.1021/jacs.5c19143

[11] K. Zhang, M. Oestreich, "Atroposelective Synthesis of Azobenzenes by Palladium-Catalyzed Cross-Coupling of Racemic Biaryl Triflates and Diazenyl Pronucleophiles" *J. Am. Chem. Soc.* ***147***, (2025): 32329–32334, https://doi.org/10.1021/jacs.5c09097.

[12] D.-S. Zheng, W.-W. Zhang, Q. Gu, S.-L. You, "Rh(III)-Catalyzed Atroposelective C–H Iodination of 1-Aryl Isoquinolines" *ACS Catal.* ***13***, (2023): 5127–5134, https://doi.org/10.1021/acscatal.3c00751.

[13] P. Ramírez-López, A. Ros, A. Romero-Arenas, J. Iglesias-Sigüenza, R. Fernández, J. M. Lassaletta, "Synthesis of IAN-type N,N-Ligands via Dynamic Kinetic Asymmetric Buchwald–Hartwig Amination" *J. Am. Chem. Soc.* ***138***, (2016): 12053–12056, https://doi.org/10.1021/jacs.6b07972.
