# Supplementary material for: Atroposelective Access to π‐Conjugated 1,2‐Azaborepines Enabled by Palladium‐Catalyzed Cyclization of N‐Heterobiaryls with Alkynylboronates
Source: Adv Sci (Weinh). 2026 May 19:e75755. Online ahead of print. doi: 10.1002/advs.75755 (PMC13335858; doi:10.1002/advs.75755)

checkCIF/PLATON report

Structure factors have been supplied for datablock(s) 20250724b

THIS REPORT IS FOR GUIDANCE ONLY. IF USED AS PART OF A REVIEW PROCEDURE FOR PUBLICATION, IT SHOULD NOT REPLACE THE EXPERTISE OF AN EXPERIENCED CRYSTALLOGRAPHIC REFEREE.

No syntax errors found. CIF dictionary Interpreting this report

Datablock: 20250724b

```
Bond precision:   C-C = 0.0041 A          Wavelength=1.54178

Cell:              a=8.7555(2)      b=14.1862(3)      c=25.5392(6)
                   alpha=90         beta=90           gamma=90
Temperature:       298 K

                Calculated                    Reported
Volume          3172.16(12)                   3172.15(12)
Space group     P 21 21 21                    P 21 21 21
Hall group      P 2ac 2ab                     P 2ac 2ab
Moiety formula  C42 H34 B N                   C42 H34 B N
Sum formula     C42 H34 B N                   C42 H34 B N
Mr              563.51                        563.51
Dx,g cm-3       1.180                         1.180
Z               4                             4
Mu (mm-1)       0.506                         0.506
F000            1192.0                        1192.0
F000'           1195.03
h,k,lmax        10,17,31                      10,17,31
Nref            6285[ 3554]                   6182
Tmin,Tmax       0.766,0.801                   0.438,0.754
Tmin'           0.766

Correction method= # Reported T Limits: Tmin=0.438 Tmax=0.754
AbsCorr = MULTI-SCAN

Data completeness= 1.74/0.98       Theta(max)= 72.447

                                                       wR2(reflections)=
R(reflections)= 0.0409( 5698)                          0.1119( 6182)

S = 1.086                  Npar= 502
```

The following ALERTS were generated. Each ALERT has the format
test-name_ALERT_alert-type_alert-level.
Click on the hyperlinks for more details of the test.

Alert level C

```
PLAT089_ALERT_3_C Poor Data / Parameter Ratio (Zmax < 18) ........       7.05 Note
PLAT340_ALERT_3_C Low Bond Precision on  C-C Bonds ...............    0.00412 Ang.
PLAT911_ALERT_3_C Missing FCF Refl Between Thmin & STh/L=    0.600          9 Report
                1  1  0,   2  1  0,  10  5  0,   0  1  2,   0  2  3,   0  0  4,
                1  2  4,   0  0  6,   9  0 14,
```

Alert level G

```
PLAT002_ALERT_2_G Number of Distance or Angle Restraints on AtSite         24 Note
PLAT003_ALERT_2_G Number of Uiso or U(i,j)  Restrained non-H-Atoms         24 Report
PLAT174_ALERT_4_G The CIF-Embedded .res File Contains FLAT Records          1 Report
PLAT175_ALERT_4_G The CIF-Embedded .res File Contains SAME Records          1 Report
PLAT178_ALERT_4_G The CIF-Embedded .res File Contains SIMU Records          1 Report
PLAT188_ALERT_3_G A Non-default SIMU Restraint Value has been used     0.0100 Report
PLAT189_ALERT_3_G A Non-default SAME Restraint Value for First Par     0.0100 Report
PLAT189_ALERT_3_G A Non-default SAME Restraint Value for SecondPar     0.0100 Report
PLAT230_ALERT_2_G Hirshfeld Test Diff for    C35      --C36      .        5.7 s.u.
PLAT301_ALERT_3_G Main Residue  Disorder .............(Resd    1)         25% Note
PLAT860_ALERT_3_G Number of Least-Squares Restraints ............         406 Note
PLAT910_ALERT_3_G Missing FCF Reflection(s) Below Theta(Min)[Deg]=       3.56 Note
                0  0  2,
PLAT912_ALERT_4_G Missing # of FCF Reflections Above STh/L=  0.600          7 Note
PLAT913_ALERT_3_G Missing # of Very Strong Reflections in FCF ....          3 Note
                2  1  0,   0  2  3,   1  2  4,
PLAT969_ALERT_5_G The 'Henn et al.' R-Factor-gap value ..........      2.943 Note
              Predicted wR2: Based on SigI**2  3.80 or SHELX Weight 10.30
PLAT978_ALERT_2_G Number C-C Bonds with Positive Residual Density.          0 Info
```

0 **ALERT level A** = Most likely a serious problem - resolve or explain
0 **ALERT level B** = A potentially serious problem, consider carefully
3 **ALERT level C** = Check. Ensure it is not caused by an omission or oversight
16 **ALERT level G** = General information/check it is not something unexpected

0 ALERT type 1 CIF construction/syntax error, inconsistent or missing data
4 ALERT type 2 Indicator that the structure model may be wrong or deficient
10 ALERT type 3 Indicator that the structure quality may be low
4 ALERT type 4 Improvement, methodology, query or suggestion
1 ALERT type 5 Informative message, check

It is advisable to attempt to resolve as many as possible of the alerts in all categories. Often the minor alerts point to easily fixed oversights, errors and omissions in your CIF or refinement strategy, so attention to these fine details can be worthwhile. In order to resolve some of the more serious problems it may be necessary to carry out additional measurements or structure refinements. However, the purpose of your study may justify the reported deviations and the more serious of these should normally be commented upon in the discussion or experimental section of a paper or in the "special_details" fields of the CIF. checkCIF was carefully designed to identify outliers and unusual parameters, but every test has its limitations and alerts that are not important in a particular case may appear. Conversely, the absence of alerts does not guarantee there are no aspects of the results needing attention. It is up to the individual to critically assess their own results and, if necessary, seek expert advice.

Publication of your CIF in IUCr journals

A basic structural check has been run on your CIF. These basic checks will be run on all CIFs submitted for publication in IUCr journals (*Acta Crystallographica*, *Journal of Applied Crystallography*, *Journal of Synchrotron Radiation*); however, if you intend to submit to *Acta Crystallographica Section C* or *E* or *IUCrData*, you should make sure that full publication checks are run on the final version of your CIF prior to submission.

Publication of your CIF in other journals

Please refer to the *Notes for Authors* of the relevant journal for any special instructions relating to CIF submission.

PLATON version of 04/06/2025; check.def file version of 30/05/2025

Datablock 20250724b - ellipsoid plot

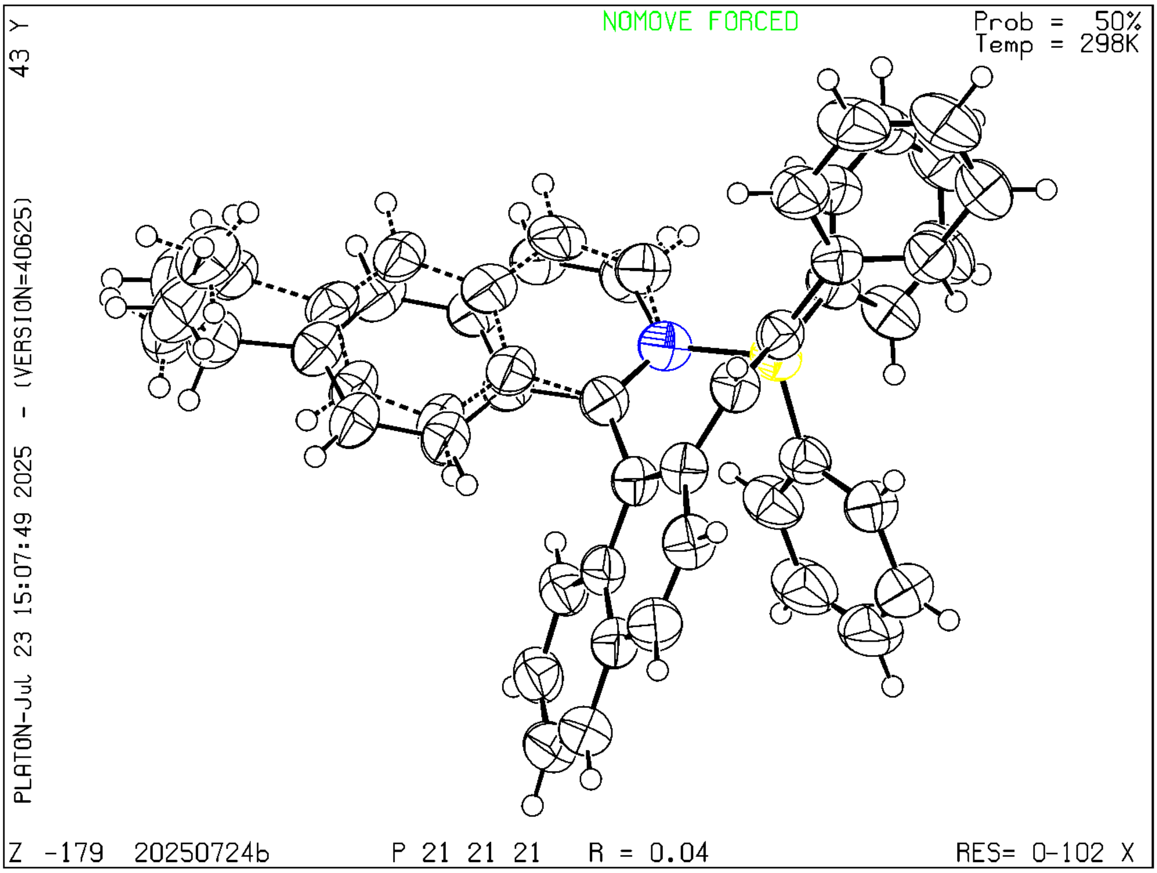

Supplement: Supplementary file 2 — Supporting File 2: advs75755‐sup‐0002‐cif.zip. [file ADVS-9999-e75755-s001.zip › advs75755-sup-0002-cif/checkcif for compound 4.pdf]
