# Supplementary material for: Atroposelective Access to π‐Conjugated 1,2‐Azaborepines Enabled by Palladium‐Catalyzed Cyclization of N‐Heterobiaryls with Alkynylboronates
Source: Adv Sci (Weinh). 2026 May 19:e75755. Online ahead of print. doi: 10.1002/advs.75755 (PMC13335858; doi:10.1002/advs.75755)

checkCIF/PLATON report

Structure factors have been supplied for datablock(s) 20250801b

THIS REPORT IS FOR GUIDANCE ONLY. IF USED AS PART OF A REVIEW PROCEDURE FOR PUBLICATION, IT SHOULD NOT REPLACE THE EXPERTISE OF AN EXPERIENCED CRYSTALLOGRAPHIC REFEREE.

No syntax errors found. CIF dictionary Interpreting this report

Datablock: 20250801b

```
Bond precision:   C-C = 0.0051 A          Wavelength=0.71073

Cell:             a=12.1726(12)      b=13.5587(14)      c=21.354(3)
                  alpha=90           beta=90            gamma=90
Temperature:      298 K
```

	Calculated	Reported
Volume	3524.4(7)	3524.3(7)
Space group	P 21 21 21	P 21 21 21
Hall group	P 2ac 2ab	P 2ac 2ab
Moiety formula	C45 H34 B N S2	C45 H34 B N S2
Sum formula	C45 H34 B N S2	C45 H34 B N S2
Mr	663.66	663.66
Dx,g cm-3	1.251	1.251
Z	4	4
Mu (mm-1)	0.185	0.185
F000	1392.0	1392.0
F000'	1393.44	
h,k,lmax	15,17,27	15,17,27
Nref	8106[4509]	8067
Tmin,Tmax	0.927,0.935	0.530,0.746
Tmin'	0.927	

```
Correction method= # Reported T Limits: Tmin=0.530 Tmax=0.746
AbsCorr = MULTI-SCAN

Data completeness= 1.79/1.00       Theta(max)= 27.500

R(reflections)= 0.0509( 5580)        wR2(reflections)= 0.1173( 8067)

S = 1.022                Npar= 488
```

The following ALERTS were generated. Each ALERT has the format
test-name_ALERT_alert-type_alert-level.
Click on the hyperlinks for more details of the test.

Alert level C

```
PLAT234_ALERT_4_C Large Hirshfeld Difference S2       --C45      .       0.16 Ang.
PLAT242_ALERT_2_C Low    'MainMol' Ueq as Compared to Neighbors of        C42 Check
PLAT340_ALERT_3_C Low Bond Precision on  C-C Bonds ...............    0.00507 Ang.
PLAT906_ALERT_3_C Large K Value in the Analysis of Variance ......      2.032 Check
PLAT910_ALERT_3_C Missing FCF Reflection(s) Below Theta(Min)[Deg]=       2.54 Note
                1  1  0,   1  0  1,   0  1  1,   1  1  1,   0  0  2,   0  1  2,
PLAT911_ALERT_3_C Missing FCF Refl Between Thmin & STh/L=    0.600          2 Report
                2  2  0,   3  2  0,
```

Alert level G

```
PLAT002_ALERT_2_G Number of Distance or Angle Restraints on AtSite         12 Note
PLAT003_ALERT_2_G Number of Uiso or U(i,j)  Restrained non-H-Atoms         12 Report
PLAT172_ALERT_4_G The CIF-Embedded .res File Contains DFIX Records          4 Report
PLAT174_ALERT_4_G The CIF-Embedded .res File Contains FLAT Records          1 Report
PLAT175_ALERT_4_G The CIF-Embedded .res File Contains SAME Records          1 Report
PLAT176_ALERT_4_G The CIF-Embedded .res File Contains SADI Records          1 Report
PLAT178_ALERT_4_G The CIF-Embedded .res File Contains SIMU Records          1 Report
PLAT187_ALERT_4_G The CIF-Embedded .res File Contains RIGU Records          1 Report
PLAT188_ALERT_3_G A Non-default SIMU Restraint Value has been used     0.0200 Report
PLAT189_ALERT_3_G A Non-default SAME Restraint Value for First Par     0.0100 Report
PLAT190_ALERT_3_G A Non-default RIGU Restraint Value for First Par     0.0020 Report
PLAT191_ALERT_3_G A Non-default SADI Restraint Value has been used     0.0100 Report
PLAT301_ALERT_3_G Main Residue  Disorder ..............(Resd    1)        10% Note
PLAT411_ALERT_2_G Short Inter H...H Contact  H37      ..H44A     .       2.01 Ang.
                                          1/2-x,2-y,1/2+z  =      2_575 Check
PLAT860_ALERT_3_G Number of Least-Squares Restraints .............        241 Note
PLAT912_ALERT_4_G Missing # of FCF Reflections Above STh/L=  0.600         14 Note
PLAT933_ALERT_2_G Number of HKL-OMIT Records in Embedded .res File          3 Note
               -1 -1 -2,   1  1  2,   2  2  0,
PLAT969_ALERT_5_G The 'Henn et al.' R-Factor-gap value ..........       2.481 Note
              Predicted wR2: Based on SigI**2  4.73 or SHELX Weight 11.47
PLAT978_ALERT_2_G Number C-C Bonds with Positive Residual Density.          0 Info
```

0 **ALERT level A** = Most likely a serious problem - resolve or explain
0 **ALERT level B** = A potentially serious problem, consider carefully
6 **ALERT level C** = Check. Ensure it is not caused by an omission or oversight
19 **ALERT level G** = General information/check it is not something unexpected

0 ALERT type 1 CIF construction/syntax error, inconsistent or missing data
6 ALERT type 2 Indicator that the structure model may be wrong or deficient
10 ALERT type 3 Indicator that the structure quality may be low
8 ALERT type 4 Improvement, methodology, query or suggestion
1 ALERT type 5 Informative message, check

It is advisable to attempt to resolve as many as possible of the alerts in all categories. Often the minor alerts point to easily fixed oversights, errors and omissions in your CIF or refinement strategy, so attention to these fine details can be worthwhile. In order to resolve some of the more serious problems it may be necessary to carry out additional measurements or structure refinements. However, the purpose of your study may justify the reported deviations and the more serious of these should normally be commented upon in the discussion or experimental section of a paper or in the "special_details" fields of the CIF. checkCIF was carefully designed to identify outliers and unusual parameters, but every test has its limitations and alerts that are not important in a particular case may appear. Conversely, the absence of alerts does not guarantee there are no aspects of the results needing attention. It is up to the individual to critically assess their own results and, if necessary, seek expert advice.

Publication of your CIF in IUCr journals

A basic structural check has been run on your CIF. These basic checks will be run on all CIFs submitted for publication in IUCr journals (*Acta Crystallographica*, *Journal of Applied Crystallography*, *Journal of Synchrotron Radiation*); however, if you intend to submit to *Acta Crystallographica Section C* or *E* or *IUCrData*, you should make sure that full publication checks are run on the final version of your CIF prior to submission.

Publication of your CIF in other journals

Please refer to the *Notes for Authors* of the relevant journal for any special instructions relating to CIF submission.

PLATON version of 04/06/2025; check.def file version of 30/05/2025

Datablock 20250801b - ellipsoid plot

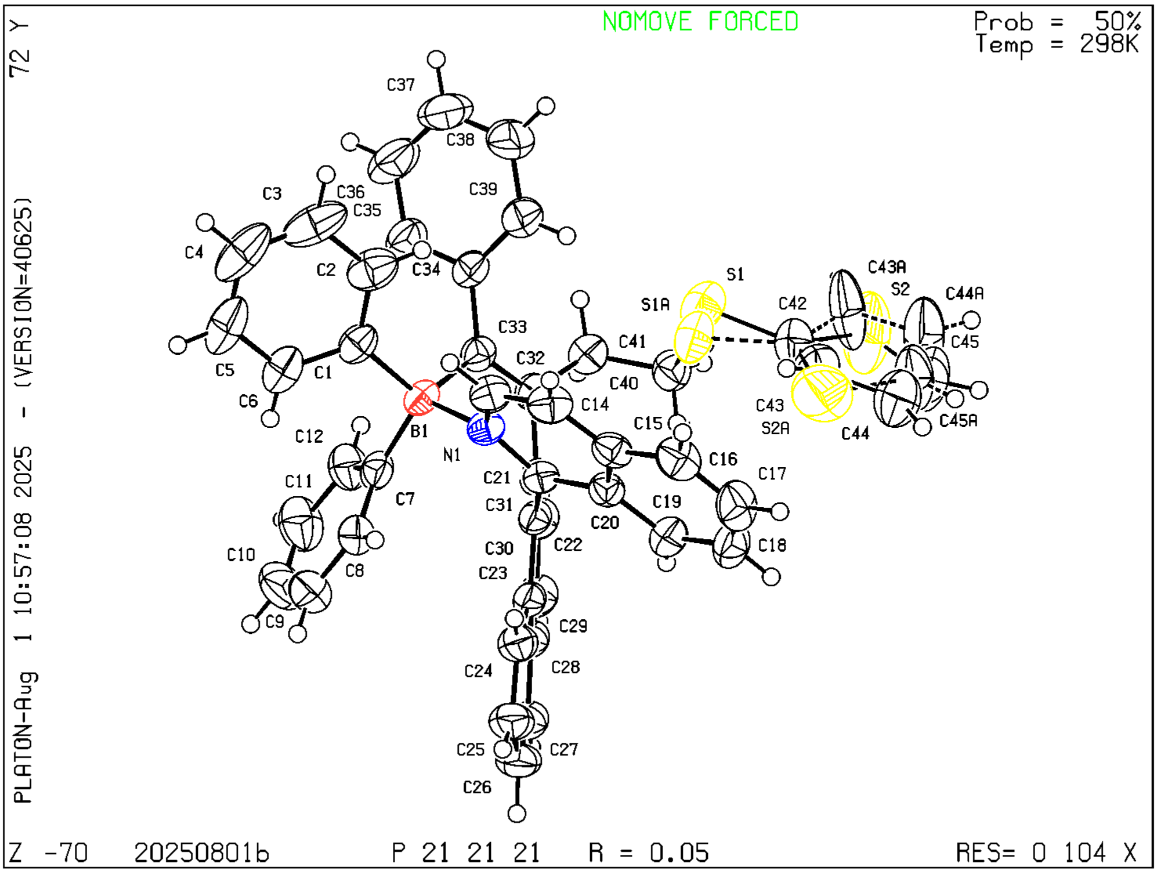

Supplement: Supplementary file 2 — Supporting File 2: advs75755‐sup‐0002‐cif.zip. [file ADVS-9999-e75755-s001.zip › advs75755-sup-0002-cif/checkcif for compound 46.pdf]
